# A multi-scale approach reveals that NF-κB cRel enforces a B-cell decision to divide

**DOI:** 10.15252/msb.20145554

**Published:** 2015-02-13

**Authors:** Maxim N Shokhirev, Jonathan Almaden, Jeremy Davis-Turak, Harry A Birnbaum, Theresa M Russell, Jesse A D Vargas, Alexander Hoffmann

**Affiliations:** 1 Department of Chemistry and Biochemistry, Signaling Systems Laboratory, UCSDLa Jolla, CA, USA; 2 San Diego Center for Systems Biology, UCSDLa Jolla, CA, USA; 3 Bioinformatics and Systems Biology Graduate Program, UCSDLa Jolla, CA, USA; 4 Biological Sciences Graduate Program, UCSDLa Jolla, CA, USA; 5 Institute for Quantitative and Computational BiosciencesLos Angeles, CA, USA; 6 Department of Microbiology, Immunology and Molecular Genetics, UCLALos Angeles, CA, USA; 7 Fluidigm CorporationSouth San Francisco, CA, USA

**Keywords:** apoptosis, B-lymphocyte, cell cycle, cell fate decision, NF-κB cRel

## Abstract

Understanding the functions of multi-cellular organs in terms of the molecular networks within each cell is an important step in the quest to predict phenotype from genotype. B-lymphocyte population dynamics, which are predictive of immune response and vaccine effectiveness, are determined by individual cells undergoing division or death seemingly stochastically. Based on tracking single-cell time-lapse trajectories of hundreds of B cells, single-cell transcriptome, and immunofluorescence analyses, we constructed an agent-based multi-modular computational model to simulate lymphocyte population dynamics in terms of the molecular networks that control NF-κB signaling, the cell cycle, and apoptosis. Combining modeling and experimentation, we found that NF-κB cRel enforces the execution of a cellular decision between mutually exclusive fates by promoting survival in growing cells. But as cRel deficiency causes growing B cells to die at similar rates to non-growing cells, our analysis reveals that the phenomenological decision model of wild-type cells is rooted in a biased race of cell fates. We show that a multi-scale modeling approach allows for the prediction of dynamic organ-level physiology in terms of intra-cellular molecular networks.

## Introduction

B-lymphocytes are central to immune responses as producers of antibodies and mediators of immunological memory. Upon recognition of specific antigens or pathogen-derived ‘danger’ signals, B cells may enter a proliferative program (Murphy *et al*, 2007). This physiological process can be recapitulated *ex vivo* using agonists of the B-cell receptor or Toll-like receptors (TLRs), which recognize specific pathogen-derived substances. Such agonists elicit a dynamic population response in which individual cells may undergo several rounds of cell division, exit the cell cycle and/or die by programmed cell death (Rawlings *et al*, 2012). Indeed, while the population response is generally robustly reproducible and predictable, the behavior of individual cells is seemingly stochastic. In each generation, only a fraction of cells divide, while others die, and the timing of division and death is highly variable, typically well-modeled by long-tailed log-normal distributions and resulting in a spectrum of many generations at any given time point after stimulation (Hawkins *et al*, 2007b, 2009).

Given the physiological and pathological importance of the B-cell response, the underlying biochemical processes involved in transducing receptor signals, cell growth, cell cycling, and programmed cell death by apoptosis have been well studied (recently reviewed in Browne, 2012; Gerondakis & Siebenlist, 2010; Link & Hurlin, 2014; Renault & Chipuk, 2013). Their involvement in B-cell expansion has been characterized by measuring population cell numbers or apoptotic cells, bulk replicative activity (by measuring DNA synthesis), or distributions of generational cell counts at given time points (by dye dilution studies coupled to FACS). For example, deficiency in the NF-κB transcription factor cRel was reported to result in a substantially reduced B-population response, due to deficiencies in cell-cycle entry and cell survival (Gerondakis *et al*, 1998; Grumont *et al*, 1998). Further, potential cRel-dependent mediators of these processes have been identified, such as the genes coding for CyclinD (Wang *et al*, 1996; Guttridge *et al*, 1999; Huang *et al*, 2001), Myc (Duyao *et al*, 1990), and Bcl_XL_ (Chen *et al*, 1999). Yet, how these functions coordinately produce the dynamics of the population response, the generation-specific distributions, or fate control at the individual cell level remains poorly understood.

Previous studies have shown that the time to the first division is substantially longer than that of subsequent divisions, and the timing of cell death is also generation dependent (Hawkins *et al*, 2009). Yet there are competing theories for how fate (i.e., whether the cell divides or dies) is determined. Some studies invoke a molecular race hypothesis, which posits that processes leading to cell division and apoptosis are proceeding concurrently within cells, with the faster of the two determining the outcomes (Hawkins *et al*, 2007b; Duffy *et al*, 2012); however, other observations support the notion that cells decide their fate prior to it being manifest (Hawkins *et al*, 2009; Shokhirev & Hoffmann, 2013). In particular, the cyton (Hawkins *et al*, 2007b) and fcyton (Shokhirev & Hoffmann, 2013) age- and generation-structured models describe lymphocyte population dynamics assuming a molecular race or decision between division and death, respectively. Further, it is unclear what the determinants are for the variability in timing which in the former but not the latter model underlies the variability of cell fate determination. Previous studies offer evidence that the inherent variability in timing of receptor-induced apoptosis of transformed liver cells is caused primarily by cell-to-cell variability in the steady state (Gaudet *et al*, 2012).

Recent advances in single-cell analysis and modeling render answers to these questions within reach. Flow cytometry and immunofluorescence microscopy, which provide snapshots of a few attributes of the cells within a population, may be complemented with single-cell mRNA sequencing, which provides transcriptome-wide measurements, and live cell microscopy, which provides longitudinal information at single-cell resolution; however, challenges in data analysis and integration persist. Interestingly, kinetic models that capture the dynamic control of molecular networks can function as platforms for data integration and provide a predictive understanding; for example, iterative experimental and modeling studies have delineated numerous negative and positive feedback loops that control the dynamics of NF-κB (Basak *et al*, 2012), or identified determinants of cell-cycle progression (Conradie *et al*, 2010) and cell death/survival fate decisions (Loriaux *et al*, 2013).

## Results

**Figure 1:**
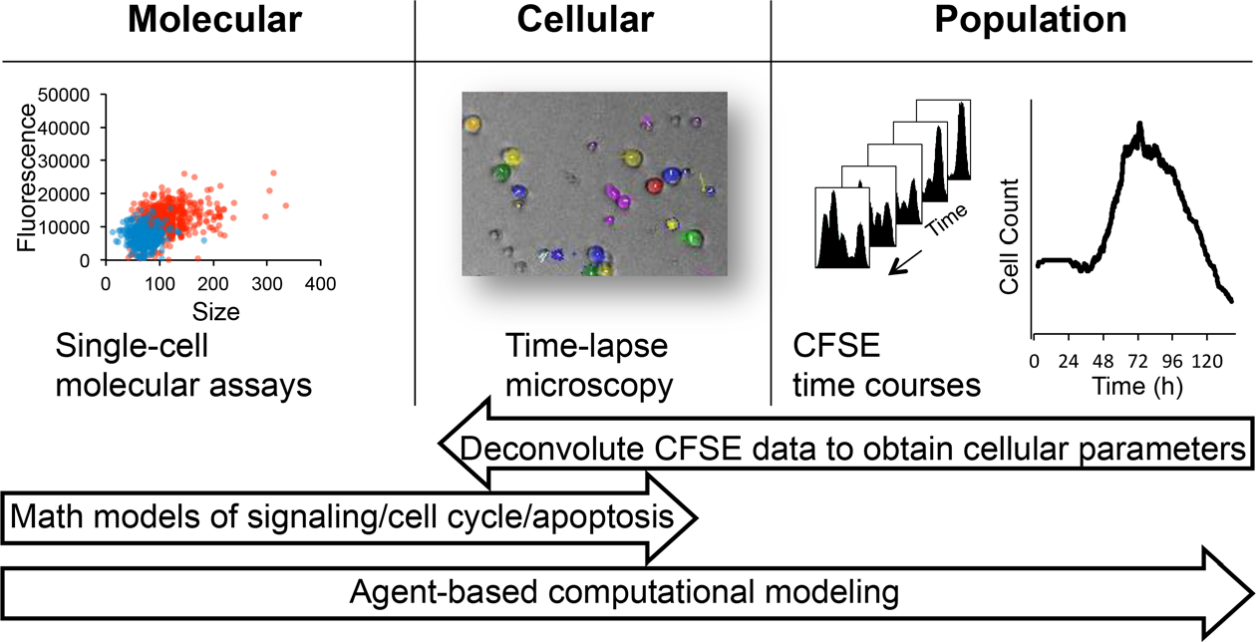
Developing a multi-scale understanding of the B-cell immune response We employed a multi-scale approach to studying the B-cell response. Time-lapse microscopy observations of B-cell populations revealed cellular growth trajectories, distribution of division and death time, as well as the fraction of cells responding in each generation. Single-cell molecular assays provided insights into the upregulation of key molecular players upon activation within individual cells. The number of cells in each generation was measured by the division tracking dye CFSE and deconvoluted into maximum-likelihood cellular parameters using the FlowMax computational tool. We used our observations to parameterize a multi-scale agent-based mathematical model consisting of established modules for signaling, apoptosis, and the cell cycle which allowed us to mechanistically study molecular perturbations on population dynamics.

Here, we aimed to construct a multi-modular mathematical model that accounts for B-cell population dynamics in terms of intra-cellular molecular network dynamics. Starting at the B-cell population scale, we employed carboxyfluorescein succinimidyl ester (CFSE) flow cytometry and live time-lapse microscopy tracking of cell lineages to characterize the model topology and parameters at the cell biological scale. Starting at the molecular network scale, we used single-cell RNAseq and quantitative immunofluorescence to characterize the connections between several regulatory molecular network modules (Fig1).

### Time-lapse microscopy reveals generation-specific single-cell behavior

**Figure 2:**
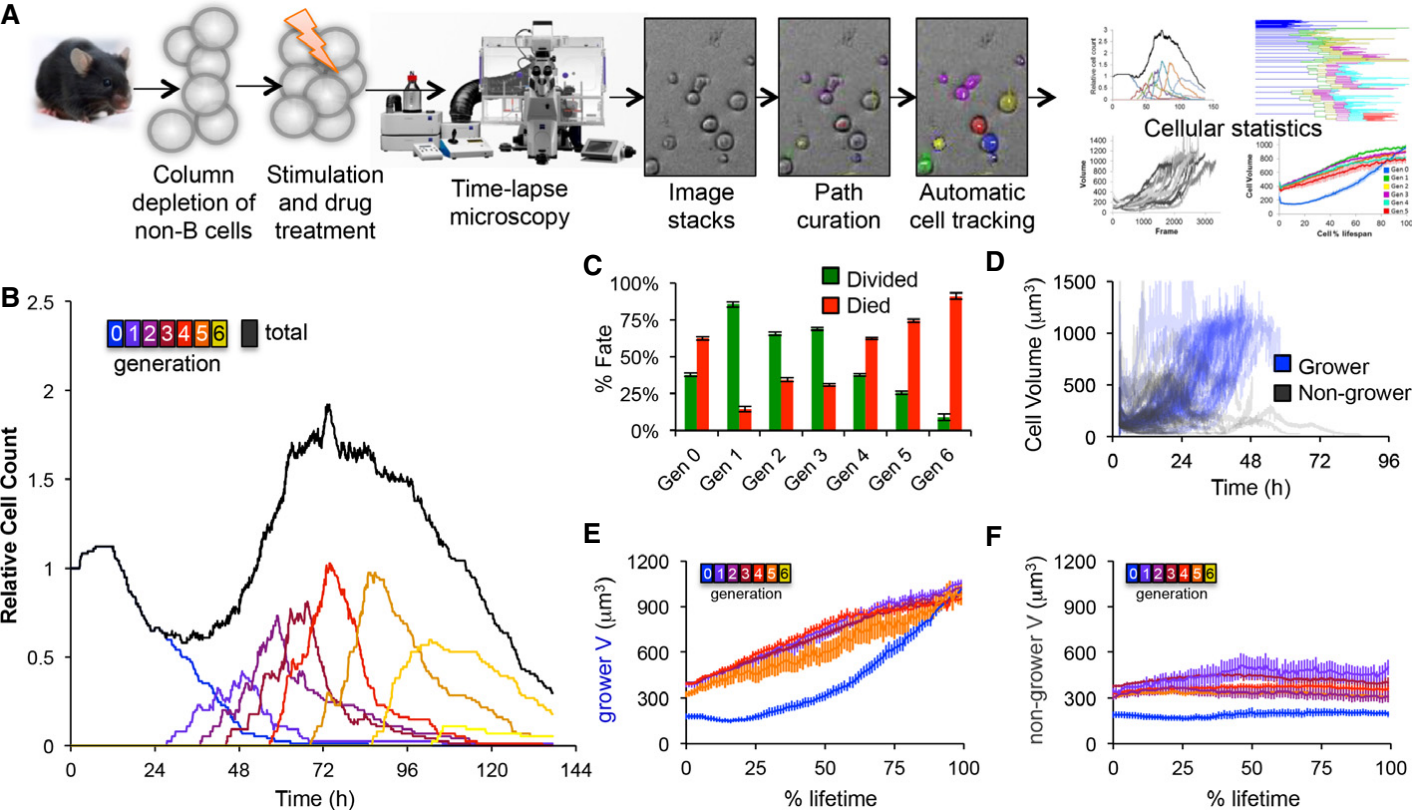
Time-lapse microscopy reveals two distinct generation-dependent growth patterns for B cells A Overview of the time-lapse microscopy experimental and analysis pipeline. B cells were purified from mouse spleen, stimulated with TLR9 agonist CpG, imaged on an environmentally controlled microscope for 6 days, and tracked using a semi-automated tracking tool to quantify generation-dependent cell statistics.

In order to obtain cell lineage information that accounts for the population response, we tracked 1,295 live primary B cells using a time-lapse microscopy pipeline (Fig2A). We developed a semi-automated image analysis method, combining the advantages of computational automation and human input to minimize errors (see Materials and Methods). Analysis of wild-type B cells responding to high CpG stimulation confirmed the expected population expansion followed by a contraction period (Fig2B). After cells that died from mechanical death in the initial phase (Hawkins *et al*, 2007a) were filtered out (Supplementary Fig S1A and B), we found that approximately 38% of the starting, ‘generation 0’, cells divided; then, 85% of generation 1 cells divided with subsequent generations, showing a steady decrease in this fraction such that only 9% of cells divided in generation 6 (Fig2C). To quantify the cellular response, we classified cell size trajectories into two categories: (i) cells that grew by at least 350 μm^3^ (representing at least two standard deviations on average, Supplementary Fig S1C) or reached a final size of at least 800 μm^3^ (based on the bimodal size distribution (Supplementary Fig S1D) and to ensure that large generation 1+ cells are included), dubbed ‘growers’ and (ii) cells that did not meet these criteria, dubbed ‘non-growers’ (Fig2D). To test the sensitivity of the growth threshold, we repeated the quantification with a 25% lower and higher growth threshold, revealing that few cells exhibited ambiguous growth (Supplementary Fig S1E). Averaging the growth trajectories of ‘growers’ (Fig2E) and ‘non-growers’ (Fig2F) in each generation normalized by percent cell lifespan revealed that progenitors (generation 0) that grew exhibited a growth delay followed by rapid growth to approximately fivefold their starting size, while generation 1+ growers did not exhibit the delay phase, and started growing immediately after mitosis. Furthermore, ‘grower’ cells generally grew to the same size on average in all generations except prior to their final division. While ‘non-growers’ by definition did not exhibit significant growth (as defined above), they nevertheless typically exhibited some growth.

B Generational cell counts relative to initial count.

C The observed fraction of cells dividing or dying in each generation. Error bars = 1/*n*, where *n* = 85, 55, 93, 103, 125, 90, 45 for generations 0–6, respectively.

D Growth trajectories of generation 0 cells that grew by more than 350 μm^3^ or ended with a volume of at least 800 μm^3^ (blue) and trajectories of generation 0 cells that did not end with a large volume (black).

E, F Cell size trajectories as a function of % lifetime for growers (E) and non-growers (F) in each generation (colors as in B). Error bars are SD, with *n* = 34, 44, 60, 54, 40, 15, 2 growing cells, 51, 11, 33, 49, 85, 75, 43 non-growing cells in generations 0–6, respectively.

### Distinguishing between race and decision models in cell fate determination

**Figure 3:**
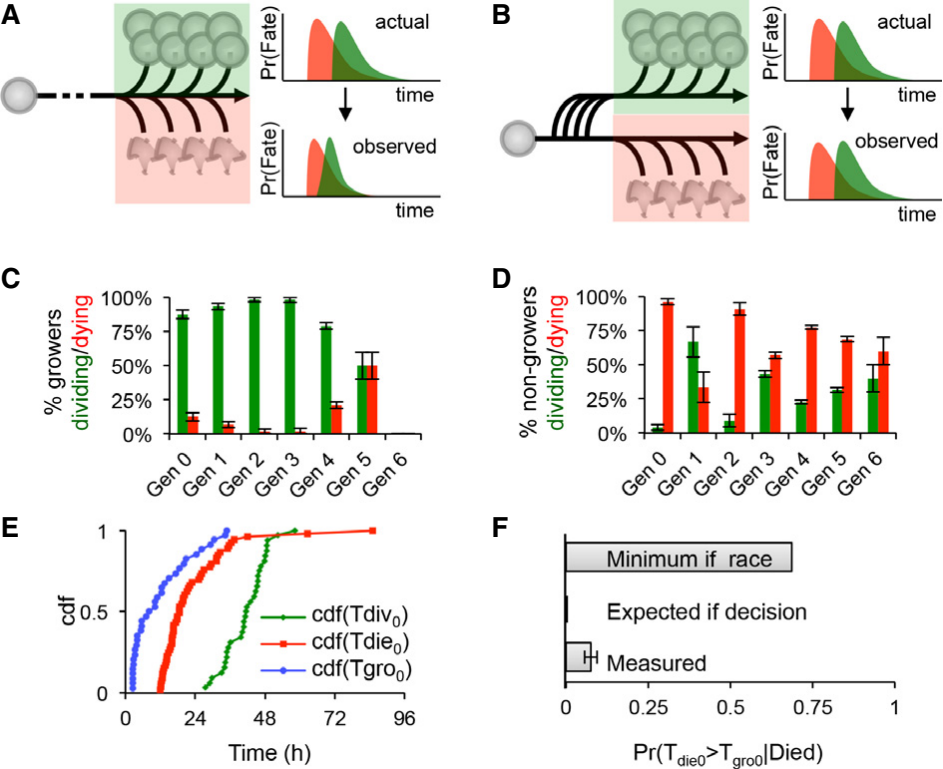
B cells decide to divide or die and are protected from the alternate fate A Flowchart depicting the scenario in which the cell fate of growing cells is determined by a race between division (green) and death (red); hypothetical division and death time distributions before and after mutual censorship are shown on the right.

To further characterize the underlying cellular mechanisms, we next tested whether cell cycle and apoptosis were parallel racing processes (Fig3A), as previously suggested (Duffy *et al*, 2012), or whether the growth phase was indicative of a prior decision to assume the division fate instead of the death fate (Fig3B). For each generation, we counted the fraction of ‘growers’ that divided and died within a 24-h period: 12–36 h in generation 0 or 0–24 h in generations 1+ (Fig3C), as well as the fraction of ‘non-growers’ that divided and died within the same periods for each generation (Fig3D). Our results indicate that virtually all ‘growers’ in the first four generations divided, supporting the notion of an early decision that predisposes B cells to a particular fate (Fig3B). Interestingly, following the first generation, there was a significant fraction of cells that divided that had been classified as ‘non-growers’; however, such poor growth almost always occurred in the last division (Supplementary Fig S2). To further test this important distinction, we noted the time point at which growth starts (Tgro), the time to division (Tdiv), and time to death (Tdie) of progenitor cells and calculated the expected lower-bound probability that a dying cell would have grown, provided a ‘molecular race’ or ‘decision’ model (Fig3E and F). Our analysis revealed that even under relatively relaxed assumptions, the data are inconsistent with both processes occurring simultaneously in cells (i.e., race). A decision model, which commits cells to either fate, is more consistent with the observed behavior. In other words, because time to death is typically earlier than time to division (Fig3E), and because time to start growing, Tgro, is typically much earlier than Tdiv or Tdie, our analysis predicts most cells would grow prior to death if the two processes were indeed running in parallel as implied by the ‘race’ model.

B Flowchart depicting the scenario in which entering the growth phase or not represents an early commitment to one fate; the independent division (green) and death (red) time distributions are shown on the right.

C–F Analysis of response (growth), division, and death statistics for WT B cells. Error bars = 1/*n*. Measured generational probabilities that a growing (C) or non-growing (D) cell divided (green) or died (red) within a 24-h period (12–36 h for gen 0). Measured cumulative distributions (E) for the time to start growing (blue), time to divide (green), and time to die (red) of generation 0 cells. Distributions were used to calculate the lower bound expected probability that a dying cell had started to grow (F) assuming a molecular race between fates or an early commitment to one fate, as compared to the measured probability. For further details, please see Supplementary Methods.

Next, we tested this hypothesis with an alternate method, using computational deconvolution of flow cytometric measurements of the generational populations at specific time points (Supplementary Fig S3A). CFSE-stained B cells were stimulated with CpG, and fluorescence histograms indicative of each generation were analyzed by the software tool FlowMax (Shokhirev & Hoffmann, 2013) to identify maximum-likelihood cellular parameters. Employing either the cyton model (which assumes that responding cells may die, Supplementary Fig S3B) or the fcyton model (which assumes that they do not, Supplementary Fig S3C), we asked which derived cellular parameters best agreed with those observed by time-lapse microscopy (Supplementary Fig S3D). While both models accurately fit the CFSE time course (Supplementary Fig S3E), the race (cyton) model requires a much longer distribution for Tdie_0_ than the decision (fcyton) model (Supplementary Fig S3F); shorter Tdie_0_ parameters are more consistent with the experimental microscopy dataset.

While our results suggest that cell fate is determined early, it is unclear what contributes to the cell-to-cell variability. Recent studies have shown that extrinsic variability in cell states rather than intrinsic signaling noise can account for variability in cell fate decision in mammalian cells (Spencer *et al*, 2009, 2013; Lee *et al*, 2010). As recently divided sibling cells have more similar cell states, we determined whether fate and timing are more correlated between related cells (Supplementary Fig S4). Indeed, sister cells were observed to undergo the same fate approximately 90% of the time, while cousin cells were more likely to experience different fates in all generations (Supplementary Fig S4A). Further, the timing of the decision process, interdivision time, and to a smaller extent lifespan were significantly correlated between sister cells: Pr(ΔTgro ≤ 4 h) = 0.90, *R*^2^ = 0.74, and *R*^2^ = 0.39, respectively (Supplementary Fig S4B–D). Furthermore, the correlations decreased with a subsequent division (i.e., between cousins): Pr(ΔTgro ≤ 4 h) = 0.77, *R*^2^ = 0.44, and *R*^2^ = 0.38, respectively, consistent with mixing times on the order of hours to days, more consistent with variability in molecular network states rather than genetic or epigenetic sources of cell-to-cell variability (Spencer *et al*, 2009).

### Molecular determinants of cell fate decision processes

**Figure 4:**
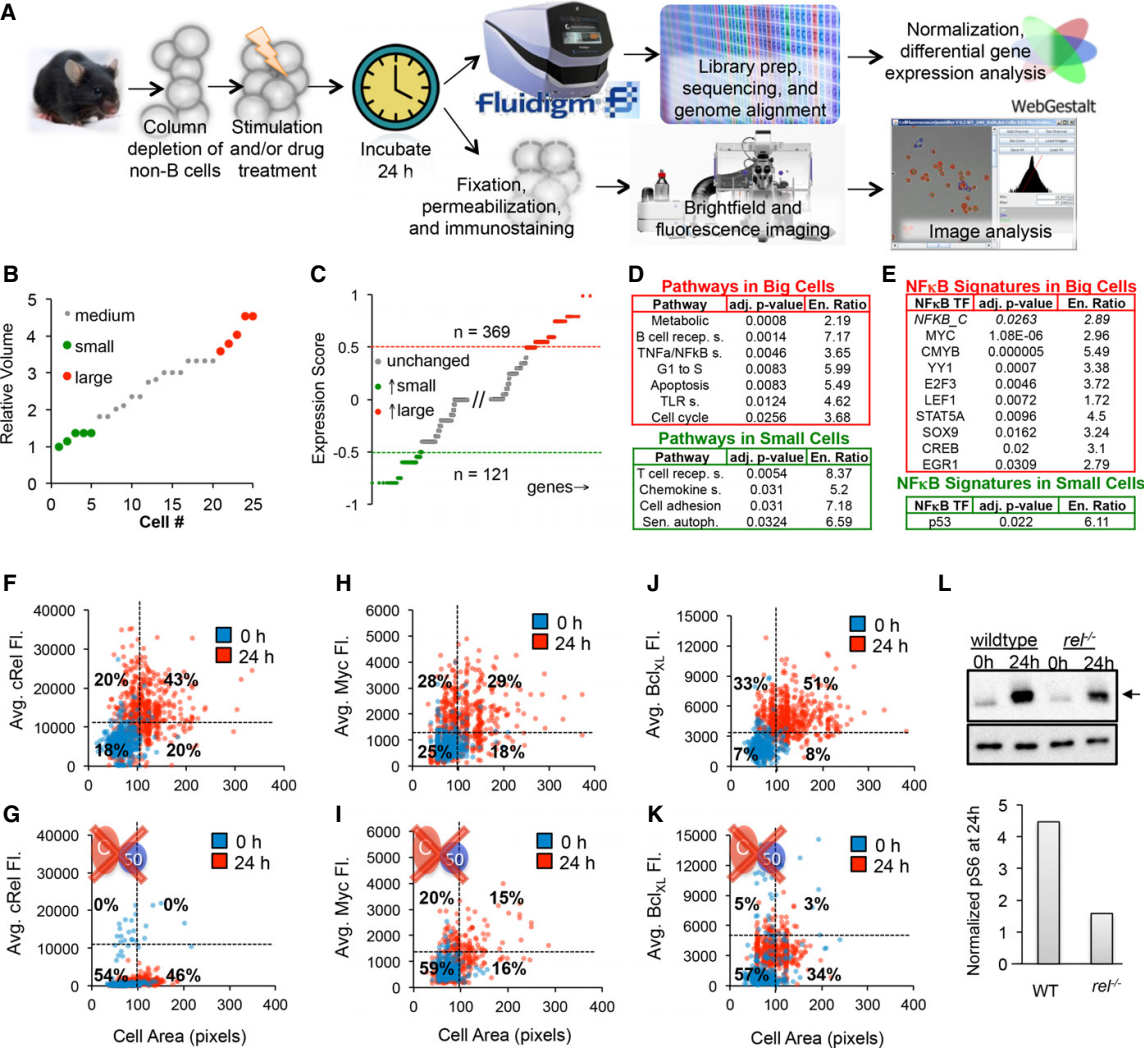
Molecular assays suggest that NF-κB enforces an upstream fate decision A–L Naïve purified B cells were stimulated with 250 nM CpG for 24 h and analyzed using single-cell RNA sequencing (A top). Five small and five large B cells were captured in a microfluidics chip (B), and their transcriptomes were sequenced to reveal sets of genes typically upregulated in big cells (C, red) or small cells (C, green). Pathway analysis on genes upregulated in large B cells (D, top) and small cells (D, bottom) was performed. (E) Transcription factor motif enrichment analysis on the genes upregulated in large cells (E, top) and small cells (E, bottom) was performed and filtered to show only significantly upregulated (*P* < 0.05) and known NF-κB target genes or NF-κB itself. NF-κB cRel abundances of purified naïve B cells stimulated with 250 nM CpG for 24 h were obtained by quantifying average fluorescence in fixed B cells stained with anti-cRel antibody conjugated to fluorophore, or anti-Bcl_XL_ antibody bound to a fluorescent secondary antibody (A bottom). The 0-h average fluorescence was used to determine significant upregulation of NF-κB cRel (F), growth regulator Myc (H), and anti-apoptotic regulator Bcl_XL_ (J) at 24 h (*P* < 0.05). Immunoblot for p-S6 (arrow), a downstream target of mTORc1, with anti-tubulin control after 24 h CpG stimulation in WT and cRel-deficient B cells and gel quantification is shown (L). NF-κB cRel-deficient cells were used to approximate the technical noise (G) or dependence of Myc (I), Bcl_XL_ (K), and mTORc1 (L) on NF-κB cRel. Quadrants in (F–K) indicate fraction of cells at 24 h compared to 0 h. Growth was manually defined as a cell area > 100 pixels to avoid cell selection bias in images.

To characterize the molecular connections that underlie fate decision processes, we turned to single-cell molecular assays (Fig4A). Following CpG stimulation of B cells for 24 h, we sequenced the transcriptomes of five large and five small cells using a single-cell autoprep system, which allowed us to image and measure the size of individual B cells trapped in a microfluidic chip (Fig4B). After normalizing transcript counts to RNA spike-in controls, we identified 369 upregulated and 121 downregulated genes in large versus small cells (Fig4C). Using pathway enrichment tools, we identified pathways that were significantly upregulated in large cells, including metabolism, the control of apoptosis and the G1-to-S transition, and NF-κB, a known key regulator of B-cell expansion (Fig4D). Further, we performed a transcription factor enrichment analysis on the upregulated and downregulated gene sets and found that binding motifs of nine transcription factors that are known NF-κB target genes, as well as NF-κB itself, were enriched among the genes upregulated in big cells (Fig4E), while p53 was the only known NF-κB target gene transcription factor enriched in the set of genes downregulated in big cells.

Next, for immunofluorescence, we stained stimulated B cells for cRel, and measured average fluorescence as a function of cell area (FigF). We found that compared to a 0 h control, B cells were larger (63% of cells) and had higher cRel abundance (63% of cells) after 24 h of stimulation. Furthermore, 68% of large cells had upregulated cRel at 24 h. To confirm the specificity of our analysis, we showed that cRel-deficient B cells had no detectable cRel fluorescence at 24 h (Fig4G). Similarly to NF-κB cRel, approximately 50% of cells showed significantly increased levels of NF-κB RelA after 24 h, with approximately 56% of large cells showing increased NF-κB RelA abundance after 24 h of stimulation (Supplementary Fig S5A). Staining for Myc, a master transcriptional regulator of cell growth and known NF-κB target gene, we revealed that 57% of cells had upregulated Myc levels at 24 h compared to 0 h, and 62% of large cells had elevated Myc levels (Fig4H). Upon NF-κB cRel deletion, only 35% of cells had elevated Myc levels (Fig4I). Similarly, BcL_XL_, a known NF-κB cRel target gene and anti-apoptotic regulator, was found to be elevated primarily in large cells (Fig4J). Whereas BcL_XL_ was upregulated in 85% of large cells, only 8% of all large cells upregulated BcL_XL_ in the absence of cRel (Fig4K). Quantitative RT–PCR confirmed that BcL_XL_ was upregulated at the mRNA level and that NF-κB cRel contributes about two-third of the BcL_XL_ expression at 20 h (Supplementary Fig S5B).

Repeating the immunofluorescence analysis in the presence of 1 ng/ml rapamycin, a known mTORc1 inhibitor (Supplementary Fig S5C), we found the same fraction of cells had upregulated cRel abundance after 24 h of stimulation, though the fraction of large cells was reduced, suggesting that cRel upregulation is independent of mTORc1. Conversely, we tested the role of NF-κB cRel in regulating mTORc1 and found that the abundance of p-S6, an indicator of mTORc1 activity, by immunoblot was reduced by approximately a factor of 2 in NF-κB cRel-deficient B cells (Fig4K), though cell growth itself was not substantially diminished presumably due to compensatory mechanisms including NF-κB family members RelA and RelB. Our analysis supports a model in which NF-κB is a regulator of both cell survival and cell growth.

### Constructing a multi-scale model to predict B-cell population dynamics

The described analyses of CFSE time courses, time-lapse microscopy, and molecular studies led us to test whether B-cell population dynamics may be accounted for with a mathematical model of intra-cellular molecular networks that exist in cell-specific steady states due to biochemical variability. We implemented established ordinary differential equation (ODE) kinetic models of the NF-κB signaling system (Alves *et al*, 2014), apoptotic control network (Loriaux *et al*, 2013), and the cell cycle (Conradie *et al*, 2010) (Supplementary Fig S6A). Introducing sources of extrinsic variability, we found that variability in protein levels alone was sufficient to produce cell-to-cell variability in nuclear NF-κB concentration, cell-cycle duration, and lifetime typically observed (Supplementary Fig S6B). Importantly, the cell-cycle model with added sources of extrinsic noise produced relatively short cell-cycle durations of ∽10–20 h, similar to generation 1+ cells, but did not readily account for the generation 0 delay (Fig2). Further, we found that introducing extrinsic protein variability resulted in substantial cell growth variability.

**Figure 5:**
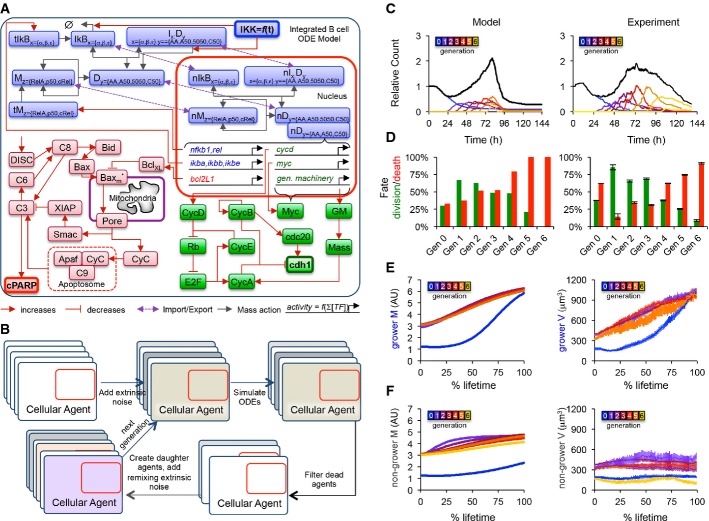
Multi-scale agent-based modeling of the B-cell response A Established ordinary differential equation models for NF-κB signaling (Alves *et al*, 2014), apoptosis (Loriaux & Hoffmann, 2012), and the cell cycle (Conradie *et al*, 2010) were implemented and combined into one integrated model. Blue, green, and red colors represent NF-κB, apoptosis, and cell-cycle modules, respectively, while bolded species represent active IKK (input), cleaved PARP (death readout), and cdh1 abundance (mitosis readout).

Based on our molecular analysis, we constructed an integrated ODE model (FigA and Supplementary Methods) with NF-κB-controlled synthesis of BcL_XL_, a key regulator in the apoptosis module, as well as NF-κB-controlled synthesis of CycD, a key regulator in the cell-cycle module. Furthermore, in the cell-cycle module, growth is controlled by general machinery (GM), which represents the ribosomes and other cellular components that promote the accumulation of cell mass. Mass in turn promotes the growth of general machinery, creating a positive feedback loop that results in exponential growth and cellular progression through the cell cycle. However, since we observed B cells to delay growth prior to the first division (Fig2), we needed to model the control of general machinery (GM) in more detail. Hence, we incorporated NF-κB-controlled synthesis of Myc, a transcription factor that promotes cell growth, which is typically low in quiescent cells but a known NF-κB target gene. To obtain population dynamics, the integrated ODE model was incorporated into cellular agents (Fig5B), which kept track of their generation, age, and independent set of starting synthesis/degradation or total protein concentrations, which were drawn from normal or log-normal distributions, respectively. The models were solved until the agent died [defined as (cPARP) > 25,000 molecules/cell] or completed mitosis [(cdh1) > 0.2], at which point it was removed or replaced by two new daughter agents, respectively. We subjected daughter agents to extrinsic re-mixing noise to account for loss of correlation with successive generations. When training the model on our results from the wild-type condition, we retained the value of all published NF-κB, cell-cycle, and apoptosis parameters, leaving a set of free parameters specifying Bcl_XL_, CycD, and Myc transcript synthesis and degradation, as well as parameters controlling the growth and survival of cells (Supplementary Methods and Supplementary Table S9). Remarkably, we were able to recapitulate the observed population dynamics (Fig5C), the fraction of cells dividing or dying in each generation (Fig5D), as well as the growth trajectories of growing and non-growing cells in each generation (Fig 5E and F) by fitting just these free parameters from within biologically plausible ranges to a set of features (Supplementary Tables S9 and S10, and Supplementary Methods).

B Instances of the integrated model were incorporated into cellular agents, extrinsic noise was introduced to mimic cell-to-cell variability, and the agent-based model was solved one generation at a time, with division resulting in the creation of two new agents, and death resulting in the removal of the agent from the population.

C–F A comparison of agent-based modeling solutions to the time-lapse microscopy dataset is shown. Cell counts normalized to start count (C), fraction of cells dividing or dying in each generation (D), average size of growers in each generation as a function of % lifetime (E), and average size of non-growers in each generation as a function of % lifetime (F) are compared. Error bars represent SEM or 1/*n*.

### Model-enabled perturbation studies: NF-κB cRel enforces the execution of the cell fate decision by biasing a fate race of growing cells against death

**Figure 6:**
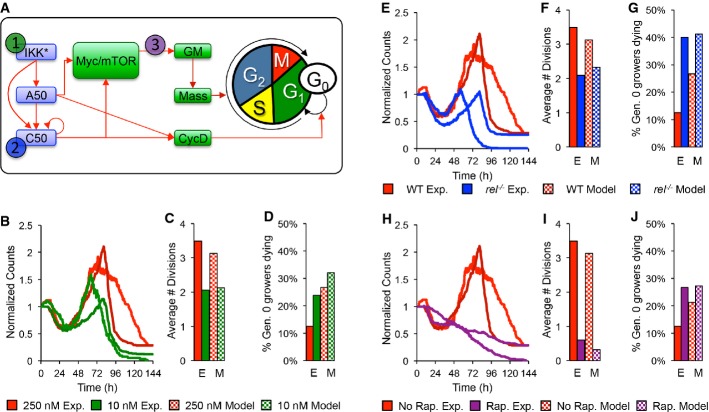
The multi-scale model predicts the effects of low stimulus, cRel knockout, and rapamycin treatment A After parameterizing the multi-scale model using results from wild-type B cells stimulated with 250 nM CpG (red), we predicted the effects of decreasing IKK duration (green), lack of NF-κB cRel (blue), and decreased protein synthesis (purple) *in silico* and compared the results to those from analogous time-lapse microscopy experiments where we stimulated with only 10 nM CpG, used cRel deficient cells, or pretreated with 1 ng/ml rapamycin.

We next asked whether the model could be used for studies of genetic or pharmacological perturbations. In particular, we examined the population behavior in B cells exposed to reduced stimulus concentrations in the absence of NF-κB cRel, or when treated with the cell growth inhibitor rapamycin (Fig6A). Model predictions were compared to time-lapse microscopy experiments in which the same conditions were applied. First, we simulated the low dose stimulation condition by allowing for a faster decay of the active IKK species (see Supplementary Table S9 and Supplementary Methods). The model predicted a dramatic decrease in the total B-cell population (FigB), resulting from a decrease in the fraction of cells that divide in generations 3+ (Fig6C); however, cell size trajectories (Supplementary Fig S7B and C) and fate timing (Supplementary Fig S7D–F) were unaffected. An equivalent analysis of subsequent time-lapse experiments confirmed these predictions (FigB–D, Supplementary Fig S7), although the model predicted a later peak in total cell counts (FigB, Supplementary Table S11). Next, we simulated cRel deficiency by setting the translation rate of the cRel protein to zero. The multi-scale model recapitulated a decreased population response (FigE) previously observed, but now showed that this is caused primarily by a reduction in the number of divisions (Fig6F). Model simulations suggest that cell growth is not cRel dependent since cRel-deficient cells had highly correlated growth trajectories (Supplementary Fig S7B and C). Furthermore, the model predicted that NF-κB cRel deficiency would not affect timing of the decision, division, or death processes (Supplementary Fig S7D–F), but that a higher percentage of growing cells would die (FigG). A side-by-side comparison with the results from experimental cell tracking of cRel-deficient cells confirmed these predictions (Supplementary Table S11). Finally, treatment with rapamycin, the inhibitor of mTOR, which results in defective cell growth and ribosome biosynthesis, as well as a decrease in cells that divide more than once (FigI), was recapitulated well by simply decreasing the global protein translation rate by 30% (Fig6A, H–J). Importantly, this also resulted in longer delays prior to division (*P *< 0.0014, Mann–Whitney *U*-test) and death (*P* < 0.0009, Mann–Whitney *U*-test) in time-lapse microscopy datasets (Supplementary Fig S7E and F purple lines); although the delay in division timing and in the start of growth (Tgro) was not as dramatic as predicted (Supplementary Fig S7D purple lines), it was still statistically significant (*P* < 1e-6, Mann–Whitney *U*-test). Interestingly, while the model accurately predicted that cell growth trajectories would not be affected, it overestimated the delay in cell-cycle duration and survival timing and incorrectly predicted a delayed time to grow (Supplementary Table S11).

B–J Side-by-side comparison of modeling and experimental results: total cell counts (B, E, H), average number of divisions (C, F, I), and fraction of growing progenitors that died (D, G, J).

**Figure 7:**
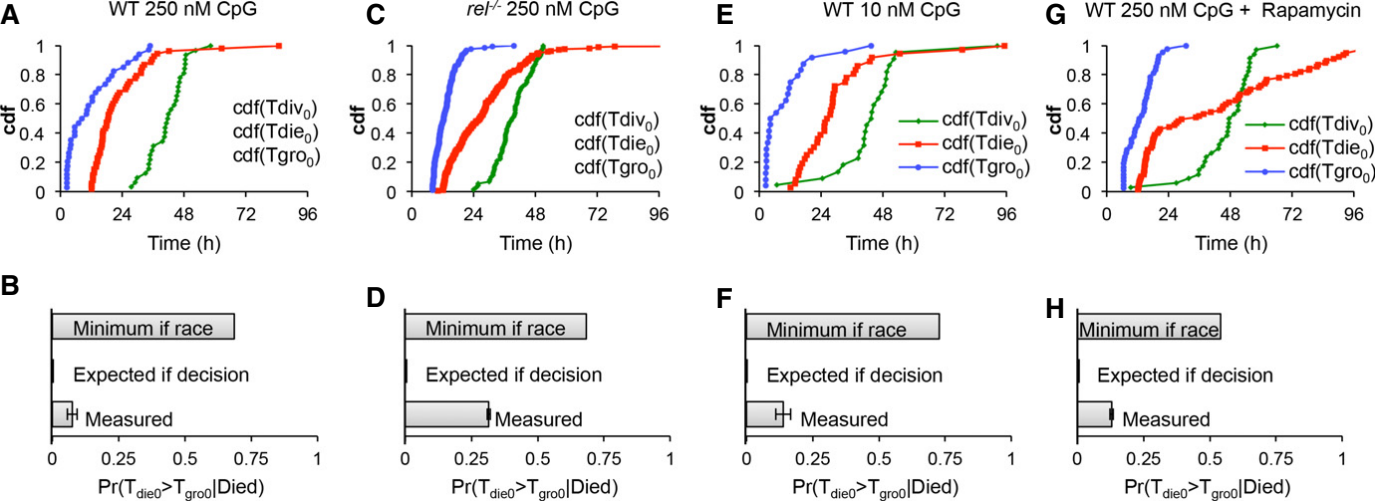
B-cell decision enforcement is NF-κB cRel dependent A–H Time-lapse microscopy images of wild-type B cells stimulated with 250 nM CpG (A, B), NF-κB cRel-deficient B cells stimulated with 250 nM CpG (C, D), wild-type cells stimulated with 10 nM CpG (E, F), and wild-type B cells stimulated with 250 nM CpG and pretreated with 1 ng/ml rapamycin for 1 h (G, H) were tracked. The observed cumulative distributions (A, C, E, G) for time to start growing (Tgro), time to divide (Tdiv), and time to die (Tdie) were used to estimate the minimum probability of observing grown cells that die in generation 0 assuming that division and death were occurring simultaneously (molecular race), and compared to the actual sampled probabilities for each condition (B, D, F, H).

The model predictions regarding cRel's role in protecting growing cells from apoptosis (Fig6G), prompted us to examine our experimental data further. We tabulated the observed probability that a dying cell had grown for the wild-type, cRel-deficient, low stimulus, and rapamycin-treated conditions (Fig7). The probability of observing dying ‘growers’ approximately tripled when cells lacked cRel (Fig7D compare to B), suggesting that growth and death were no longer mutually exclusive. The increased probability was still lower than the minimum probability expected for a complete loss of decision enforcement, calculated using observed distributions for the time to start growing, divide, and die (Fig7C). A lack of decision enforcement was not seen when a lower dose of the stimulus (Fig7E and F) or rapamycin drug treatment (Fig7G and H) was used, confirming NF-κB cRel's specific role. These studies suggest that the phenomenological cell fate decision is mediated at the molecular level by cRel, which biases a cell fate race in growing cells against cell death, rendering them pre-determined for division.

### Extrinsic molecular network noise determines the magnitude of the population response

**Figure 8:**
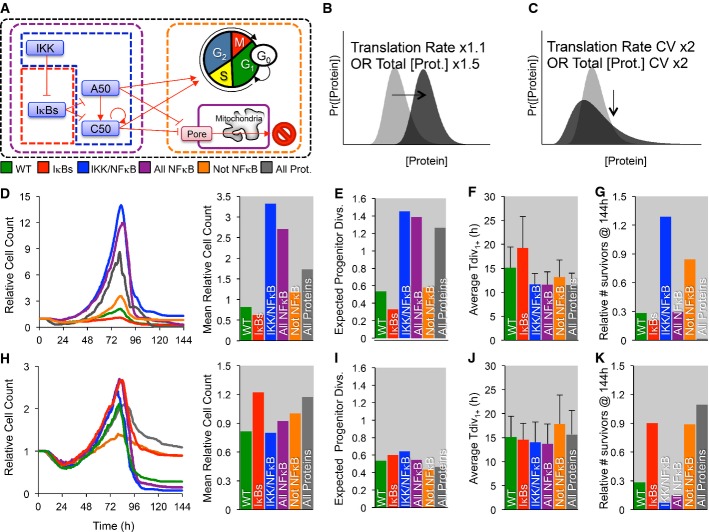
*In silico* increases in the coefficient of variability or average protein abundances differentially affect population dynamics A–K The cell-to-cell distribution of total IKK (green), NF-κB cRel/NF-κB p50 (blue), both NF-κB C,50 and total IKK (purple), non-NF-κB proteins (orange), or all proteins (gray) was varied (A). Specifically, the protein production (B, D–G) was increased (Total IKK mean ×1.5, cRel/p50 induction ×1.5, or protein production ×1.5), or the coefficient of variation (C, H–K) was doubled, and the population dynamics and maximum relative cell count (D, H), mean number of times a progenitor is expected to divide given the observed fraction of dividers in each generation (E, I), average generation 1,2,… division times (F, J), and the number of *in silico* cell surviving at the end of the simulation (G, K) were compared. Error bars = SD. No error bars are shown for D, E, G, H, I, and K as they represent one global feature for each simulation.

Utilizing the multi-scale model, we explored how the average and the variability of protein abundances within the molecular network may affect the population response. In this analysis, we distinguished between negative regulators of NF-κB signaling (the IκBs), the positive regulators (IKK and the NF-κB monomers RelA, p50, and cRel), or both, as well as apoptosis and cell-cycle regulators, or all proteins (Fig8A). Increased average abundance (Fig8B) was achieved by increasing the translation rate or the total protein abundance (if constant) by 10 or 50%, respectively, while increased protein variability (Fig8C) was achieved by doubling the coefficient of variation (CV) of the translation rate or total protein abundance (if constant). As expected, moderately increasing the average protein abundance resulted in dramatic changes to the population dynamics (Fig8D), as long as the positive regulators were among those affected (blue, purple, and gray conditions). Our analysis indicates that this is primarily caused by an increase in the number of division rounds that progenitors underwent (Fig8E), as well as due to typically shorter interdivision times (Fig8F). Meanwhile, increasing the expression of negative regulators of NF-κB (IκBs) decreased the population response (Fig8D), decreased propensity to divide (Fig8E), and resulted in typically longer cell-cycle duration (Fig8F). Furthermore, increasing the positive regulators alone and to a lesser extent the cell-cycle/apoptosis proteins resulted in an accumulation of non-dividing and surviving cells (Fig8G; blue, orange), while increasing negative regulators (IκBs) tended to decrease survival (Fig8G; red versus green, purple versus blue, gray versus orange).

However, when manipulating the variability of expression only, we found that increased variability in negative regulators of NF-κB and non-NF-κB proteins resulted in increased cell counts over time, due to accumulation of non-dividing surviving cells (Fig8K; red, orange, gray). Increasing the CV of both the positive and negative regulators resulted in modest increases in the number of times a progenitor divided (Fig8I); however, doubling the CV of negative regulators also resulted in increased survival (Fig8K). Increased variability for apoptosis and cell-cycle proteins also resulted in higher survival (Fig8K; orange, gray); however, on average cells experienced fewer division rounds (Fig8I), resulting in broad population dynamics, indicating that cell-cycle regulation is sensitive to relatively large increases in protein variability (Fig8H). Thus, the multi-scale model enabled us to test the role that extrinsic variability plays in a module-specific manner, revealing that extrinsic noise in the expression of negative regulators of NF-κB can lead to hyper-proliferative phenotypes due in part to long-term cell survival, while positive regulators of NF-κB determine the number of divisions.

## Discussion

The complexity and inherent heterogeneity of the B-cell population response poses serious challenges to predicting modes of disease action and the potential efficacy of drugs. In this study, a combination of single-cell molecular assays, single-cell time-lapse microscopy, and population flow cytometry allowed us to construct a multi-scale model, in which the intra-cellular network of NF-κB signaling, cell-cycle, and apoptosis control accounts for the cell population dynamics in response to mitogen, which provides a framework for genetic and pharmacological perturbation studies that begin to link molecular scale perturbations to organ-level phenotypes and function.

### Agent-based multi-scale modeling of the B-cell immune response

Agent-based models (ABMs) explicitly describe autonomous entities within a system and provide a natural computational framework for modeling immune processes (recently reviewed in An *et al*, 2009; Narang *et al*, 2012). As such, ABMs have been successfully utilized to provide insights into the dynamics of the NF-κB signaling system (Pogson *et al*, 2006), wound healing (Walker *et al*, 2004), the multi-scale effects of acute inflammation (An, 2008), the implications of transgenerational epigenetic inheritance (Jiao *et al*, 2012), and the evolution of aging (Shokhirev & Johnson, 2014).

In the absence of an established framework for multi-scale B-cell modeling, we took a parsimonious approach toward model construction. Since the number of parameters typically scales nonlinearly with the size of the model, our strategy was to use previously established models and manually parameterize the connections between them based on experimental studies following genetic or pharmacological perturbations. While numerous regulators of B-cell signaling and proliferation have been identified, we focused here (for the purposes of this first version of a B-cell ABM) on the stimulus-responsive NF-κB signaling system as a key determinant of B-cell population dynamics (Pohl *et al*, 2002). Several important regulators are known NF-κB target genes (Duyao *et al*, 1990; Wang *et al*, 1996; Chen *et al*, 1999; Guttridge *et al*, 1999; Huang *et al*, 2001); however, how they function together to produce the observed population dynamics remained poorly understood. We took both an unbiased approach by sequencing the transcriptomes of small cells and growing cells, and a targeted approach via single-cell measurements of key proteins by immunofluorescence. While there was significant cell-to-cell transcriptome variability (Supplementary Table S2), there was a clear NF-κB signaling signature in large but not small cells (Fig4), indicating that the fate variability may originate upstream of the NF-κB system. Indeed, NF-κB cRel and RelA are upregulated after 24 h of stimulation in large but not small cells (Fig4F and Supplementary Fig S5A), causing the upregulation of the growth regulator Myc (Fig4H and I) and the anti-apoptotic regulator Bcl_XL_ (Fig4J and K) and the activity of the metabolic regulator mTORc1 (Fig4L). These studies quantified the connectivity of NF-κB with downstream effector functions, enabling us to parameterize the relative contributions of NF-κB cRel, RelA, and non-NF-κB transcriptional regulators toward the activation of these downstream effectors. This placed NF-κB in a position of biasing the fate of growing cells toward division over death.

In addition, we were also able to confirm that fate decisions and division and death times are correlated between sibling cells (Supplementary Fig S4), which is consistent with apparent cell-to-cell variability being due to differences in protein turnover processes (Gaudet *et al*, 2012; Flusberg *et al*, 2013) resulting in distributions of single-cell proteomes within a population. Such variability constitutes noise that is extrinsic to the molecular processes explicitly represented in the model, thus justifying an ordinary differential equation formulation with distributed initial states to model a population of B cells, akin to previous studies (Spencer & Sorger, 2011; Loriaux & Hoffmann, 2012).

The resulting simulations recapitulated major features of the cellular and population responses with an accuracy that was surprising given that the three models were connected with a minimum number of reactions (Supplementary Methods,Supplementary Table S9). In particular, the maximum relative cell count (FigC), the characteristic total population expansion and contraction curve (Fig5C), the number of divisions observed (Fig5C and D), the fraction of cells responding in each generation (Fig5D), and the average growth trajectories of growing (Fig5E) and non-growing (Fig5F) cells were captured by the model, among others (see Supplementary Tables S9 and S10). The model was then used to predict population dynamics following a number genetic and pharmacological perturbations that yielded a first set of biological insights.

### NF-κB cRel enforces a cell fate decision by protecting growing cells against death

Previous studies have described the B-cell response as a molecular race between division and death processes (Hawkins *et al*, 2007b; Duffy *et al*, 2012), while others have argued for an early decision process (Hyrien *et al*, 2010; Shokhirev & Hoffmann, 2013; Chakravorty *et al*, 2014). A decision model is consistent with the observation that a subset of generation 0 cells prepare for several rounds of rapid divisions by simultaneously deactivating quiescence (Yusuf & Fruman, 2003; Hawkins *et al*, 2009) and activating growth pathways such as Myc and mTOR (Grumont *et al*, 2002; Wang *et al*, 2011). In contrast, cell death is a default pathway as unstimulated B cells will undergo apoptosis *in vitro* (Supplementary Fig S8), though cell lifetime may be extended by expressing anti-apoptotic regulators as a consequence of signaling (recently reviewed in Renault & Chipuk, 2013).

To probe whether the division or death fate was a result of a fate race or a decision, we tracked B cells in long time course microscopy studies to characterize several key properties of the response. There is a pronounced but variable delay in growth initiation prior to the first division, while generation 1+ cells start growing immediately (FigD). Tracking cell size trajectories and their eventual fate allowed us to show that B cells that had entered the growth phase were protected from death (Fig3). Further, a mathematical model which assumed a race between division and death (Hawkins *et al*, 2007b), applied to flow cytometry data, could not account for the death time distribution observed in microscopy experiments (Supplementary Fig S3), even when early death (within the first 12 h), potentially caused by mechanical manipulation of cells, is filtered out (Supplementary Fig S1).

Using the multi-scale model, we explored NF-κB's role in determining B-cell population dynamics. As expected, *in silico* knockout of NF-κB cRel substantially reduced the population response (FigE), allowing for fewer divisions (Fig6F). This was due to a greater fraction of growing cells dying (Fig6G), but fate timing and growth trajectories were predicted to and remained largely unchanged (Supplementary Fig S7). Importantly, time-lapse microscopy experiments confirmed these model predictions (FigE–G, Supplementary Fig S7). Further, model simulations predicted and experimental studies confirmed that in the absence of cRel, cells that have entered the growth phase may not be committed to divide, but instead are prone to death (Fig7D). Thus, cRel's function may be described as enforcing a decision to divide, with the population response of cRel-deficient cells resembling that of a molecular race more closely than that of wild-type cells. Indeed, our work with cRel-deficient models and cells suggests that the fate decision at the cell biological scale may be described as a fate race that is highly biased against death by NF-κB cRel. Other NF-κB members such as RelA and RelB may contribute as well, and their combined function is likely critical for promoting entry into the growth phase also.

### The population response is sensitive to extrinsic noise in the signaling module

The present model version could be used to explore how molecular-level perturbations affect cell population dynamics (Fig8). It may not be surprising that increasing the abundance of negative regulators of NF-κB diminished the population response; however, the sensitivity to small increases in the positive regulators was striking (Fig8D), affirming the strategy for searching for cancer-causing mutations that alter NF-κB control (Staudt, 2010). We also found an increased population response (Fig8H) due to enhanced survival (Fig8K) if instead the variability but not the average of protein abundances was increased in the model. Our data suggest that deregulation (i.e., increased variability) of negative regulators was particularly important (Fig8H–K). Increased cell-to-cell variability of cell-cycle and apoptosis proteins resulted in accumulation of long-lived cells, decreasing the fraction of dividing cells in each generation (Fig8I) and increasing the length of the cell cycle (Fig8J). Our analysis points to the importance of quantitative data at the single-cell level (e.g., the distributions of protein abundances, even when average measurements remain unaltered) in the diagnosis and prognosis of disease using single-cell technologies (Chattopadhyay *et al*, 2014; Macaulay & Voet, 2014).

In sum, the multi-scale model we present here is a first attempt at connecting molecular networks to B-cell population dynamics and demonstrates that much of the population behavior, including the observed biasing of cell fate, emerges when NF-κB is allowed to affect mammalian models for cell cycling and apoptosis. This model enables *in silico* molecular perturbation studies, allows the testing of many molecular factors and mechanisms simultaneously, and can serve as a framework for refinement within the iterative Systems Biology approach.

## Materials and Methods

### B-cell purification and incubation

Primary splenocytes were isolated from 6- to 8-week-old mice, and naïve B cells purified using magnetic bead separation (Miltenyi Biotec) and stimulated with 250 nM, or 10 nM CpG ODN 1668 (Invivogen). mTORc1 inhibition was achieved by 1 h pretreatment of 1 ng/ml rapamycin (Sigma) prior to addition of stimulus. B cells were grown in fresh media with 1% penicillin streptomycin solution (Mediatech Inc.), 5 mM L-glutamine (Mediatech Inc.), 25 mM HEPES buffer (Mediatech Inc.), 10% FCS, and 55 µM 2-ME (Fisher Scientific) at a concentration of 5 × 10^4^ cells/ml in 48 well plates, or 1,536 flat-bottom tissue-culture plates at 37°C for a period of 1–6 days.

### Time-lapse microscopy

Purified naïve B cells were grown in 1,536 flat-bottom tissue-culture-treated microwells (Greiner Bio-One). Images were acquired on an Axio Observer Z1 inverted microscope (Carl Zeiss Microscopy GmbH, Germany) with a 10×, 0.3 NA air immersion objective to a Coolsnap HQ2 CCDcamera (Photometrics, Canada) using ZEN imaging software (Carl Zeiss Microscopy GmbH). Environmental conditions were maintained at 37°C, 10% CO_2_ with a heated enclosure, and CO_2_ controller (Pecon, Germany). Phase-contrast images were taken every minute for 6 days.

### Cell tracking

A semi-automated computational approach was used to track B cells in phase-contrast images. First, image intensities were normalized to maximize contrast. Next, edges were identified using a Sobel transformation and global thresholding. Cells were identified using a customized Hough transformation assuming cells were approximately circular. Next, approximate linear paths were manually drawn for each cell until the cell was observed to divide, die, or leave the field of view (also manually annotated to ensure accuracy). Cells entering the field of view after 24 h of stimulation (i.e., potentially after the first division) and debris were tracked but removed from the subsequent analysis. After all paths were drawn, all cell boundaries were optimized simultaneously from frame to frame. During automatic optimization, cells were modeled as deformable two-dimensional polygons with forces acting upon each vertex that ensured the polygons did not grow/shrink too quickly, did not overlap other polygons, were attracted to edges in the image, and were attracted to their respective manually curated path. The relative magnitudes of the forces were manually calibrated to ensure appropriate behavior. Cell size trajectories were fitted using a piecewise function consisting of a linear no-growth period, followed by exponential growth: 
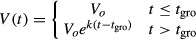


The quality of individual cell tracks were assessed by calculating RMSD from *V*(*t*), and the *t*_gro_ value was assumed to be the fitted inflection point in this function (i.e., when cells were predicted to start exponential growth). Growing cells were defined as having an average ending volume at least 350 μm^3^ above the average starting volume, or if the final volume was at least 800 μm^3^. Cells that grew but then decreased in size or that did not meet any of these conditions were labeled as non-growing. The Java platform-independent executable for tracking cells is included as Supplementary File S1. Tracked videos of WT 250 nM CpG, WT 10 nM CpG, NF-κB cRel deficient 250 nM CpG, and WT 250 nM CpG + Rapamycin treatment B cells are provided as Supplementary Files S2, S3, S4, and S5, respectively.

### Calculating the expected probability that a dying cell would have started growing

For a detailed methodology and notes, please see Supplementary Methods. In short, if division and death are parallel biological processes running within a cell, it is possible to calculate the lower bound on the expected fraction of progenitor cells that would start growing and then die if the time to die is typically earlier than the time to division. We predict this lower bound from the observed distributions for the time to decide to grow (Tgro_0_), time to die (Tdie_0_), time to divide (Tdiv_0_), and the observed fraction of dividing cells in generation 0, and then compare the predicted lower bound to the actual observed fraction of growing cells that die.

### Single-cell RNAseq

Stimulated wild-type B cells were collected at 24 h post-stimulation and concentrated to 5 × 10^5^ cells per ml. Cells were loaded onto a 10–17 µM primed C_1_ single-cell auto prep array IFC (Fluidigm), and phase-contrast images were taken of all viable cells as determined by the Live/Dead® Viability/Cytotoxicity Kit (Invitrogen). ERCC RNA spikein controls (Life Technologies) were added to the lysis mix at a 1:200 dilution. Tube controls (bulk cell positive control and no cell negative control) were also prepared according to the Fluidigm protocol. Lysis, reverse transcription, and PCR were performed using the SMARTer Ultra Low RNA Kit (CloneTech) and Advantage® 2 PCR Kit (CloneTech) on individual cells using the C_1_ Single-Cell Auto Prep System (Fluidigm). Cell size was manually determined from images using ImageJ software. Sample libraries for the five smallest and five largest cells along with the controls were prepared using the Nextera XT DNA Sample Preparation (Illumina), and library quality was assayed using the Quant-iT PicoGreen dye (Life Technologies) quantification on a Qubit® 2.0 Fluorometer (Life Technologies) and by gel electrophoresis. Libraries were sequenced by the UCLA Broad Stem Cell Research Center High Throughput Sequencing Core on Illumina HiSeq 2000 sequencers according to manufacturer recommendations. Reads were aligned to the ENSEMBL NCBI m37 genome (Church *et al*, 2009) using rna-STAR (Dobin *et al*, 2013). To compute spike-in concentrations for normalization purposes, the 23 most abundant RNA spike-in concentrations (at least one read in all samples) were compared to the expected concentrations in log-log space, and the y-intercept in log-space was used to compute normalized spike-in concentrations for each sample, [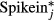]. The normalized expression of gene *i*, in sample *j*, was then computed as: 
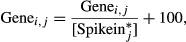


A constant count of 100 was added because spike-ins with counts < 100 were variable across samples. To assess the overall quality of each cell, we correlated their genome wide transcript rpkm values to the average across all cells as well as to the positive tube control. We found that one large cell had significantly lower correlation, so we omitted it from further analysis. To determine whether a particular gene was upregulated in big cells, we computed an expression score: 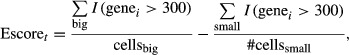


where *I*(gene_*i*_ > 300) is 1 if gene i has above 300 read count in a particular sample. A gene with an expression score ≥ 0.5 was considered upregulated in big cells, while a gene with an expression score ≤ −0.5 was considered downregulated in big cells. These sets of upregulated and downregulated genes were analyzed for pathway enrichment and transcription factor motif enrichment using WebGestalt (Wang *et al*, 2013). Significant transcription factors were further filtered to remove non-NF-κB downstream targets as defined in Supplementary Table S1. The single-cell RNA seq feature counts and analysis are included as a Microsoft Excel file (Supplementary File S6).

### Immunofluorescence

1 × 10^6^ cells were collected after 24 h incubation at 37°C and 5% CO_2_, washed with cold 1 × PBS, resuspended in Annexin binding buffer containing 10 µl Annexin-V conjugated to AlexaFluor350 (Life Technologies), stained for 1 h at 25°C, fixed with 4% para-formaldehyde (Electron Microscopy Sciences) for 15 min, washed with 1 × PBS, and incubated in cold blocking buffer (1 × PBS, 5% normal goat serum, 0.4% Triton™X-100, 0.02% SDS), washed with IF buffer, counted, and then incubated at 4°C overnight in IF buffer containing 1:100 primary antibody such that cell densities and antibody concentrations were normalized across all conditions. After incubation with primary antibody, cells were washed 3 × 5 min with IF buffer and incubated in IF buffer containing 1:1,000 secondary antibody for 1 h, washed with IF buffer (3 × 5 min), plated into μ-slide 8-well plates (ibidi), and visualized using on an Axio Observer Z1 inverted microscope (Carl Zeiss Microscopy GmbH) with a 20×, 0.3 NA air immersion objective, acquired to a Coolsnap HQ2 CCD camera (Photometrics) using ZEN imaging software (Carl Zeiss Microscopy GmbH). Cells in images were manually identified in phase contrast (circular shape), and average fluorescence values were recorded after local background subtraction. Fluorescence from debris was manually excluded from calculations. Cell viability prior to fixation was confirmed with Annexin-V staining in the blue channel. Antibodies used for NF-κB RelA (Rabbit anti-p65, sc-372), cRel (anti-mouse cRel conjugated to PE, #12-6111-80), Myc (Rabbit anti-cMyc, ab32072), and Bcl_XL_ (Rabbit anti-Bcl_XL_, ab2568) were obtained from SantaCruz Biotechnology, eBioScience, AbCam, and AbCam, respectively. Goat anti-rabbit-conjugated secondary antibodies were obtained from Life Technologies (A-11001). To quantify changes in expression, we found significance thresholds for cell size and average cell fluorescence for the indicated protein after 24 h as compared to the 0 h control. Percentages (FigH–K, and Supplementary Fig S5A and C) show the fraction of cells from the 24 h time point occupying each quadrant. Significant cell size was defined to be > 100 pixels manually, while significant abundance was defined as a value that is greater than or equal to the 95th percentile abundance from the 0 h datasets. All immunofluorescence images and the custom Java software used to analyze the images are provided as a zipped file (Supplementary File S7).

### CFSE flow cytometry and FlowMax analysis

Cells were removed from media, stained with 10 ng/ml propidium iodide, and measured using an Accuri C6 Flow Cytometer (Accuri Inc.) over a 6-day time course. CFSE histograms were constructed after software compensation for fluorescence spillover and manual gating on viable (PI-negative) B cells using FlowMax software. All measurements were performed in duplicate (B cells from the same spleen were cultured in separate wells, two wells per time point to ensure that each time course represented a single population of cells subject to only experimental variability). The FlowMax computational tool (Shokhirev & Hoffmann, 2013) was used to construct 1D log-transformed CFSE histograms of viable cells. After specifying the fluorescence of the undivided peak manually for each time point, maximum-likelihood fcyton model parameter ranges were determined by filtering, and clustering 1,000 best-fit solutions and their corresponding sensitivity ranges. The top solution cluster was plotted by randomly sampling parameters from within the maximum-likelihood parameter ranges. To account for potential censorship of the fraction of dividing cells or division and death time distributions when both division and death processes were active simultaneously (i.e., cyton model), Monte Carlo sampling of cell populations was used to approximate population model parameters directly.

### Western blot analysis

Whole-cell lysates were prepared using RIPA buffer lysis of B cells. The resulting lysates were resolved on a 10% SDS–PAGE and proteins detected using the Bio-Rad ChemiDoc XRS System and SuperSignal west femto substrate (Thermo Scientific). Antibodies used to identify the protein of interest are as follows: S6 Ribosomal Protein (Cell signaling #2217) and α-tubulin (Santa Cruz sc-5286). Quantification was performed using ImageJ software using the 0 h protein levels for normalization.

### RT–PCR

RNA extraction was performed using RNAeasy Mini Kit (Qiagen). cDNA synthesis of purified RNA was done with iScript cDNA Synthesis kit (Bio-Rad). Quantitative RT–PCR was performed with SYBR Green PCR Master Mix reagent (Stratagene) and Eppendorf Mastercycler realplex system using the Δ(Δ*C*_t_) method with β-actin as normalization control.

### Multi-scale agent-based modeling

Ordinary differential equation models of the cell-cycle (Conradie *et al*, 2010), apoptosis (Loriaux & Hoffmann, 2012), and NF-κB signaling (Alves *et al*, 2014) were implemented in Matlab (Mathworks), using the ode15s solver for stiff problems. Please refer to the Supplementary Methods for the list of model reactants, reactions, constants, and free parameters, as well as the fitting methodology and parameter sensitivities. The modules were connected by imposing cooperative Hill activation of the CyclinD, Myc, and Bcl_XL_ promoters. For simplicity, we also assumed that the growth of general cellular machinery, the model species representing catabolism and protein synthesis in the cell, was dependent on the current mass, and the concentration of Myc. The integrated model consisting of the three modules constituted one cellular agent and was solved independently in a generation-by-generation fashion until the simulation time limit was reached, the cell divided ([cdh20] > 0.2), or the cell died ([cPARP] ≥ 25,000 molecules/cell). Upon division, two new copies of the model were generated with half the mass and general machinery (we assumed that the concentration of all other species was unchanged). We assumed a normally distributed variability in the partitioning of the mass (the mass and general machinery were multiplied by a constant *r*_*a*_ or *r*_*b*_ such that *r*_*a*_ ∽ *N*(1.0, CV_partition_), and *r*_*b*_ = 1 – *r*_*a*_, where CV_partition_ is a the coefficient of variability of daughters measured from wild-type microscopy experiments. In addition, to mimic protein concentration remixing which leads to the loss of correlation with subsequent divisions, we generated independent log-normally distributed (if not modeling synthesis and degradation) or normally distributed protein synthesis and degradation reaction rates as well as log-normally distributed total IKK concentrations and set the daughter values to the average of the newly generated value and the value inherited from the mother. This ensured that the daughter cells had correlated protein dynamics, but that the correlation decreased with each generation (Hawkins *et al*, 2009). At the time of division, species in the nucleus (i.e., NF-κBs/IκBs) were redistributed evenly among the nucleus and cytoplasm to mimic nuclear envelope breakdown and reformation. Models that ended in death were removed from the pool of running models. Multi-scale models, which consisted of many such cellular agents, were initialized at generation 0 to contain *n* = 250 independent integrated models. To model cell-to-cell protein abundance variability, we initialized each model with initial protein concentrations sampled from lognormal distributions if the total protein concentration was fixed, or with normally distributed protein synthesis and degradation rates if the protein had explicit synthesis and degradation reactions defined. In addition, the total amount of IKK, the upstream signal responsible for NF-κB activation, was also assumed to be log-normally distributed. Finally, the initial mass and general machinery of cells was normally distributed as determined by microscopy. After an initial equilibration phase (at least 24 h) with only basal IKK signaling to NF-κB and no death signaling, a quiescent steady state was achieved as defined by lack of cell-cycle progression and model species stabilization. After equilibration, all models were solved independently for each generation until the simulation time was elapsed (144 h). The full set of model constants, reactions, fluxes, species, parameterizations, parameter sensitivity, fitting procedure, and model construction methods are described in the supplementary tables, and the model construction details and fitting routines are described in Supplementary Methods. The full model code is provided as a collection of zipped MATLAB files (Supplementary File S8).

### Predicting the role of extrinsic abundance noise for specific sets of proteins

We used the multi-scale model to predict the effect of increasing the variability or mean protein levels in specific modules. To do this, we grouped proteins in the model into functionally distinct sets: the negative regulators of activation (IκBs), the positive regulators of activation (IKK and the NF-κB monomers), and all other proteins (cell-cycle and apoptosis). Then, we tested the effect of increasing the average abundance of the proteins in these functional sets by increasing the translation rate of the proteins (IκBs, NF-κBs, cell-cycle/apoptosis proteins) by 10%, or increasing the total concentration by 50% (for proteins which are assumed to be constant in the model such as IKK and some cell-cycle proteins). We also tested the effect of increasing the extrinsic noise of these protein sets by increasing the CV associated with the cell-to-cell protein variability in the synthesis/degradation rates (assumed to be drawn from a normal distribution with mean centered on the initial parameter value) or the total protein abundance (assumed to be log-normally distributed with mean equal to the initial starting concentration). We then quantified the mean relative population count throughout the simulation, the expected number of divisions that progenitors underwent, a summary statistic for the fractions of cells dividing in each generation (fs), the average interdivision time for generation 1+ cells (a measure of cell-cycle speed), and the relative number of cells remaining at the end of the simulation (accumulation of surviving non-dividing cells).

### Data availability

Supplementary files, parameter tables, movies of tracked cells, code, raw images from time-lapse microscopy, and CFSE flow cytometry datasets (FCS3.0 files) are available for download at http://www.signalingsystems.ucla.edu/max/. Model parameters are also available as Supplementary Dataset S1. Single-cell RNA sequencing datasets are available from GEO: GSE64156 (http://www.ncbi.nlm.nih.gov/geo/query/acc.cgi?acc=GSE64156).

## Supporting Information

Supplementary Legends

Review Process File
