# Supplementary figures and images for: A multi-scale approach reveals that NF-κB cRel enforces a B-cell decision to divide (part 1 of 3)
Source: Mol Syst Biol. 2015 Feb 13;11(2):783. doi: 10.15252/msb.20145554 (PMC4358656; doi:10.15252/msb.20145554)

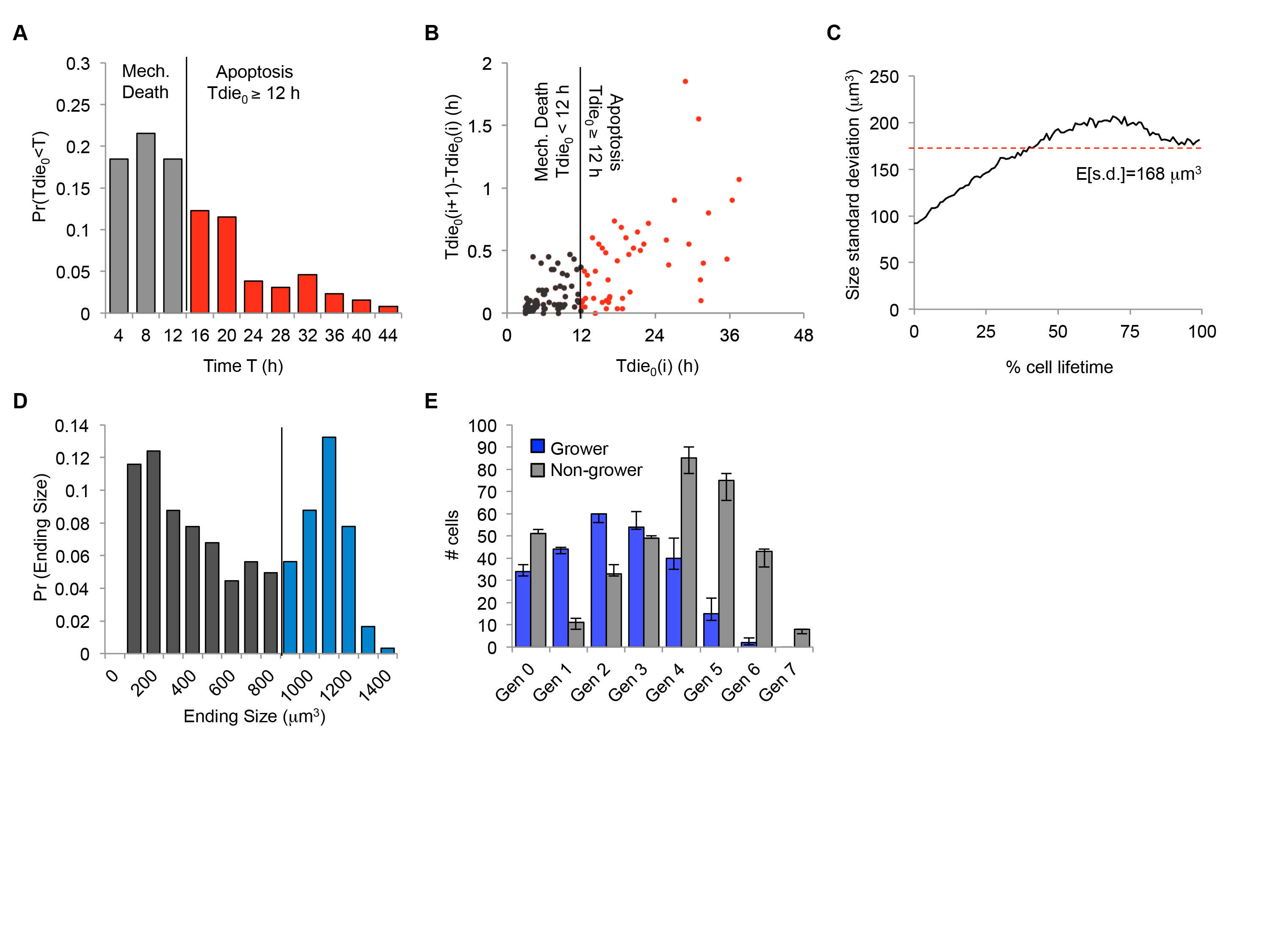

Supplement: Supplementary file 1 [file msb0011-0783-sd1.png]

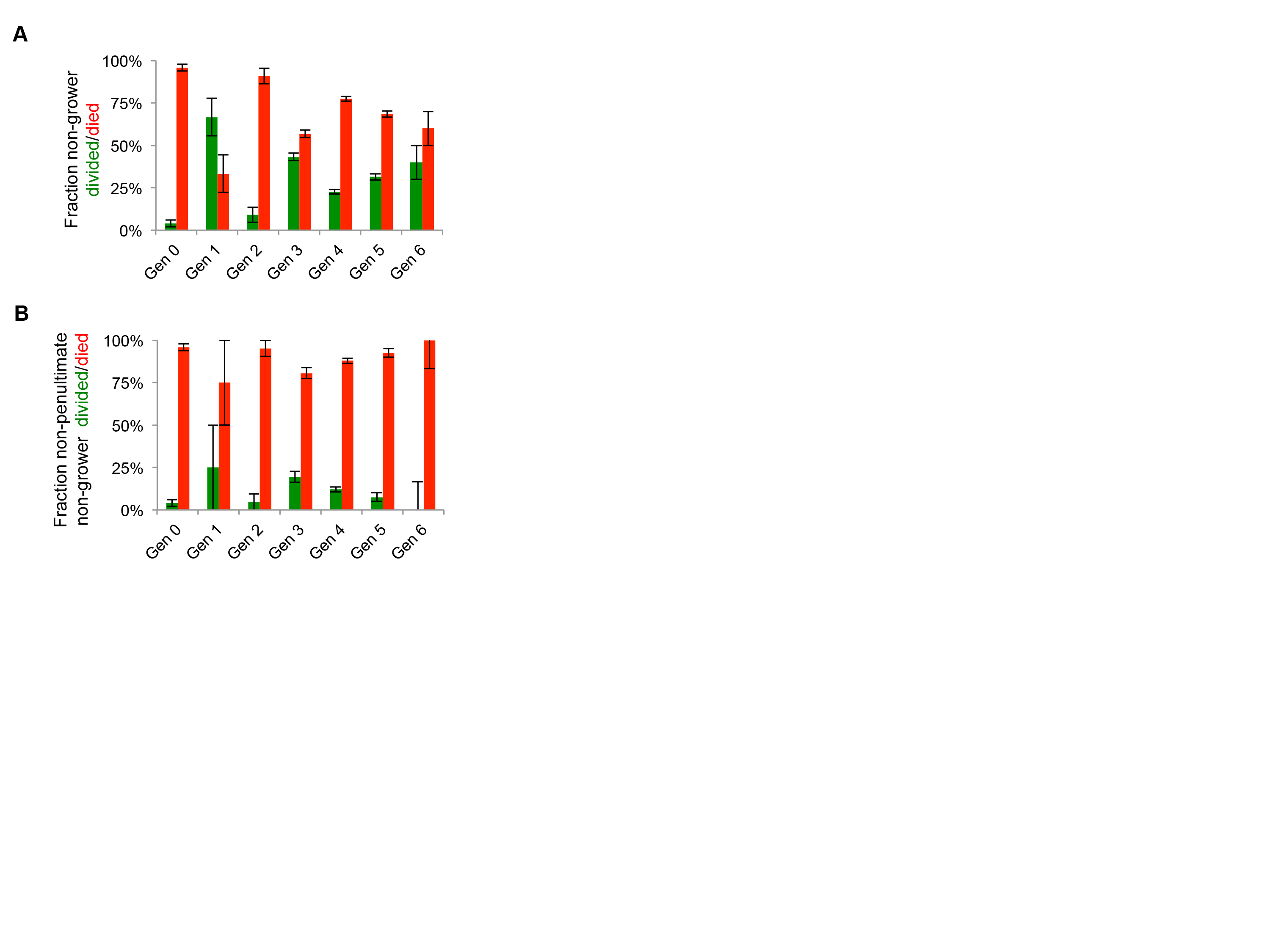

Supplement: Supplementary file 2 [file msb0011-0783-sd2.png]

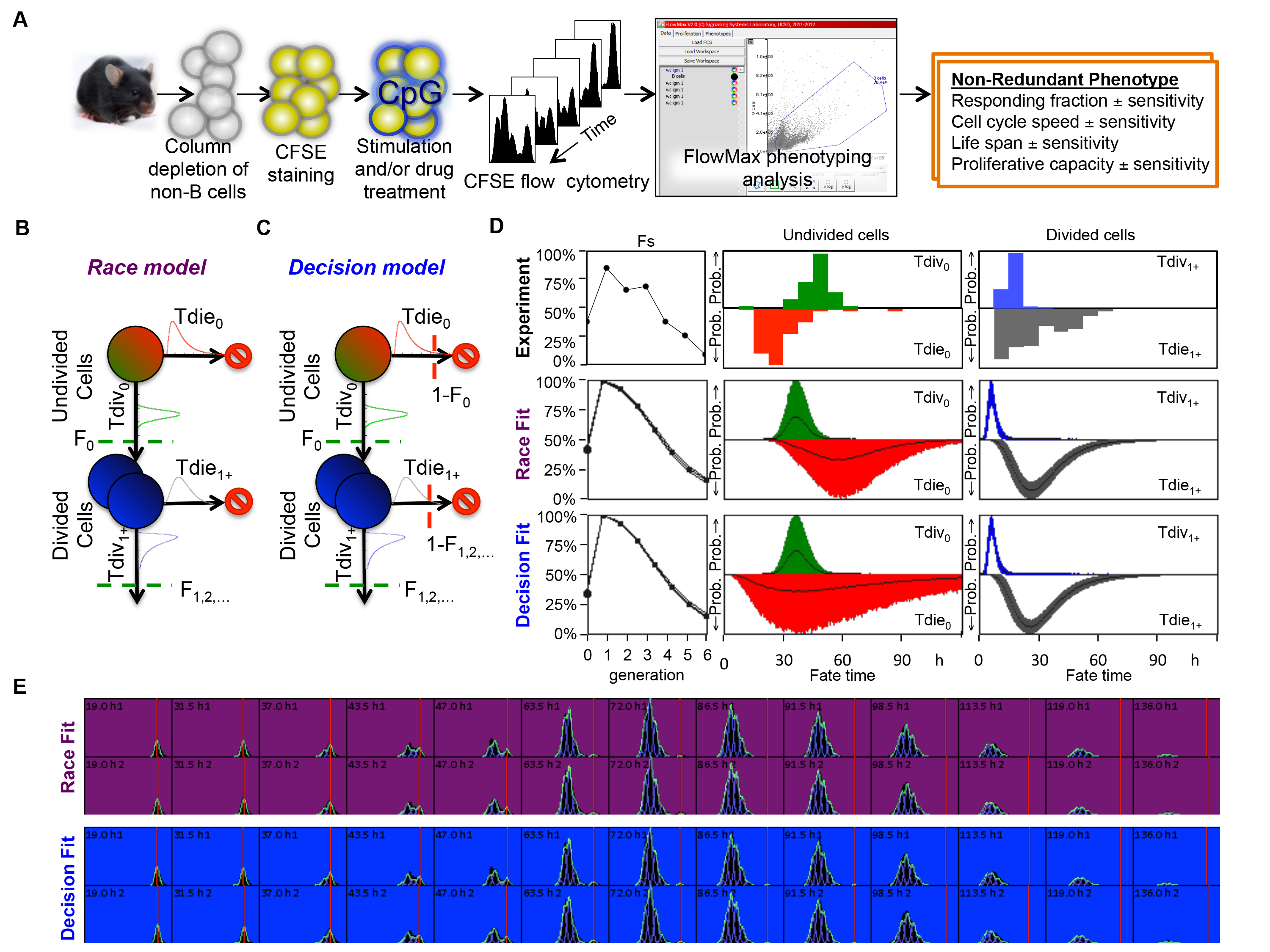

Supplement: Supplementary file 3 [file msb0011-0783-sd3.png]

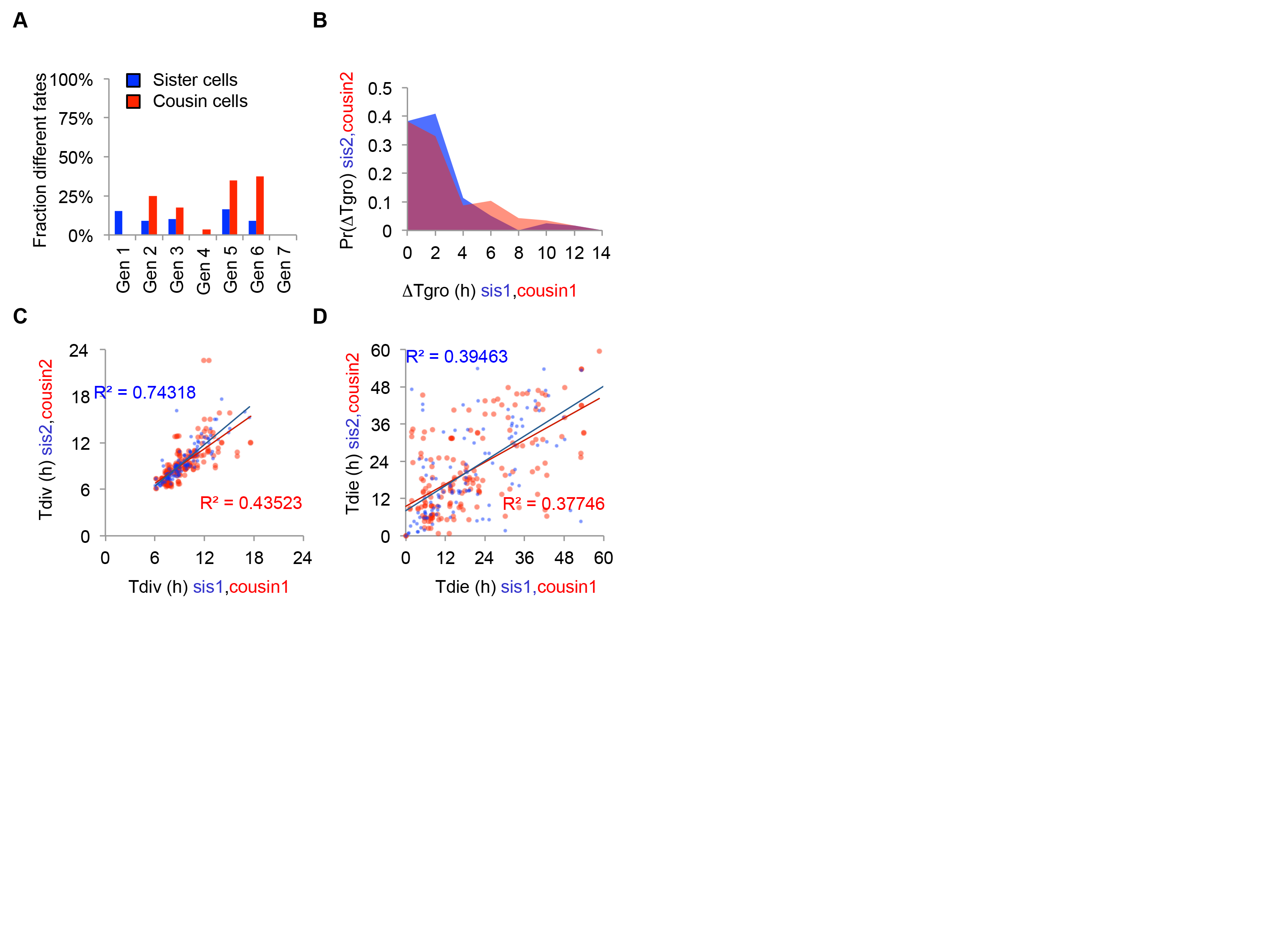

Supplement: Supplementary file 4 [file msb0011-0783-sd4.png]

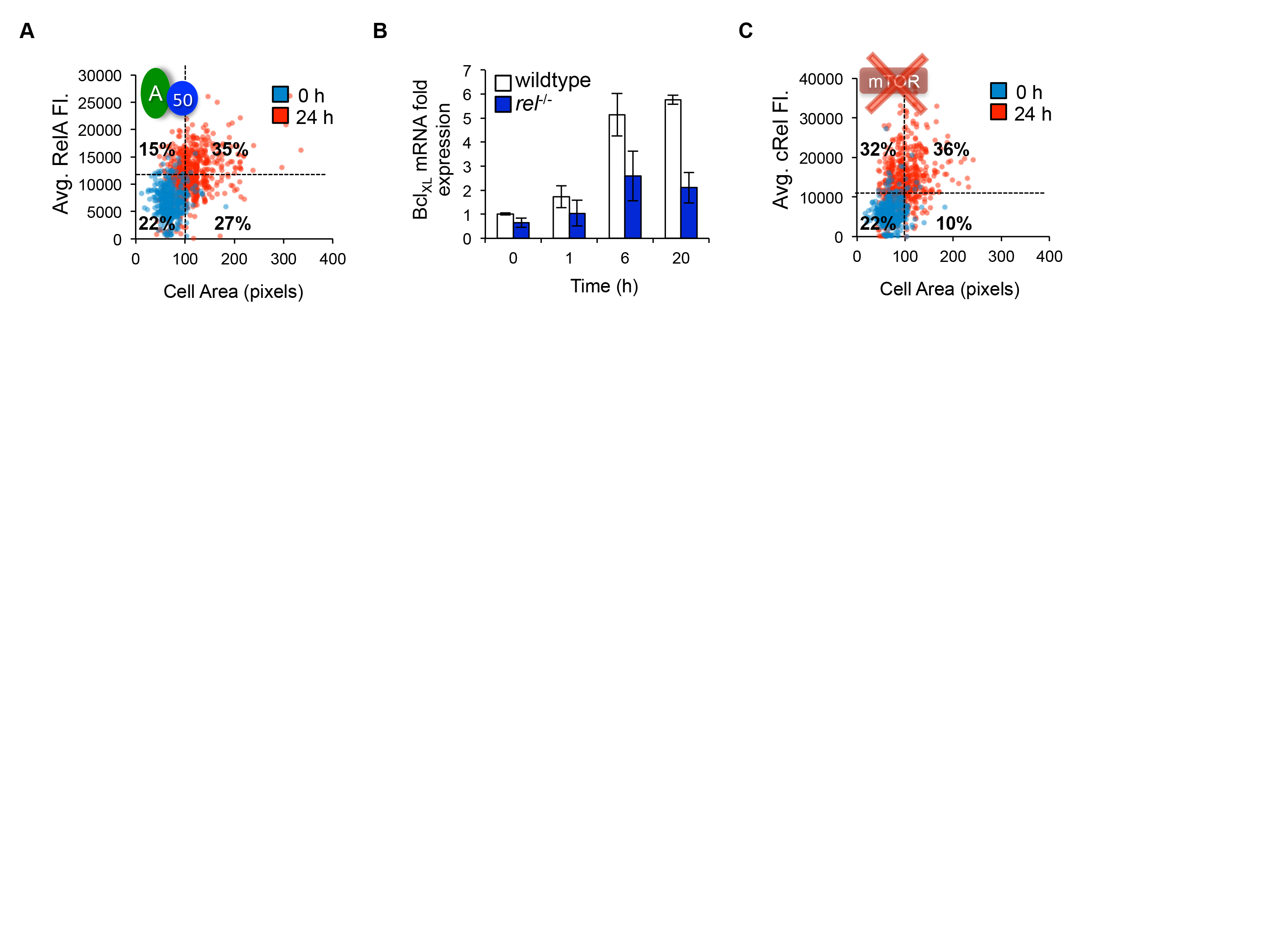

Supplement: Supplementary file 5 [file msb0011-0783-sd5.png]

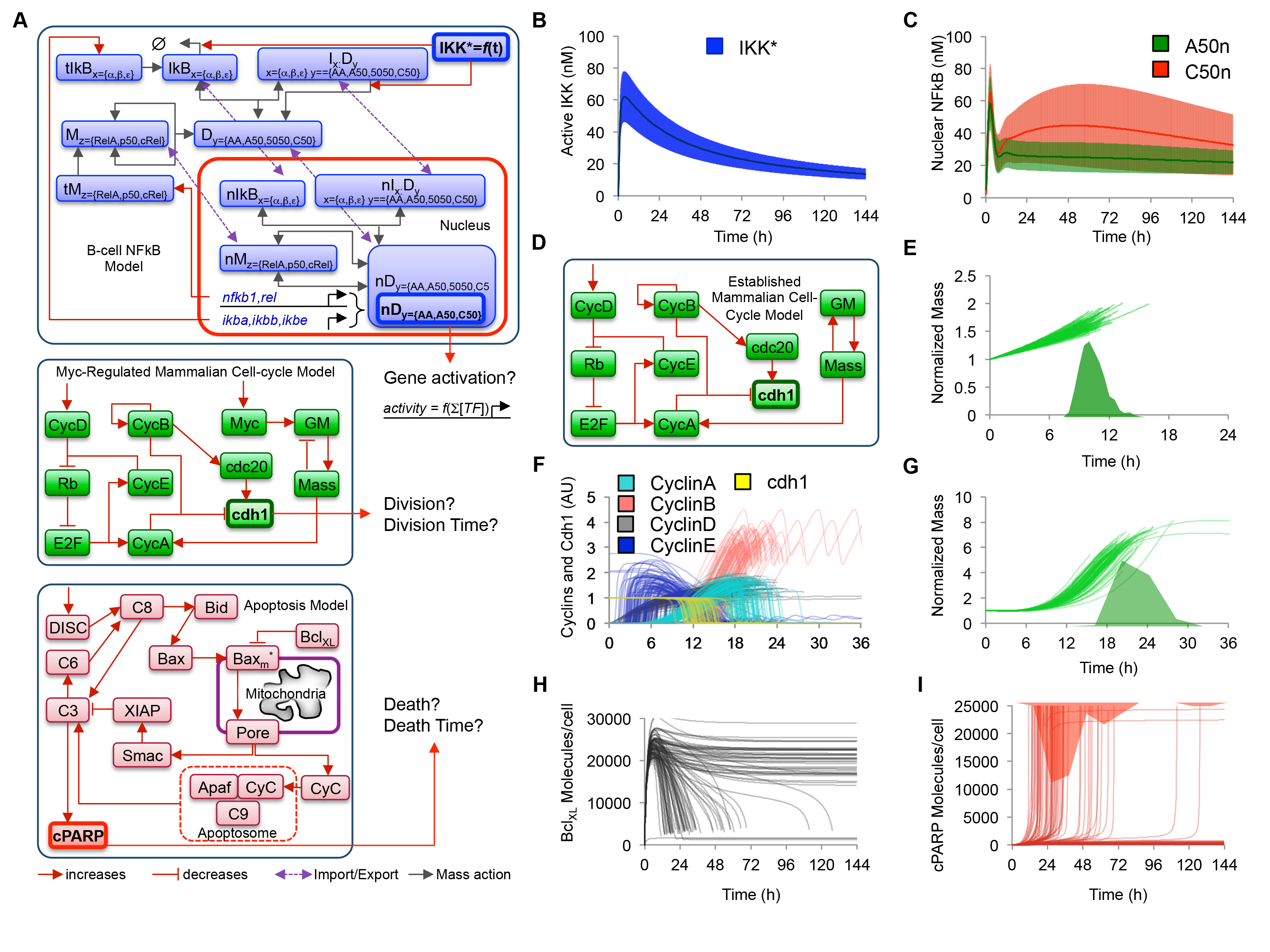

Supplement: Supplementary file 6 [file msb0011-0783-sd6.png]

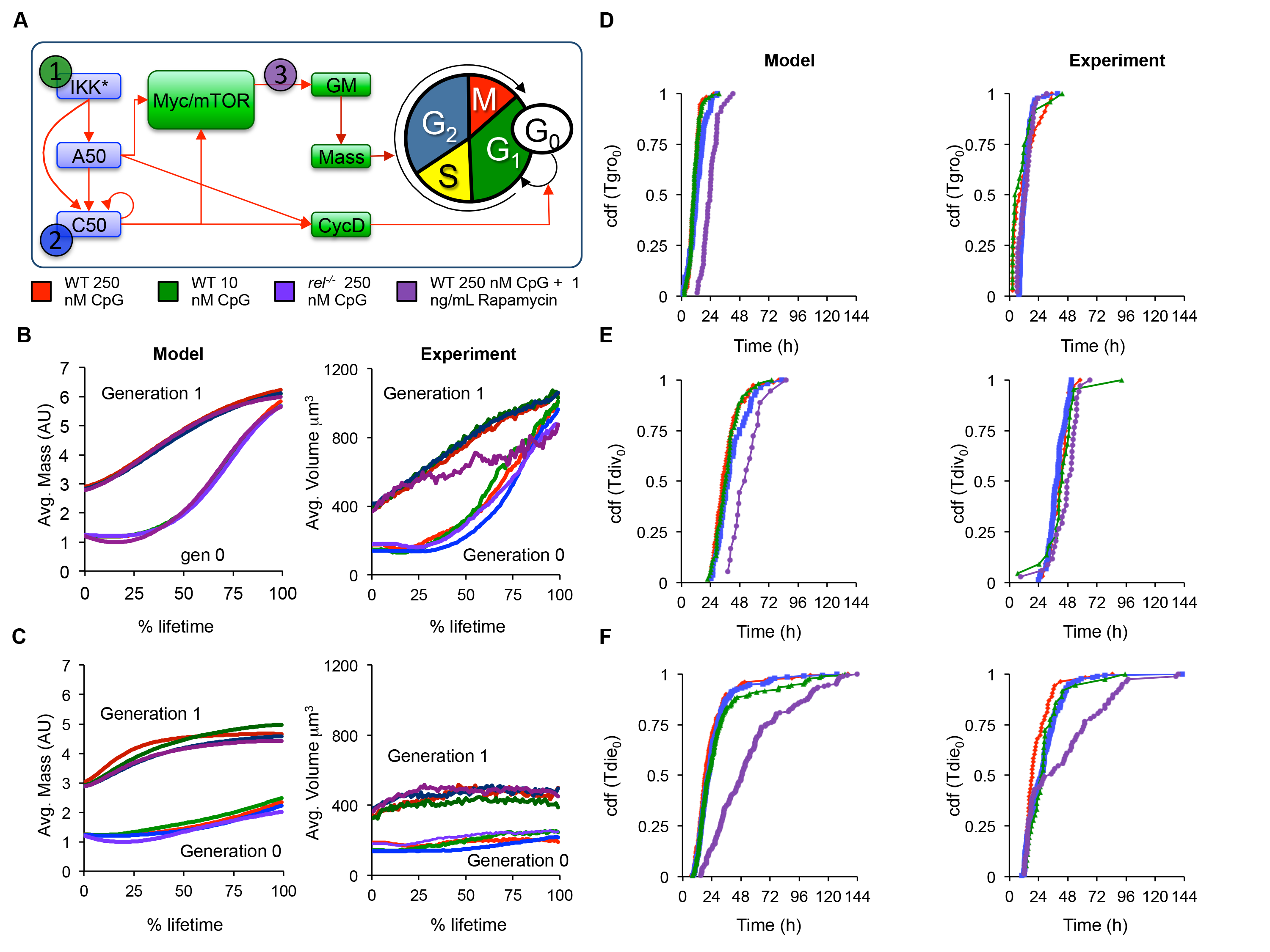

Supplement: Supplementary file 7 [file msb0011-0783-sd7.png]

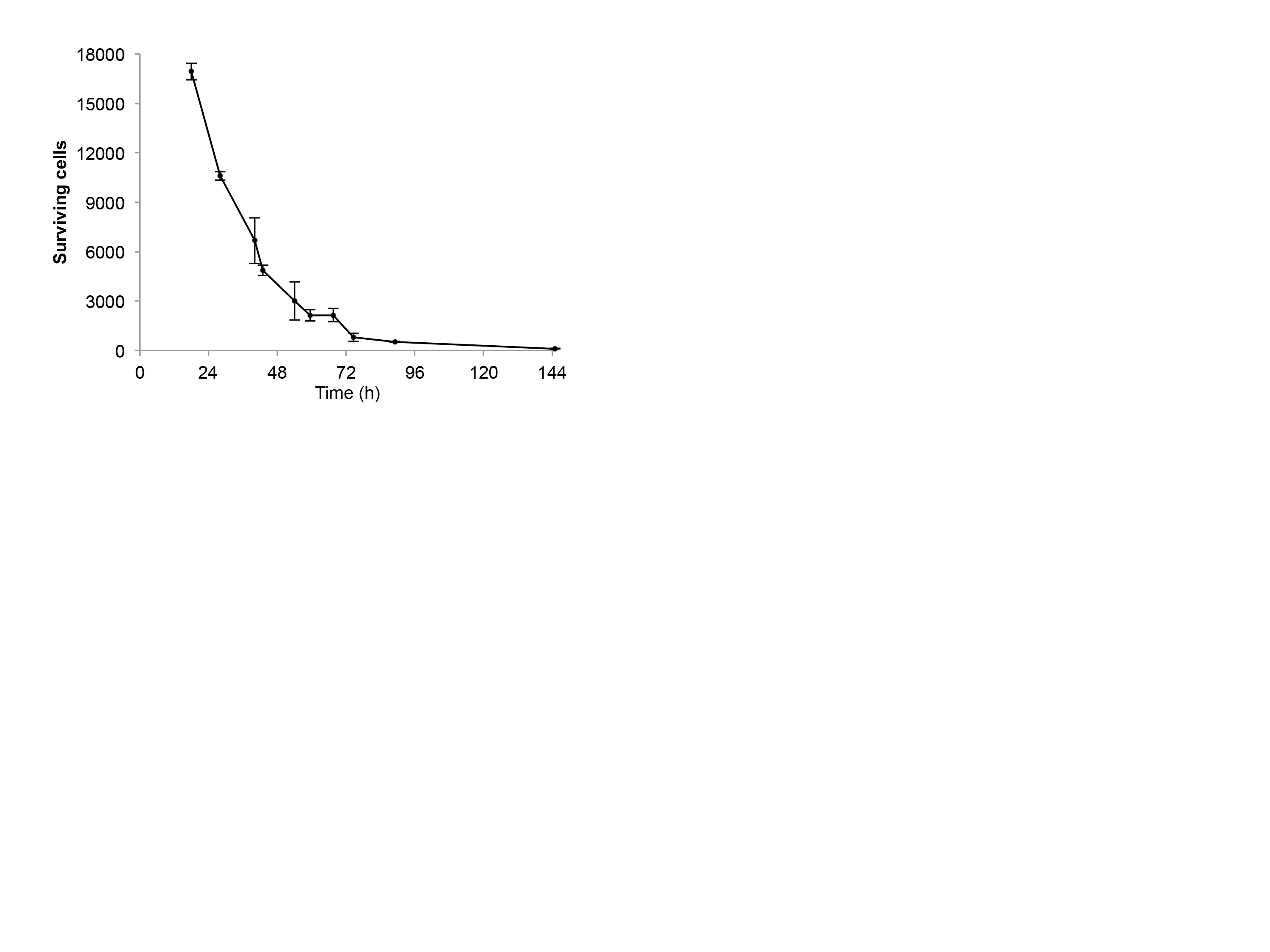

Supplement: Supplementary file 8 [file msb0011-0783-sd8.png]

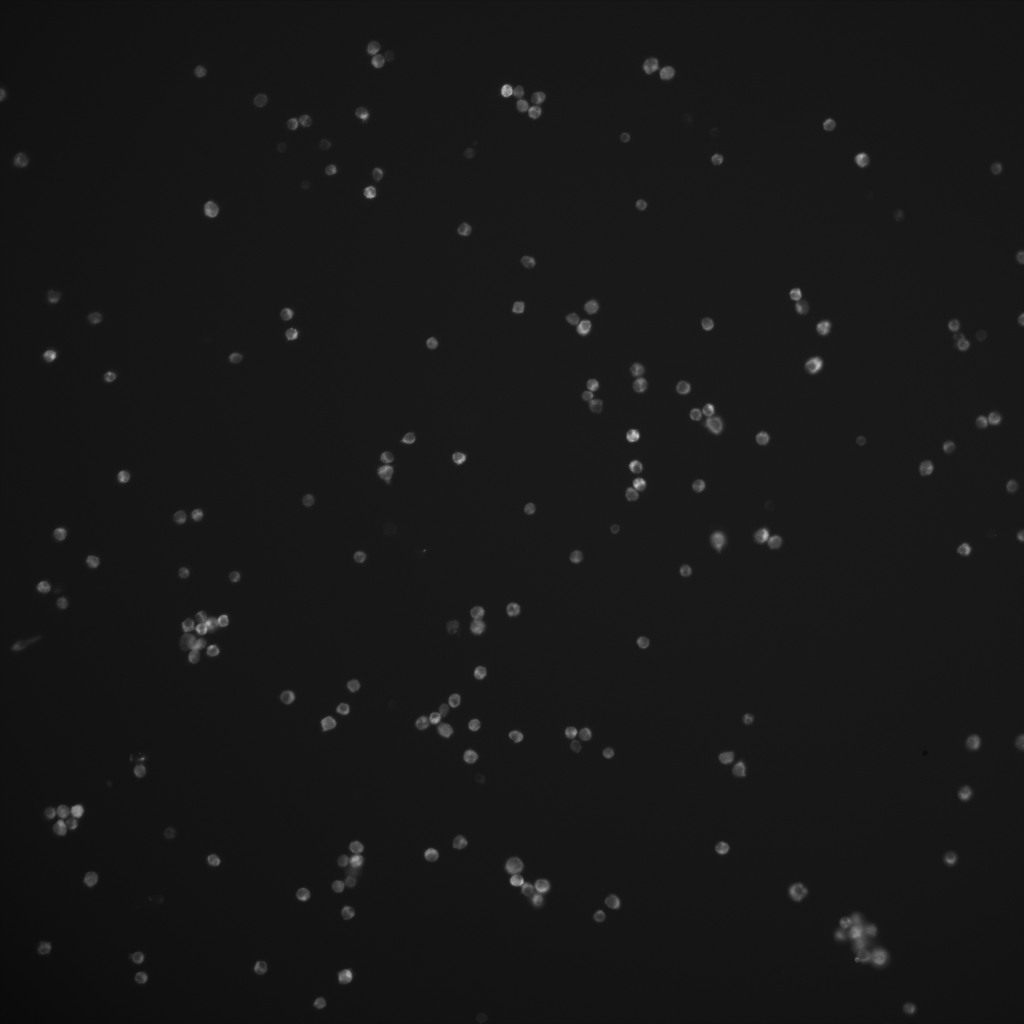

Supplement: Supplementary file 18 [file msb0011-0783-sd18.zip › Snap-28_c4_ORG.png]

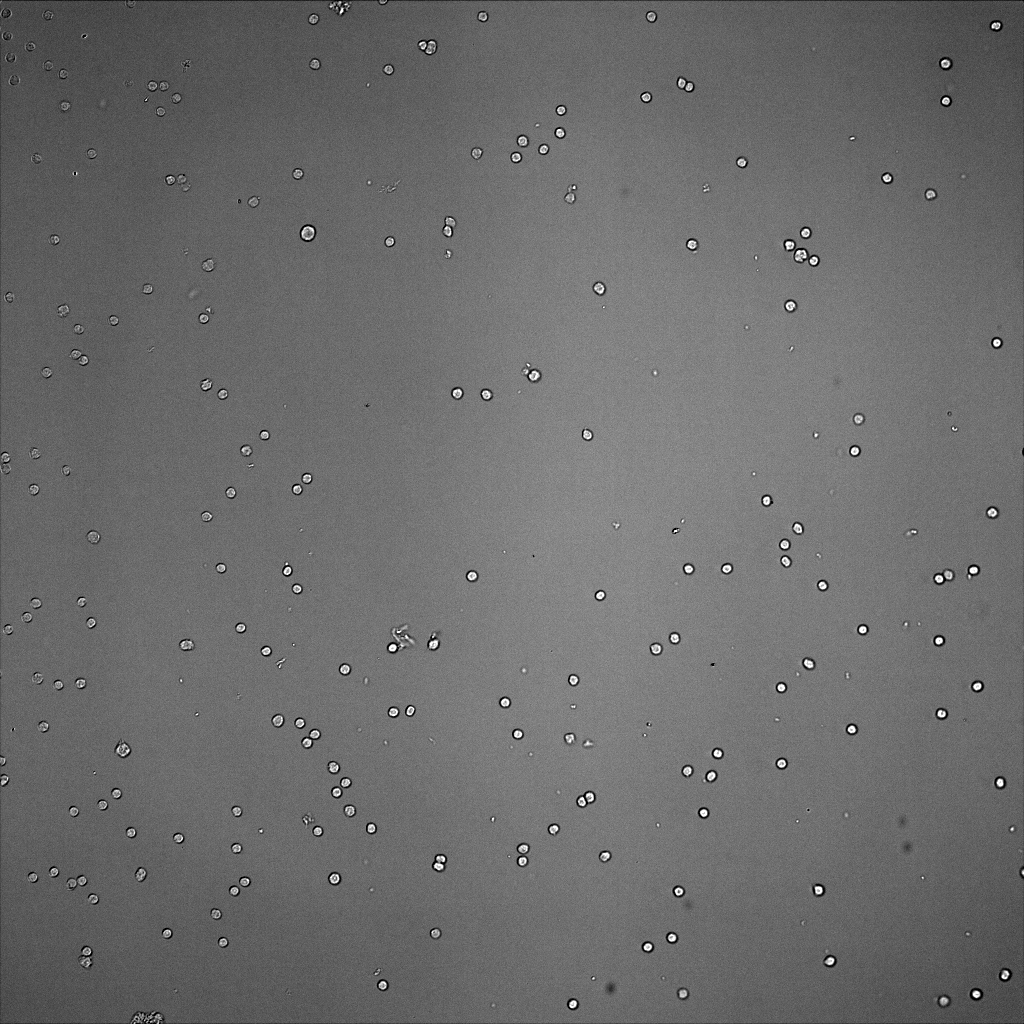

Supplement: Supplementary file 18 [file msb0011-0783-sd18.zip › Snap-15_c1_ORG.png]

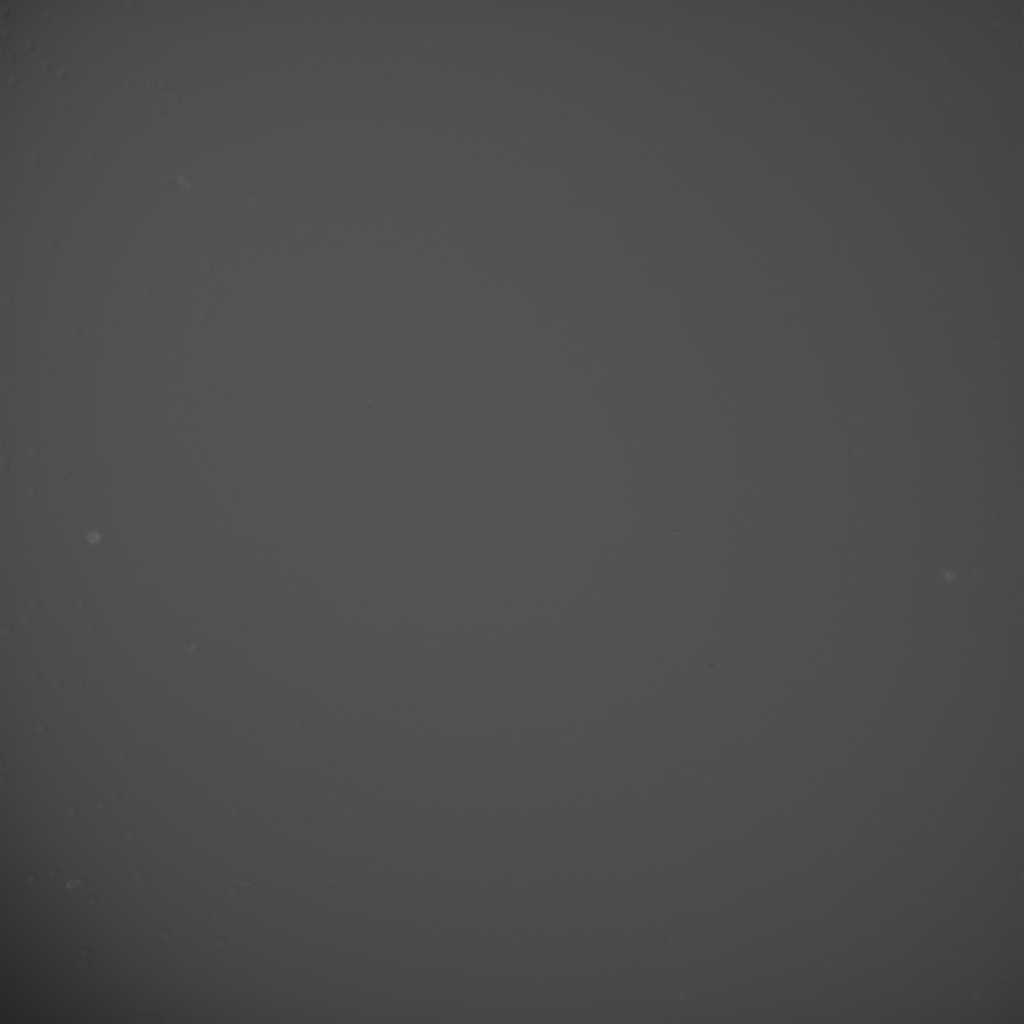

Supplement: Supplementary file 18 [file msb0011-0783-sd18.zip › Snap-15_c2_ORG.png]

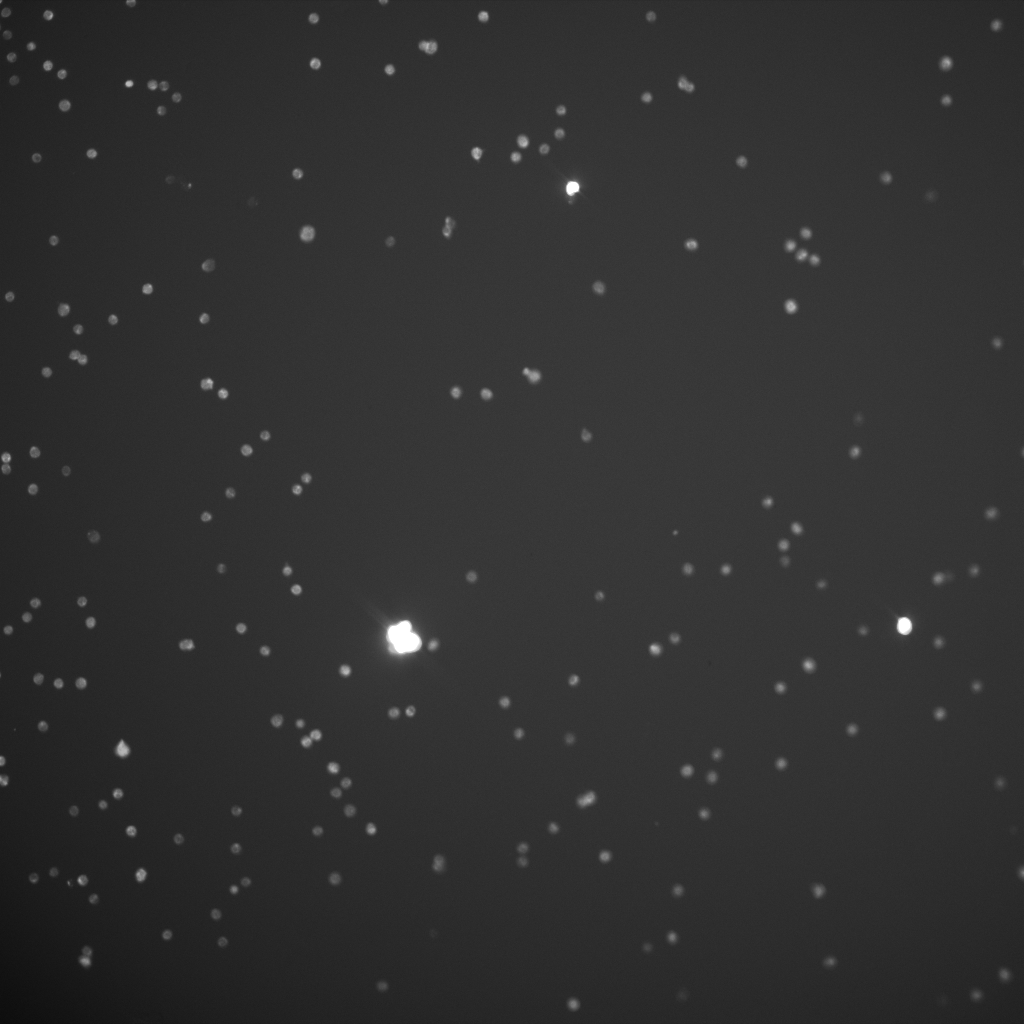

Supplement: Supplementary file 18 [file msb0011-0783-sd18.zip › Snap-15_c3_ORG.png]

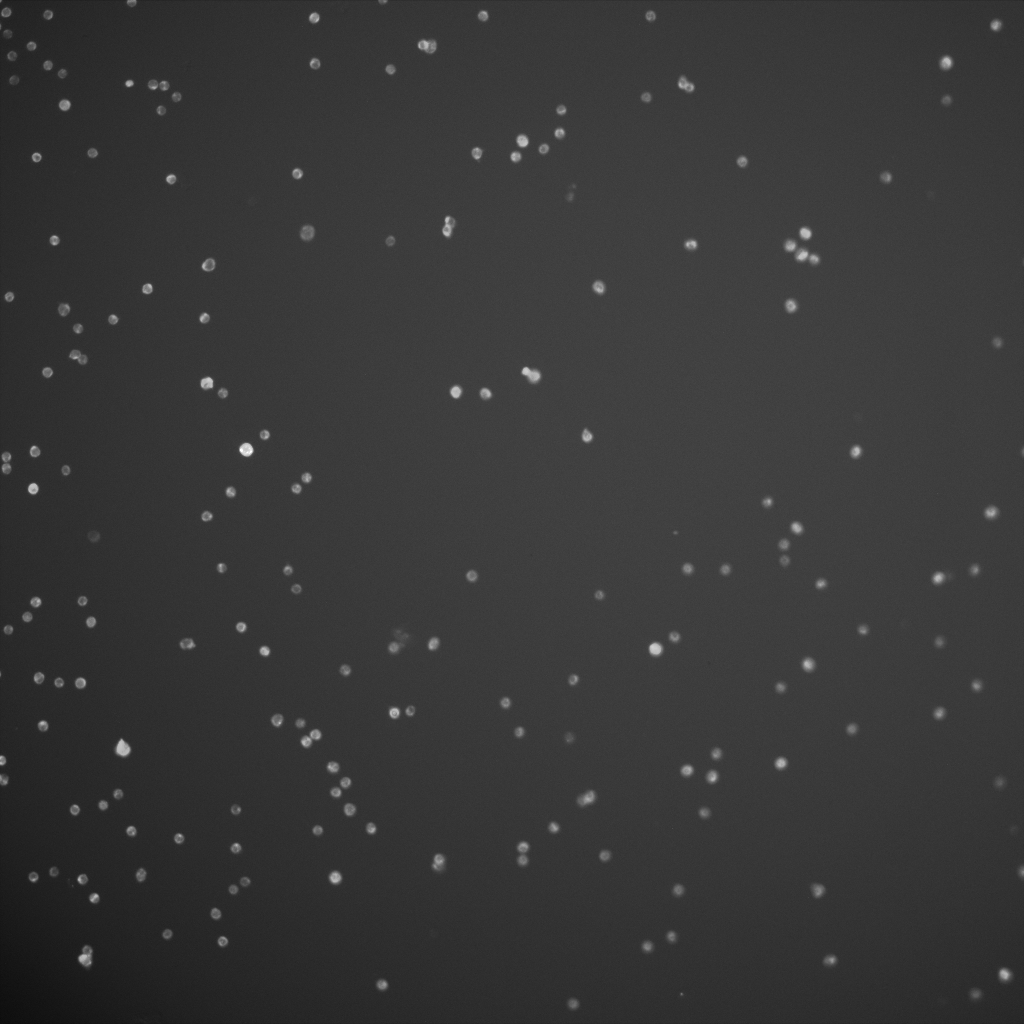

Supplement: Supplementary file 18 [file msb0011-0783-sd18.zip › Snap-15_c4_ORG.png]

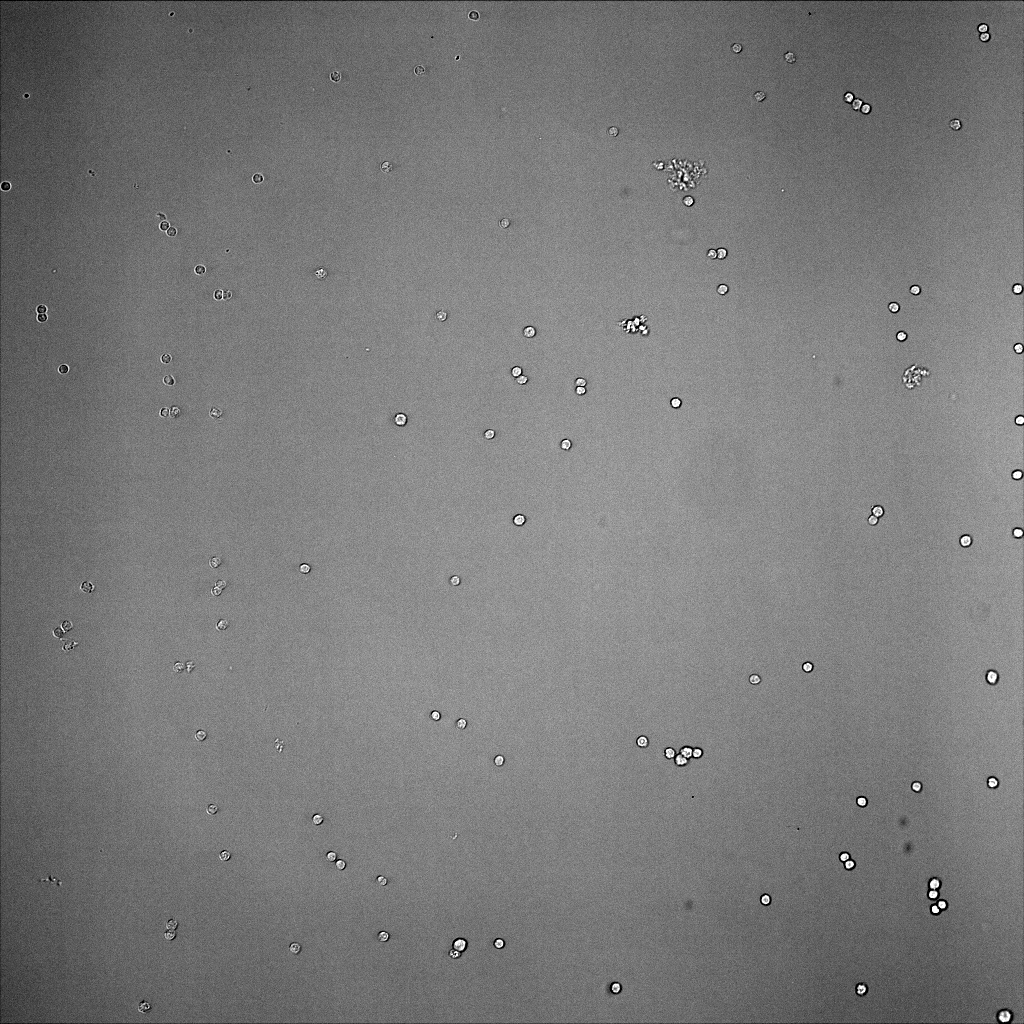

Supplement: Supplementary file 18 [file msb0011-0783-sd18.zip › Snap-16_c1_ORG.png]

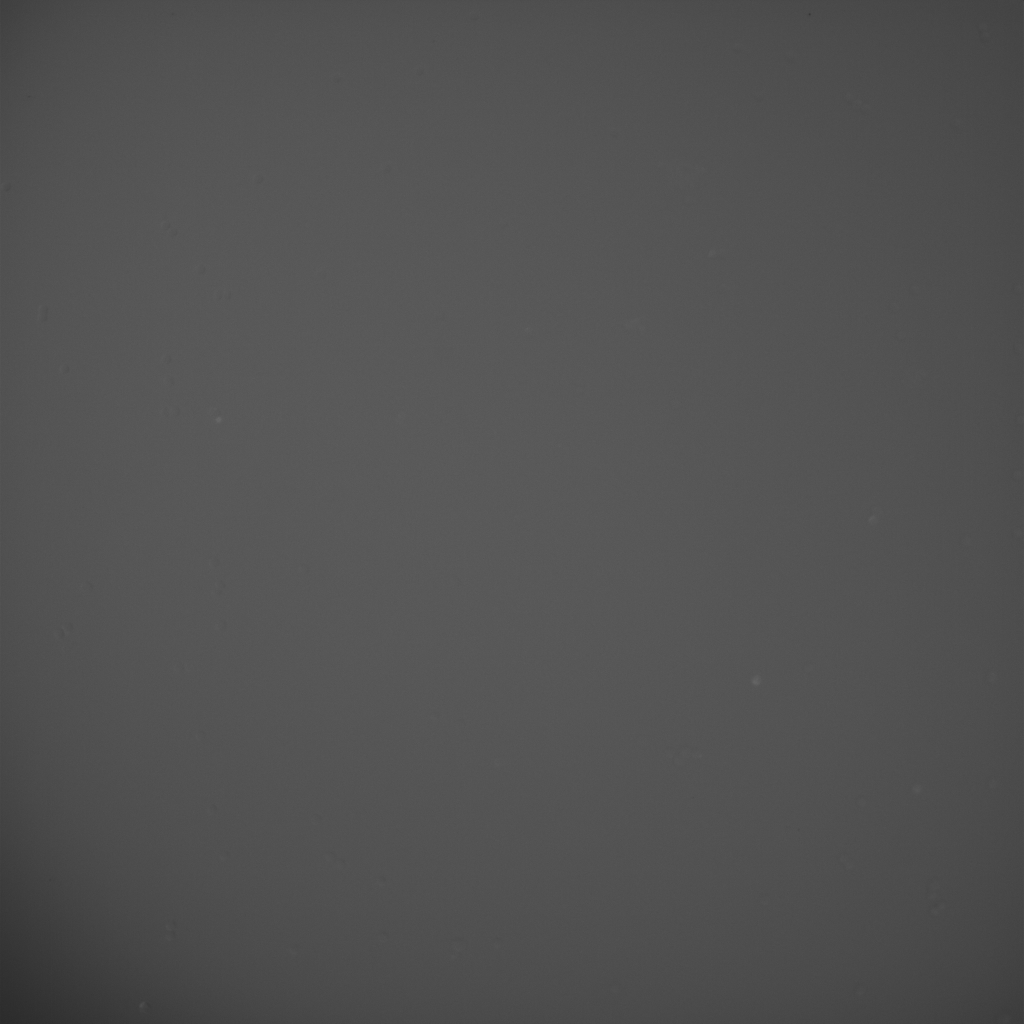

Supplement: Supplementary file 18 [file msb0011-0783-sd18.zip › Snap-16_c2_ORG.png]

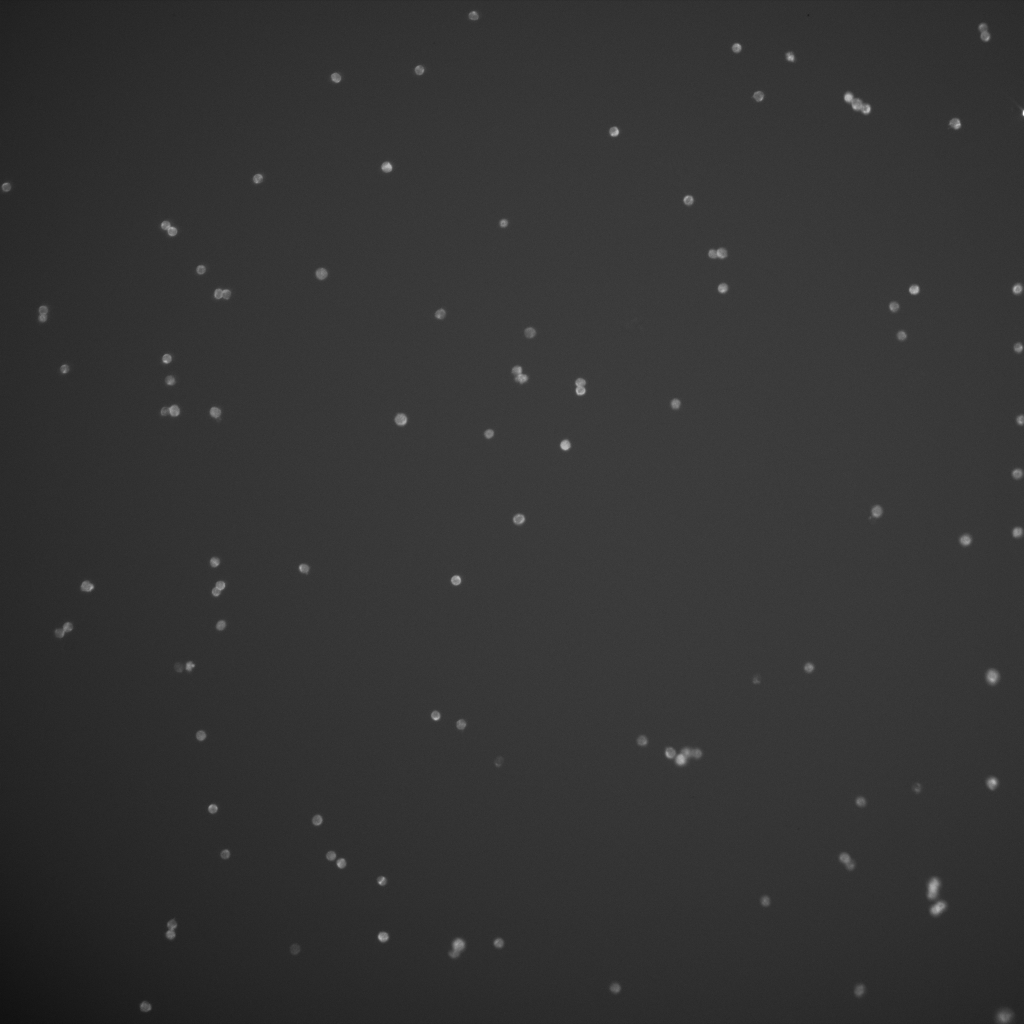

Supplement: Supplementary file 18 [file msb0011-0783-sd18.zip › Snap-16_c3_ORG.png]

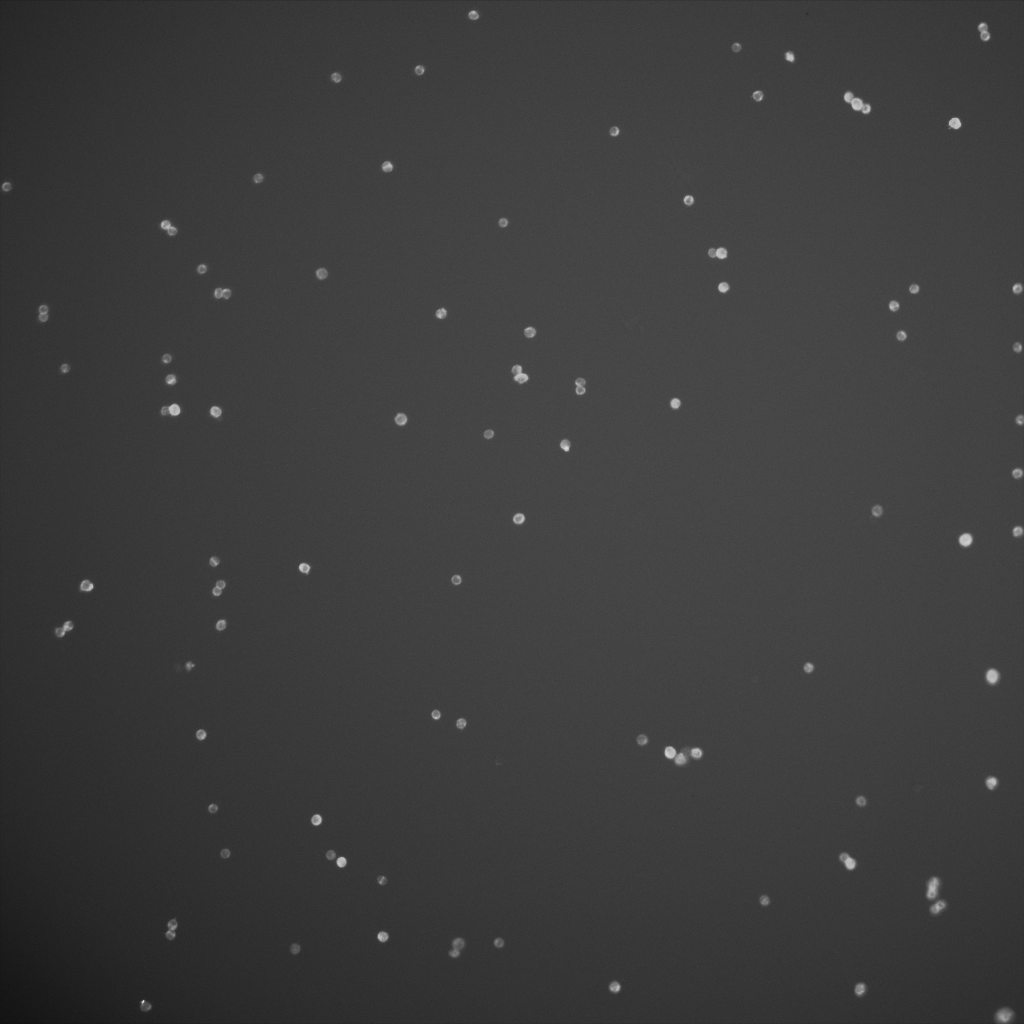

Supplement: Supplementary file 18 [file msb0011-0783-sd18.zip › Snap-16_c4_ORG.png]

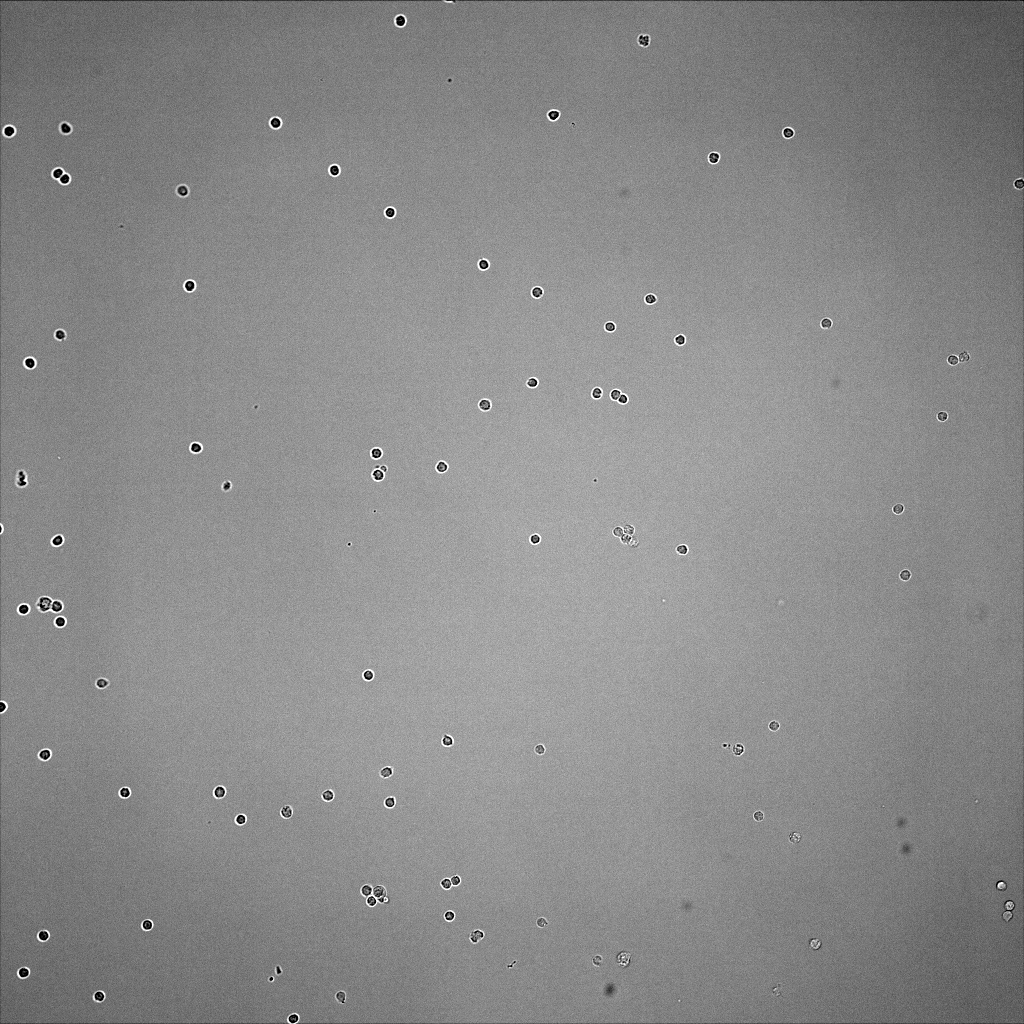

Supplement: Supplementary file 18 [file msb0011-0783-sd18.zip › Snap-17_c1_ORG.png]

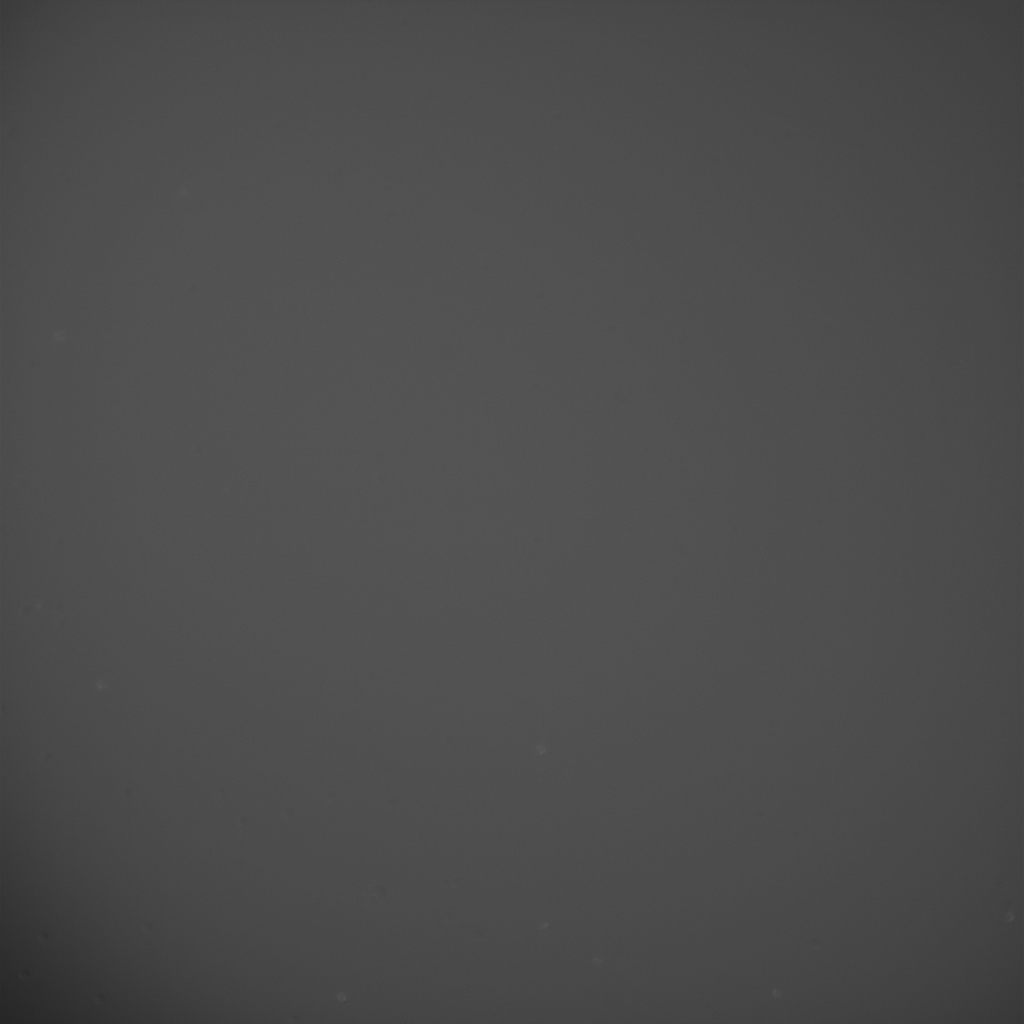

Supplement: Supplementary file 18 [file msb0011-0783-sd18.zip › Snap-17_c2_ORG.png]

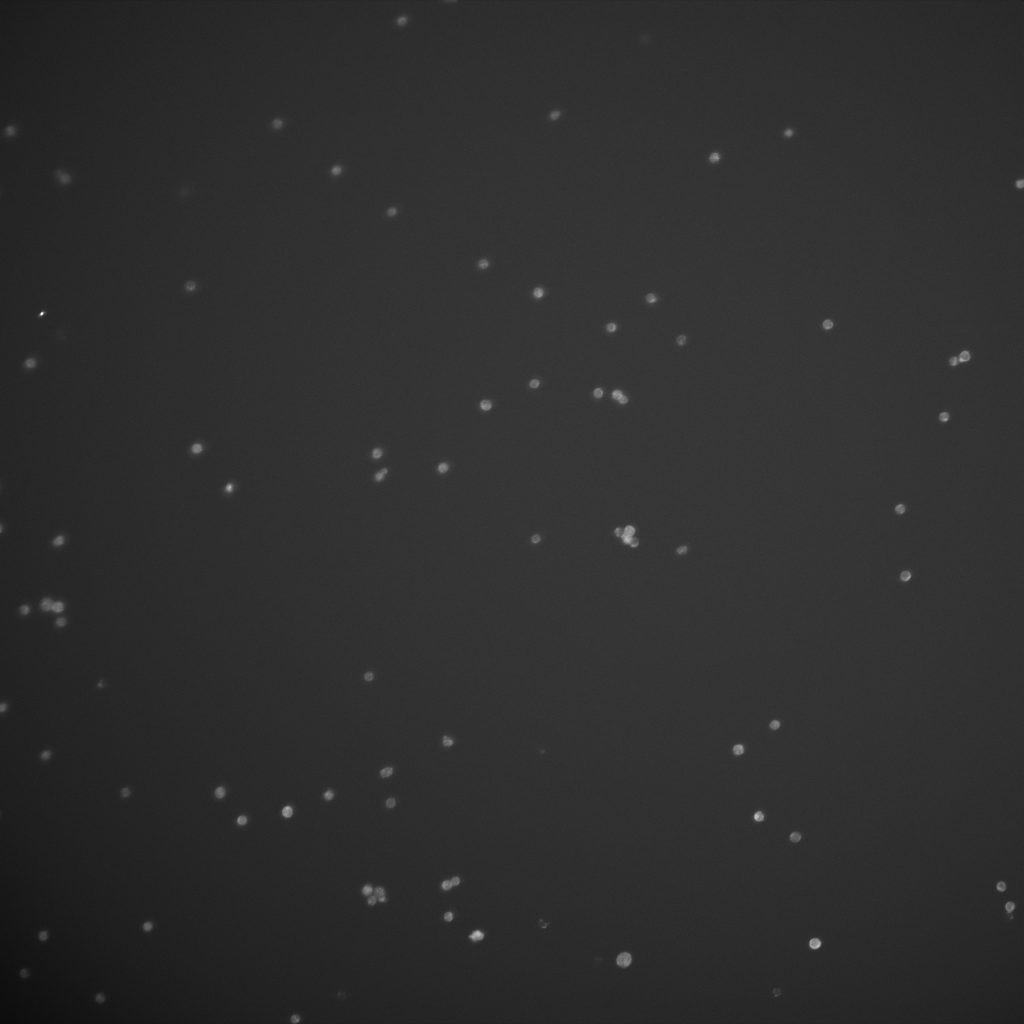

Supplement: Supplementary file 18 [file msb0011-0783-sd18.zip › Snap-17_c3_ORG.png]

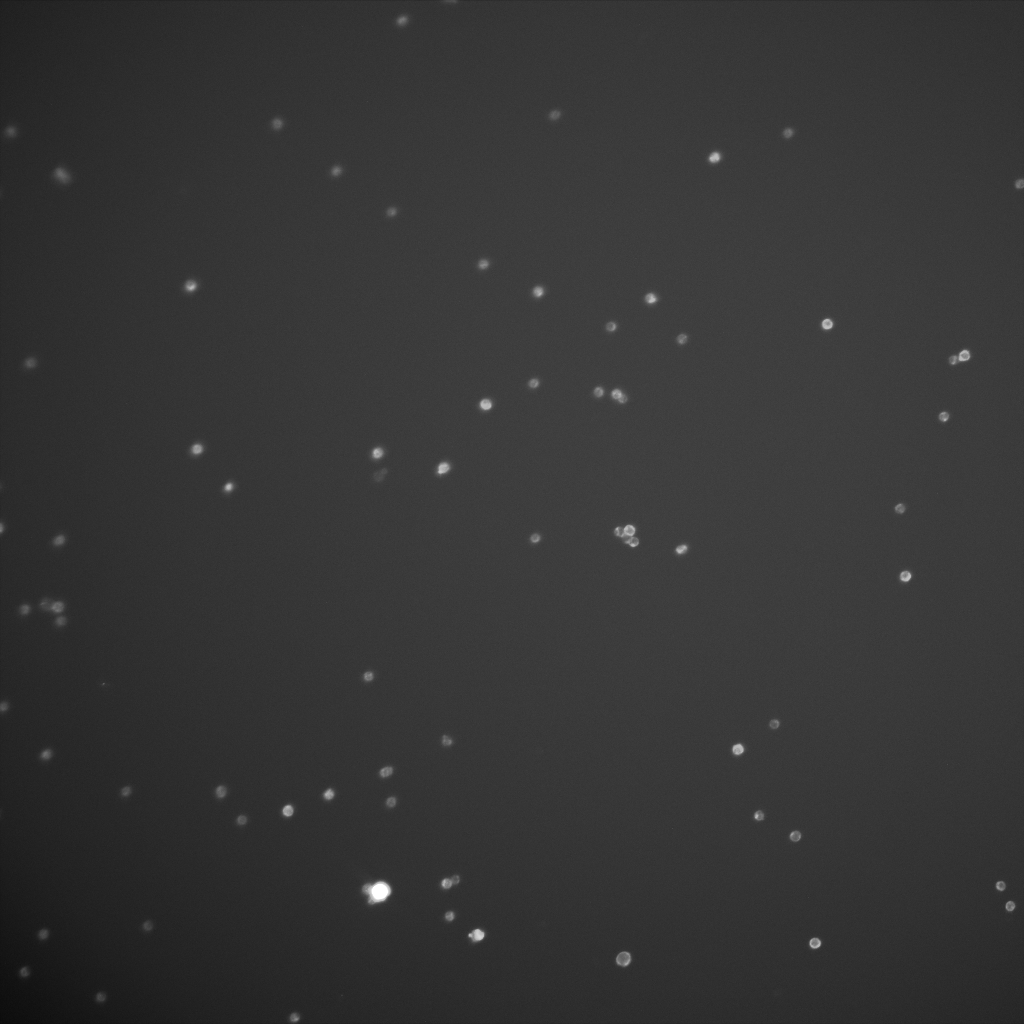

Supplement: Supplementary file 18 [file msb0011-0783-sd18.zip › Snap-17_c4_ORG.png]

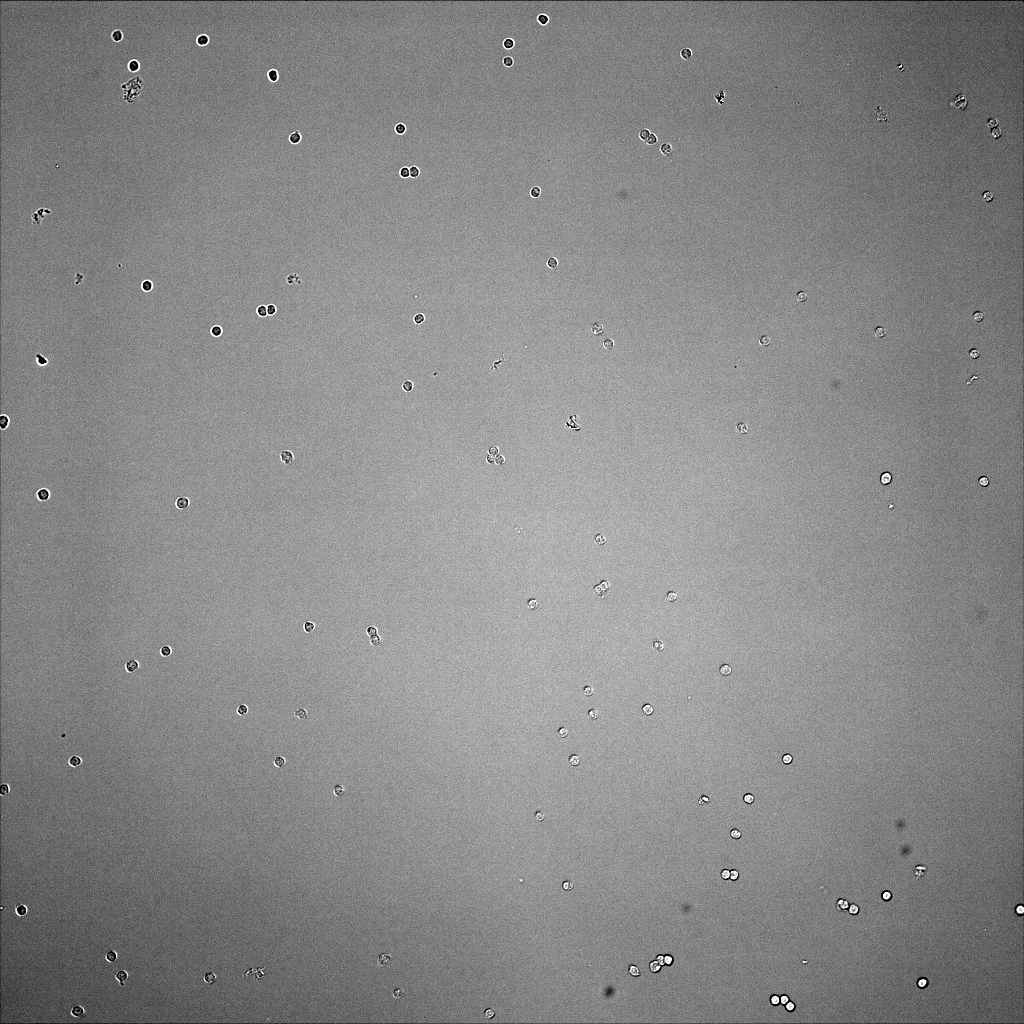

Supplement: Supplementary file 18 [file msb0011-0783-sd18.zip › Snap-18_c1_ORG.png]

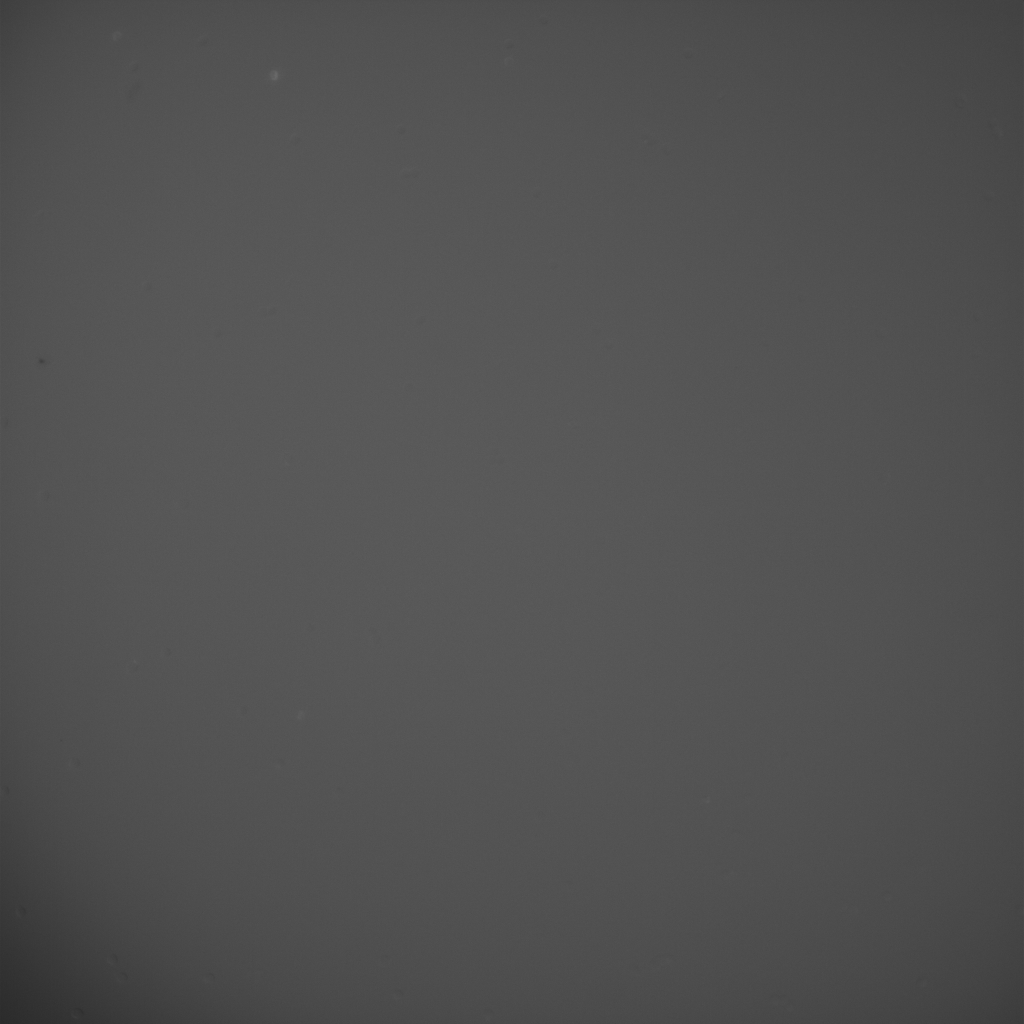

Supplement: Supplementary file 18 [file msb0011-0783-sd18.zip › Snap-18_c2_ORG.png]

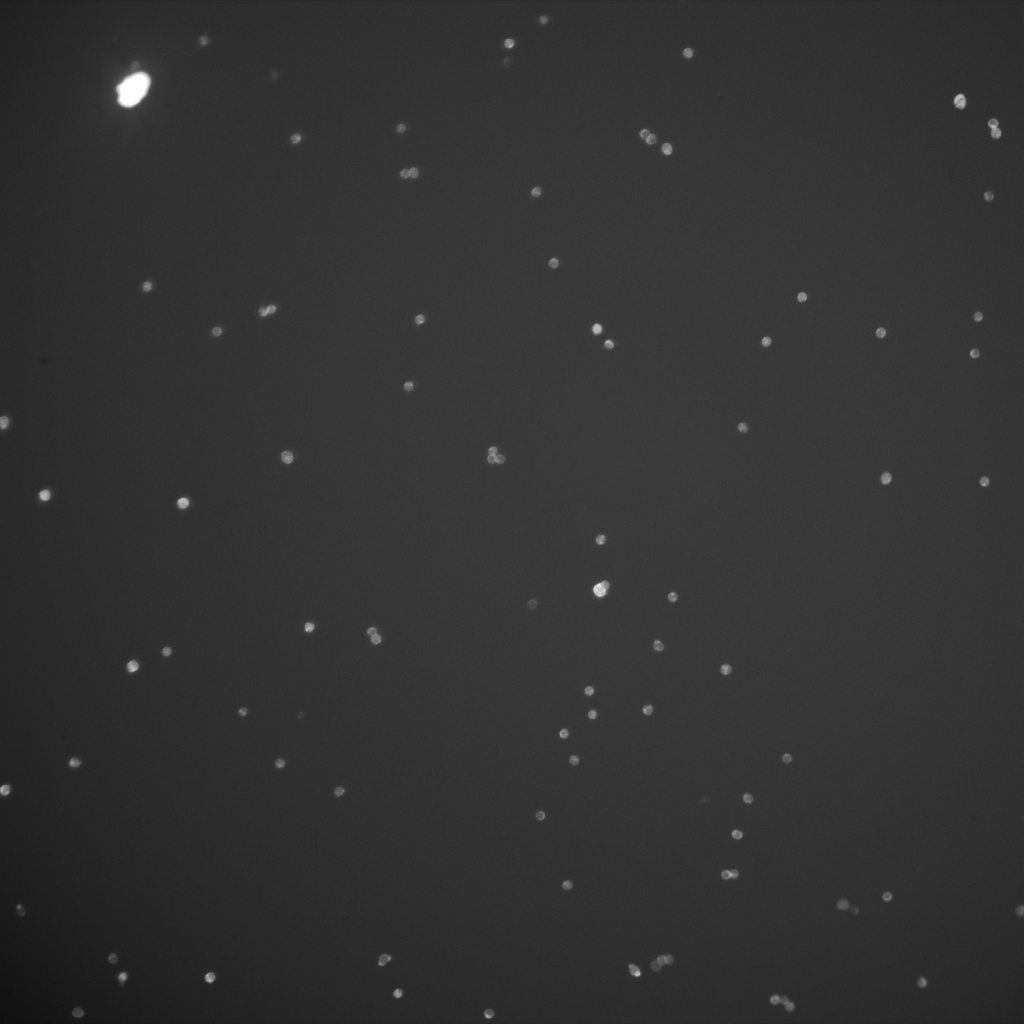

Supplement: Supplementary file 18 [file msb0011-0783-sd18.zip › Snap-18_c3_ORG.png]

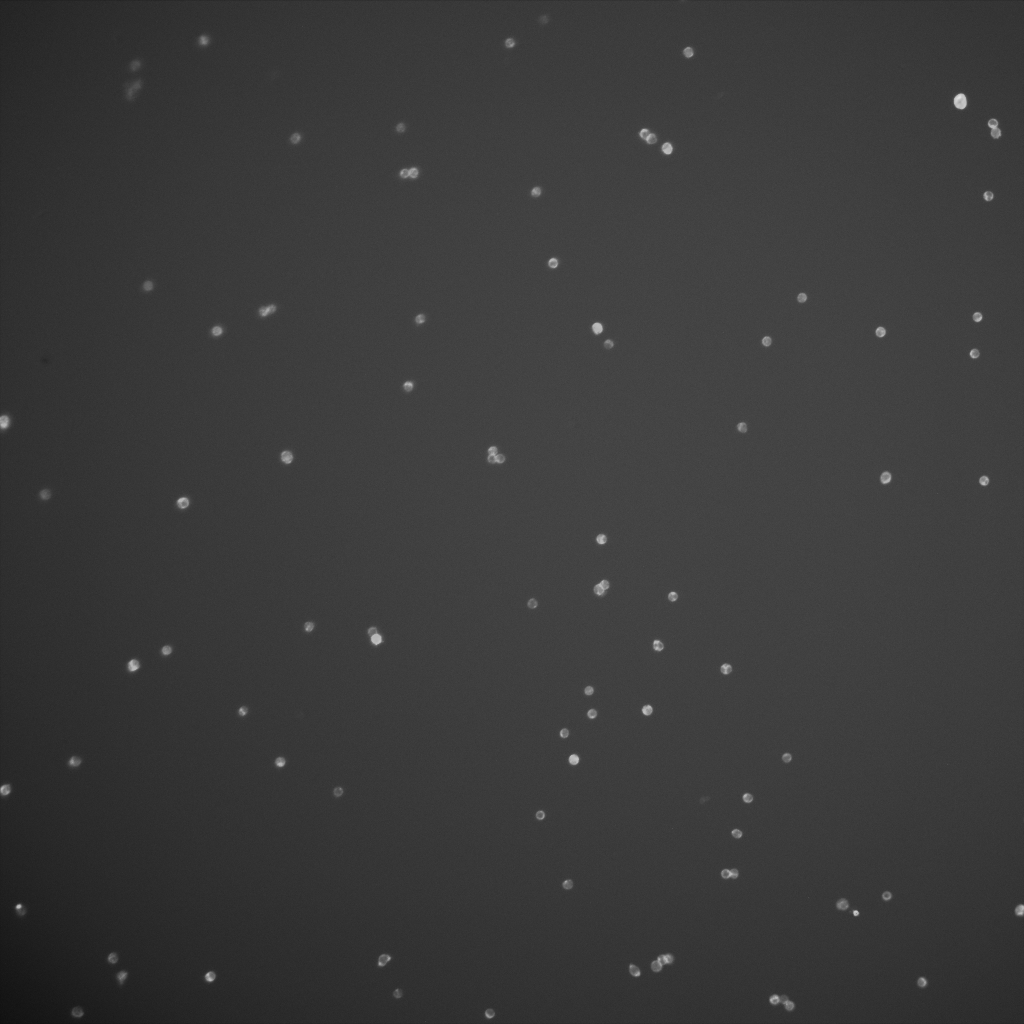

Supplement: Supplementary file 18 [file msb0011-0783-sd18.zip › Snap-18_c4_ORG.png]

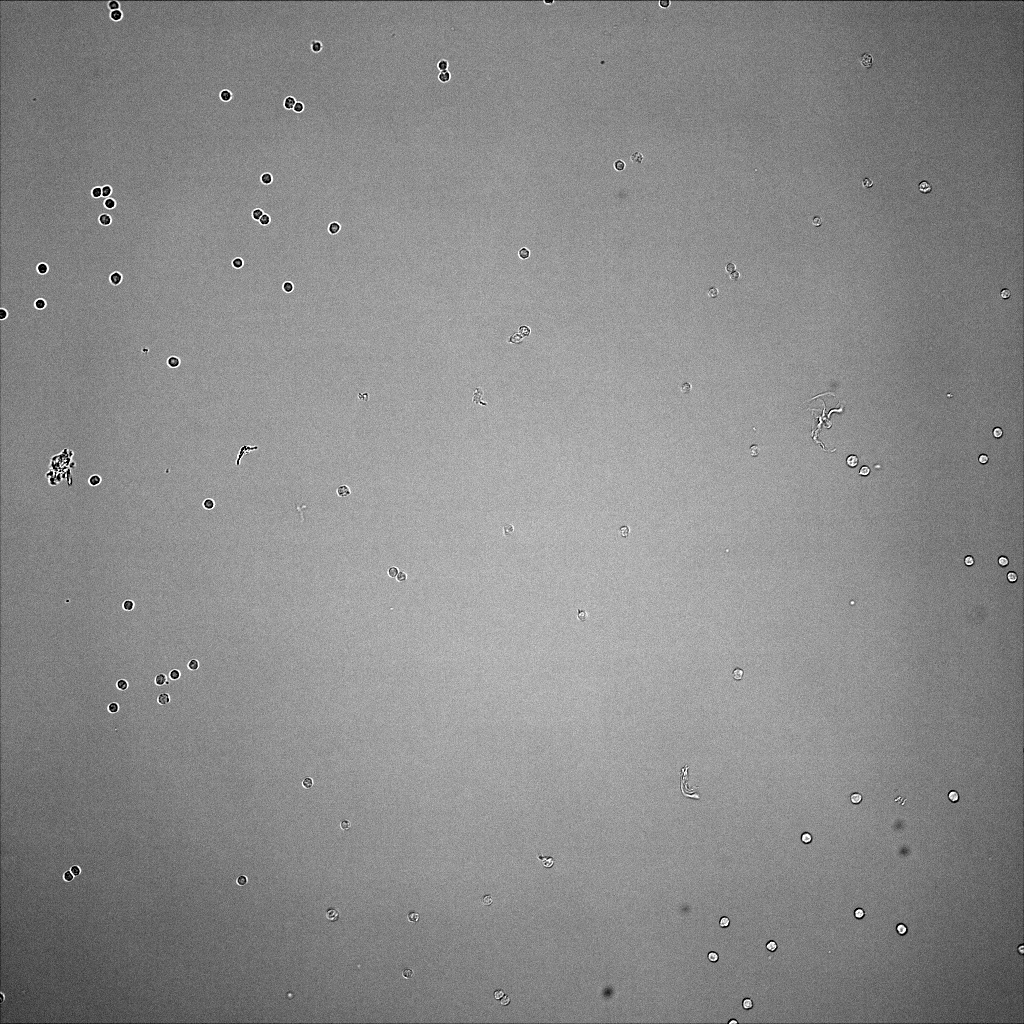

Supplement: Supplementary file 18 [file msb0011-0783-sd18.zip › Snap-19_c1_ORG.png]

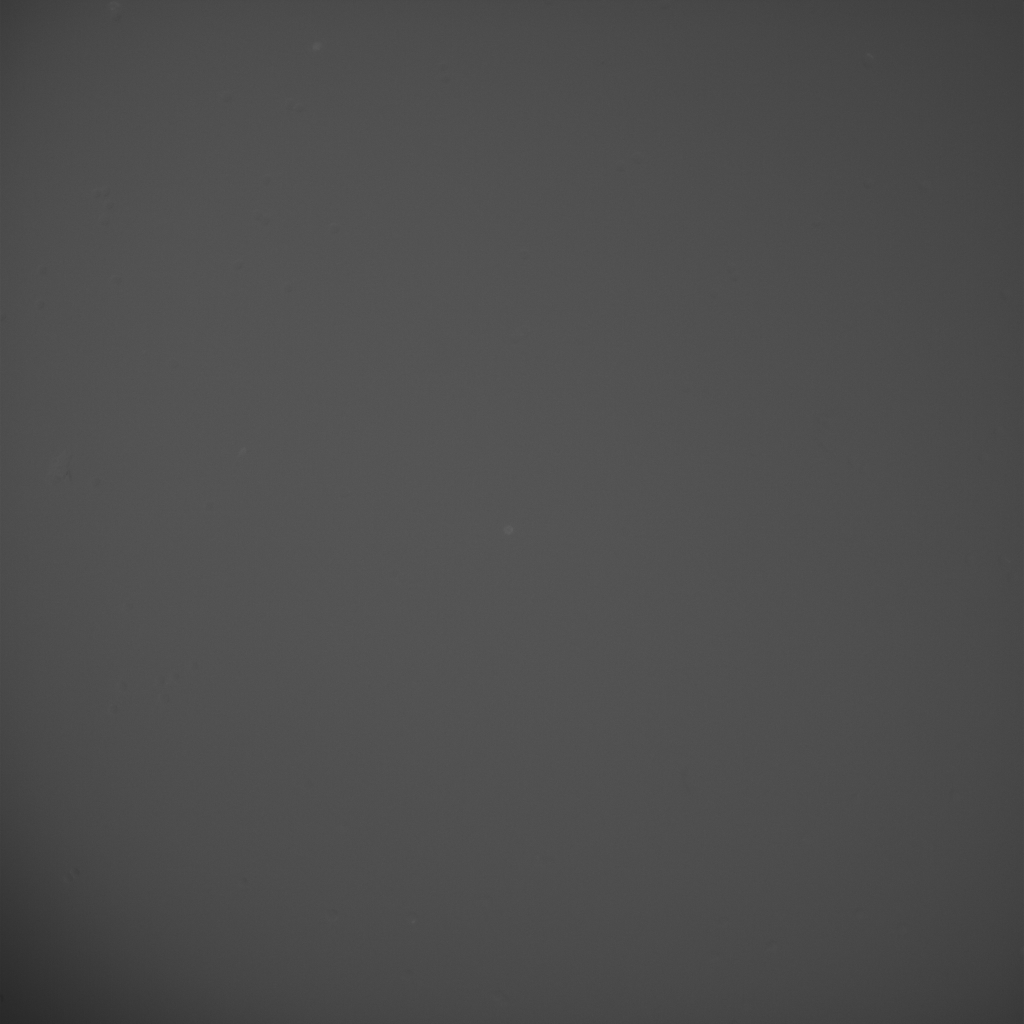

Supplement: Supplementary file 18 [file msb0011-0783-sd18.zip › Snap-19_c2_ORG.png]

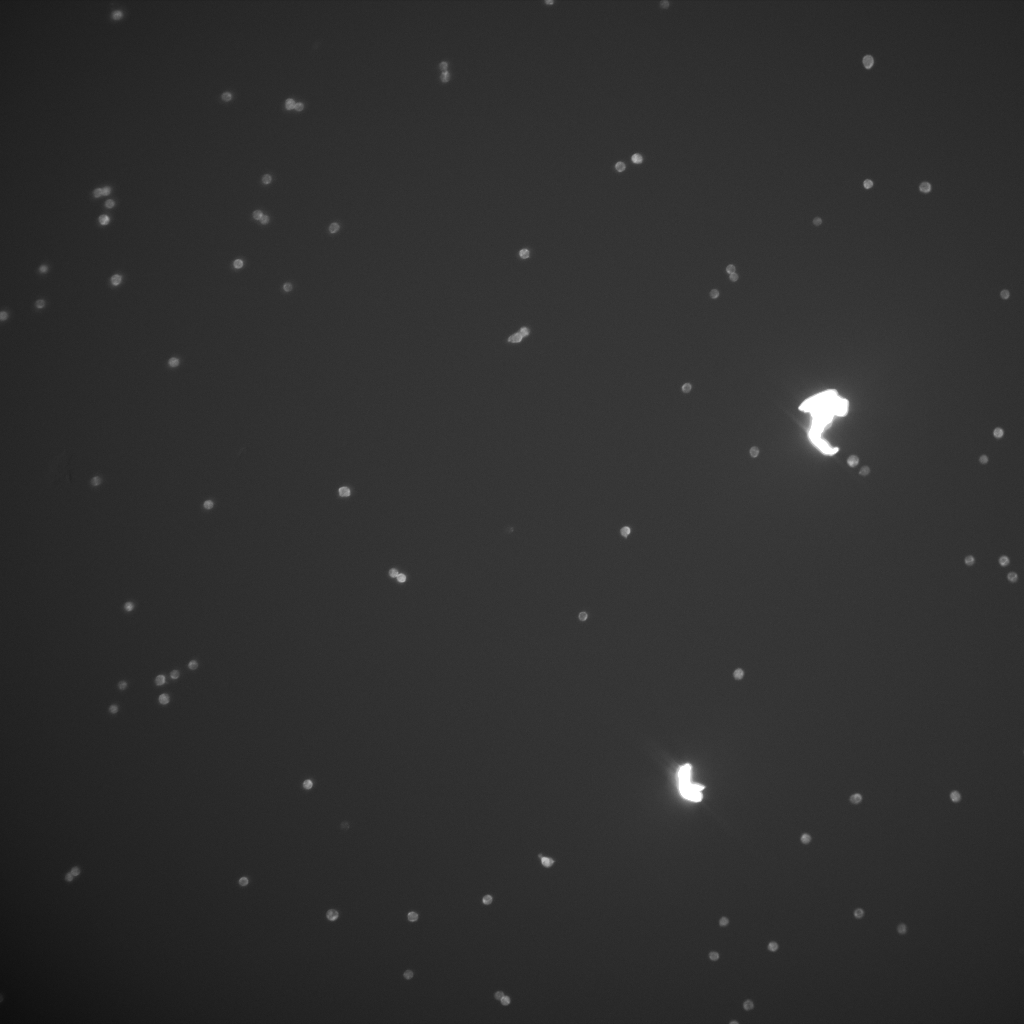

Supplement: Supplementary file 18 [file msb0011-0783-sd18.zip › Snap-19_c3_ORG.png]

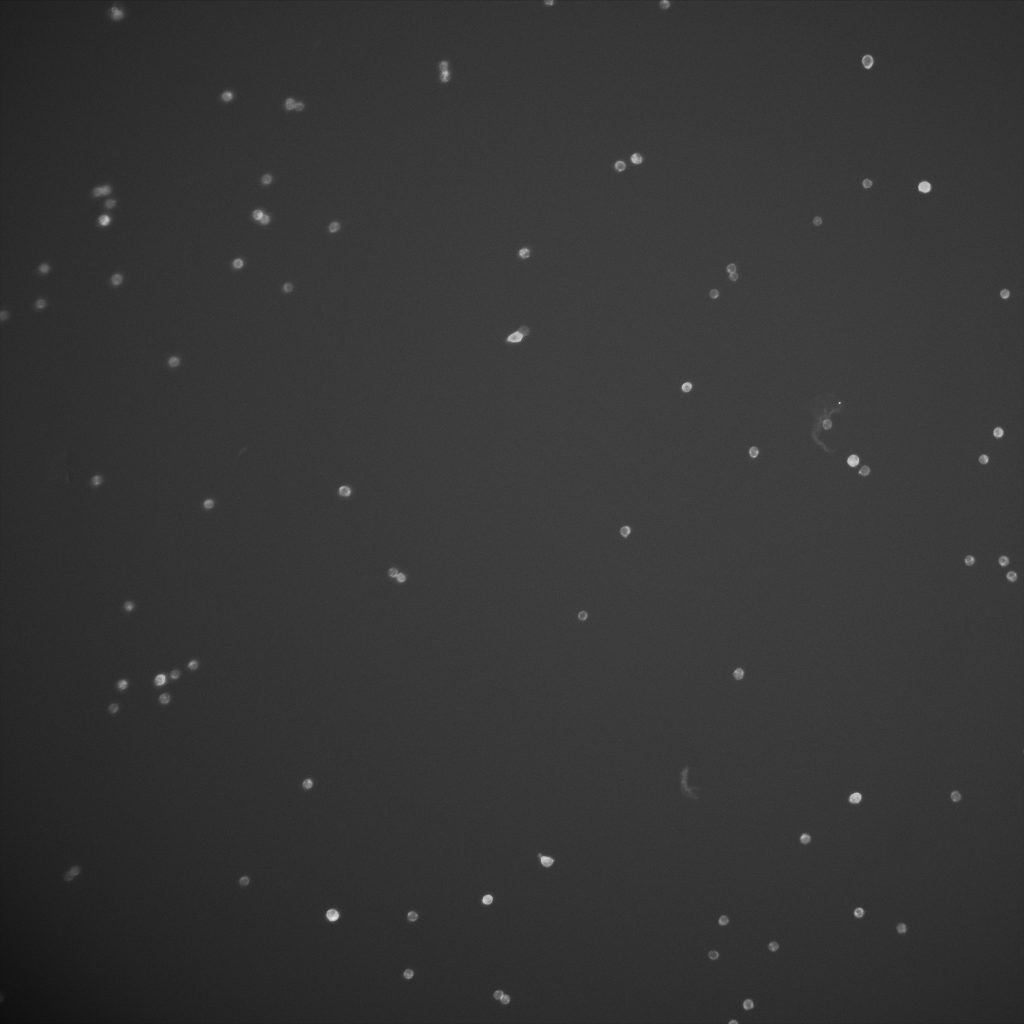

Supplement: Supplementary file 18 [file msb0011-0783-sd18.zip › Snap-19_c4_ORG.png]

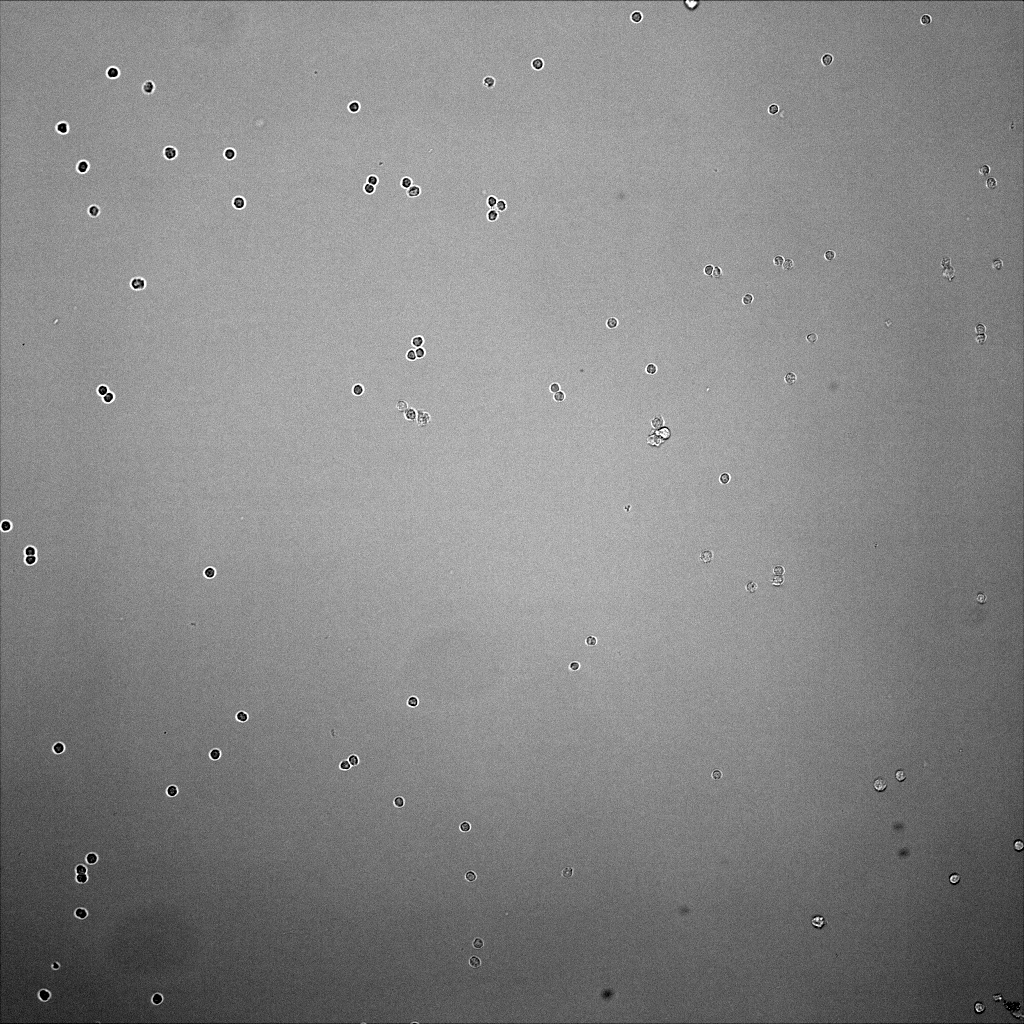

Supplement: Supplementary file 18 [file msb0011-0783-sd18.zip › Snap-20_c1_ORG.png]

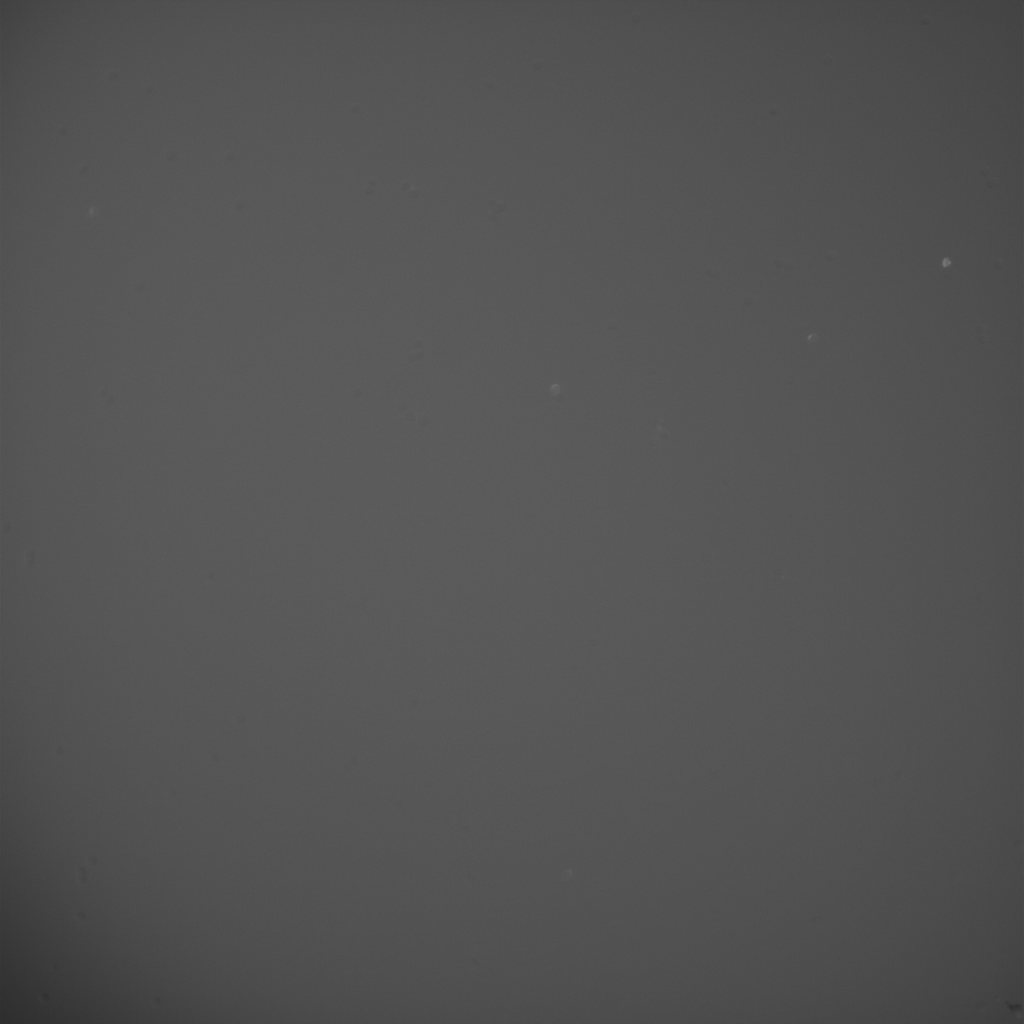

Supplement: Supplementary file 18 [file msb0011-0783-sd18.zip › Snap-20_c2_ORG.png]

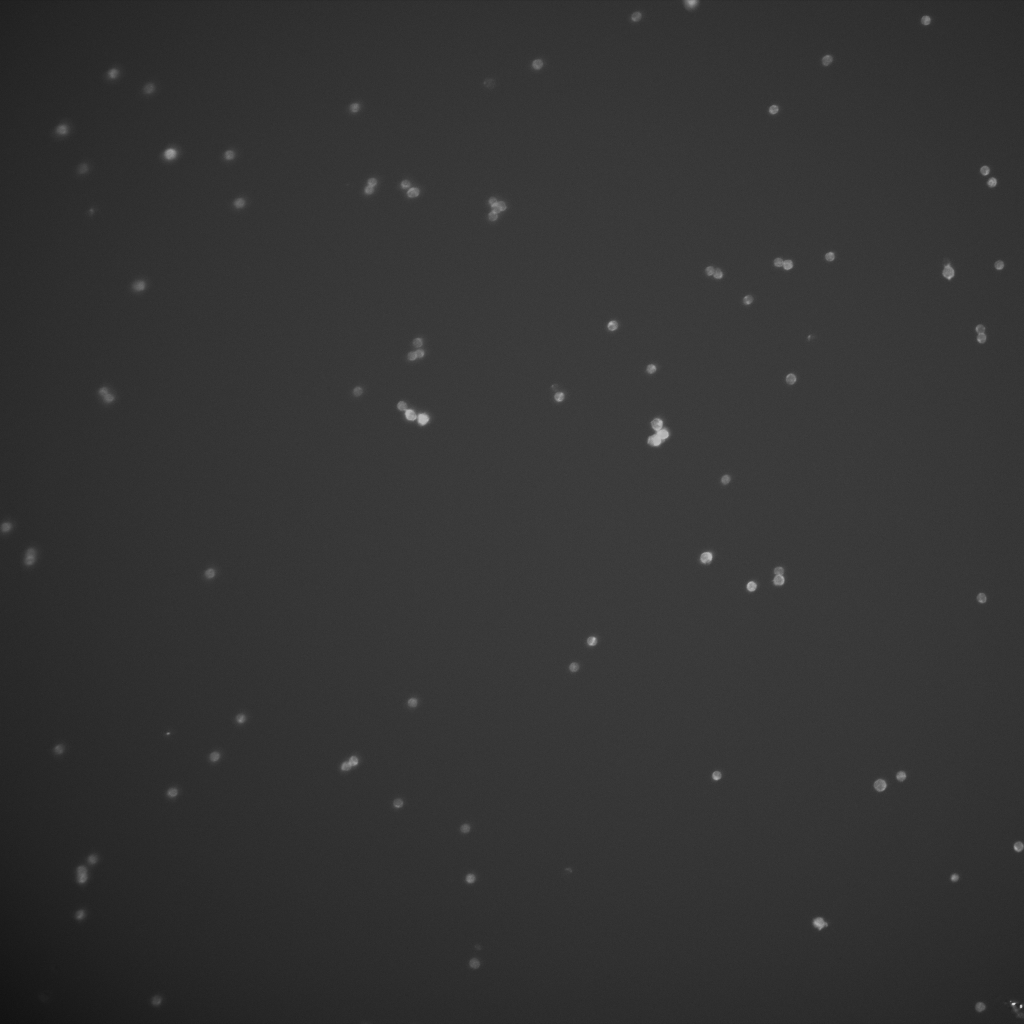

Supplement: Supplementary file 18 [file msb0011-0783-sd18.zip › Snap-20_c3_ORG.png]

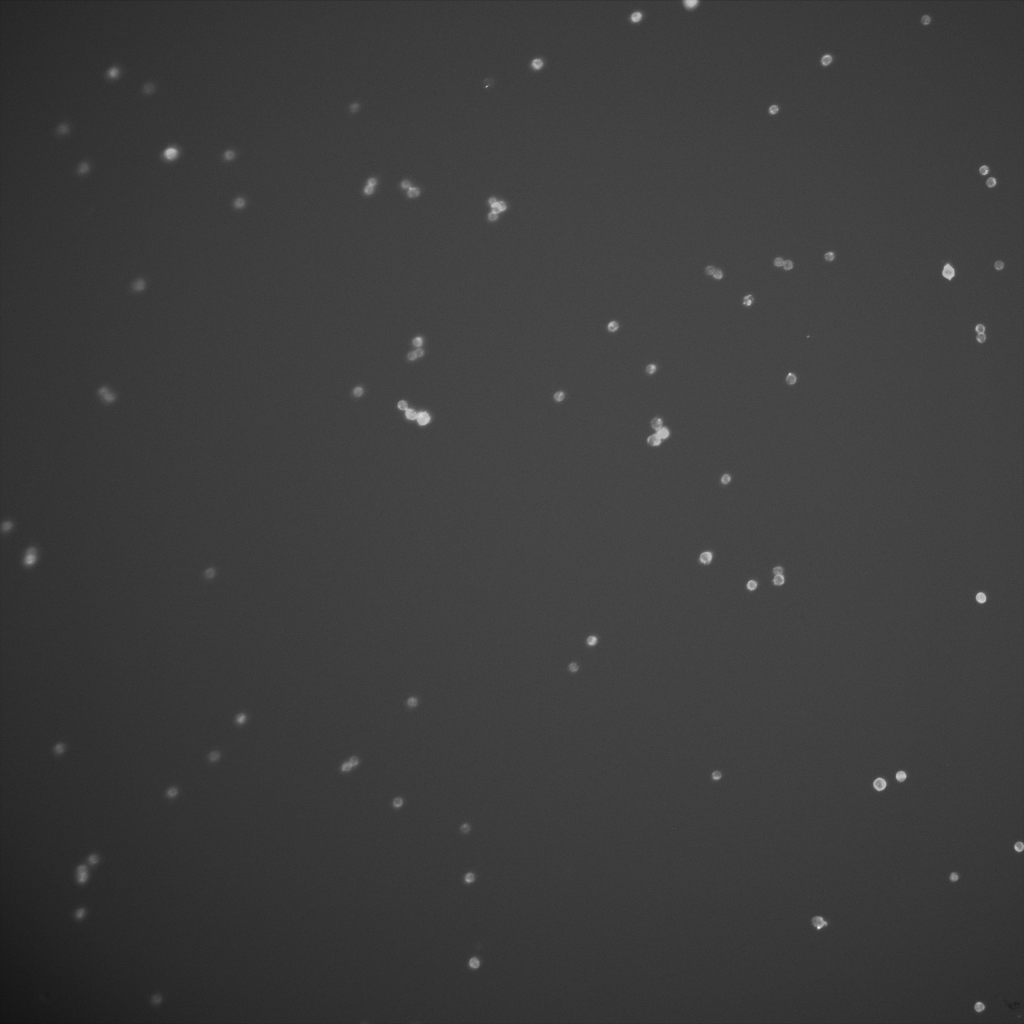

Supplement: Supplementary file 18 [file msb0011-0783-sd18.zip › Snap-20_c4_ORG.png]

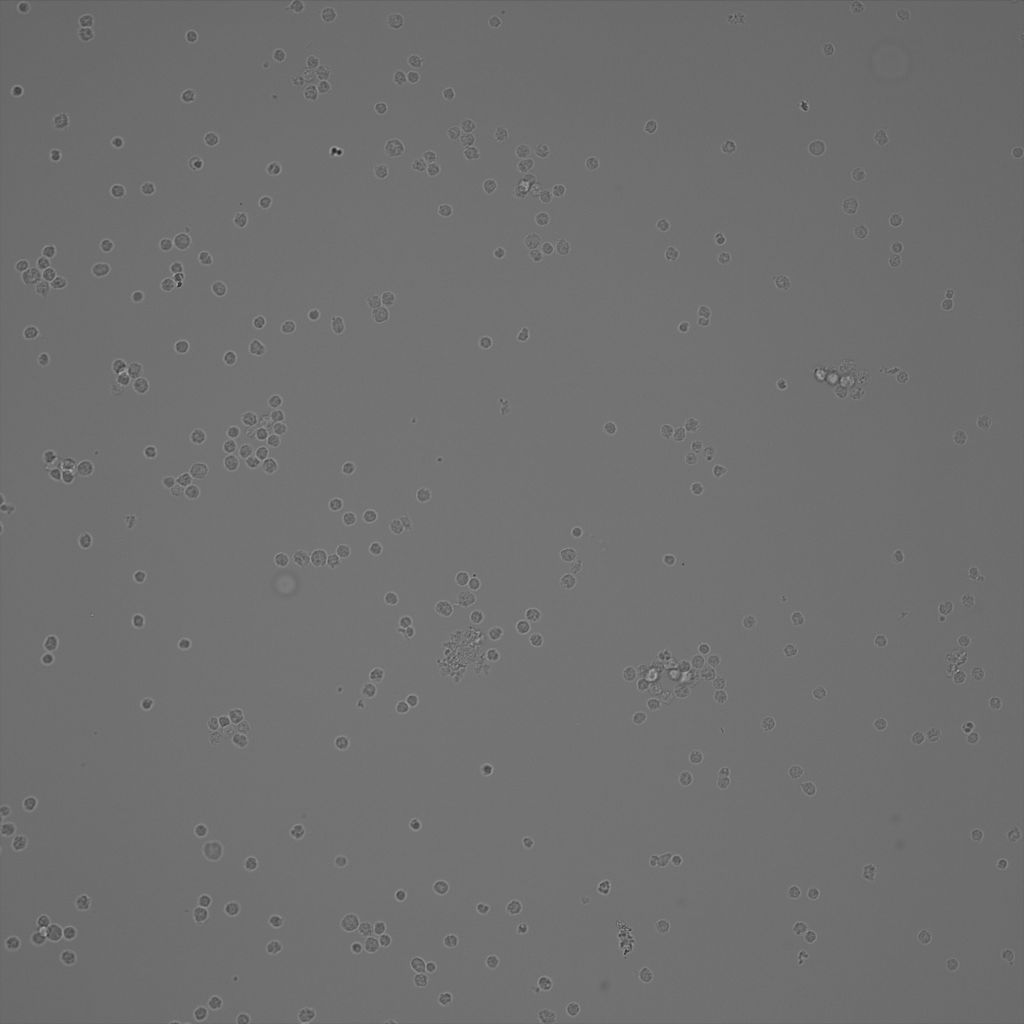

Supplement: Supplementary file 18 [file msb0011-0783-sd18.zip › Snap-21_c1_ORG.png]

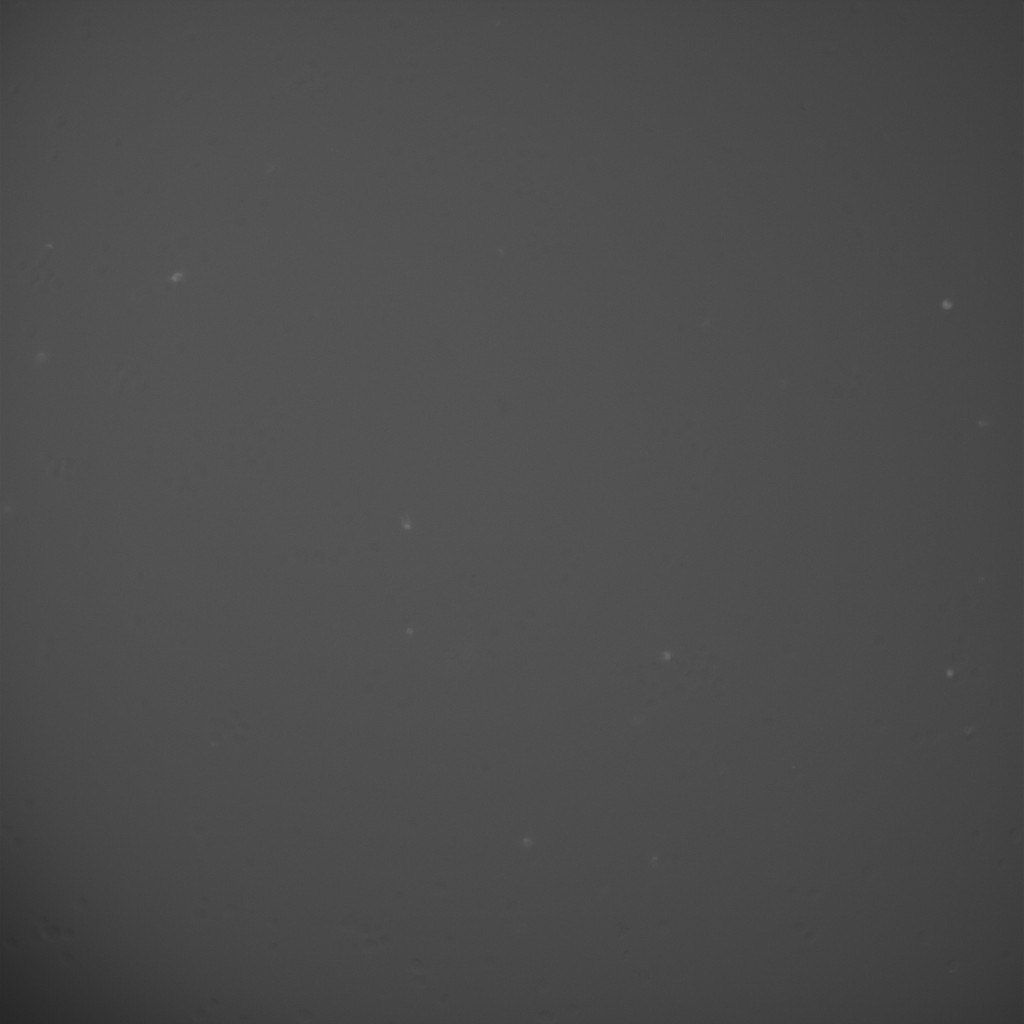

Supplement: Supplementary file 18 [file msb0011-0783-sd18.zip › Snap-21_c2_ORG.png]

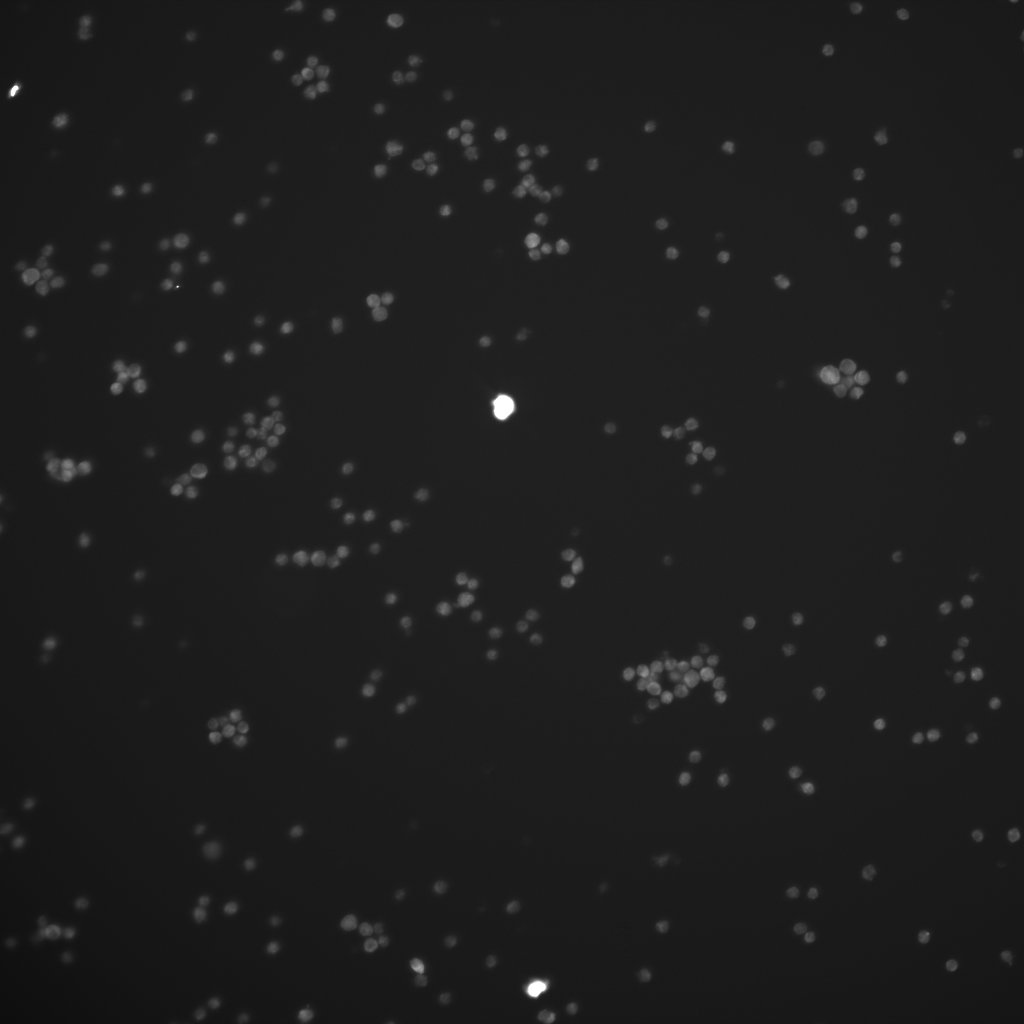

Supplement: Supplementary file 18 [file msb0011-0783-sd18.zip › Snap-21_c3_ORG.png]

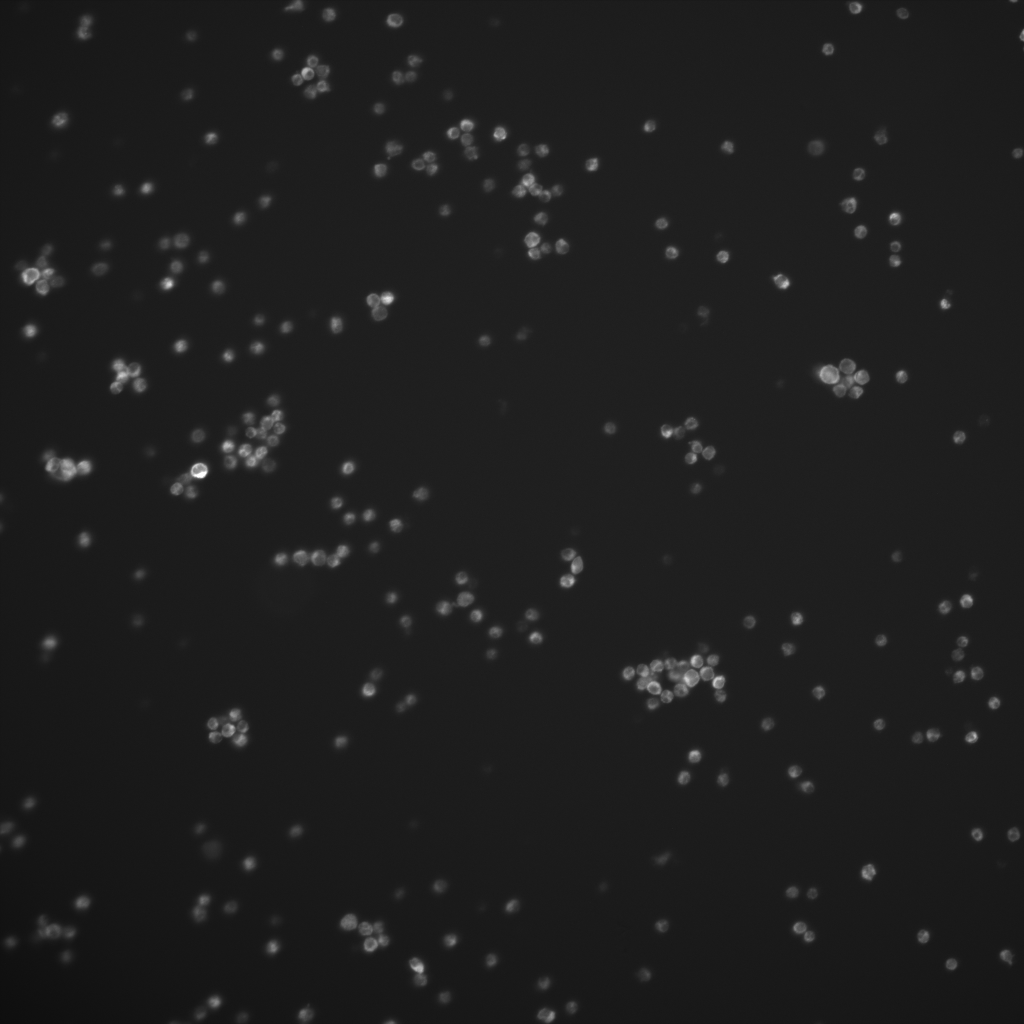

Supplement: Supplementary file 18 [file msb0011-0783-sd18.zip › Snap-21_c4_ORG.png]

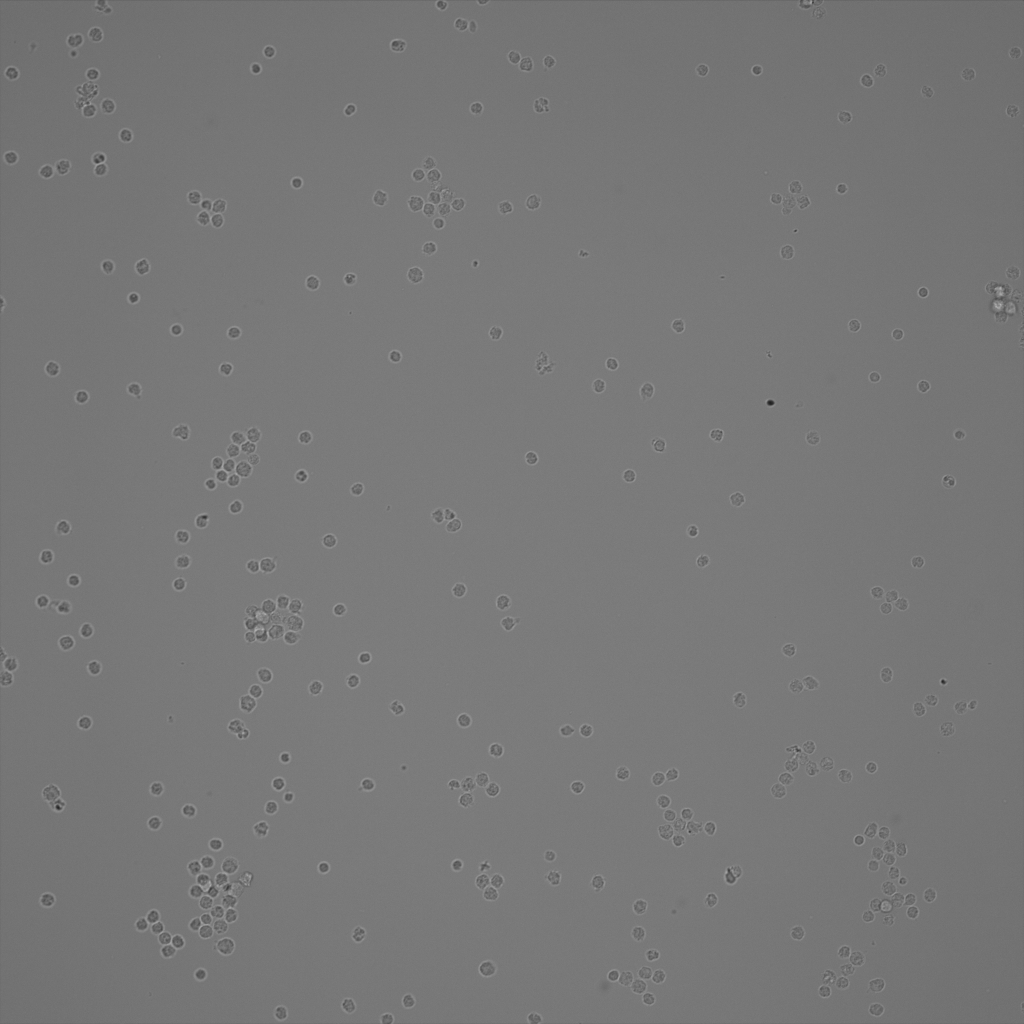

Supplement: Supplementary file 18 [file msb0011-0783-sd18.zip › Snap-22_c1_ORG.png]

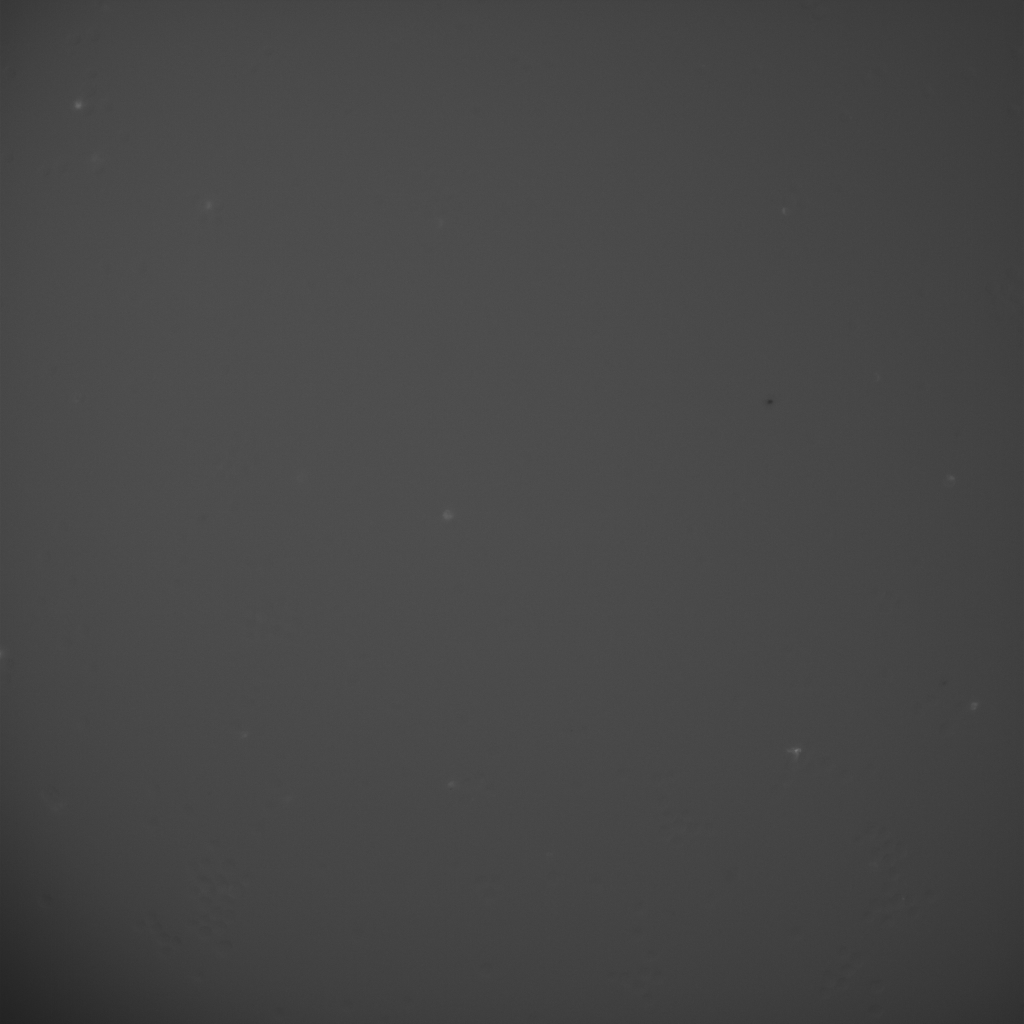

Supplement: Supplementary file 18 [file msb0011-0783-sd18.zip › Snap-22_c2_ORG.png]

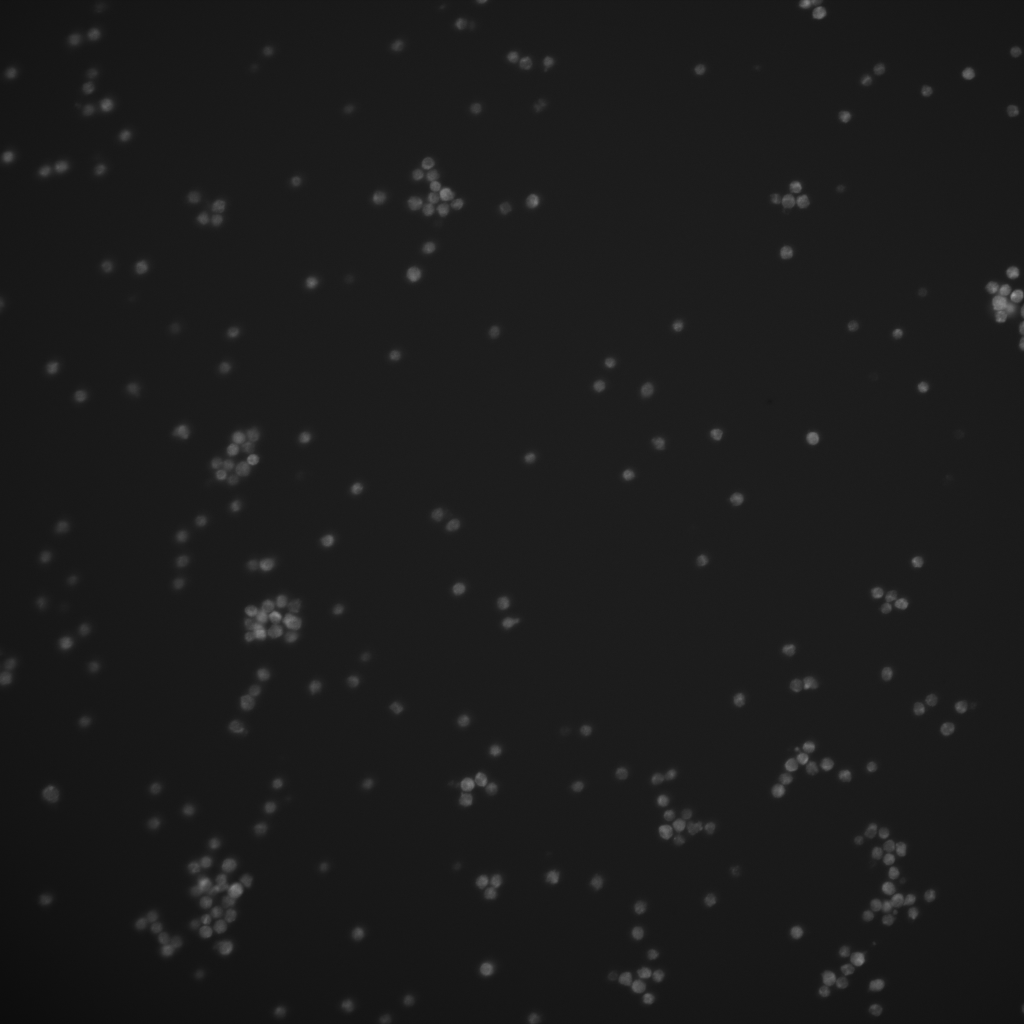

Supplement: Supplementary file 18 [file msb0011-0783-sd18.zip › Snap-22_c3_ORG.png]

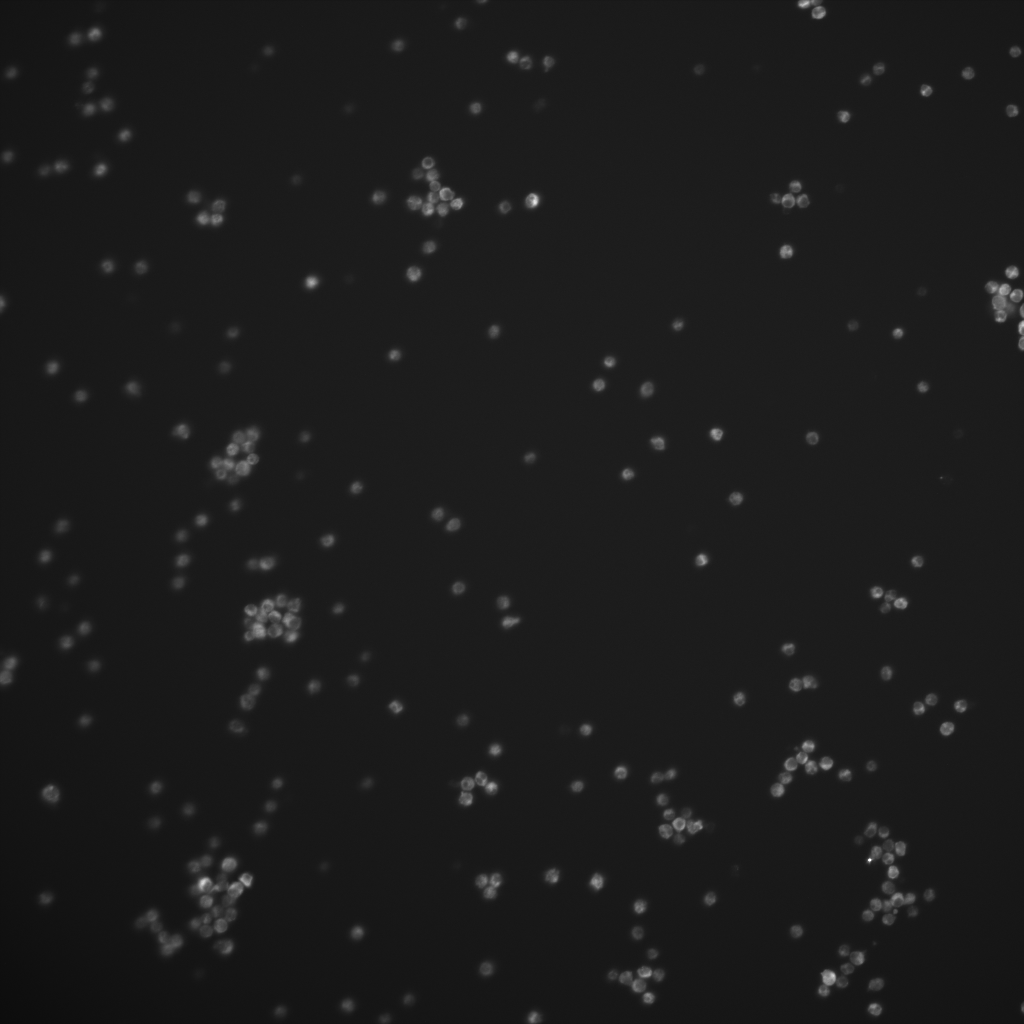

Supplement: Supplementary file 18 [file msb0011-0783-sd18.zip › Snap-22_c4_ORG.png]

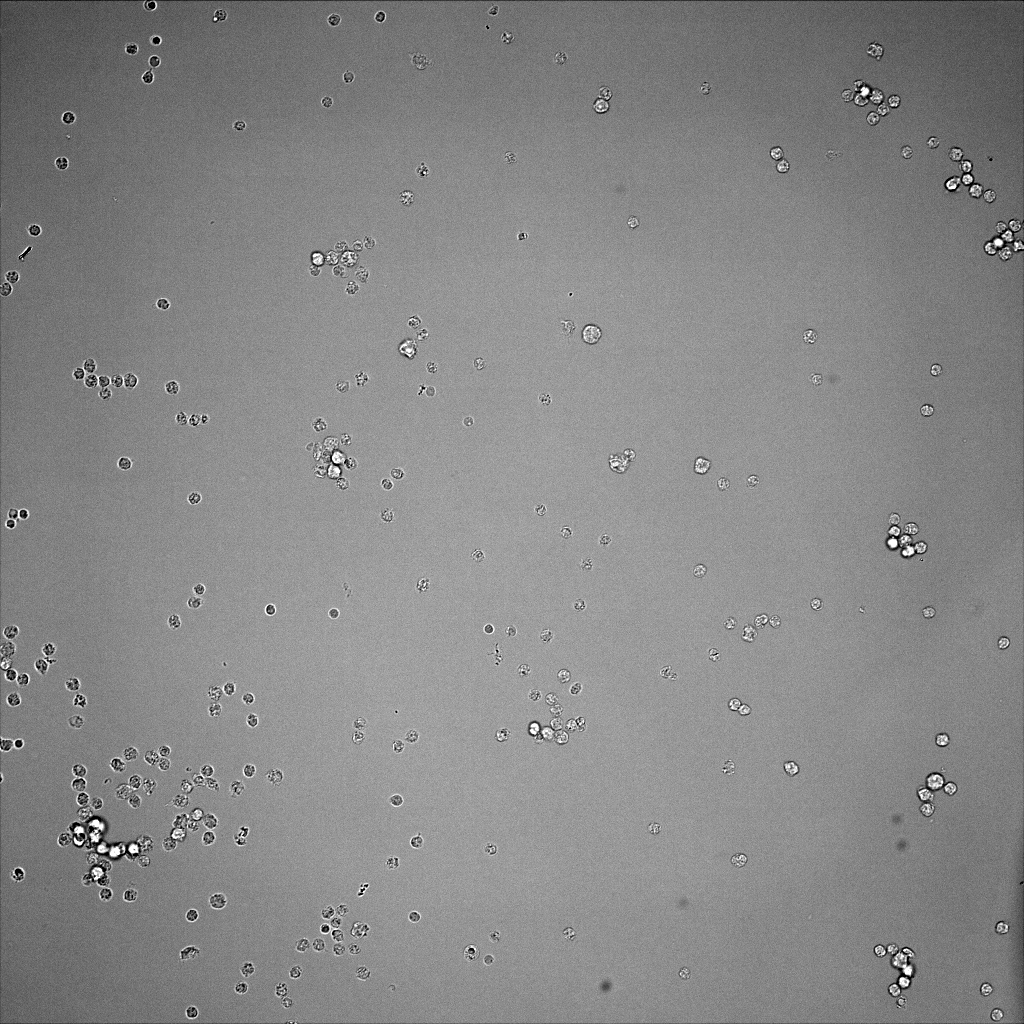

Supplement: Supplementary file 18 [file msb0011-0783-sd18.zip › Snap-23_c1_ORG.png]

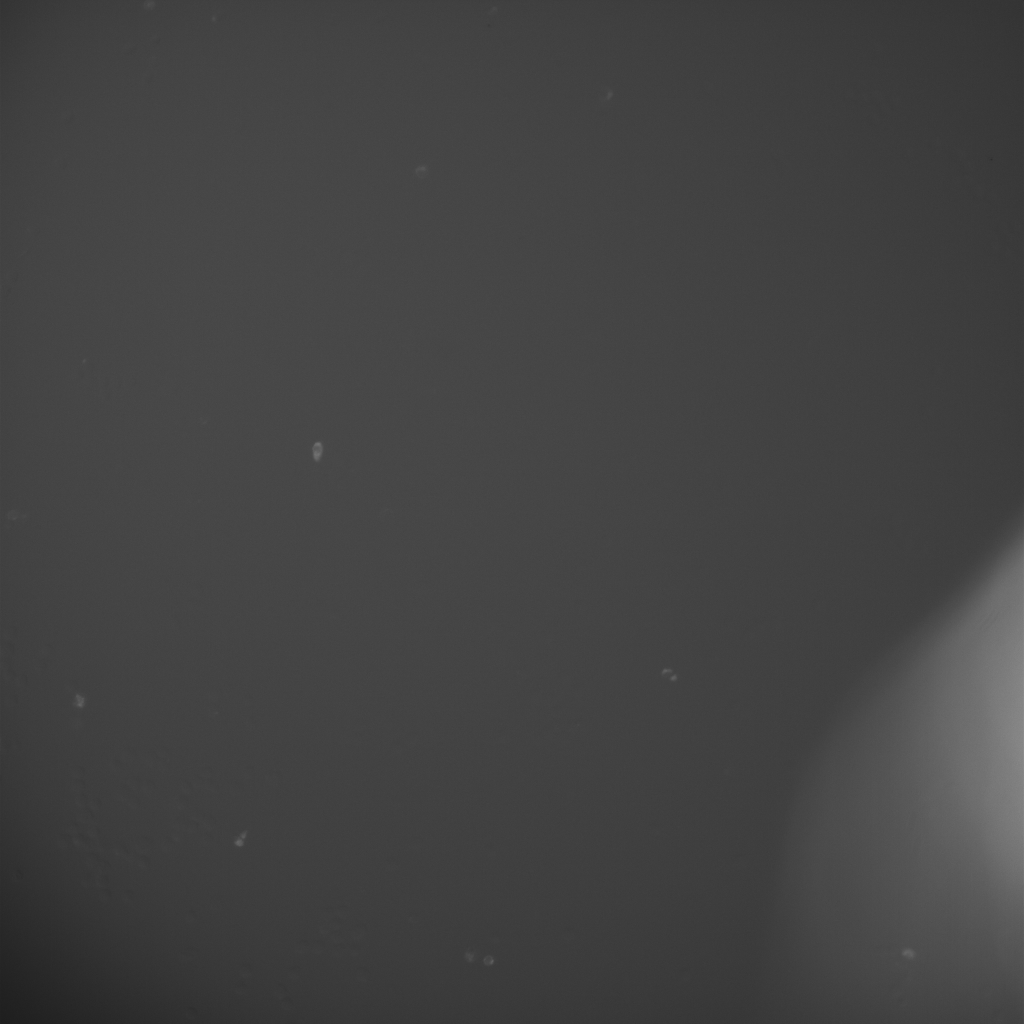

Supplement: Supplementary file 18 [file msb0011-0783-sd18.zip › Snap-23_c2_ORG.png]

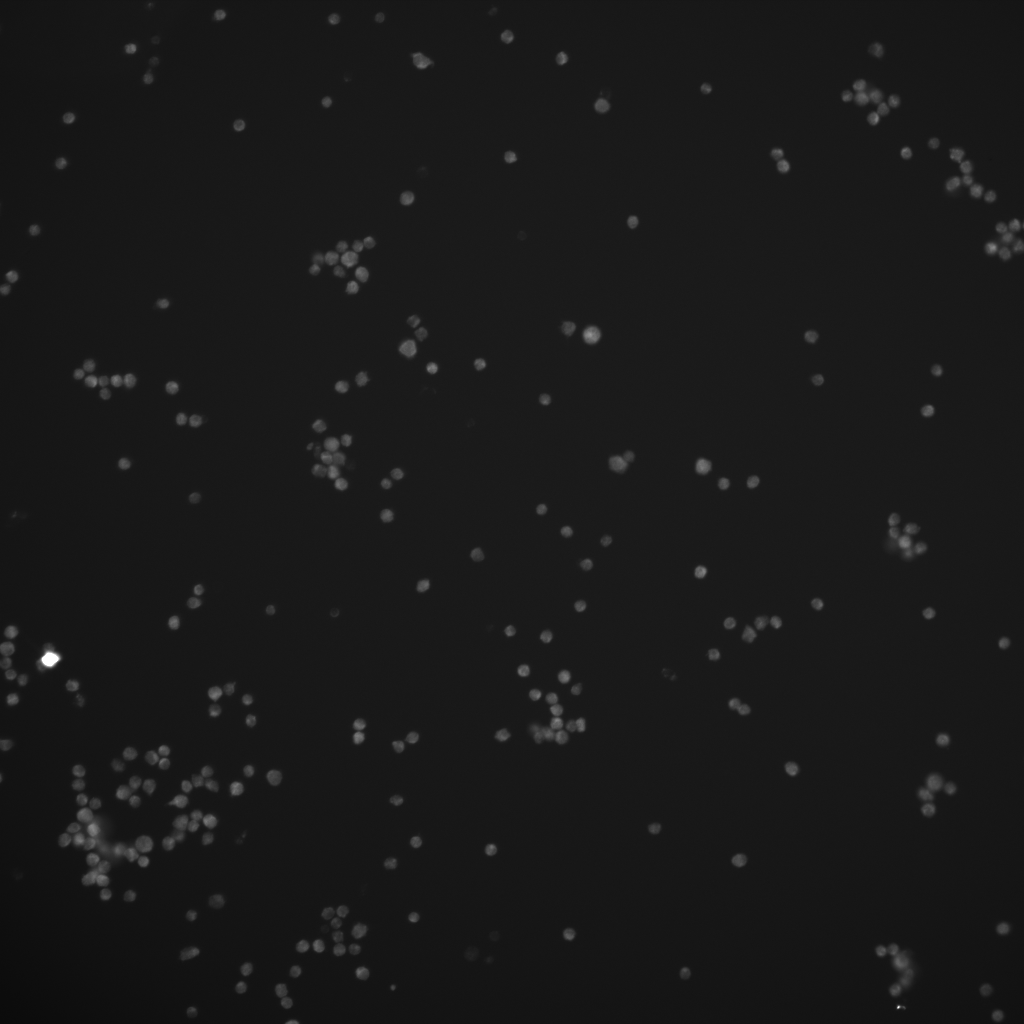

Supplement: Supplementary file 18 [file msb0011-0783-sd18.zip › Snap-23_c3_ORG.png]

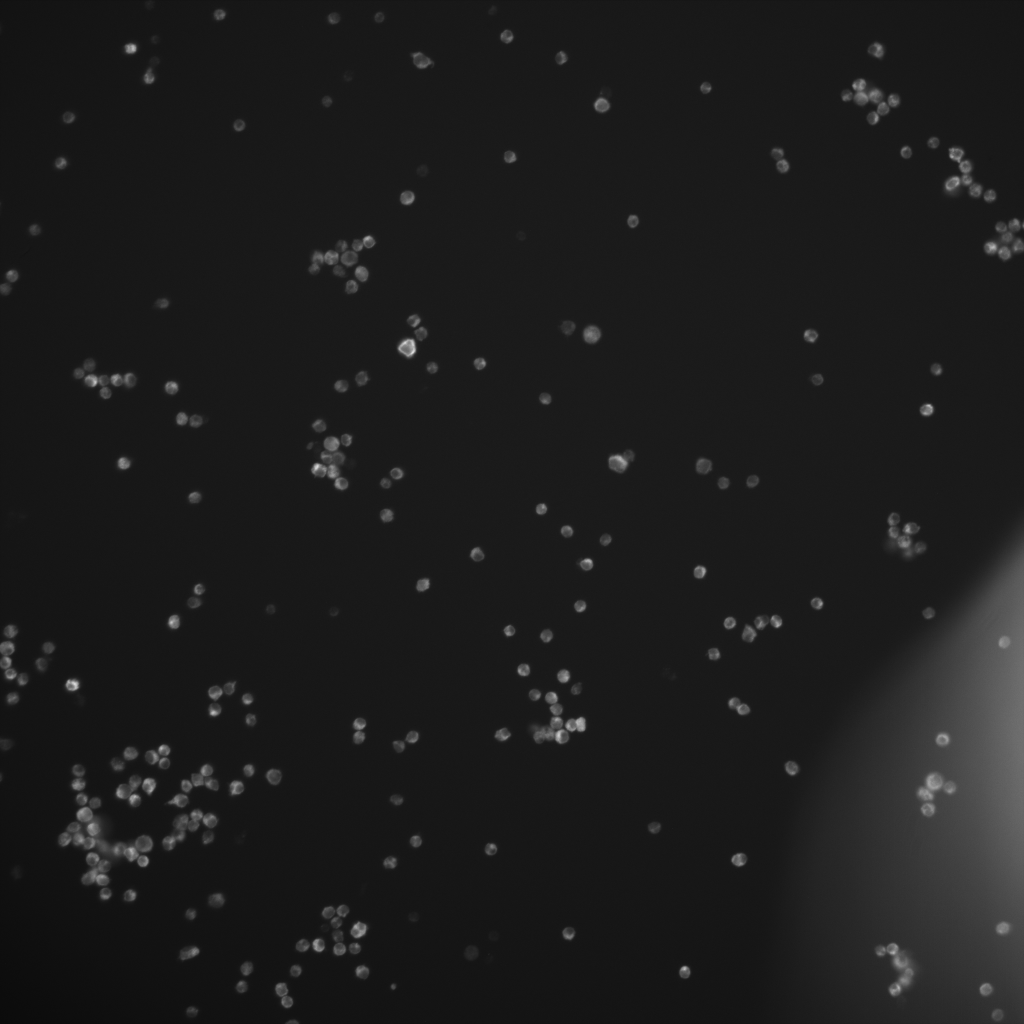

Supplement: Supplementary file 18 [file msb0011-0783-sd18.zip › Snap-23_c4_ORG.png]

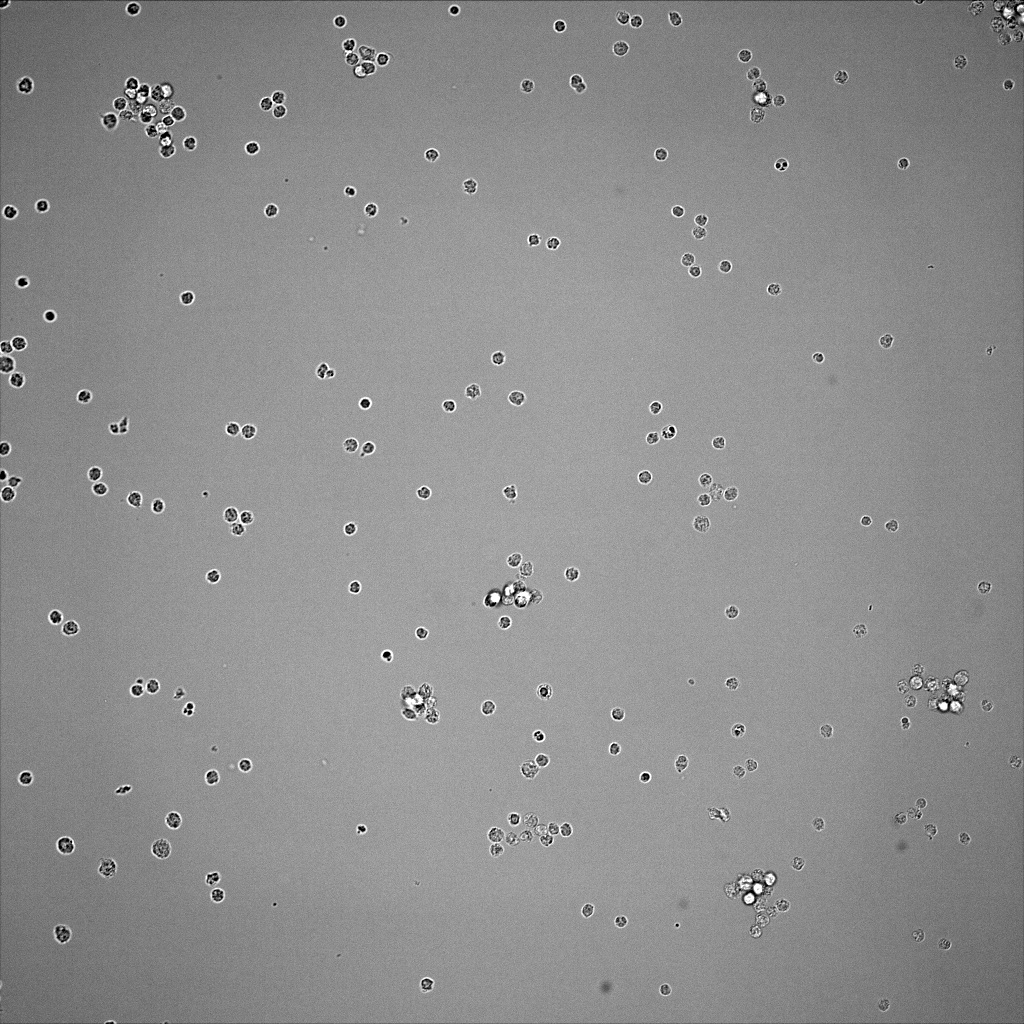

Supplement: Supplementary file 18 [file msb0011-0783-sd18.zip › Snap-24_c1_ORG.png]

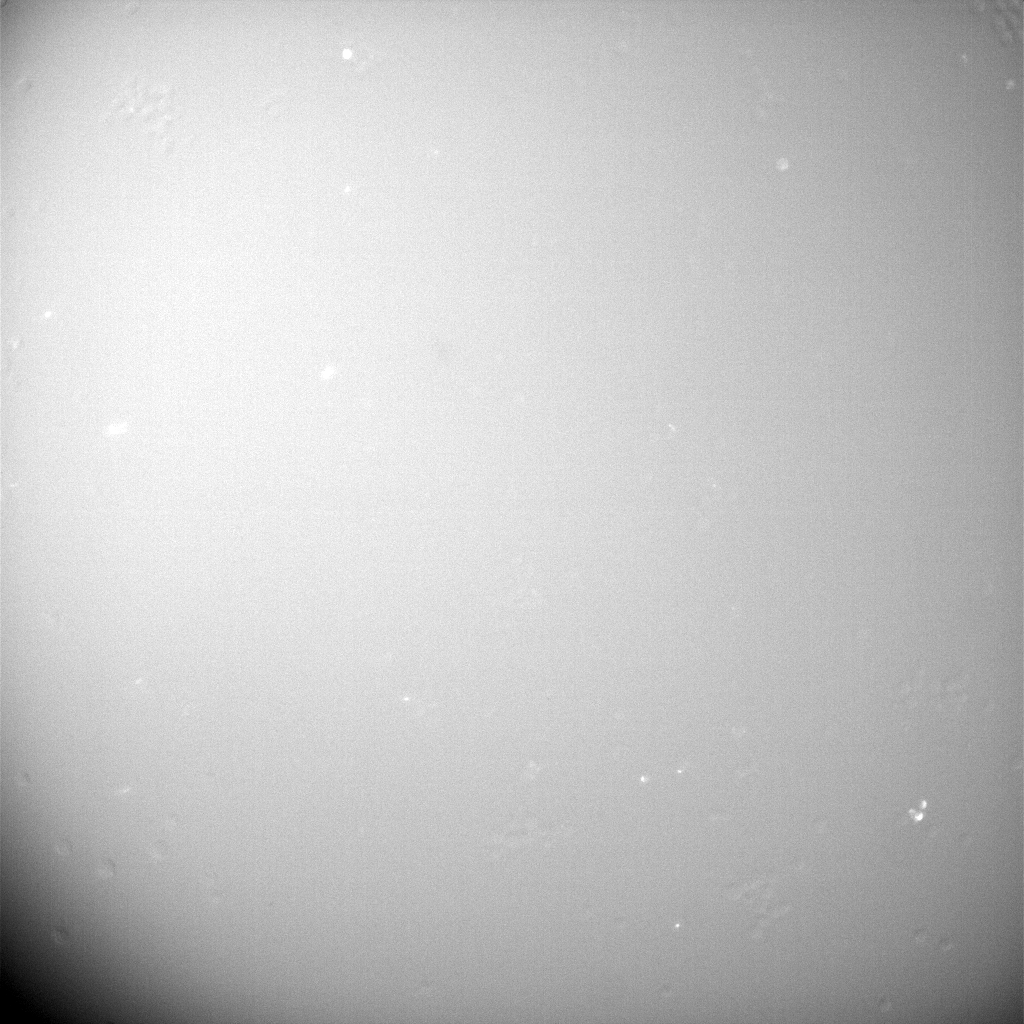

Supplement: Supplementary file 18 [file msb0011-0783-sd18.zip › Snap-24_c2_ORG.png]

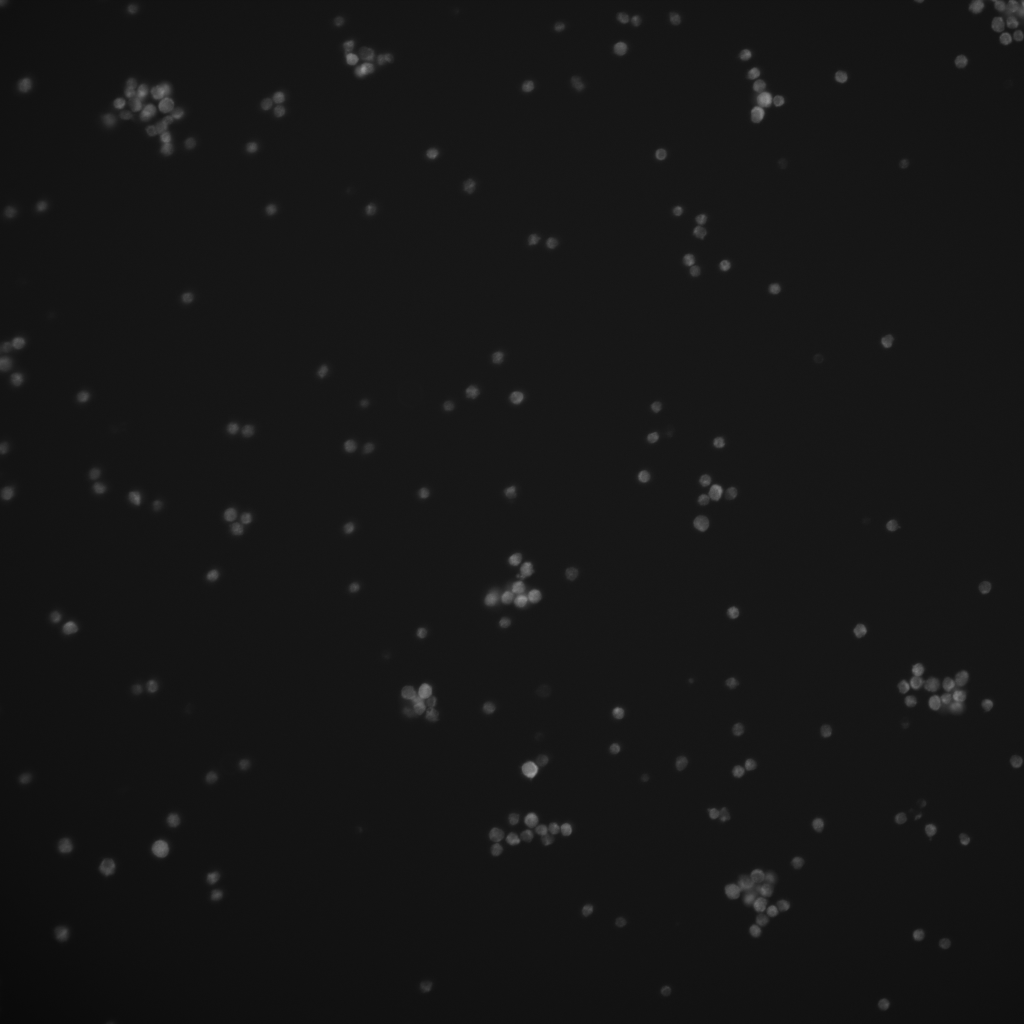

Supplement: Supplementary file 18 [file msb0011-0783-sd18.zip › Snap-24_c3_ORG.png]

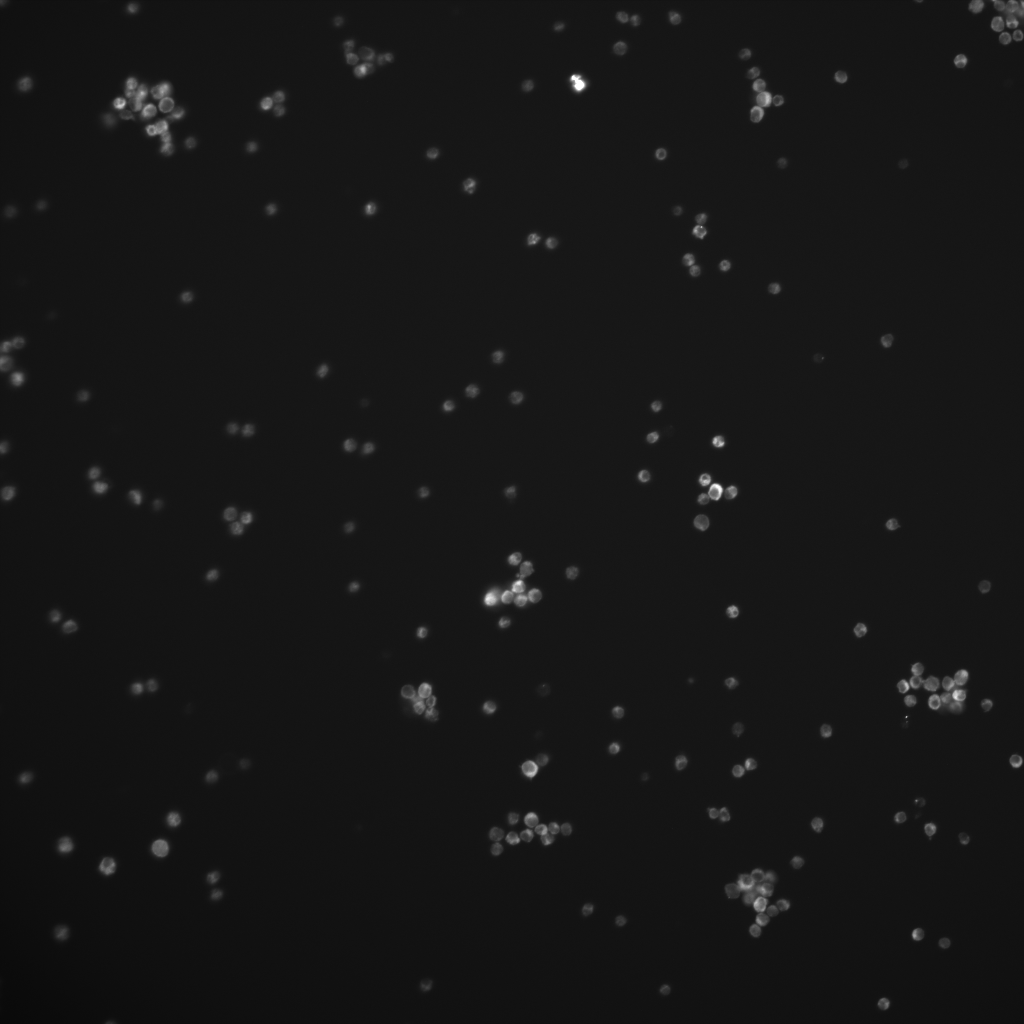

Supplement: Supplementary file 18 [file msb0011-0783-sd18.zip › Snap-24_c4_ORG.png]

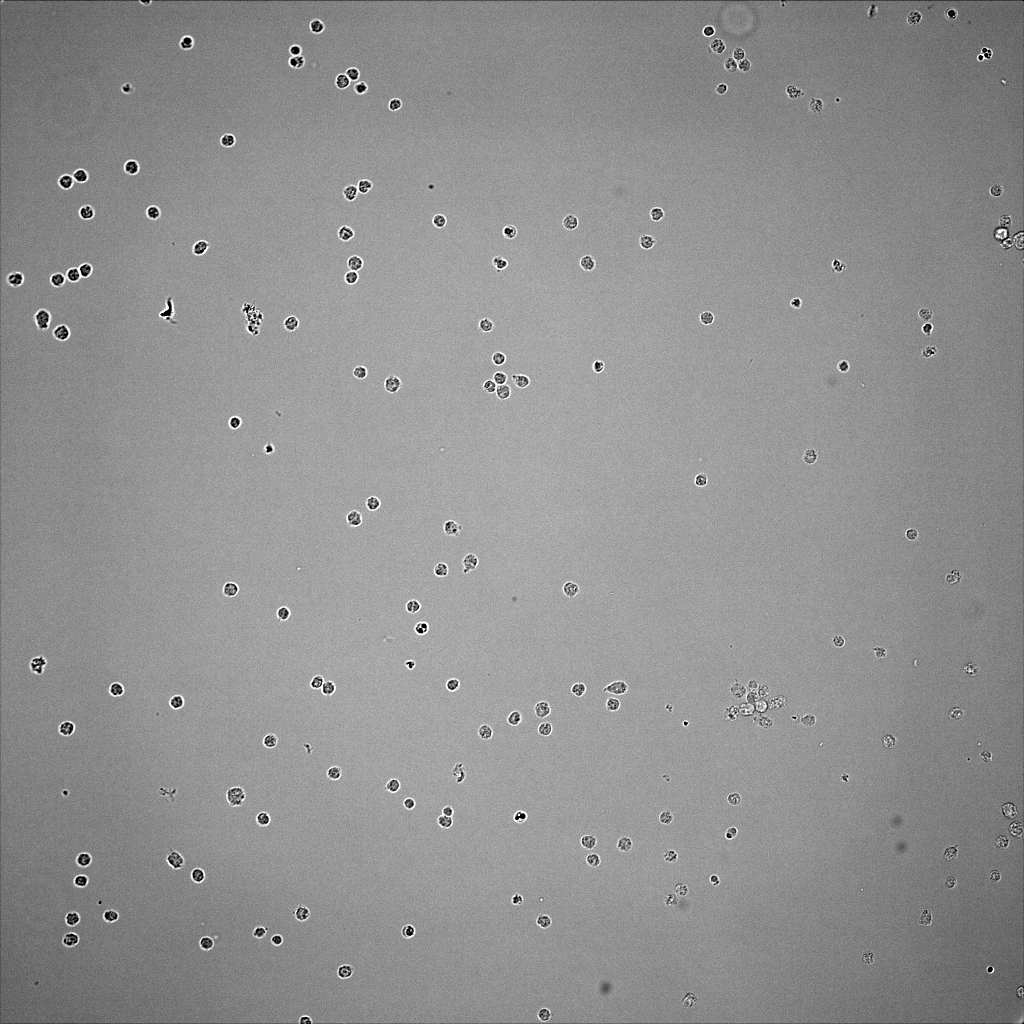

Supplement: Supplementary file 18 [file msb0011-0783-sd18.zip › Snap-25_c1_ORG.png]

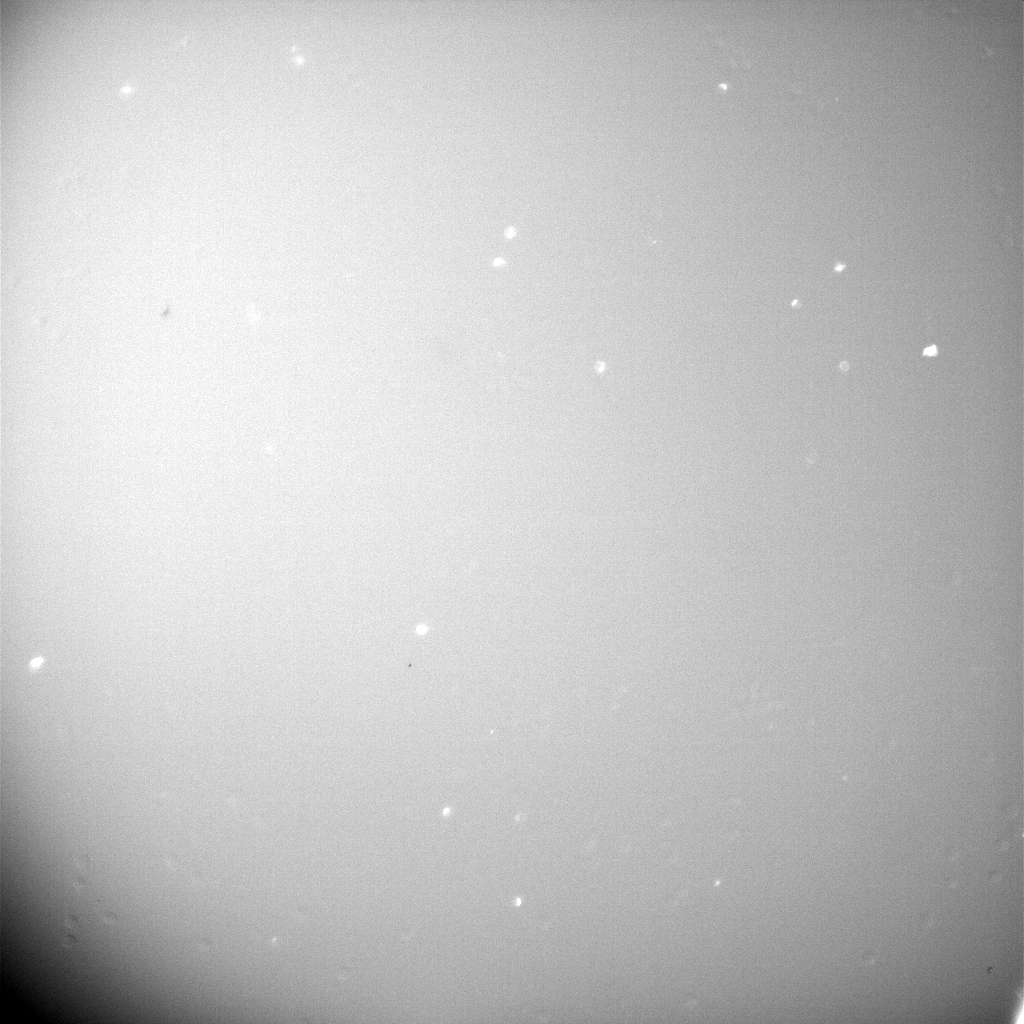

Supplement: Supplementary file 18 [file msb0011-0783-sd18.zip › Snap-25_c2_ORG.png]

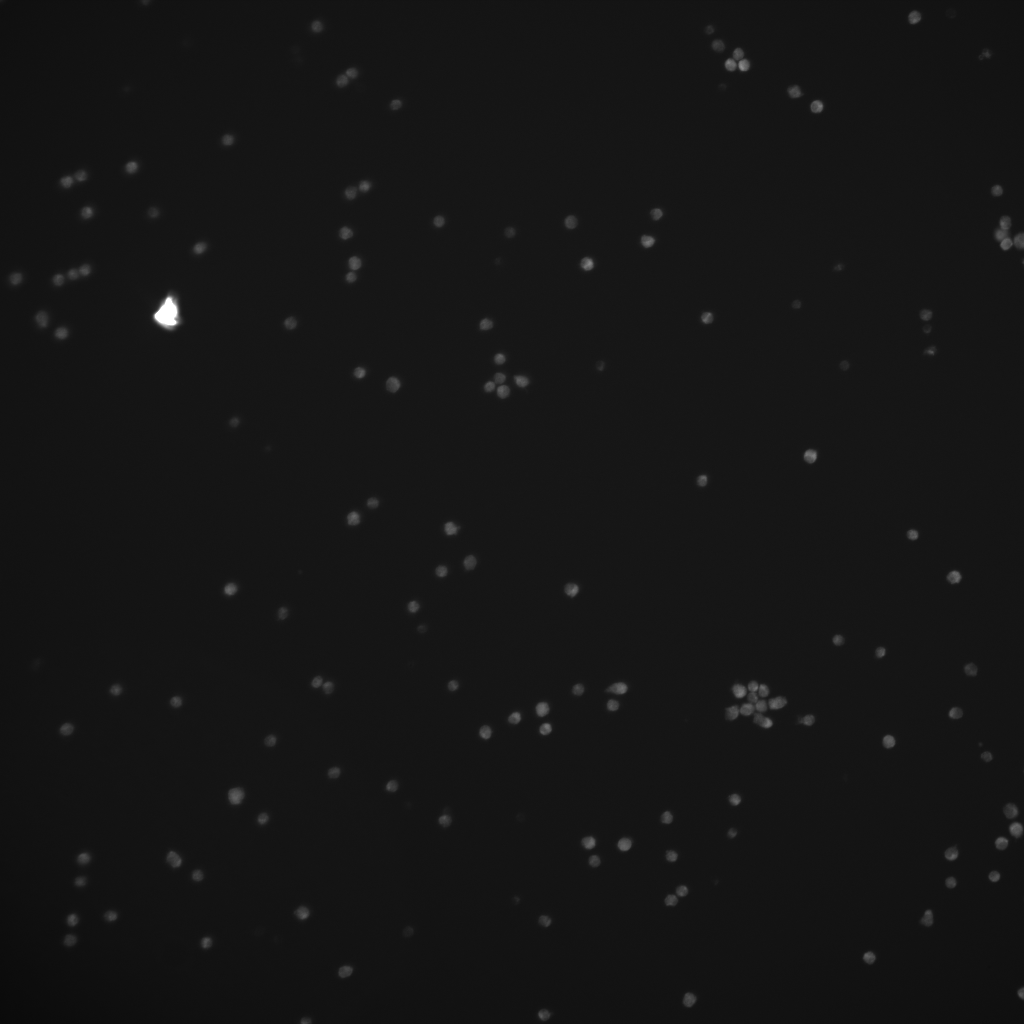

Supplement: Supplementary file 18 [file msb0011-0783-sd18.zip › Snap-25_c3_ORG.png]

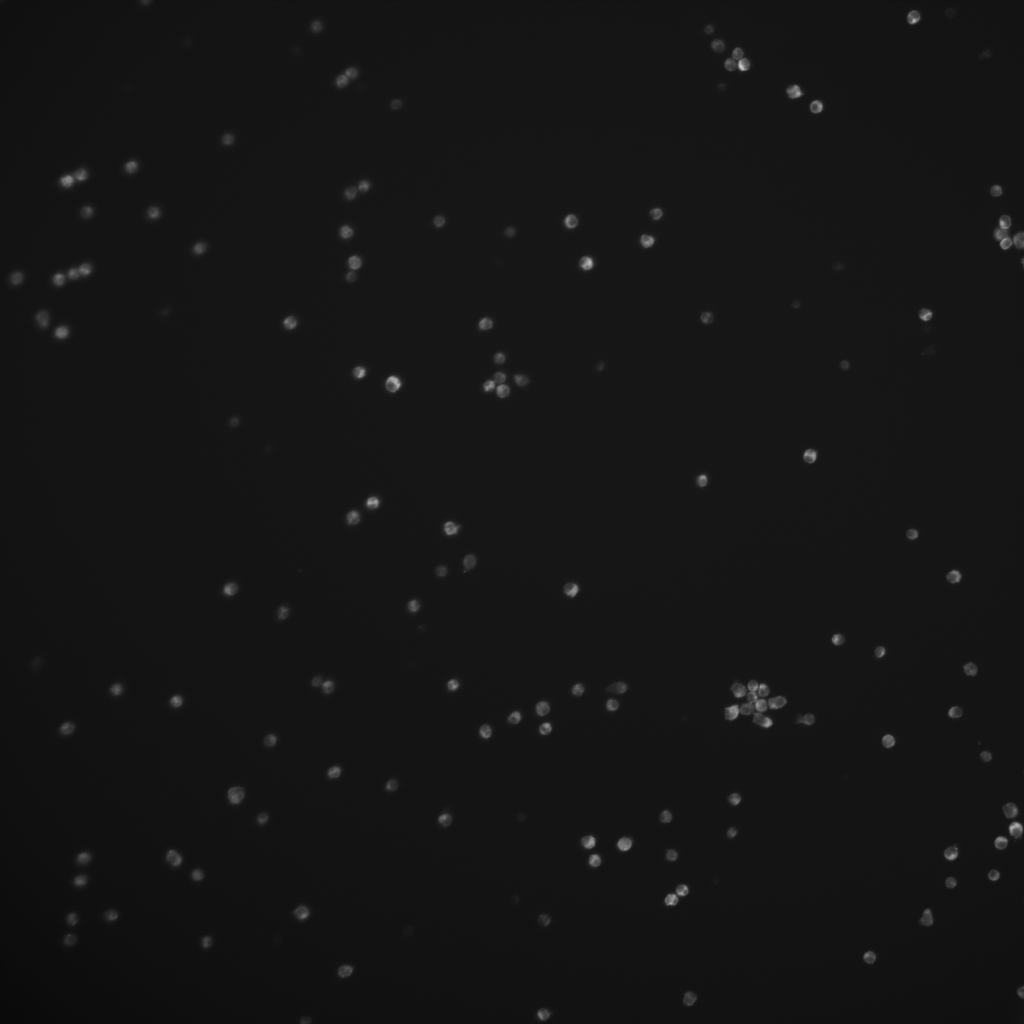

Supplement: Supplementary file 18 [file msb0011-0783-sd18.zip › Snap-25_c4_ORG.png]

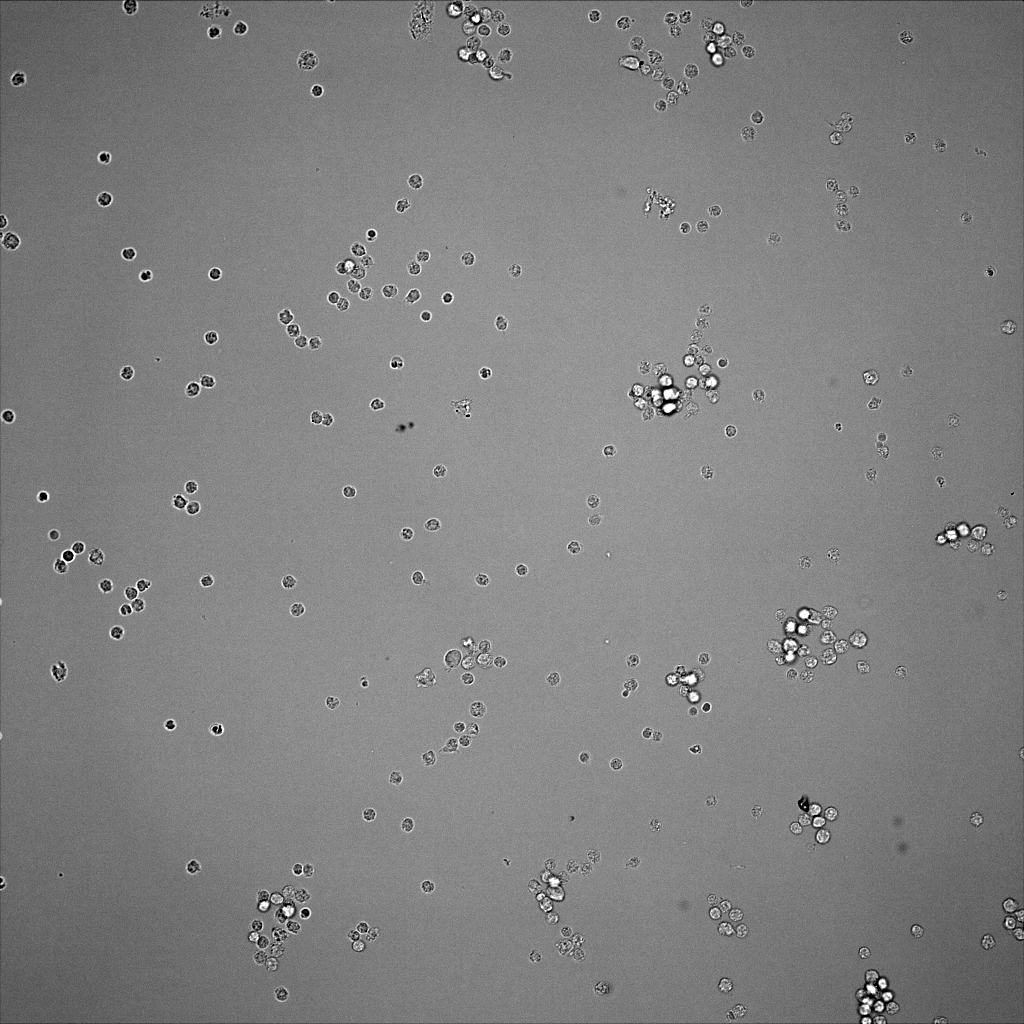

Supplement: Supplementary file 18 [file msb0011-0783-sd18.zip › Snap-26_c1_ORG.png]

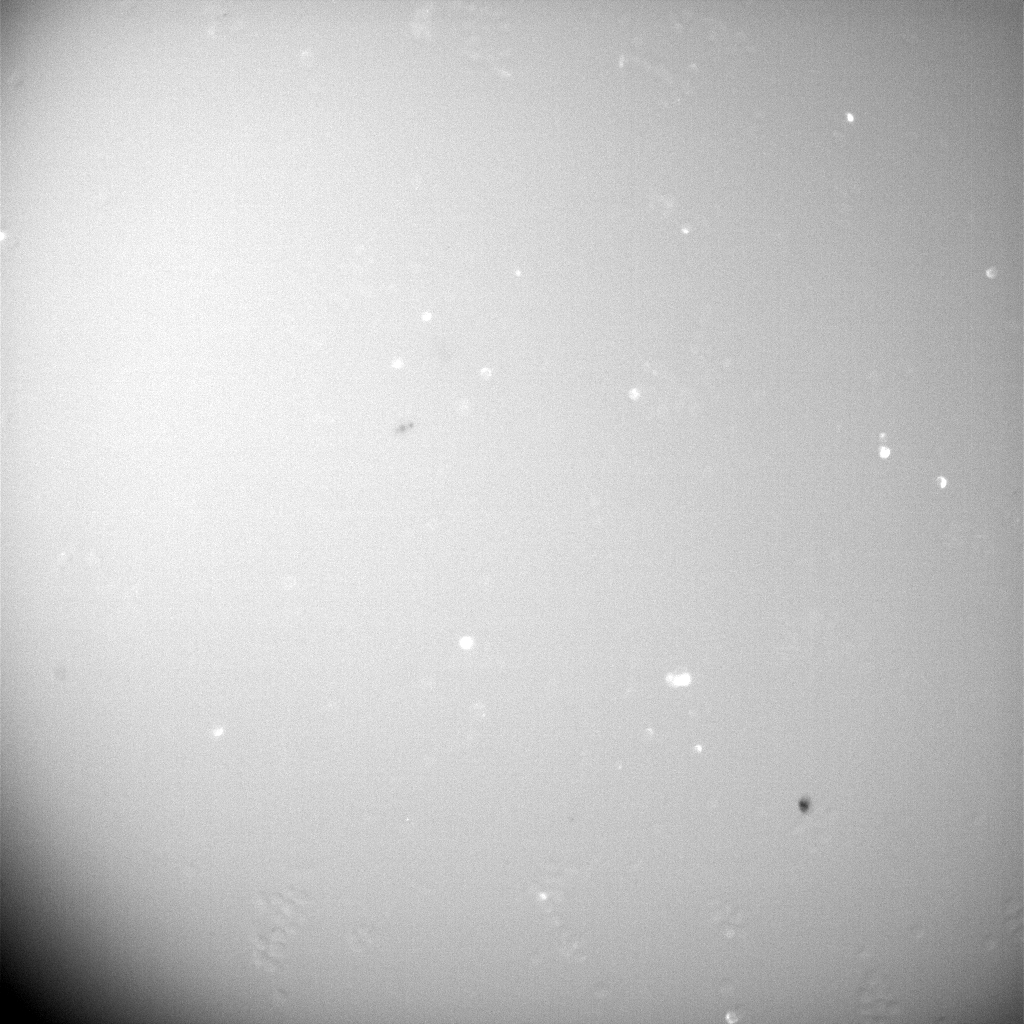

Supplement: Supplementary file 18 [file msb0011-0783-sd18.zip › Snap-26_c2_ORG.png]

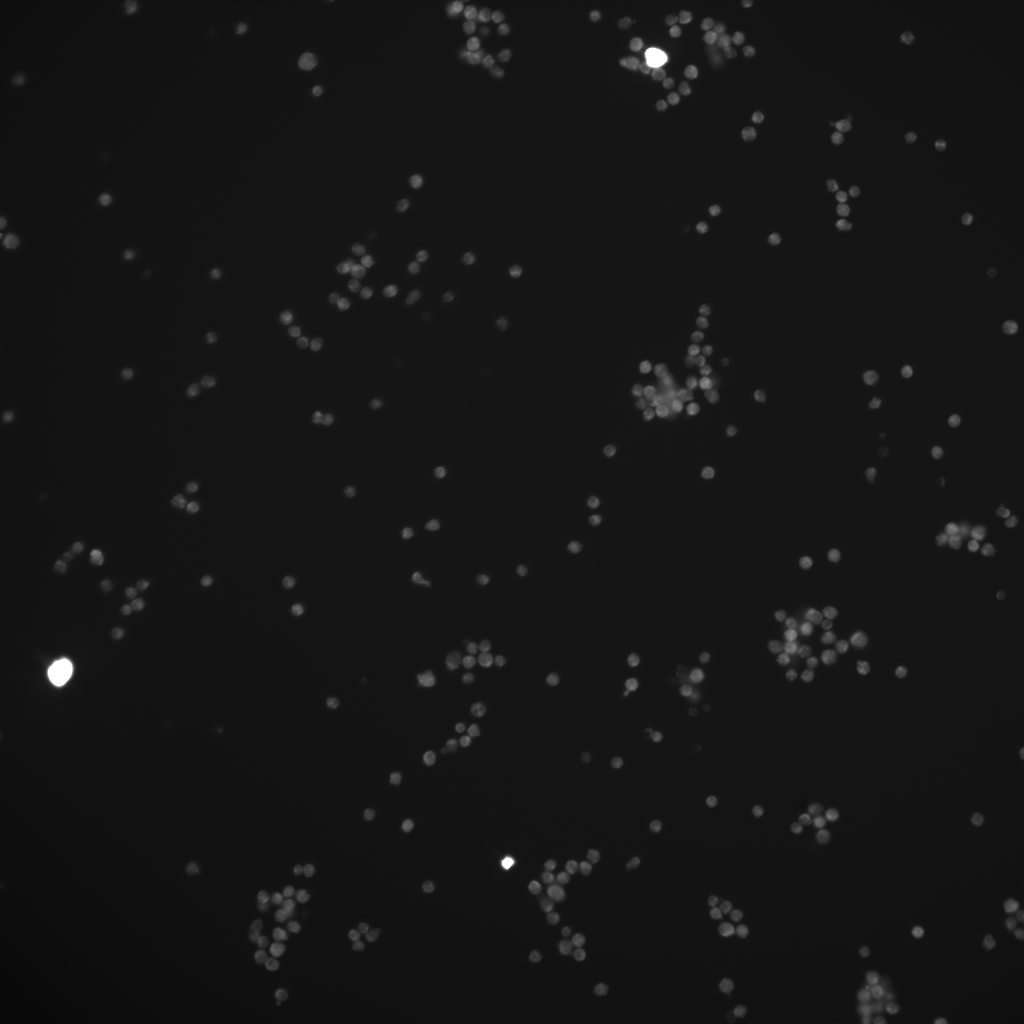

Supplement: Supplementary file 18 [file msb0011-0783-sd18.zip › Snap-26_c3_ORG.png]

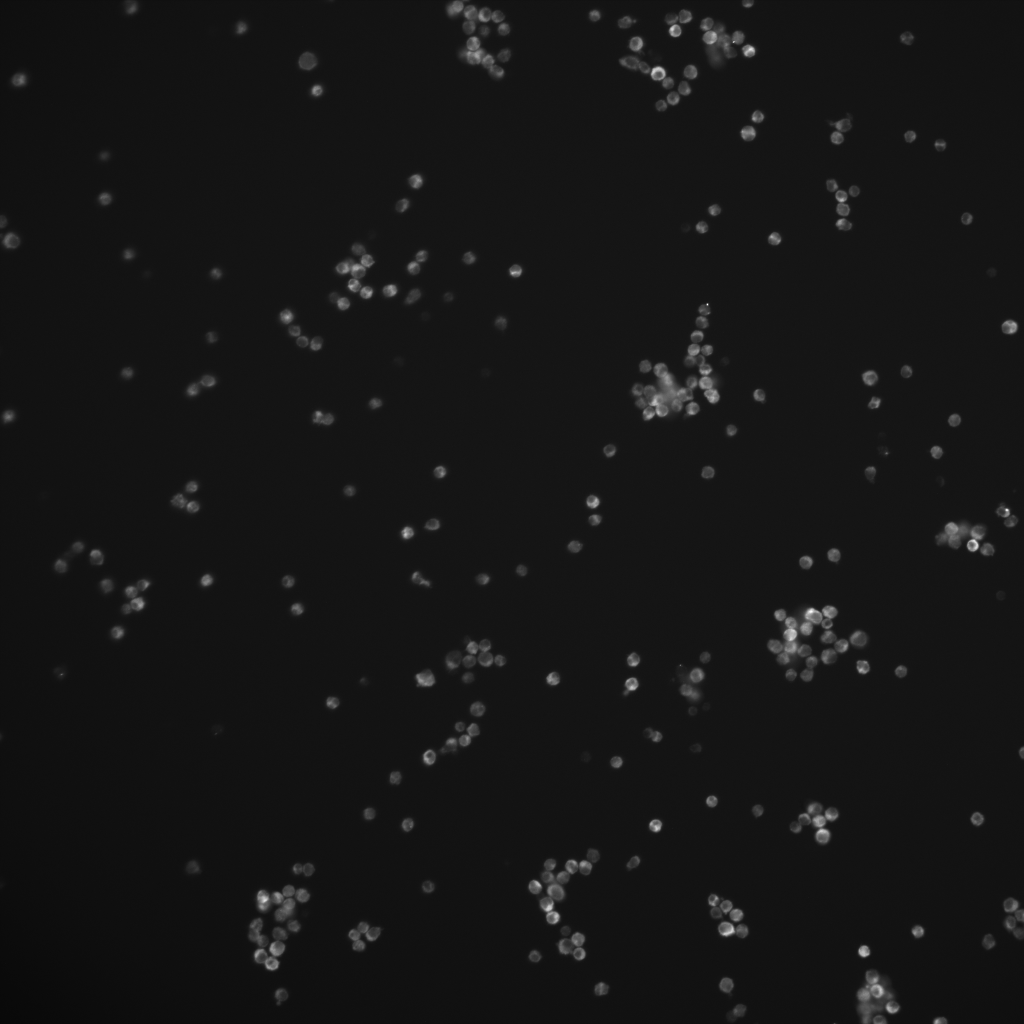

Supplement: Supplementary file 18 [file msb0011-0783-sd18.zip › Snap-26_c4_ORG.png]

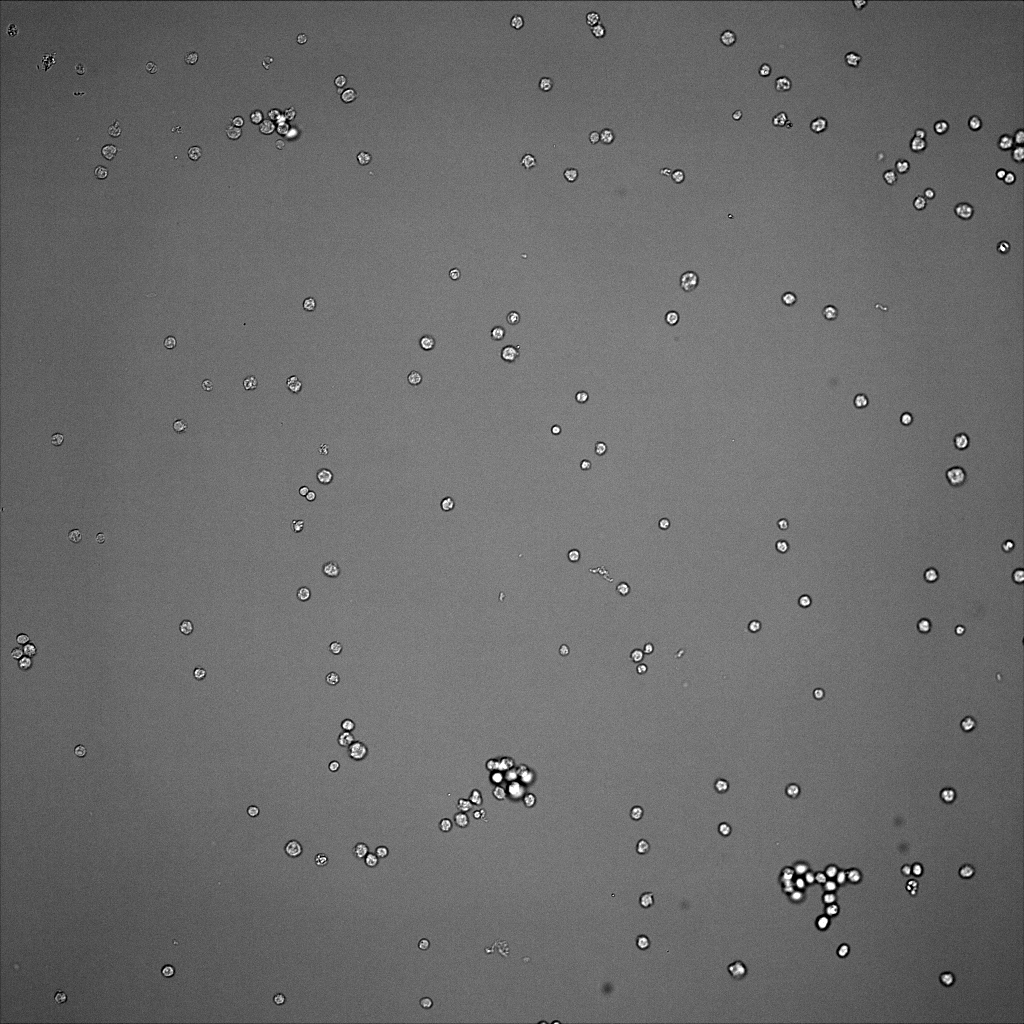

Supplement: Supplementary file 18 [file msb0011-0783-sd18.zip › Snap-27_c1_ORG.png]

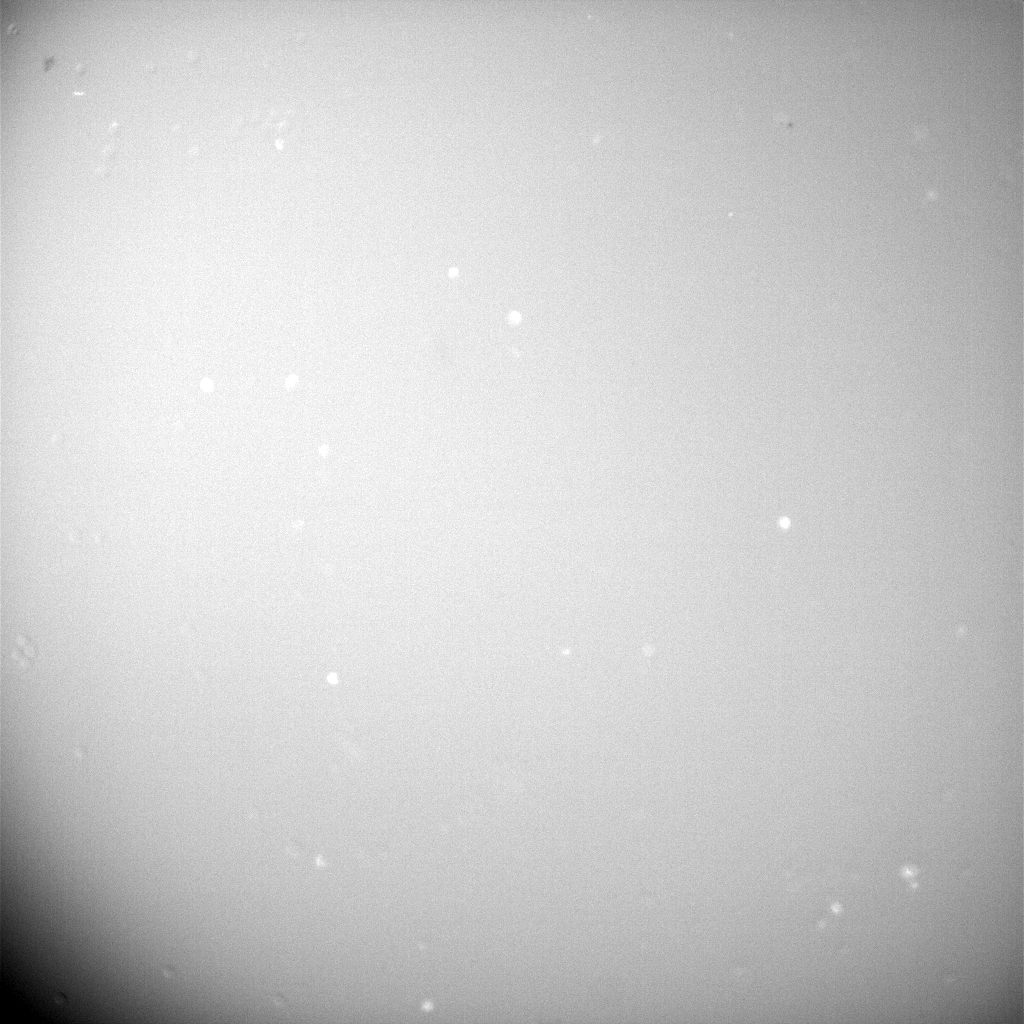

Supplement: Supplementary file 18 [file msb0011-0783-sd18.zip › Snap-27_c2_ORG.png]

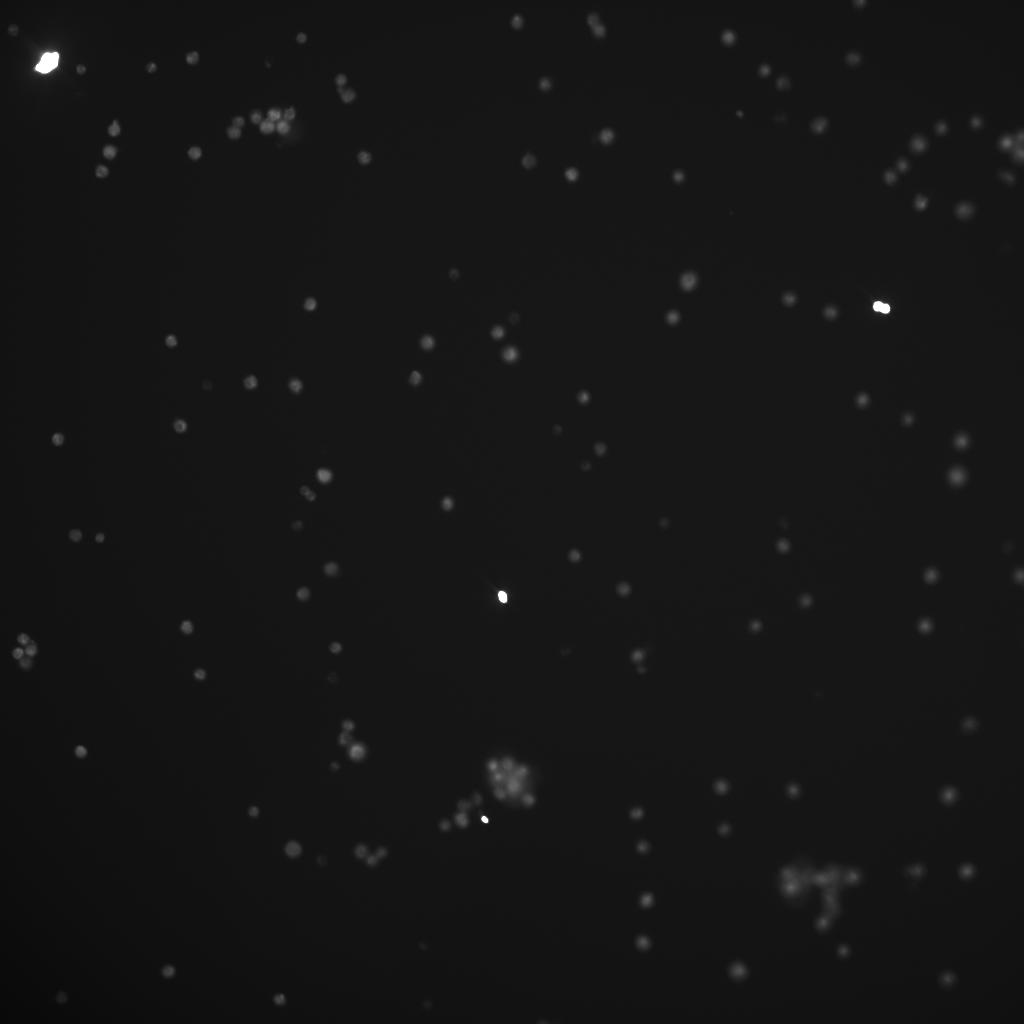

Supplement: Supplementary file 18 [file msb0011-0783-sd18.zip › Snap-27_c3_ORG.png]

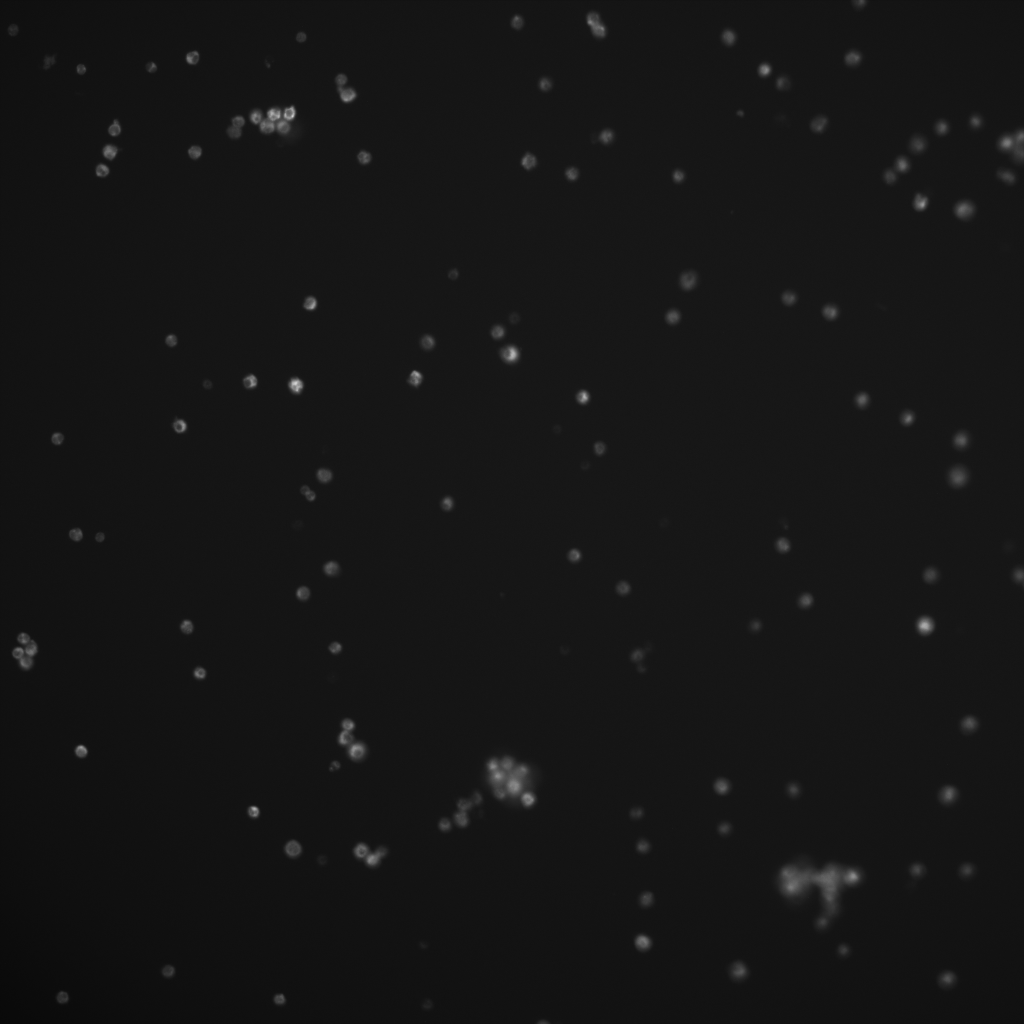

Supplement: Supplementary file 18 [file msb0011-0783-sd18.zip › Snap-27_c4_ORG.png]

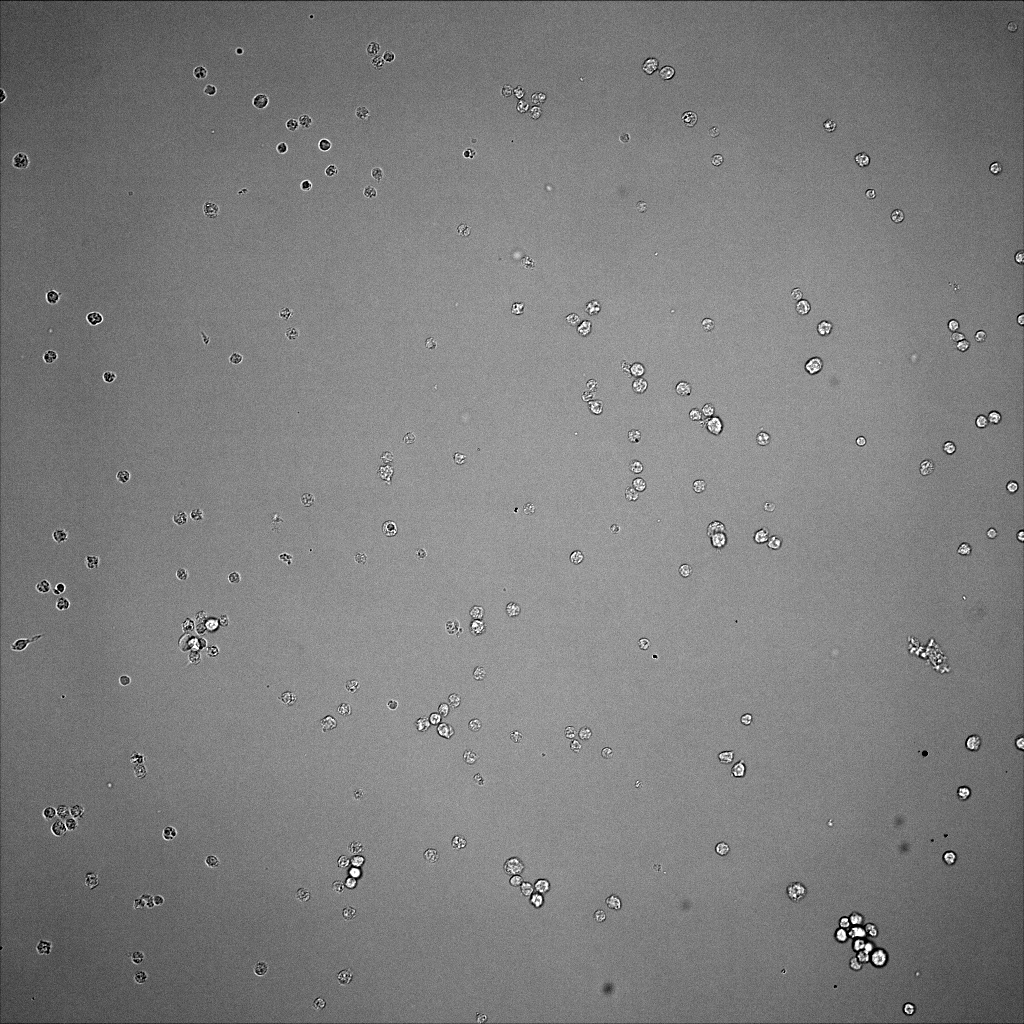

Supplement: Supplementary file 18 [file msb0011-0783-sd18.zip › Snap-28_c1_ORG.png]

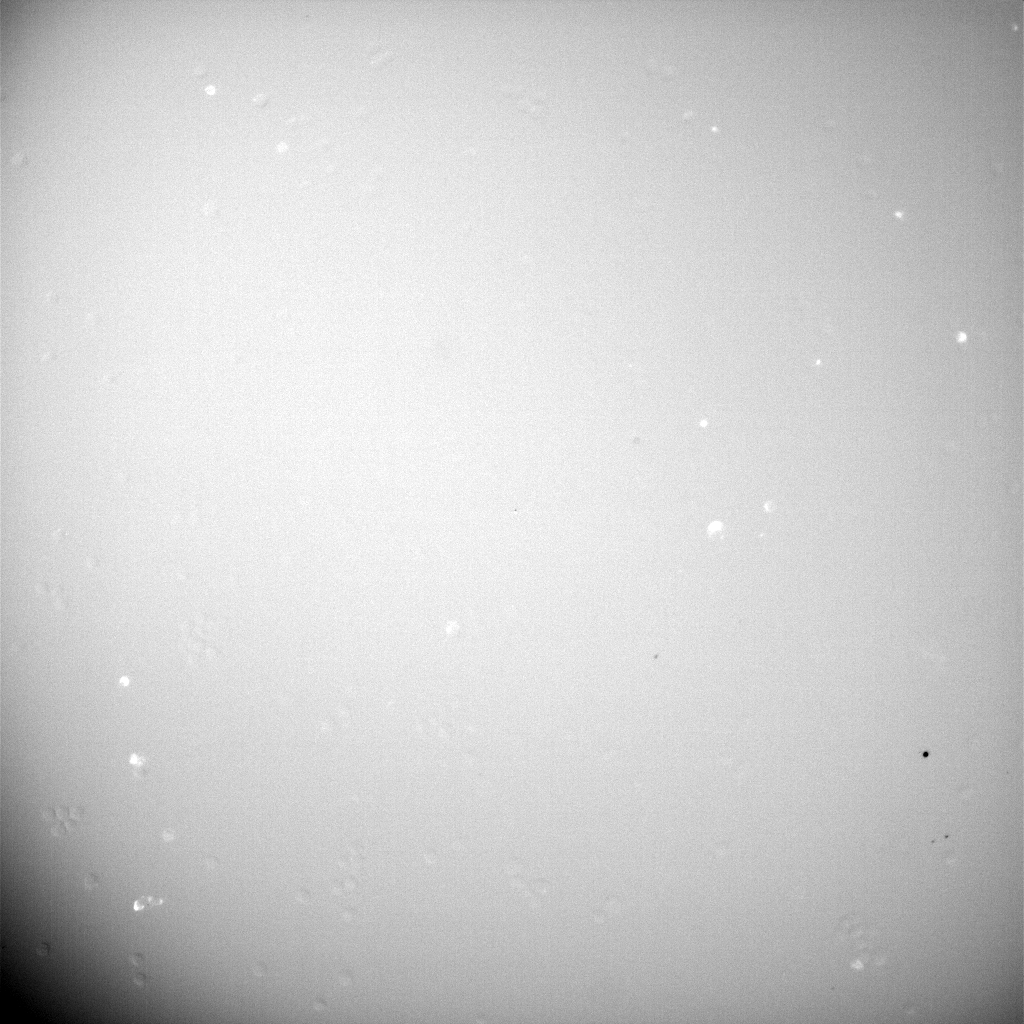

Supplement: Supplementary file 18 [file msb0011-0783-sd18.zip › Snap-28_c2_ORG.png]

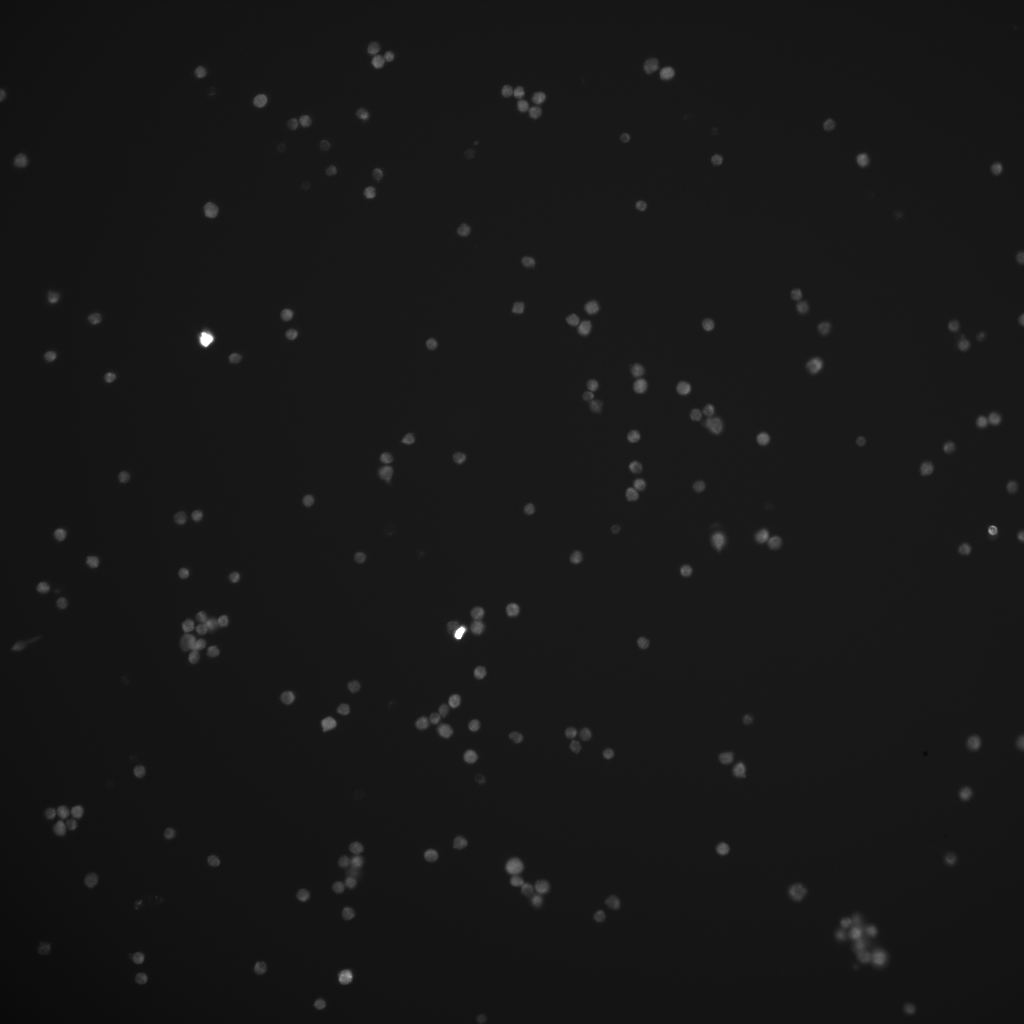

Supplement: Supplementary file 18 [file msb0011-0783-sd18.zip › Snap-28_c3_ORG.png]

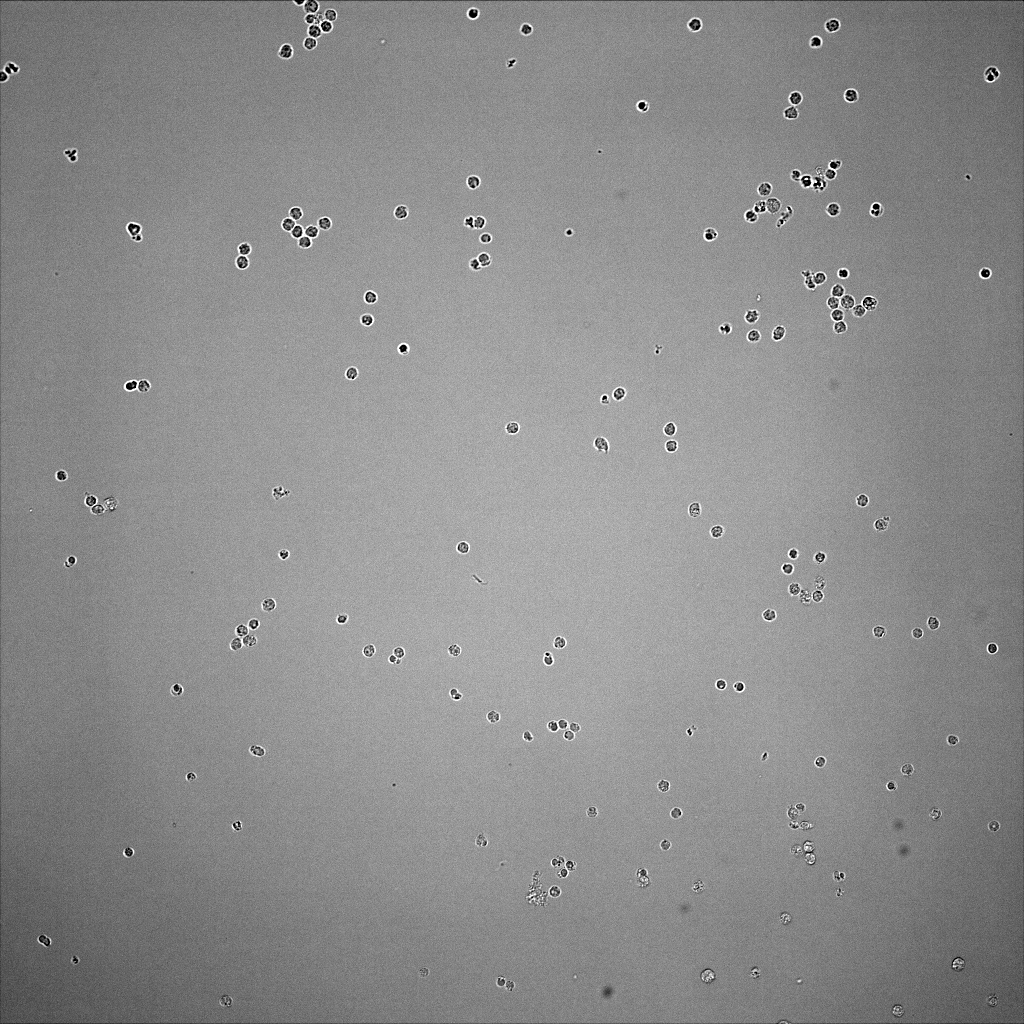

Supplement: Supplementary file 18 [file msb0011-0783-sd18.zip › Snap-46_c1_ORG.png]

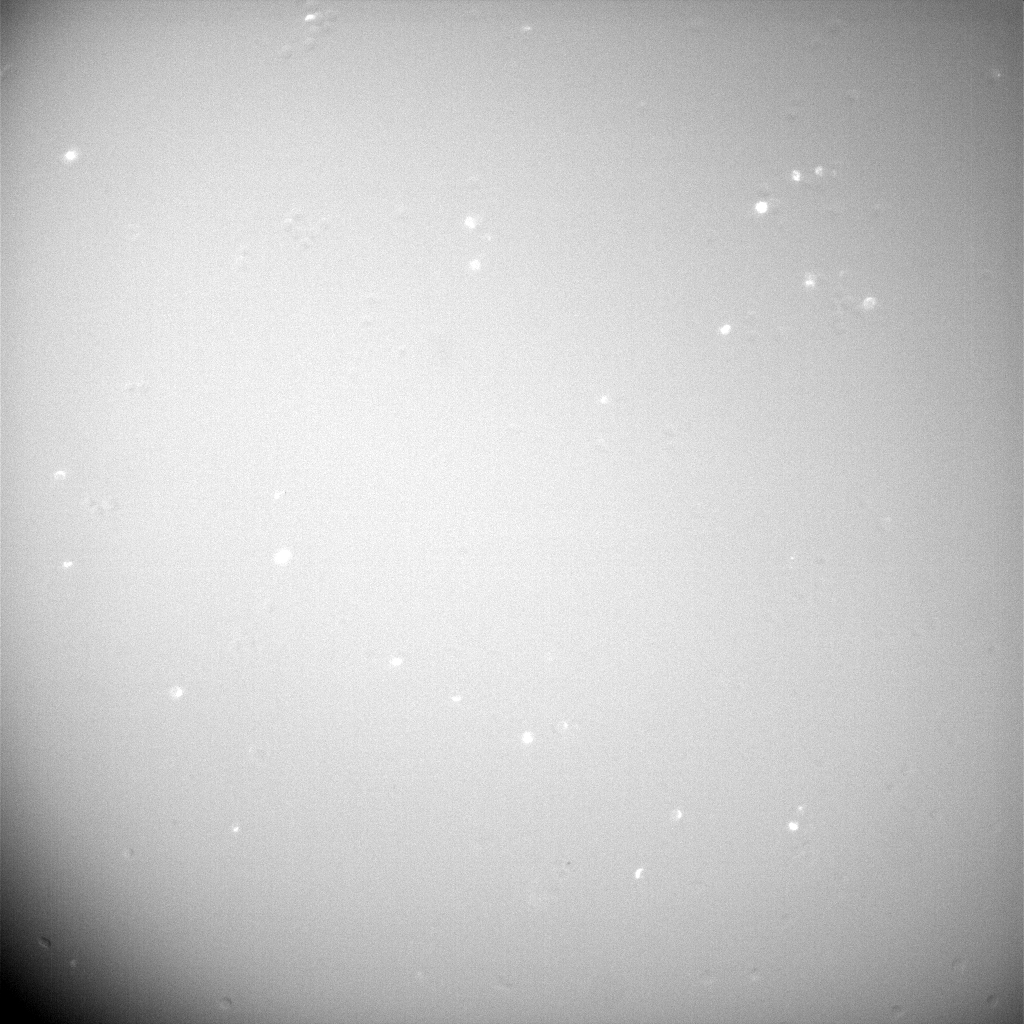

Supplement: Supplementary file 18 [file msb0011-0783-sd18.zip › Snap-46_c2_ORG.png]

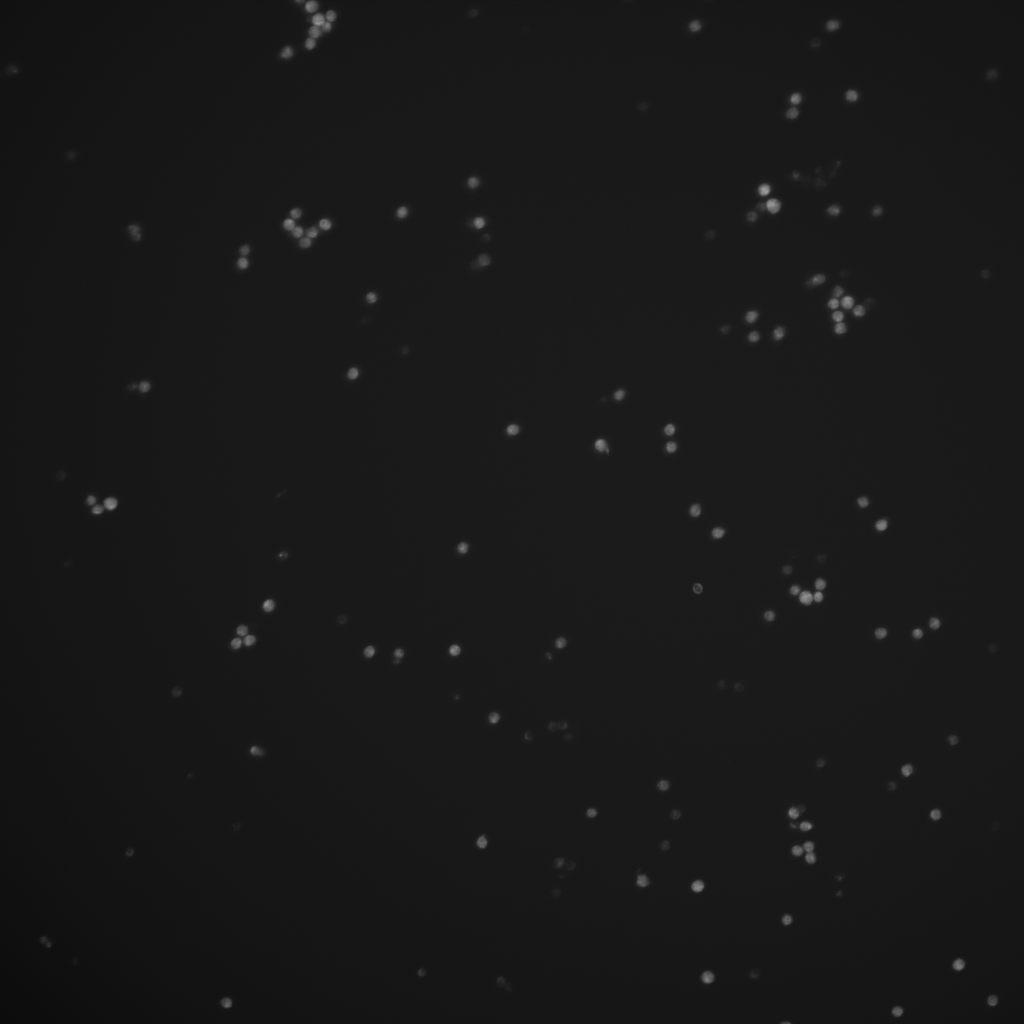

Supplement: Supplementary file 18 [file msb0011-0783-sd18.zip › Snap-46_c3_ORG.png]

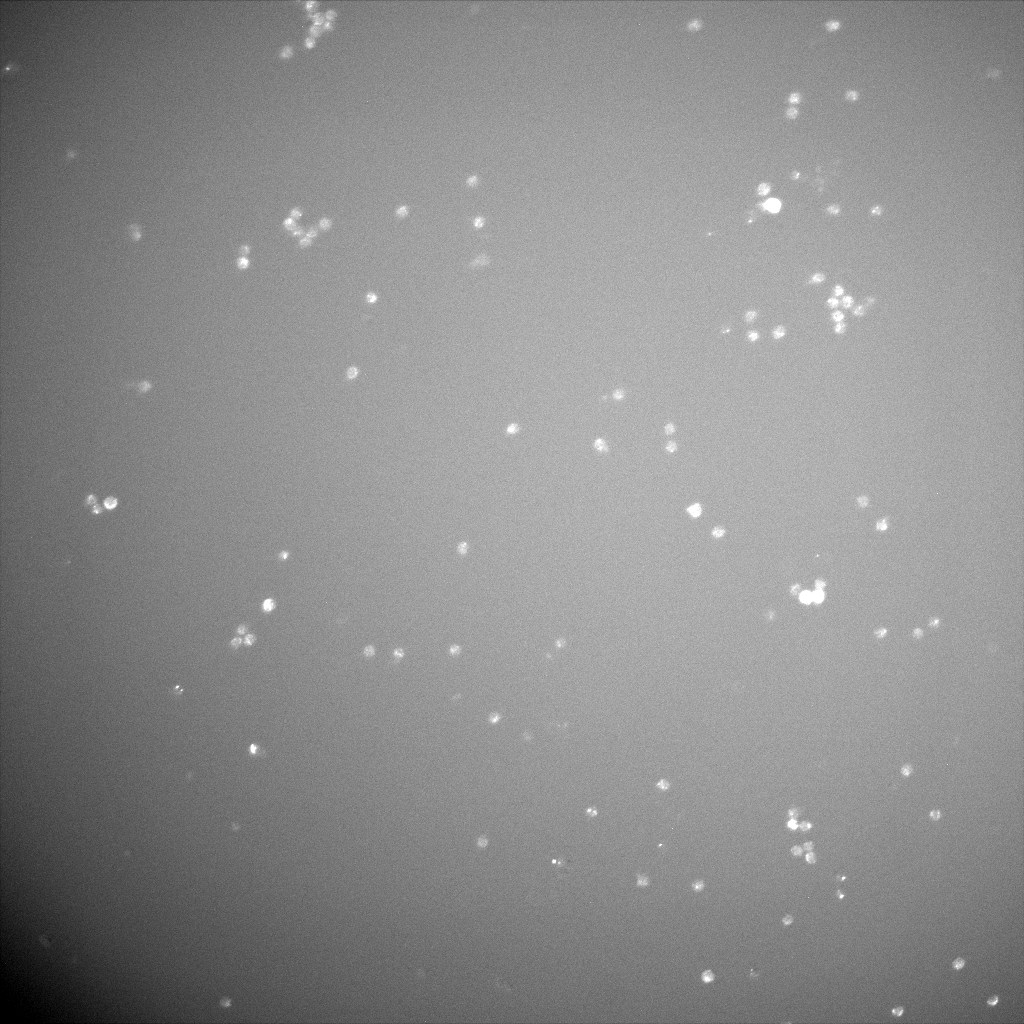

Supplement: Supplementary file 18 [file msb0011-0783-sd18.zip › Snap-46_c4_ORG.png]

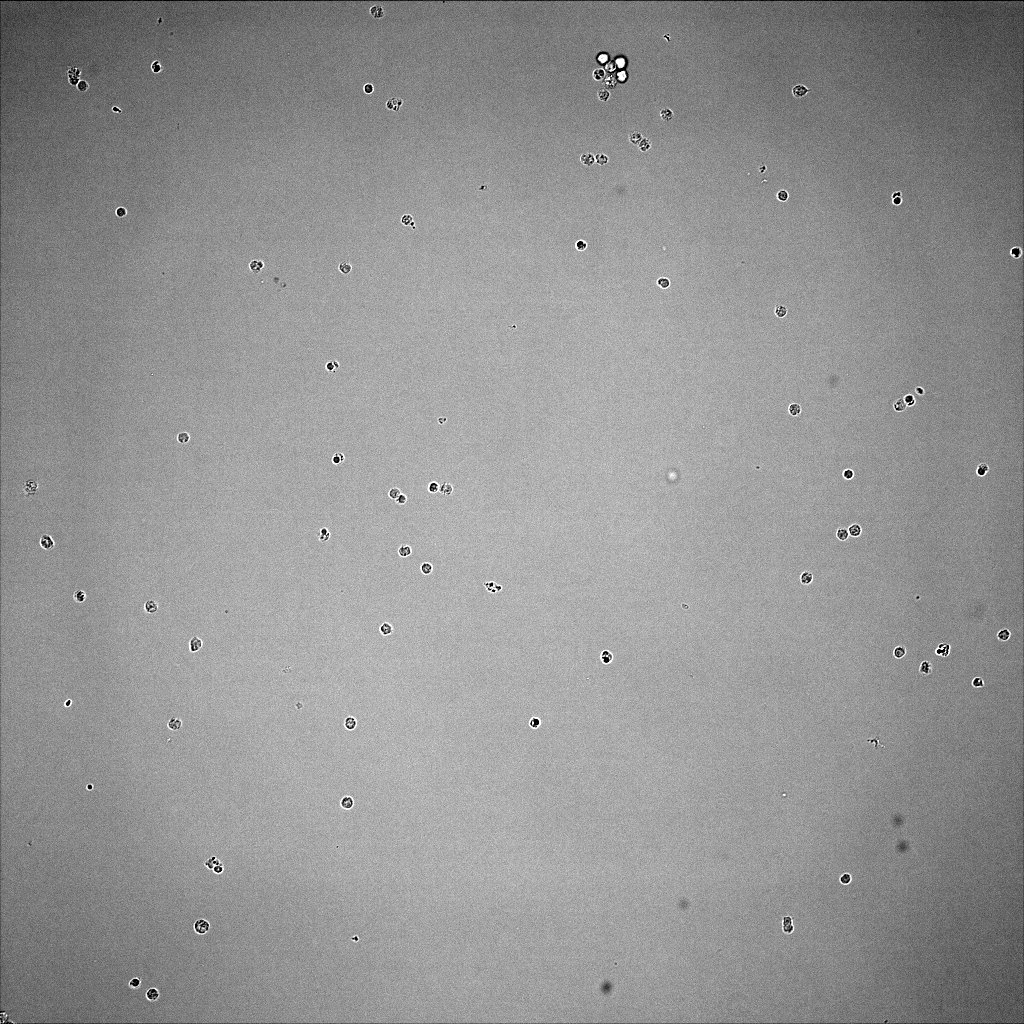

Supplement: Supplementary file 18 [file msb0011-0783-sd18.zip › Snap-47_c1_ORG.png]

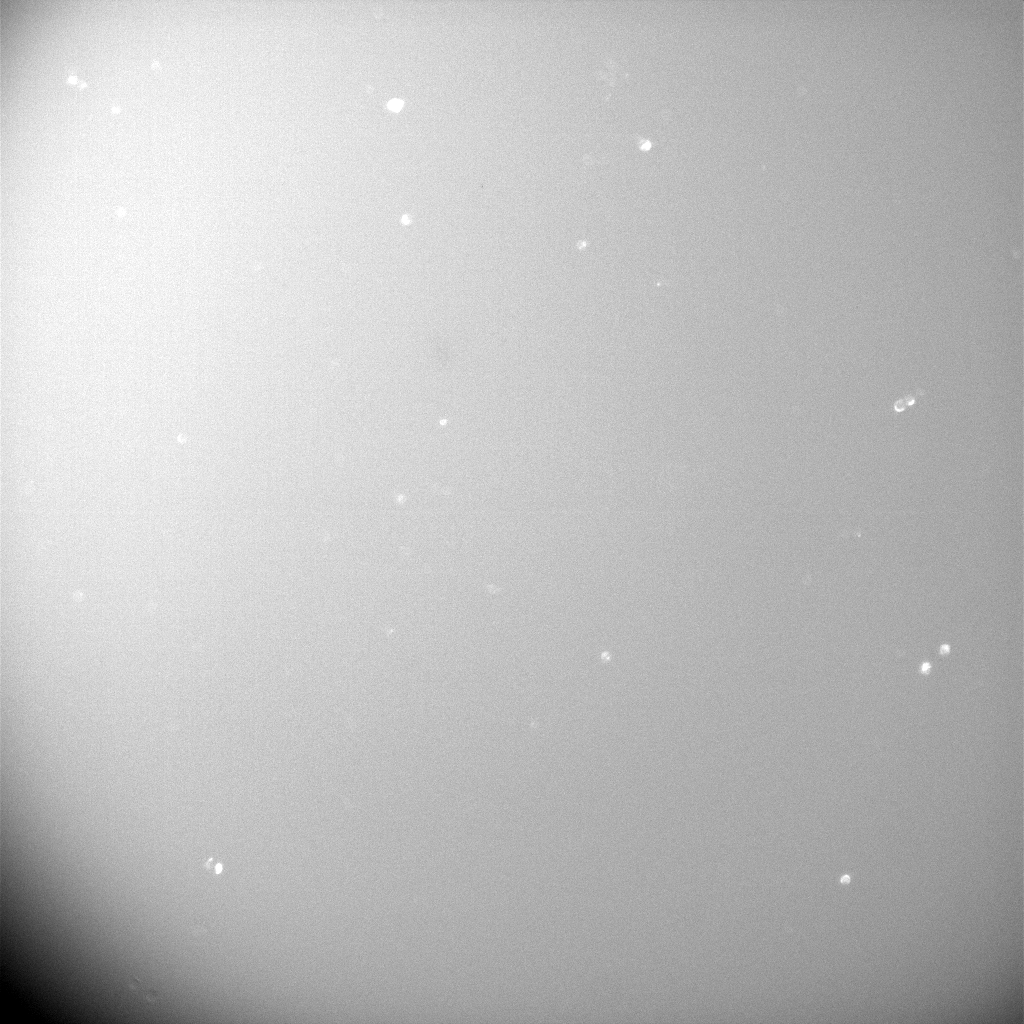

Supplement: Supplementary file 18 [file msb0011-0783-sd18.zip › Snap-47_c2_ORG.png]

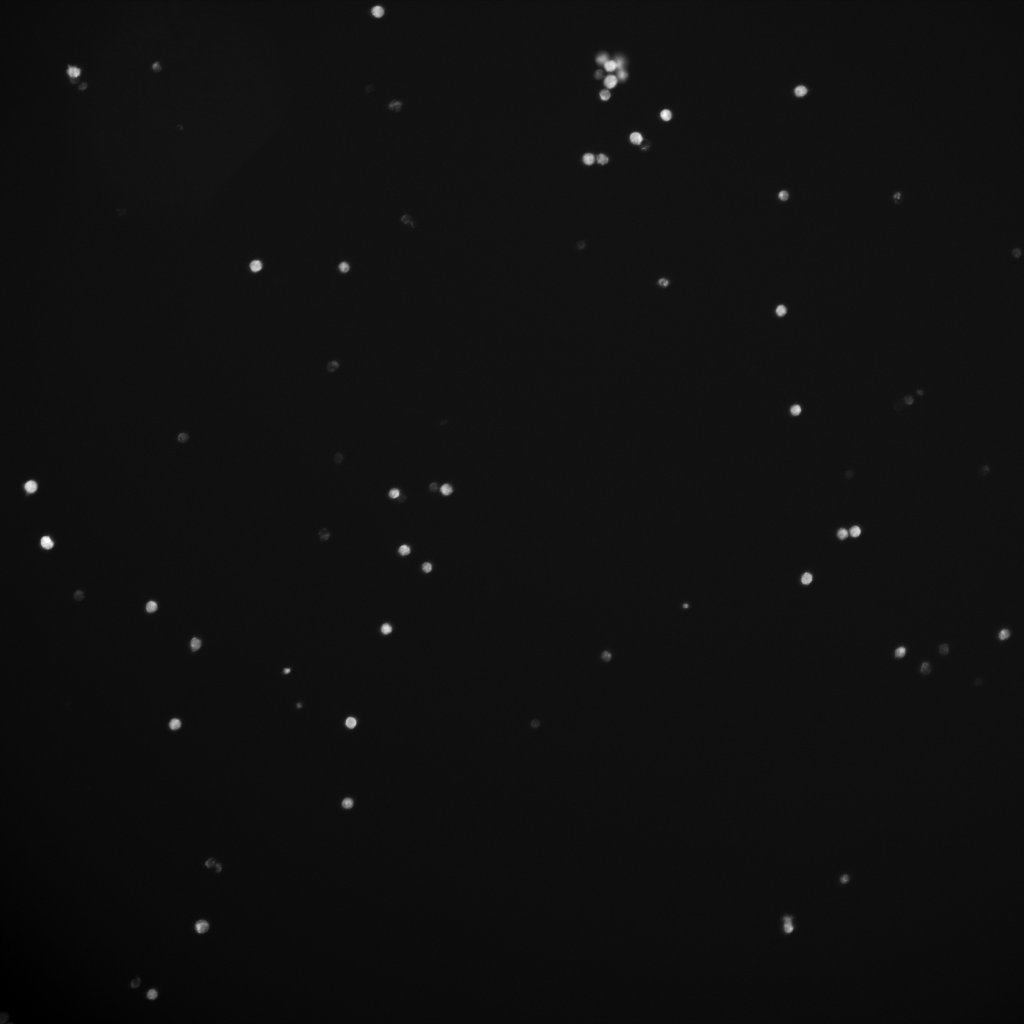

Supplement: Supplementary file 18 [file msb0011-0783-sd18.zip › Snap-47_c3_ORG.png]

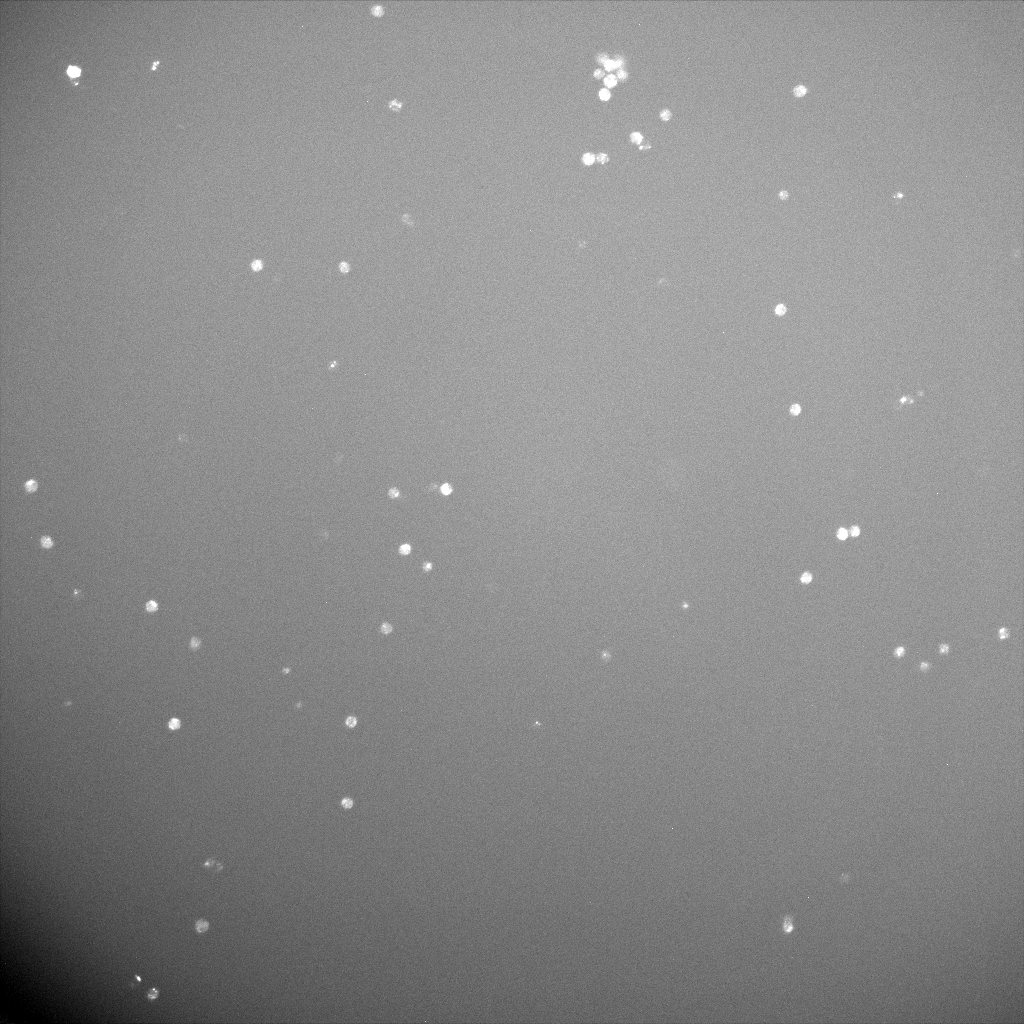

Supplement: Supplementary file 18 [file msb0011-0783-sd18.zip › Snap-47_c4_ORG.png]

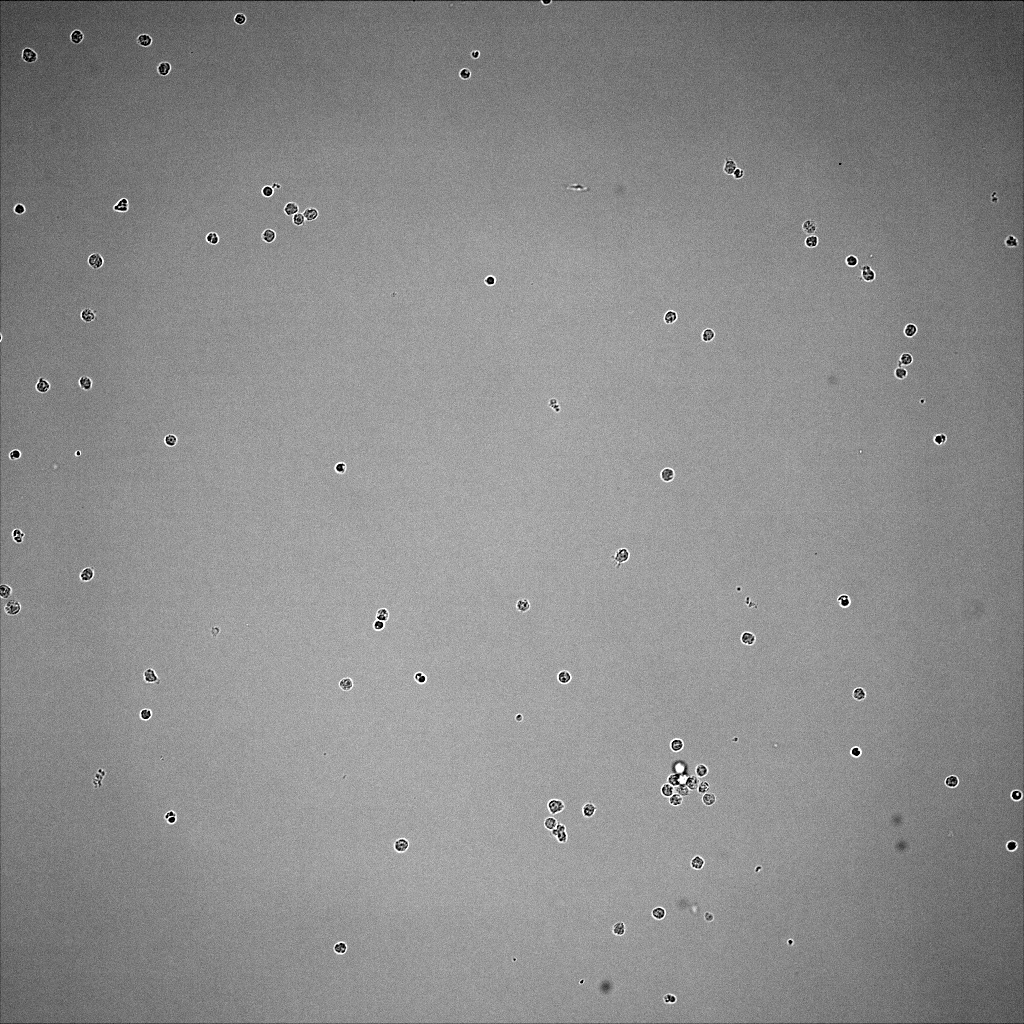

Supplement: Supplementary file 18 [file msb0011-0783-sd18.zip › Snap-48_c1_ORG.png]

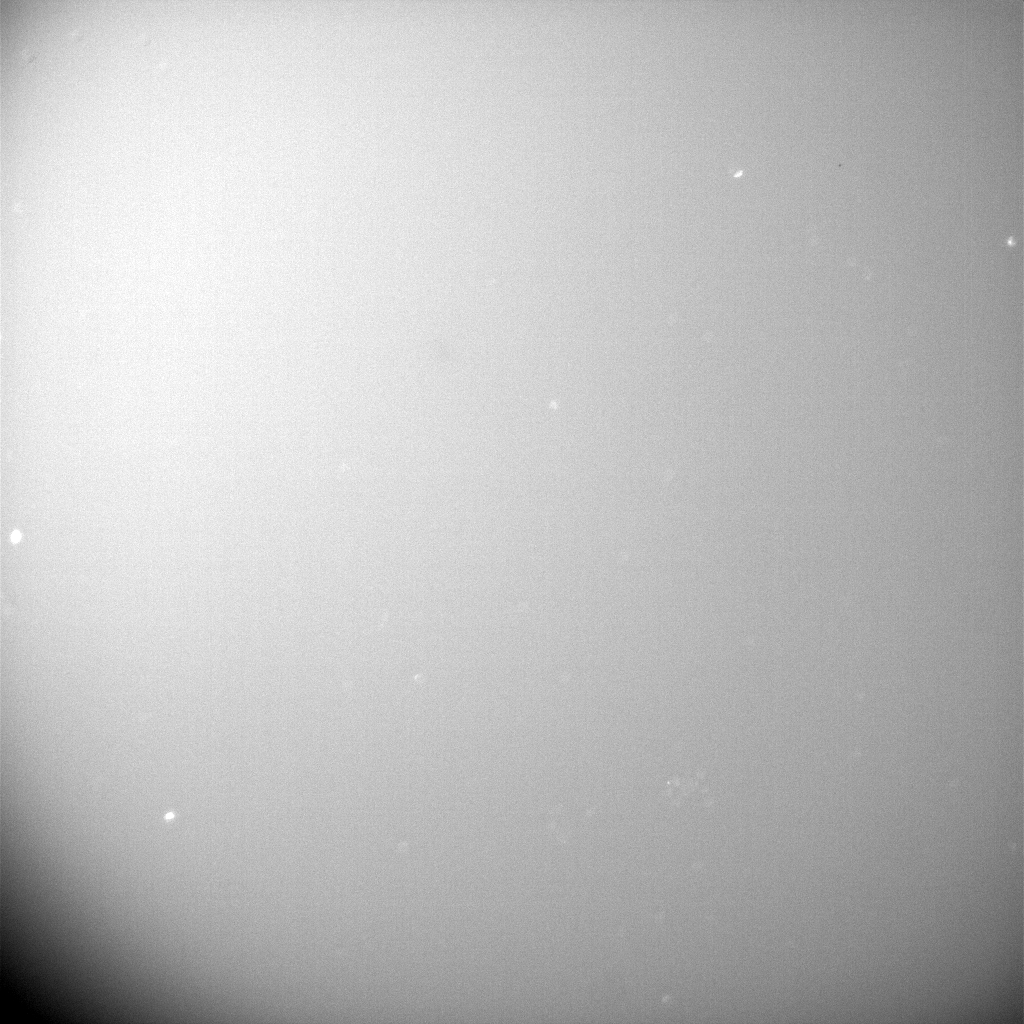

Supplement: Supplementary file 18 [file msb0011-0783-sd18.zip › Snap-48_c2_ORG.png]

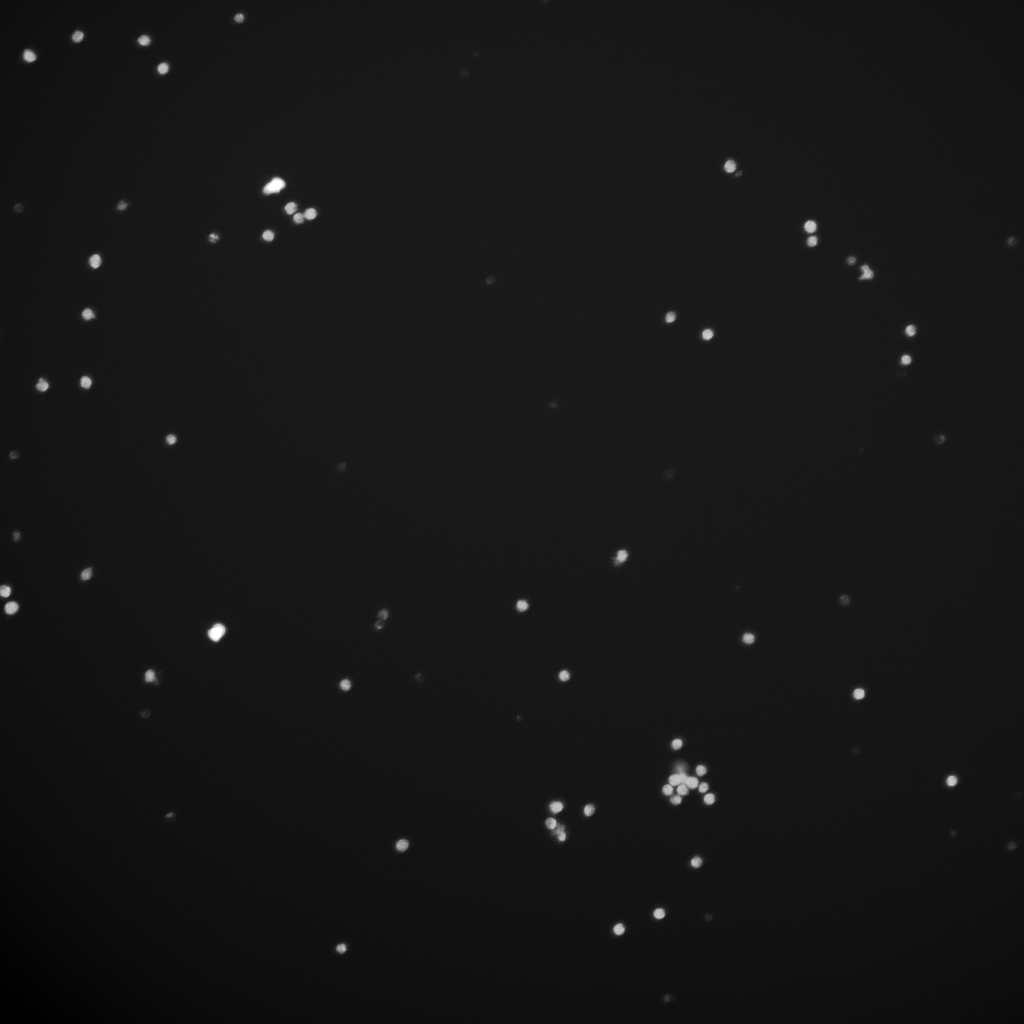

Supplement: Supplementary file 18 [file msb0011-0783-sd18.zip › Snap-48_c3_ORG.png]

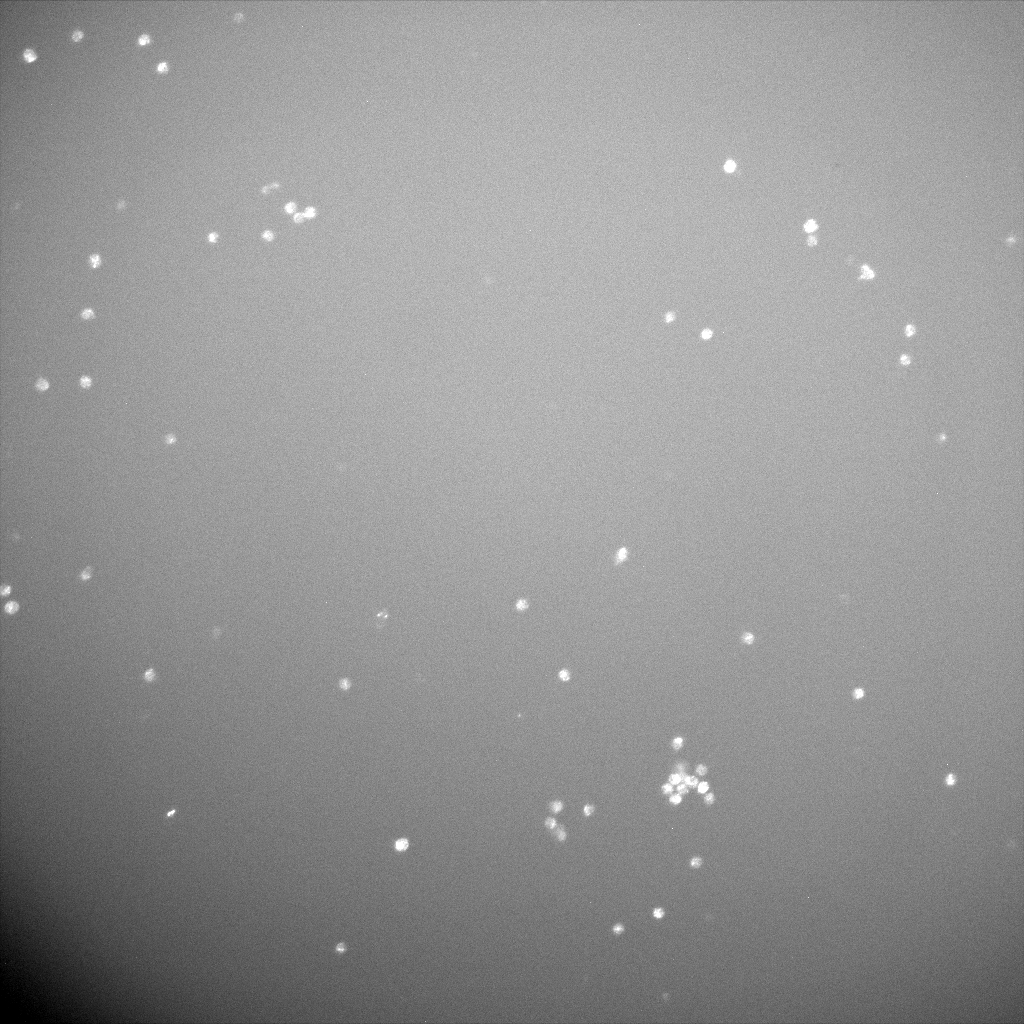

Supplement: Supplementary file 18 [file msb0011-0783-sd18.zip › Snap-48_c4_ORG.png]

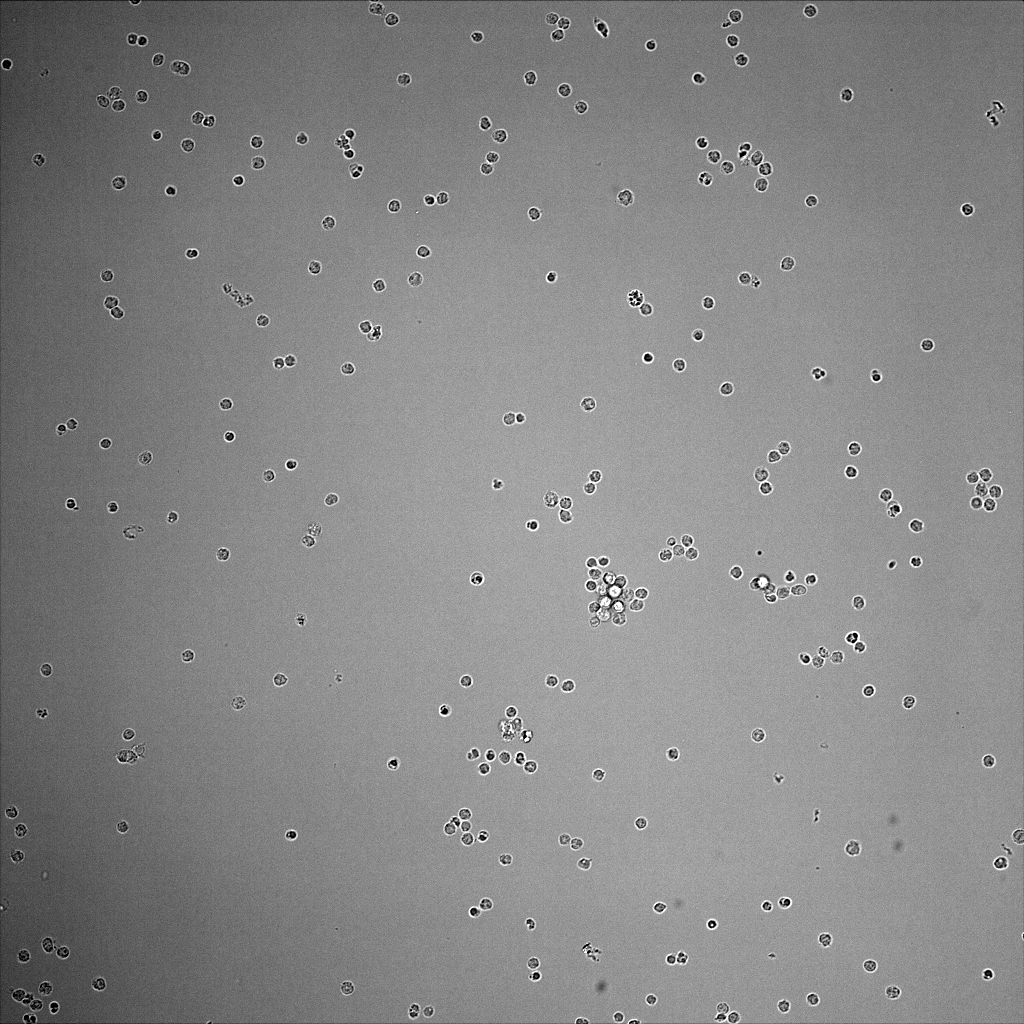

Supplement: Supplementary file 18 [file msb0011-0783-sd18.zip › Snap-49_c1_ORG.png]

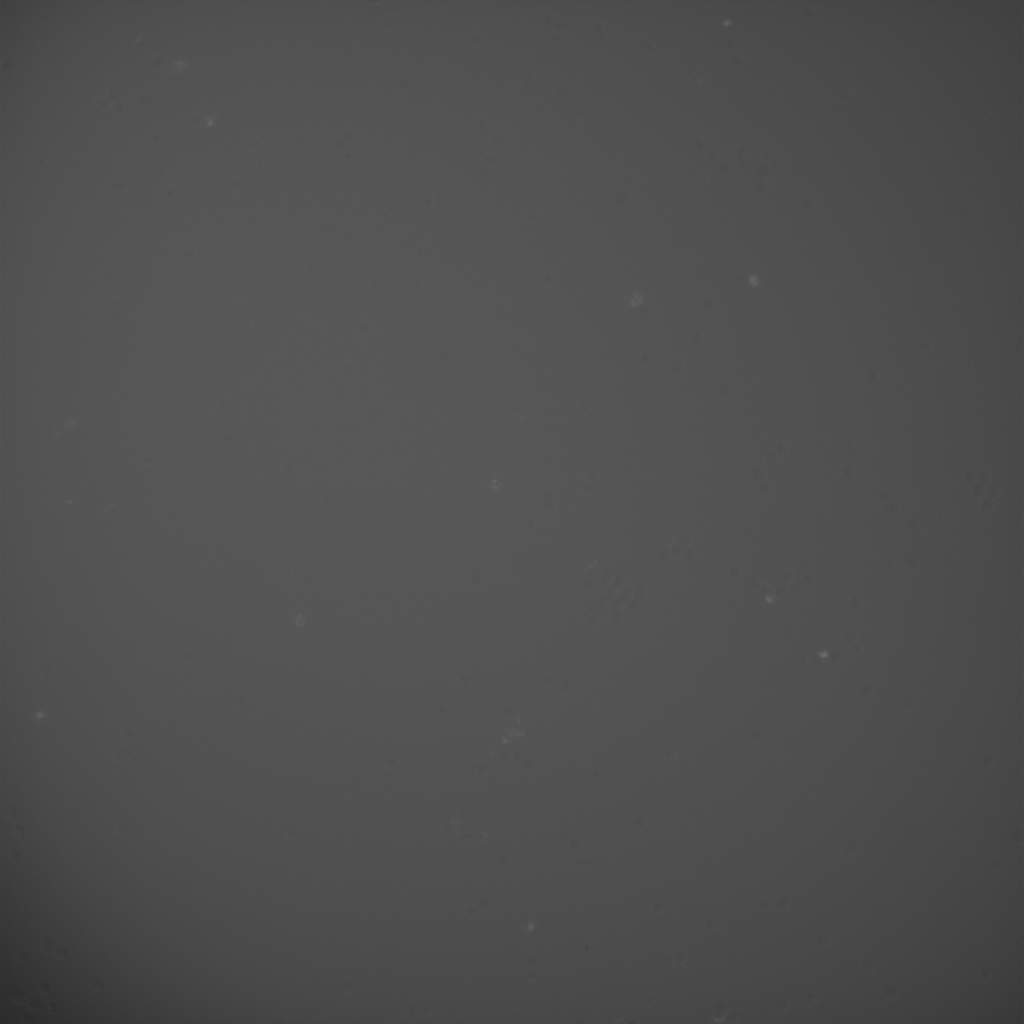

Supplement: Supplementary file 18 [file msb0011-0783-sd18.zip › Snap-49_c2_ORG.png]

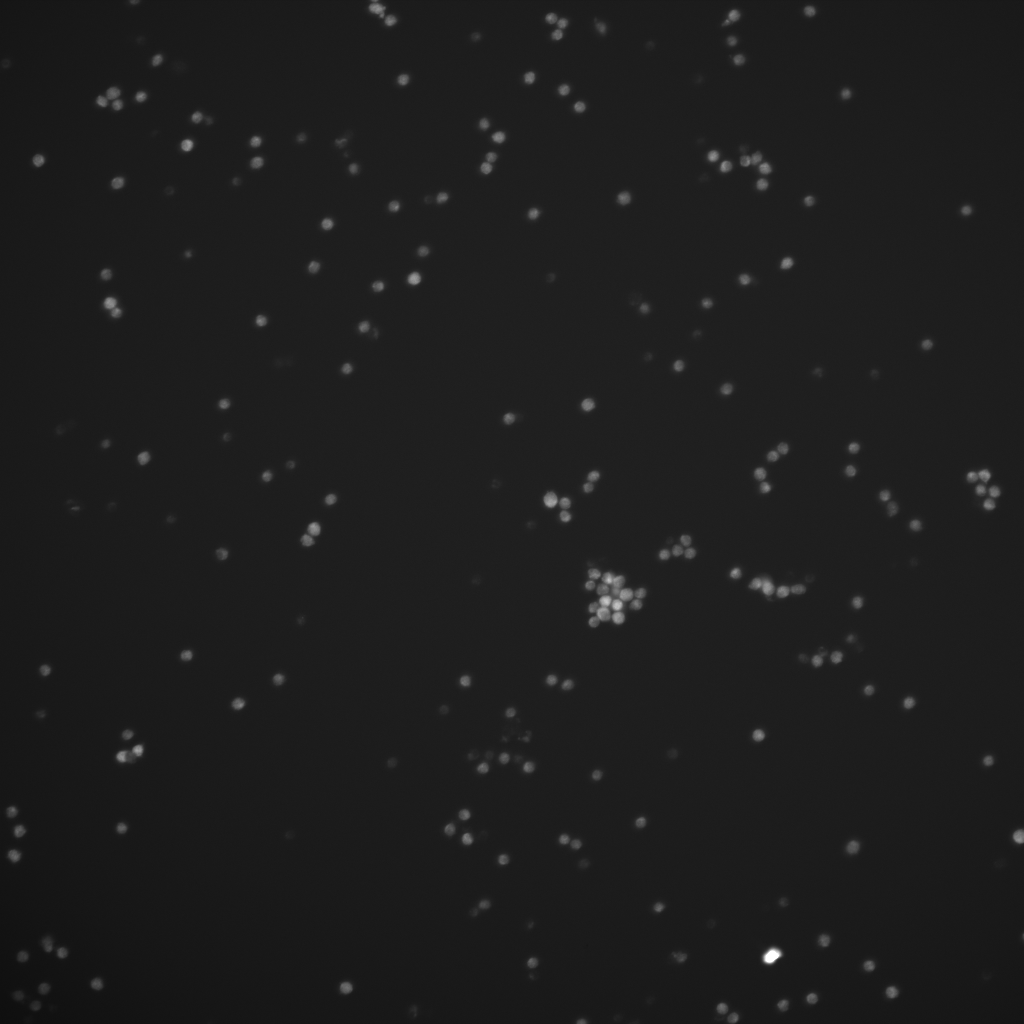

Supplement: Supplementary file 18 [file msb0011-0783-sd18.zip › Snap-49_c3_ORG.png]

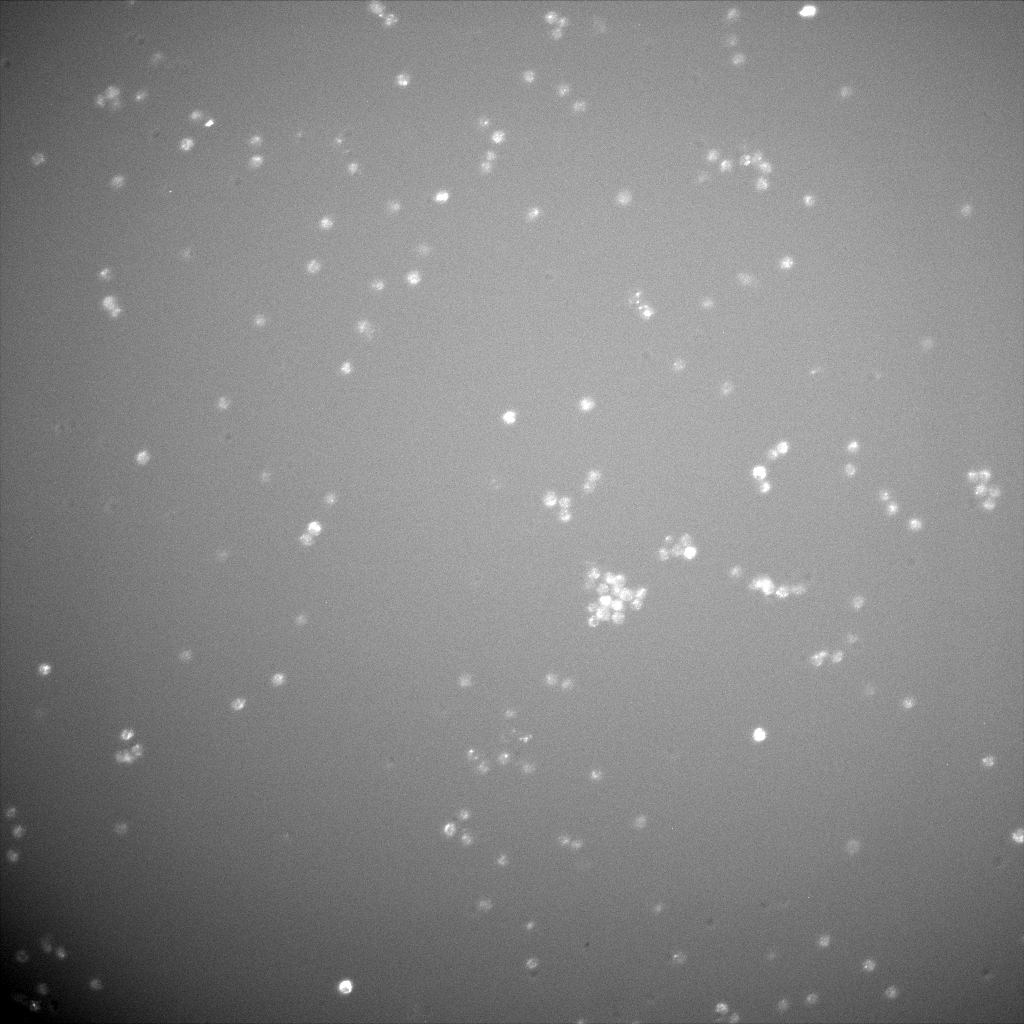

Supplement: Supplementary file 18 [file msb0011-0783-sd18.zip › Snap-49_c4_ORG.png]

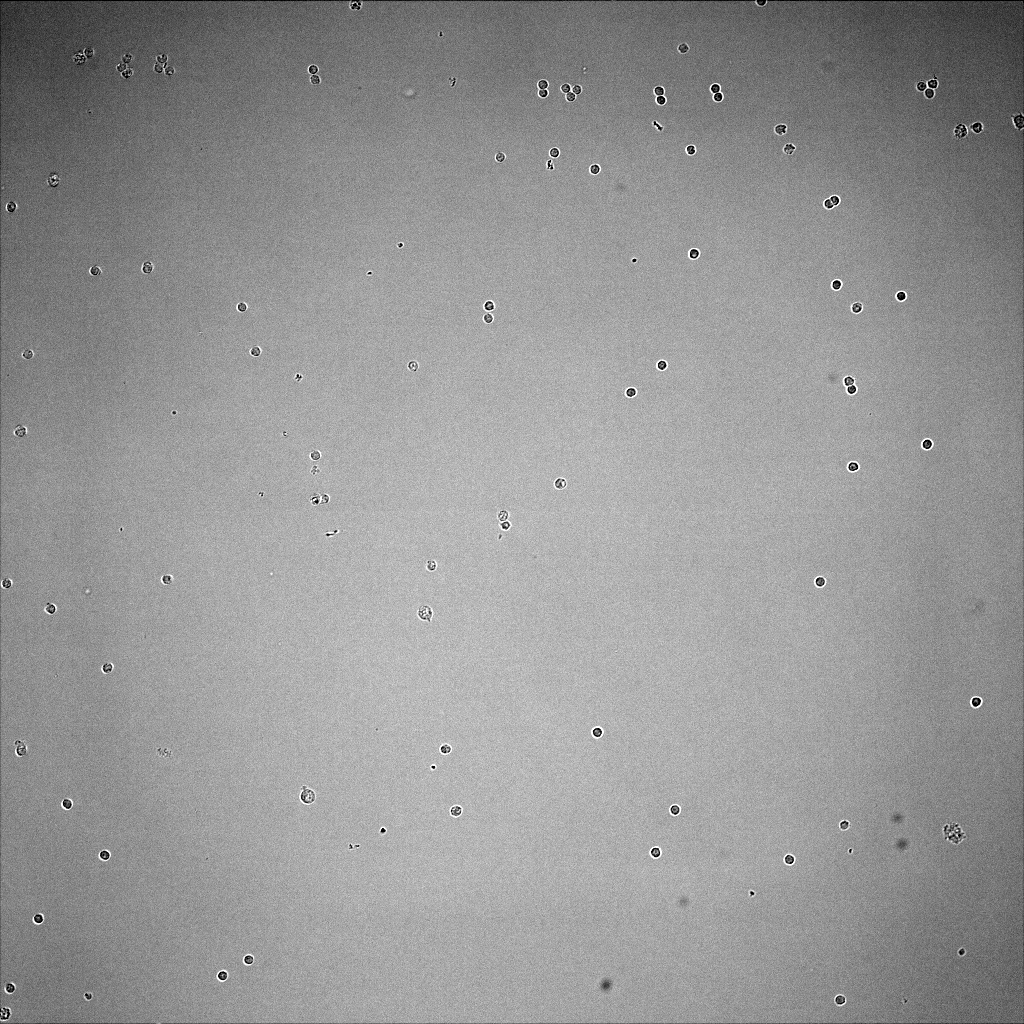

Supplement: Supplementary file 18 [file msb0011-0783-sd18.zip › Snap-38_c1_ORG.png]

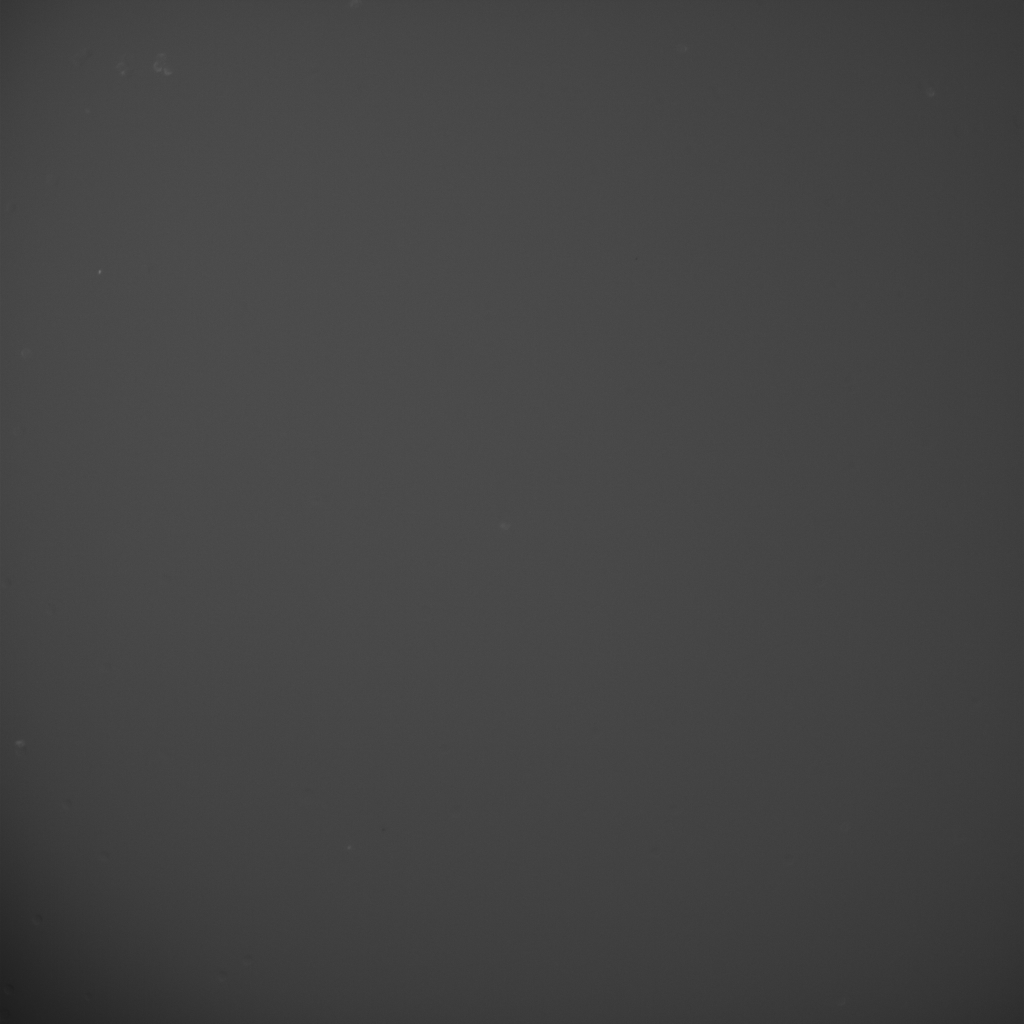

Supplement: Supplementary file 18 [file msb0011-0783-sd18.zip › Snap-38_c2_ORG.png]

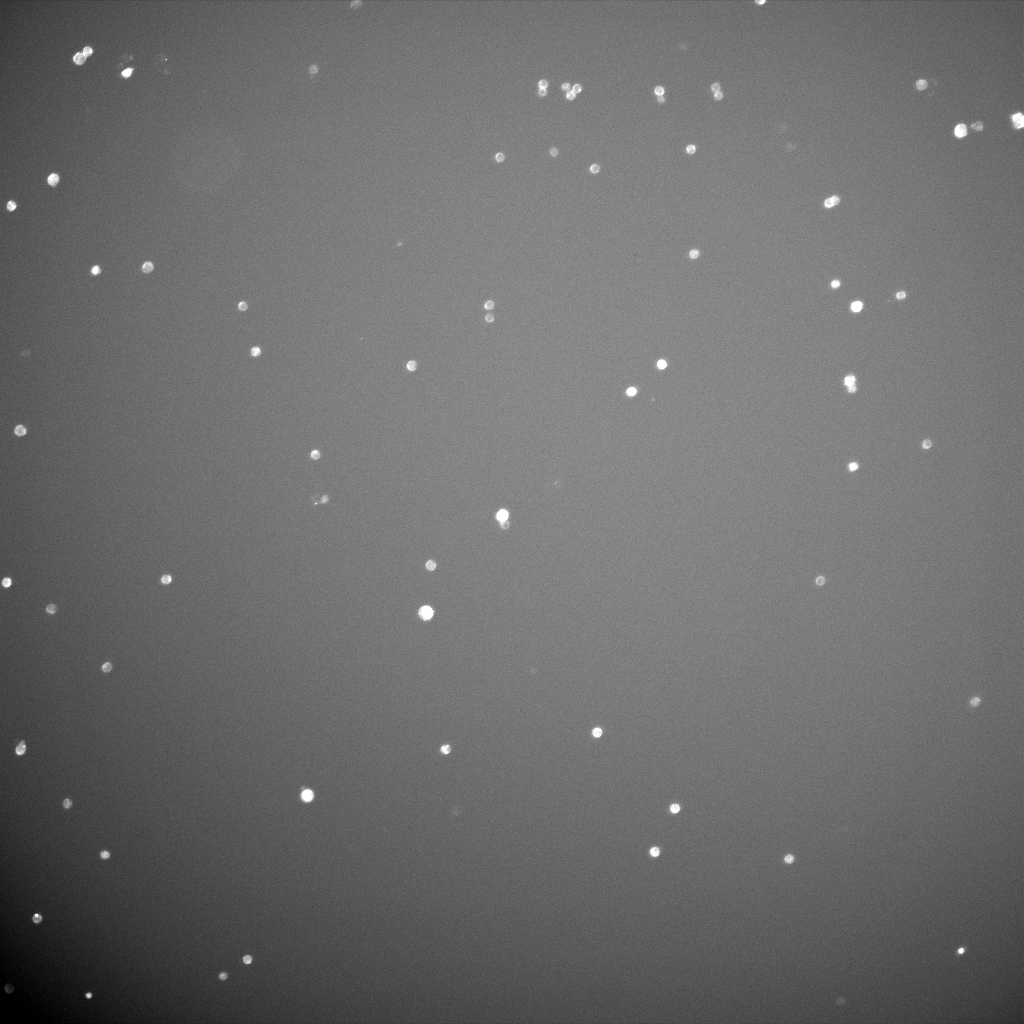

Supplement: Supplementary file 18 [file msb0011-0783-sd18.zip › Snap-38_c3_ORG.png]

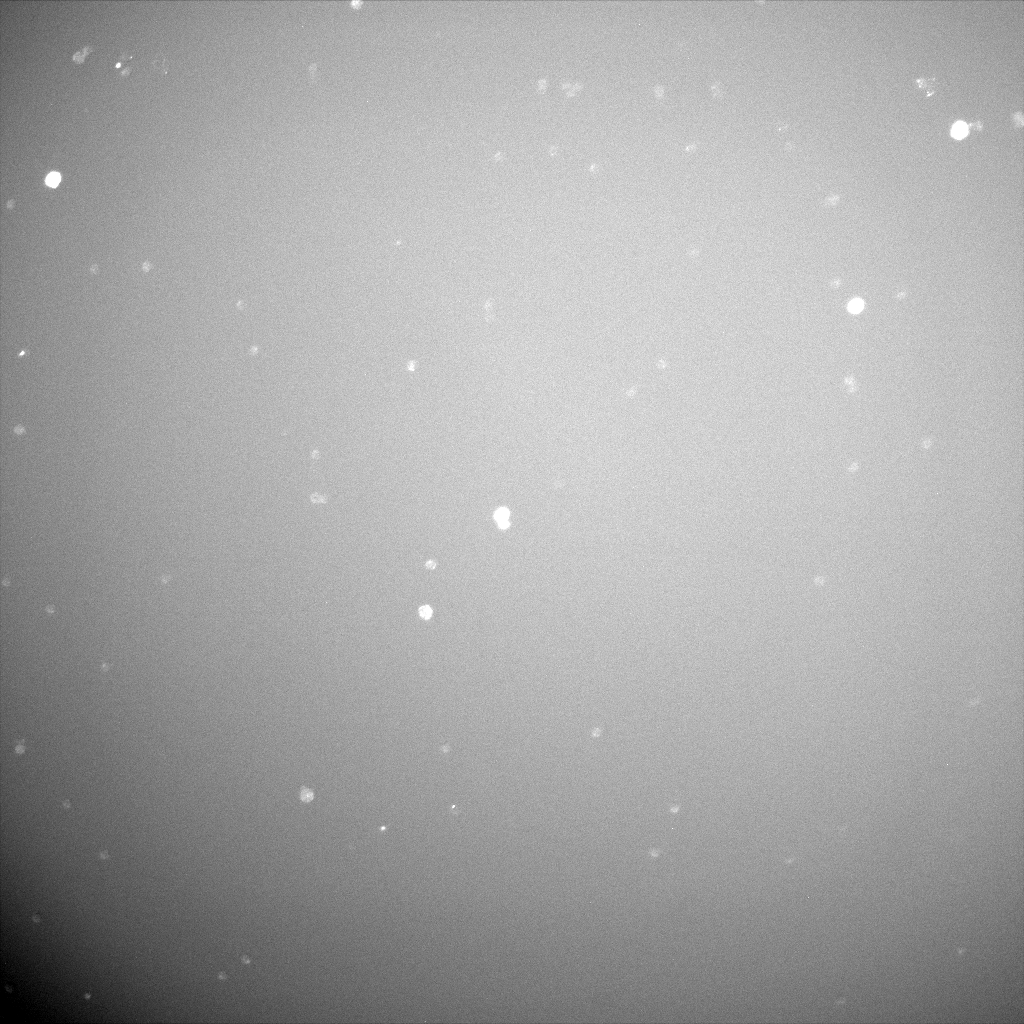

Supplement: Supplementary file 18 [file msb0011-0783-sd18.zip › Snap-38_c4_ORG.png]

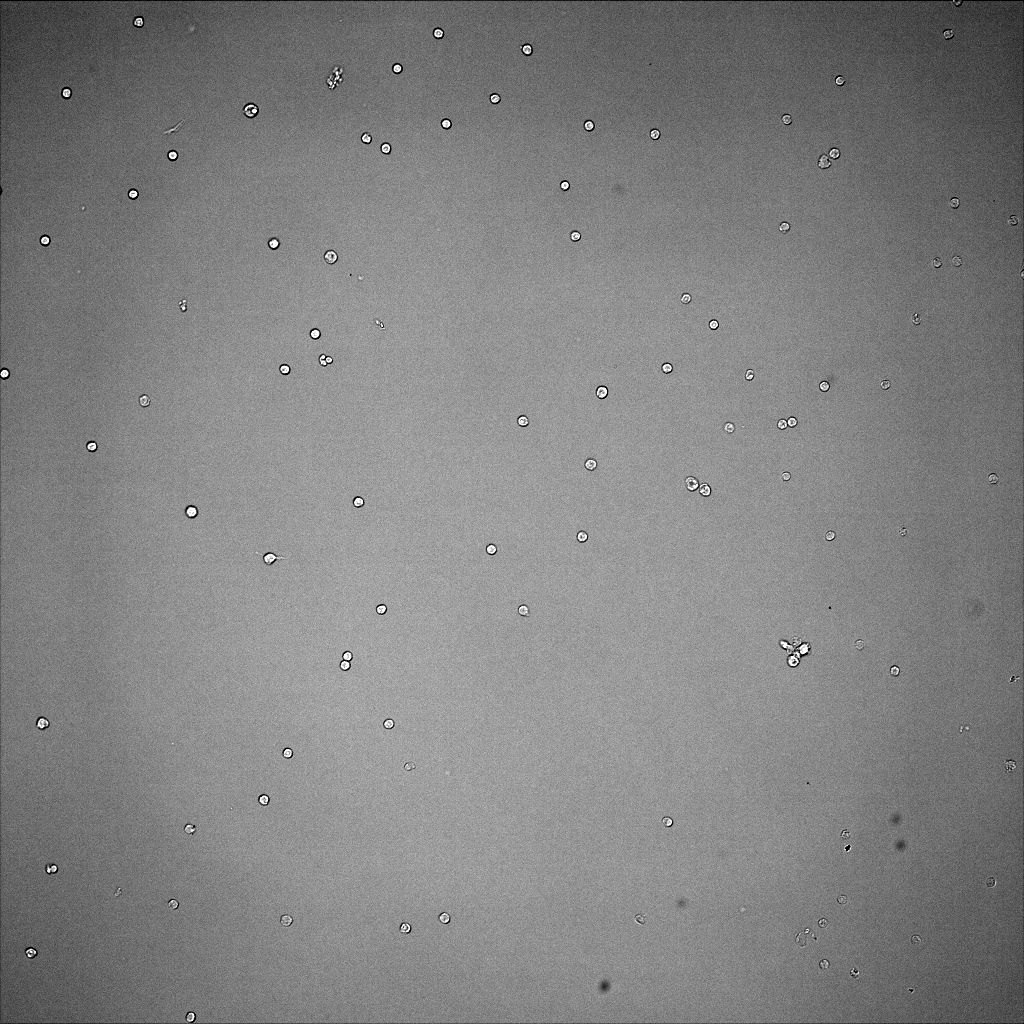

Supplement: Supplementary file 18 [file msb0011-0783-sd18.zip › Snap-39_c1_ORG.png]

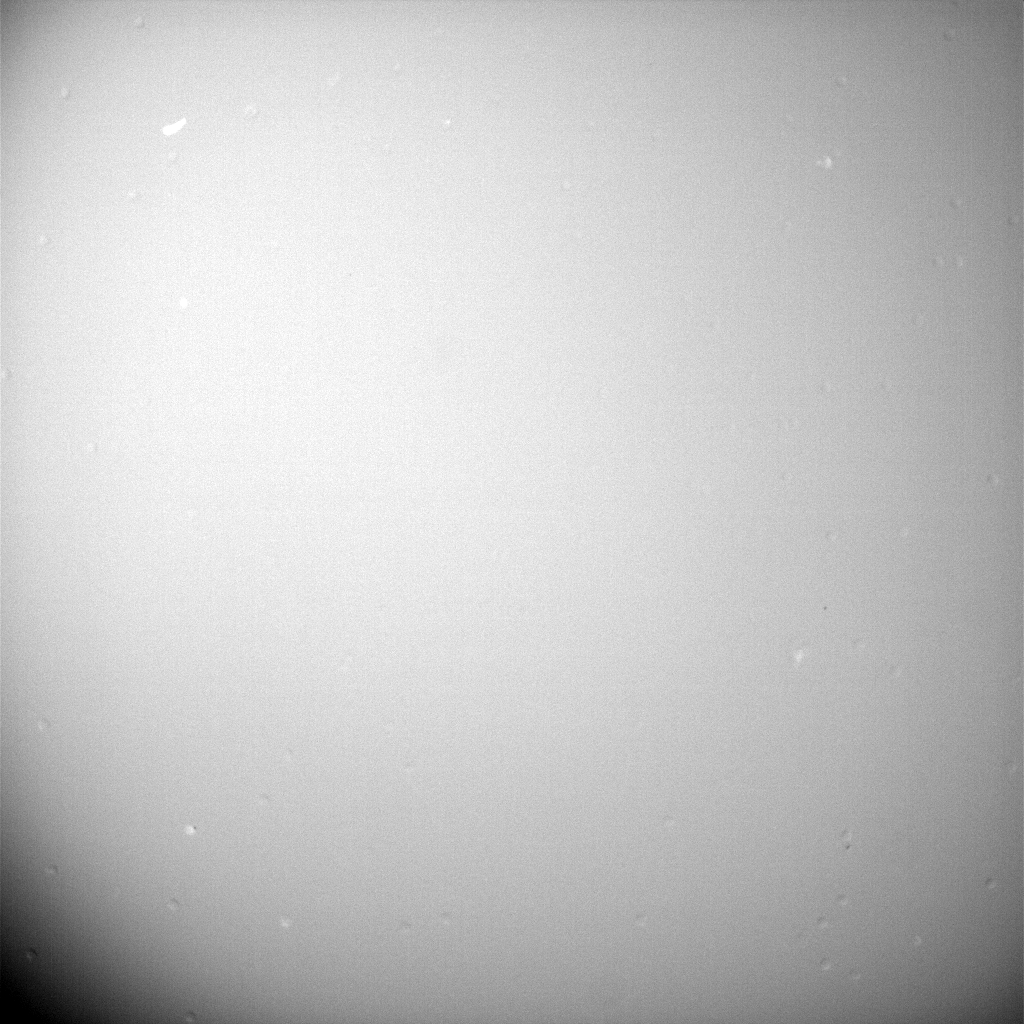

Supplement: Supplementary file 18 [file msb0011-0783-sd18.zip › Snap-39_c2_ORG.png]

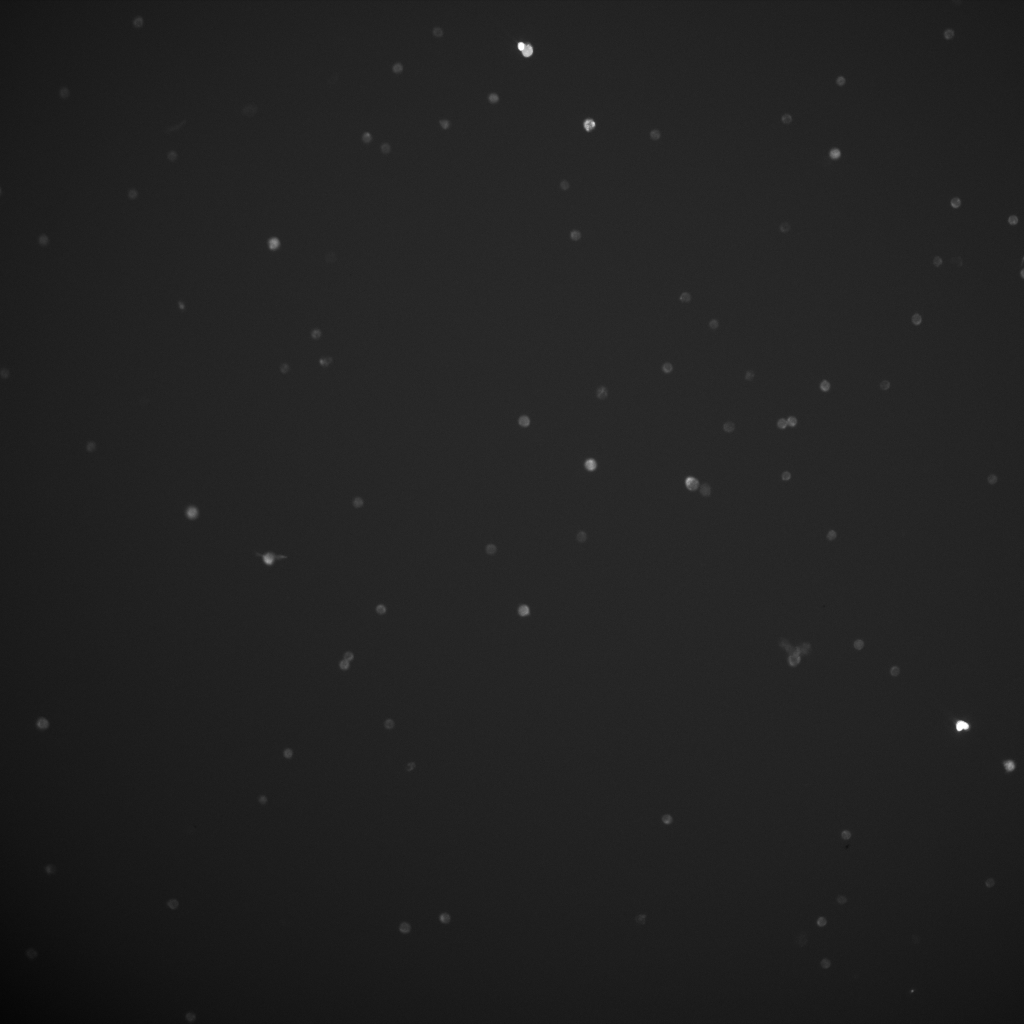

Supplement: Supplementary file 18 [file msb0011-0783-sd18.zip › Snap-39_c3_ORG.png]

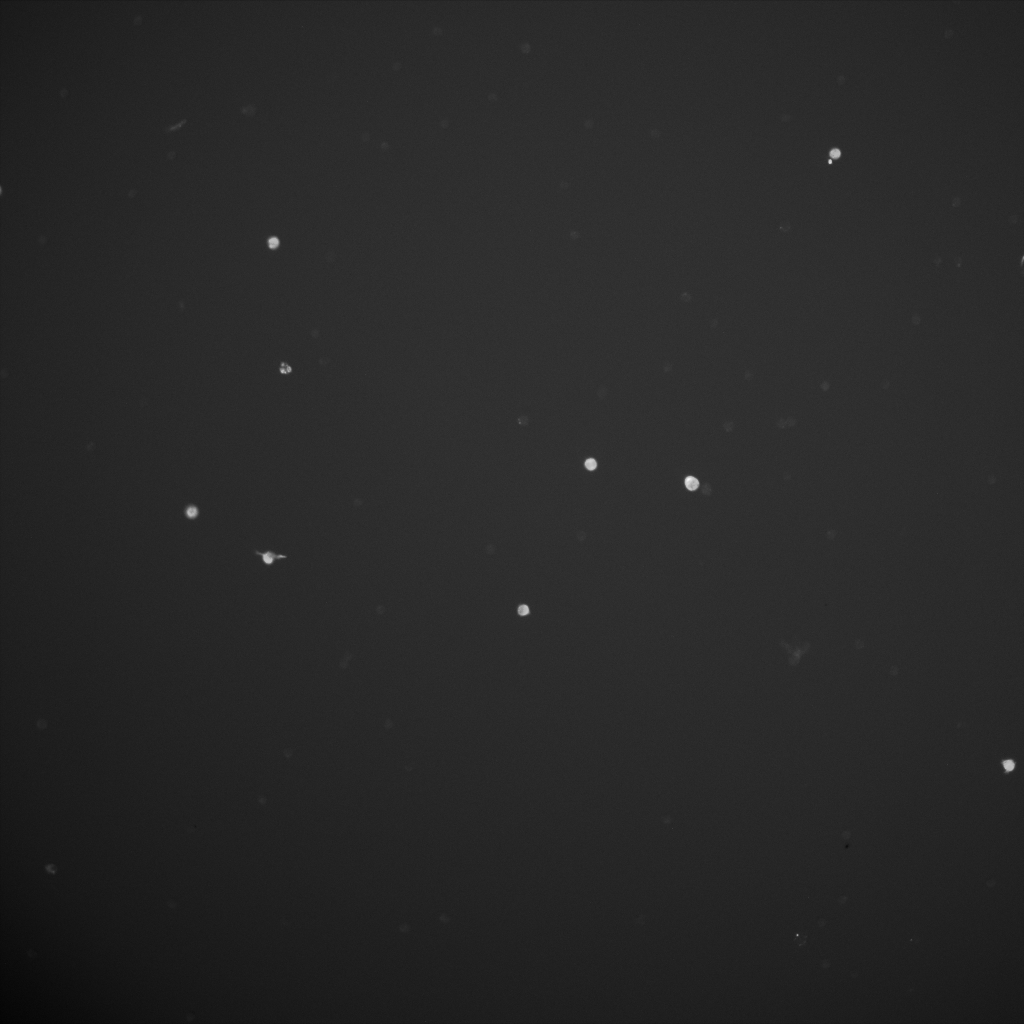

Supplement: Supplementary file 18 [file msb0011-0783-sd18.zip › Snap-39_c4_ORG.png]

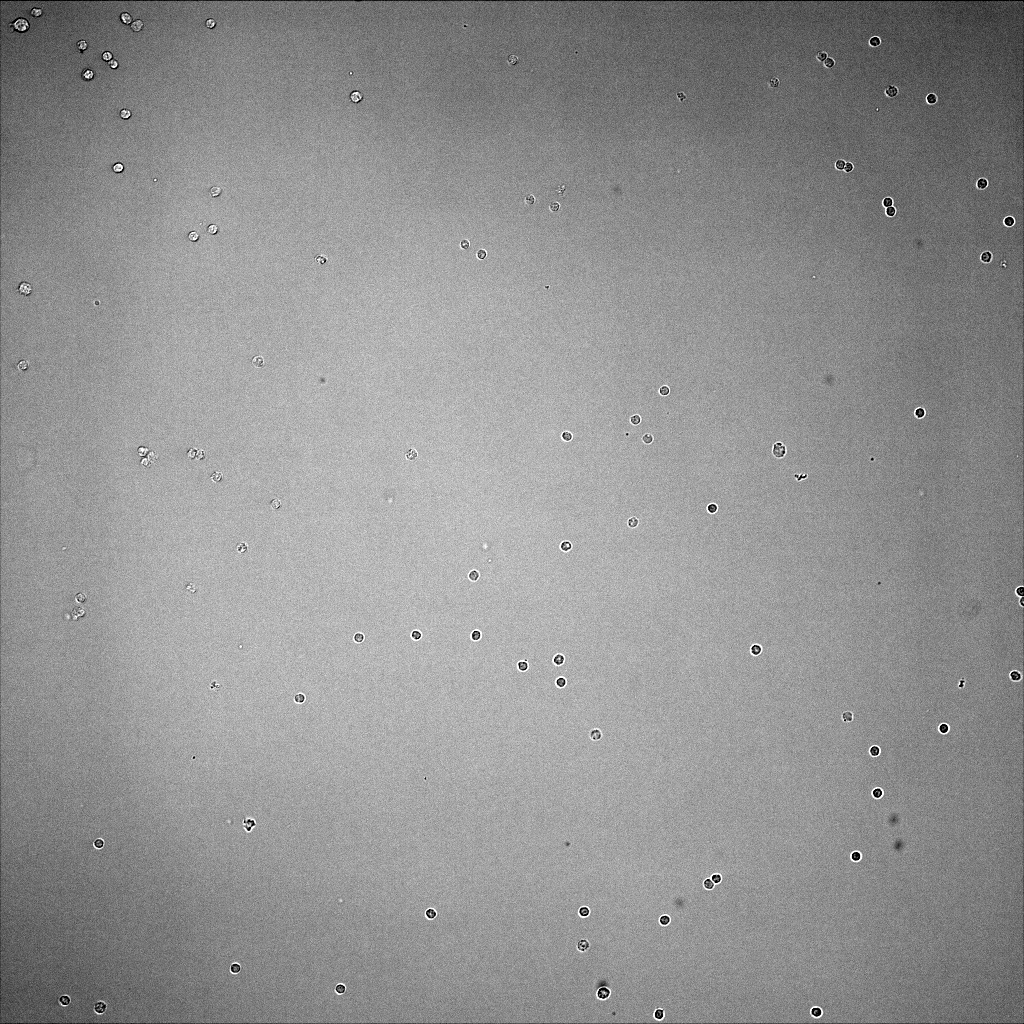

Supplement: Supplementary file 18 [file msb0011-0783-sd18.zip › Snap-40_c1_ORG.png]

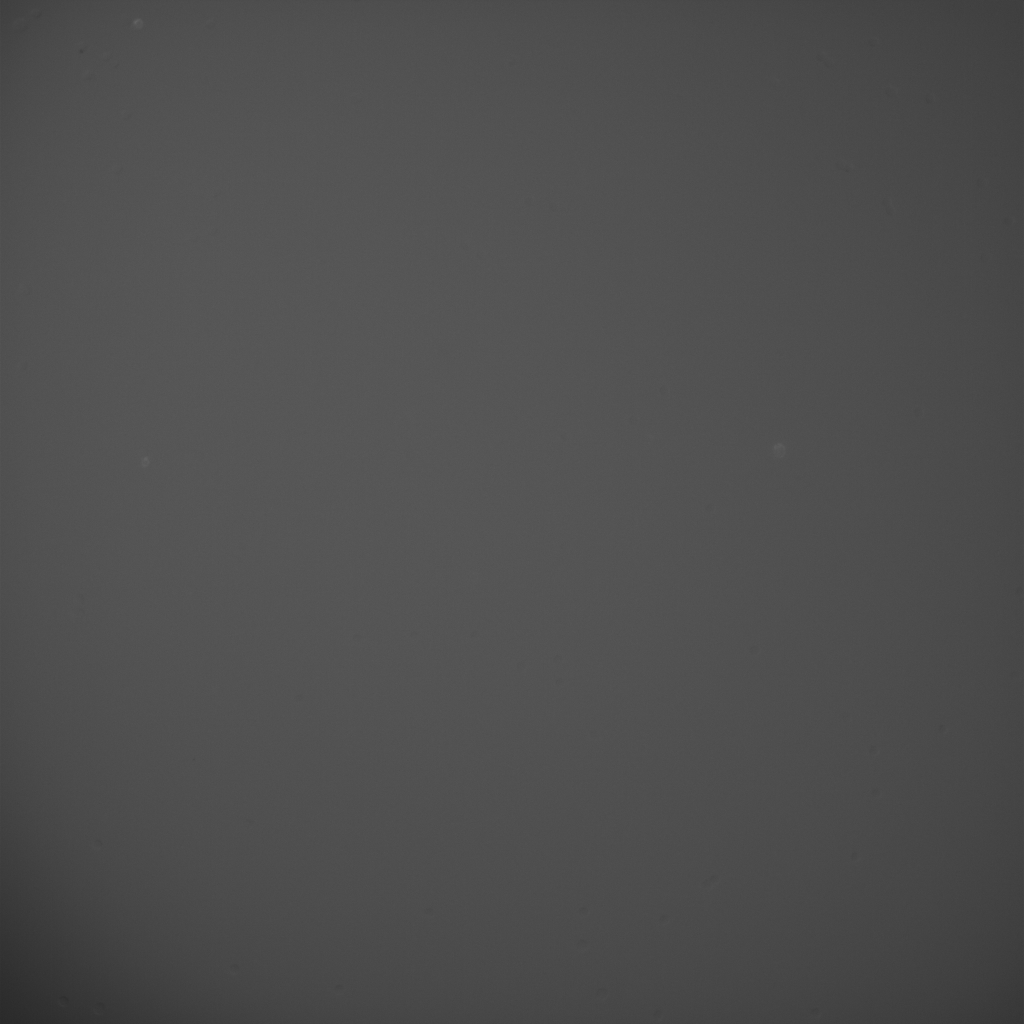

Supplement: Supplementary file 18 [file msb0011-0783-sd18.zip › Snap-40_c2_ORG.png]

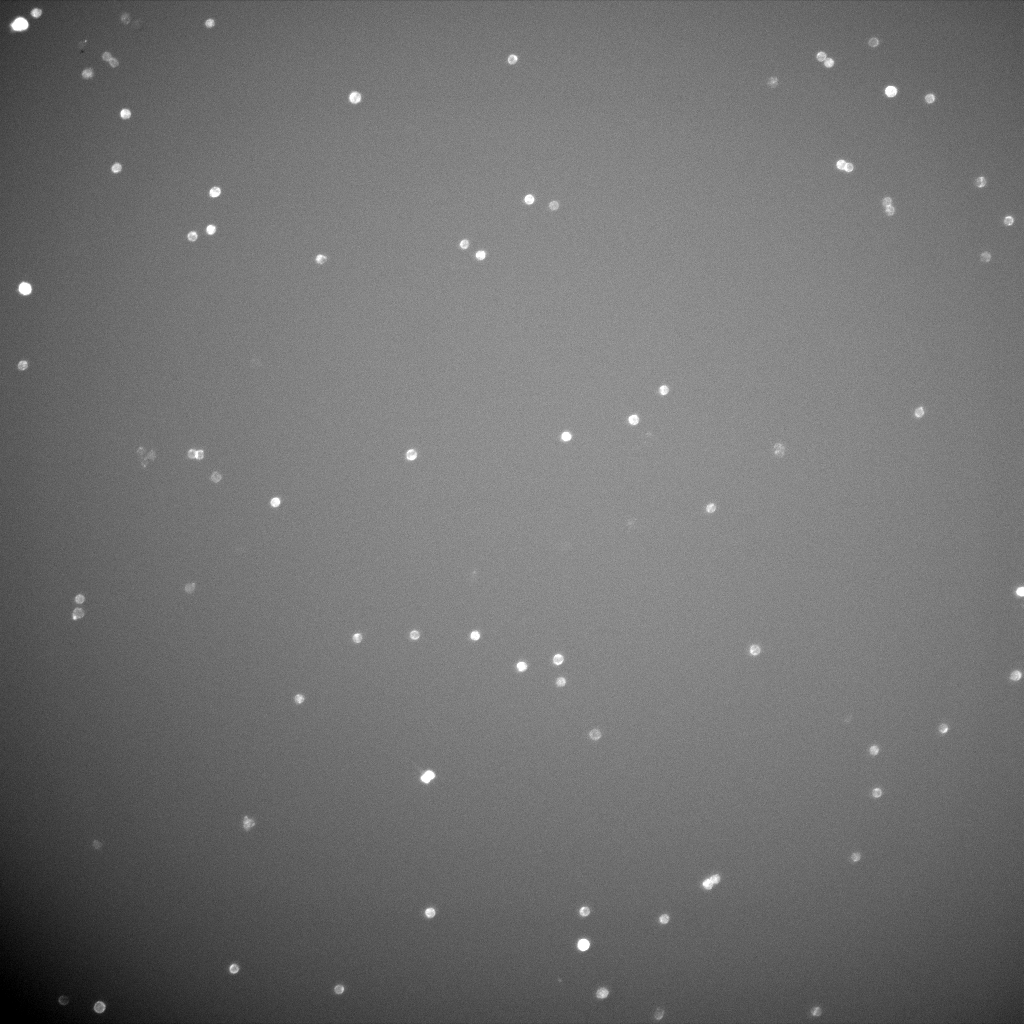

Supplement: Supplementary file 18 [file msb0011-0783-sd18.zip › Snap-40_c3_ORG.png]

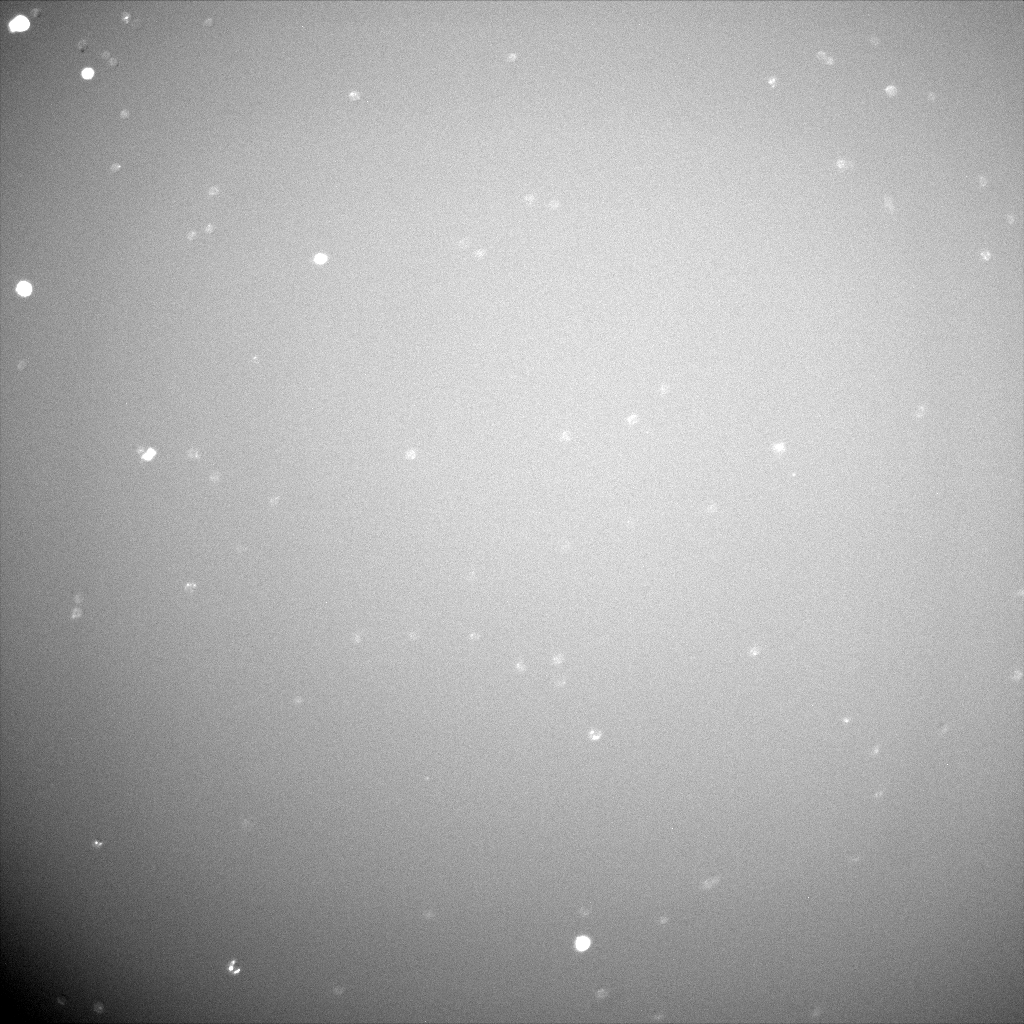

Supplement: Supplementary file 18 [file msb0011-0783-sd18.zip › Snap-40_c4_ORG.png]

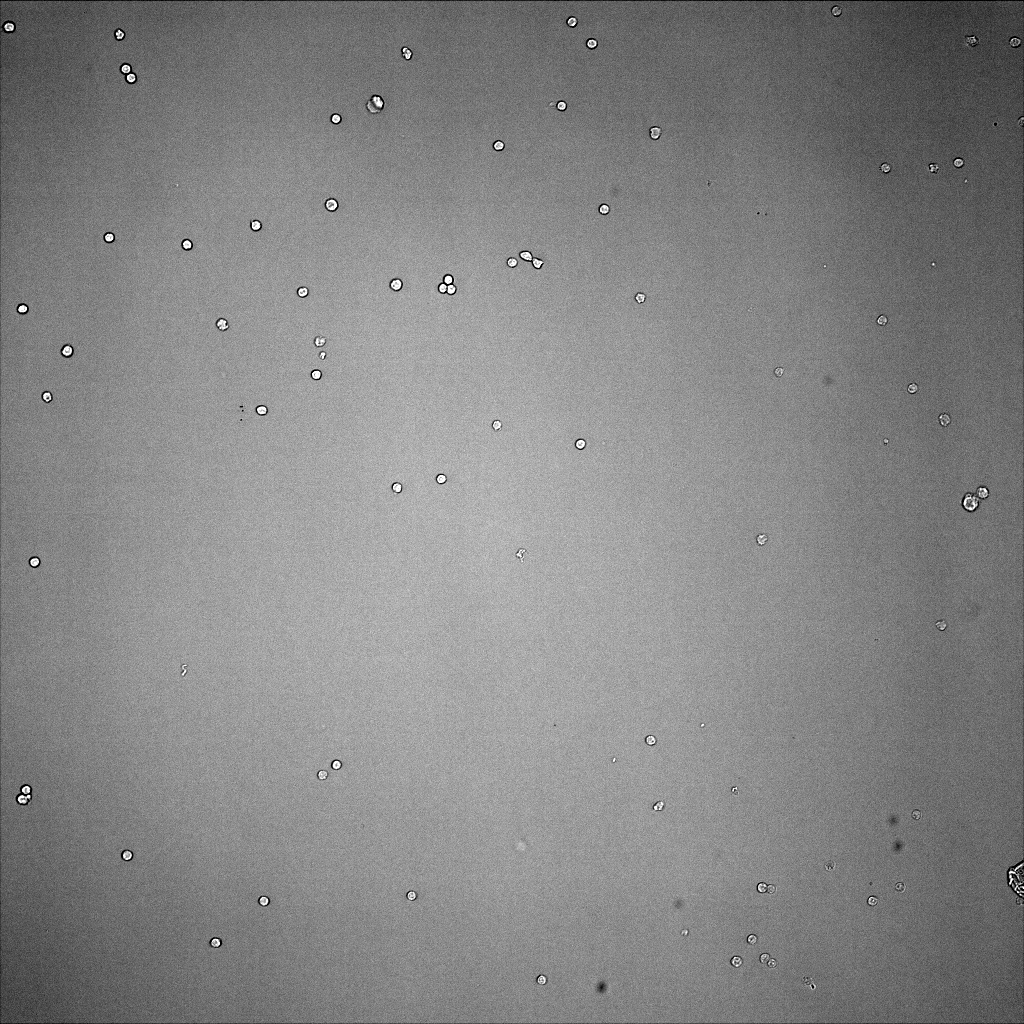

Supplement: Supplementary file 18 [file msb0011-0783-sd18.zip › Snap-41_c1_ORG.png]

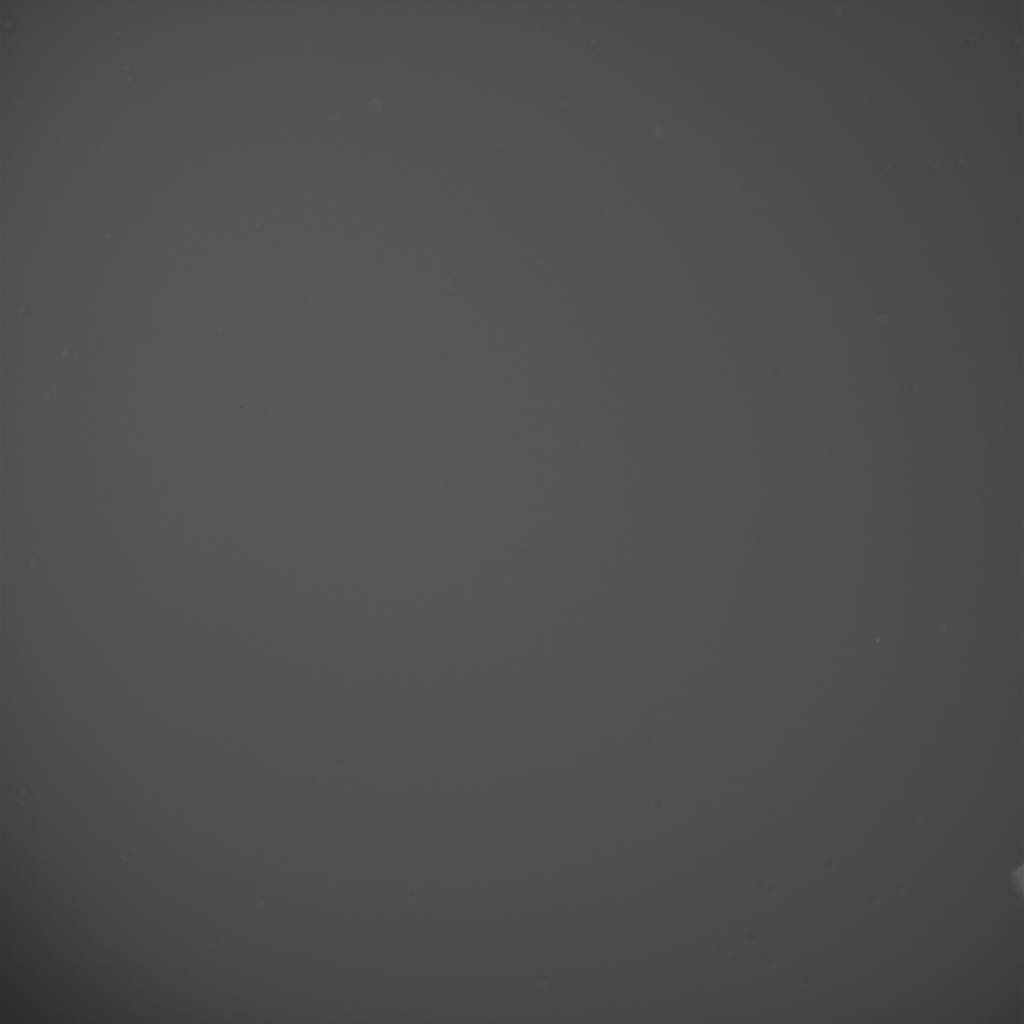

Supplement: Supplementary file 18 [file msb0011-0783-sd18.zip › Snap-41_c2_ORG.png]

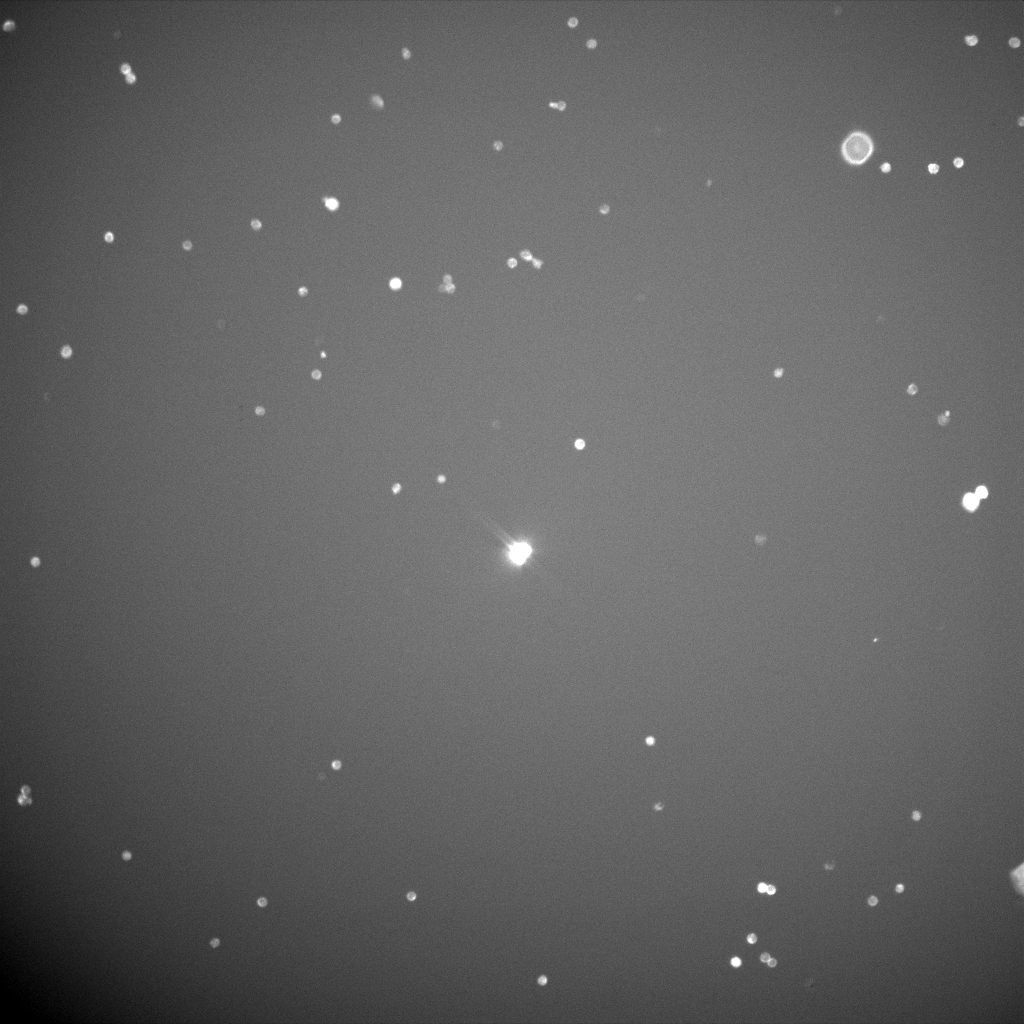

Supplement: Supplementary file 18 [file msb0011-0783-sd18.zip › Snap-41_c3_ORG.png]

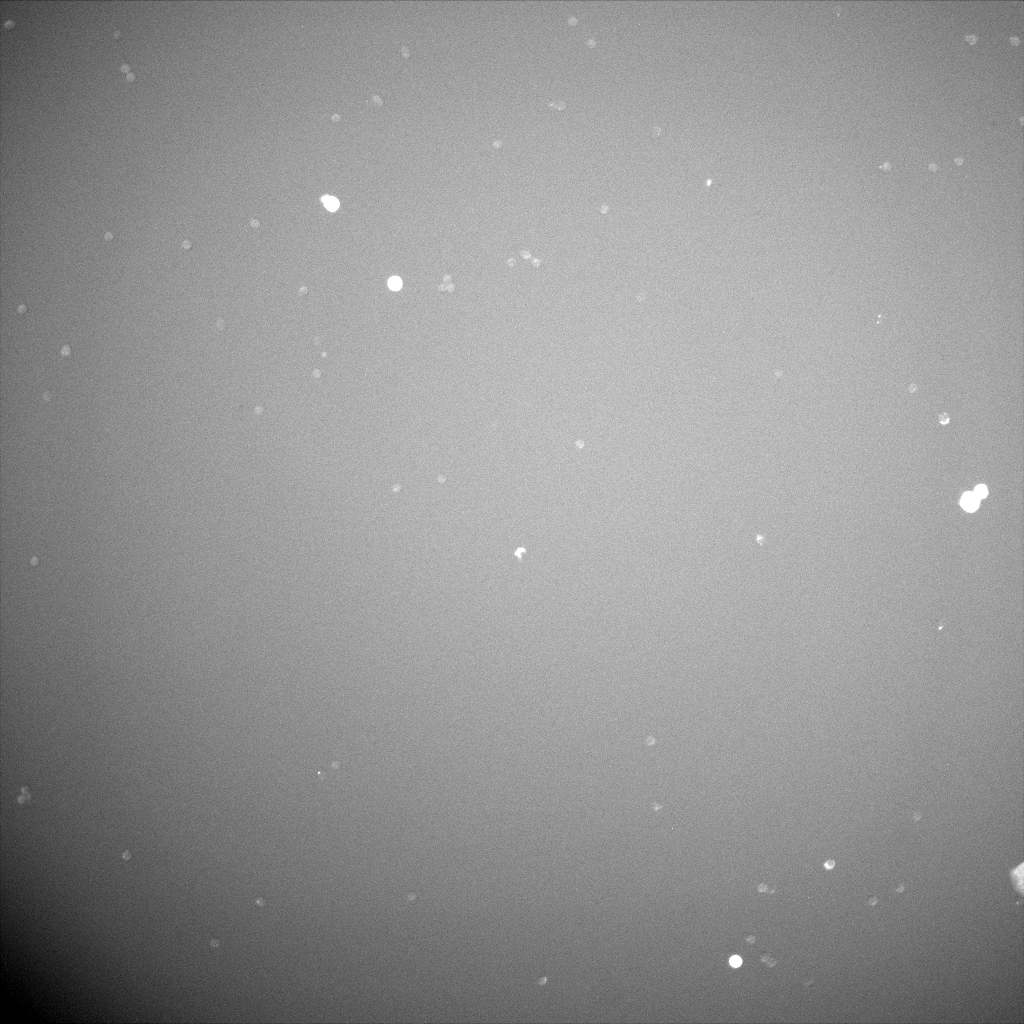

Supplement: Supplementary file 18 [file msb0011-0783-sd18.zip › Snap-41_c4_ORG.png]

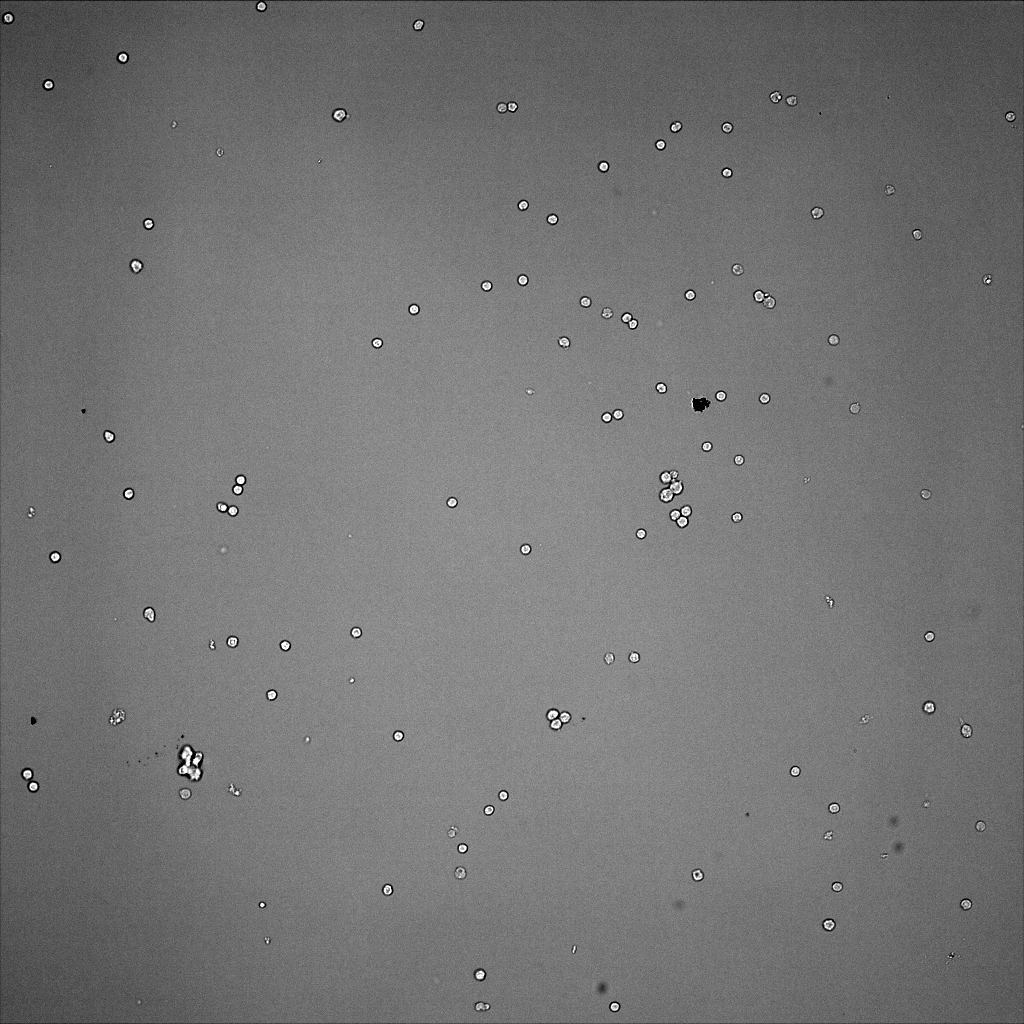

Supplement: Supplementary file 18 [file msb0011-0783-sd18.zip › Snap-42_c1_ORG.png]

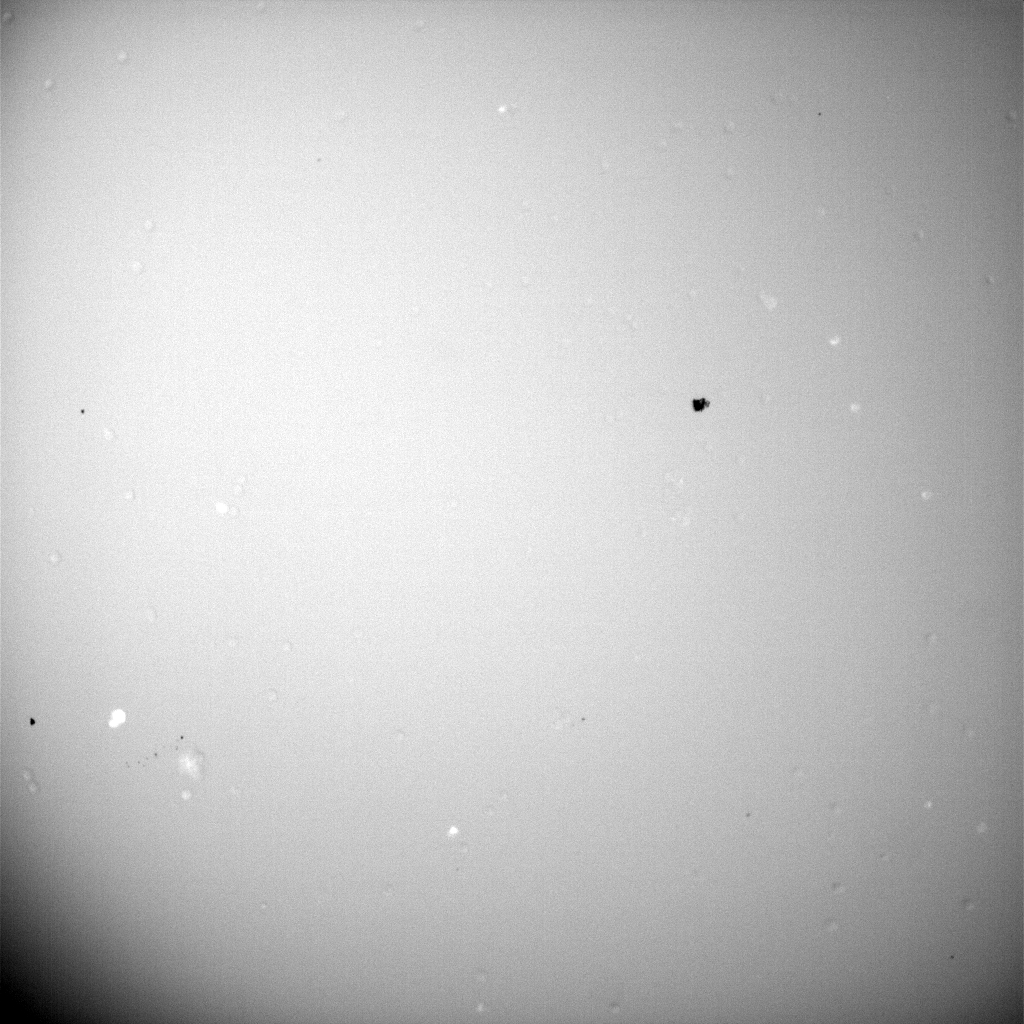

Supplement: Supplementary file 18 [file msb0011-0783-sd18.zip › Snap-42_c2_ORG.png]

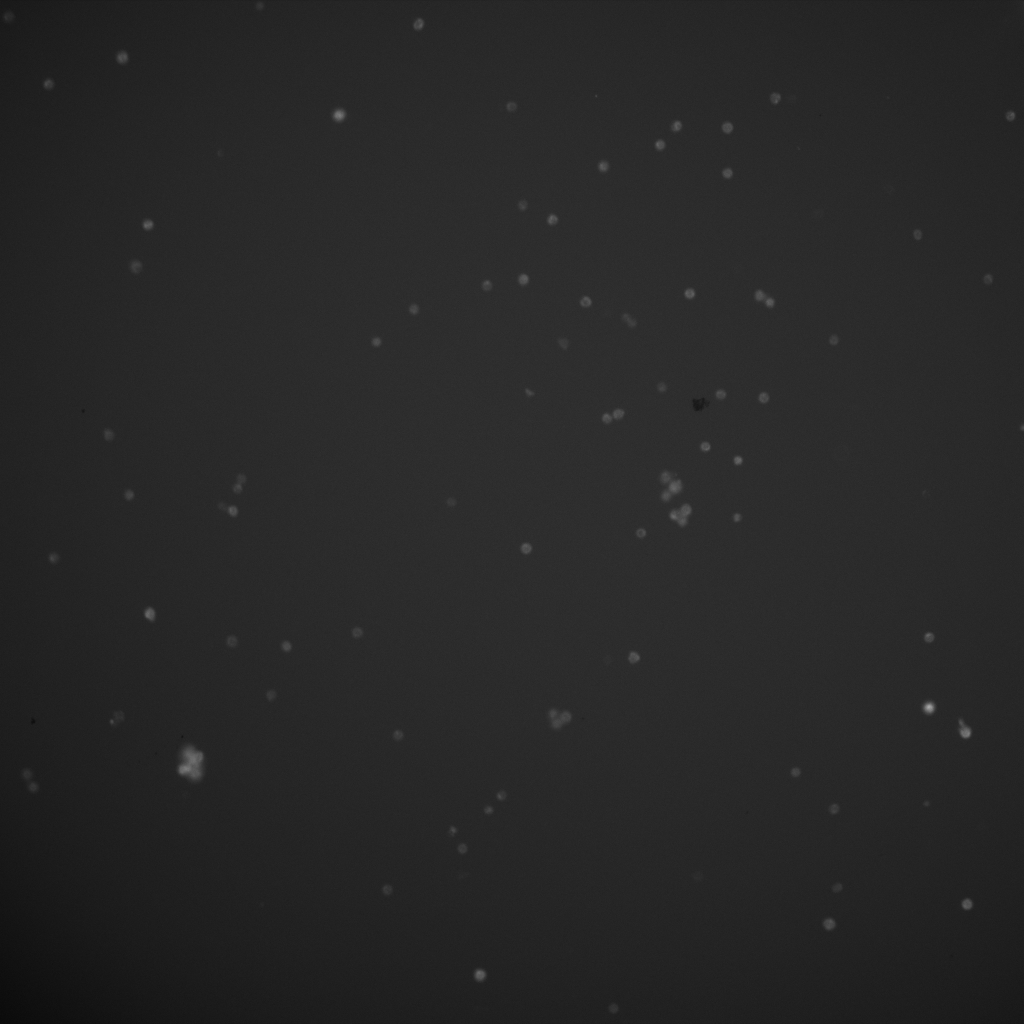

Supplement: Supplementary file 18 [file msb0011-0783-sd18.zip › Snap-42_c3_ORG.png]

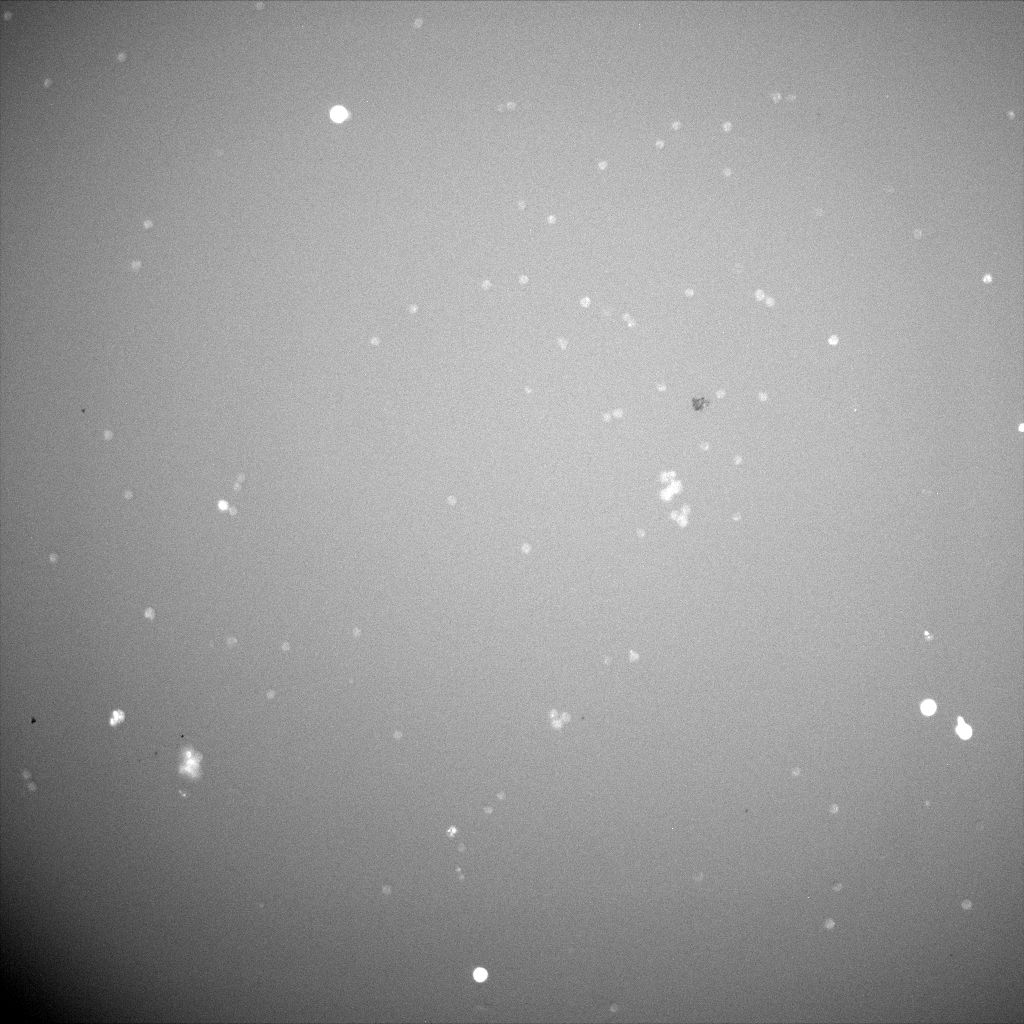

Supplement: Supplementary file 18 [file msb0011-0783-sd18.zip › Snap-42_c4_ORG.png]
