# Supplementary material for: A multi-scale approach reveals that NF-κB cRel enforces a B-cell decision to divide
Source: Mol Syst Biol. 2015 Feb 13;11(2):783. doi: 10.15252/msb.20145554 (PMC4358656; doi:10.15252/msb.20145554)
Supplement: Supplementary file 9 [file msb0011-0783-sd9.docx]

**Supplementary Tables**

Maxim N. Shokhirev, Jonathan Almaden, Jeremy Davis-Turak, Harry A. Birnbaum, Theresa M. Russell, Jesse A.D. Vargas, Alexander Hoffmann. **“A multi-scale approach reveals that NFκB cRel enforces a B-cell decision to divide**.”

Table of Contents

[*Table S1. Correlations between cell transcriptomes. 2*](#_Toc402119869)

[*Table S2. NFκB target genes that are transcriptional regulators. 3*](#_Toc402119870)

[*Table S3. Integrated B-cell model species. 4*](#_Toc402119871)

[*Table S4. Integrated B-cell model rate constants. 5*](#_Toc402119872)

[*Table S5. Integrated B-cell model flux reactions. 15*](#_Toc402119873)

[*Table S6. Integrated B-cell model reactions. 19*](#_Toc402119874)

[*Table S7. Other simulation parameters 22*](#_Toc402119875)

[*Table S8. List of constants in new model reactions 23*](#_Toc402119876)

[*Table S9. Parameterization of free parameters and sensitivity analysis 24*](#_Toc402119877)

[*Table S10. Population response features being fitted during model parameterization 25*](#_Toc402119878)

[*Table S11. Evaluating model fitting to experimental WT, cRel KO, low CpG, and Rapamycin treated datasets. 26*](#_Toc402119879)

[*References 27*](#_Toc402119880)

# Table S1. Correlations between cell transcriptomes.

|  | Small 1 | Small 2 | Small 3 | Small 4 | Small 5 | Large 1 | Large 2 | Large 3 | Large 4 | Large 5 | Neg. | Bulk + |
| --- | --- | --- | --- | --- | --- | --- | --- | --- | --- | --- | --- | --- |
| Small 1 | 1.00 | 0.77 | 0.58 | 0.63 | 0.63 | 0.65 | 0.40 | 0.65 | 0.67 | 0.59 | 0.27 | 0.61 |
| Small 2 | 0.77 | 1.00 | 0.70 | 0.64 | 0.65 | 0.65 | 0.45 | 0.71 | 0.70 | 0.66 | 0.33 | 0.65 |
| Small 3 | 0.58 | 0.70 | 1.00 | 0.59 | 0.73 | 0.67 | 0.49 | 0.63 | 0.72 | 0.74 | 0.34 | 0.62 |
| Small 4 | 0.63 | 0.64 | 0.59 | 1.00 | 0.73 | 0.83 | 0.45 | 0.81 | 0.79 | 0.61 | 0.41 | 0.82 |
| Small 5 | 0.63 | 0.65 | 0.73 | 0.73 | 1.00 | 0.82 | 0.49 | 0.76 | 0.87 | 0.77 | 0.39 | 0.73 |
| Large 1 | 0.65 | 0.65 | 0.67 | 0.83 | 0.82 | 1.00 | 0.46 | 0.82 | 0.84 | 0.66 | 0.37 | 0.81 |
| Large 2 | 0.40 | 0.45 | 0.49 | 0.45 | 0.49 | 0.46 | 1.00 | 0.44 | 0.51 | 0.51 | 0.30 | 0.44 |
| Large 3 | 0.65 | 0.71 | 0.63 | 0.81 | 0.76 | 0.82 | 0.44 | 1.00 | 0.83 | 0.62 | 0.40 | 0.80 |
| Large 4 | 0.67 | 0.70 | 0.72 | 0.79 | 0.87 | 0.84 | 0.51 | 0.83 | 1.00 | 0.71 | 0.43 | 0.75 |
| Large 5 | 0.59 | 0.66 | 0.74 | 0.61 | 0.77 | 0.66 | 0.51 | 0.62 | 0.71 | 1.00 | 0.35 | 0.64 |
| Negative | 0.27 | 0.33 | 0.34 | 0.41 | 0.39 | 0.37 | 0.30 | 0.40 | 0.43 | 0.35 | 1.00 | 0.37 |
| Bulk + | 0.61 | 0.65 | 0.62 | 0.82 | 0.73 | 0.81 | 0.44 | 0.80 | 0.75 | 0.64 | 0.37 | 1.00 |

Transcriptomes of five large and five small cells were sequenced and aligned to the mouse genome. Gene counts were normalized to RNA spike in pseudo-counts and a constant positive count of 100 was added to all genes to represent experimental sensitivity as seen in the variability in spikein counts between samples (see methods). Colors represent degree of correlation (Pearson’s r). The second large cell was removed from further analysis due to poor correlation with all other cells (similar to negative).

# Table S2. NFκB target genes that are transcriptional regulators.

| **Name** | **Description** | **Name** | **Description** |
| --- | --- | --- | --- |
| Androgen receptor | Hormone receptor | IRF-2 | Interferon regulatory factor-2 |
| Bcl-3 | Coactivator for NF-kB p50 and p52 | IRF-4 | Interferon regulatory factor-4 |
| BMI-1 | Polycomb chromatin modifier | IRF-7 | Interferon regulatory factor -7 |
| CDX1 | Homeobox protein | junB | Proto-oncogene |
| c-fos | Proto-oncogene | Lef1 | Transcription factor in Wnt/b-catenin pathway |
| c-myb | Proto-oncogene | LZIP | Leukocyte cell mobility |
| c-myc | Proto-oncogene | NLRP2 | NF-kB pathway inhibitor |
| c-rel | Proto-oncogene | NURR1 | Nuclear orphan receptor |
| C/EBPdelta | Transcription factor | Osterix | Bone transcription factor |
| DC-SCRIPT | Dendritic cell zinc finger protein | p53 | TF, Tumor suppressor |
| Dmp1 | Myb-like transcription factor | Progesterone receptor | Transcription factor |
| E2F3a | Cell cycle regulator | PU.1 | Transcription factor |
| Elf3 | Ets family transcription factor | relb | Transcription factor |
| ELYS | Embryonic large molecule derived from yolk sac | Snail | Transcription factor |
| Egr-1 | Mitogen-induced early response gene; zinc finger | Sox9 | Transcription factor |
| ETR101 | TPA-inducible, Jun-like transcription factor | Stat5a | Transcription factor |
| Gata-3 | T-cell differentiation Factor | Tfec | Transcription factor |
| Glucocorticoid receptor | Promoter 1B of the GR | Twist | Transcription repressor |
| HIF-1alpha | Hypoxia-inducible factor | WT1 | Zinc finger transcription factor |
| HOXA9 | Homeobox protein | YY1 | Transcription factor |
| IRF-1 | Interferon regulatory factor-1 |  |  |

A list was adapted from ([Gilmore, 2014](#_ENREF_5)). Transcription factors with motifs upregulated in large (red) or small (green) cells have been highlighted.

# Table S3. Integrated B-cell model species.

| # | **Species** | **x_0_** | **#** | **Species** | **x_0_** | **#** | **Species** | **x_0_** | **#** | **Species** | **x_0_** |
| --- | --- | --- | --- | --- | --- | --- | --- | --- | --- | --- | --- |
| **1** | tIkBa | 0 | **37** | IkBbA50n | 0 | **73** | pC3 | 10000 | **109** | CytoC | 0 |
| **2** | IkBa | 0 | **38** | IkBeA50 | 0 | **74** | C8-pC3 | 0 | **110** | Apaf | 100000 |
| **3** | IkBan | 0 | **39** | IkBeA50n | 0 | **75** | C3 | 0 | **111** | Apaf-CytoC | 0 |
| **4** | tIkBb | 0 | **40** | IkBdA50 | 0 | **76** | pC6 | 10000 | **112** | act_Apaf | 0 |
| **5** | IkBb | 0 | **41** | IkBdA50n | 0 | **77** | C3-pC6 | 0 | **113** | pC9 | 100000 |
| **6** | IkBbn | 0 | **42** | 5050 | 0 | **78** | C6 | 0 | **114** | Apop | 0 |
| **7** | tIkBe | 0 | **43** | 5050n | 0 | **79** | C6-pC8 | 0 | **115** | Apop-pC3 | 0 |
| **8** | IkBe | 0 | **44** | IkBa5050 | 0 | **80** | XIAP | 100000 | **116** | cSmac | 0 |
| **9** | IkBen | 0 | **45** | IkBa5050n | 0 | **81** | XIAP-C3 | 0 | **117** | Apop-XIAP | 0 |
| **10** | tIkBd | 0 | **46** | IkBb5050 | 0 | **82** | PARP | 1000000 | **118** | cSmac-XIAP | 0 |
| **11** | IkBd | 0 | **47** | IkBb5050n | 0 | **83** | C3-PARP | 0 | **119** | C3_Ub | 0 |
| **12** | IkBdn | 0 | **48** | IkBe5050 | 0 | **84** | CPARP | 0 | **120** | CycA | 0.014254 |
| **13** | tRelA | 0 | **49** | IkBe5050n | 0 | **85** | Bid | 60000 | **121** | CycB | 2.228995 |
| **14** | RelA | 0 | **50** | IkBd5050 | 0 | **86** | C8-Bid | 0 | **122** | CycD | 0.411217 |
| **15** | RelAn | 0 | **51** | IkBd5050n | 0 | **87** | tBid | 0 | **123** | CycE | 0.170235 |
| **16** | tP50 | 0 | **52** | C50 | 0 | **88** | Mcl1 | 20000 | **124** | tCycD | 0 |
| **17** | P50 | 0 | **53** | C50n | 0 | **89** | Mcl1-tBid | 0 | **125** | tBclXL | 1000 |
| **18** | P50n | 0 | **54** | IkBaC50 | 0 | **90** | Bax | 80000 | **126** | Cdh1 | 1 |
| **19** | tcRel | 0 | **55** | IkBaC50n | 0 | **91** | tBid-Bax | 0 | **127** | CA | 0.001343 |
| **20** | cRel | 0 | **56** | IkBbC50 | 0 | **92** | act_Bax | 0 | **128** | CD | 0.039049 |
| **21** | cReln | 0 | **57** | IkBbC50n | 0 | **93** | Baxm | 0 | **129** | CDc20 | 0.610581 |
| **22** | AA | 0 | **58** | IkBeC50 | 0 | **94** | BclXL | 30000 | **130** | CDc20T | 2.967532 |
| **23** | AAn | 0 | **59** | IkBeC50n | 0 | **95** | Baxm-Bcl2 | 0 | **131** | CE | 0.016231 |
| **24** | IkBaAA | 0 | **60** | IkBdC50 | 0 | **96** | Bax2 | 0 | **132** | GM | 0.747611 |
| **25** | IkBaAAn | 0 | **61** | IkBdC50n | 0 | **97** | Bax2-Bcl2 | 0 | **133** | IEP | 0.791894 |
| **26** | IkBbAA | 0 | **62** | L | 0 | **98** | Bax4 | 0 | **134** | Mass | 0.930762 |
| **27** | IkBaAAn | 0 | **63** | R | 1000 | **99** | Bax4-Bcl2 | 0 | **135** | p27 | 0.024804 |
| **28** | IkBeAA | 0 | **64** | L-R | 0 | **100** | M | 500000 | **136** | PPX | 1 |
| **29** | IkBeAAn | 0 | **65** | DISC | 0 | **101** | Bax4-M | 0 | **137** | pp-Rb | 9.859403 |
| **30** | IkBdAA | 0 | **66** | flip | 2000 | **102** | AMito | 0 | **138** | E2F | 1.2212 |
| **31** | IkBdAAn | 0 | **67** | flip-DISC | 0 | **103** | mCytoC | 500000 | **139** | p-E2F | 3.639173 |
| **32** | A50 | 0 | **68** | pC8 | 10e4 | **104** | AMito-mCytoC | 0 | **140** | Rb | 0.000975 |
| **33** | A50n | 0 | **69** | DISC-pC8 | 0 | **105** | aCytoC | 0 | **141** | E2F-Rb | 0.035082 |
| **34** | IkBaA50 | 0 | **70** | C8 | 0 | **106** | mSmac | 100000 | **142** | p-E2F-Rb | 0.104543 |
| **35** | IkBaA50n | 0 | **71** | Bar | 1000 | **107** | AMito-mSmac | 0 | **143** | Myc | 0 |
| **36** | IkBbA50 | 0 | **72** | Bar-C8 | 0 | **108** | aSmac | 0 | **144** | tMyc | 0 |

Note: active IKK (IKK*) species is modeled as a time-dependent input function described in **Table S6**.

Prior to simulation, the model was equilibrated starting from initial concentrations, x_0_, with basal IKK signaling and no death signal (L=0) for a period of 24 h. Due to relatively high initial cyclin levels (see Conradie, 2010), the equilibration was repeated until the cell stopped dividing ([Mass] and [GM] was halved as usual) followed by a 24 h period of quiescent equilibration.

# Table S4. Integrated B-cell model rate constants.

| **#** | **Parameter** | **Description** | **Value** | | **Units** | | **Justification** |  |
| --- | --- | --- | --- | --- | --- | --- | --- | --- |
| **1** | **ki(1,α)** | →tIkbα | 0.0009 | | nM min^-1^ | | Parameter value chosen to fit mRNA and protein Expression profiles as measure by RNase Protection (RPA) and Western blot assays, reformulated from ([Werner et al, 2008](#_ENREF_16))to fit a Hill function where Hill coefficient = 1.1 |  |
| **2** | **ki(2,α)** | tIkbα→ | 0.0438 | | min^-1^ | | ([Shih et al, 2009](#_ENREF_14)) |  |
| **3** | **ki(3,α)** | tIkbα→Ikbα | 12 | | Proteins/ mRNA min^-1^ | | Derived from the elongation rate of the ribosome and corrected for th nucleotide spacing between adjacent ribosomes on the same transcript: 30 nt sec^-1^/150 nt = 12 min^-1^ |  |
| **4** | **ki(4,α)** | Ikbα→ | 0.12 | | min^-1^ | | As in #2 |  |
| **5** | **ki(5,α)** | nIkbα→ | 0.12 | | min^-1^ | | As in #2 |  |
| **6** | **ki(6,α)** | Ikbα→ nIkbα | 0.06 | | min^-1^ | | As in #2 |  |
| **7** | **ki(7,α)** | nIkbα→ Ikbα | 0.012 | | min^-1^ | | As in #2 |  |
| **8** | **ki(8,α)** | transcription delay | 0 | | min | | As in #1 |  |
| **9** | **ki(9,α)** | Hill c for inducible txn. | 1.1 | |  | | ([Alves et al, 2014](#_ENREF_2)) |  |
| **10** | **ki(10,α)** | Hill Kd | 150 | | nM | | As in #1 |  |
| **11** | **ki(11,α)** | Ikbα+IKK→IKK | 0.00135 | | nM^-1^ min^-1^ | | Based on measured IkB degradation time courses given numerical input |  |
| **12** | **ki(12,α)** | Ikbα:NFκB+IKK→IKK | 0.00135 | | nM^-1^ min^-1^ | | As in #11 |  |
| **13** | **ki(13,α)** | Ikbα:NFκB→ | 0.00024 | | min^-1^ | | Based on estimated 48 h half-life |  |
| **14** | **ki(14,α)** | nIkbα:NFκB→ | 0.00024 | | min^-1^ | | As in #13 |  |
| **15** | **ki(1,β)** | →tIkbβ | 0.0006 | | nM min^-1^ | | As in #1 |  |
| **16** | **ki(2,β)** | tIkbβ→ | 0.00288 | | min^-1^ | | As in #2 |  |
| **17** | **ki(3,β)** | tIkbβ→Ikbβ | 12 | | Proteins/ mRNA min^-1^ | | As in #3 |  |
| **18** | **ki(4,β)** | Ikbβ→ | 0.12 | | min^-1^ | | As in #2 |  |
| **19** | **ki(5,β)** | nIkbβ→ | 0.12 | | min^-1^ | | As in #2 |  |
| **20** | **ki(6,β)** | Ikbβ→ nIkbβ | 0.009 | | min^-1^ | | As in #2 |  |
| **21** | **ki(7,β)** | nIkbβ→ Ikbβ | 0.012 | | min^-1^ | | As in #2 |  |
| **22** | **ki(8,β)** | transcription delay | 45 | | min | | As in #1 |  |
| **23** | **ki(9,β)** | Hill c for inducible txn. | 1.1 | |  | | As in #9 |  |
| **24** | **ki(10,β)** | Hill Kd | 150 | | nM | | As in #1 |  |
| **25** | **ki(11,β)** | Ikbβ+IKK→IKK | 0.00045 | | nM^-1^ min^-1^ | | As in #11 |  |
| **26** | **ki(12,β)** | Ikbβ:NFκB+IKK→IKK | 0.00045 | | nM^-1^ min^-1^ | | As in #11 |  |
| **27** | **ki(13,β)** | Ikbβ:NFκB→ | 0.00024 | | min^-1^ | | As in #13 |  |
| **28** | **ki(14,β)** | nIkbβ:NFκB→ | 0.00024 | | min^-1^ | | As in #13 |  |
| **29** | **ki(1,ε)** | →tIkbε | 7.20E-05 | | nM min^-1^ | | As in #1 |  |
| **30** | **ki(2,ε)** | tIkbε→ | 0.00384 | | min^-1^ | | As in #2 |  |
| **31** | **ki(3,ε)** | tIkbε→Ikbε | 12 | | Proteins/ mRNA min^-1^ | | As in #3 |  |
| **32** | **ki(4,ε)** | Ikbε→ | 0.01155 | | min^-1^ | | Schuerenberg and Hoffmann, in preparation |  |
| **33** | **ki(5,ε)** | nIkbε→ | 0.01155 | | min^-1^ | | As in #32 |  |
| **34** | **ki(6,ε)** | Ikbε→ nIkbε | 0.045 | | min^-1^ | | As in #2 |  |
| **35** | **ki(7,ε)** | nIkbε→ Ikbε | 0.012 | | min^-1^ | | As in #2 |  |
| **36** | **ki(8,ε)** | transcription delay | 45 | | min | | As in #1 |  |
| **37** | **ki(9,ε)** | Hill c for inducible txn. | 1.1 | |  | | As in #9 |  |
| **38** | **ki(10,ε)** | Hill Kd | 150 | | nM | | As in #1 |  |
| **39** | **ki(11,ε)** | Ikbε+IKK→IKK | 0.0003375 | | nM^-1^ min^-1^ | | As in #11 |  |
| **40** | **ki(12,ε)** | Ikbε:NFκB+IKK→IKK | 0.0003375 | | nM^-1^ min^-1^ | | As in #11 |  |
| **41** | **ki(13,ε)** | Ikbε:NFκB→ | 0.00024 | | min^-1^ | | As in #13 |  |
| **#** | **Name** | **Description** | **Value** | | **Units** | | **Justification** |  |
| **42** | **ki(14,ε)** | nIkbε:NFκB→ | 0.00024 | | min^-1^ | | As in #13 |  |
| **43** | **km(1,RelA)** | →tRelA | 7.20E-05 | | nM min^-1^ | | As in #1 |  |
| **44** | **km(2,RelA)** | tRelA→ | 0.00288 | | min^-1^ | | As in #2 |  |
| **45** | **km(3,RelA)** | tRelA→RelA | 12 | | Proteins/ mRNA min | | As in #3 |  |
| **46** | **km(4,RelA)** | RelA→ | 0.0231 | | min^-1^ | | Based on estimated 30 min half-life of NFκB monomers |  |
| **47** | **km(5,RelA)** | nRelA→ | 0.0231 | | min^-1^ | | As in #46 |  |
| **48** | **km(6,RelA)** | transcription delay | 60 | | min | | Based on estimated 1 h delay from RPA experiments |  |
| **49** | **km(7,RelA)** | Hill c for inducible txn. | 1 | |  | | Assumed to be similar to #9 |  |
| **50** | **km(8,RelA)** | Hill Kd | 150 | | nM | | As in #1 |  |
| **51** | **km(1,p50)** | →tp50 | 4.68E-05 | | nM min^-1^ | | Reduced to account for induced expression and a basal steady state of 4 nM RelA:p50 and 2 nM cRel:p50 in the nucleus. |  |
| **52** | **km(2,p50)** | tp50→ | 0.00288 | | min^-1^ | | As in #2 |  |
| **53** | **km(3,p50)** | tp50→p50 | 12 | | Proteins/ mRNA min | | As in #3 |  |
| **54** | **km(4,p50)** | p50→ | 0.0231 | | min^-1^ | | As in #46 |  |
| **55** | **km(5,p50)** | np50→ | 0.0231 | | min^-1^ | | As in #46 |  |
| **56** | **km(6,p50)** | transcription delay | 60 | | min | | As in #48 |  |
| **57** | **km(7,p50)** | Hill c for inducible txn. | 1 | |  | | As in #49 |  |
| **58** | **km(8,p50)** | Hill Kd | 150 | | nM | | As in #1 |  |
| **59** | **km(1,cRel)** | →tcRel | 4.68E-05 | | nM min^-1^ | | As in #51 |  |
| **60** | **km(2,cRel)** | tcRel→ | 0.00288 | | min^-1^ | | As in #2 |  |
| **61** | **km(3,cRel)** | tcRel→cRel | 12 | | Proteins/ mRNA min | | As in #3 |  |
| **62** | **km(4,cRel)** | cRel→ | 0.0231 | | min^-1^ | | As in #46 |  |
| **63** | **km(5,cRel)** | ncRel→ | 0.0231 | | min^-1^ | | As in #46 |  |
| **64** | **km(6,cRel)** | transcription delay | 60 | | min | | As in #48 |  |
| **65** | **km(7,cRel)** | Hill c for inducible txn. | 1 | |  | | As in #49 |  |
| **66** | **km(8,cRel)** | Hill Kd | 150 | | nM | | As in #1 |  |
| **67** | **kd(1,AA)** | RelA+RelA→AA | 0.0006 | | nM^-1^ min^-1^ | | Tsui et al. in press |  |
| **68** | **kd(2,AA)** | nRelA+nRelA→nAA | 0.0006 | | nM^-1^ min^-1^ | | As in #67 |  |
| **69** | **kd(3,AA)** | AA→ RelA+RelA | 0.48 | | min^-1^ | | As in #67 |  |
| **70** | **kd(4,AA)** | nAA→nRelA+nRelA | 0.048 | | min^-1^ | | As in #67 |  |
| **71** | **kd(5,AA)** | AA→nAA | 5.4 | | min^-1^ | | As in #2 |  |
| **72** | **kd(6,AA)** | nAA→AA | 0.0048 | | min^-1^ | | As in #2 |  |
| **73** | **kd(7,AA)** | AA→ | 0.00024 | | min^-1^ | | Based on estimated 48 hour half-life |  |
| **74** | **kd(8,AA)** | nAA→ | 0.00024 | | min^-1^ | | As in #73 |  |
| **75** | **kd(9,AA)** | IkB:AA→IkB | 0.00024 | | min^-1^ | | See #13 |  |
| **76** | **kd(10,AA)** | nIkB:AA→nIkB | 0.00024 | | min^-1^ | | See #13 |  |
| **77** | **kd(1,A50)** | RelA+p50→A50 | 0.001896 | | nM^-1^ min^-1^ | | As in #67 |  |
| **78** | **kd(2,A50)** | nRelA+np50→nA50 | 0.001896 | | nM^-1^ min^-1^ | | As in #67 |  |
| **79** | **kd(3,A50)** | A50→ RelA+p50 | 0.01896 | | min^-1^ | | As in #67 |  |
| **80** | **kd(4,A50)** | nA50→nRelA+np50 | 0.001896 | | min^-1^ | | As in #67 |  |
| **81** | **kd(5,A50)** | A50→nA50 | 5.4 | | min^-1^ | | As in #2 |  |
| **82** | **kd(6,A50)** | nA50→A50 | 0.0048 | | min^-1^ | | As in #2 |  |
| **83** | **kd(7,A50)** | A50→ | 0.00024 | | min^-1^ | | As in #73 |  |
| **84** | **kd(8,A50)** | nA50→ | 0.00024 | | min^-1^ | | As in #73 |  |
| **85** | **kd(9,A50)** | IkB:A50→IkB | 0.00024 | | min^-1^ | | See #13 |  |
| **86** | **kd(10,A50)** | nIkB:A50→nIkB | 0.00024 | | min^-1^ | | See #13 |  |
| **87** | **kd(1,5050)** | p50+p50→A50 | 0.0018 | | nM^-1^ min^-1^ | | As in #67 |  |
| **88** | **kd(2,5050)** | np50+np50→nA50 | 0.0018 | | nM^-1^ min^-1^ | | As in #67 |  |
| **#** | **Name** | **Description** | **Value** | | **Units** | | **Justification** |  |
| **89** | **kd(3,5050)** | 5050→ p50+p50 | 0.054 | | min^-1^ | | As in #67 |  |
| **90** | **kd(4,5050)** | N5050→np50+np50 | 0.0054 | | min^-1^ | | As in #67 |  |
| **91** | **kd(5,5050)** | 5050→n5050 | 5.4 | | min^-1^ | | As in #2 |  |
| **92** | **kd(6,5050)** | N5050→5050 | 0.0048 | | min^-1^ | | As in #2 |  |
| **93** | **kd(7,5050)** | 5050→ | 0.00024 | | min^-1^ | | As in #73 |  |
| **94** | **kd(8,5050)** | N5050→ | 0.00024 | | min^-1^ | | As in #73 |  |
| **95** | **kd(9,5050)** | IkB:5050→IkB | 0.00024 | | min^-1^ | | See #13 |  |
| **96** | **kd(10,5050)** | nIkB:5050→nIkB | 0.00024 | | min^-1^ | | See #13 |  |
| **97** | **kd(1,C50)** | cRel+p50→A50 | 0.0018 | | nM^-1^ min^-1^ | | As in #67 |  |
| **98** | **kd(2,C50)** | ncRel +np50→nA50 | 0.0018 | | nM^-1^ min^-1^ | | As in #67 |  |
| **99** | **kd(3,C50)** | C50→ cRel +p50 | 0.054 | | min^-1^ | | As in #67 |  |
| **100** | **kd(4,C50)** | nC50→ncRel+np50 | 0.0054 | | min^-1^ | | As in #67 |  |
| **101** | **kd(5,C50)** | C50→nC50 | 5.4 | | min^-1^ | | As in #2 |  |
| **102** | **kd(6,C50)** | nC50→C50 | 0.0048 | | min^-1^ | | As in #2 |  |
| **103** | **kd(7,C50)** | C50→ | 0.00024 | | min^-1^ | | As in #73 |  |
| **104** | **kd(8,C50)** | nC50→ | 0.00024 | | min^-1^ | | As in #73 |  |
| **105** | **kd(9,C50)** | IkB:C50→IkB | 0.00024 | | min^-1^ | | See #13 |  |
| **106** | **kd(10,C50)** | nIkB:C50→nIkB | 0.00024 | | min^-1^ | | See #13 |  |
| **107** | **kdm(AA,A)** | Monomer induction | 0 | |  | | RelA is not induced by NFκB dimers. |  |
| **108** | **kdm(AA,50)** | Monomer induction | 0 | |  | | Set to 0 since RelA homodimer is 1000 fold less than RelA:p50 or cRel:p50 heterodimers in the nucleus. |  |
| **109** | **kdm(AA,C)** | Monomer induction. | 0 | |  | | As in #108 |  |
| **110** | **kdm(A50,A)** | Monomer induction | 0 | |  | | As in #107 |  |
| **111** | **kdm(A50,50)** | Monomer induction | 10 | |  | | Manually calibrated to ensure late-phase signaling of cRel:p50 heterodimer. |  |
| **112** | **kdm(A50,C)** | Monomer induction. | 10 | |  | | As in #111 |  |
| **113** | **kdm(5050,A)** | Monomer induction | 0 | |  | | As in #107, also p50 homodimer does not induce gene expression. |  |
| **114** | **kdm(5050,50)** | Monomer induction | 0 | |  | | As in #113 |  |
| **115** | **kdm(5050,C)** | Monomer induction. | 0 | |  | | As in #113 |  |
| **116** | **kdm(C50,A)** | Monomer induction | 0 | |  | | As in #107 |  |
| **117** | **kdm(C50,50)** | Monomer induction | 10 | |  | | As in #111 |  |
| **118** | **kdm(C50,C)** | Monomer induction. | 10 | |  | | As in #111 |  |
| **119** | **kp(1,AA,α)** | AA+IkBα→AA:IkBα | 0.001344 | | nM^-1^ min^-1^ | | As in #67 |  |
| **120** | **kp(2,AA,α)** | AA:IkBα→ AA+IkBα | 0.001344 | | min^-1^ | | As in #67 |  |
| **121** | **kp (3,AA,α)** | nAA+nIkBα→nAA:IkBα | 2.68E-05 | | nM^-1^ min^-1^ | | As in #67 |  |
| **122** | **kp(4,AA,α)** | nAA:IkBα→ nAA+IkBα | 2.68E-05 | | min^-1^ | | As in #67 |  |
| **123** | **kp(5,AA,α)** | AA:IkBα→ nAA:IkBα | 0.276 | | min^-1^ | | As in #2 |  |
| **124** | **kp(6,AA,α)** | nAA:IkBα→ AA:IkBα | 0.84 | | min^-1^ | | As in #2 |  |
| **125** | **kp(7,AA,α)** | Induction strength | 25 | |  | | As in #1 |  |
| **126** | **kp(1,AA,β)** | AA+IkBβ→AA:IkBβ | 0.3 | | nM^-1^ min^-1^ | | As in #67 |  |
| **127** | **kp(2,AA,β)** | AA:IkBβ→ AA+IkBβ | 0.3 | | min^-1^ | | As in #67 |  |
| **128** | **kp (3,AA,β)** | nAA+nIkββ→nAA:IkBβ | 0.0012 | | nM^-1^ min^-1^ | | As in #67 |  |
| **129** | **kp(4,AA,β)** | nAA:IkBβ→ nAA+IkBβ | 0.0012 | | min^-1^ | | As in #67 |  |
| **130** | **kp(5,AA,β)** | AA:IkBβ→ nAA:IkBβ | 0.0276 | | min^-1^ | | As in #2 |  |
| **131** | **kp(6,AA,β)** | nAA:IkBβ→ AA:IkBβ | 0.42 | | min^-1^ | | As in #2 |  |
| **132** | **kp(7,AA,β)** | Induction strength | 1 | |  | | As in #1 |  |
| **133** | **kp(1,AA,ε)** | AA+IkBε→AA:IkBε | 0.000213 | | nM^-1^ min^-1^ | | As in #67 |  |
| **134** | **kp(2,AA,ε)** | AA:IkBε→ AA+IkBε | 0.000213 | | min^-1^ | | As in #67 |  |
| **135** | **kp (3,AA,ε)** | nAA+nIkβε→nAA:IkBε | 0.01692 | | nM^-1^ min^-1^ | | As in #67 |  |
| **136** | **kp(4,AA,ε)** | nAA:IkBε→ nAA+IkBε | 0.01692 | | min^-1^ | | As in #67 |  |
| **137** | **kp(5,AA,ε)** | AA:IkBε→ nAA:IkBε | 0.138 | | min^-1^ | | As in #2 |  |
| **138** | **kp(6,AA,ε)** | nAA:IkBε→ AA:IkBε | 0.42 | | min^-1^ | | As in #2 |  |
| **#** | **Name** | **Description** | **Value** | | **Units** | | **Justification** |  |
| **139** | **kp(7,AA,ε)** | Induction strength | 25 | |  | | As in #1 |  |
| **140** | **kp(1,A50,α)** | A50+IkBα→A50:IkBα | 0.004806 | | nM^-1^ min^-1^ | | As in #67 |  |
| **141** | **kp(2,A50,α)** | A50:IkBα→ A50+IkBα | 0.004806 | | min^-1^ | | As in #67 |  |
| **142** | **kp (3,A50,α)** | nA50+nIkBα→nA50:IkBα | 0.0006 | | nM^-1^ min^-1^ | | As in #67 |  |
| **143** | **kp(4,A50,α)** | nA50:IkBα→ nA50+IkBα | 0.0006 | | min^-1^ | | As in #67 |  |
| **144** | **kp(5,A50,α)** | A50:IkBα→ nA50:IkBα | 0.276 | | min^-1^ | | As in #2 |  |
| **145** | **kp(6,A50,α)** | nA50:IkBα→ A50:IkBα | 0.84 | | min^-1^ | | As in #2 |  |
| **146** | **kp(7,A50,α)** | Induction strength | 200 | |  | | As in #1 |  |
| **147** | **kp(1,A50,β)** | A50+IkBβ→A50:IkBβ | 0.000213 | | nM^-1^ min^-1^ | | As in #67 |  |
| **148** | **kp(2,A50,β)** | A50:IkBβ→ A50+IkBβ | 0.000213 | | min^-1^ | | As in #67 |  |
| **149** | **kp (3,A50,β)** | nA50+nIkββ→nA50:IkBβ | 0.01692 | | nM^-1^ min^-1^ | | As in #67 |  |
| **150** | **kp(4,A50,β)** | nA50:IkBβ→ nA50+IkBβ | 0.01692 | | min^-1^ | | As in #67 |  |
| **151** | **kp(5,A50,β)** | A50:IkBβ→ nA50:IkBβ | 0.0276 | | min^-1^ | | As in #2 |  |
| **152** | **kp(6,A50,β)** | nA50:IkBβ→ A50:IkBβ | 0.42 | | min^-1^ | | As in #2 |  |
| **153** | **kp(7,A50,β)** | Induction strength | 1 | |  | | As in #1 |  |
| **154** | **kp(1,A50,ε)** | A50+IkBε→A50:IkBε | 0.001344 | | nM^-1^ min^-1^ | | As in #67 |  |
| **155** | **kp(2,A50,ε)** | A50:IkBε→ A50+IkBε | 0.001344 | | min^-1^ | | As in #67 |  |
| **156** | **kp (3,A50,ε)** | nA50+nIkβε→nA50:IkBε | 0.006 | | nM^-1^ min^-1^ | | As in #67 |  |
| **157** | **kp(4,A50,ε)** | nA50:IkBε→ nA50+IkBε | 0.006 | | min^-1^ | | As in #67 |  |
| **158** | **kp(5,A50,ε)** | A50:IkBε→ nA50:IkBε | 0.138 | | min^-1^ | | As in #2 |  |
| **159** | **kp(6,A50,ε)** | nA50:IkBε→ A50:IkBε | 0.42 | | min^-1^ | | As in #2 |  |
| **160** | **kp(7,A50,ε)** | Induction strength | 25 | |  | | As in #1 |  |
| **161** | **kp(1,5050,α)** | 5050+IkBα→5050:IkBα | 0 | | nM^-1^ min^-1^ | | As in #67 |  |
| **162** | **kp(2,5050,α)** | 5050:IkBα→ 5050+IkBα | 0 | | min^-1^ | | As in #67 |  |
| **163** | **kp (3,5050,α)** | n5050+nIkBα→n5050:IkBα | 0 | | nM^-1^ min^-1^ | | As in #67 |  |
| **164** | **kp(4,5050,α)** | n5050:IkBα→ n5050+IkBα | 0 | | min^-1^ | | As in #67 |  |
| **165** | **kp(5,5050,α)** | 5050:IkBα→ n5050:IkBα | 0 | | min^-1^ | | As in #2 |  |
| **166** | **kp(6,5050,α)** | n5050:IkBα→ 5050:IkBα | 0 | | min^-1^ | | As in #2 |  |
| **167** | **kp(7,5050,α)** | Induction strength | 0 | |  | | As in #1 |  |
| **168** | **kp(1,5050,β)** | 5050+IkBβ→5050:IkBβ | 0 | | nM^-1^ min^-1^ | | As in #67 |  |
| **169** | **kp(2,5050,β)** | 5050:IkBβ→ 5050+IkBβ | 0 | | min^-1^ | | As in #67 |  |
| **170** | **kp (3,5050,β)** | n5050+nIkββ→n5050:IkBβ | 0 | | nM^-1^ min^-1^ | | As in #67 |  |
| **171** | **kp(4,5050,β)** | n5050:IkBβ→ 50A50+IkBβ | 0 | | min^-1^ | | As in #67 |  |
| **172** | **kp(5,5050,β)** | 5050:IkBβ→ 50A50:IkBβ | 0 | | min^-1^ | | As in #2 |  |
| **173** | **kp(6,5050,β)** | n5050:IkBβ→ 5050:IkBβ | 0 | | min^-1^ | | As in #2 |  |
| **174** | **kp(7,5050,β)** | Induction strength | 0 | |  | | As in #1 |  |
| **175** | **kp(1,5050,ε)** | 5050+IkBε→5050:IkBε | 0 | | nM^-1^ min^-1^ | | As in #67 |  |
| **176** | **kp(2,5050,ε)** | 5050:IkBε→ 5050+IkBε | 0 | | min^-1^ | | As in #67 |  |
| **177** | **kp (3,5050,ε)** | n5050+nIkβε→n5050:IkBε | 0 | | nM^-1^ min^-1^ | | As in #67 |  |
| **178** | **kp(4,5050,ε)** | n5050:IkBε→ n5050+IkBε | 0 | | min^-1^ | | As in #67 |  |
| **179** | **kp(5,5050,ε)** | 5050:IkBε→ n5050:IkBε | 0 | | min^-1^ | | As in #2 |  |
| **180** | **kp(6,5050,ε)** | n5050:IkBε→ 5050:IkBε | 0 | | min^-1^ | | As in #2 |  |
| **181** | **kp(7,5050,ε)** | Induction strength | 0 | |  | | As in #1 |  |
| **182** | **kp(1,C50,α)** | C50+IkBα→A50:IkBα | 0.003006 | | nM^-1^ min^-1^ | | As in #67 |  |
| **183** | **kp(2,C50,α)** | C50:IkBα→ A50+IkBα | 0.003006 | | min^-1^ | | As in #67 |  |
| **184** | **kp (3,C50,α)** | nC50+nIkBα→nA50:IkBα | 0.0048 | | nM^-1^ min^-1^ | | As in #67 |  |
| **185** | **kp(4,C50,α)** | nC50:IkBα→ nA50+IkBα | 0.0048 | | min^-1^ | | As in #67 |  |
| **186** | **kp(5,C50,α)** | C50:IkBα→ nA50:IkBα | 0.276 | | min^-1^ | | As in #2 |  |
| **187** | **kp(6,C50,α)** | nC50:IkBα→ A50:IkBα | 0.84 | | min^-1^ | | As in #2 |  |
| **188** | **kp(7,C50,α)** | Induction strength | 1 | |  | | As in #1 |  |
| **189** | **kp(1,C50,β)** | C50+IkBβ→C50:IkBβ | 0.000213 | | nM^-1^ min^-1^ | | As in #67 |  |
| **190** | **kp(2,C50,β)** | C50:IkBβ→ C50+IkBβ | 0.000213 | | min^-1^ | | As in #67 |  |
| **191** | **kp (3,C50,β)** | nC50+nIkββ→nC50:IkBβ | 0.01692 | | nM^-1^ min^-1^ | | As in #67 |  |
| **192** | **kp(4,C50,β)** | nC50:IkBβ→ nC50+IkBβ | 0.01692 | | min^-1^ | | As in #67 |  |
| **193** | **kp(5,C50,β)** | C50:IkBβ→ nC50:IkBβ | 0.0276 | | min^-1^ | | As in #2 |  |
| **194** | **kp(6,C50,β)** | nC50:IkBβ→ C50:IkBβ | 0.42 | | min^-1^ | | As in #2 |  |
| **195** | **kp(7,C50,β)** | Induction strength | 1 | |  | | As in #1 |  |
| **196** | **kp(1,C50,ε)** | C50+IkBε→C50:IkBε | 0.001344 | | nM^-1^ min^-1^ | | As in #67 |  |
| **197** | **kp(2,C50,ε)** | C50:IkBε→ C50+IkBε | 0.001344 | | min^-1^ | | As in #67 |  |
| **#** | **Name** | **Description** | **Value** | | **Units** | | **Justification** |  |
| **198** | **kp (3,C50,ε)** | nC50+nIkβε→nC50:IkBε | 2.68E-05 | | nM^-1^ min^-1^ | | As in #67 |  |
| **199** | **kp(4,C50,ε)** | nC50:IkBε→ nC50+IkBε | 2.68E-05 | | min^-1^ | | As in #67 |  |
| **200** | **kp(5,C50,ε)** | C50:IkBε→ nC50:IkBε | 0.138 | | min^-1^ | | As in #2 |  |
| **201** | **kp(6,C50,ε)** | nC50:IkBε→ C50:IkBε | 0.42 | | min^-1^ | | As in #2 |  |
| **202** | **kp(7,C50,ε)** | Induction strength | 250 | |  | | As in #1 |  |
| **203** | **ka(1)** | L+R→L:R | 2.40E-05 | | mol^-1^ min^-1^ | | Constants were derived using py-substitution to achieve similar steady state concentrations of species as described in an extant model of mammalian apoptosis ([Albeck et al, 2008](#_ENREF_1)), while also allowing cells to survive low doses of stimulation. See ([Loriaux et al, 2013](#_ENREF_11)). |  |
| **204** | **ka(2)** | L:R→L+R | 6.00E-05 | | min^-1^ | | As in #203. Note that we kept L low and constant. |  |
| **205** | **ka(3)** | L:R→DISC (R*) | 0.6 | | min^-1^ | | As in #203 |  |
| **206** | **ka(4)** | flip + DISC → flip:DISC | 6.00E-05 | | mol^-1^ min^-1^ | | As in #203 |  |
| **207** | **ka(5)** | flip:DISC → flip + DISC | 0.06 | | min^-1^ | | As in #203 |  |
| **208** | **ka(6)** | pC8 + DISC → DISC:pC8 | 6.00E-06 | | mol^-1^ min^-1^ | | As in #203 |  |
| **209** | **ka(7)** | DISC:pC8 → pC8 + DISC | 0.06 | | min^-1^ | | As in #203 |  |
| **210** | **ka(8)** | DISC:pC8 → C8 + DISC | 60 | | min^-1^ | | As in #203 |  |
| **211** | **ka(9)** | C8 + BAR → BAR:C8 | 6.00E-05 | | mol^-1^ min^-1^ | | As in #203 |  |
| **212** | **ka(10)** | BAR:C8 → C8 + BAR | 0.06 | | min^-1^ | | As in #203 |  |
| **213** | **ka(11)** | pC3 + C8 → pC3:C8 | 6.00E-06 | | mol^-1^ min^-1^ | | As in #203 |  |
| **214** | **ka(12)** | pC3:C8 → pC3 + C8 | 0.06 | | min^-1^ | | As in #203 |  |
| **215** | **ka(13)** | pC3:C8 → C3 + C8 | 60 | | min^-1^ | | As in #203 |  |
| **216** | **ka(14)** | pC6 + C3 → pC6:C3 | 6.00E-06 | | mol^-1^ min^-1^ | | As in #203 |  |
| **217** | **ka(15)** | pC6:C3 → pC6 + C3 | 0.06 | | min^-1^ | | As in #203 |  |
| **218** | **ka(16)** | pC6:C3 → C6 + C3 | 60 | | min^-1^ | | As in #203 |  |
| **219** | **ka(17)** | pC8 + C6 →pC8:C6 | 6.00E-06 | | mol^-1^ min^-1^ | | As in #203 |  |
| **220** | **ka(18)** | pC8:C6 → pC8 + C6 | 0.06 | | min^-1^ | | As in #203 |  |
| **221** | **ka(19)** | pC8:C6 → C8 + C6 | 60 | | min^-1^ | | As in #203 |  |
| **222** | **ka(20)** | XIAP + C3 → XIAP:C3 | 0.00012 | | mol^-1^ min^-1^ | | As in #203 |  |
| **223** | **ka(21)** | XIAP:C3 →XIAP + C3 | 0.06 | | min^-1^ | | As in #203 |  |
| **224** | **ka(22)** | XIAP:C3 →XIAP + C3_U | 6 | | min^-1^ | | As in #203 |  |
| **225** | **ka(23)** | PARP + C3 → PARP:C3 | 6.00E-05 | | mol^-1^ min^-1^ | | As in #203 |  |
| **226** | **ka(24)** | PARP:C3 → PARP + C3 | 0.06 | | min^-1^ | | As in #203 |  |
| **227** | **ka(25)** | PARP:C3 → CPARP + C3 | 1200 | | min^-1^ | | As in #203 |  |
| **228** | **ka(26)** | Bid + C8 → Bid:C8 | 6.00E-06 | | mol^-1^ min^-1^ | | As in #203 |  |
| **229** | **ka(27)** | Bid:C8 → Bid + C8 | 0.06 | | min^-1^ | | As in #203 |  |
| **230** | **ka(28)** | Bid:C8→ tBid + C8 | 60 | | min^-1^ | | As in #203 |  |
| **231** | **ka(29)** | tBid + Bcl2c → tBid:Bcl2c | 6.00E-05 | | mol^-1^ min^-1^ | | As in #203 |  |
| **232** | **ka(30)** | tBid:Bcl2c → tBid + Bcl2c | 0.06 | | min^-1^ | | As in #203 |  |
| **233** | **ka(31)** | Bax + tBid →Bax:tBid | 6.00E-06 | | mol^-1^ min^-1^ | | As in #203 |  |
| **234** | **ka(32)** | Bax:tBid → Bax + tBid | 0.06 | | min^-1^ | | As in #203 |  |
| **235** | **ka(33)** | Bax:tBid → aBax + tBid | 60 | | min^-1^ | | As in #203 |  |
| **236** | **ka(34)** | aBax → MBax | 0.6 | | min^-1^ | | As in #203 |  |
| **237** | **ka(35)** | MBax → aBax | 60 | | min^-1^ | | As in #203 |  |
| **238** | **ka(36)** | MBax + BclXL → MBax:BclXL | 6.00E-05 | | mol^-1^ min^-1^ | | As in #203 |  |
| **239** | **ka(37)** | MBax:BclXL → MBax + BclXL | 0.06 | | min^-1^ | | As in #203 |  |
| **240** | **ka(38)** | MBax + MBax → Bax2 | 6.00E-05 | | min^-1^ | | As in #203 |  |
| **241** | **ka(39)** | Bax2 → MBax + MBax | 0.06 | | min^-1^ | | As in #203 |  |
| **242** | **ka(40)** | Bax2 + Bcl2 → MBax2:Bcl2 | 6.00E-05 | | mol^-1^ min^-1^ | | As in #203 |  |
| **243** | **ka(41)** | MBax2:Bcl2 →Bax2 + Bcl2 | 0.06 | | min^-1^ | | As in #203 |  |
| **244** | **ka(42)** | Bax2 + Bax2→ Bax4 | 6.00E-05 | | mol^-1^ min^-1^ | | As in #203 |  |
| **245** | **ka(43)** | Bax4 → Bax2 + Bax2 | 0.06 | | min^-1^ | | As in #203 |  |
| **246** | **ka(44)** | Bax4 + Bcl2 → MBax4:Bcl2 | 6.00E-05 | | mol^-1^ min^-1^ | | As in #203 |  |
| **247** | **ka(45)** | MBax4:cl2→Bax4 + Bcl2 | 0.06 | | min^-1^ | | As in #203 |  |
| **248** | **ka(46)** | Bax4 + Mito → Bax4:Mito | 6.00E-05 | | mol^-1^ min^-1^ | | As in #203 |  |
| **249** | **ka(47)** | Bax4:Mito →Bax4 + Mito | 0.06 | | min^-1^ | | As in #203 |  |
| **250** | **ka(48)** | Bax4:Mito → AMito | 60 | | min^-1^ | | As in #203 |  |
| **#** | **Name** | **Description** | **Value** | | **Units** | | **Justification** |  |
| **251** | **ka(49)** | AMito + mCytoC → AMito:mCytoC | 0.00012 | | mol^-1^ min^-1^ | | As in #203 |  |
| **252** | **ka(50)** | AMito:mCytoC → AMito + mCytoC | 0.06 | | min^-1^ | | As in #203 |  |
| **253** | **ka(51)** | AMito:mCytoC → AMito + ACytoC | 600 | | min^-1^ | | As in #203 |  |
| **254** | **ka(52)** | AMito + mSMac → AMito:mSmac | 0.00012 | | mol^-1^ min^-1^ | | As in #203 |  |
| **255** | **ka(53)** | AMito:mSMac →AMito + mSmac | 0.06 | | min^-1^ | | As in #203 |  |
| **256** | **ka(54)** | AMito + mSMac→ AMito + ASmac | 600 | | mol^-1^ min^-1^ | | As in #203 |  |
| **257** | **ka(55)** | ACytoC → cCytoC | 60 | | min^-1^ | | As in #203 |  |
| **258** | **ka(56)** | cCytoC → ACytoC | 0.6 | | min^-1^ | | As in #203 |  |
| **259** | **ka(57)** | Apaf + cCytoC → Apaf:cCytoC | 3.00E-05 | | mol^-1^ min^-1^ | | As in #203 |  |
| **260** | **ka(58)** | Apaf:cCytoC → Apaf + cCytoC | 0.06 | | min^-1^ | | As in #203 |  |
| **261** | **ka(59)** | Apaf:cCytoC → Apaf* + cCytoC | 60 | | min^-1^ | | As in #203 |  |
| **262** | **ka(60)** | Apaf* + Procasp9→ Apoptosome | 3.00E-06 | | mol^-1^ min^-1^ | | As in #203 |  |
| **263** | **ka(61)** | Apoptosome →Apaf* + Procasp9 | 0.06 | | min^-1^ | | As in #203 |  |
| **264** | **ka(62)** | Apop + pC3→Apop:pC3 | 3.00E-07 | | mol^-1^ min^-1^ | | As in #203 |  |
| **265** | **ka(63)** | Apop:pC3 → Apop + pC3 | 0.06 | | min^-1^ | | As in #203 |  |
| **266** | **ka(64)** | Apop:pC3→Apop + C3 | 60 | | min^-1^ | | As in #203 |  |
| **267** | **ka(65)** | ASmac →cSmac | 60 | | min^-1^ | | As in #203 |  |
| **268** | **ka(66)** | cSmac →ASmac | 0.6 | | min^-1^ | | As in #203 |  |
| **269** | **ka(67)** | Apop + XIAP→Apop:XIAP | 0.00012 | | mol^-1^ min^-1^ | | As in #203 |  |
| **270** | **ka(68)** | Apop:XIAP → Apop + XIAP | 0.06 | | min^-1^ | | As in #203 |  |
| **271** | **ka(69)** | cSmac + XIAP→ cSmac:XIAP | 0.00042 | | mol^-1^ min^-1^ | | As in #203 |  |
| **272** | **ka(70)** | cSmac:XIAP→cSmac + XIAP | 0.06 | | min^-1^ | | As in #203 |  |
| **273** | **ka(71)** | →R | 2.312439 | | mol/min | | As in #203 |  |
| **274** | **ka(72)** | R → | 0.01155245 | | min^-1^ | | As in #203 |  |
| **275** | **ka(73)** | →flip | 1.15719366 | | mol/min | | As in #203 |  |
| **276** | **ka(74)** | flip→ | 0.01155245 | | min^-1^ | | As in #203 |  |
| **277** | **ka(75)** | flip:DISC→ | 0.01155245 | | min^-1^ | | As in #203 |  |
| **278** | **ka(76)** | →pC8 | 233.519067 | | mol/min | | As in #203 |  |
| **279** | **ka(77)** | pC8→ | 0.01155245 | | min^-1^ | | As in #203 |  |
| **280** | **ka(78)** | → BAR | 21.1716068 | | mol/min | | As in #203 |  |
| **281** | **ka(79)** | BAR→ | 0.03465736 | | min^-1^ | | As in #203 |  |
| **282** | **ka(80)** | BAR:C8→ | 0.11552453 | | min^-1^ | | As in #203 |  |
| **283** | **ka(81)** | →Bid | 477.094542 | | mol/min | | As in #203 |  |
| **284** | **ka(82)** | Bid→ | 0.01155245 | | min^-1^ | | As in #203 |  |
| **285** | **ka(83)** | →Mcl1 | 246.045482 | | mol/min | | As in #203 |  |
| **286** | **ka(84)** | Mcl1→ | 0.01155245 | | min^-1^ | | As in #203 |  |
| **287** | **ka(85)** | tBid:Bcl2c → | 0.01155245 | | min^-1^ | | As in #203 |  |
| **288** | **ka(86)** | →Bax | 1201.64059 | | mol/min | | As in #203 |  |
| **289** | **ka(87)** | Bax → | 0.01155245 | | min^-1^ | | As in #203 |  |
| **290** | **ka(88)** | BclXLt →BclXL | .307 | | min^-1^ | | Value was derived such that the steady-state flux was identical to the original BclXL production flux, which did not explicitly model translation from transcript. |  |
| **291** | **ka(89)** | BclXL→ | 0.01155245 | | min^-1^ | | As in #203 |  |
| **292** | **ka(90)** | Baxm:BclXL → | 0.01155245 | | min^-1^ | | As in #203 |  |
| **293** | **ka(91)** | Bax2:BclXL→ | 0.01155245 | | min^-1^ | | As in #203 |  |
| **294** | **ka(92)** | Bax4:BclXL→ | 0.01155245 | | min^-1^ | | As in #203 |  |
| **295** | **ka(93)** | AMito→Mito | 0.11552453 | | min^-1^ | | As in #203 |  |
| **296** | **ka(94)** | Apaf*→Apaf | 0.11552453 | | min^-1^ | | As in #203 |  |
| **297** | **ka(95)** | →XIAP | 69320.9971 | | mol/min | | As in #203 |  |
| **298** | **ka(96)** | XIAP→ | 0.01155245 | | min^-1^ | | As in #203 |  |
| **299** | **ka(97)** | →mSmac | 69315.2463 | | mol/min | | As in #203 |  |
| **#** | **Name** | **Description** | **Value** | | **Units** | | **Justification** |  |
| **300** | **ka(98)** | mSmac → | 0.01155245 | | min^-1^ | | As in #203 |  |
| **301** | **ka(99)** | cSmac → | 0.01155245 | | min^-1^ | | As in #203 |  |
| **302** | **ka(100)** | cSmac:XIAP→ | 0.01155245 | | min^-1^ | | As in #203 |  |
| **303** | **ka(101)** | → pC3 | 7156.42702 | | mol/min | | As in #203 |  |
| **304** | **ka(102)** | pC3 → | 0.01155245 | | min^-1^ | | As in #203 |  |
| **305** | **ka(103)** | C3_U → | 0.01155245 | | min^-1^ | | As in #203 |  |
| **306** | **ka(104)** | →pC6 | 6942.8207 | | mol/min | | As in #203 |  |
| **307** | **ka(105)** | pC6→ | 0.01155245 | | min^-1^ | | As in #203 |  |
| **308** | **ka(106)** | C6 → | 0.11552453 | | min^-1^ | | As in #203 |  |
| **309** | **ka(107)** | Apop:XIAP → Apop | 0.01155245 | | min^-1^ | | As in #203 |  |
| **310** | **ka(108)** | →Parp | 11571.1993 | | mol/min | | As in #203 |  |
| **311** | **ka(109)** | Parp→ | 0.01155245 | | min^-1^ | | As in #203 |  |
| **312** | **ka(110)** | cParp→ | 0.06931472 | | min^-1^ | | As in #203 |  |
| **313** | **ka(111)** | →L | 0 | | mol/min | | Set to zero to mimic a constant low death stimulus observed under non-stimulated conditions (see Fig 8). |  |
| **314** | **ka(112)** | L → | 0 | | min^-1^ | | As in #313 |  |
| **315** | **ka(113)** | →mCytoC | 5776.27053 | | mol/min | | As in #203 |  |
| **316** | **ka(114)** | mCytoC → | 0.01155245 | | min^-1^ | | As in #203 |  |
| **317** | **ka(115)** | cCytoC→ | 0.11552453 | | min^-1^ | | As in #203 |  |
| **399** | **PP1A** | kcc(44)/(kcc(43)*(kcc(45)*([CycA]+[CycE])+kcc(46)*[CycB])+1) | | | | | As in #318 |  |
| **318** | **kcc(1)** | Early and Delayed Response Genes (ERG/DERG) parameters in the original model that have been replaced with NFκB-mediated activation of the cell-cycle. See also Table S9. | 0.004167 | | Units are arbitrary and non-biological. All rate constants are per min as with the NFκB and apoptosis models. | Refer to ([Conradie et al, 2010](#_ENREF_3)) for details. | | |
| **319** | **kcc(2)** |  | 0.004167 | | As in #318 | As in #318 | | |
| **320** | **kcc(3)** |  | 0.1 | | As in #318 | As in #318 | | |
| **321** | **kcc(4)** |  | 0.005833 | | As in #318 | As in #318 | | |
| **322** | **kcc(5)** |  | 0.000833 | | As in #318 | As in #318 | | |
| **323** | **kcc(6)** |  | 0.3 | | As in #318 | As in #318 | | |
| **324** | **kcc(7)** |  | 0.166667 | | As in #318 | As in #318 | | |
| **325** | **kcc(8)** | kcc(77)kcc(8)[tCycD] | 0.833333 | | As in #318 | As in #318 | | |
| **326** | **kcc(9)** | kcc(9)[CD]  kcc(9)[CycD] | 0.083333 | | As in #318 | As in #318 | | |
| **327** | **kcc(10)** | kcc(10)[p27][CycD] | 16.66667 | | As in #318 | As in #318 | | |
| **328** | **kcc(11)** | kcc(11)[CD] | 0.166667 | | As in #318 | As in #318 | | |
| **329** | **kcc(12)** | kcc(77)(kcc(12)+kcc(13)[E2F]) | 0 | | As in #318 | As in #318 | | |
| **330** | **kcc(13)** |  | 0.01 | | As in #318 | As in #318 | | |
| **331** | **kcc(14)** | V8 | 0.001667 | | As in #318 | As in #318 | | |
| **332** | **kcc(15)** | V8 | 0.033333 | | As in #318 | As in #318 | | |
| **333** | **kcc(16)** | kcc(16)[p27][CycE]  kcc(16)[p27][CycA] | 16.66667 | | As in #318 | As in #318 | | |
| **334** | **kcc(17)** | kcc(17)[CE]  kcc(17)[CA] | 0.166667 | | As in #318 | As in #318 | | |
| **335** | **kcc(18)** | V8 | 0.1 | | As in #318 | As in #318 | | |
| **336** | **kcc(19)** |  | 1 | | As in #318 | As in #318 | | |
| **337** | **kcc(20)** |  | 0.05 | | As in #318 | As in #318 | | |
| **338** | **kcc(21)** | kcc(77)kcc(21)[E2F]max(0,GMAct*(vcc(33)-vcc(55))*300)) if [Mass] > 0.5, 0 otherwise | 0.008333 | | As in #318 | As in #318 | | |
| **339** | **kcc(22)** | kcc(22)[CDc20][CycA]  kcc(22)[CDc20][CA] | 0.333333 | | As in #318 | As in #318 | | |
| **#** | **Name** | **Description** | **Value** | | **Units** | **Justification** | | |
| **340** | **kcc(23)** | kcc(77)(kcc(24)*(([CycB]/kcc(25))^2/([CycB]/kcc(25))^2+1))+kcc(23) | 0.001667 | | As in #318 | As in #318 | | |
| **341** | **kcc(24)** |  | 0.01 | | As in #318 | As in #318 | | |
| **342** | **kcc(25)** |  | 0.1 | | As in #318 | As in #318 | | |
| **343** | **kcc(26)** | V2 | 0.000833 | | As in #318 | As in #318 | | |
| **344** | **kcc(27)** |  | 0.333333 | | As in #318 | As in #318 | | |
| **345** | **kcc(28)** |  | 0.016667 | | As in #318 | As in #318 | | |
| **346** | **kcc(29)** | kcc(77)kcc(29) | 0.333333 | | As in #318 | As in #318 | | |
| **347** | **kcc(30)** | V6 | 0.166667 | | As in #318 | As in #318 | | |
| **348** | **kcc(31)** |  | 1.666667 | | As in #318 | As in #318 | | |
| **349** | **kcc(32)** |  | 0.5 | | As in #318 | As in #318 | | |
| **350** | **kcc(33)** |  | 1 | | As in #318 | As in #318 | | |
| **351** | **kcc(34)** |  | 0.5 | | As in #318 | As in #318 | | |
| **352** | **kcc(35)** | RB total (not used) | 1.666667 | | As in #318 | As in #318 | | |
| **353** | **kcc(36)** | [E2F-Rb](kcc(40) (([CycD]+[CD])kcc(36)+kcc(39)[CycA]+kcc(38)[CycB]+kcc(37)[CycD]))  [p-E2F-Rb](kcc(40) (([CycD]+[CD])kcc(36)+kcc(39)[CycA]+kcc(38)[CycB]+kcc(37)[CycD])) | 3.3 | | As in #318 | As in #318 | | |
| **354** | **kcc(37)** |  | 5 | | As in #318 | As in #318 | | |
| **355** | **kcc(38)** |  | 5 | | As in #318 | As in #318 | | |
| **356** | **kcc(39)** |  | 3 | | As in #318 | As in #318 | | |
| **357** | **kcc(40)** |  | 0.166667 | | As in #318 | As in #318 | | |
| **358** | **kcc(41)** | [pp-Rb](kcc(41)(kcc(44)-PP1A)+kcc(42)PP1A) | 0 | | As in #318 | As in #318 | | |
| **359** | **kcc(42)** |  | 0.333333 | | As in #318 | As in #318 | | |
| **360** | **kcc(43)** | PP1A | 1 | | As in #318 | As in #318 | | |
| **361** | **kcc(44)** | [pp-Rb](kcc(41)(kcc(44)-PP1A)+kcc(42)PP1A) | 1 | | As in #318 | As in #318 | | |
| **362** | **kcc(45)** | PP1A | 25 | | As in #318 | As in #318 | | |
| **363** | **kcc(46)** | PP1A | 2 | | As in #318 | As in #318 | | |
| **364** | **kcc(47)** | ((kcc(47)+kcc(48)[CDc20](1-[Cdh1]))/(kcc(49)-[Cdh1]+1) if [Cdh1] <=1 else 0 | 0.125 | | As in #318 | As in #318 | | |
| **365** | **kcc(48)** |  | 2.333333 | | As in #318 | As in #318 | | |
| **366** | **kcc(49)** |  | 0.01 | | As in #318 | As in #318 | | |
| **367** | **kcc(50)** | V4[Cdh1])/(kcc(50)+[Cdh1]) | 0.01 | | As in #318 | As in #318 | | |
| **368** | **kcc(51)** | V4 | 0.666667 | | As in #318 | As in #318 | | |
| **369** | **kcc(52)** |  | 0 | | As in #318 | As in #318 | | |
| **370** | **kcc(53)** |  | 1 | | As in #318 | As in #318 | | |
| **371** | **kcc(54)** |  | 0.3 | | As in #318 | As in #318 | | |
| **372** | **kcc(55)** | kcc(77)kcc(55) | 0.000833 | | As in #318 | As in #318 | | |
| **373** | **kcc(56)** | kcc(56)[PPX] | 0.000833 | | As in #318 | As in #318 | | |
| **374** | **kcc(57)** | (kcc(57)[CycB](1-[IEP]))/(kcc(59)-[IEP]+1) | 0.011667 | | As in #318 | As in #318 | | |
| **375** | **kcc(58)** | (kcc(58)[PPX][IEP])/(kcc(60)+[IEP]) | 0.03 | | As in #318 | As in #318 | | |
| **376** | **kcc(59)** | (kcc(57)[CycB](1-[IEP]))/(kcc(59)-[IEP]+1) | 0.01 | | As in #318 | As in #318 | | |
| **377** | **kcc(60)** | (kcc(58)[PPX][IEP])/(kcc(60)+[IEP]) | 0.01 | | As in #318 | As in #318 | | |
| **378** | **kcc(61)** | kcc(77)(kcc(61)+kcc(62)[CycB]) | 0 | | As in #318 | As in #318 | | |
| **379** | **kcc(62)** |  | 0.025 | | As in #318 | As in #318 | | |
| **380** | **kcc(63)** | kcc(63)[CDc20T] kcc(63)[CDc20] | 0.025 | | As in #318 | As in #318 | | |
| **381** | **kcc(64)** | (kcc(64)[IEP]([CDc20T]-[CDc20]))/(kcc(66)-[CDc20]+[CDc20T]) | 0.083333 | | As in #318 | As in #318 | | |
| **382** | **kcc(65)** | kcc(65)[CDc20]/(kcc(67)+[CDC20]) | 0.041667 | | As in #318 | As in #318 | | |
| **383** | **kcc(66)** | (kcc(64)[IEP]([CDc20T]-[CDc20]))/(kcc(66)-[CDc20]+[CDc20T]) | 0.005 | | As in #318 | As in #318 | | |
| **384** | **kcc(67)** | kcc(65)[CDc20]/(kcc(67)+[CDC20]) | 0.005 | | As in #318 | As in #318 | | |
| **385** | **kcc(68)** | E2F Total (not used) | 0.833333 | | As in #318 | As in #318 | | |
| **386** | **kcc(69)** | [p-E2F]kcc(69) | 0.016667 | | As in #318 | As in #318 | | |
| **387** | **kcc(70)** | [E2F](kcc(70)+kcc(71) *([CycA]+[CycB]))) | 8.33E-05 | | As in #318 | As in #318 | | |
| **388** | **kcc(71)** |  | 0.016667 | | As in #318 | As in #318 | | |
| **389** | **kcc(72)** | [E2F][Rb]kcc(72)  [Rb][p-E2F]kcc(72) | 166.6667 | | As in #318 | As in #318 | | |
| **390** | **kcc(73)** | [p-E2F-Rb]kcc(73) | 3.333333 | | As in #318 | As in #318 | | |
| **391** | **kcc(74)** | kcc(74)(0.01+0.99*GMAct*GMSize) if RbFrac < 0.8, kcc(74)*0.01 else | 0.002 | | As in #318 | As in #318 | | |
| **#** | **Name** | **Description** | **Value** | | **Units** | **Justification** | | |
| **392** | **kcc(75)** | kcc(75)[GM] | 0.00015 | | As in #318 | As in #318 | | |
| **393** | **kcc(76)** | kcc(77)kcc(76)[GM] | 0.0045 | | As in #318 | As in #318 | | |
| **394** | **kcc(77)** | Eps, the efficiency of protein translation reactions. | 1 or 0.7 when simulating rapamycin | | As in #318 | As in #318  0.7 was manually derived to fit rapamycin treated data in this study. See also Table S9 for further details. | | |
| **395** | **kcc(78)** | kcc(78)CycDAct | 0.003 | | As in #318 | Free parameter (see Table S9). | | |
| **396** | **kcc(79)** | kcc(79)[tCycD] | 0.002 | | As in #318 | As in #395 | | |
| **397** | **kcc(80)** | max(kcc(80)BclAct,0.01kcc(80) | 8 | | As in #318 | As in #395 | | |
| **398** | **kcc(81)** | kcc(81)[tBcl2] | 0.005 | | As in #318 | As in #395 | | |
| **399** | **kcc(82)** | kcc(82)[tMycTor] | 0.0035 | | As in #318 | As in #395 | | |
| **400** | **kcc(83)** | kcc(83)[MycTor] | 0.0025 | | As in #318 | As in #395 | | |
| **401** | **kcc(84)** | kcc(84)[Mass] | 0.0025 | | As in #318 | As in #395 | | |
| **402** | **kcc(85)** | kcc(85)MycAct | 1 | | As in #318 | As in #395 | | |
| **403** | **kcc(86)** | kcc(86)[tMycTor] | 0.0231 | | As in #318 | Reported 30 min half-life ([Jones & Cole, 1987](#_ENREF_7)) | | |
| **404** | **V2** | kcc(28)*[CDc20]+kcc(26)*(1-[Cdh1])+kcc(27)*[Cdh1] | | | | As in #318 | | |
| **405** | **V4** | kcc(51)*(kcc(54)[CycA]+kcc(53)[CycB]+kcc(52)[CycE]) | | | | As in #318 | | |
| **406** | **V6** | kcc(30)+kcc(31)*(kcc(34)[CycA]+kcc(33)[CycB]+kcc(32)[CycE] | | | | As in #318 | | |
| **407** | **V8** | ((kcc(19)*([CycA]+[CycE])+kcc(20)[CycB]))*kcc(15))/  ([CycE]+[CE]_kcc(18))+kcc(14) | | | | As in #318 | | |
| **408** | **H** | Hill coefficient for Myc/Bcl/CycD/GM promoter activation | | 2 | | Set to 2 to ensure delayed dynamics in generation 0 cells as observed in this study. | | |
| **409** | **k(1,Myc/TOR)** | d[tMyc]/dt parameter | | 0.45 | | Derived from ([Grumont et al, 2002](#_ENREF_6)) and from pS6/IF measurements in this study | | |
| **410** | **k(2,Myc/TOR)** | d[tMyc]/dt parameter | | 0.45 | | As in #409 | | |
| **411** | **k(3,Myc/TOR)** | d[tMyc]/dt parameter | | 0.1 | | NFκB independent activity assumed to be low  As in #409 | | |
| **412** | **k(4,Myc/TOR)** | d[tMyc]/dt parameter | | 1 | | Scaling factor | | |
| **413** | **k(5,Myc/TOR)** | d[tMyc]/dt parameter | | 0.01 | | Basal transcription | | |
| **413** | **k(1,Bcl)** | d[tBcl_XL_]/dt parameter | | 0.3 | | Derived from IF and RT-PCR in this study | | |
| **414** | **k(2,Bcl)** | d[tBcl_XL_]/dt parameter | | 0.6 | | As in #413 | | |
| **415** | **k(3,Bcl)** | d[tBcl_XL_]/dt parameter | | 0.1 | | NFκB independent activity assumed to be low | | |
| **416** | **k(4,Bcl)** | d[tBcl_XL_]/dt parameter | | 1 | | As in #412 | | |
| **417** | **k(5,Bcl)** | d[tBcl_XL_]/dt parameter | | 0.01 | | As in #413 | | |
| **418** | **k(1,CycD)** | d[tCycD]/dt parameter | | 0.45 | | We assumed equal NFκB RelA and cRel dependence for Cyclin D transcription as it is unclear how each monomer contributes to cyclin D2 or 3 mediated cell-cycle progression.([Piatelli et al, 2003](#_ENREF_13)) | | |
| **419** | **k(2,CycD)** | d[tCycD]/dt parameter | | 0.45 | |  |  |  |
| **420** | **k(3,CycD)** | d[tCycD]/dt parameter | | 0.1 | | NFκB independent activity assumed to be low to ensure the observed dynamics. | | |
| **421** | **k(4,CycD)** | d[tCycD]/dt parameter | | 1 | | As in #412 | | |
| **422** | **k(5,CycD)** | d[tCycD]/dt parameter | | 0.01 | | As in #413 | | |
| **423** | **MycKd** | NFκB scaling parameter that determines NFκB-mediated Myc activation | | 40 nM | | Manually fitted to ensure wildtype B cell population dynamics. | | |
| **424** | **Bcl_XL_Kd** | NFκB scaling parameter that determines NFκB-mediated Bcl_XL_ activation | | 40 nM | | As in #423 | | |
| **425** | **CycDKd** | NFκB scaling parameter that determines NFκB-mediated CycD activation | | 40 nM | | As in #423 | | |
| **426** | **GMKd** | Myc scaling parameter that determines Myc -mediated GM growth | | 40 nM | | As in #423 | | |
| **#** | **Name** | **Description** | | **Units** | | **Justification** | | |
| **427** | **MycAct** | (((k(1,Myc)[nA50]+k(2,Myc)[nC50]+k(3,Myc)[IKK*])/MycKd)^H)/(k(4,Myc)+ ((k(1,Myc)[nA50]+k(2,Myc)[nC50]+k(3,Myc)[IKK*])/MycTorKd)^H)*(1-k(5,Myc))+k(5,Myc) | | Unitless 0-1 | | Hill-based expression of Myc transcription activity. See #408-413 | | |
| **428** | **BclAct** | (((k(1,Bcl)[nA50]+k(2, Bcl)[nC50]+k(3, Bcl)[IKK*])/ Bcl _XL_Kd)^H)/(k(4, Bcl)+ ((k(1, Bcl)[nA50]+k(2, Bcl)[nC50]+k(3, Bcl)[IKK*])/ Bcl_XL_ Kd)^H)*(1-k(5,Bcl))+k(5,Bcl) | | Unitless 0-1 | | Hill-based expression of Bcl_XL_ transcription activity. See #413-417 | | |
| **429** | **CycDAct** | (((k(1,CycD)[nA50]+k(2,CycD)[nC50]+k(3,CycD)[IKK*])/CycDKd)^H)/(k(4,CycD)+ ((k(1,CycD)[nA50]+k(2,CycD)[nC50]+k(3,CycD)[IKK*])/CycDKd)^H)*(1-k(5,CycD))+k(5,CycD) | | Unitless 0-1 | | Hill-based expression of CycD transcription activity. See #418-422 | | |
| **430** | **GMAct** | ([Myc]/GMKd)^H/(1+([Myc]/GMKd)^H) | | Unitless 0-1 | | Assume basic Hill relationships between Myc and the accumulation of general machinery in the cell. See ([Link & Hurlin, 2014](#_ENREF_10); [Nie et al, 2012](#_ENREF_12); [Wang et al, 2011](#_ENREF_15)) and ([Limon & Fruman, 2012](#_ENREF_9); [Zeng & Chi, 2013](#_ENREF_17)) reviewing myc and mTOR in the context of B-cell growth. | | |
| **431** | **RbFrac** | ([Rb]+[E2F-Rb]+[p-E2F-Rb])/( [Rb]+[E2F-Rb]+[p-E2F-Rb]+[pp-Rb]) | | Unitless 0-1 | | As in #318 | | |
| **432** | **IKK_Kd** | 1950 for 250 nM condition, 1500 for 10 nM condition. | | nM^-1^ | | Manually fitted to produce experimentally observed population behavior in wildtype cells. See also Table S9. | | |

Rate constants used to simulate the physicokinetic models for NFκB signaling, cell-cycle progression, and apoptosis. Note: We did not model mTOR explicitly in the model because the exact relationship between Myc and TOR is not well characterized in B cells. In our study we found that mTOR is downstream of NFkB activation, but the exact mechanisms are still poorly known. Instead, we assume that cell growth and the synthesis of general cellular machinery can be approximated by Myc activation, which is known to be under NFkB regulatory control. The rate constants that we introduced into the model are explored in further detail below (Table S8).

# Table S5. Integrated B-cell model flux reactions.

| **#** | **Name** | **Flux equation** | **Justification** |
| --- | --- | --- | --- |
| **1-4** | vd(1,d∈{AA,A50,5050,C50}) | kd(1,d)[m∈{RelA,RelA,p50,cRel}][ n∈{RelA,p50,p50,p50}] | See ([Alves et al, 2014](#_ENREF_2)) |
| **5-8** | vd(2, d∈{AA,A50,5050,C50}) | kd(2,d) [m∈{nRelA,nRelA,np50,ncRel}][ n∈{nRelA,np50,np50,np50}] | As in #1 |
| **9-12** | vd(3,d∈{AA,A50,5050,C50}) | kd(3,d)[d] | As in #1 |
| **13-16** | vd(4,d∈{AA,A50,5050,C50}) | kd(4,d)[ e∈{nAA,nA50,n5050,nC50}] | As in #1 |
| **17-20** | vd(5,d∈{AA,A50,5050,C50}) | kd(5,d)[d] | As in #1 |
| **21-24** | vd(6,d∈{AA,A50,5050,C50}) | kd(6,d)[ e∈{nAA,nA50,n5050,nC50}] | As in #1 |
| **25-28** | vd(7,d∈{AA,A50,5050,C50}) | kd(7,d)[d] | As in #1 |
| **29-32** | vd(8,d∈{AA,A50,5050,C50}) | kd(8,d)[ e∈{nAA,nA50,n5050,nC50}] | As in #1 |
| **33-44** | vdi(1, d∈{AA,A50,5050,C50}, i∈{α,β,∈}) | kp(1,d,i)[d][ j∈{IkBα, IkBβ,IkB∈}] | As in #1 |
| **45-56** | vdi(2, d∈{AA,A50,5050,C50}, i∈{α,β,∈}) | kp(2,d,i)[e ∈{nAA,nA50,n5050,nC50}][j∈{nIkBα, nIkBβ,nIkB∈}] | As in #1 |
| **57-68** | vdi(3, d∈{AA,A50,5050,C50}, i∈{α,β,∈}) | kp(3,d,i)[d: j∈{IkBα, IkBβ,IkB∈}] | As in #1 |
| **69-80** | vdi(4, d∈{AA,A50,5050,C50}, i∈{α,β,∈}) | kp(4,d,i)[ e ∈{nAA,nA50,n5050,nC50} : j∈{nIkBα, nIkBβ,nIkB∈}] | As in #1 |
| **81-92** | vdi(5, d∈{AA,A50,5050,C50}, i∈{α,β,∈}) | kp(5,d,i)[d: j∈{IkBα, IkBβ,IkB∈}] | As in #1 |
| **93-104** | vdi(6, d∈{AA,A50,5050,C50}, i∈{α,β,∈}) | kp(6,d,i)[ e ∈{nAA,nA50,n5050,nC50} : j∈{nIkBα, nIkBβ,nIkB∈}] | As in #1 |
| **105-116** | vdi(7, d∈{AA,A50,5050,C50}, i∈{α,β,∈}) | ki(12, j∈{IkBα, IkBβ,IkB∈}) [d: j∈{IkBα, IkBβ,IkB∈}][IKK*] | As in #1 |
| **117-128** | vdi(8, d∈{AA,A50,5050,C50}, i∈{α,β,∈}) | ki(13, i)[d: j∈{IkBα, IkBβ,IkB∈}] | As in #1 |
| **129-140** | vdi(9, d∈{AA,A50,5050,C50}, i∈{α,β,∈}) | ki(14, i)[ e ∈{nAA,nA50,n5050,nC50} : j∈{nIkBα, nIkBβ,nIkB∈}] | As in #1 |
| **141-152** | vdi(10, d∈{AA,A50,5050,C50}, i∈{α,β,∈}) | kd(9, d)[d: j∈{IkBα, IkBβ,IkB∈}] | As in #1 |
| **153-164** | vdi(11, d∈{AA,A50,5050,C50}, i∈{α,β,∈}) | ki(10, d)[ e ∈{nAA,nA50,n5050,nC50} : j∈{nIkBα, nIkBβ,nIkB∈}] | As in #1 |
| **165-167** | vi(i∈{α,β,∈},t) | ki(1,i)(1+Σ_d∈(AA,A50,C50)_(kp(7,i)([e ∈{nAA,nA50,n5050,nC50}]\|_t-ki(8,i)_ /ki(10,i))^ ki(9, i))/ (1+Σ_d∈(AA,A50,C50)_(([e ∈{nAA,nA50,n5050,nC50}]\|_t-ki(8,i)_ /ki(10,i))^ ki(9, i))/ | As in #1. The transcription rate is computed using a modified hill function which uses a sum of weighted contributions from each dimer and an explicit transcriptional delay to compute the transcription rate of each IkB transcript. |
| **168-170** | vm(m∈{A,50,C},t) | km(1,m)(1+Σ_d∈(AA,A50,C50)_(kdm(d,m)([e ∈{nAA,nA50,n5050,nC50}]\|_t-km(6,i)_ /km(8,m))^ km(7,m)) /(1+Σ_d∈(AA,A50,C50)_(([e ∈{nAA,nA50,n5050,nC50}]\|_t-km(6,i)_ /km(8,m))^km(7,m)) | As in #165-167. The transcription rate is computed using a modified hill function which uses a sum of weighted contributions from each dimer and an explicit transcriptional delay to compute the transcription rate of each NFκB monomer. |
| **171** | vtm(1,m∈{A,50,C}) | km(2,m)[n∈{tRelA,tp50,tcRel}] | As in #1 |
| **172** | vtm(2,m∈{A,50,C}) | kcc(77)km(3,m)[n∈{tRelA,tp50,tcRel}] | As in #1 |
| **173** | vtm(3,m∈{A,50,C}) | km(4,m)[m] | As in #1 |
| **174** | vtm(4,m∈{A,50,C}) | km(5,m)[ n∈{nRelA,np50,ncRel}]] | As in #1 |
| **175** | vti(1, i∈{α,β,∈}) | ki(2,i)[ j∈{tIkBα, tIkBβ,tIkB∈}] | As in #1 |
| **#** | **Name** | **Flux equation** | **Justification** |
| **176** | vti(2, i∈{α,β,∈}) | kcc(77)ki(3,i)[ j∈{tIkBα, tIkBβ,tIkB∈}] | As in #1 |
| **177** | vti(3, i∈{α,β,∈}) | ki(11,i) [ j∈{IkBα, IkBβ,IkB∈}][IKK*] | As in #1 |
| **178** | vti(4, i∈{α,β,∈}) | ki(4,i) [ j∈{IkBα, IkBβ,IkB∈}] | As in #1 |
| **179** | vti(5, i∈{α,β,∈}) | ki(5,i) [ j∈{nIkBα, nIkBβ,nIkB∈}] | As in #1 |
| **180** | vti(6, i∈{α,β,∈}) | ki(6,i) [ j∈{IkBα, IkBβ,IkB∈}] | As in #1 |
| **181** | vti(7, i∈{α,β,∈}) | ki(7,i) [ j∈{nIkBα, nIkBβ,nIkB∈}] | As in #1 |
| **182** | va(1) | ka(1)[L][R] | ([Loriaux et al, 2013](#_ENREF_11)) |
| **183** | va(2) | ka(2)[L-R] | As in #182 |
| **184** | va(3) | ka(3)[flip][DISC] | As in #182 |
| **185** | va(4) | ka(4)[flip-DISC] | As in #182 |
| **186** | va(5) | ka(5)[pC8][DISC] | As in #182 |
| **187** | va(6) | ka(6)[DISC-pC8] | As in #182 |
| **188** | va(7) | ka(7)[DISC-pC8] | As in #182 |
| **189** | va(8) | ka(8)[C8][BAR] | As in #182 |
| **190** | va(9) | ka(9)[BAR-C8] | As in #182 |
| **191** | va(10) | ka(10)[pC3][C8] | As in #182 |
| **192** | va(11) | ka(11)[pC3-C8] | As in #182 |
| **193** | va(12) | ka(12)[pC3-C8] | As in #182 |
| **194** | va(13) | ka(13)[pC6][C3] | As in #182 |
| **195** | va(14) | ka(14)[pC6-C3] | As in #182 |
| **196** | va(15) | ka(15)[pC6-C3] | As in #182 |
| **197** | va(16) | ka(16)[pC8][C6] | As in #182 |
| **198** | va(17) | ka(17)[pC8-C6] | As in #182 |
| **199** | va(18) | ka(18)[pC8-C6] | As in #182 |
| **200** | va(19) | ka(19)[XIAP][C3] | As in #182 |
| **201** | va(20) | ka(20)[XIAP-C3] | As in #182 |
| **202** | va(21) | ka(21)[XIAP-C3] | As in #182 |
| **203** | va(22) | ka(22)[PARP][C3] | As in #182 |
| **204** | va(23) | ka(23)[PARP-C3] | As in #182 |
| **205** | va(24) | ka(24)[PARP-C3] | As in #182 |
| **206** | va(25) | ka(25)[Bid][C8] | As in #182 |
| **207** | va(26) | ka(26)[Bid-C8] | As in #182 |
| **208** | va(27) | ka(27)[Bid-C8] | As in #182 |
| **209** | va(28) | ka(28)[tBid][Mcl1] | As in #182 |
| **210** | va(29) | ka(29)[tBid-Mcl1] | As in #182 |
| **211** | va(30) | ka(30)[Bax][tBid] | As in #182 |
| **212** | va(31) | ka(31)[Bax-tBid] | As in #182 |
| **213** | va(32) | ka(32)[Bax-tBid] | As in #182 |
| **214** | va(33) | ka(33)[aBax] | As in #182 |
| **215** | va(34) | ka(34)[mBax] | As in #182 |
| **216** | va(35) | ka(35)[mBax][BclXL] | As in #182 |
| **217** | va(36) | ka(36)[mBax-BclXL] | As in #182 |
| **218** | va(37) | ka(37)[mBax]^2 | As in #182 |
| **219** | va(38) | ka(38)[Bax2] | As in #182 |
| **220** | va(39) | ka(39)[Bax2][BclXL] | As in #182 |
| **221** | va(40) | ka(40)[mBax2-BclXL] | As in #182 |
| **222** | va(41) | ka(41)[Bax2]^2 | As in #182 |
| **223** | va(42) | ka(42)[Bax4] | As in #182 |
| **224** | va(43) | ka(43)[Bax4][BclXL] | As in #182 |
| **225** | va(44) | ka(44)[mBax4-BclXL] | As in #182 |
| **226** | va(45) | ka(45)[Bax4][Mito] | As in #182 |
| **227** | va(46) | ka(46)[Bax4-Mito] | As in #182 |
| **228** | va(47) | ka(47)[Bax4-Mito] | As in #182 |
| **229** | va(48) | ka(48)[AMito][mCytoC] | As in #182 |
| **230** | va(49) | ka(49)[AMito-mCytoC] | As in #182 |
| **231** | va(50) | ka(50)[AMito-mCytoC] | As in #182 |
| **232** | va(51) | ka(51)[AMito][mSMac] | As in #182 |
| **233** | va(52) | ka(52)[AMito-mSMac] | As in #182 |
| **234** | va(53) | ka(53)[AMito][mSMac] | As in #182 |
| **235** | va(54) | ka(54)[aCytoC] | As in #182 |
| **236** | va(55) | ka(55)[cCytoC] | As in #182 |
| **237** | va(56) | ka(56)[Apaf][cCytoC] | As in #182 |
| **238** | va(57) | ka(57)[Apaf-cCytoC] | As in #182 |
| **#** | **Name** | **Flux equation** | **Justification** |
| **239** | va(58) | ka(58)[Apaf-cCytoC] | As in #182 |
| **240** | va(59) | ka(59)[Apaf*][ProC9] | As in #182 |
| **241** | va(60) | ka(60)[Apop] | As in #182 |
| **242** | va(61) | ka(61)[Apop][pC3] | As in #182 |
| **243** | va(62) | ka(62)[Apop-pC3] | As in #182 |
| **244** | va(63) | ka(63)[Apop-pC3] | As in #182 |
| **245** | va(64) | ka(64)[aSmac] | As in #182 |
| **246** | va(65) | ka(65)[cSmac] | As in #182 |
| **247** | va(66) | ka(66)[Apop][XIAP] | As in #182 |
| **248** | va(67) | ka(67)[Apop-XIAP] | As in #182 |
| **249** | va(68) | ka(68)[cSmac][XIAP] | As in #182 |
| **250** | va(69) | ka(69)[cSmac-XIAP] | As in #182 |
| **251** | va(70) | ka(70) | As in #182 |
| **252** | va(71) | ka(71)[R} | As in #182 |
| **253** | va(72) | ka(72) | As in #182 |
| **254** | va(73) | ka(73)[flip] | As in #182 |
| **255** | va(74) | ka(74)[flip-DISC] | As in #182 |
| **256** | va(75) | ka(75) | As in #182 |
| **257** | va(76) | ka(76)[pC8] | As in #182 |
| **258** | va(77) | ka(77) | As in #182 |
| **259** | va(78) | ka(78)[BAR} | As in #182 |
| **260** | va(79) | ka(79)[BAR-C8] | As in #182 |
| **261** | va(80) | ka(80) | As in #182 |
| **262** | va(81) | ka(81)[Bid] | As in #182 |
| **263** | va(82) | ka(82) | As in #182 |
| **264** | va(83) | ka(83)[Mcl1] | As in #182 |
| **265** | va(84) | ka(84)[tBid-Mcl1] | As in #182 |
| **266** | va(85) | ka(85) | As in #182 |
| **267** | va(86) | ka(86)[Bax] | As in #182 |
| **268** | va(87) | ka(87) | As in #182 |
| **269** | va(88) | ka(88)[BclXL] | As in #182 |
| **270** | va(89) | ka(89)[mBax-BclXL] | As in #182 |
| **271** | va(90) | ka(90)[Bax2-BclXL] | As in #182 |
| **272** | va(91) | ka(91)[Bax4-BclXL] | As in #182 |
| **273** | va(92) | ka(92)[AMito] | As in #182 |
| **274** | va(93) | ka(93)[Apaf*] | As in #182 |
| **275** | va(94) | ka(94) | As in #182 |
| **276** | va(95) | ka(95)[XIAP] | As in #182 |
| **277** | va(96) | ka(96) | As in #182 |
| **278** | va(97) | ka(97)[mSmac] | As in #182 |
| **279** | va(98) | ka(98)[cSmac] | As in #182 |
| **280** | va(99) | ka(99)[cSmac-XIAP] | As in #182 |
| **281** | va(100) | ka(100) | As in #182 |
| **282** | va(101) | ka(101)[pC3] | As in #182 |
| **283** | va(102) | ka(102)[C3_U] | As in #182 |
| **284** | va(103) | ka(103) | As in #182 |
| **285** | va(104) | ka(104)[pC6] | As in #182 |
| **286** | va(105) | ka(105)[C6] | As in #182 |
| **287** | va(106) | ka(106)[Apop-XIAP] | As in #182 |
| **288** | va(107) | ka(107) | As in #182 |
| **289** | va(108) | ka(108)[Parp] | As in #182 |
| **290** | va(109) | ka(109)[cParp] | As in #182 |
| **291** | va(110) | ka(110) | As in #182 |
| **292** | va(111) | ka(111)[L] | As in #182 |
| **293** | va(112) | ka(112) | As in #182 |
| **294** | va(113) | ka(113)[mCytoC] | As in #182 |
| **295** | va(114) | ka(114)[cCytoC] | As in #182 |
| **296** | vcc(1) | kcc(79)[tCycD] | See the original paper ([Conradie et al, 2010](#_ENREF_3)). |
| **297** | vcc(2) | kcc(81)[tBcl2] | As in #296 |
| **298** | vcc(3) | kcc(9)[CD] | As in #296 |
| **299** | vcc(4) | kcc(9)[CycD] | As in #296 |
| **300** | vcc(5) | kcc(16)[p27][CycE] | As in #296 |
| **#** | **Name** | **Flux equation** | **Justification** |
| **301** | vcc(6) | kcc(16)[p27][CycA] | As in #296 |
| **302** | vcc(7) | kcc(10)[p27][CycD] | As in #296 |
| **303** | vcc(8) | kcc(11)[CD] | As in #296 |
| **304** | vcc(9) | kcc(22)[CDc20][CycA] | As in #296 |
| **305** | vcc(10) | kcc(22)[CDc20][CA] | As in #296 |
| **306** | vcc(11) | kcc(17)[CE] | As in #296 |
| **307** | vcc(12) | kcc(17)[CA] | As in #296 |
| **308** | vcc(13) | V8[CE] | As in #296 |
| **309** | vcc(14) | V8[CycE] | As in #296 |
| **310** | vcc(15) | V6[p27] | As in #296 |
| **311** | vcc(16) | V6[CE] | As in #296 |
| **312** | vcc(17) | V6[CD] | As in #296 |
| **313** | vcc(18) | V6[CA] | As in #296 |
| **314** | vcc(19) | V2[CycB] | As in #296 |
| **315** | vcc(20) | ((kcc(47)+kcc(48)[CDc20](1-[Cdh1]))/(kcc(49)-[Cdh1]+1) if [Cdh1] <=1 else 0 | As in #296 |
| **316** | vcc(21) | V4[Cdh1])/(kcc(50)+[Cdh1]) | As in #296 |
| **317** | vcc(22) | kcc(56)[PPX] | As in #296 |
| **318** | vcc(23) | (kcc(57)[CycB](1-[IEP]))/(kcc(59)-[IEP]+1) | As in #296 |
| **319** | vcc(24) | (kcc(58)[PPX][IEP])/(kcc(60)+[IEP]) | As in #296 |
| **320** | vcc(25) | kcc(63)[CDc20T] | As in #296 |
| **321** | vcc(26) | (kcc(64)[IEP]([CDc20T]-[CDc20]))/(kcc(66)-[CDc20]+[CDc20T]) | As in #296 |
| **322** | vcc(27) | kcc(65)[CDc20]/(kcc(67)+[CDC20]) | As in #296 |
| **323** | vcc(28) | kcc(63)[CDc20] | As in #296 |
| **324** | vcc(29) | [E2F-Rb](kcc(40)(([CycD]+[CD])kcc(36)+kcc(39)[CycA]+kcc(38)[CycB]+kcc(37)[CycD])) | As in #296 |
| **325** | vcc(30) | [p-E2F- Rb](kcc(40)(([CycD]+[CD])kcc(36)+ kcc(39)[CycA]+kcc(38)[CycB]+kcc(37)[CycD])) | As in #296 |
| **326** | vcc(31) | kcc(74)(0.01+0.99*GMAct*GMSize) if RbFrac < 0.8, kcc(74)*0.01 otherwise | As in #296 |
| **327** | vcc(32) | kcc(75)[GM] | As in #296 |
| **328** | vcc(33) | kcc(77)kcc(76)[GM] | As in #296 |
| **329** | vcc(34) | kcc(78)CycDAct | As in #296 |
| **330** | vcc(35) | kcc(77)(kcc(61)+kcc(62)[CycB]) | As in #296 |
| **331** | vcc(36) | kcc(77)kcc(21)[E2F]max(0,GMAct*(vcc(33)-vcc(55))*300)) if [Mass] > 0.5, 0 otherwise | As in #296 |
| **332** | vcc(37) | kcc(77)kcc(55) | As in #296 |
| **333** | vcc(38) | kcc(77)(kcc(12)+kcc(13)[E2F]) | As in #296 |
| **334** | vcc(39) | kcc(77)kcc(8)[tCycD] | As in #296 |
| **335** | vcc(40) | kcc(77)kcc(29) | As in #296 |
| **336** | vcc(41) | max(kcc(80)BclAct,0.01kcc(80) | As in #296 |
| **337** | vcc(42) | kcc(77)(kcc(24)*(([CycB]/kcc(25))^2/([CycB]/kcc(25))^2+1))+kcc(23) | As in #296 |
| **338** | vcc(43) | [Rb](kcc(40)*([CycD]+[CD]kcc(36)+kcc(39)[CycA]+kcc(38)[CycB]+kcc(37)[CycE])) | As in #296 |
| **339** | vcc(44) | [pp-Rb](kcc(41)(kcc(44)-PP1A)+kcc(42)PP1A) | As in #296 |
| **340** | vcc(45) | [E2F-Rb]kcc(73) | As in #296 |
| **341** | vcc(46) | [E2F](kcc(70)+kcc(71)([CycA]+[CycB]))) | As in #296 |
| **342** | vcc(47) | [p-E2F]kcc(69) | As in #296 |
| **343** | vcc(48) | [E2F][Rb]kcc(72) | As in #296 |
| **344** | vcc(49) | [p-E2F-Rb]kcc(73) | As in #296 |
| **345** | vcc(50) | [Rb][p-E2F]kcc(72) | As in #296 |
| **346** | vcc(51) | [p-E2F-Rb]kcc(69) | As in #296 |
| **347** | vcc(52) | [E2F-Rb](kcc(70)+kcc(71)([CycA]+[CycB]))) | As in #296 |
| **348** | vcc(53) | kcc(82)[tMycTor] | As in #296 |
| **349** | vcc(54) | kcc(83)[MycTor] | As in #296 |
| **350** | vcc(55) | kcc(84)[Mass] | As in #296 |
| **351** | vcc(56) | kcc(85)MycAct | As in #296 |
| **341** | vcc(57) | kcc(86)[tMycTor] | As in #296 |

The fluxes governing the mass-action changes of species in the model as listed.

# Table S6. Integrated B-cell model reactions.

| **#** | **Species** | **Reaction** | **Justification** |
| --- | --- | --- | --- |
| **1** | d[tRelA]/dt | vm(RelA)-vtm(1,RelA)[tRelA] | See ([Alves et al, 2014](#_ENREF_2)) |
| **2** | d[tp50]/dt | vm(p50)-vtm(1,p50)[tp50] | As in #1 |
| **3** | d[tcRel]/dt | vm(cRel)-vtm(1,cRel)[tcRel] | As in #1 |
| **4** | d[RelA]/dt | vd(3,AA)*2+vd(3,A50)-vd(1,AA)-vd(1,A50)+vtm(2,RelA)[tRelA]-vtm(3,RelA)[RelA] | As in #1 |
| **5** | d[nRelA]/dt | vd(4,AA)*2+vd(4,A50)-vd(2,AA)-vd(2,A50)-vtm(4,RelA)[nRelA] | As in #1 |
| **6** | d[p50]/dt | vd(3,5050)*2+vd(3,A50)-+vd(3,C50)-vd(1,A50)-vd(1,C50)-vd(1,5050) )+vtm(2,p50)[tp50]-vtm(3,p50)[p50] | As in #1 |
| **7** | d[np50]/dt | vd(4,5050)*2+vd(4,A50)-+vd(4,C50)-vd(2,A50)-vd(2,C50)-vd(2,5050) -vtm(4,p50)[np50] | As in #1 |
| **8** | d[cRel]/dt | vd(3,C50)-vd(1,C50) )+vtm(2,cRel)[tcRel]-vtm(3,cRel)[cRel] | As in #1 |
| **9** | d[ncRel]/dt | vd(4,C50)-vd(2,C50) -vtm(4,cRel)[ncRel] | As in #1 |
| **10** | d[AA]/dt | vd(1,AA)-vd(3,AA)-vd(5,AA)+vd(6,AA)-vd(7,AA)-vdi(1,AA,[α,β,ε])+vdi(2,AA, [α,β,ε])+vdi(7,AA, [α,β,ε])+vdi(8,AA,[α,β,ε]) | As in #1 |
| **11** | d[A50]/dt | vd(1,A50)-vd(3,A50)-vd(5,A50)+vd(6,A50)-vd(7,A50)-vdi(1,A50,[α,β,ε])+vdi(2,A50, [α,β,ε])+vdi(7,A50, [α,β,ε])+vdi(8,A50,[α,β,ε]) | As in #1 |
| **12** | d[5050]/dt | vd(1,5050)-vd(3,5050)-vd(5,5050)+vd(6,5050)-vd(7,5050)-vdi(1,5050,[α,β,ε])+vdi(2,5050, [α,β,ε])+vdi(7,5050, [α,β,ε])+vdi(8,5050,[α,β,ε]) | As in #1 |
| **13** | d[C50]/dt | vd(1,C50)-vd(3,C50)-vd(5,C50)+vd(6,C50)-vd(7,C50)-vdi(1,C50,[α,β,ε])+vdi(2,C50, [α,β,ε])+vdi(7,C50, [α,β,ε])+vdi(8,C50,[α,β,ε]) | As in #1 |
| **14** | d[nAA]/dt | vd(2,AA)-vd(4,AA)-vd(6,AA)+vd(5,AA)-vd(8,AA)-vdi(2,AA,[α,β,ε])+vdi(4,AA, [α,β,ε])+vdi(9,AA, [α,β,ε]) | As in #1 |
| **15** | d[nA50]/dt | vd(2,A50)-vd(4,A50)-vd(6,A50)+vd(5,A50)-vd(8,A50)-vdi(2,A50,[α,β,ε])+vdi(4,A50, [α,β,ε])+vdi(9,A50, [α,β,ε]) | As in #1 |
| **16** | d[n5050]/dt | vd(2,5050)-vd(4,5050)-vd(6,5050)+vd(5,5050)-vd(8,5050)-vdi(2,5050,[α,β,ε])+vdi(4,5050, [α,β,ε])+vdi(9,5050, [α,β,ε]) | As in #1 |
| **17** | d[nC50]/dt | vd(2,C50)-vd(4,C50)-vd(6,C50)+vd(5,C50)-vd(8,C50)-vdi(2,C50,[α,β,ε])+vdi(4,C50, [α,β,ε])+vdi(9,C50, [α,β,ε]) | As in #1 |
| **18** | d[AA:IkBα,AA:IkBβ, AA:IkBε]/dt | vdi(1,AA, [α,β,ε])-vdi(3,AA, [α,β,ε])-vdi(5,AA, [α,β,ε])+vdi(6,AA, [α,β,ε])-vdi(7,AA, [α,β,ε])-vdi(8,AA, [α,β,ε])-vdi(10,AA, [α,β,ε]) | As in #1 |
| **19** | d[A50:IkBα,A50:IkBβ, A50:IkBε]/dt | vdi(1,A50, [α,β,ε])-vdi(3,A50, [α,β,ε])-vdi(5,A50, [α,β,ε])+vdi(6,A50, [α,β,ε])-vdi(7,A50, [α,β,ε])-vdi(8,A50, [α,β,ε])-vdi(10,A50, [α,β,ε]) | As in #1 |
| **20** | d[5050:IkBα, 5050:IkBβ, 5050:IkBε]/dt | vdi(1,5050, [α,β,ε])-vdi(3,5050, [α,β,ε])-vdi(5,5050, [α,β,ε])+vdi(6,5050, [α,β,ε])-vdi(7,5050, [α,β,ε])-vdi(8,5050, [α,β,ε])-vdi(10,5050, [α,β,ε]) | As in #1 |
| **21** | d[C50:IkBα,C50:IkBβ, C50:IkBε]/dt | vdi(1,C50, [α,β,ε])-vdi(3,C50, [α,β,ε])-vdi(5,C50, [α,β,ε])+vdi(6,C50, [α,β,ε])-vdi(7,C50, [α,β,ε])-vdi(8,C50, [α,β,ε])-vdi(10,c50, [α,β,ε]) | As in #1 |
| **22** | d[nAA:nIkBα,nAA:nIkBβ, nAA:nIkBε]/dt | vdi(2,AA, [α,β,ε])-vdi(4,AA, [α,β,ε])-vdi(6,AA, [α,β,ε])+vdi(5,AA, [α,β,ε])-vdi(9,AA, [α,β,ε])-vdi(11,AA, [α,β,ε]) | As in #1 |
| **23** | d[nA50:nIkBα, nA50:nIkBβ, nA50:nIkBε]/dt | vdi(2,A50, [α,β,ε])-vdi(4,A50, [α,β,ε])-vdi(6,A50, [α,β,ε])+vdi(5,A50, [α,β,ε])-vdi(9,A50, [α,β,ε])-vdi(11,A50, [α,β,ε]) | As in #1 |
| **24** | d[n5050:nIkBα, n5050:nIkBβ, n5050:nIkBε]/dt | vdi(2,5050, [α,β,ε])-vdi(4,5050, [α,β,ε])-vdi(6,5050, [α,β,ε])+vdi(5,5050, [α,β,ε])-vdi(9,5050, [α,β,ε])-vdi(11,5050, [α,β,ε]) | As in #1 |
| **25** | d[nA50:nIkBα, nA50:nIkBβ, nA50:nIkBε]/dt | vdi(2,C50, [α,β,ε])-vdi(4,C50, [α,β,ε])-vdi(6,C50, [α,β,ε])+vdi(5,C50, [α,β,ε])-vdi(9,C50, [α,β,ε])-vdi(11,C50, [α,β,ε]) | As in #1 |
| **26** | d[tIkbα,tIkBβ,tIkBε]/dt | vi([α,β,∈])- vti(1, [α,β,ε])[tIkbα,tIkBβ,tIkBε] | As in #1 |
| **27** | d[Ikbα,IkBβ,IkBε]/dt | Σd=[AA,A50,5050,C50]{vdi(3,d, [Ikbα,IkBβ,IkBε])+vdi(10,d, [Ikbα,IkBβ,IkBε])- vdi(1,d, [Ikbα,IkBβ,IkBε])}+vti(2, [α,β,ε])[tIkbα,tIkBβ,tIkBε]-vti(3, [α,β,ε])[Ikbα,IkBβ,IkBε]- vti(4, [α,β,ε])[Ikbα,IkBβ,IkBε]- vti(6, [α,β,ε])[nIkbα,nIkBβ,nIkBε]+ vti(7, [α,β,ε])[Ikbα,IkBβ,IkBε] | As in #1 |
| **#** | **Species** | **Reaction** | **Justification** |
| **28** | d[nIkbα,nIkBβ,nIkBε]/dt | Σd=[AA,A50,5050,C50]{vdi(4,d, [Ikbα,IkBβ,IkBε])+vdi(11,d, [Ikbα,IkBβ,IkBε])- vdi(2,d, [Ikbα,IkBβ,IkBε])}- vti(5, [α,β,ε])[nIkbα,nIkBβ,nIkBε]+ vti(6, [α,β,ε])[Ikbα,IkBβ,IkBε]- vti(7, [α,β,ε])[nIkbα,nIkBβ,nIkBε] | As in #1 |
| **29** | d[L]/dt | 0 | See ([Loriaux et al, 2013](#_ENREF_11)) |
| **30** | d[R]/dt | -va(1) + va(2) + va(71) - va(72) | As in #29 |
| **31** | d[L-R]/dt | va(1) - va(2) - va(3) | As in #29 |
| **32** | d[DISC]/dt | va(3) - va(4) + va(5) - va(6) + va(7) + va(8) | As in #29 |
| **33** | d[flip]/dt | -va(4) + va(5) + va(73) - va(74) | As in #29 |
| **34** | d[flip-DISC]/dt | va(4) - va(5) - va(75) | As in #29 |
| **35** | d[pC8]/dt | -va(6) + va(7) - va(17) + va(18) + va(76) - va(77) | As in #29 |
| **36** | d[DISC-pC8]/dt | va(6) - va(7) - va(8) | As in #29 |
| **37** | d[C8]/dt | va(8) - va(9) + va(10) - va(11) + va(12) + va(13) + va(19) - va(26) + va(27) + va(28) | As in #29 |
| **38** | d[Bar]/dt | -va(9) + va(10) + va(78) - va(79) | As in #29 |
| **39** | d[Bar-C8]/dt | va(9) - va(10) - va(80) | As in #29 |
| **40** | d[pC3]/dt | -va(11) + va(12) - va(62) + va(63) + va(101) - va(102) | As in #29 |
| **41** | d[C8-pC3]/dt | va(11) - va(12) - va(13) | As in #29 |
| **42** | d[C3]/dt | va(13) - va(14) + va(15) + va(16) - va(20) + va(21) - va(23) + va(24) + va(25) + va(64) | As in #29 |
| **43** | d[pC6]/dt | -va(14) + va(15) + va(104) - va(105) | As in #29 |
| **44** | d[C3-pC6]/dt | va(14) - va(15) - va(16) | As in #29 |
| **45** | d[C6]/dt | va(16) - va(17) + va(18) + va(19) - va(106) | As in #29 |
| **46** | d[C6-pC8]/dt | va(17) - va(18) - va(19) | As in #29 |
| **47** | d[XIAP]/dt | -va(20) + va(21) + va(22) - va(67) + va(68) - va(69) + va(70) + va(95) - va(96) | As in #29 |
| **48** | d[XIAP-C3]/dt | va(20) - va(21) - va(22) | As in #29 |
| **49** | d[PARP]/dt | -va(23) + va(24) + va(108) - va(109) | As in #29 |
| **50** | d[C3-PARP]/dt | va(23) - va(24) - va(25) | As in #29 |
| **51** | d[CPARP]/dt | va(25) - va(110) | As in #29 |
| **52** | d[Bid]/dt | -va(26) + va(27) + va(81) - va(82) | As in #29 |
| **53** | d[C8-Bid]/dt | va(26) - va(27) - va(28) | As in #29 |
| **54** | d[tBid]/dt | va(28) - va(29) + va(30) - va(31) + va(32) + va(33) | As in #29 |
| **55** | d[Mcl1]/dt | -va(29) + va(30) + va(83) - va(84) | As in #29 |
| **56** | d[Mcl1-tBid]/dt | + va(29) - va(30) - va(85) | As in #29 |
| **57** | d[Bax]/dt | -va(31) + va(32) + va(86) - va(87) | As in #29 |
| **58** | d[tBid-Bax]/dt | va(31) - va(32) - va(33) | As in #29 |
| **59** | d[act_Bax]/dt | va(33) - va(34) + va(35) | As in #29 |
| **60** | d[Baxm]/dt | va(34) - va(35) -1/mvol*va(36) + va(37) -1/mvol*2*va(38) +2*va(39) | As in #29 |
| **61** | d[Bcl2]/dt | -1/mvol*va(36) + va(37) -1/mvol*va(40) + va(41) -1/mvol*va(44) + va(45) + va(88) - va(89) | As in #29 |
| **62** | d[Baxm-Bcl2]/dt | 1/mvol*va(36) - va(37) - va(90) | As in #29 |
| **63** | d[Bax2]/dt | 1/mvol*va(38) - va(39) -1/mvol*va(40) + va(41) -2/mvol*va(42) +2*va(43) | As in #29 |
| **64** | d[Bax2-Bcl2]/dt | 1/mvol*va(40) - va(41) - va(91) | As in #29 |
| **65** | d[Bax4]/dt | 1/mvol*va(42) - va(43)-1/mvol*va(44) + va(45) -1/mvol*va(46) + va(47) | As in #29 |
| **66** | d[Bax4-Bcl2]/dt | 1/mvol*va(44) - va(45) - va(92) | As in #29 |
| **67** | d[M]/dt | -1/mvol*va(46) + va(47) + va(93) | As in #29 |
| **68** | d[Bax4-M]/dt | 1/mvol*va(46) - va(47) - va(48) | As in #29 |
| **69** | d[AMito]/dt | va(48) -1/mvol*va(49) + va(50) + va(51) -1/mvol*va(52) + va(53) + va(54) - va(93) | As in #29 |
| **70** | d[mCytoC]/dt | -1/mvol*va(49) + va(50) + va(113) - va(114) | As in #29 |
| **71** | d[AMito-mCytoC]/dt | 1/mvol*va(49) - va(50) - va(51) | As in #29 |
| **72** | d[ACytoC]/dt | va(51) - va(55) + va(56) | As in #29 |
| **73** | d[mSmac]/dt | -1/mvol*va(52) + va(53) + va(97) - va(98) | As in #29 |
| **74** | d[AMito-mSmac]/dt | 1/mvol*va(52) - va(53) - va(54) | As in #29 |
| **75** | d[ASmac]/dt | va(54) - va(65) + va(66) | As in #29 |
| **76** | d[CytoC]/dt | va(55) - va(56) - va(58) + va(59) + va(57) - va(115) | As in #29 |
| **77** | d[Apaf]/dt | -va(58) + va(59) + va(94) | As in #29 |
| **78** | d[Apaf-CytoC]/dt | va(58) - va(59) - va(57) | As in #29 |
| **79** | d[act_Apaf]/dt | va(57) - va(60) + va(61) - va(94) | As in #29 |
| **#** | **Species** | **Reaction** | **Justification** |
| **80** | d[pC9]/dt | -va(60) + va(61) | As in #29 |
| **81** | d[Apop]/dt | va(60) - va(61) - va(62) + va(63) + va(64) - va(67) + va(68) + va(107) | As in #29 |
| **82** | d[Apop-pC3]/dt | va(62) - va(63) - va(64) | As in #29 |
| **83** | d[cSmac]/dt | va(65) - va(66) - va(69) + va(70) - va(99) | As in #29 |
| **84** | d[Apop-XIAP]/dt | va(67) - va(68) - va(107) | As in #29 |
| **85** | d[cSmac-XIAP]/dt | va(69) - va(70) - va(100) | As in #29 |
| **86** | d[C3_Ub]/dt | va(22) - va(103) | As in #29 |
| **87** | d[CycA]/dt | vcc(36)+vcc(12)+vcc(18)-vcc(9)-vcc(6) | See ([Conradie et al, 2010](#_ENREF_3)) |
| **88** | d[CycB]/dt | vcc(42)-vcc(19) | As in #87 |
| **89** | d[CycD]/dt | vcc(39)+vcc(17)+vcc(8)-vcc(7)-vcc(4) | As in #87 |
| **90** | d[CycE]/dt | vcc(38)+vcc(11)+vcc(16)-vcc(14)-vcc(5) | As in #87 |
| **91** | d[mCycD]/dt | vcc(34)-vcc(1) | As in #87 |
| **92** | d[mBcl2]/dt | vcc(41)-vcc(2) | As in #87 |
| **93** | d[Cdh1]/dt | vcc(20)-vcc(21) | As in #87 |
| **94** | d[CA]/dt | vcc(6)-vcc(12)-vcc(18)-vcc(10) | As in #87 |
| **95** | d[CD]/dt | vcc(7)-vcc(8)-vcc(17)-vcc(3) | As in #87 |
| **96** | d[CDc20]/dt | vcc(26)-vcc(27)-vcc(28) | As in #87 |
| **97** | d[CDc20T]/dt | vcc(35)-vcc(25) | As in #87 |
| **98** | d[CE]/dt | vcc(5)-vcc(11)-vcc(13)-vcc(16) | As in #87 |
| **99** | d[GM]/dt | vcc(31)-vcc(32) | As in #87 |
| **100** | d[IEP]/dt | vcc(23)-vcc(24) | As in #87 |
| **101** | d[Mass]/dt | vcc(33)-vcc(55) | As in #87 |
| **102** | d[p27]/dt | vcc(40)+vcc(3)+vcc(8)-vcc(15)-vcc(5)-vcc(6)-vcc(7)+vcc(11)+vcc(12)+vcc(13)+vcc(10) | As in #87 |
| **103** | d[PPX]/dt | vcc(37)-vcc(22) | As in #87 |
| **104** | d[pp-Rb]/dt | vcc(29)+vcc(30)+vcc(43)-vcc(44) | As in #87 |
| **105** | d[E2F]/dt | vcc(29)+vcc(45)+vcc(47)-vcc(46)-vcc(48) | As in #87 |
| **106** | d[p-E2F]/dt | vcc(30)+vcc(49)+vcc(46)-vcc(47)-vcc(50) | As in #87 |
| **107** | d[Rb]/dt | vcc(44)+vcc(45)+vcc(49)-vcc(48)-vcc(50)-vcc(43) | As in #87 |
| **108** | d[E2F-Rb]/dt | vcc(51)+vcc(48)-vcc(52)-vcc(29)-vcc(45) | As in #87 |
| **109** | d[p-E2F-Rb]/dt | vcc(52)+vcc(50)-vcc(51)-vcc(30)-vcc(49) | As in #87 |
| **110** | d[MYC]/dt | vcc(53)-vcc(54) | As in #87 |
| **111** | d[mMYC]/dt | vcc(56)-vcc(57) | As in #87 |
| **112** | d[IKK*]/dt | [IKKt]*(0.5*[(t/50)^2/((t/50)^2+1)]*(1-[(t/IKK_Kd) /((t/ IKK_Kd) +1)] | Form generates typical IKK profile (see ([Alves et al, 2014](#_ENREF_2))) with parameters selected such that the nuclear NFkB output qualitatively matches results from experiments (([Lenert et al, 2001](#_ENREF_8)). |

ODE reactions describing the species rate of change are shown in terms of parameters and species. Some reactions were updated to reflect additional species that were introduced to model connections between the three models. Please see Text S1 and Tables S8,9 for more details.

# Table S7. Other simulation parameters

| **#** | **Parameter** | **Value** | **Description** | **Justification** |
| --- | --- | --- | --- | --- |
| **1** | DegRateCV | 0.1 | Protein degradation CV | For simplicity we assumed degradation rates were normally distributed with a CV of 0.1 about the parameter value. This is similar to the approach taken in ([Loriaux et al, 2013](#_ENREF_11)) and results a steady state protein concentration variability with CV ~ 0.25 ([Gaudet et al, 2012](#_ENREF_4)) |
| **2** | TotalProtCV | 0.25 | If no protein syn/deg then the total free protein abundance CV | We assumed log-normally distributed initial protein concentrations about the x_0_ mean with a CV of 0.25 as in ([Gaudet et al, 2012](#_ENREF_4)). This affected IKK, and Rb or E2F containing species in the model. |
| **3** | PosCV | 0.1 or 0.2 | CV of translation rate for NFkB monomers | See #1. |
| **4** | NegCV | 0.1 or 0.2 | CV of translation rate for IkBs | See #1. |
| **5** | NonCV | 0.1 or 0.2 | CV of translation rate for apoptosis and cell-cycle proteins | See #1. |
| **6** | PosX | 1 or 1.1 | Translation rate multiplier for NFkB monomers | Used to test modest increases to the positive regulators of NFkB signaling, the NFkB monomers (See Fig 8). |
| **7** | NegX | 1 or 1.1 | Translation rate multiplier for IkB proteins | Used to test modest increases to the negative regulators of NFkB signaling, the IkBs (See Fig 8). |
| **8** | NonX | 1 or 1.1 | Production rate multiplier for apoptosis and cell-cycle proteins | Used to test modest increases to the proteins participating in the cell-cycle and apoptosis (See Fig 8). Total Rb and E2F species were multiplied by 1.5 during the test since these cell-cycle related proteins are not produced or degraded. |
| **9** | PCV | 0.07 | Partition volume CV | The observed variability in relative volumes was normal with mean 1 and standard deviation of 0.07 from microscopy studies in this work |
| **10** | VCV | 0.05 | Starting volume CV | The observed starting volume variability of viable cells from microscopy studies. |
| **11** | N | 250 | Initial number of cellular agents used in multiscale model. | This number was large enough to ensure that repeated simulations produced very similar results. |
| **12** | Tmax | 144 h | Total simulation time. | Six days of simulations was sufficient for capturing both the expansion and contraction periods of the population response. This is also the typical duration of time-lapse experiments. |
| **13** | AbsTol | 1e-5 | Absolution simulation tolerance for Matlab function ode15s | This was sufficiently low to produce accurate results. Increasing this number further resulted in noticeable changes in solution accuracy. |
| **14** | RelTol | 1e-3 | Relative tolerationce of simulation s using Matlab function ode15s | As in #13 |

Parameters describing the population variance (extrinsic noise) within specific modules of the model, multipliers, and general simulations parameters are shown. For more details please refer to the provided Matlab simulation files (File S8).

# Table S8. List of constants in new model reactions

| **Const.** | **Value** | **Justification/Description** |
| --- | --- | --- |
| ***k_1_*** | 0.307 min^-1^ | Fitted such that steady-state [Bcl_XL_] equal to original model parameter (see Loriaux *et al*, 2013) |
| ***k_2_*** | 0.01155 min^-1^ | (Loriaux *et al*, 2013) |
| ***k_3_*** | 1 min^-1^ | Quadrupled to ensure that steady state [CycD] is sufficient to initiate cell cycle given Myc-dependent growth and added extrinsic noise |
| ***k_4_*** | 0.5 min^-1^ | (Conradie *et al*, 2010) |
| ***k_5_*** | 0.0231 min^-1^ | Experimentally determined 30 min half-life (see Jones and Cole, 1987) |
| ***p_e_*** | 1 | Global translation efficiency. We fit a lower value for the rapamycin treated condition. A value of 1 is used for all other conditions (Conradie *et al,* 2010) |
| ***k_norm_*** | 0.5 | Normalization of the maximum activity to ~ 0.45. Trained on EMSA time course (Lenert *et al*, 2001). |
| ***k_inc_*** | 0.02 min^-1^ | IKK initial activation constant, ensures maximal activity between 0.5 – 2 h. (Alves *et al,*  2014) |
| ***k*** | 2 | A Hill coefficient of 2 was used to ensure the observed sharp decay in IKK activity after initial stimulation (Alves *et al*, 2014). |
| ***IKK_tot_*** | 140 nM | (Alves *et al*, 2014) |
| ***k_other_*** | 0.1 | (Gerondakis *et al,* 2002) and biochemical experiments in this study. |
| ***k_A50_ Myc*** | 0.45 | RelA:p50 and cRel:p50 induction of Myc is assumed to be equal |
| ***k_A50_***  ***CycD*** | 0.45 | RelA:p50 and cRel:p50 induction of CycD is assumed to be approximately equal since we do not have evidence to the contrary |
| ***k_A50_ Bcl_XL_*** | 0.3 | cRel deficient cells have approximately 1/3 the Bcl_XL_ abundance, cRel:p50 results in roughly twice the induction strength for Bcl_XL_ |
| ***k_C50_ Myc*** | 0.45 | See kA50 Myc above |
| ***k_C50_***  ***CycD*** | 0.45 | See kA50 CycD above |
| ***k_C50_ Bcl_XL_*** | 0.6 | See kA50 Bcl_XL_ above |
| ***k_m_*** | 2e-6 nM min^-1^ | (Alves *et al*, 2014) |
| ***Kd*** | 150 nM | (Alves *et al,* 2014) |
| $\boldsymbol{f}_{\boldsymbol{RB}}$ | 0-1 | Fraction of inactive RB (Conradie *et al*, 2010) |
| ***k_b_*** | 0.01 | Basal promoter activity as in (Alves *et al*, 2014) |
| ***k_n_*** | 2 | Hill coefficient of 2 was necessary to ensure quiescence with basal IKK activation in our model. |
| ***k_Lsyn_*** | 0 | Since we did not stimulate apoptosis with a death ligand, we assumed constant [L] to mimic lack of survival signaling and to ensure observed Tdie distributions. Ligand binding to receptor in the apoptosis model was assumed to not deplete L |
| ***k_Ldeg_*** |  |  |

Constants present in novel model reactions (see Text S1). These values were derived from previously studies or from the datasets in this study. Note that *f_RB_* is a fraction that is depenent on the current abundance of specific Rb species in the cell-cycle module as described in the original model, included here to highlight that it was not a fitted quantity.

# Table S9. Parameterization of free parameters and sensitivity analysis

| **Param.** | **Range** | **Fitted** | **High CpG features fitting** | **Fitted Features missing** | **+10% Features missing** | **-10% Features missing** |
| --- | --- | --- | --- | --- | --- | --- |
| ***p_1_*** | 0.001-1 nM⋅min^-1^ | 0.005 | M,N,A,E,G0,X0, D0,G1,X1,D1,R0,R1,N0,N1,F0,F1, F3,FP |  | M,E,R1 | M,A,X1,F1,F3 |
| ***p_2_*** | 0.001-1 min^-1^ | 0.007 |  |  | M,A,E | M,E,N0,N1,R0,R1 |
| ***p_3_*** | 0.001-1 AU⋅min^-1^ | 0.003 |  |  | M,A,F3 | M,N,E |
| ***p_4_*** | 0.001-1 min^-1^ | 0.002 |  |  | M,E,N0,N1,R0,R1 | M,E |
| ***p_5_*** | 0.001-1 min^-1^ | 1 |  |  | M,E,N0,N1,R0,R1 | M,N,A,E,X1,F3 |
| ***p_6_*** | 0.001-1 min^-1^ | 0.004 |  |  | M,N,A,X1,F1,F3,R1 | E,R1 |
| ***p_7_*** | 0.001-1 min^-1^ | 0.002 |  |  | M,N,A,E,X0,D1,X1,F1,F3 | M,A,E,N0,N1, R0,R1 |
| ***p_8_*** | 0.001-1 AU⋅min^-1^ | 0.002 |  |  | M,A,E,N0,N1, R0,R1 | M,E |
| ***p_9_*** | 0.0001-0.1 min^-1^ | 0.0002 |  |  | M,F1 | A,R1 |
| ***p_10_*** | 0.001-1 min^-1^ | 0.004 |  |  | E,X0,N0,N1,R0,R1 | E,F1 |
| ***p_11_*** | 0.001-1 min^-1^ | 0.002 |  |  | M,A,E | A,E,F1,N0,N1, R0,R1 |
| ***p_x_*** | 10-100 nM | 40 |  |  | M,N,A,E,D1,X1,F1 | N,E |
| ***p_↑CpG_*** | 1000-10,000 min | 1950 |  |  | M,N,E,X1 | M,N,A,D1,X1, F1,F3,fp |
| ***c_GM_*** | 1-10 AU^-2^ | 2.8 |  |  | E,X1,F1,N0,N1,R0,R1 | A,E |
| ***d_GM_*** | 0.01-1 AU^-2^ | 0.14 |  |  | E | N,A,D1,X1,F1,F3,N0,N1,R0,R1 |
| **[L]** | 0.1-10 nM | 0.4 |  |  | N,A,E,X1,F0,F3 | E |
| ***fbck*** | 1-100 nM^-1^ | 10 |  |  | N,A,E,N0,N1,R0,R1 | A,E,N0,N1,R0,R1 |
| **Param.** | **Range** | **Fitted** | **Low CpG features fitting** | **Fitted Features missing** | **+10% Features missing** | **-10% Features missing** |
| ***p_↓CpG_*** | 1000-10,000 min | 1500 | m,n,a,e,G0,X0,D0,G1,X1,D1,R0,R1,N0,N1,F0,F1,f3 | F1,f3 | n,a,e,X1,F1,f3 | m,e,X0,D1,X1, F0,F1,R0,R1 |
| **Param.** | **Range** | **Fitted** | **Rapamycin features fitting** | **Fitted Features missing** | **+10% Features missing** | **-10% Features missing** |
| ***p_e_*** | 0-1 unitless | 0.7 | u,r,p,e,g0,x0,d0,R0,R1,N0,N1,F0 | F0 | e,F0,R1 | r,e,x0,F0,N0,N1,R0,R1 |

(Features are described in Table S10).

Free parameters that were part of novel model reactions (see above) were manually fitted according to biological features observed in microscopy datasets of this study. Ranges for values were selected to reflect known distributions for protein and mRNA half-lifes (~1 h to ~100 h) or values which allowed for experimentally observed IKK dynamics, Tdie distributions, NFkB monomer abundances, and observed population cell count trajectories.

# Table S10. Population response features being fitted during model parameterization

| **Name** | **Description** | **Fitting conditions** |
| --- | --- | --- |
| M | max total count 1.75-2.25 x starting count | WT 250 nM CpG |
| N | number of gens is 7+ | WT 250 nM CpG |
| A | avg. total count is 0.9-1.1 x starting count | WT 250 nM CpG |
| E | total surviving cells 0.1 to 0.5 x starting | WT 250 nM CpG |
| G0 | avg. gen 0 growth time is 5 – 15 h | WT 250 nM/10 nM CpG |
| X0 | avg. gen 0 death time is 20 – 30 h | WT 250 nM/10 nM CpG |
| D0 | avg. gen 0 division time is 25 – 50 h | WT 250 nM/10 nM CpG |
| G1 | average gen 1+ growth time is 0-5 h | WT 250 nM/10 nM CpG |
| X1 | average gen 1+ death time is 5-15 h | WT 250 nM/10 nM CpG |
| D1 | average gen 1+ division time is 7-13 h | WT 250 nM/10 nM CpG |
| R0 | gen 0 growers grow to a mass of 6-8 AU | WT 250 nM/10 nM CpG/WT 250 nM CpG + 1 ng/ml rapamycin pre-treatment |
| R1 | gen 1 growers grow to a mass of 6-8 AU | WT 250 nM/10 nM CpG/WT 250 nM CpG + 1 ng/ml rapamycin pre-treatment |
| N0 | gen 0 growers grow at most to 5 AU | WT 250 nM/10 nM CpG/WT 250 nM CpG + 1 ng/ml rapamycin pre-treatment |
| N1 | gen 1 growers grow at most to 5 AU | WT 250 nM/10 nM CpG/WT 250 nM CpG + 1 ng/ml rapamycin pre-treatment |
| F0 | 20%-40% of gen 0 cells dividing | WT 250 nM/10 nM CpG/WT 250 nM CpG + 1 ng/ml rapamycin pre-treatment |
| F1 | 60%-100% of gen 1 cells dividing | WT 250 nM/10 nM CpG |
| F3 | 40%-80% of gen 3 cells dividing | WT 250 nM CpG |
| FP | % of gen 0 cells growing then dying < 0.2 | WT 250 nM CpG |
| m | max total count 1.1-1.5 x starting count | WT 10 nM CpG |
| n | number of gens is < 5 | WT 10 nM CpG |
| a | avg. total count is 0.5-0.7 x starting count | WT 10 nM CpG |
| p | p = avg. total count is 0.3-0.5 x starting count | WT 10 nM CpG |
| e | total surviving cells < 0.1 x starting | WT 10 nM CpG |
| f3 | <30% of gen 3 cells dividing | WT 10 nM CpG |
| u | max total count < starting count | WT 250 nM CpG + 1 ng/ml rapamycin pre-treatment |
| r | number of gens is 3 | WT 250 nM CpG + 1 ng/ml rapamycin pre-treatment |
| p | avg. total count is 0.3-0.5 x starting count | WT 250 nM CpG + 1 ng/ml rapamycin pre-treatment |
| e | total surviving cells < 0.1 x starting | WT 250 nM CpG + 1 ng/ml rapamycin pre-treatment |
| x0 | avg. gen 0 death time is 30 – 50 h | WT 250 nM CpG + 1 ng/ml rapamycin pre-treatment |
| d0 | avg. gen 0 division time is 40 – 60 h | WT 250 nM CpG + 1 ng/ml rapamycin pre-treatment |

Description of features identified in the experimental datasets which were selected for manual fitting of model parameters (Table S9).

# Table S11. Evaluating model fitting to experimental WT, cRel KO, low CpG, and Rapamycin treated datasets.

| **Statistic** | **WT 250 nM CpG** | ***rel^-/-^* 250 nM CpG** | **WT 10 nM CpG** | **1 ng Rapamycin, 250 nM CpG** |
| --- | --- | --- | --- | --- |
| **Gen 0 grower growth trajectory correlation** | r-value: 0.997*** | r-value:0.992*** | r-value:0.997*** | r-value:0.994*** |
| **Gen 1 grower growth trajectory correlation** | r-value:0.998*** | r-value:0.999*** | r-value:0.998*** | r-value:0.951*** |
| **Gen 0 non-grower growth trajectory correlation** | r-value:0.731*** | r-value:0.995*** | r-value:0.955*** | r-value:0.906*** |
| **Gen 1 non-grower growth trajectory correlation** | r-value:0.902*** | r-value:0.860*** | r-value:0.780*** | r-value:0.739*** |
| **E[Tgro_0_] error** | -1.24 h | -0.20 h | 0.53 h | 10.69 h** |
| **E[Tdiv_0_] error** | -4.52 h* | 2.30 h | -5.72 h | 7.74 h* |
| **E[Tdie_0_] error** | 0.97 h | -2.67 h | -0.45 h | 12.48 h** |
| **Total Cell Trajectory Correlation** | r-value: 0.744*** | r-values 0.535*** | r-value 0.883*** | r-value0.963*** |
| **Total normalized cell count trajectory residuals plot** | r  t | r  t | r  t | r  t |

*- p-value < 0.05, significantly different mean, Welch’s T-test

**- p-value < 0.01, significantly different mean, Welch’s T-test

***- p-value < 10E-10, significant correlation 1-tailed T-test

A comparison of the model fit to experimental time-lapse microscopy datasets for WT high CpG, cRel deficient, WT low CpG, and rapamycin treated conditions. Various measuremens of similarity for average cell growth trajectories (Figure S7 B,C), average Tgro, Tdiv, and Tdie timing (Figure S7 D,E,F), and total cell trajectories (Figure 6) are shown.

**References**

Albeck JG, Burke JM, Spencer SL, Lauffenburger DA, Sorger PK (2008) Modeling a snap-action, variable-delay switch controlling extrinsic cell death. PLoS Biol 6: 2831 – 2852

Gilmore TD (2014) NF-kB Target Genes. Boston University, http://www.bu.edu/nf-kb/gene-resources/target-genes/, Vol. 2014

Jones TR, Cole MD (1987) Rapid cytoplasmic turnover of c-myc mRNA: requirement of the 30 untranslated sequences. Mol Cell Biol 7: 4513 – 4521

Lenert P, Stunz L, Yi AK, Krieg AM, Ashman RF (2001) CpG stimulation of primary mouse B cells is blocked by inhibitory oligodeoxyribonucleotides at a site proximal to NF-kappaB activation. Antisense Nucleic Acid Drug Dev 11: 247 – 256

Limon JJ, Fruman DA (2012) Akt and mTOR in B cell activation and differentiation. Front Immunol 3: 228

Nie Z, Hu G, Wei G, Cui K, Yamane A, Resch W, Wang R, Green DR, Tessarollo L, Casellas R, Zhao K, Levens D (2012) c-Myc is a universal amplifier of expressed genes in lymphocytes and embryonic stem cells. Cell 151: 68 – 79

Piatelli M, Tanguay D, Rothstein T, Chiles T (2003) Cell cycle control mechanisms in B-1 and B-2 lymphoid subsets. Immunol Res 27: 31 – 52

Shih VF-S, Kearns JD, Basak S, Savinova OV, Ghosh G, Hoffmann A (2009) Kinetic control of negative feedback regulators of NF-jB/RelA determines their pathogen- and cytokine-receptor signaling specificity. Proc Natl Acad Sci USA 106: 9619 – 9624

Werner SL, Kearns JD, Zadorozhnaya V, Lynch C, O’Dea E, Boldin MP, Ma A, Baltimore D, Hoffmann A (2008) Encoding NF-kappaB temporal control in response to TNF: distinct roles for the negative regulators IkappaBalpha and A20. Genes Dev 22: 2093 – 2101

Zeng H, Chi H (2013) mTOR and lymphocyte metabolism. Curr Opin Immunol 25: 347 – 355
