# Supplementary material for: A multi-scale approach reveals that NF-κB cRel enforces a B-cell decision to divide
Source: Mol Syst Biol. 2015 Feb 13;11(2):783. doi: 10.15252/msb.20145554 (PMC4358656; doi:10.15252/msb.20145554)
Supplement: Supplementary file 10 [file msb0011-0783-sd10.docx]

**Supplementary Methods**

Maxim N. Shokhirev, Jonathan Almaden, Jeremy Davis-Turak, Harry A. Birnbaum, Theresa M. Russell, Jesse A.D. Vargas, Alexander Hoffmann. **“**A multi-scale approach reveals that NFκB biases a B-cell fate race between division and death.”

This text provides further details into the methodology and analyses described in the main text of this study in the form of supplementary methods descriptions.

All supplementary tables are provided separately. Corresponding computational tools and source code are available as supplementary files. Time-lapse image datasets are available upon request, while the tracking results are attached as extended view movies.

**Table of Contents**

[Calculating the expected probability that a dying cell would have started growing 2](#_Toc403356710)

[Multi-scale modeling 6](#_Toc403356711)

[Description 6](#_Toc403356712)

[Model construction 6](#_Toc403356713)

[Graphical summary of the novel reactions connecting extant NFκB, apoptosis, and cell-cycle ODE models. 10](#_Toc403356714)

[Model fitting procedure 11](#_Toc403356715)

[Parameter sensitivity analysis 11](#_Toc403356716)

[Calculating expected number of progenitor divisions 11](#_Toc403356717)

[References 12](#_Toc403356718)

# Calculating the expected probability that a dying cell would have started growing

In order to test if the molecular race (Figure 3A) and/or the molecular decision (Figure 3B) hypotheses are consistent with our datasets, we used the observed $T_{grow0}^{0}(t)$,$T_{div0}^{0}(t)$, and $T_{die0}^{0}(t)$distributions (Figure 3E), designed with a superscript “o”, to determine a lower bound on the fraction of dying cells that are expected to grow under the race or decision hypothesis. While under the decision hypothesis we would expect that all responding cells are subject only to eventual division, while non-responders will not grow or divided, the fraction of growing cells that will die, or vise-versa are not readily apparent. Therefore, we derived a lower limit on the expected probability that a dying ($fate=death$) cell will grow under the race hypothesis in terms of observed distributions, and show that the observed probability of this happening is inconsistent. We are interested in the probability that progenitors (generation 0) cells started growing and died:

| $\Pr(T_{grow0}<T_{die0},F_{0}\vert fate=death)$, | (1) |
| --- | --- |

where $T_{die0}$and $T_{grow0}$are the true probability distributions for the time to die or start growing, and$F_{0}$is the true fraction of cells that respond to the stimulus and start growing (still subject to death) in the race model. We define the probability censoring of some probability distribution$f(t)$ by a competing probability distribution $g(t)$as:

| $f(t\vert g(t))=\frac{f(t)\left(1-\int_{-\infty}^{t} g(t')dt'\right)}{\int_{-\infty}^{\infty} f(t)\left(1-\int_{-\infty}^{t} g(t')dt'\right)dt}=f(t)\left(1-\int_{-\infty}^{t} g(t')dt'\right)w_{f\vert g}$, | (2) |
| --- | --- |

where $f(t|g(t))$represents$f(t)$ given that a competing mutually explosive event has probability distribution$g(t)$, and $w_{f|g}$ is a rescaling constant. From this definition it follows that

| $\int_{-\infty}^{t} f(t\vert g(t))dt\geq\int_{-\infty}^{t} f(t)dt,\forall t,\forall g(t)$. | (3) |
| --- | --- |

Furthermore, since under the race hypothesis, all cells are subject to age-dependent death, $T_{die0}(t)$, and since $T_{die0}(t)$is typically earlier than $T_{div0}(t)$, then for each cell that divided, there must have been at least another cell that would have divided but died, or in terms of the fraction of observed generation 0 responders:

| $2F_{0}^{o}\leq F_{0}$. | (4) |
| --- | --- |

Using (3) the observed $T_{grow0}(t)$,$T_{div0}(t)$, and $T_{die0}(t)$distributions are biased toward earlier values, since death will censor decision and division fates, while division will censor late death fates:

| $\int_{-\infty}^{t} T_{grow0}^{o}(t)dt\geq\int_{-\infty}^{t} T_{grow0}(t)dt,\forall t$,  $\int_{-\infty}^{t} T_{die0}^{o}(t)dt\geq\int_{-\infty}^{t} T_{die0}(t)dt,\forall t$. | (5) |
| --- | --- |

However, of the cells for which both division and death are running in parallel (at least twice as many as had been observed to divide from equation (4)) a fraction would not have started measurably growing prior to dying (i.e. died before growth decision was reached and observed). Therefore, to calculate the true fraction of dying cells that are expected to start growing we first calculated the probability that a cell will start measurably growing prior to dying:

| $\Pr(T_{grow0}<T_{die0})=\int_{-\infty}^{\infty} T_{grow0}(t)\left(1-\int_{-\infty}^{t} T_{die0}(t')dt'\right)dt$. | (6) |
| --- | --- |

To do this, we used (5) to show that

| $\Pr(T_{grow0}<T_{die0})\geq\Pr(T_{grow0}<T_{die0}^{o})$. | (7) |
| --- | --- |

Furthermore, we can define a new function $T_{grow0}^{*}(t)$ in terms of observed distributions:

| $T_{grow0}^{o}(t)=T_{grow0}^{*}(t\vert T_{die0}^{o}(t))$. | (8) |
| --- | --- |

Since by definition of function censorship:

| $T_{grow0}^{o}(t)=T_{grow0}(t\vert T_{die0}(t))$. | (9) |
| --- | --- |

and since from (5) the $T_{die0}^{o}(t)$distribution is shifted toward lower values compare to $T_{die0}(t)$, it follows that $T_{grow0}^{*}(t)$is typically shifted toward later values when compared to the true $T_{grow0}(t)$:

| $\int_{-\infty}^{t} T_{grow0}^{*}(t)dt\leq\int_{-\infty}^{t} T_{grow0}(t)dt,\forall t$. | (10) |
| --- | --- |

Therefore, from eq. (7) and (10) we can establish a lower bound on $\Pr(T_{grow0}<T_{die0})$:

| $\Pr(T_{grow0}<T_{die0})\geq\Pr(T_{grow0}<T_{die0}^{o})\geq\Pr(T_{grow0}^{*}<T_{die0}^{o})$. | (11) |
| --- | --- |

Since both $T_{grow0}^{*}(t)$, and $T_{die0}^{o}(t)$are defined in terms of observed continuous probability functions, we next developed an expression for calculating $\Pr(T_{grow0}^{*}<T_{die0}^{o})$using a collection of discrete observations. Specifically, to calculate $cdf_{grow0}^{*}(t)$from $cdf_{grow0}^{o}(t)$we reweighed the contribution of each, i^th^, observed $T_{grow0}$ value according to the observed$cdf_{die0}^{o}$:

| $cdf_{grow0}^{*}(T_{grow0,i})=\frac{cdf_{grow0}^{o}(T_{grow0,i})\cdot w_{i}}{\sum_{i}^{m} w_{i}}$,  $w_{i}=\frac{1}{1-cdf_{die0}^{o}(T_{grow0,i})}$. | (12) |
| --- | --- |

Finally, we used (12) with (4) to obtain an expression on the lower bound for (1) given m observed $T_{grow0}$ values, and n observed $T_{die0}$values:

| $\begin{matrix} \Pr(T_{grow0}<T_{die0},F_{0}\vert fate=death)\geq2F_{0}^{o}\Pr(T_{grow0}^{*}<T_{die0}^{0})= \\ 2F_{0}^{o}\sum_{i}^{m} \left[\frac{\sum_{j}^{n} I\left(T_{grow,i}<T_{die,j}\right)k_{i}}{Z}\right],Z=\sum_{i}^{m} k_{i} \end{matrix}$. | (13) |
| --- | --- |

In essence, we used the observed $T_{die0}$distribution to obtain a $T_{grow0}$ distribution that is guaranteed to be equal to or more biased toward later values than the true $T_{grow0}$ distribution. Combined with the fact that under the race hypothesis, the observed fraction of dividing cells is at least twice as small as the true fraction of responders, this allowed us to calculate a lower bound on the probability that a dying cell would start growing prior to actually dying (Figure 3F). Under the molecular decision model, cells commit to either division or death early, and are therefore protected from the alternative (growth and division for cells committed to death, and death for cells committed to growth and division). The fraction of dying cells that are expected to be also growing under the molecular decision hypothesis is zero. To test which hypothesis is best supported, we measured the fraction of dying generation 0 cells that were growing at the time of death defined as:

| $Growing _{i}=\left\{\begin{matrix} true, & V(i,t_{\max})>V_{0}(i)+350\mu m\vert V(i,t_{\max})>800\mu m \\ false, & otherwise \end{matrix}\right.$, | (14) |
| --- | --- |

where $V(i,t_{\max})$ and $V_{0}(i)$are the average final 30 volumes and the average first 30 volume measurements for cell i, respectively.

# Multi-scale modeling

In the following sections we describe our approach to constructing the multi-scale model, the model parameterization method, basic sensitivity analysis as well as a full list of multi-scale model species, parameters, and reactions. In addition, the Matlab files used to solve the multi-scale model for a population of WT cells are provided as Supplementary File S8.

## Description

The multi-scale model is a collection of independent ODE models that are solved concurrenly given randomized initial starting concentrations or synthesis/degradation rate constants (extrinsic noise). The bookkeeping for each model is stored in a single Matlab structures and each includes all of the details required to run the model as well as the solved species concentrations through time, time at start of simulations (birth time), the generation the virtual cell belongs to and the “fate” of the virtual b-cell. Together, these records are refered to as a “cellular agent.” Simulations are carried out one generation at a time using the Matlab ode15s solver until specific stopping conditions are met (division, death, or end of simulation). For each generation, cellular agents keep track of their birth time and death/division time and statistics are collected and reported at the end of the simulations.

## Model construction

We wanted to capture the necessary details required to model NFkB signaling, cell-cycle progression and growth, and apoptosis with the multi-scale model. To do this, we selected previously published well-established models for B-cell specific NFkB signaling ([Alves et al, 2014](#_ENREF_1)), general mammalian cell cycle progression ([Conradie et al, 2010](#_ENREF_3)), and mammalian apoptosis ([Loriaux et al, 2013](#_ENREF_5)). These models are relatively complex involving dozens of species and hundreds of parameters each, therefore we sought to integrate these models by adding the minimum set of connections required to model NFkB-dependent control of survival and cell-cycle progression, while keeping the vast majority of the species and parameters as is (See Table S9 and S10 for a list of constants and fitted parameters involved in the connecting reactions described in the following sections).

To do this, we took advantage of the fact that the cell-cycle model incorporated reactions for transcriptional control of CyclinD via reaction involving “early response genes” and “delayed response genes,” which served to inactivate Rb and lead to the activation of E2F transcription factors. Previous studies have shown that CyclinD3 is an NFkB target gene ([Wang et al, 1996](#_ENREF_8)), and that it is required for B cell proliferation ([Cato et al, 2011](#_ENREF_2)). Therefore, we removed the early and delayed response gene species from the model, and instead added a CyclinD transcript species under the control of a Hill-promoter:

| $\begin{matrix} \frac{d[tCycD]}{dt}=P_{CycD}p_{CycDsyn}-[tCycD]p_{CycD\deg} \\ P_{CycD}=\left(1-k_{b}\right)\frac{\left(\frac{k_{A50}[A50_{n}]+k_{C50}[C50_{n}]+k_{other}[IKK]}{p_{x}}\right)^{k_{n}}}{1+\left(\frac{k_{A50}[A50_{n}]+k_{C50}[C50_{n}]+k_{other}[IKK]}{p_{x}}\right)^{k_{n}}}+k_{b} \end{matrix}$, | (15) |
| --- | --- |

where $p_{CycDsyn}$, $p_{CycD\deg}$,$k_{b}$,$k_{A50}$,$k_{C50}$,$k_{other}$,$p_{x}$,and$k_{n}$are parameters descrbing the transcript synthesis rate, transcript degradation rate, basal fractional activity of the promoter, the RelA:p50 induction strength, the cRel:p50 induction strength, the induction strength of other non-modeled transcription factors, the half-maximal activation concentration, and the hill constant governing the sigmoidality of the response, respectively. In addition, since NFkB also affects the abundance of anti-apoptotic Bcl factors and the Myc, we assumed similar Hill-like kinetics of activation for the tBcl_XL_ and tMyc model species:

| $\begin{matrix} \frac{d[X]}{dt}_{\begin{matrix} X=tCycD, \\ tBclXL, \\ tMyc \end{matrix}}=P_{X}p_{X}-[X]p_{X} \\ P_{X}=\left(1-k_{b}\right)\frac{\left(\frac{k_{A50,X}[A50_{n}]+k_{C50,X}[C50_{n}]+k_{other}[IKK]}{p_{x}}\right)^{k_{n}}}{1+\left(\frac{k_{A50,X}[A50_{n}]+k_{C50},X[C50_{n}]+k_{other}[IKK]}{p_{x}}\right)^{k_{n}}}+k_{b} \end{matrix}$, | (16) |
| --- | --- |

where the $k_{A50,X}$and $k_{C50,X}$parameters were unique to each promoter and determined the relative activation strength of relA:p50, cRel:p50, and other transcription factors. For simplicity, we assumed $p_{x}$was the same for all promoters, fitting this value to 40 nM.

Next, we modified the equations governing the abundance of cellular general machinery, GM in the model. In the extant model ([Conradie et al, 2010](#_ENREF_3)), cell growth is assumed to be exponential an unbounded since the growth of cell Mass was proportional to the amount of the general machinery species, while the growth of general machinery was proportional to the cell mass. In our experiments, the growth rate is more complex, with generation 0 cells exhibiting delayed growth, and a decrease in growth rate in the penultimate generation. Furthermore, we notice that the final cell volume is relatively fixed despite high variability in the interdivision time (Figure 2D). Therefore, we modified the synthesis rate of general machinery to be non-linearly dependent on the cell mass. Furthermore, since the synthesis of general machinery required for growth is governed by global metabolic and transcriptional regulators such as Myc and mTOR, we assumed that synthesis of general machinery is dependent on the abundance of Myc, a global regulator of transcriptional activation ([Nie et al, 2012](#_ENREF_6); [Wang et al, 2011](#_ENREF_7)) in a Hill-like fashion. Thus, the final equation governing the GM synthesis rate,$f_{GM}$, becomes:

| $f_{GM}=\left\{\begin{matrix} p_{GM}\left[\begin{matrix} \left(1-k_{b}\right)\left(\frac{\left([Myc]/p_{x}\right)^{2}}{1+\left([Myc]/p_{x}\right)^{2}}\right)\cdot\\ \left(c_{GM}[Mass]^{2}e^{-d_{GM}[Mass]^{2}}\right)+k_{b} \end{matrix}\right] & ,f_{RB}<0.8 \\ k_{b}p_{GM} & ,f_{RB}\geq0.8 \end{matrix}\right.$, | (17) |
| --- | --- |

where, $k_{b}$,$p_{x}$,$c_{GM}$,$d_{GM}$,and$p_{GM}$are the basal transcription rate, half-activation concentration, initial growth rate modifier, diminition growth modifier, and GM synthesis rate.$f_{RB}$is the fraction of activated Rb protein in the model ([Conradie et al, 2010](#_ENREF_3)).

To connect the apoptosis module to NFκB signaling, we assumed Hill-like promoter kinetics for a novel Bcl_XL_ transcript species, since the original model did not model transcription explicitly. To stay true to the original model Bcl_XL_ production rates, we fixed the translation rates such that steady-state Bcl_XL_ abundance was maintained for basal signaling conditions.

In addition, the apoptosis model included the “death” ligand species, L, which was used to simulate the addition of TRAIL, resulting in activation of the DISC complex and eventual death ([Loriaux et al, 2013](#_ENREF_5)). Since we did not activate the death pathway in our experiments, and since unstimulated B cells died within several days without activation of survival pathways (such as NFκB), we simulated tonic activation of the death pathway by removing fluxes that produced, degraded or bound L in the model, while providing an small initial amount of L. In effect, this resulted in an infinite pool of the death-inducing stimulus leaving us to parameterize the exact concentration used in the models to produce the desired Tdie distribution and population count trajectories.

Finally, in the original NFkB signaling model, the abundance of active IKK is defined using an input curve table ([Alves et al, 2014](#_ENREF_1)). This can lead to over-fitting as the shape of the activation curve dictates NFκB dynamics. Instead, we decided to model the IKK input curve as a combination of Hill equations, with the first term dictating the fast increase in IKK activation after stimulation, and the second term determining the long-term decay of activity (presumably due to turnover of promoter-proximal species):

| $IKK*(t)=IKK_{tot}k_{norm}\left(\frac{\left[k_{inc}t\right]^{k}}{1+\left[k_{inc}t\right]^{k}}\right)\left(1-\frac{\left[\frac{t}{p_{\downarrow CpG}\vert p_{\uparrow CpG}}\right]^{k}}{1+\left[\frac{t}{p_{\downarrow CpG}\vert p_{\uparrow CpG}}\right]^{k}}\right)$, | (18) |
| --- | --- |

where $IKK_{tot}$,$k_{norm}$,$k_{inc}$,$p_{\downarrow CpG}$/$p_{\uparrow CpG}$, and$k$are the total IKK pool, a normalization constant set to ensure ~50% maximal activation of IKK, initial activation rate set to ensure ~ 0.5 -2 h maximal activity, the decay rate parameter fitted separately for high low CpG and high CpG conditions, and the Hill coefficient ensuring sufficient sigmoidality to reproduce the general shape of the curve. To determine the overall shape and magnitude of the curve, we fitted this model of IKK activation to a published 40 h time-course of DNA-bound NFkB abundance ([Lenert et al, 2001](#_ENREF_4)), and manually calibrated the $p_{\downarrow CpG}$/$p_{\uparrow CpG}$ parameters to fit the time-lapse microscopy datasets in this study.

## Graphical summary of the novel reactions connecting extant NFκB, apoptosis, and cell-cycle ODE models.

Constants that were obstained from literature or derived from experiments are highlighted red (see Table S8). Fitted model parameters are highlighted blue (see Table S9).

**
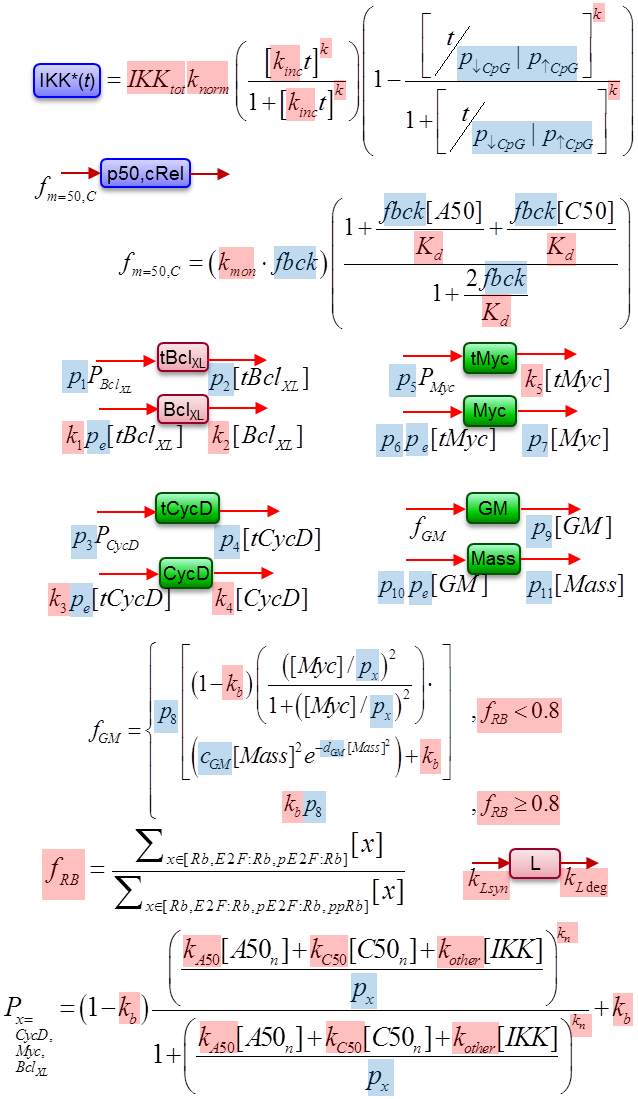

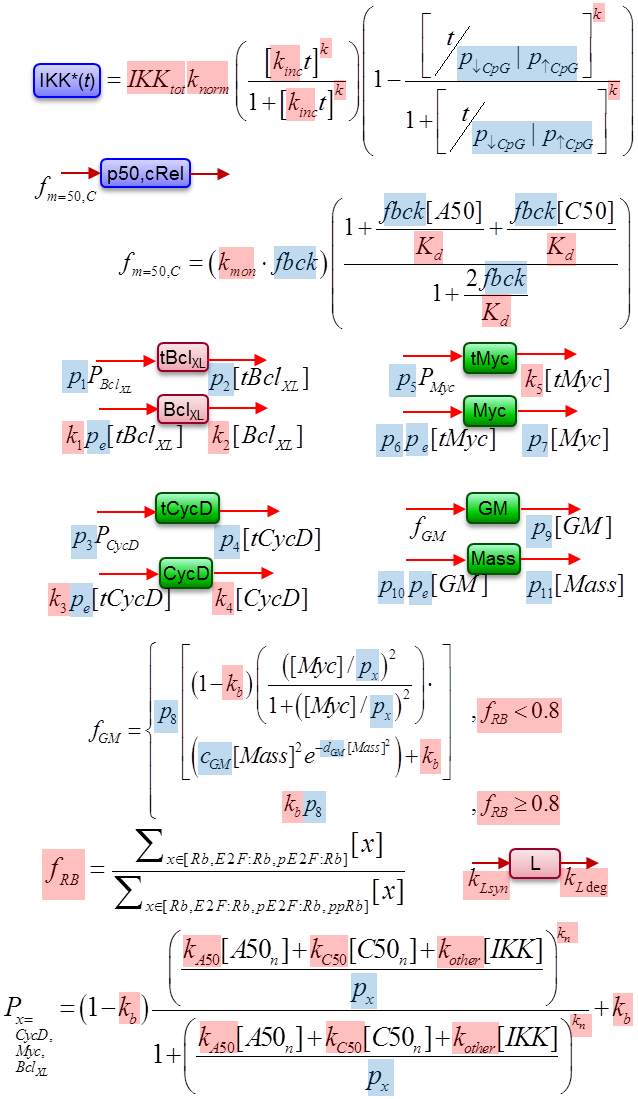

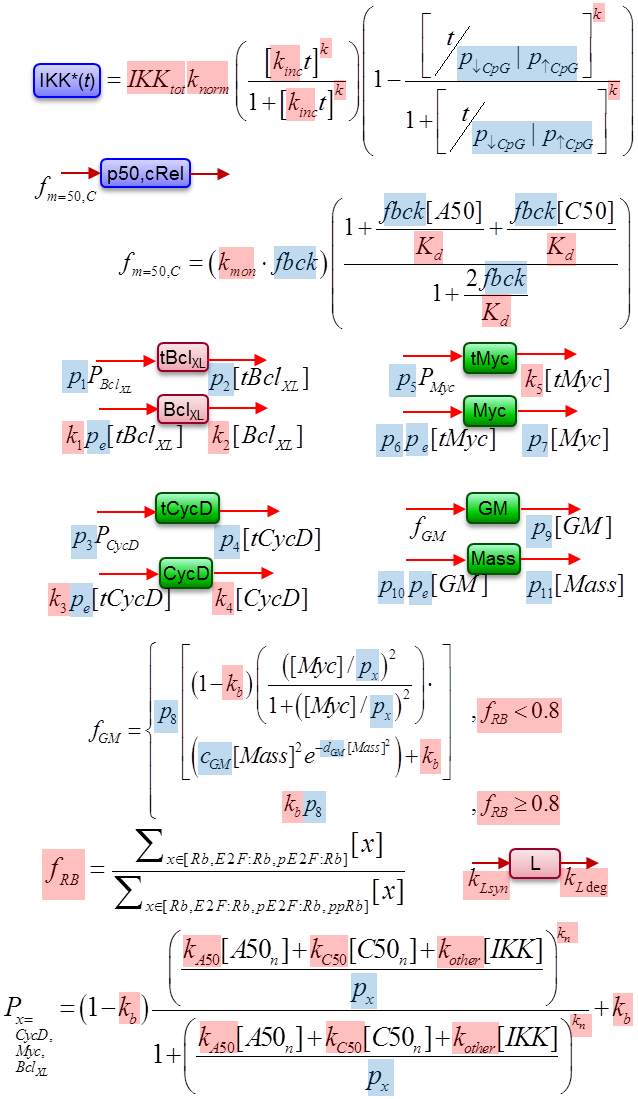

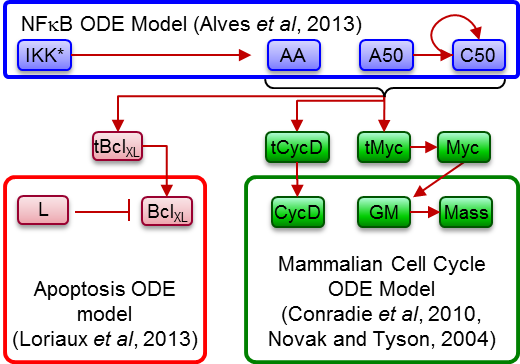
**

**
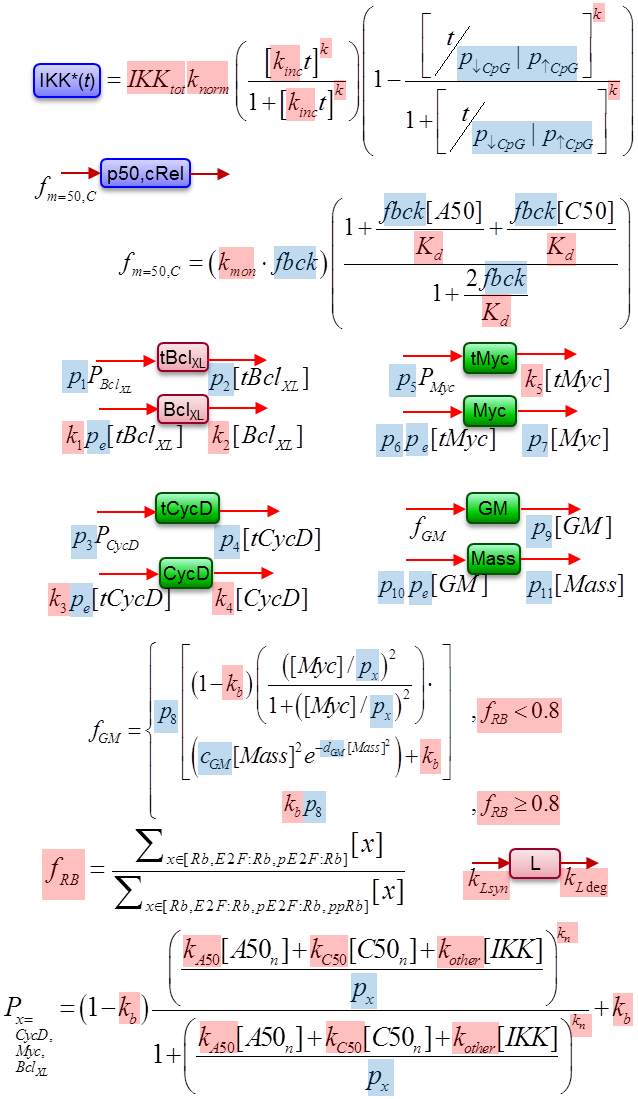
**

# Model fitting procedure

Since running the multi-scale model required performing thousands of individual ODE simulations, it was not feasible to attempt an unbiased fitting for the 19 separate free parameters (highlighted blue above and listed in Table S9. Instead, we identified features of the WT high CpG time-lapse microscopy dataset we felt were important to capture with the modeling (Table S10), and proceeded to manually change the parameters until all model features were captured. Out of the 19 parameters,$p_{e}$, and $p_{\downarrow CpG}$ were fitted to the rapamycin treated or low CpG condition, respectively. The remainng 17 parameters were fitted using the WT high CpG time-lapse microscopy dataset. It is important to note, that the vast majority of model parameters were kept exactly as published previously or set to reflect experimental observations (see also Table S8).

# Parameter sensitivity analysis

To estimate the sensitivity of the model fitting to the free parameters, we repeated the simulations with each parameter adjusted by 10% up or down from the fitted value and evaluated the population features (Table S10) that were no longer captured by the model fitting (Table S9). We defined a separate set of features for the low CpG and rapamycin pre-treated datasets analogously to the features defined for the WT high CpG condition.

# Calculating expected number of progenitor divisions

An important metric of population expansion is the expected number of divisions generation 0 (progenitor) cells undergo, which describes the proliferative capacity of naïve cells. Since this number is a function of the fates of daughter cells, and their daughter cells, and etc. the number of cells in each division must by multiplied by $2^{n}$. In addition, since $1-f_{0}$ of all progenitors are expect to remainin in generation 0, the progenitor division number is an average of the $f_{0}$* {the population average division number} and 0. Therefore, the average number of progenitor divisions becomes:

| $P_{div\#}=\frac{f_{0}\left[\frac{f_{0}\left(\sum_{i=1}^{g-1} i\left(\prod_{j=0}^{i} 2f_{j}\right)+2g(1-f_{g-1})\prod_{j=0}^{g-1} 2f_{j}\right)}{f_{0}\left(\sum_{i=1}^{g-1} \left(\prod_{j=0}^{i} 2f_{j}\right)+2(1-f_{g-1})\prod_{j=0}^{g-1} 2f_{j}\right)}\right]+(1-f_{0})(0)}{2}$ , | (19) |
| --- | --- |

where $f_{i}$ is the fraction of cells progressing to generation i+1, and $g$ is the total number of generations quantified. This number is compared between various simulations in figure 8E,I.

# References

Alves BN, Tsui R, Almaden J, Shokhirev MN, Davis-Turak J, Fujimoto J, Birnbaum H, Ponomarenko J, Hoffmann A (2014) IkappaB{varepsilon} Is a Key Regulator of B Cell Expansion by Providing Negative Feedback on cRel and RelA in a Stimulus-Specific Manner. *J Immunol* **192:** 3121-3132

Cato MH, Chintalapati SK, Yau IW, Omori SA, Rickert RC (2011) Cyclin D3 is selectively required for proliferative expansion of germinal center B cells. *Mol Cell Biol* **31:** 127-137

Conradie R, Bruggeman FJ, Ciliberto A, Csikasz-Nagy A, Novak B, Westerhoff HV, Snoep JL (2010) Restriction point control of the mammalian cell cycle via the cyclin E/Cdk2:p27 complex. *The FEBS journal* **277:** 357-367

Lenert P, Stunz L, Yi AK, Krieg AM, Ashman RF (2001) CpG stimulation of primary mouse B cells is blocked by inhibitory oligodeoxyribonucleotides at a site proximal to NF-kappaB activation. *Antisense & nucleic acid drug development* **11:** 247-256

Loriaux PM, Tesler G, Hoffmann A (2013) Characterizing the relationship between steady state and response using analytical expressions for the steady states of mass action models. *PLoS computational biology* **9:** e1002901

Nie Z, Hu G, Wei G, Cui K, Yamane A, Resch W, Wang R, Green DR, Tessarollo L, Casellas R, Zhao K, Levens D (2012) c-Myc is a universal amplifier of expressed genes in lymphocytes and embryonic stem cells. *Cell* **151:** 68-79

Wang R, Dillon CP, Shi LZ, Milasta S, Carter R, Finkelstein D, McCormick LL, Fitzgerald P, Chi H, Munger J, Green DR (2011) The transcription factor Myc controls metabolic reprogramming upon T lymphocyte activation. *Immunity* **35:** 871-882

Wang Z, Sicinski P, Weinberg RA, Zhang Y, Ravid K (1996) Characterization of the mouse cyclin D3 gene: exon/intron organization and promoter activity. *Genomics* **35:** 156-163
