# Supplementary material for: A multi-scale approach reveals that NF-κB cRel enforces a B-cell decision to divide
Source: Mol Syst Biol. 2015 Feb 13;11(2):783. doi: 10.15252/msb.20145554 (PMC4358656; doi:10.15252/msb.20145554)
Supplement: Supplementary file 12 [file msb0011-0783-sd12.zip › FAST Cheat Sheet.pdf]

FAST Cheat Sheet

The Force Algorithm Semi-automated Tracker is used to track roughly-circular cells in brightfield images using a semi-automated tracking approach. The user models each cell with a polygon across space and time (the image stack). Since cells can appear dark or bright in brightfield images, FAST uses the Sobel transform to find edges in the image, and a modified circular Hough transform to identify the approximate location and size of a cell. Keys are:

Ctrl+ Right drag or I, J, K, L: move the image around the working area.

Mouse wheel or U,P: zoom in and out (be careful not to lose track of your image)

Double right click: create a new cell in this image (the tool will try to predict location and size).

Shift+Double right click: create a placeholder cell (this "cell" will not be included in the statistics reported but otherwise acts as a regular cell). This is important to make sure that debris, or cells that drift in later are accounted for and do not interfere with the tracking of real cells.

Left click inside of a cell: select the cell

Left click on a waypoint: select the cell with the waypoint

Shift+Left click on a waypoint: switch paths after this point in time (the current path essentially "takes over" the clicked path).

Left click outside of a cell with a cell selected: place a waypoint at this location in the current image (this is your best estimate of where the cell is currently located in the image) The waypoint center will be marked with a dot, and lines will lead to the centers of the waypoint before and after (if any).

Ctrl+Shift+C: the selected cell becomes a placeholder cell (be careful as all of its daughters and lineage will also be converted to placeholder cells!)

Delete: delete the current waypoint and all waypoints afterward in the path

Shift+Delete: delete the whole cell path!

Shift+Ctrl+two right clicks: the selected cell divided into two daughter cells with centers at the two spots clicked by the user. The old cell's state is set to "divided" any further waypoints are deleted, and the first daughter is selected. During automated tracking each daughter is assumed to start with about half the size of the mother cell.

Shift+x: the selected cell died at this frame. It's marked as "dead" and no longer tries to maintain its size during automated tracking (after this frame). Its important to keep tracking the cell after it dies, as the debris may still interfere with other living cells in the vicinity.

Shift+z: the selected cell left at this frame. It's marked as "left" and is no longer tracked. It will also not be counted in the statistics and only tracked up until this point during automated tracking.

Space: go back to frame 0

A,D: traverse one frame at a time (forward and backward)

Shift+A,D: traverse 100 frames at a time

Q,E: traverse 5 frames at a time (forward and backward)

Shift+Q,E: traverse 20 frames at a time

W,S: traverse to next waypoint time or last waypoint time

F: select cell by id.

Shift+R: run the automatic optimization tracking. All cells will now be tracked simultaneously given the waypoint paths laid out by the user.

Shift+Ctrl+R: run the automatic optimization, then save all images and parameters to the specified folder. You have to specify the frame before which division and death cannot occur (cells which divide or die before this frame will not be counted in the analysis and this should be specified carefully based on your understanding of the imaging conditions and inherent biology. For example, CpG stimulated B cells had a 12 h period of mechanical death, followed by another peak of death after 12 h (biological death).

- There are buttons for saving and loading the cells, images, and statistics under the file tab on the right. Use this to save your progress or to re-run an analysis.
- There is also a Parameters tab that you can play with to change the various magnitudes of forces being applied to the cells during automatic tracking. It is recommended to try this with a few cells and a smaller section of the images (for example a 300x300 square with about 20 -30 cells)
- The last tab pane shows lists of cells grouped by their status (placeholder, unknown, died, divided), and can be selected individually.
