# Supplementary figures and images for: A multi-scale approach reveals that NF-κB cRel enforces a B-cell decision to divide (part 3 of 3)
Source: Mol Syst Biol. 2015 Feb 13;11(2):783. doi: 10.15252/msb.20145554 (PMC4358656; doi:10.15252/msb.20145554)

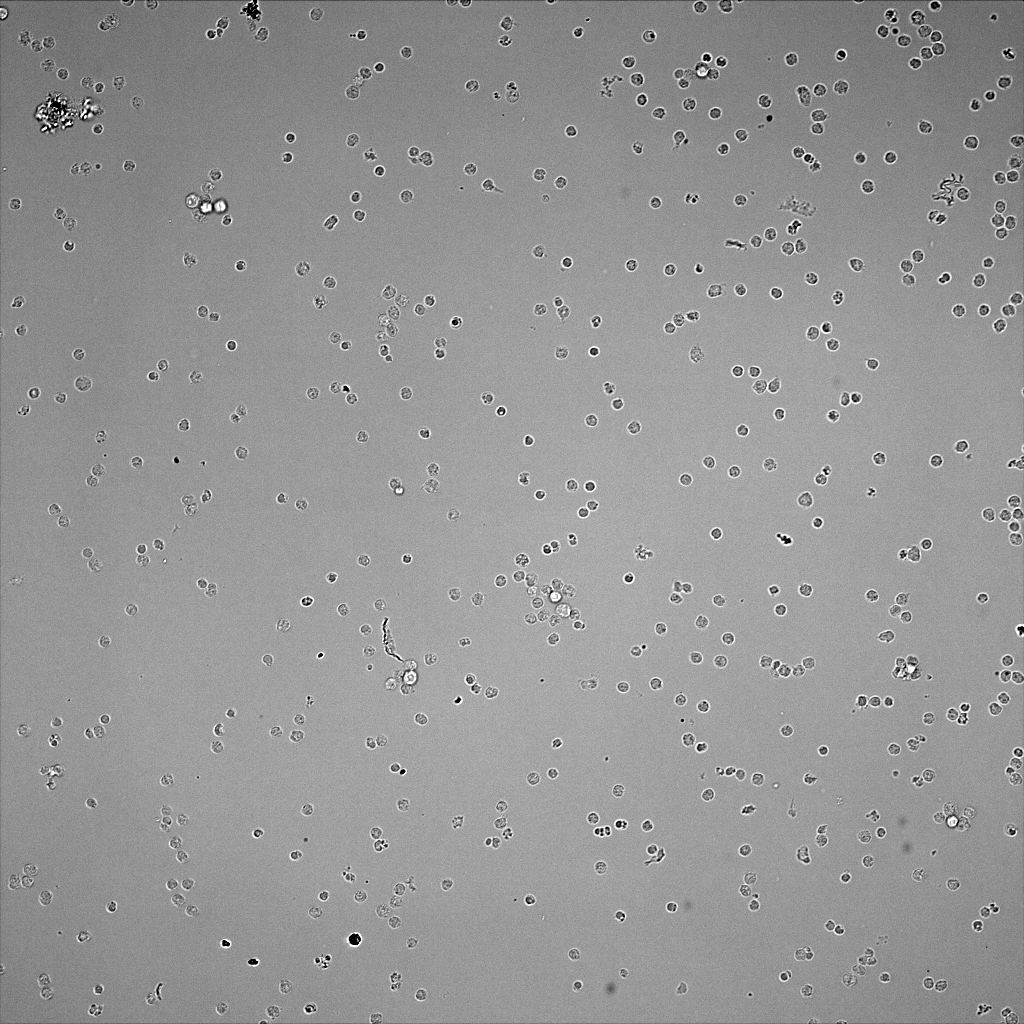

Supplement: Supplementary file 18 [file msb0011-0783-sd18.zip › Snap-173_c1_ORG.png]

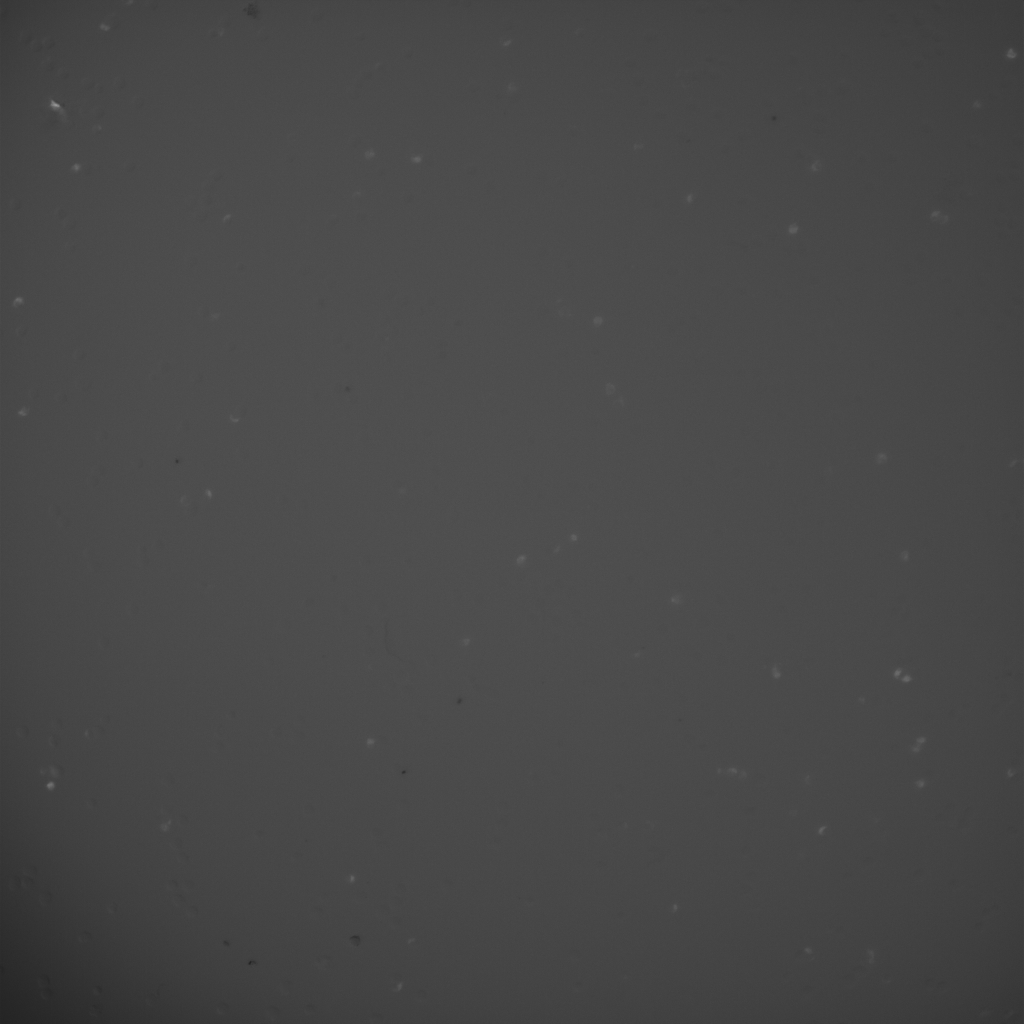

Supplement: Supplementary file 18 [file msb0011-0783-sd18.zip › Snap-173_c2_ORG.png]

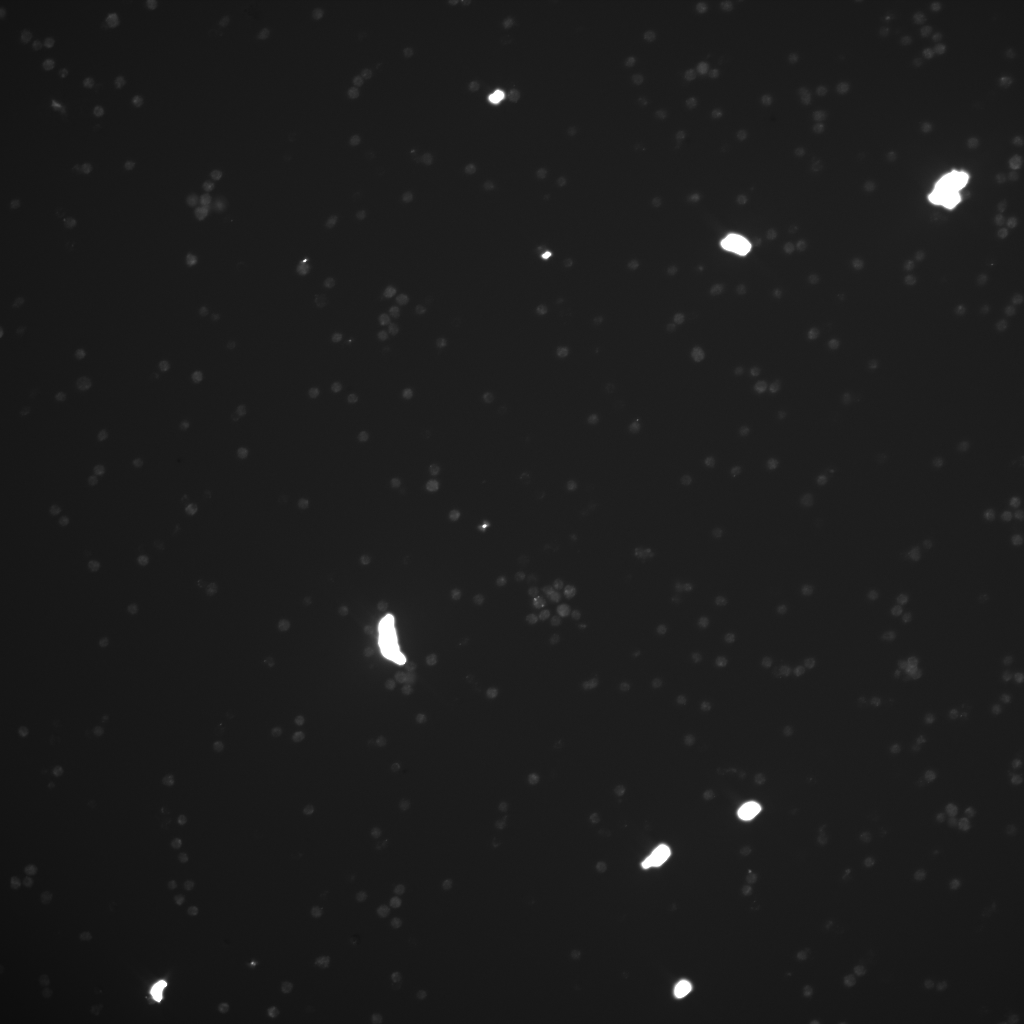

Supplement: Supplementary file 18 [file msb0011-0783-sd18.zip › Snap-173_c3_ORG.png]

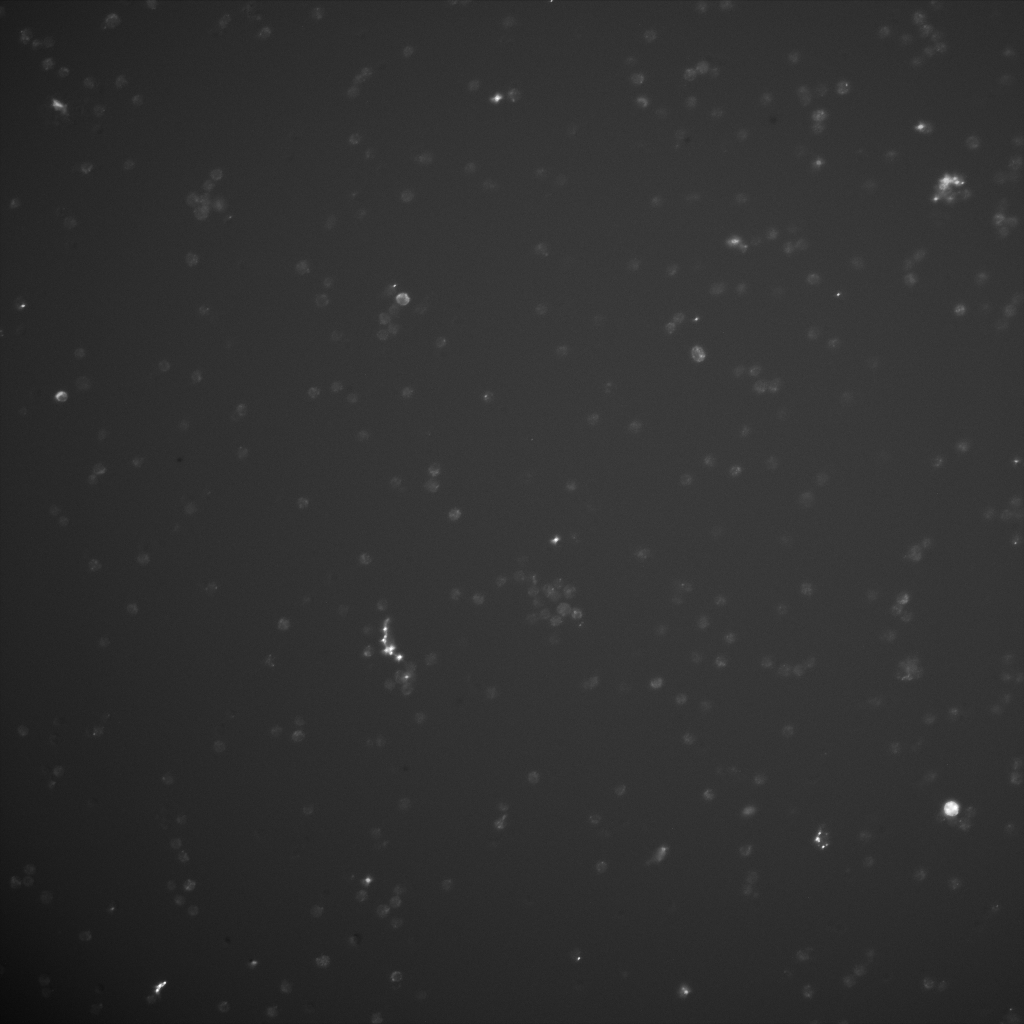

Supplement: Supplementary file 18 [file msb0011-0783-sd18.zip › Snap-173_c4_ORG.png]

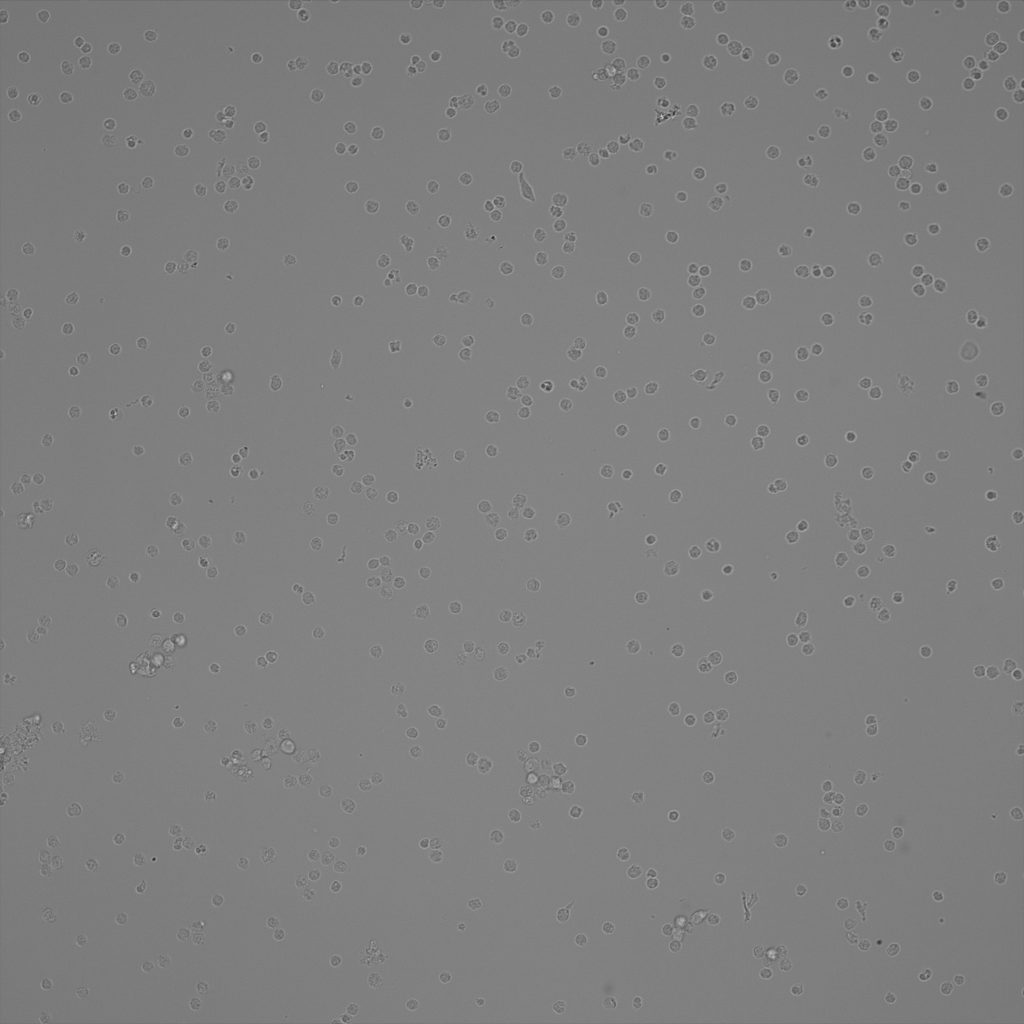

Supplement: Supplementary file 18 [file msb0011-0783-sd18.zip › Snap-174_c1_ORG.png]

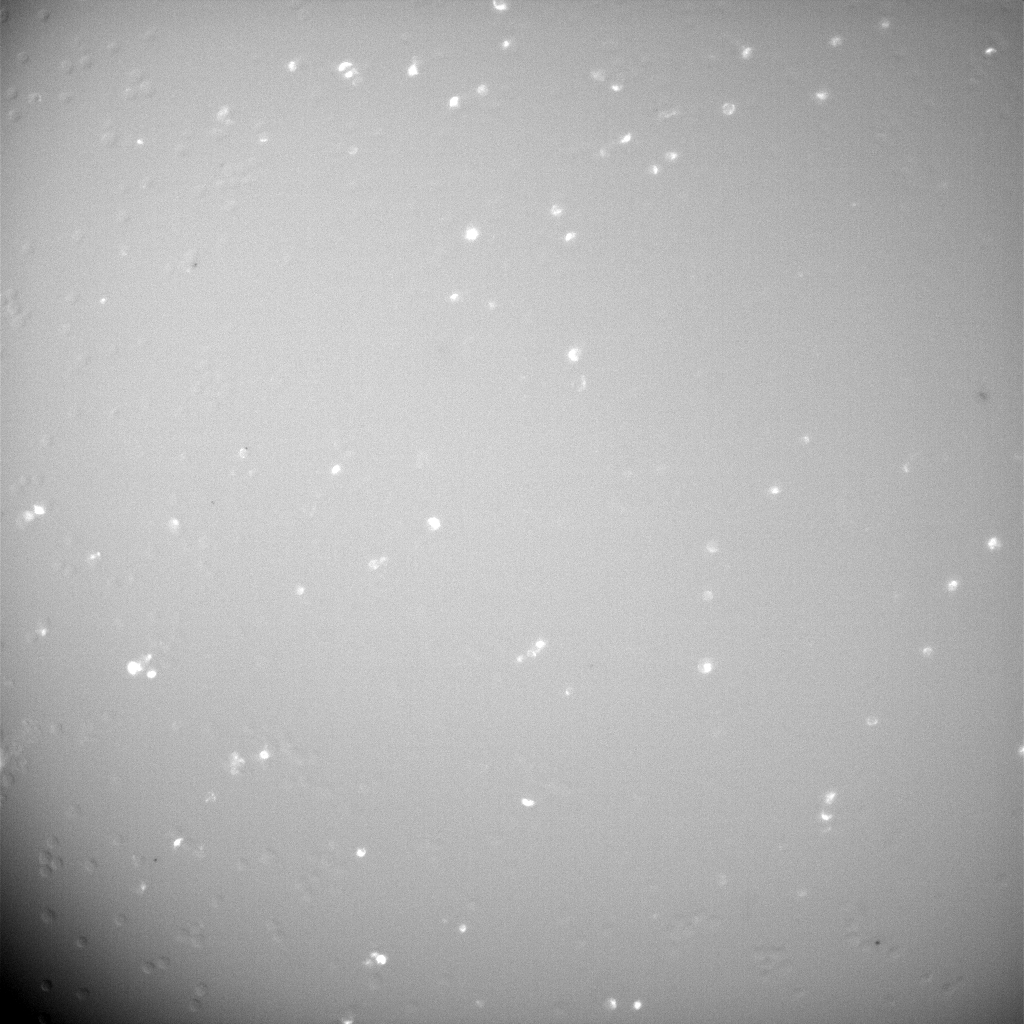

Supplement: Supplementary file 18 [file msb0011-0783-sd18.zip › Snap-174_c2_ORG.png]

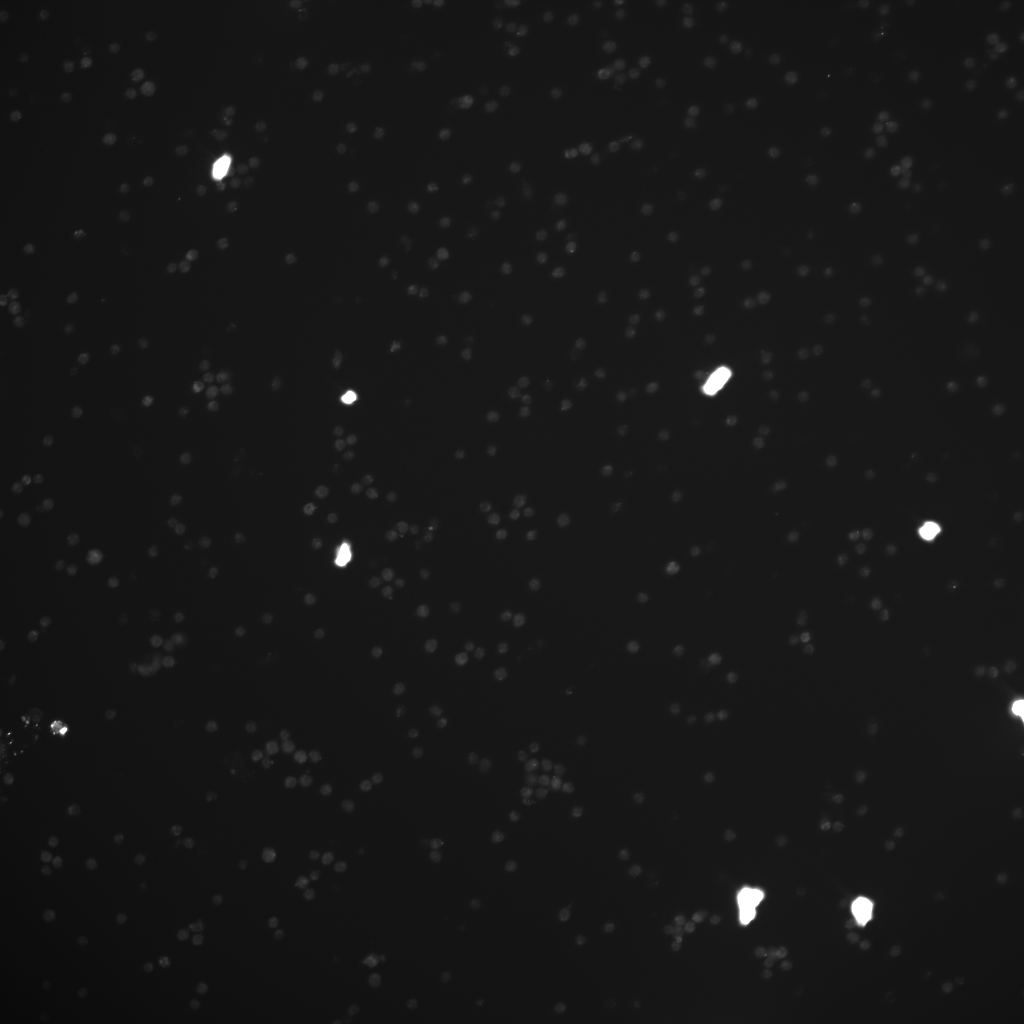

Supplement: Supplementary file 18 [file msb0011-0783-sd18.zip › Snap-174_c3_ORG.png]

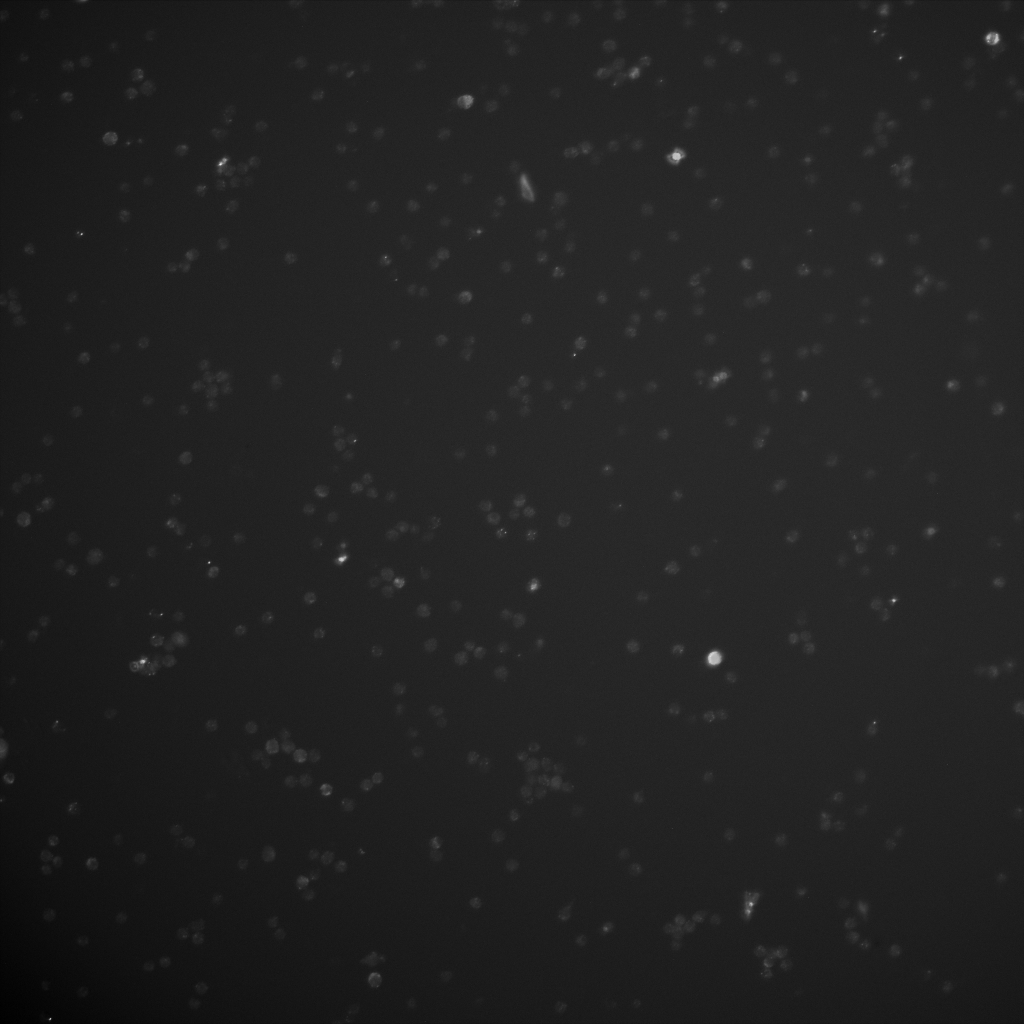

Supplement: Supplementary file 18 [file msb0011-0783-sd18.zip › Snap-174_c4_ORG.png]
