# Supplementary figures and images for: A multi-scale approach reveals that NF-κB cRel enforces a B-cell decision to divide (part 2 of 3)
Source: Mol Syst Biol. 2015 Feb 13;11(2):783. doi: 10.15252/msb.20145554 (PMC4358656; doi:10.15252/msb.20145554)

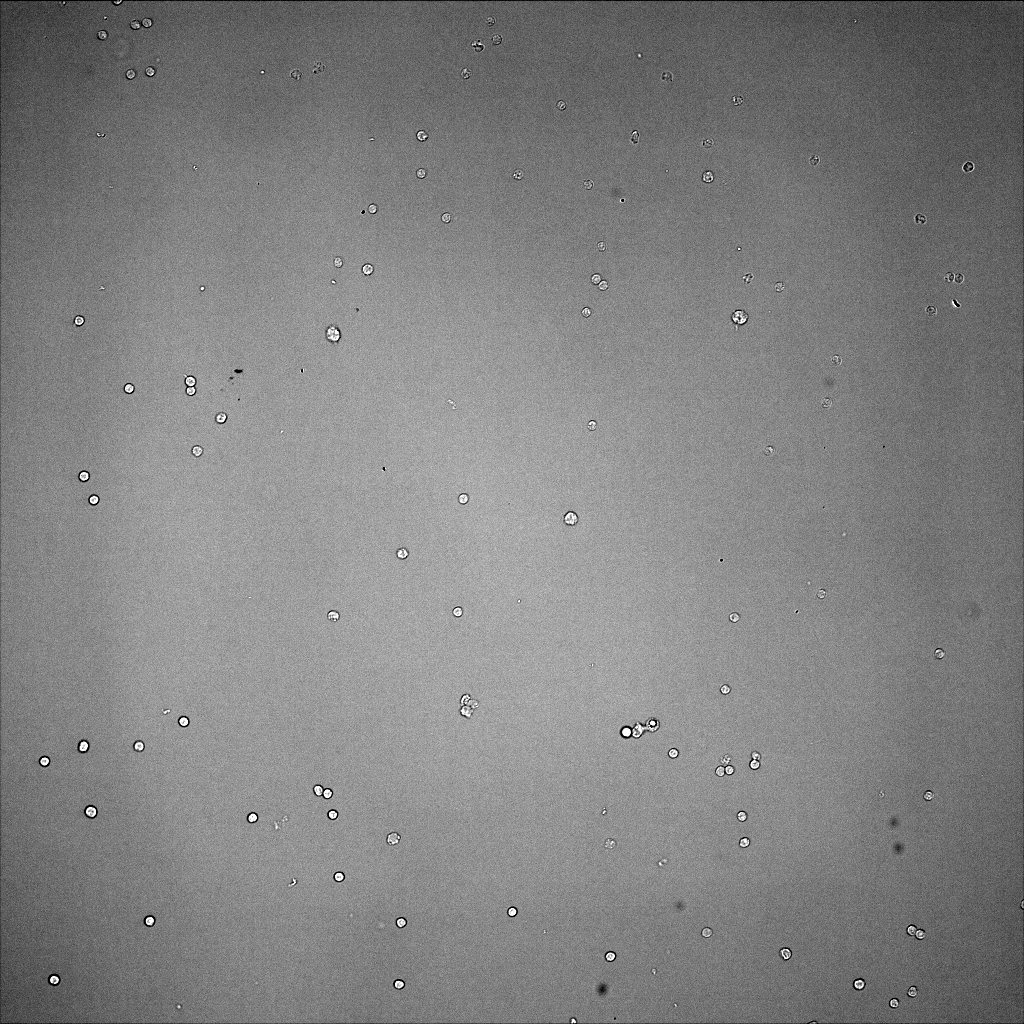

Supplement: Supplementary file 18 [file msb0011-0783-sd18.zip › Snap-43_c1_ORG.png]

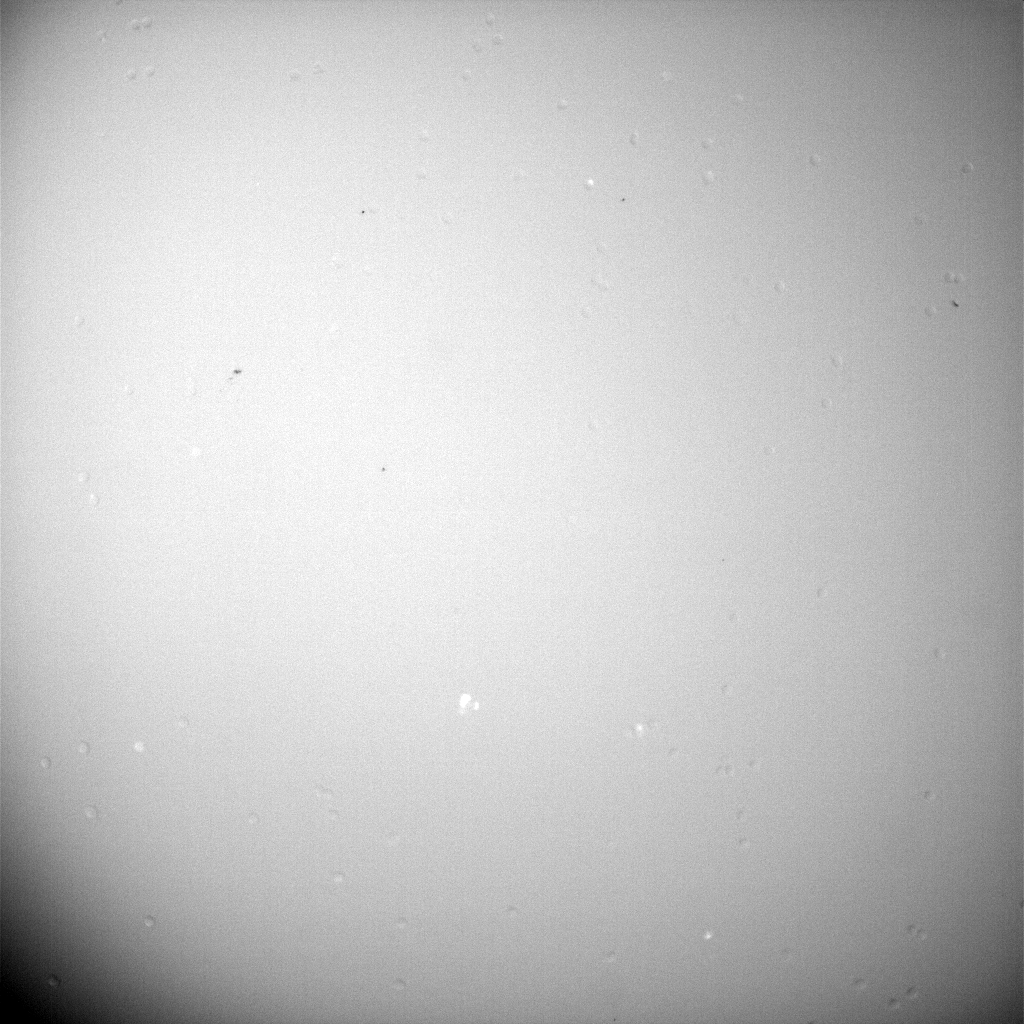

Supplement: Supplementary file 18 [file msb0011-0783-sd18.zip › Snap-43_c2_ORG.png]

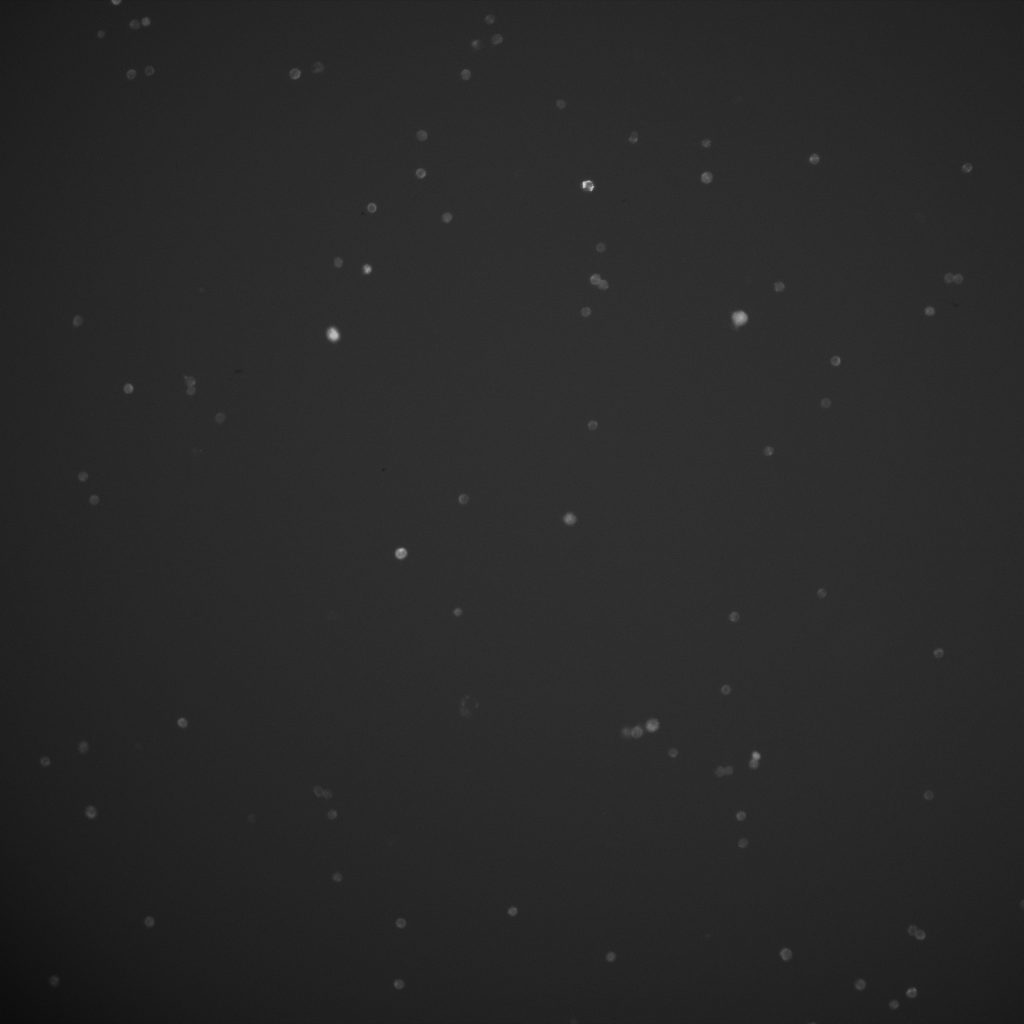

Supplement: Supplementary file 18 [file msb0011-0783-sd18.zip › Snap-43_c3_ORG.png]

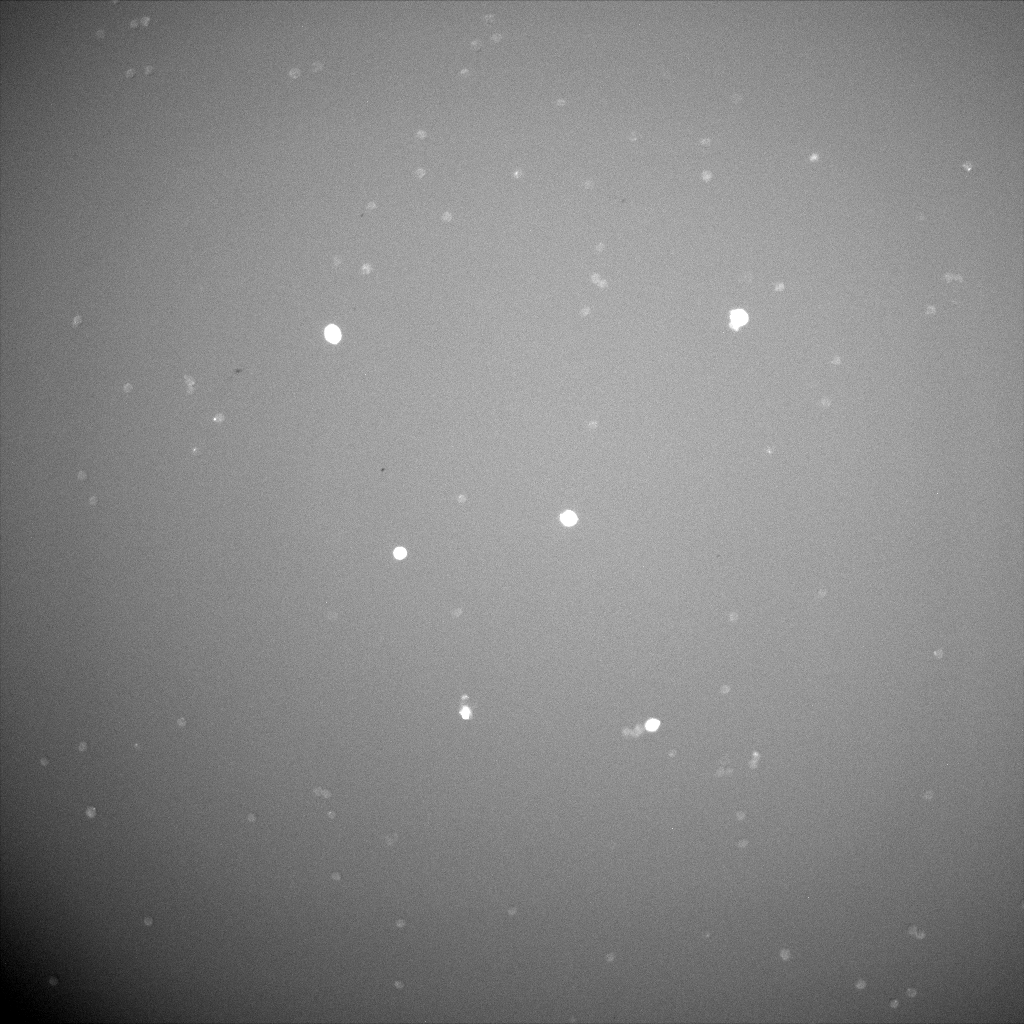

Supplement: Supplementary file 18 [file msb0011-0783-sd18.zip › Snap-43_c4_ORG.png]

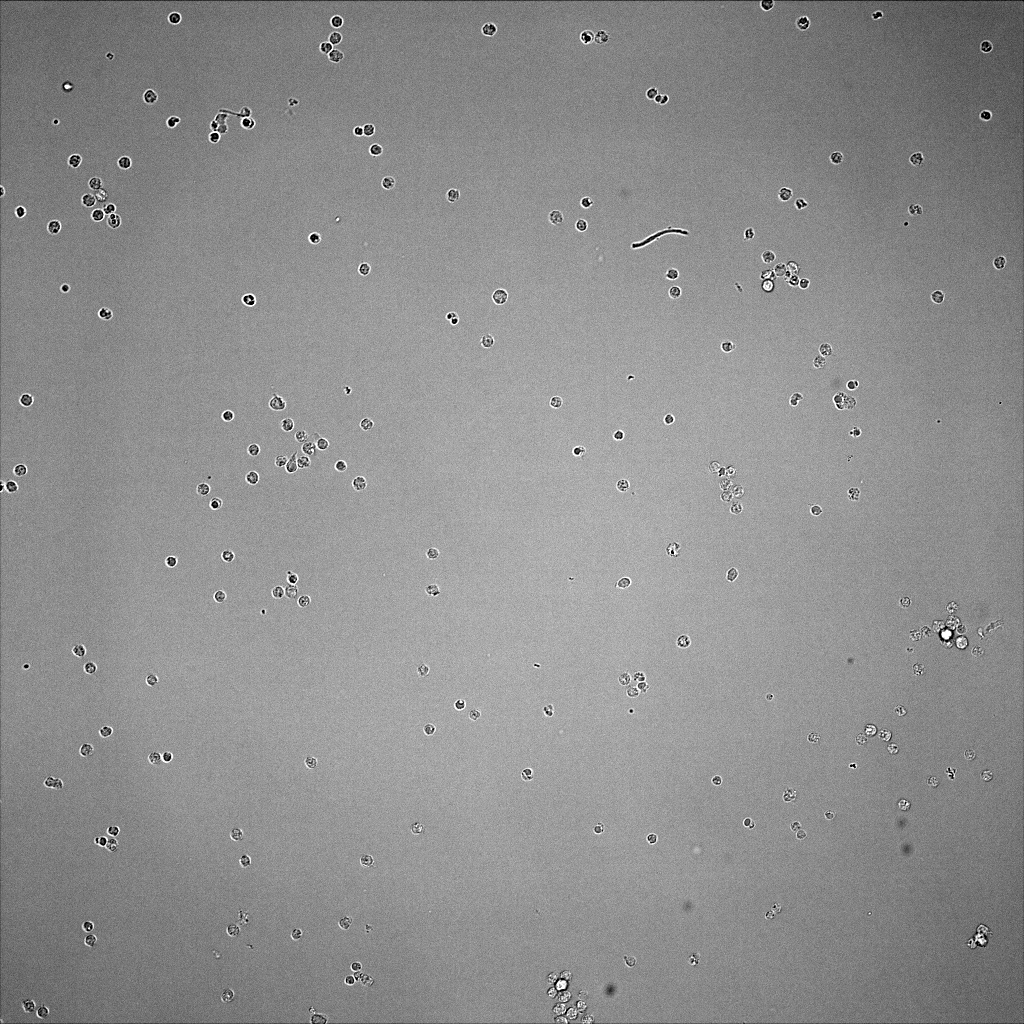

Supplement: Supplementary file 18 [file msb0011-0783-sd18.zip › Snap-44_c1_ORG.png]

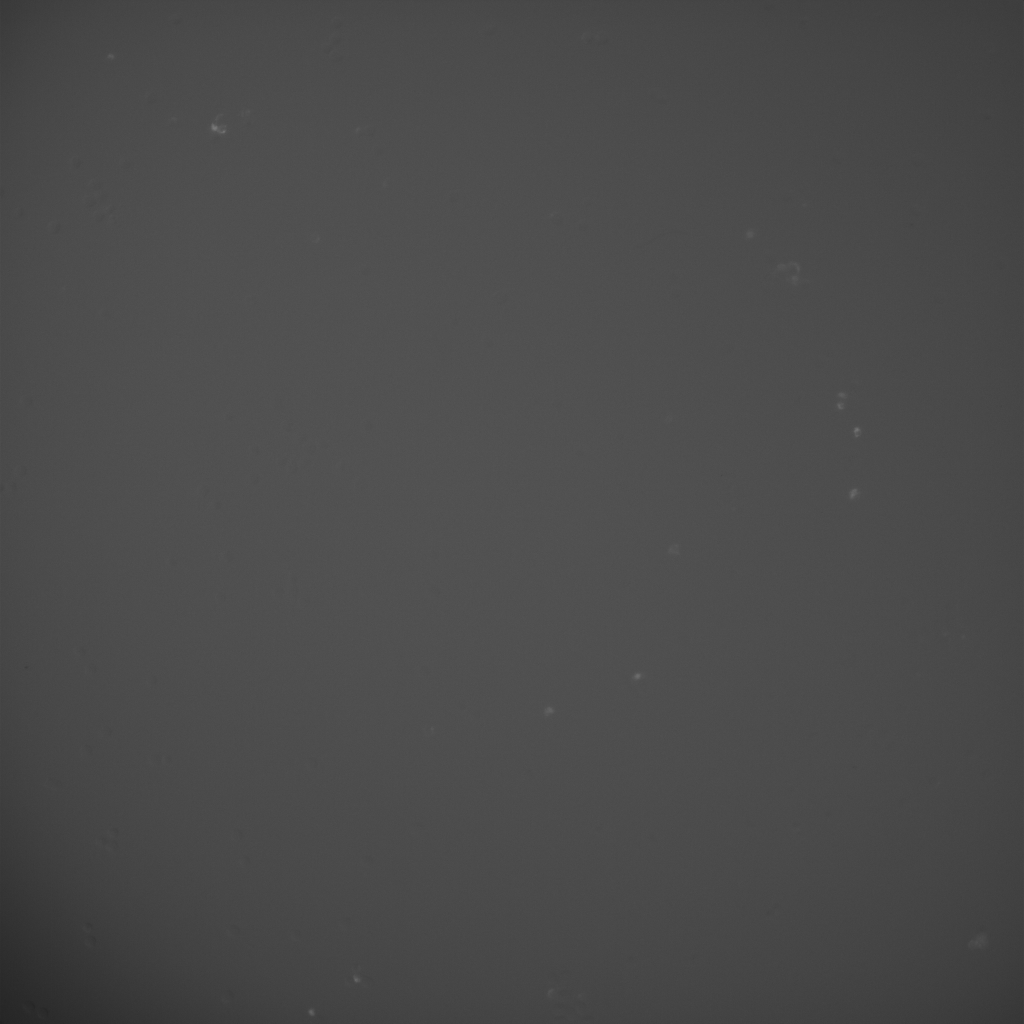

Supplement: Supplementary file 18 [file msb0011-0783-sd18.zip › Snap-44_c2_ORG.png]

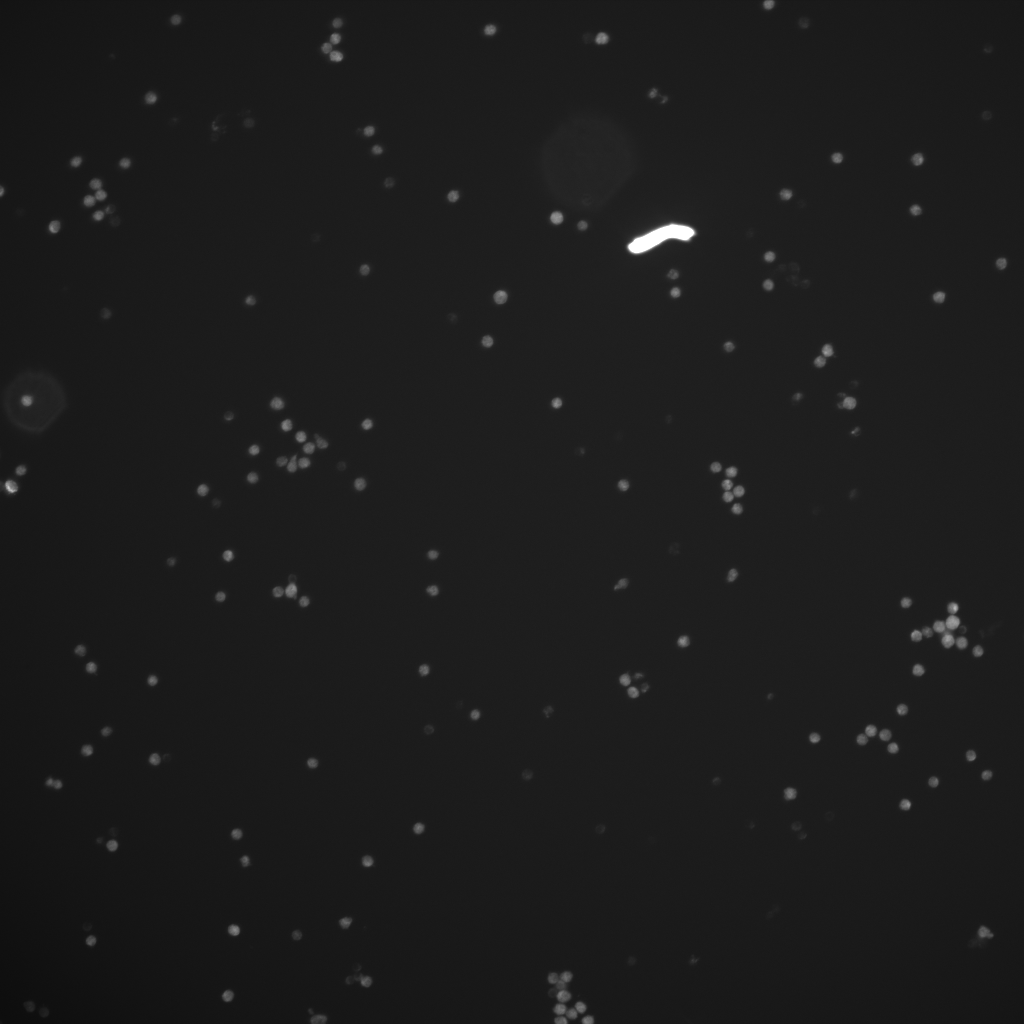

Supplement: Supplementary file 18 [file msb0011-0783-sd18.zip › Snap-44_c3_ORG.png]

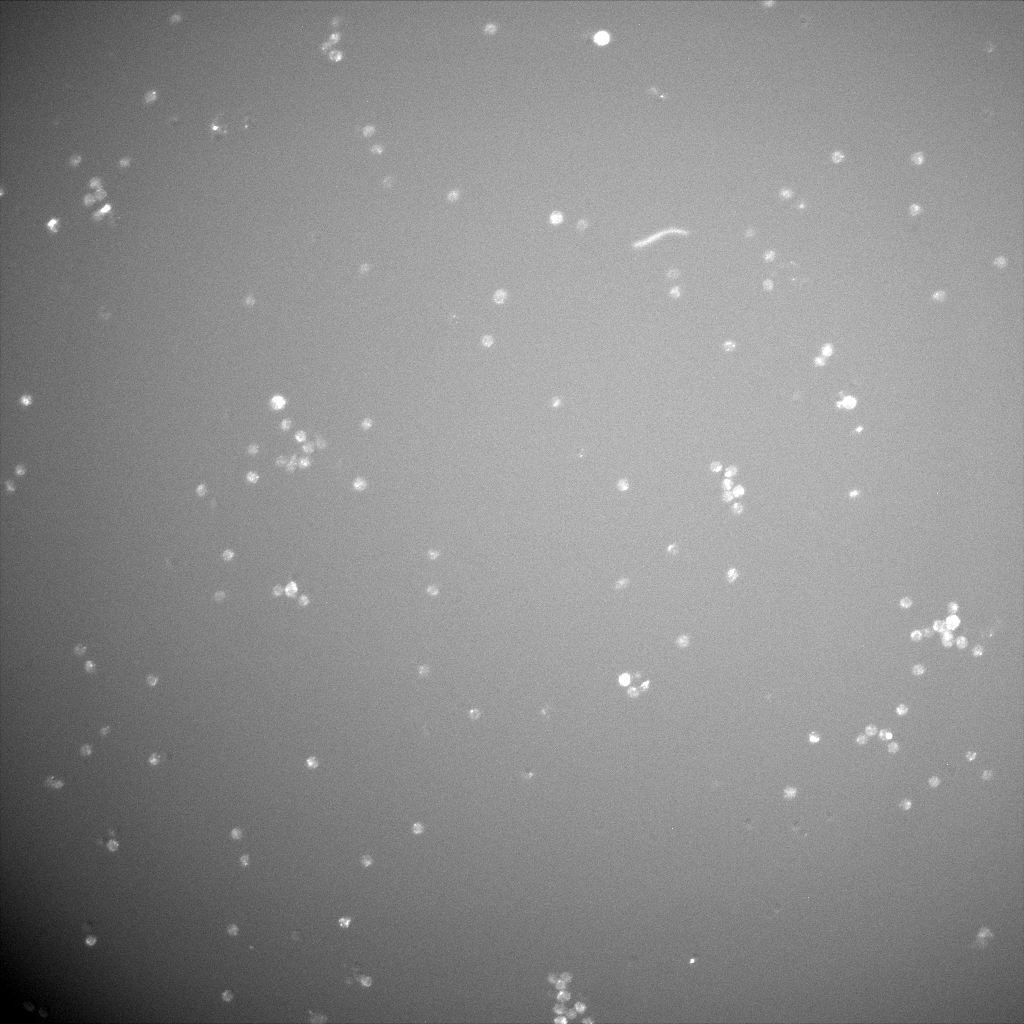

Supplement: Supplementary file 18 [file msb0011-0783-sd18.zip › Snap-44_c4_ORG.png]

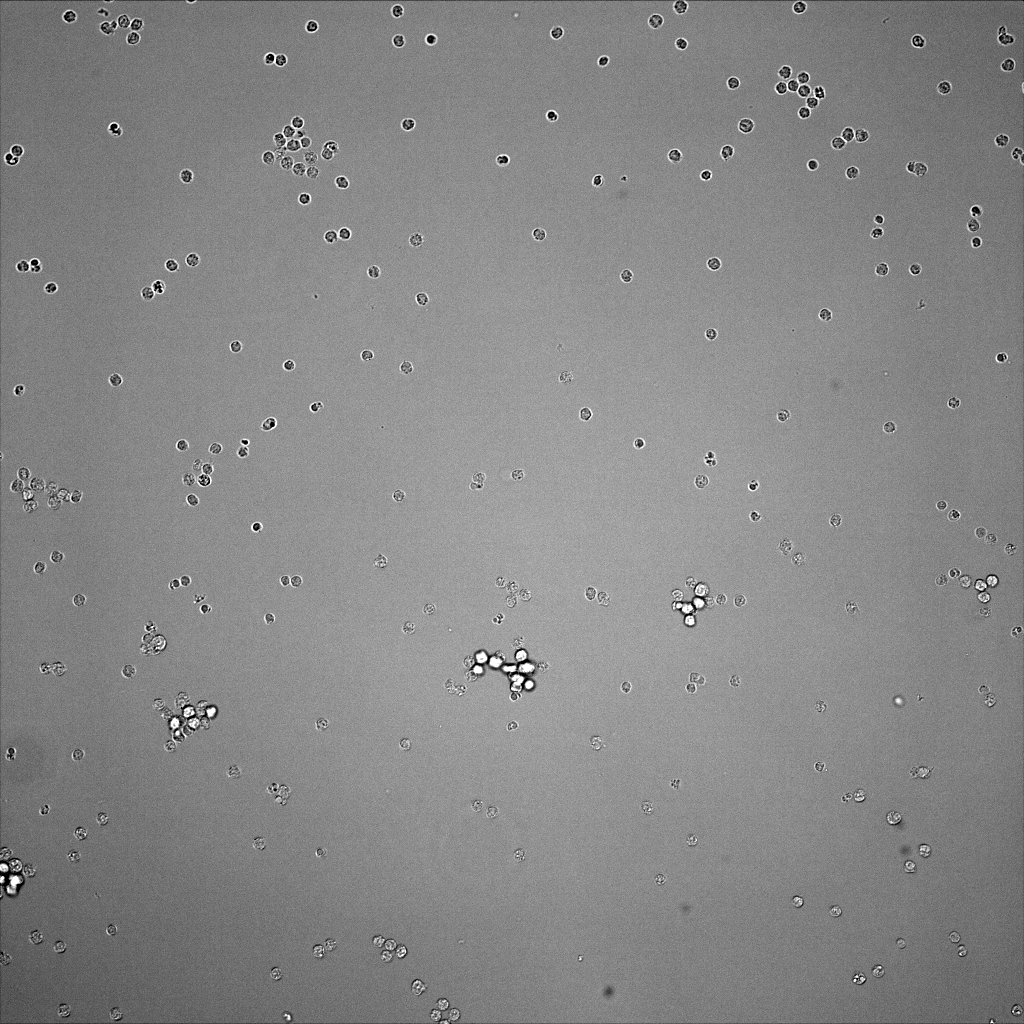

Supplement: Supplementary file 18 [file msb0011-0783-sd18.zip › Snap-45_c1_ORG.png]

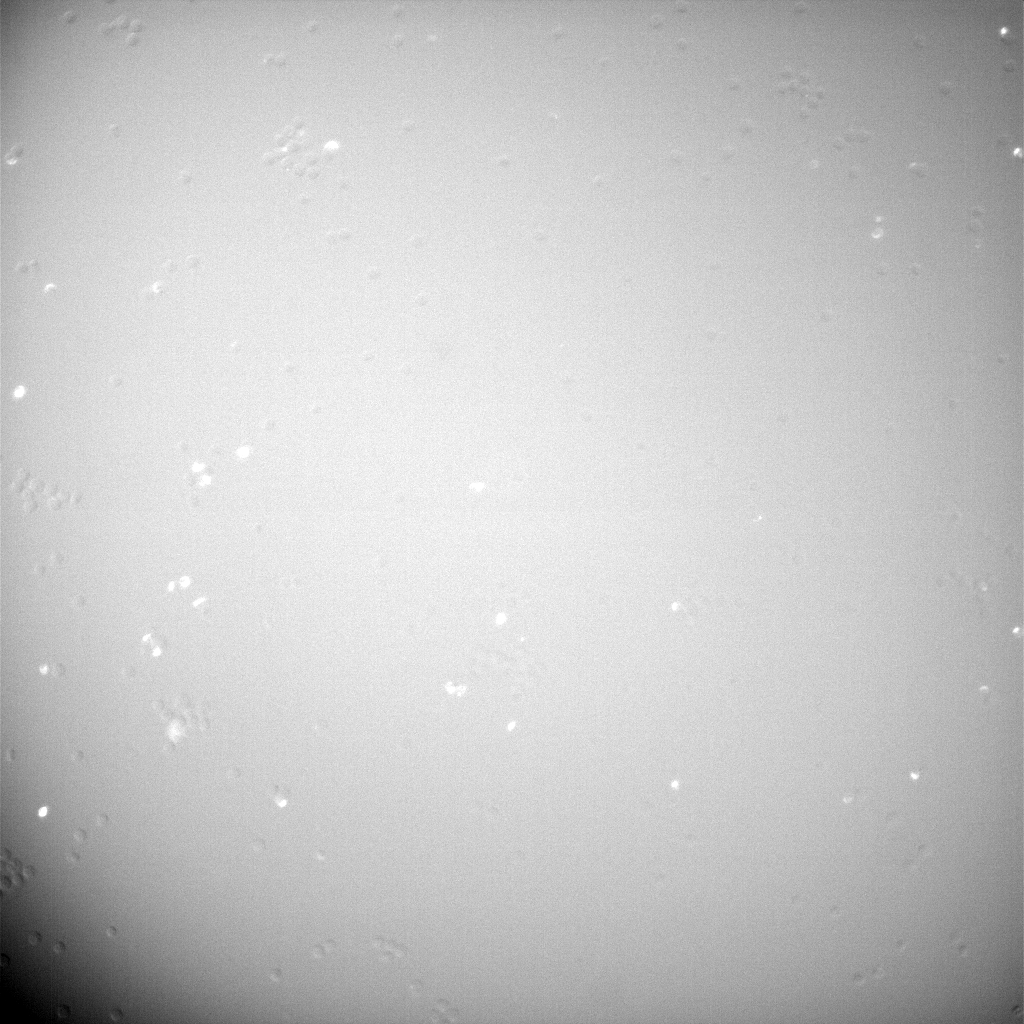

Supplement: Supplementary file 18 [file msb0011-0783-sd18.zip › Snap-45_c2_ORG.png]

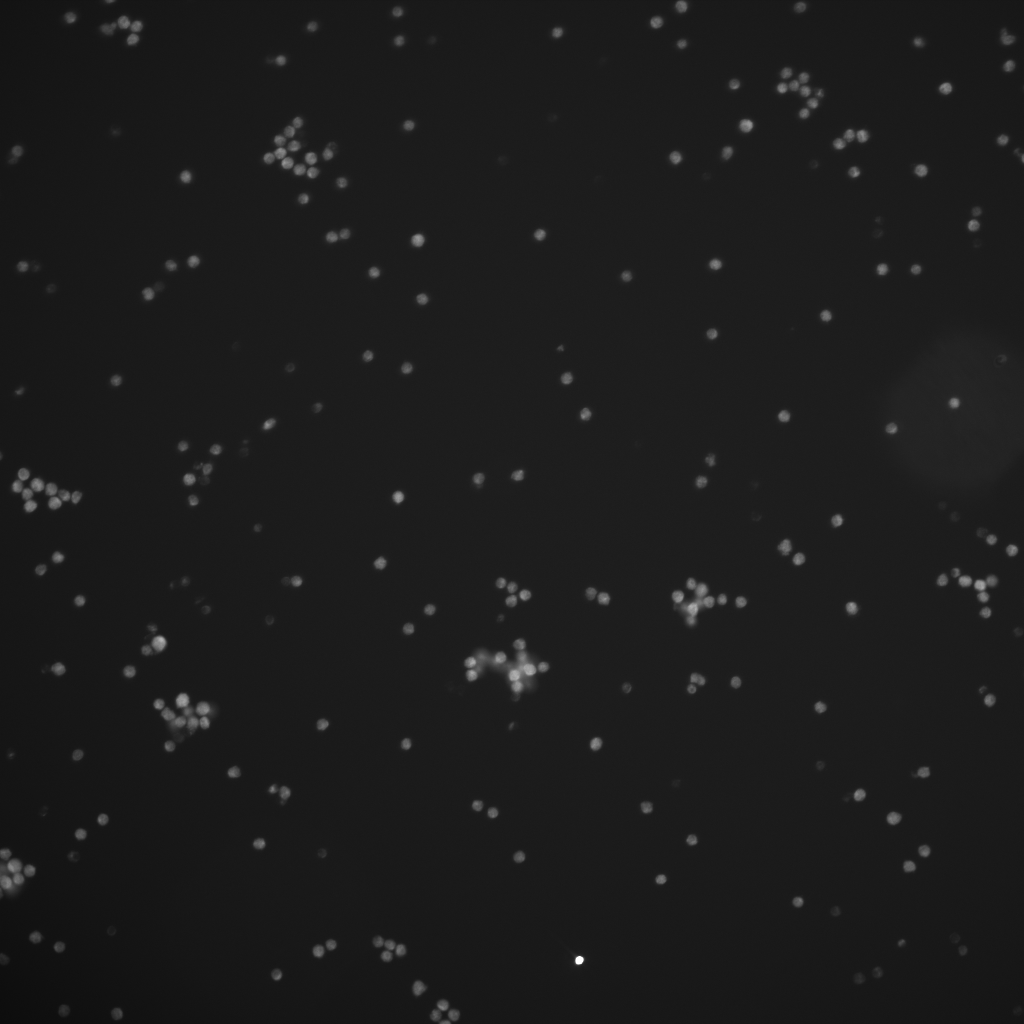

Supplement: Supplementary file 18 [file msb0011-0783-sd18.zip › Snap-45_c3_ORG.png]

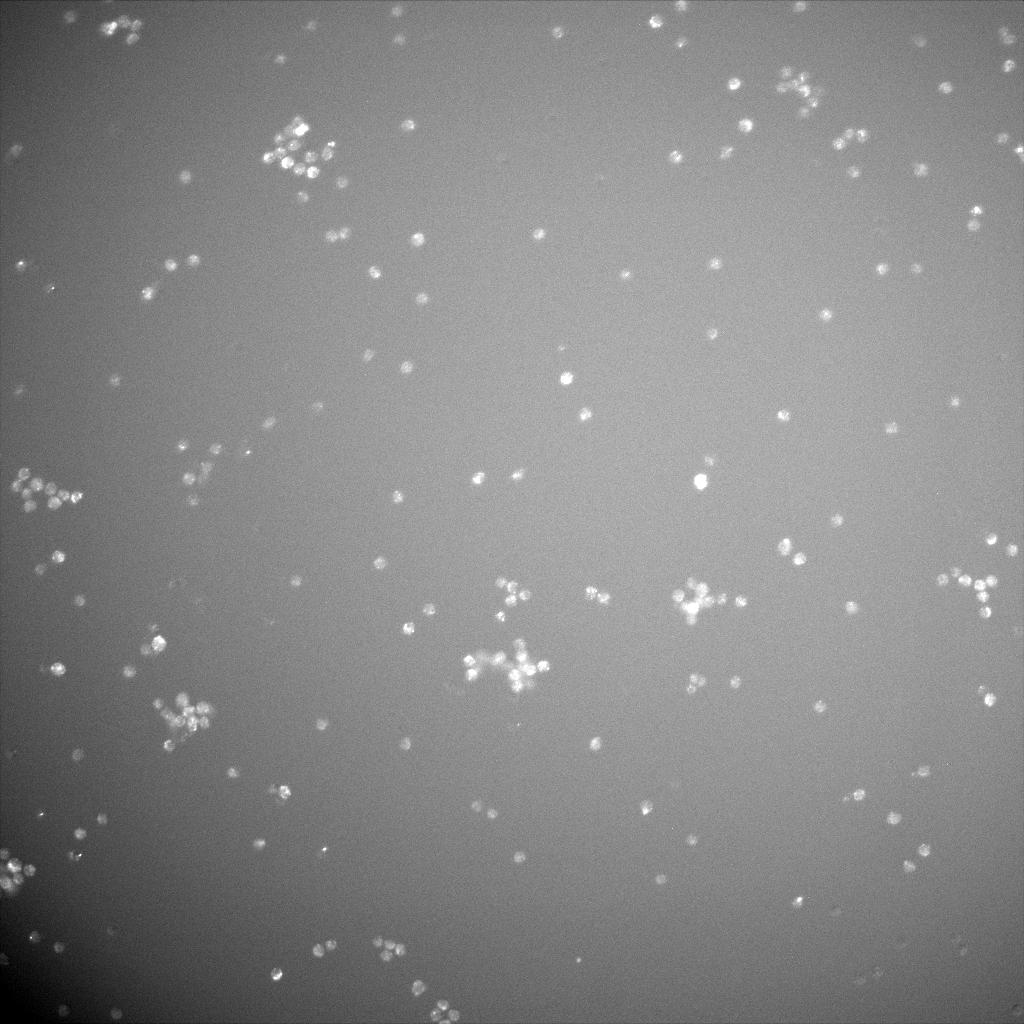

Supplement: Supplementary file 18 [file msb0011-0783-sd18.zip › Snap-45_c4_ORG.png]

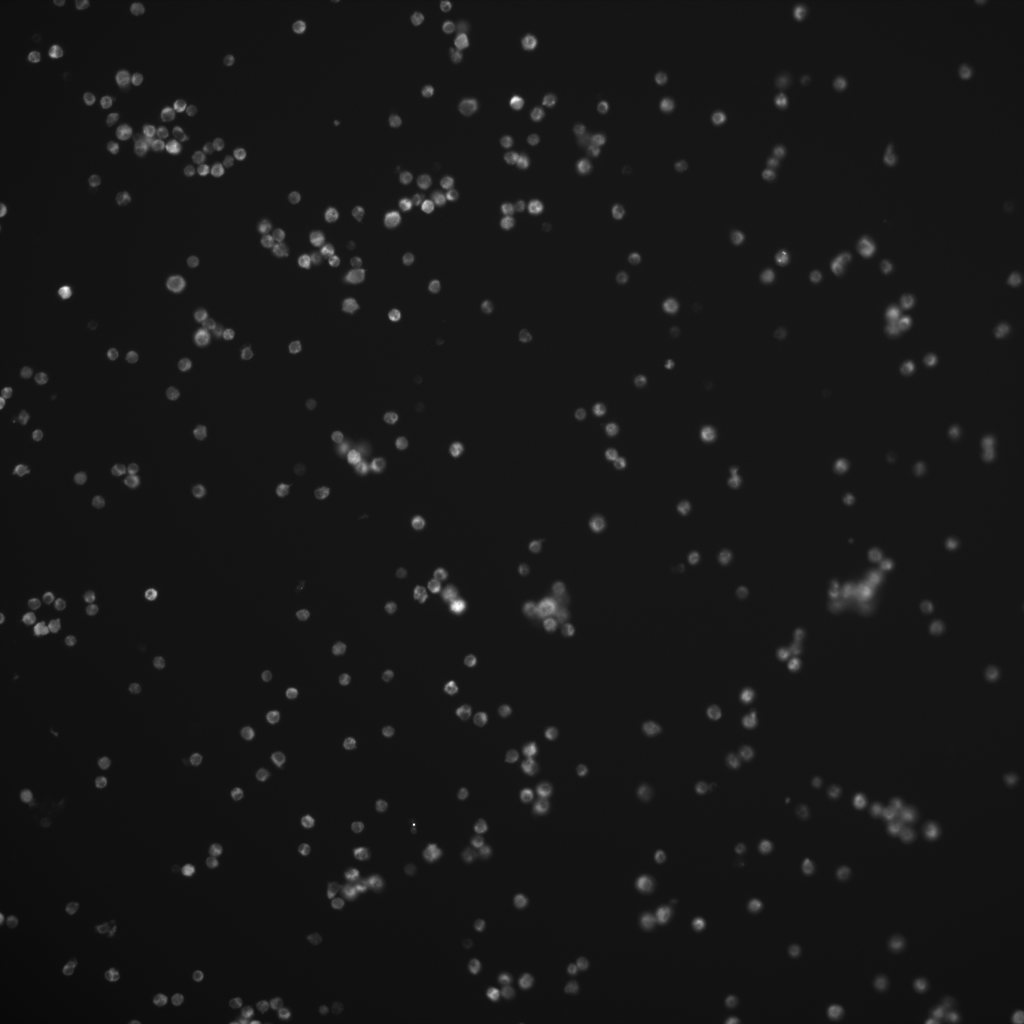

Supplement: Supplementary file 18 [file msb0011-0783-sd18.zip › Snap-155_c4_ORG.png]

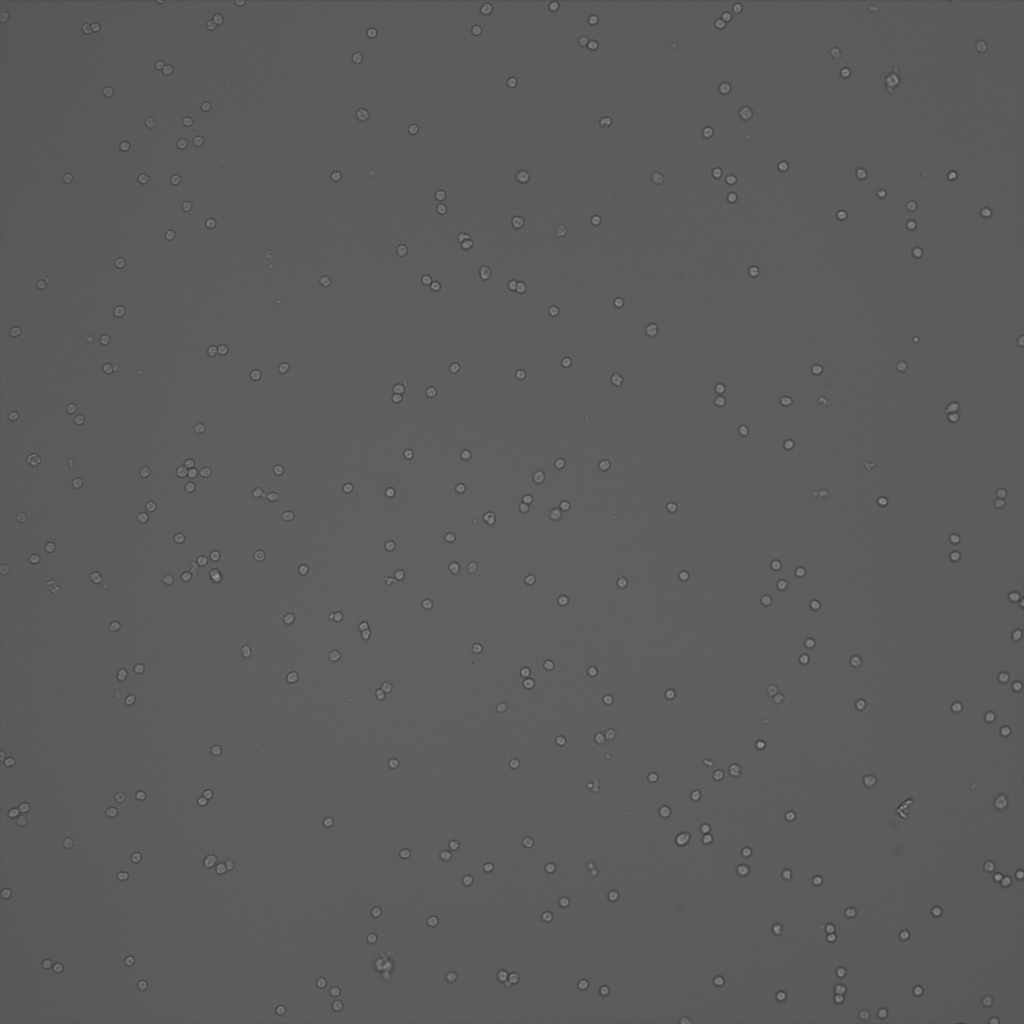

Supplement: Supplementary file 18 [file msb0011-0783-sd18.zip › Snap-142_c1_ORG.png]

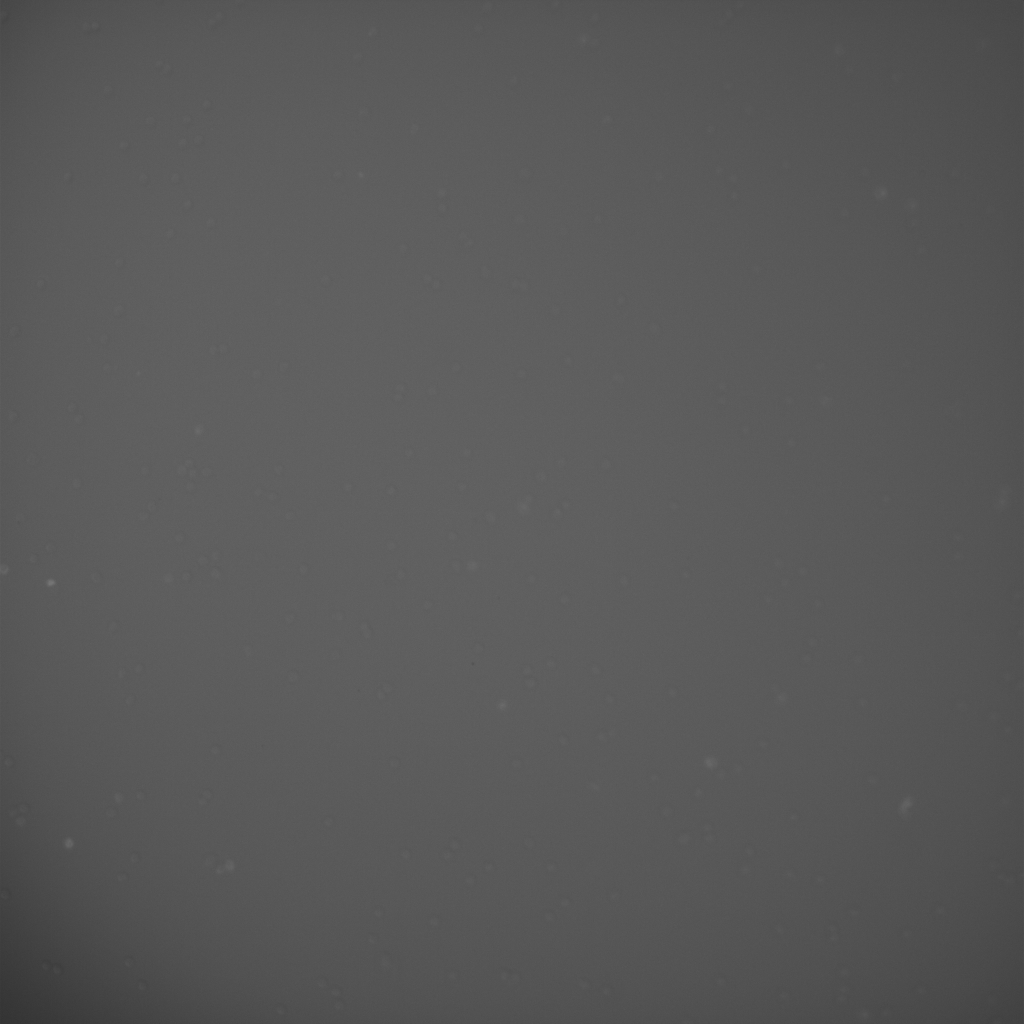

Supplement: Supplementary file 18 [file msb0011-0783-sd18.zip › Snap-142_c2_ORG.png]

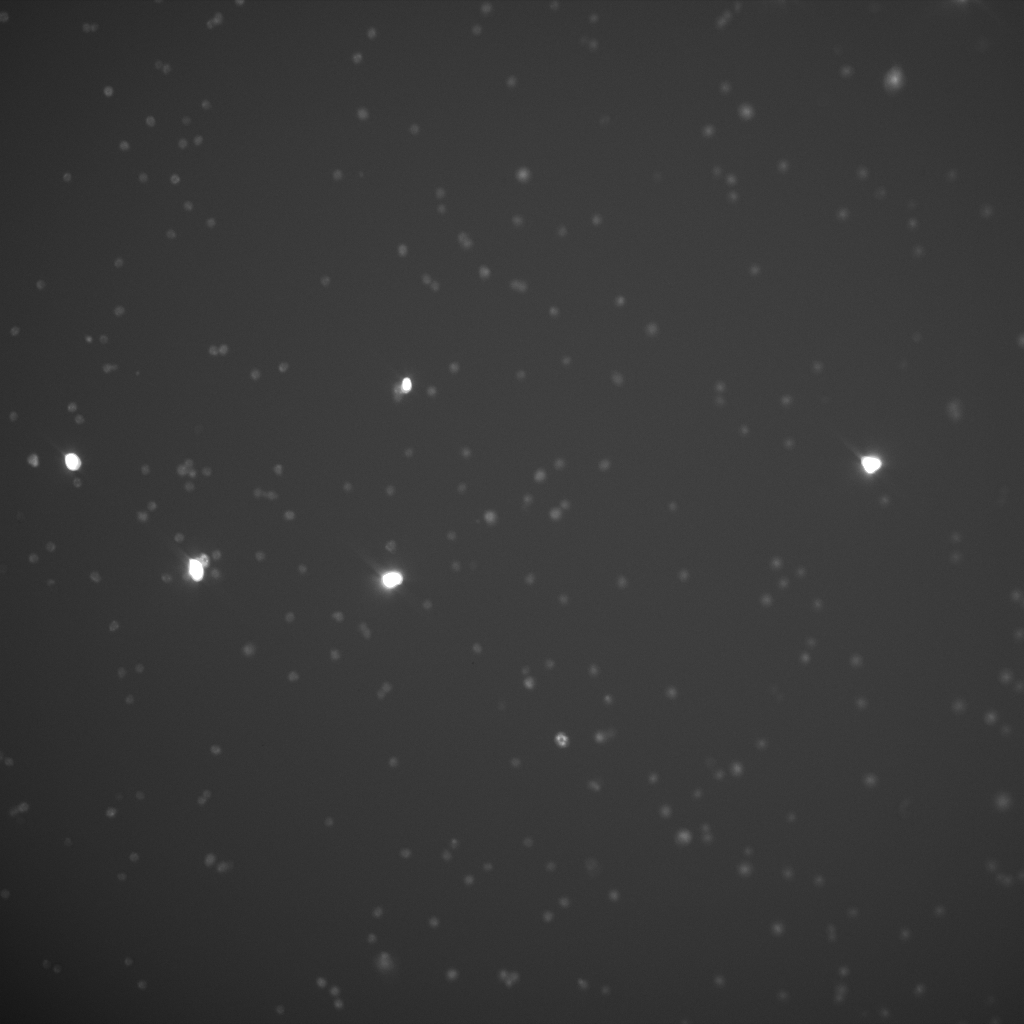

Supplement: Supplementary file 18 [file msb0011-0783-sd18.zip › Snap-142_c3_ORG.png]

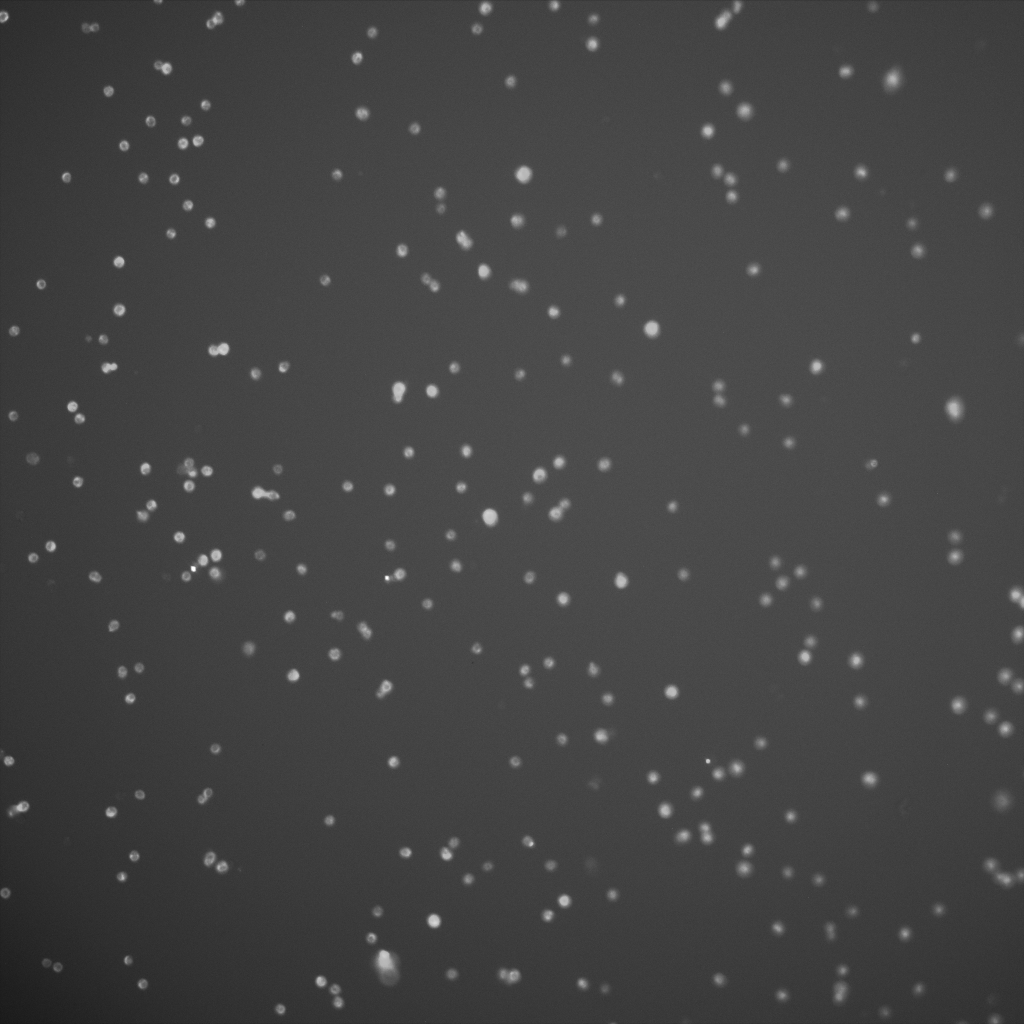

Supplement: Supplementary file 18 [file msb0011-0783-sd18.zip › Snap-142_c4_ORG.png]

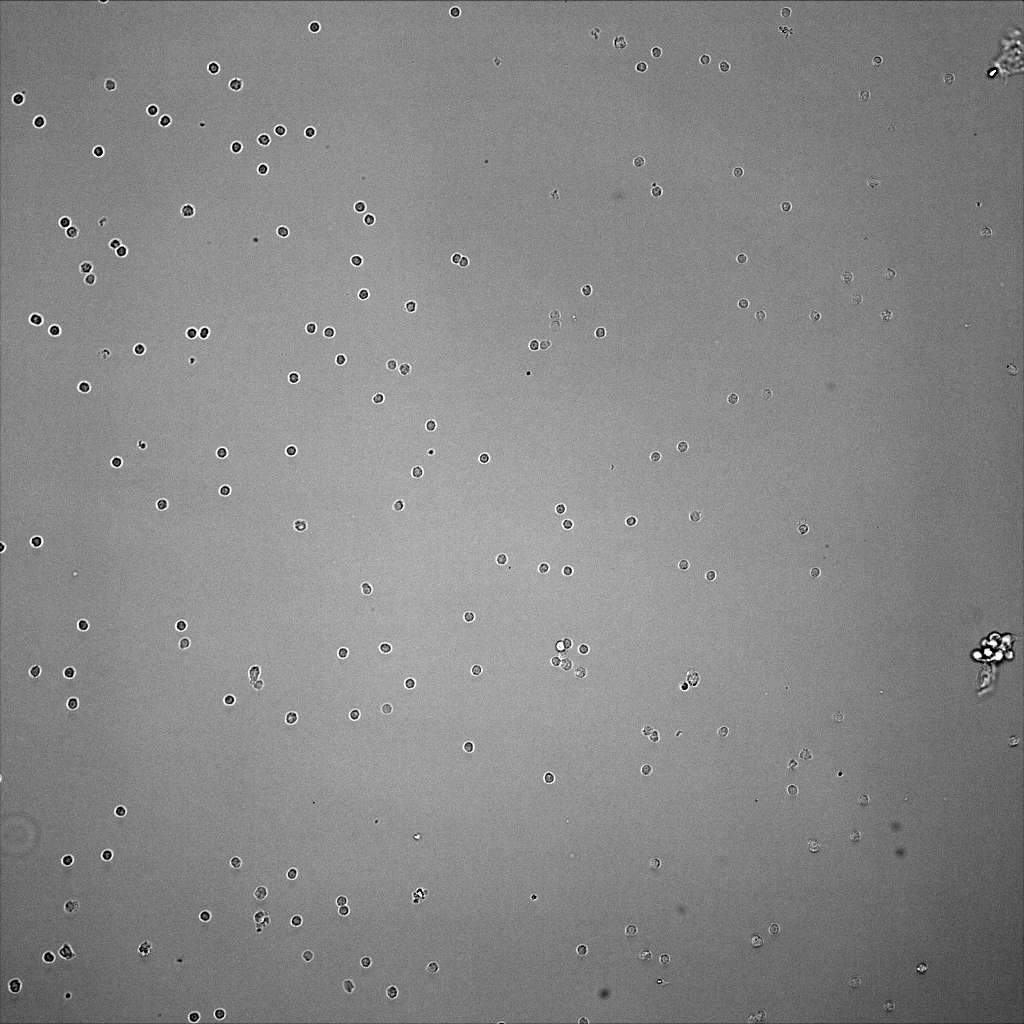

Supplement: Supplementary file 18 [file msb0011-0783-sd18.zip › Snap-143_c1_ORG.png]

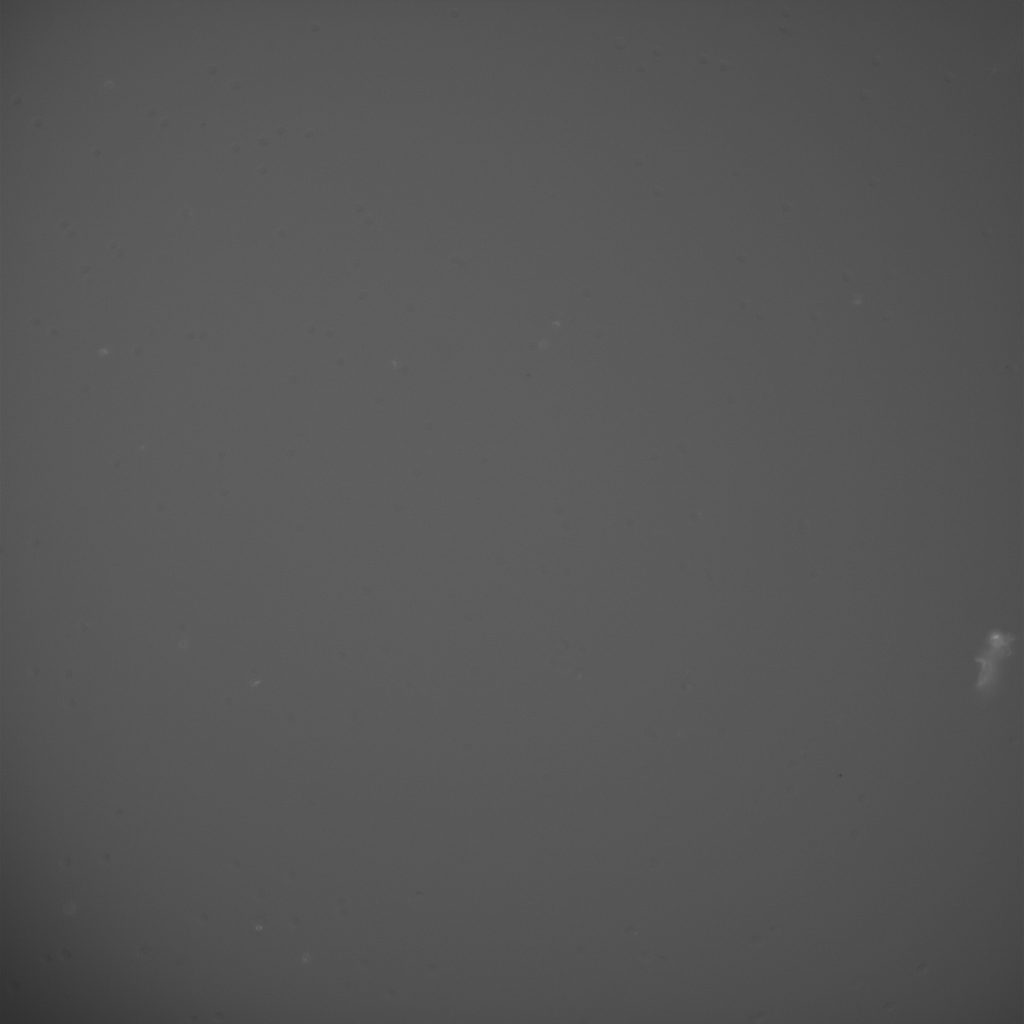

Supplement: Supplementary file 18 [file msb0011-0783-sd18.zip › Snap-143_c2_ORG.png]

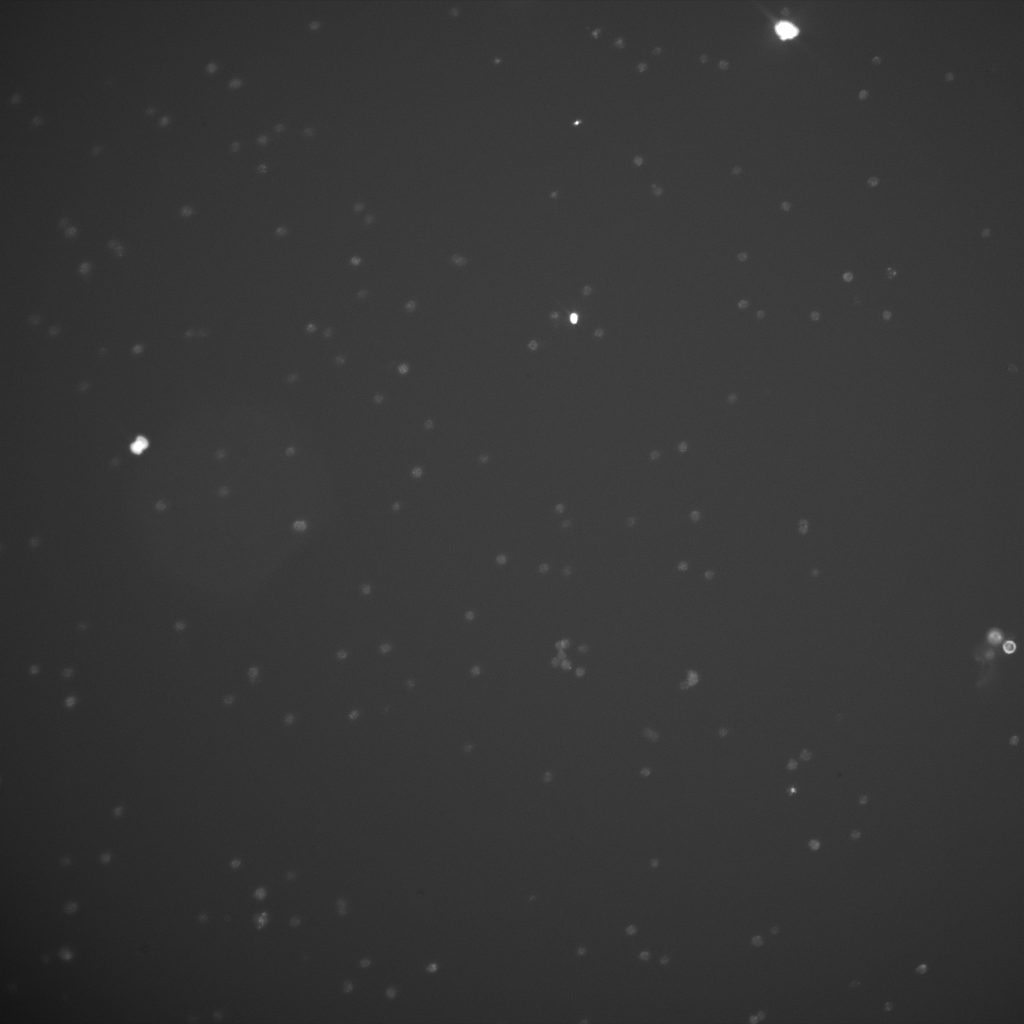

Supplement: Supplementary file 18 [file msb0011-0783-sd18.zip › Snap-143_c3_ORG.png]

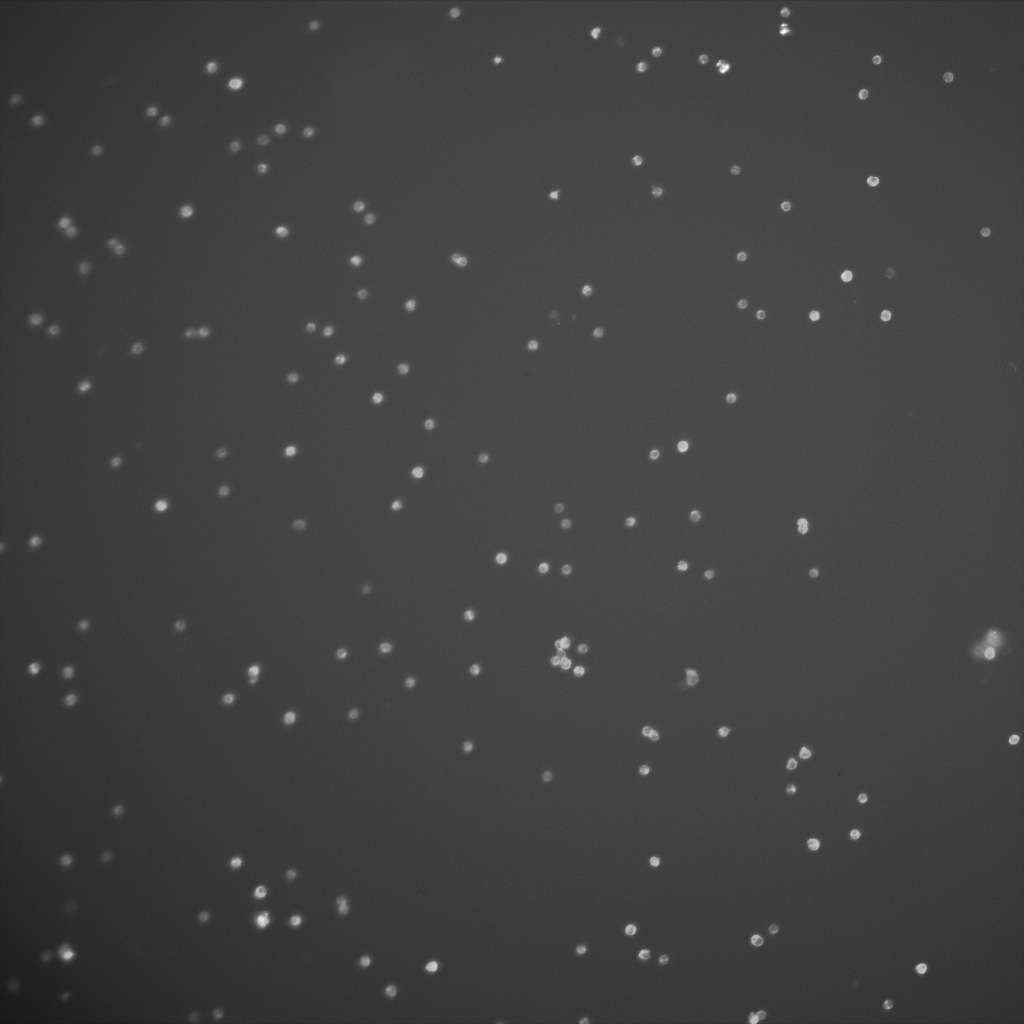

Supplement: Supplementary file 18 [file msb0011-0783-sd18.zip › Snap-143_c4_ORG.png]

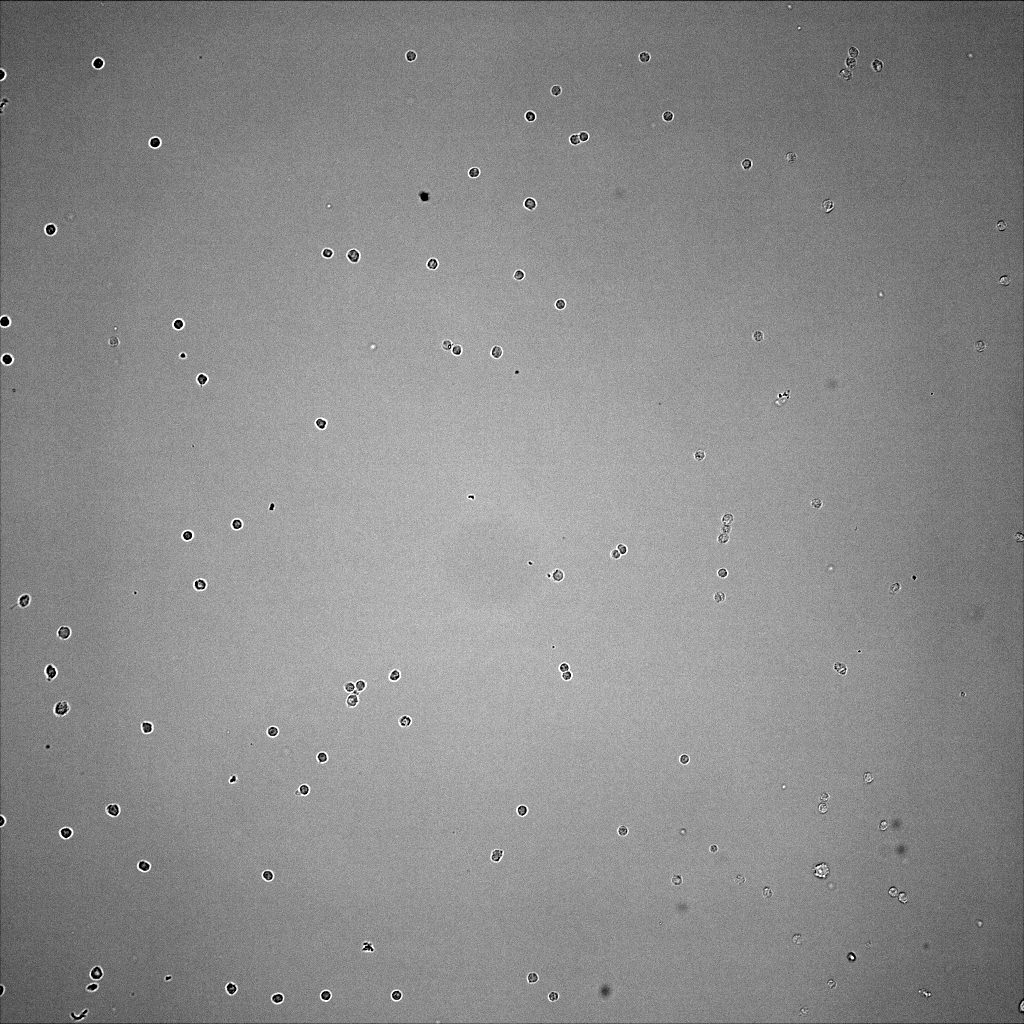

Supplement: Supplementary file 18 [file msb0011-0783-sd18.zip › Snap-144_c1_ORG.png]

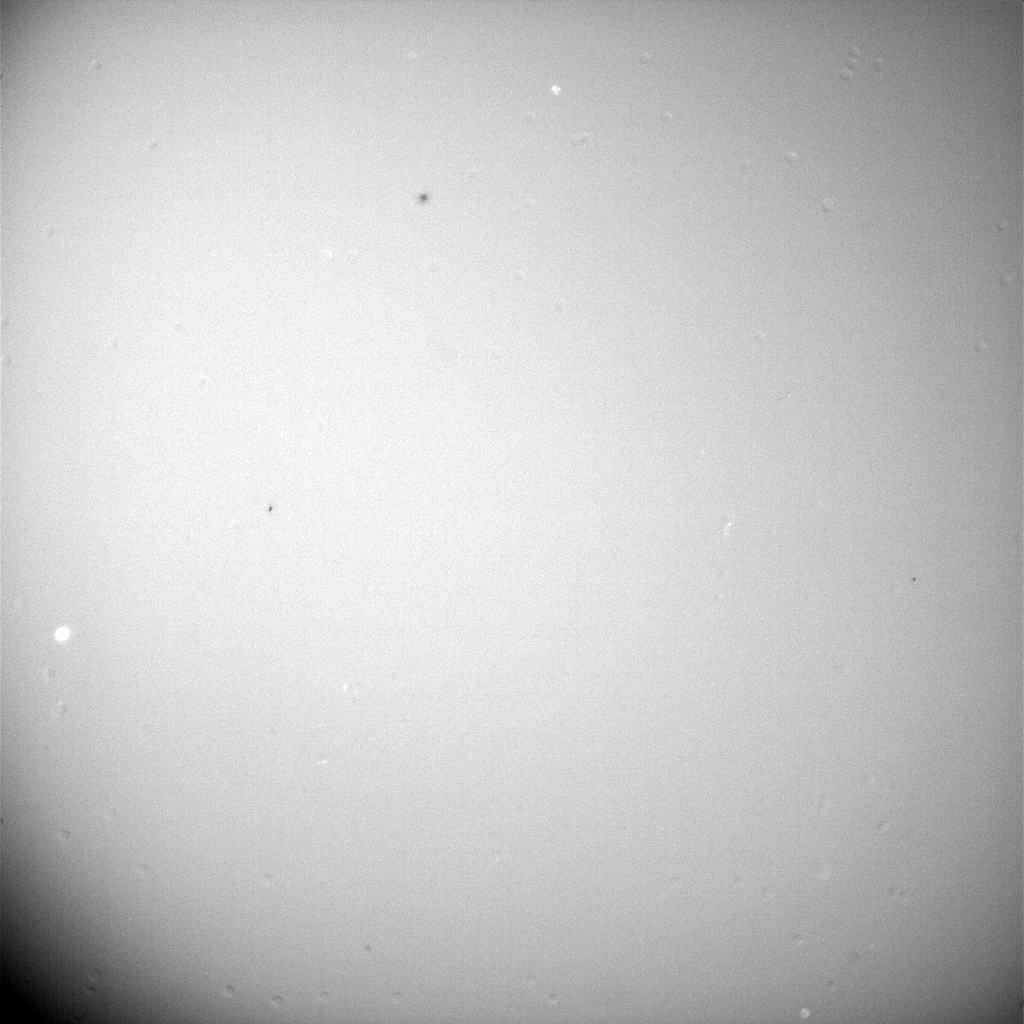

Supplement: Supplementary file 18 [file msb0011-0783-sd18.zip › Snap-144_c2_ORG.png]

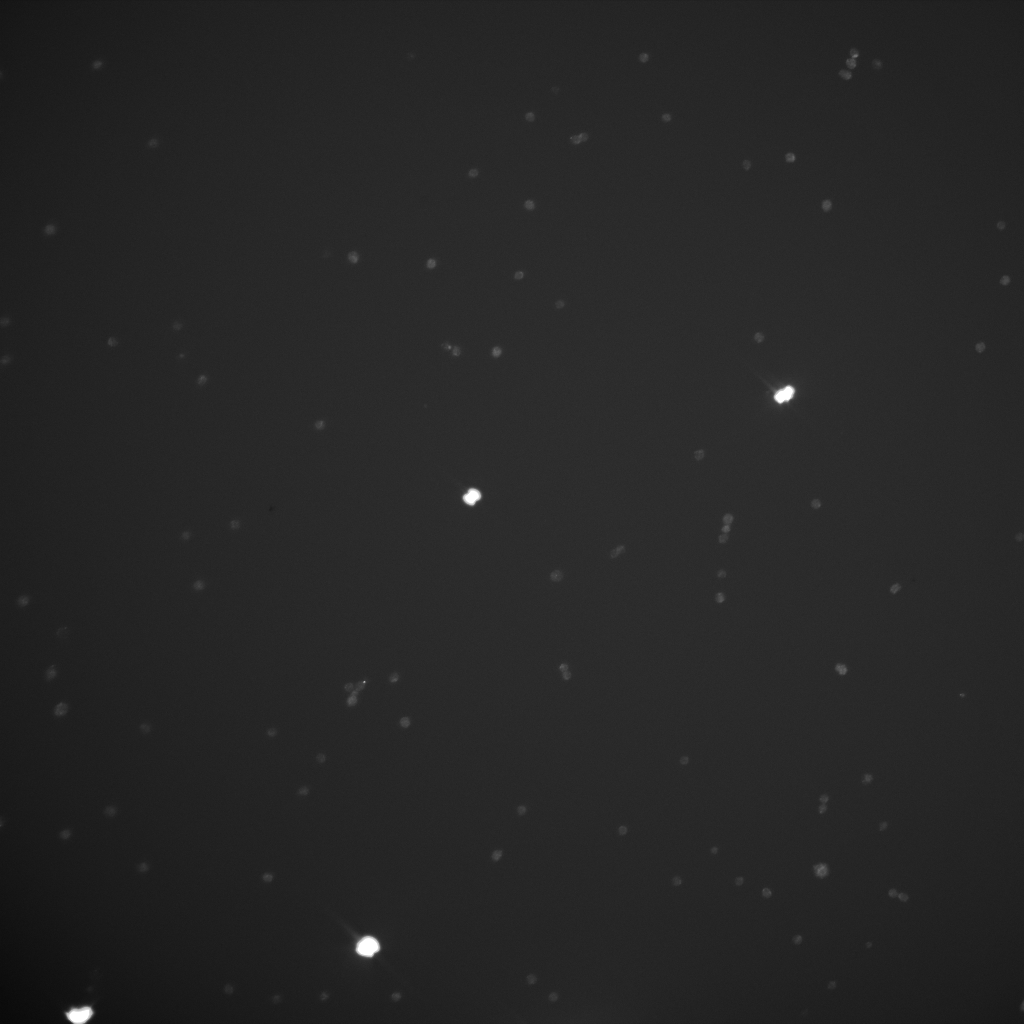

Supplement: Supplementary file 18 [file msb0011-0783-sd18.zip › Snap-144_c3_ORG.png]

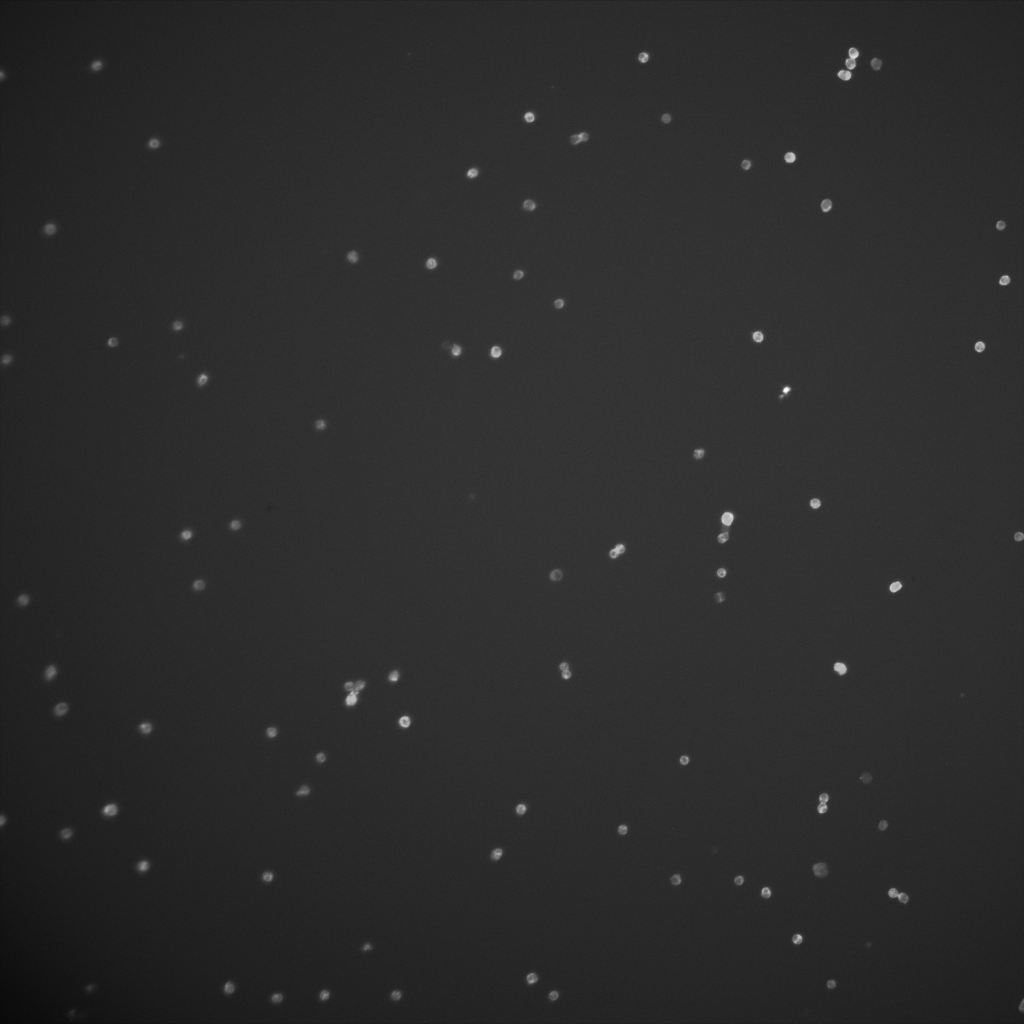

Supplement: Supplementary file 18 [file msb0011-0783-sd18.zip › Snap-144_c4_ORG.png]

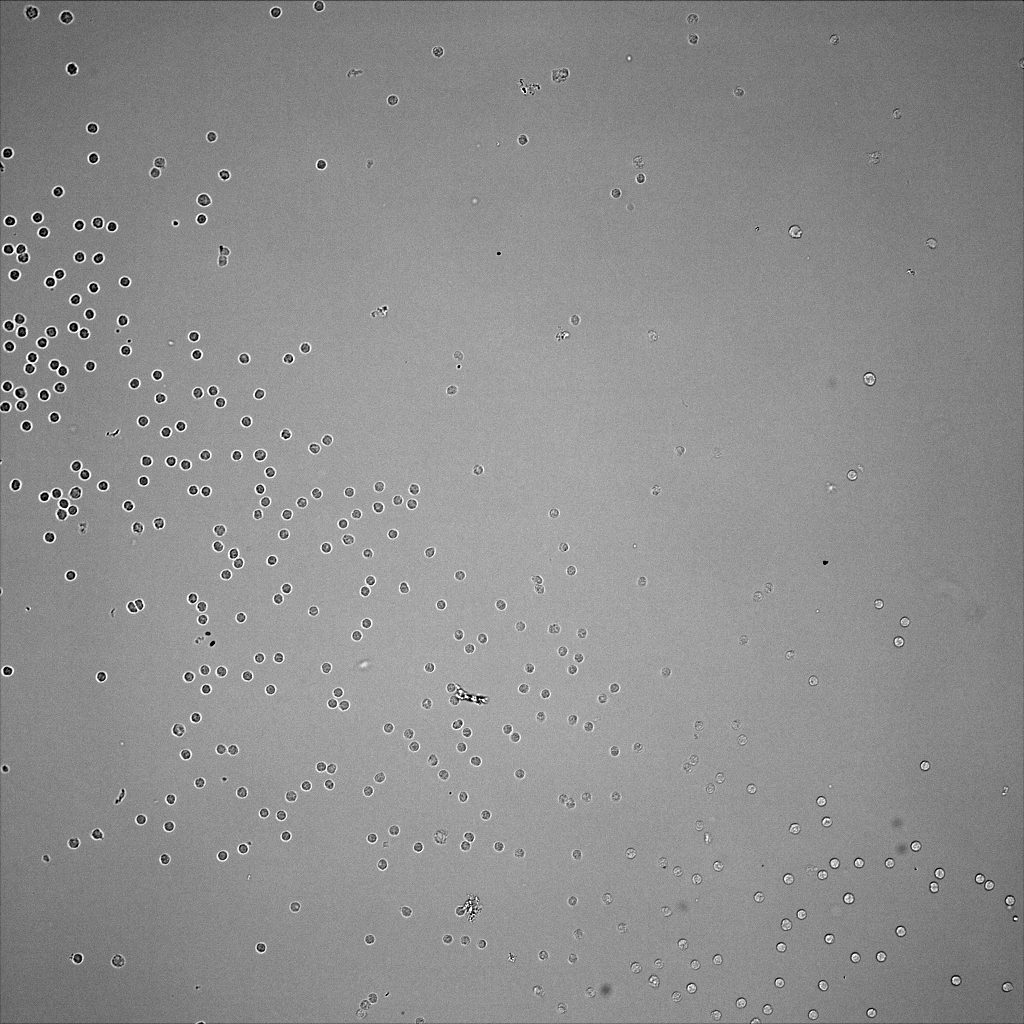

Supplement: Supplementary file 18 [file msb0011-0783-sd18.zip › Snap-145_c1_ORG.png]

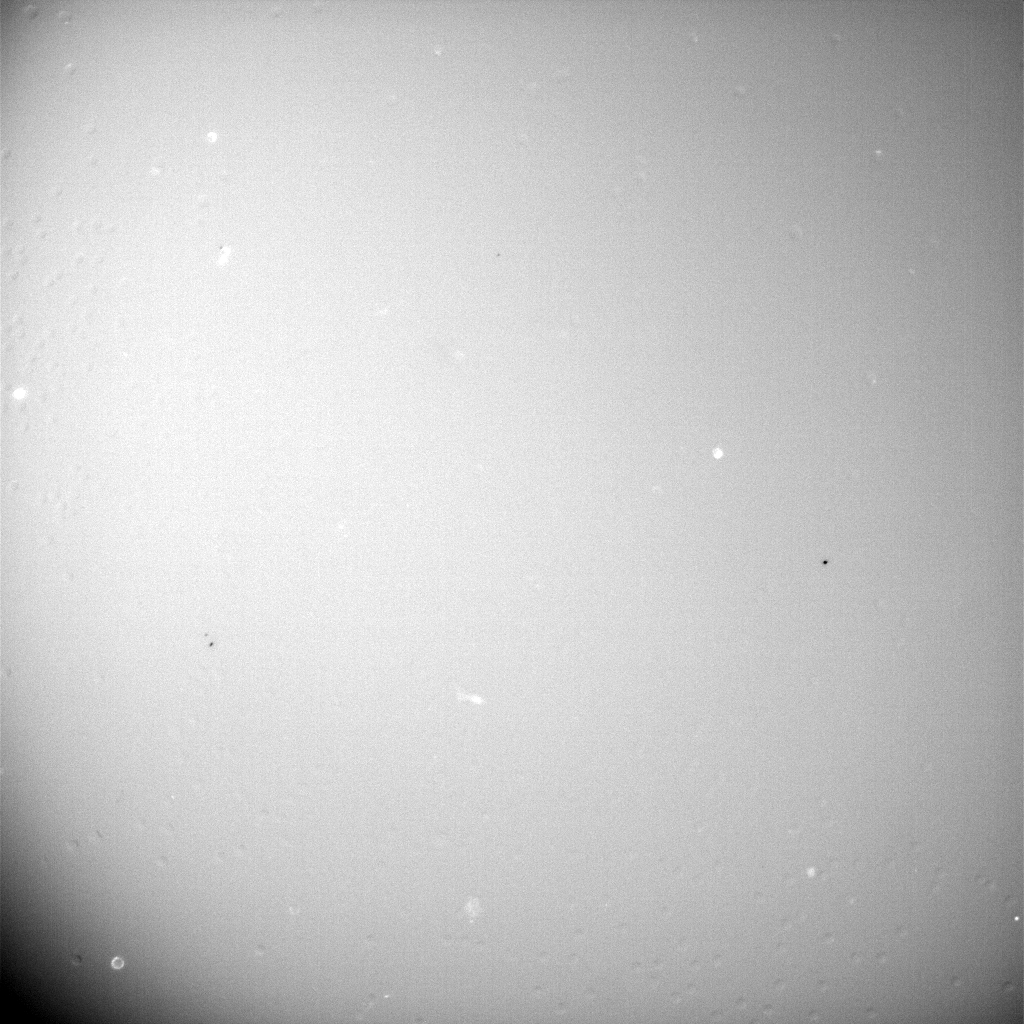

Supplement: Supplementary file 18 [file msb0011-0783-sd18.zip › Snap-145_c2_ORG.png]

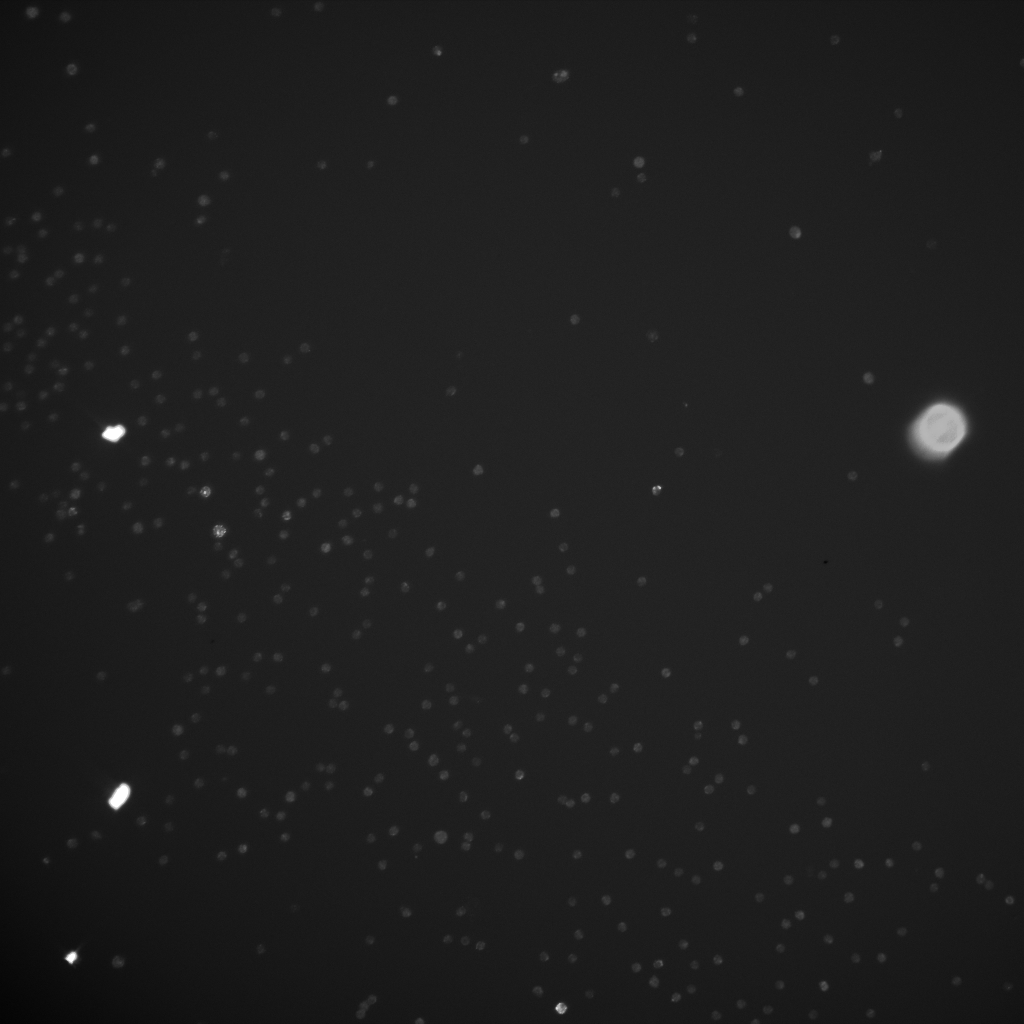

Supplement: Supplementary file 18 [file msb0011-0783-sd18.zip › Snap-145_c3_ORG.png]

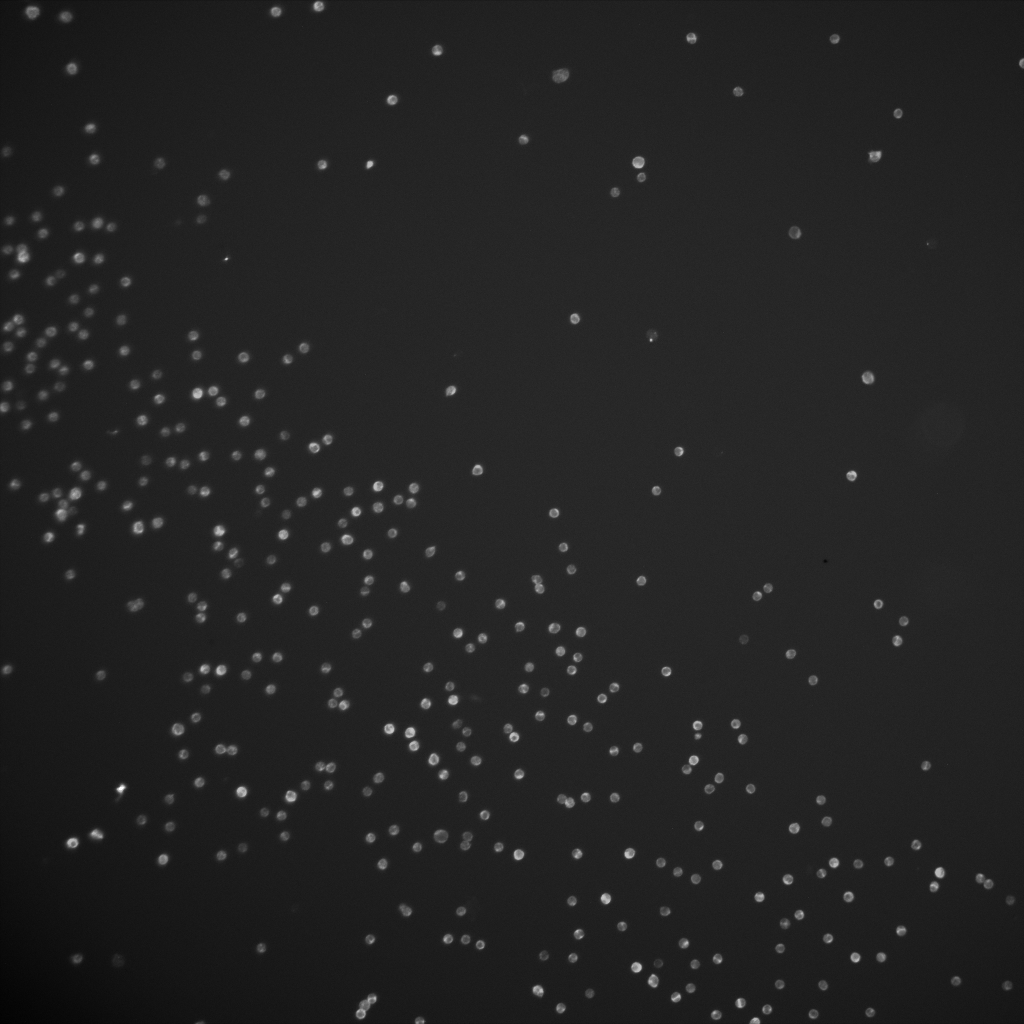

Supplement: Supplementary file 18 [file msb0011-0783-sd18.zip › Snap-145_c4_ORG.png]

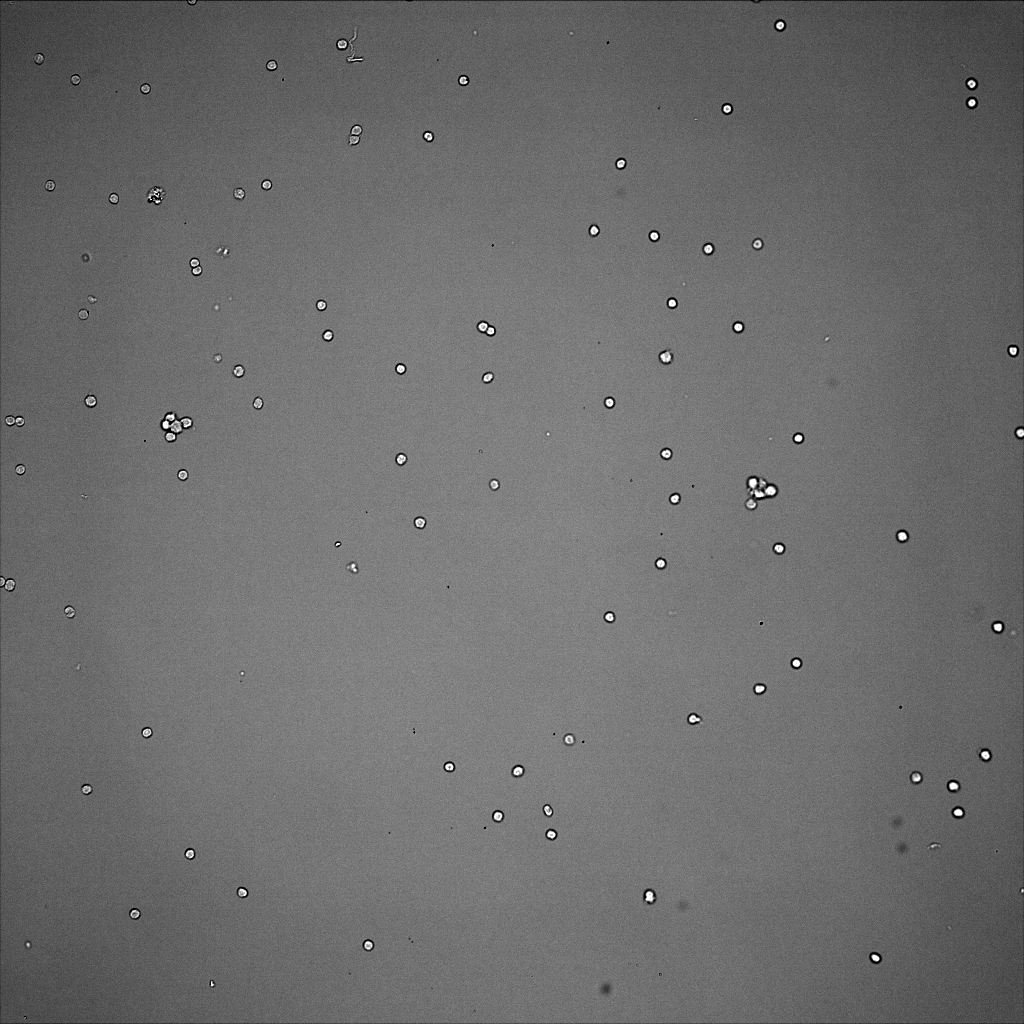

Supplement: Supplementary file 18 [file msb0011-0783-sd18.zip › Snap-146_c1_ORG.png]

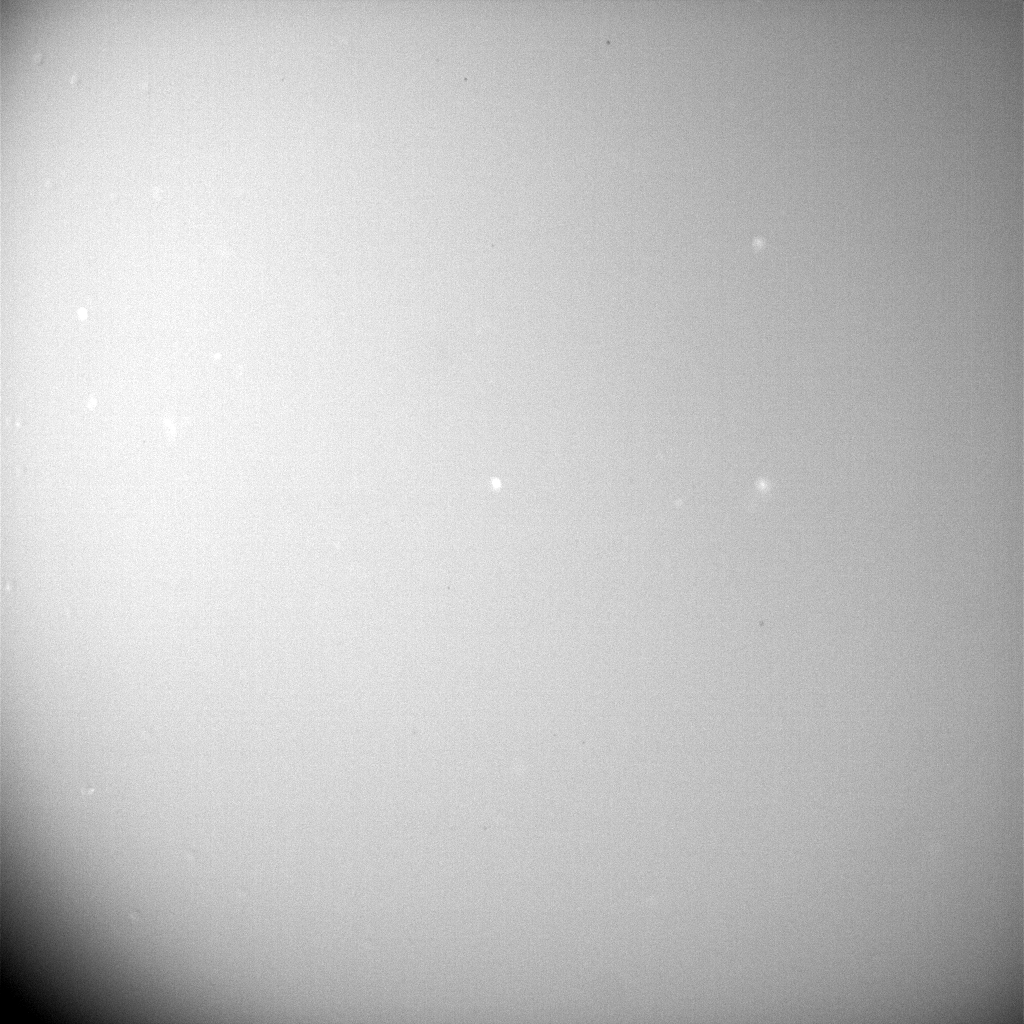

Supplement: Supplementary file 18 [file msb0011-0783-sd18.zip › Snap-146_c2_ORG.png]

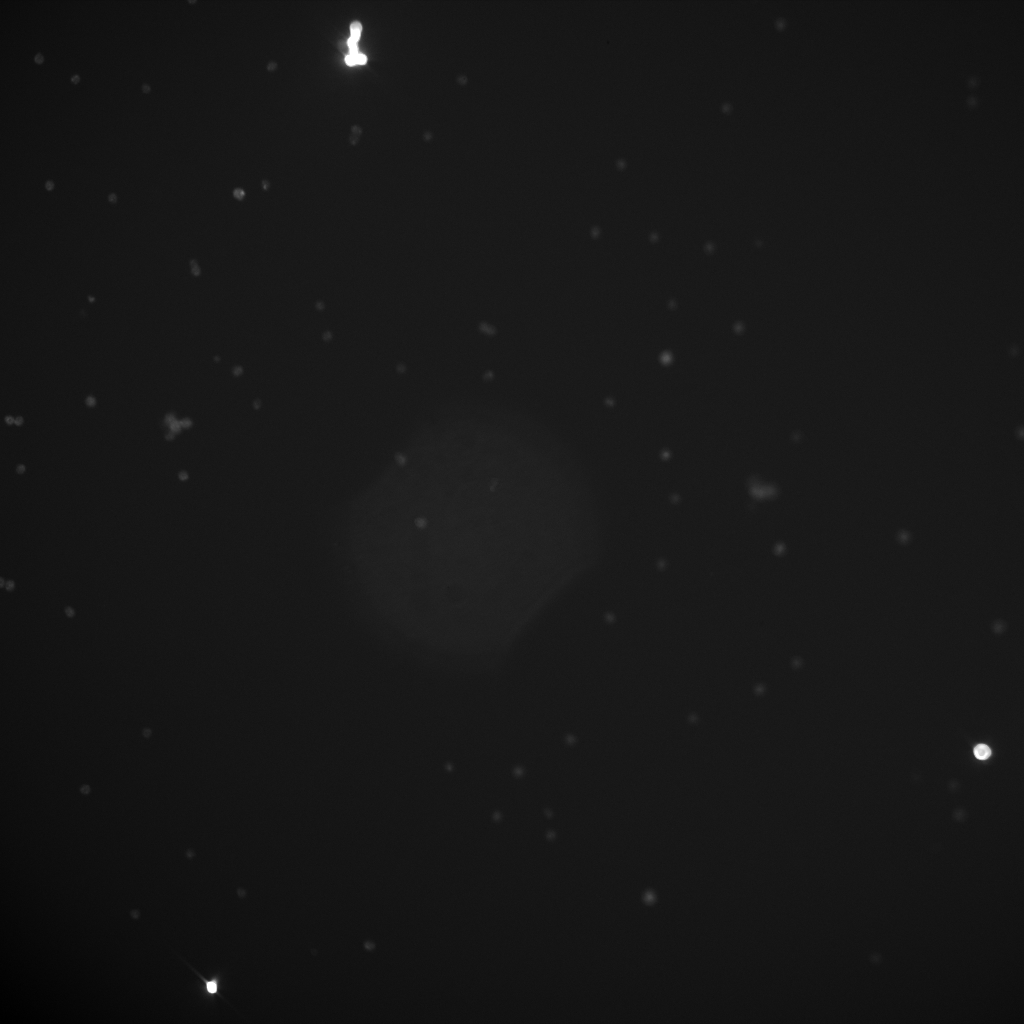

Supplement: Supplementary file 18 [file msb0011-0783-sd18.zip › Snap-146_c3_ORG.png]

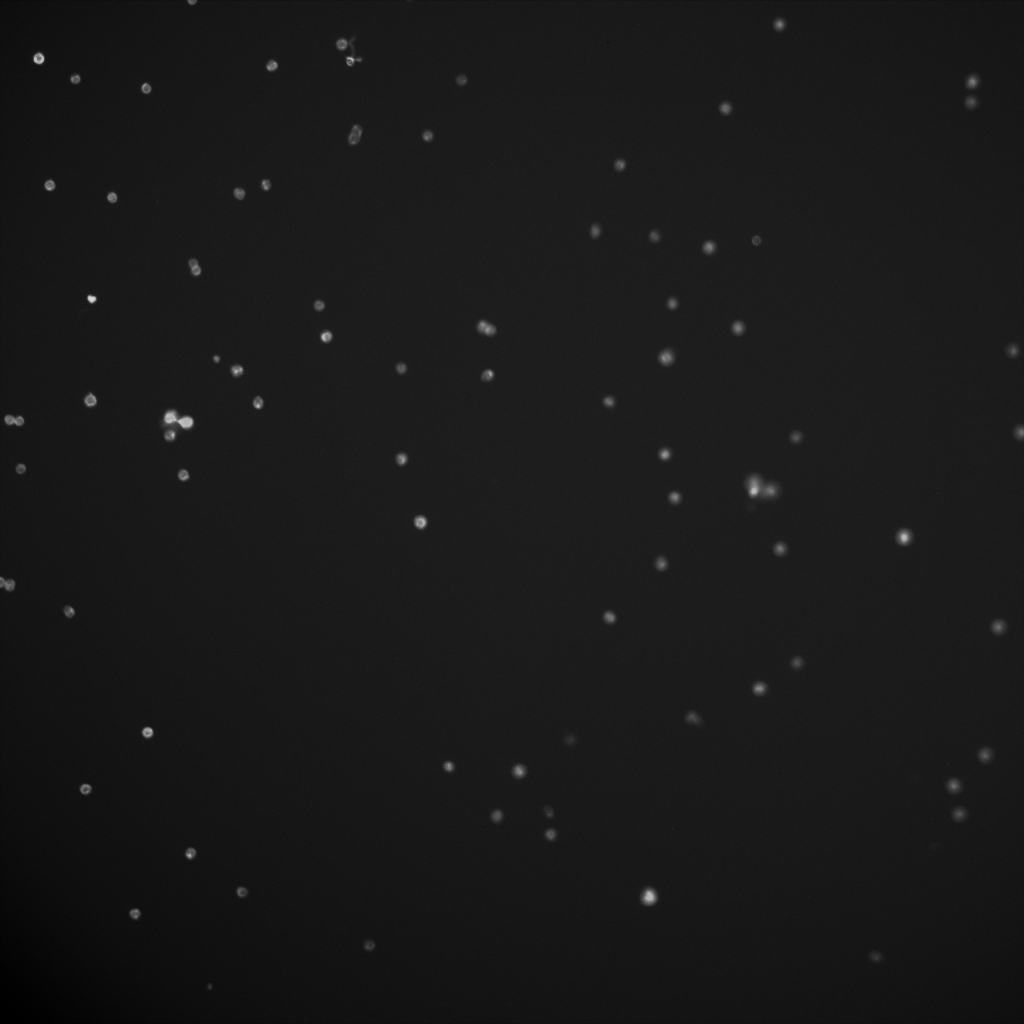

Supplement: Supplementary file 18 [file msb0011-0783-sd18.zip › Snap-146_c4_ORG.png]

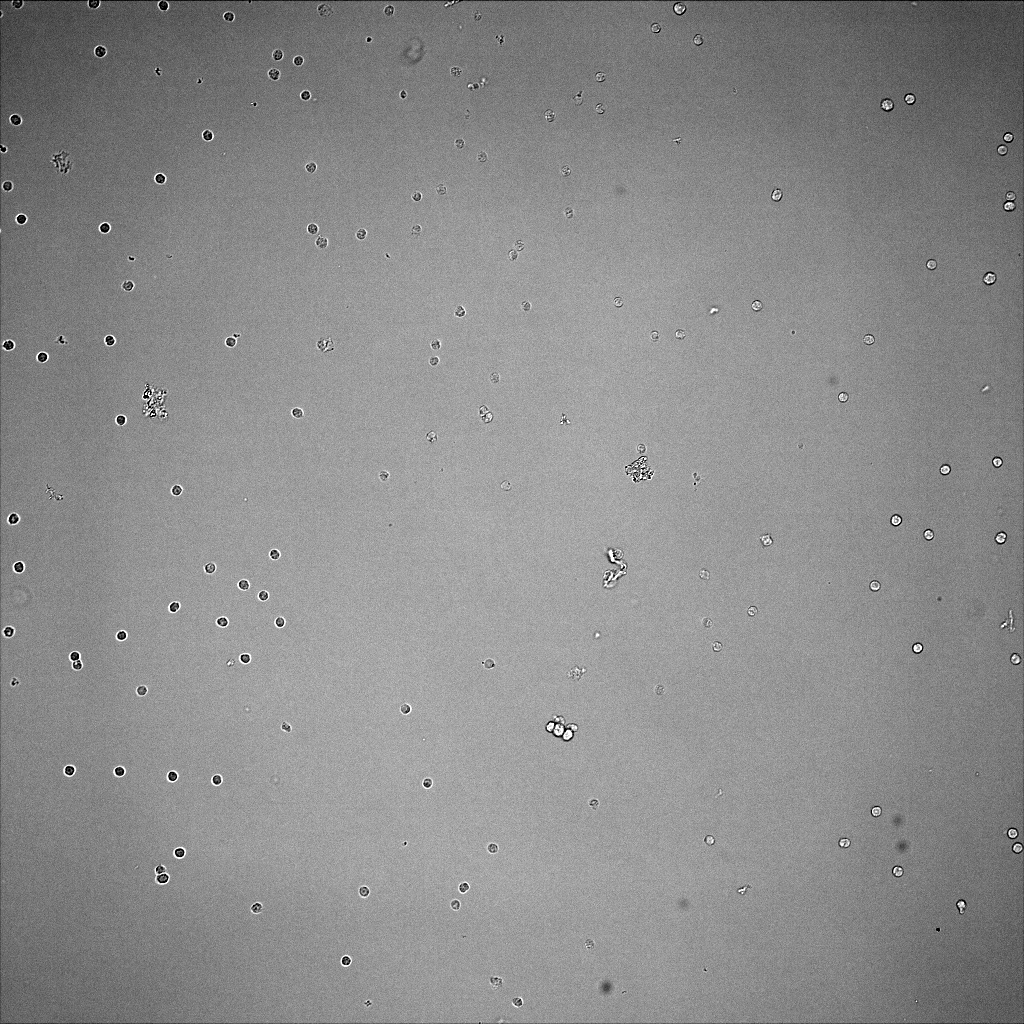

Supplement: Supplementary file 18 [file msb0011-0783-sd18.zip › Snap-147_c1_ORG.png]

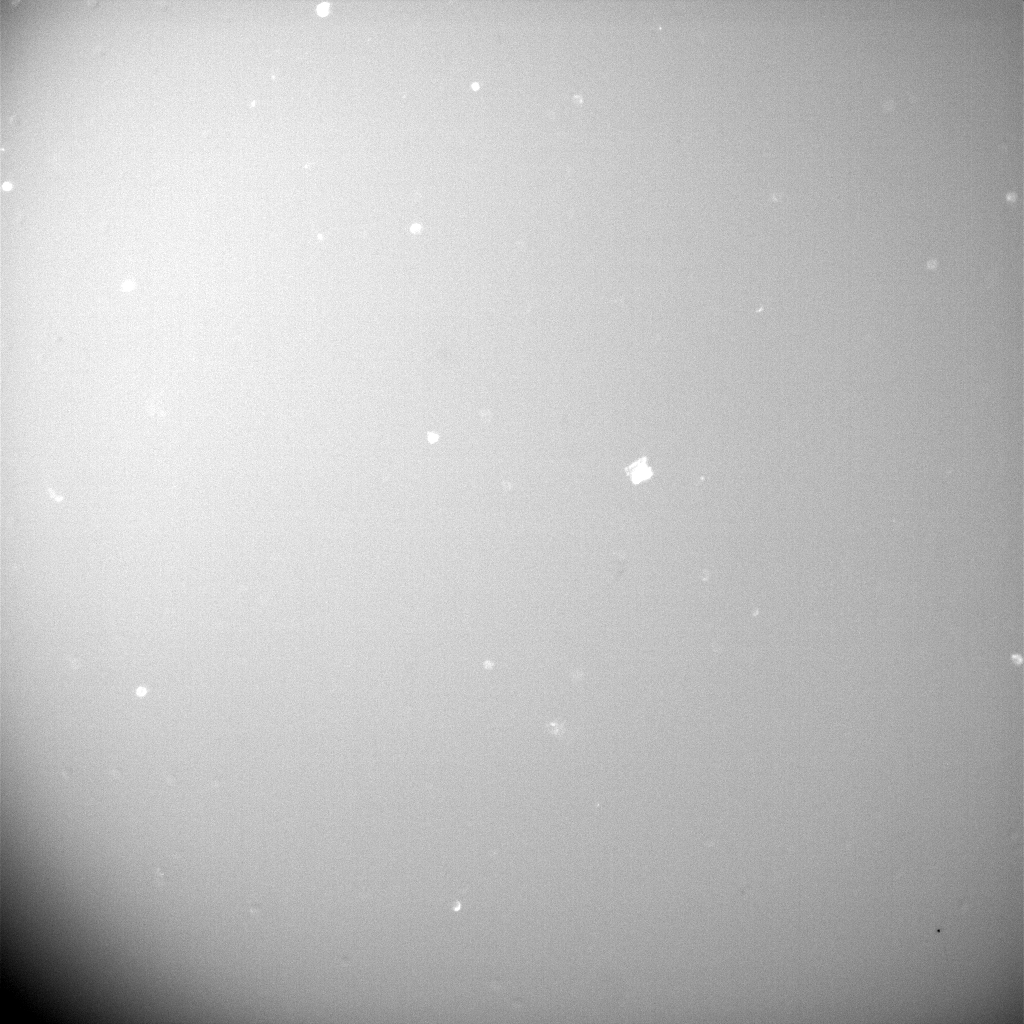

Supplement: Supplementary file 18 [file msb0011-0783-sd18.zip › Snap-147_c2_ORG.png]

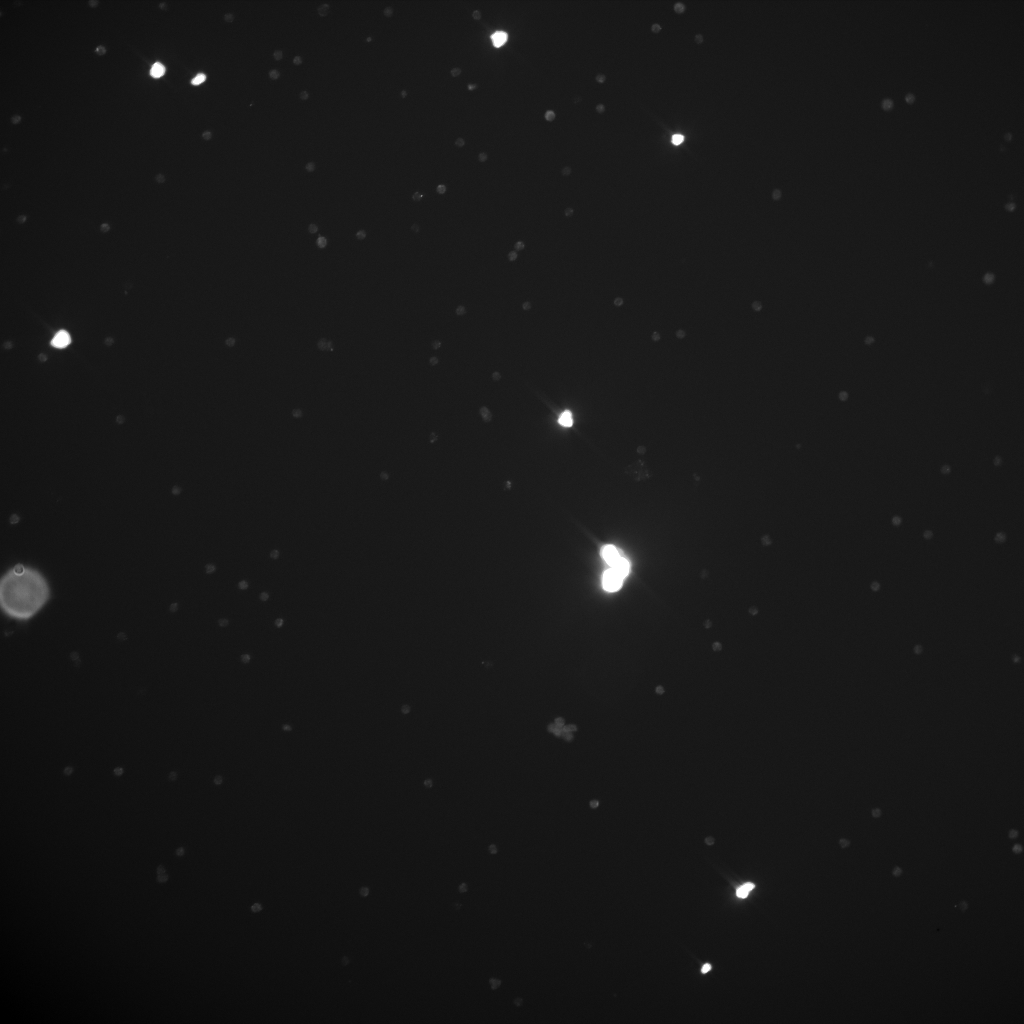

Supplement: Supplementary file 18 [file msb0011-0783-sd18.zip › Snap-147_c3_ORG.png]

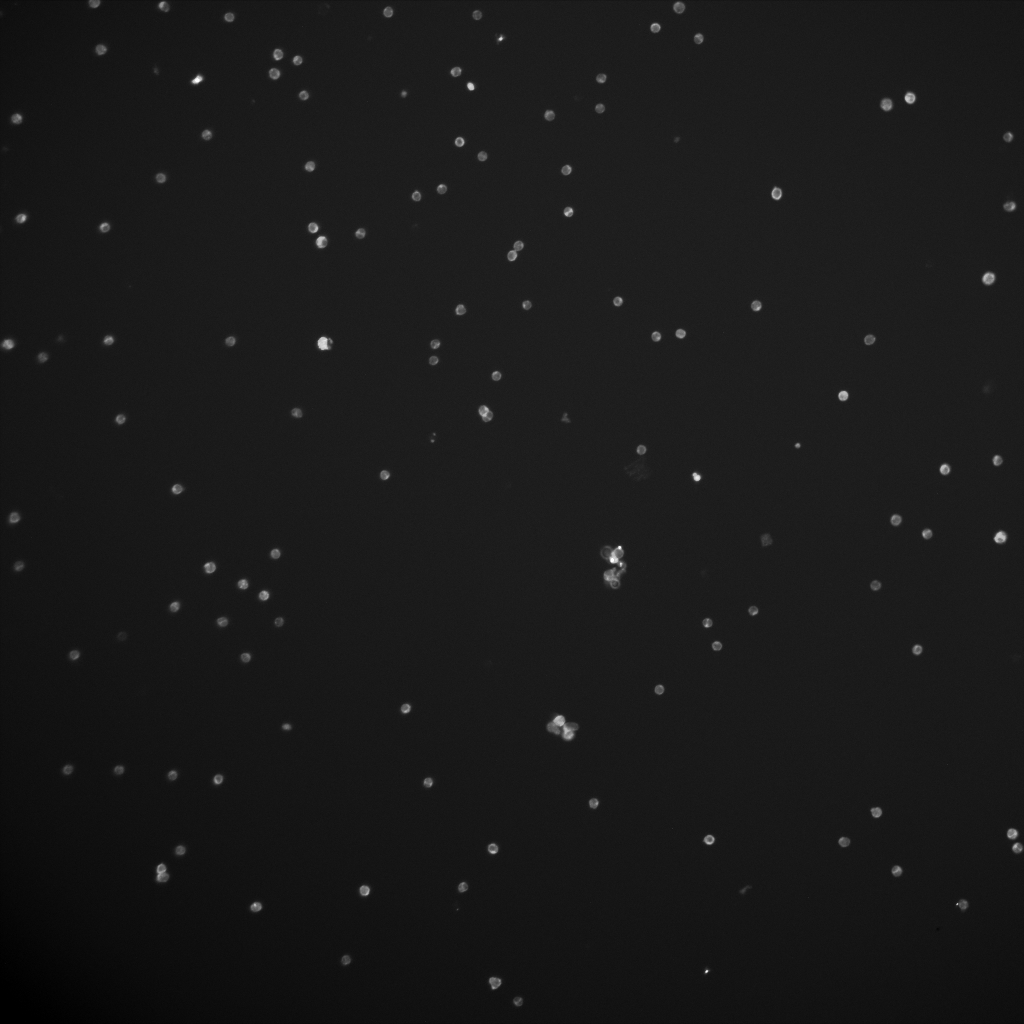

Supplement: Supplementary file 18 [file msb0011-0783-sd18.zip › Snap-147_c4_ORG.png]

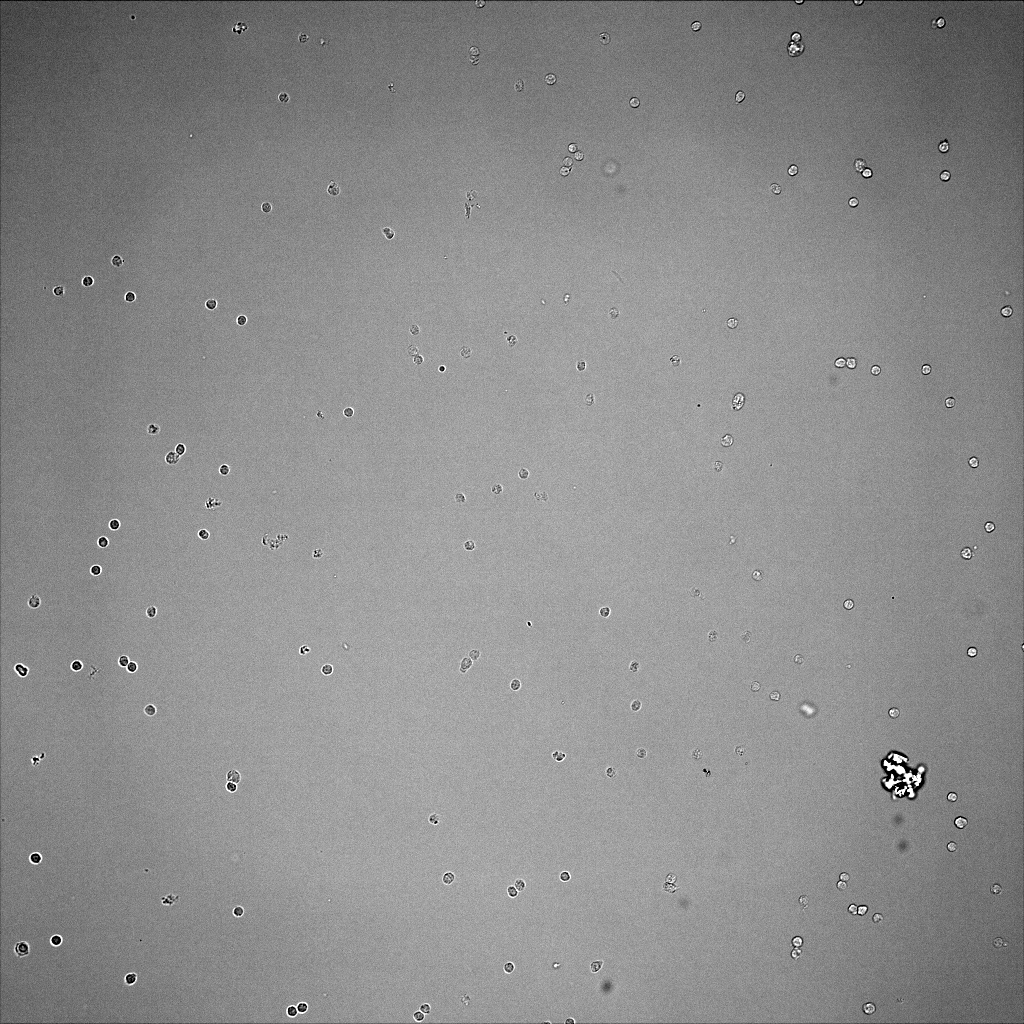

Supplement: Supplementary file 18 [file msb0011-0783-sd18.zip › Snap-148_c1_ORG.png]

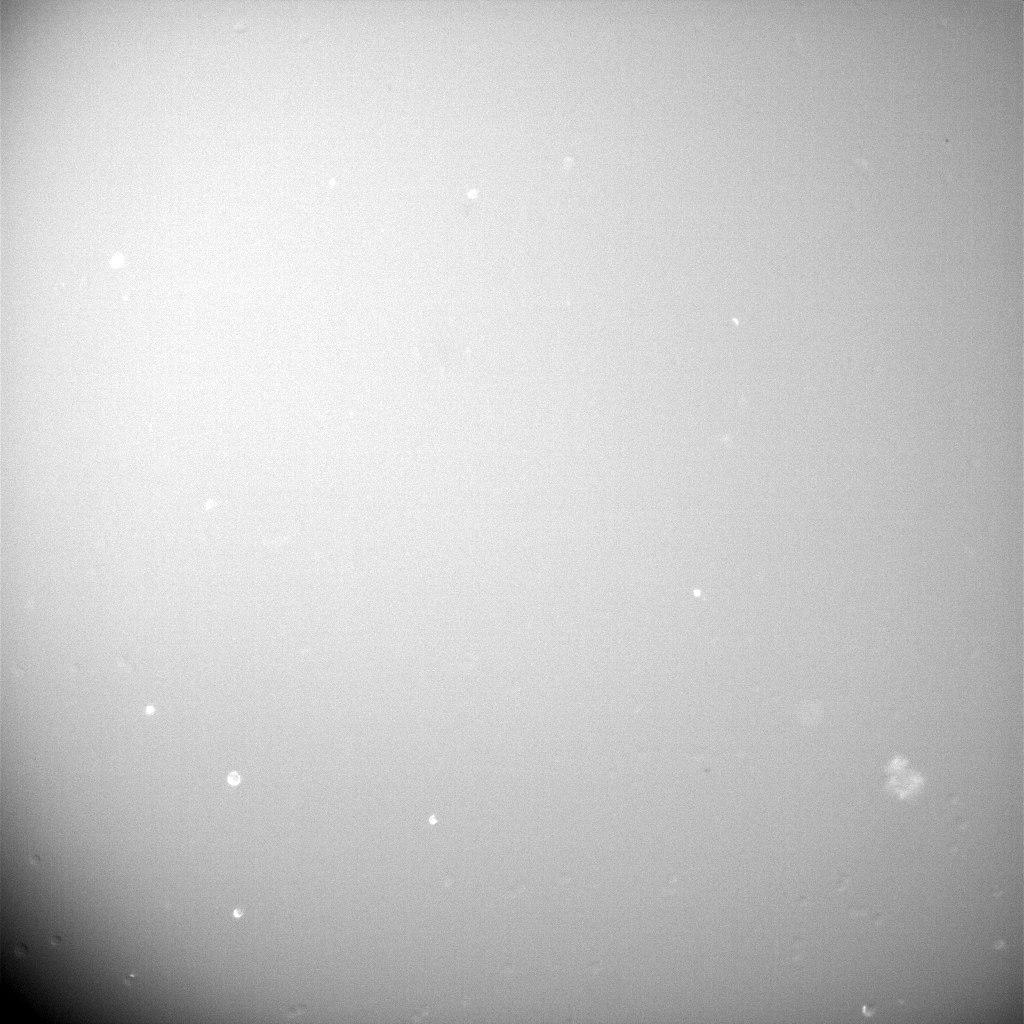

Supplement: Supplementary file 18 [file msb0011-0783-sd18.zip › Snap-148_c2_ORG.png]

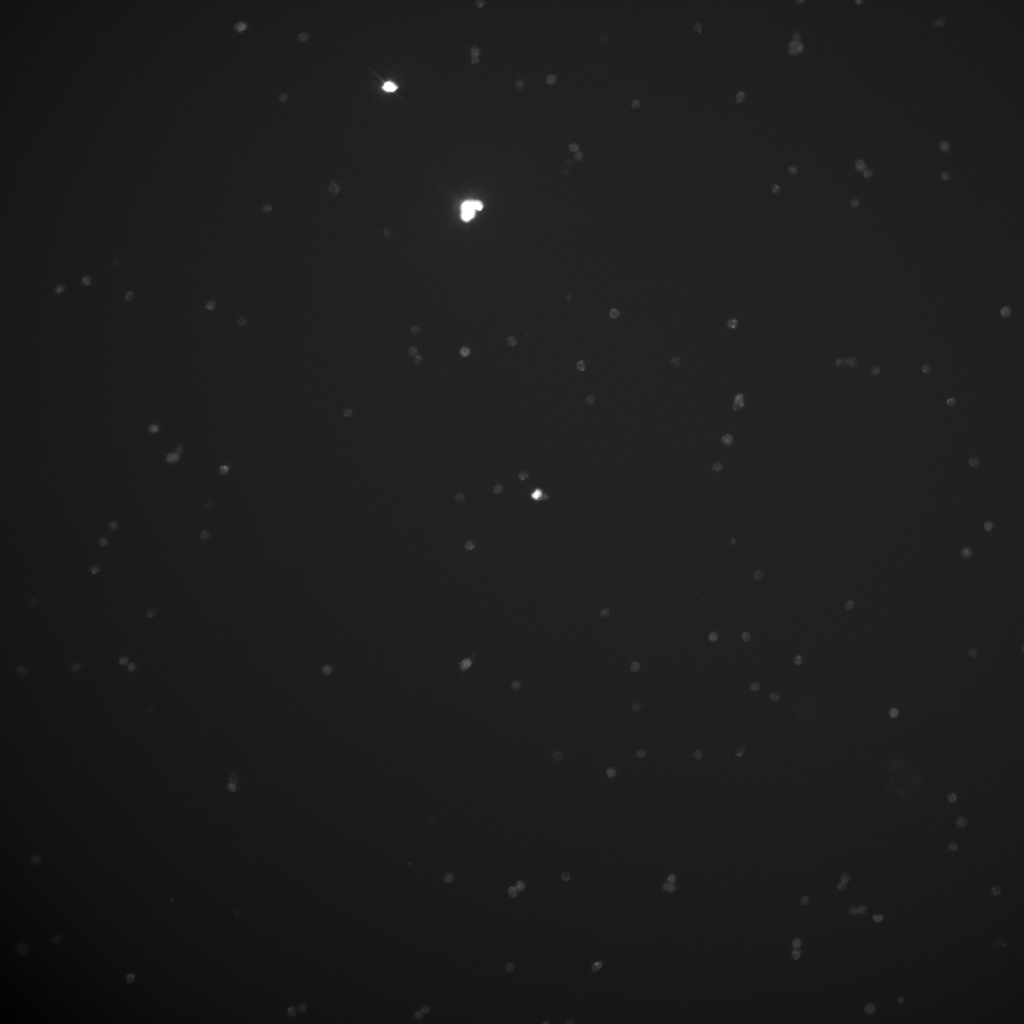

Supplement: Supplementary file 18 [file msb0011-0783-sd18.zip › Snap-148_c3_ORG.png]

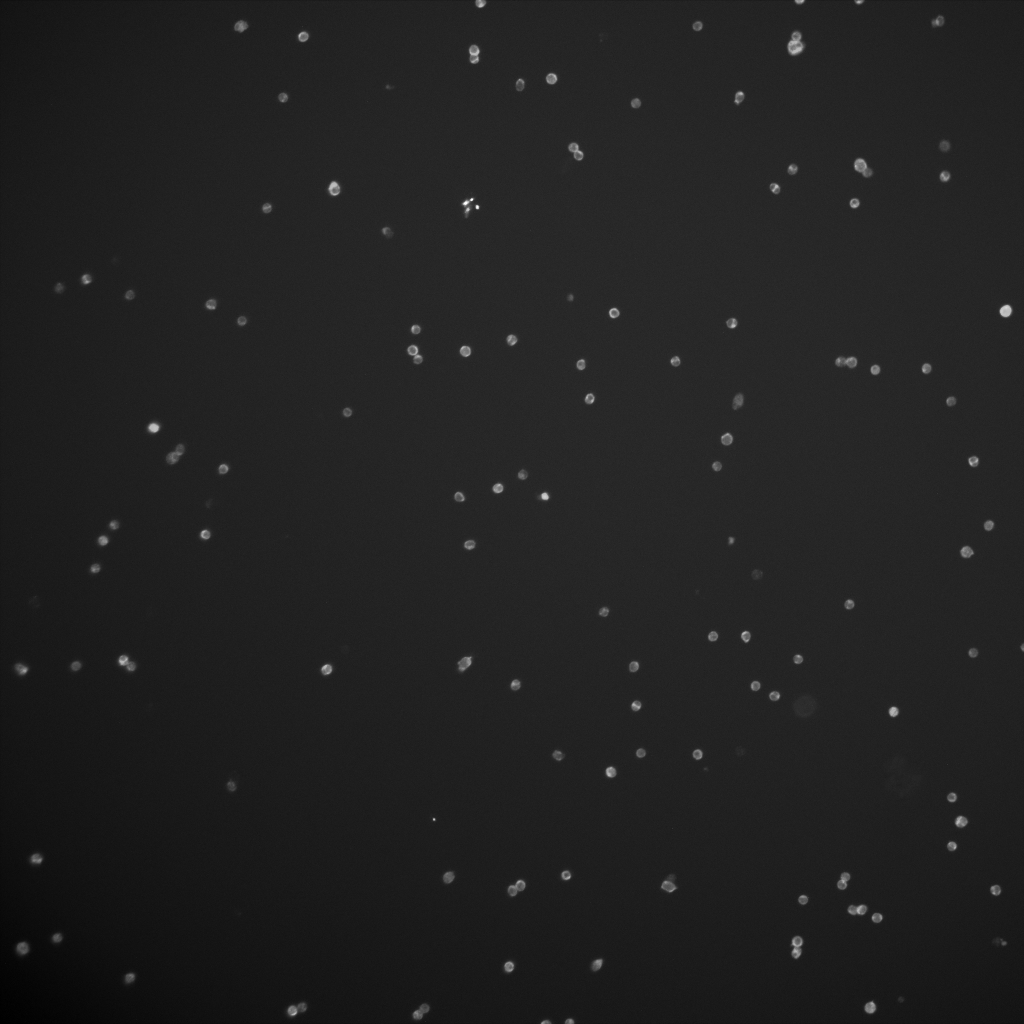

Supplement: Supplementary file 18 [file msb0011-0783-sd18.zip › Snap-148_c4_ORG.png]

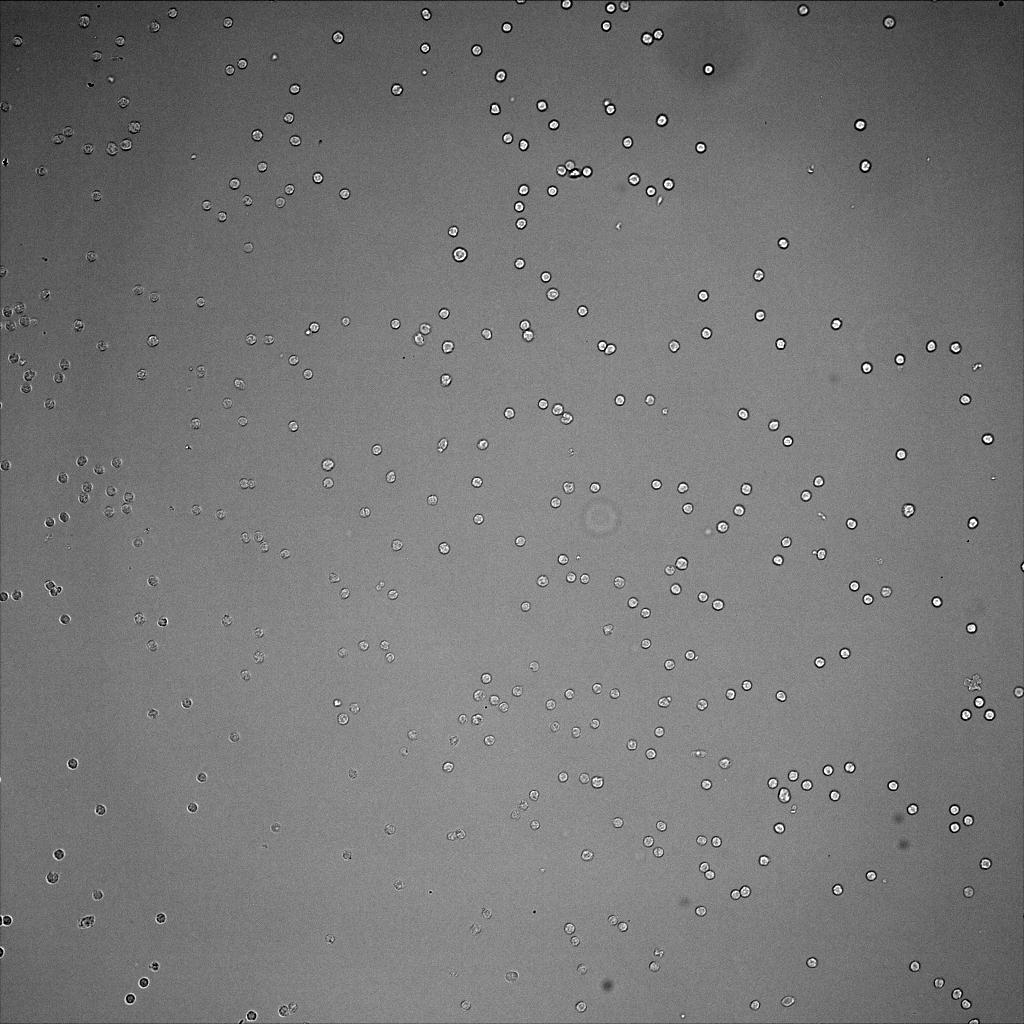

Supplement: Supplementary file 18 [file msb0011-0783-sd18.zip › Snap-149_c1_ORG.png]

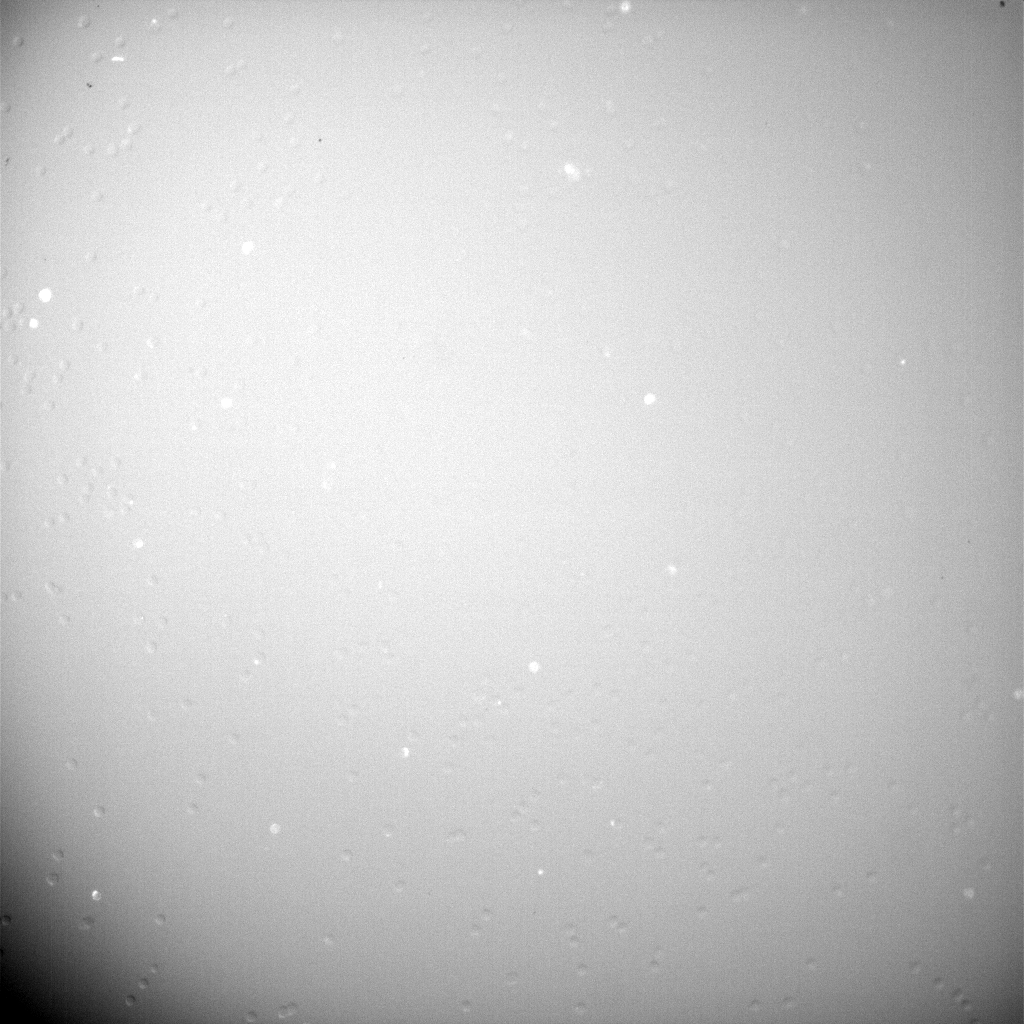

Supplement: Supplementary file 18 [file msb0011-0783-sd18.zip › Snap-149_c2_ORG.png]

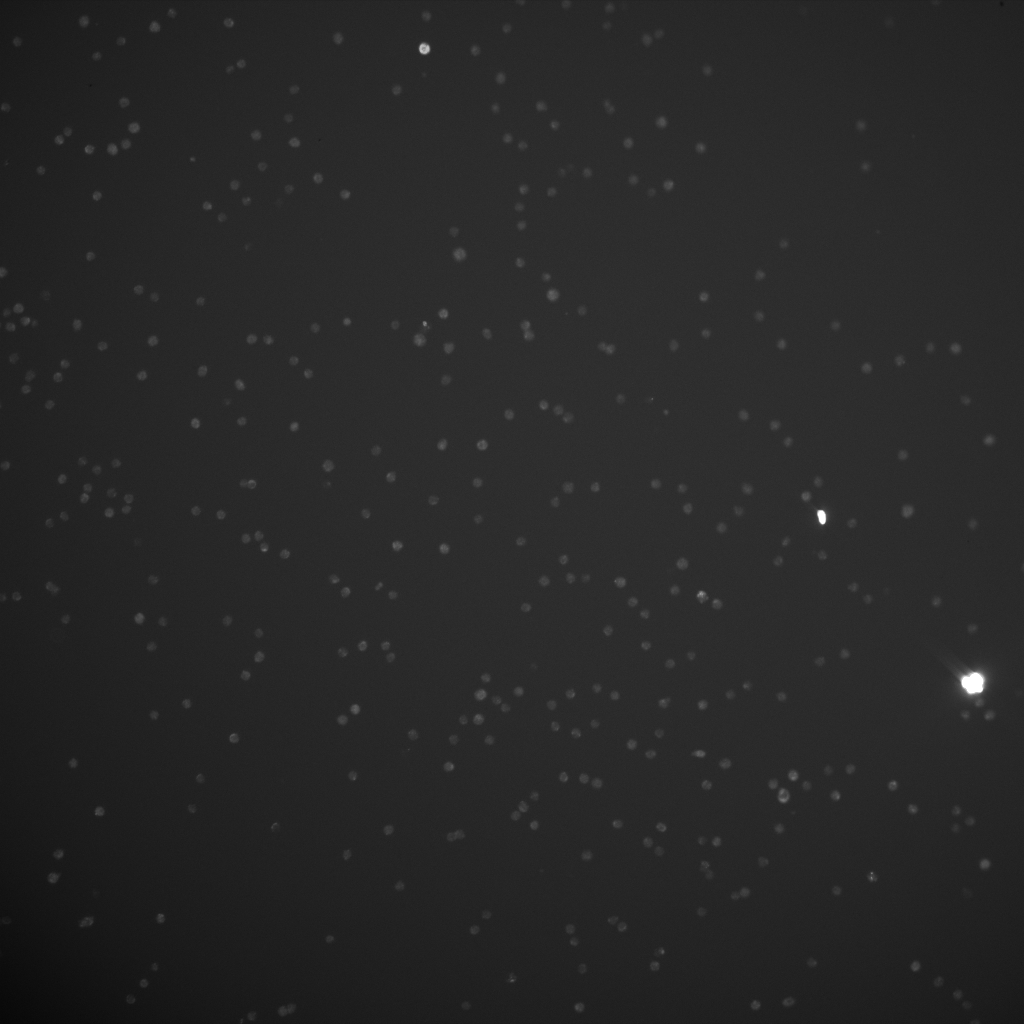

Supplement: Supplementary file 18 [file msb0011-0783-sd18.zip › Snap-149_c3_ORG.png]

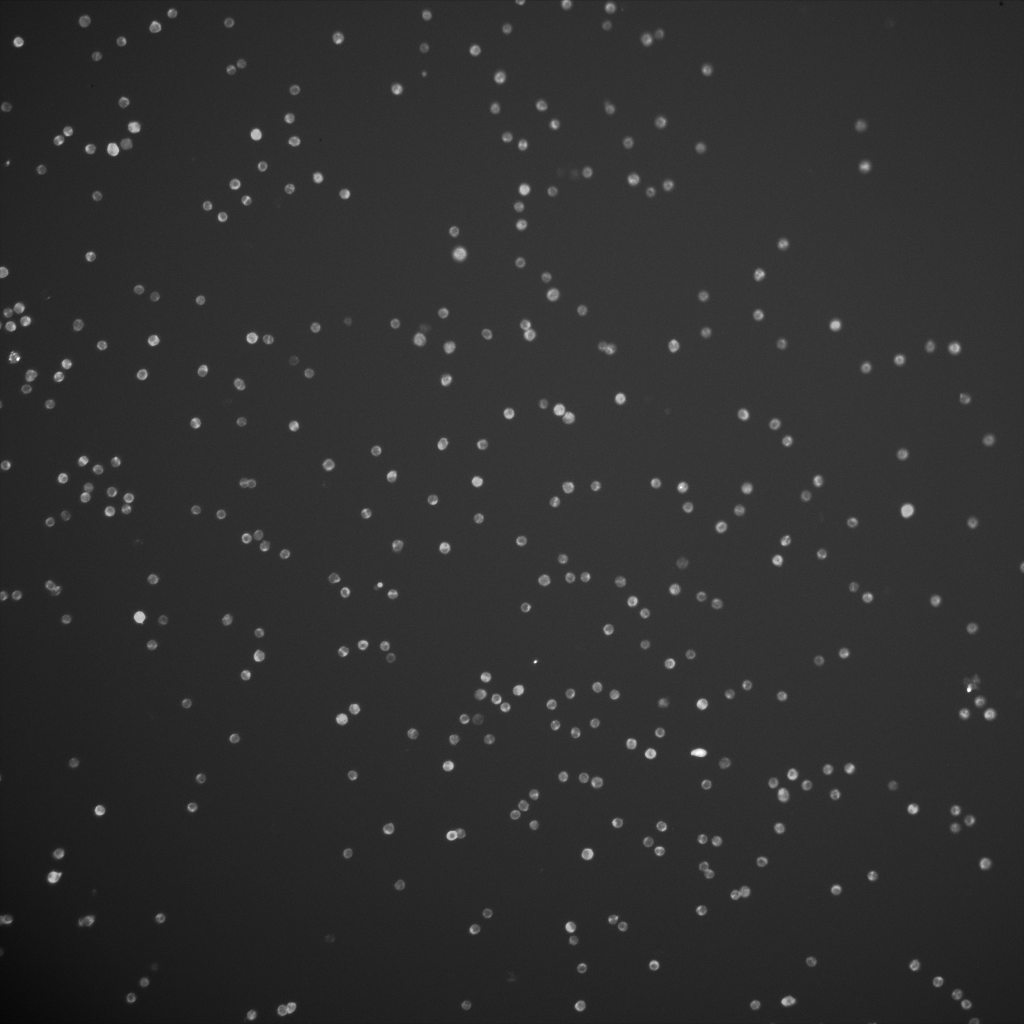

Supplement: Supplementary file 18 [file msb0011-0783-sd18.zip › Snap-149_c4_ORG.png]

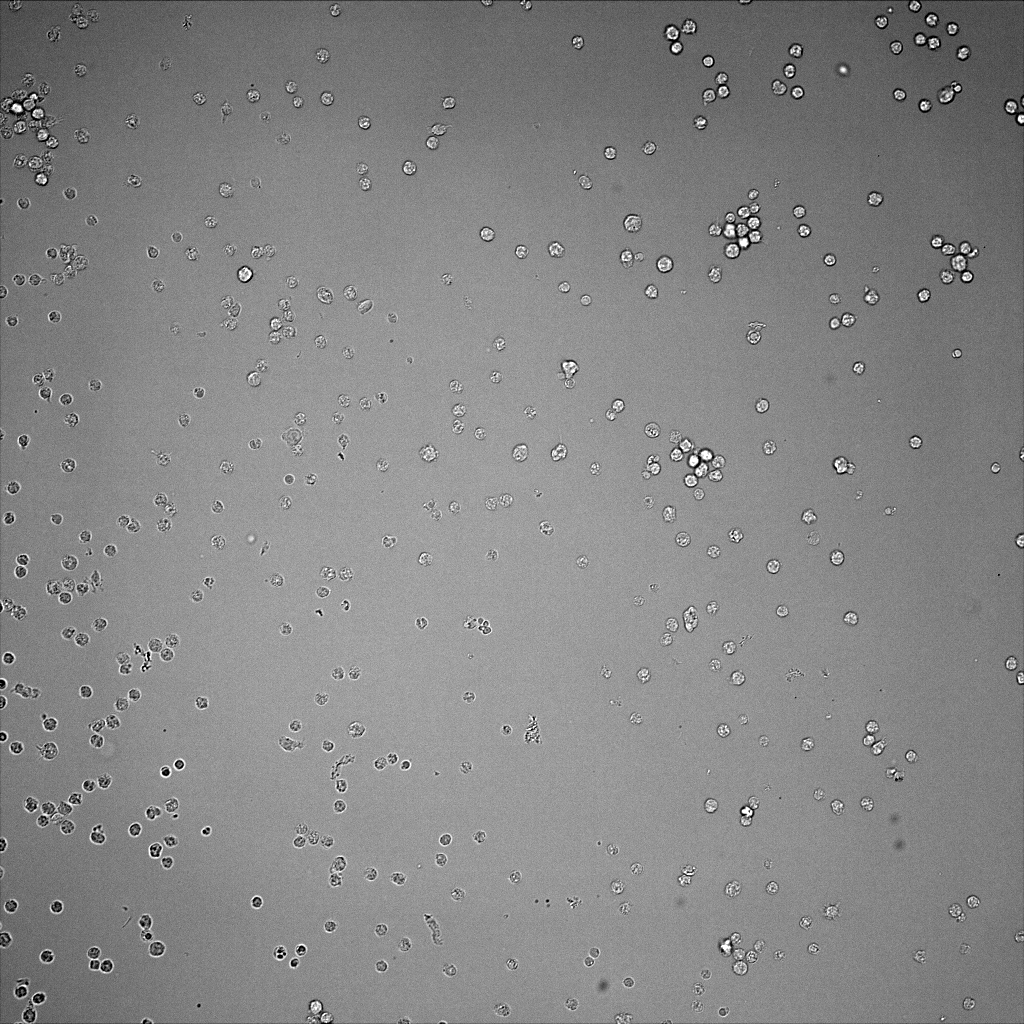

Supplement: Supplementary file 18 [file msb0011-0783-sd18.zip › Snap-150_c1_ORG.png]

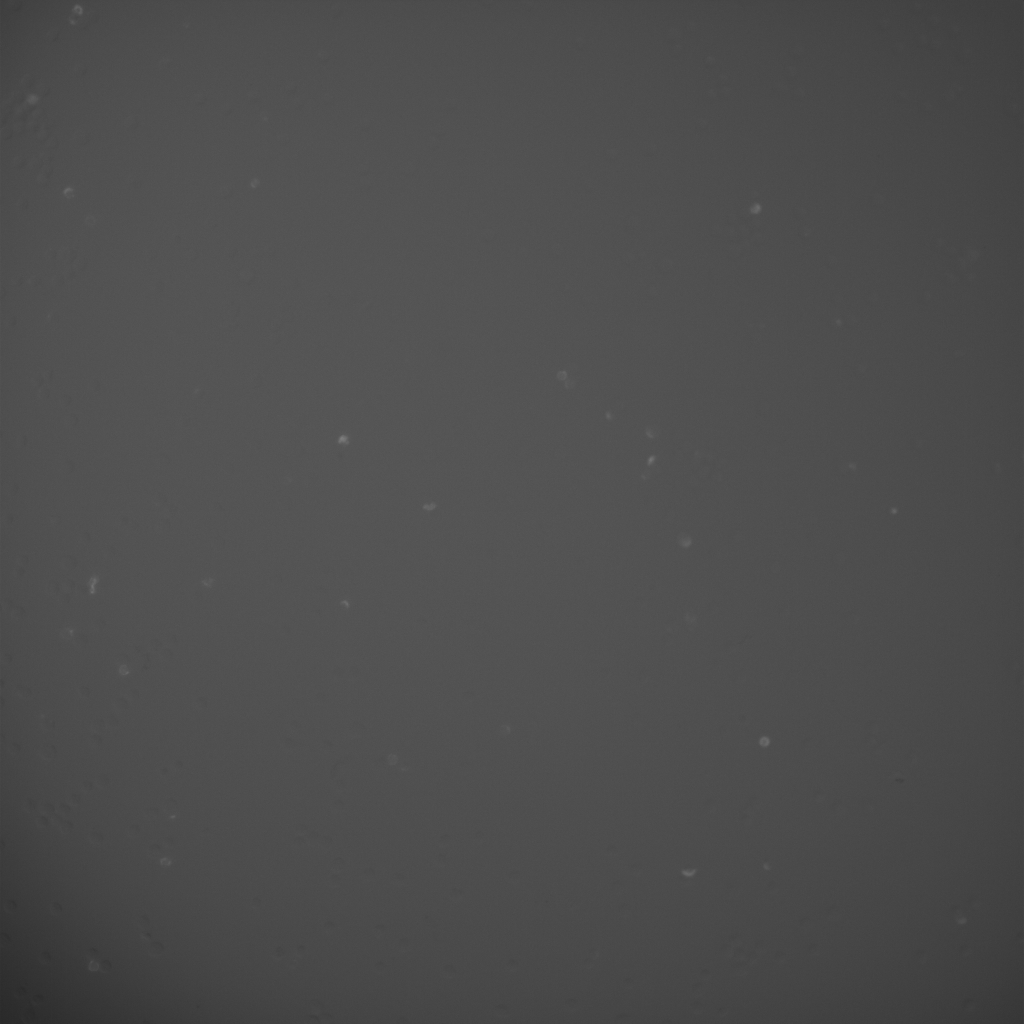

Supplement: Supplementary file 18 [file msb0011-0783-sd18.zip › Snap-150_c2_ORG.png]

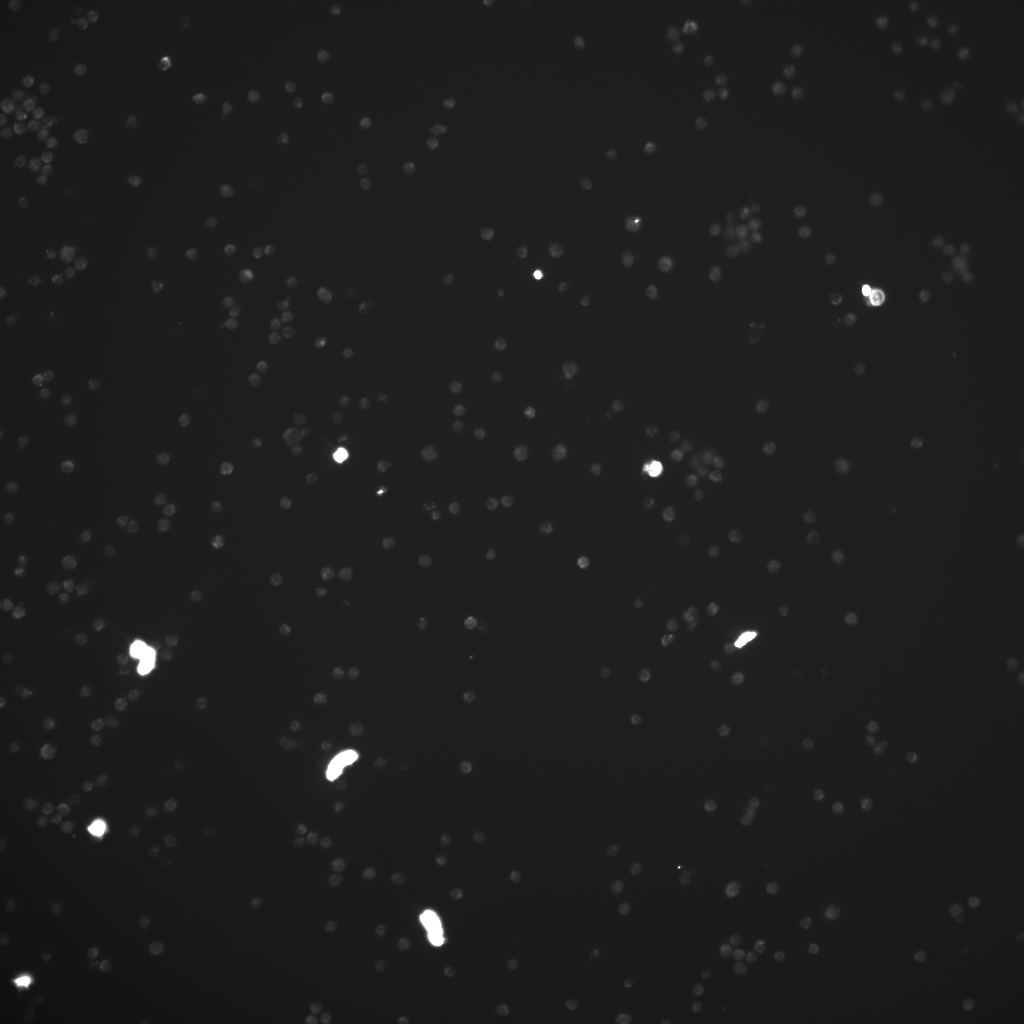

Supplement: Supplementary file 18 [file msb0011-0783-sd18.zip › Snap-150_c3_ORG.png]

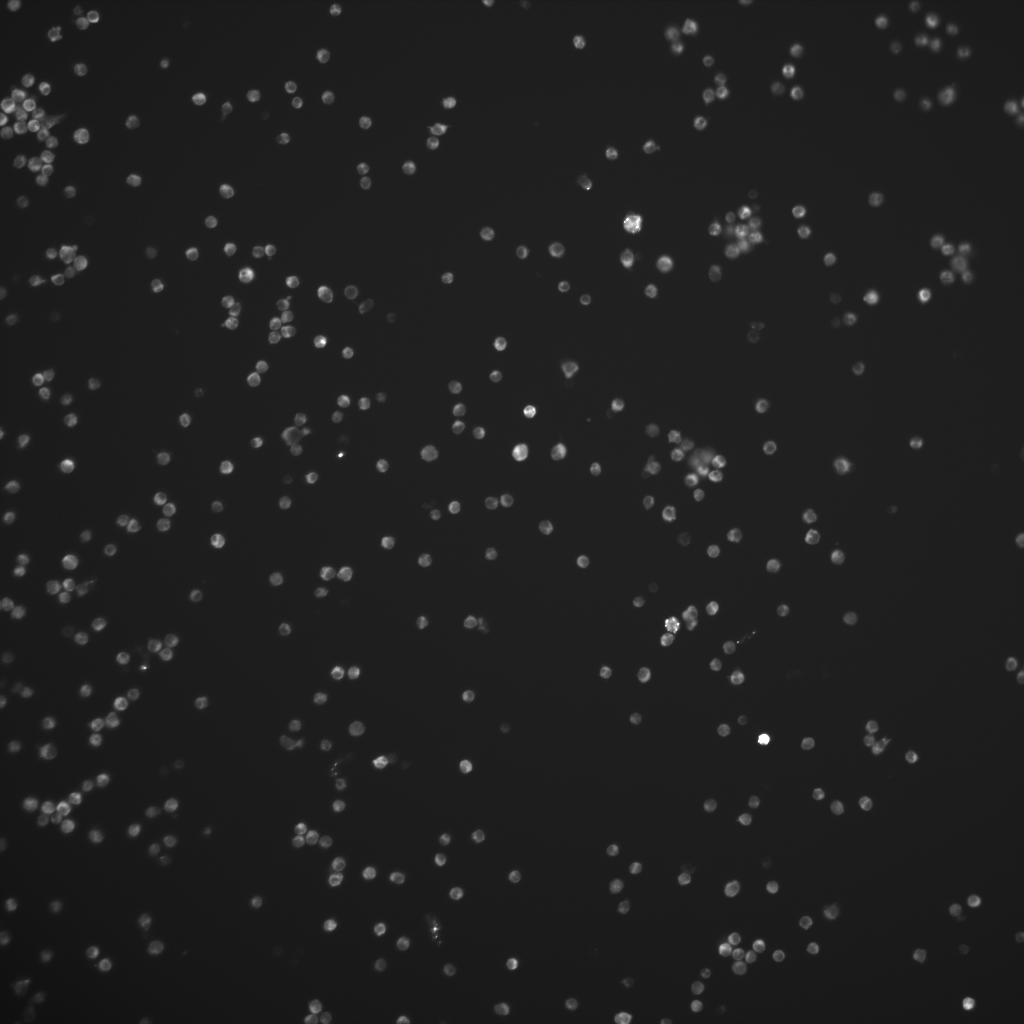

Supplement: Supplementary file 18 [file msb0011-0783-sd18.zip › Snap-150_c4_ORG.png]

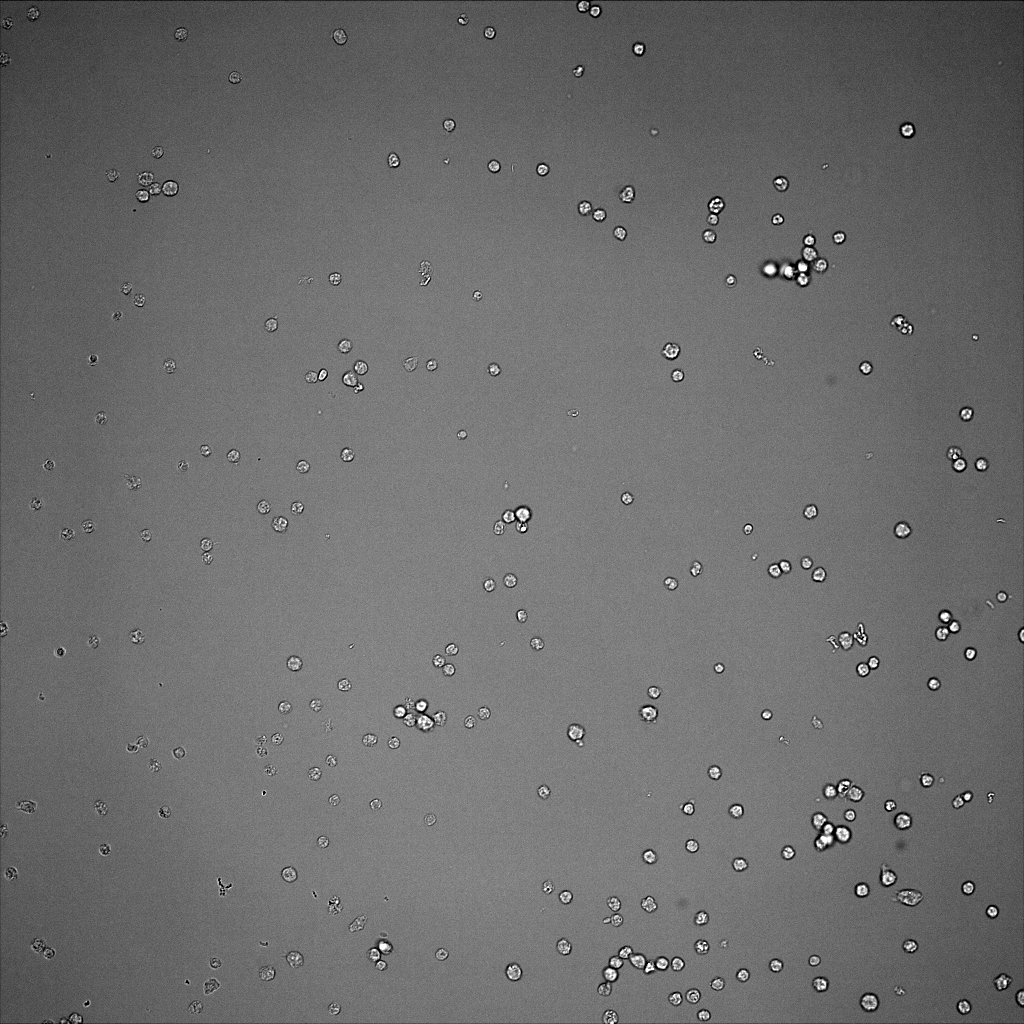

Supplement: Supplementary file 18 [file msb0011-0783-sd18.zip › Snap-151_c1_ORG.png]

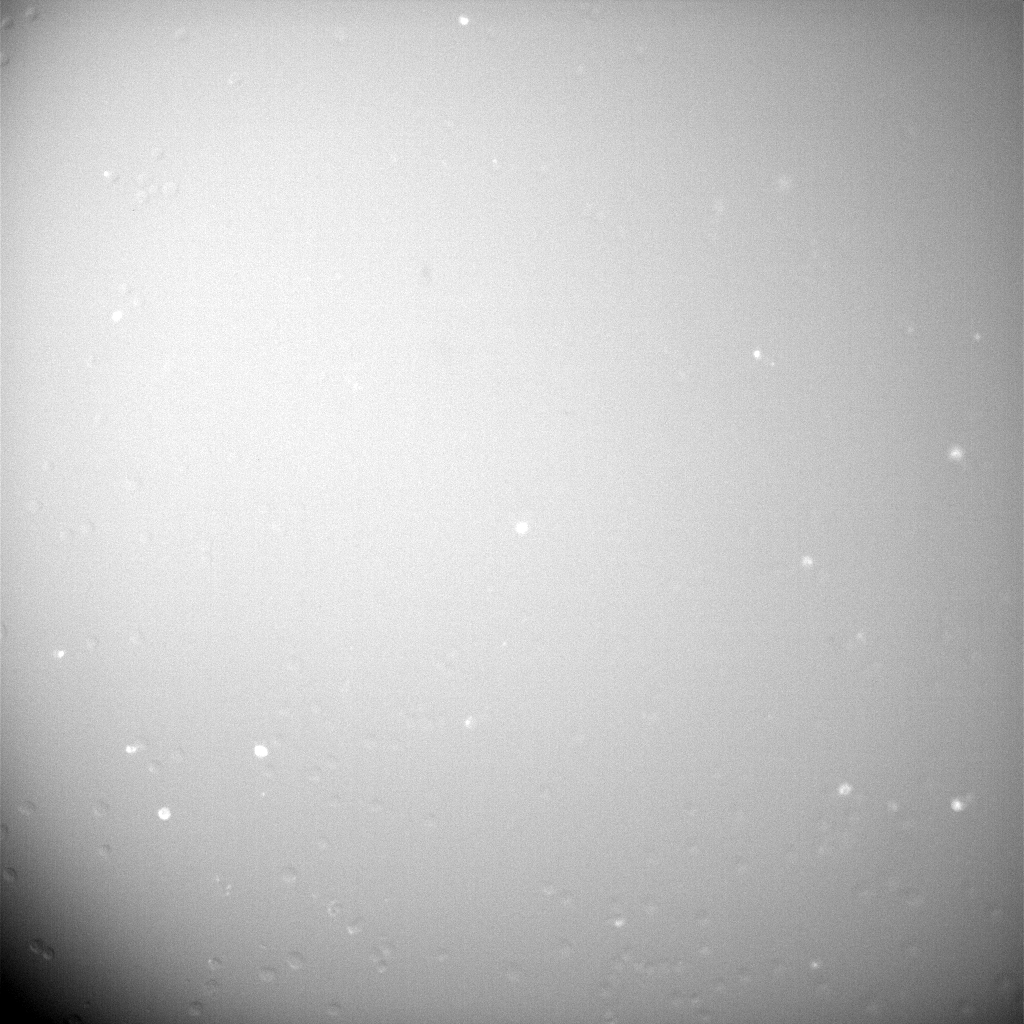

Supplement: Supplementary file 18 [file msb0011-0783-sd18.zip › Snap-151_c2_ORG.png]

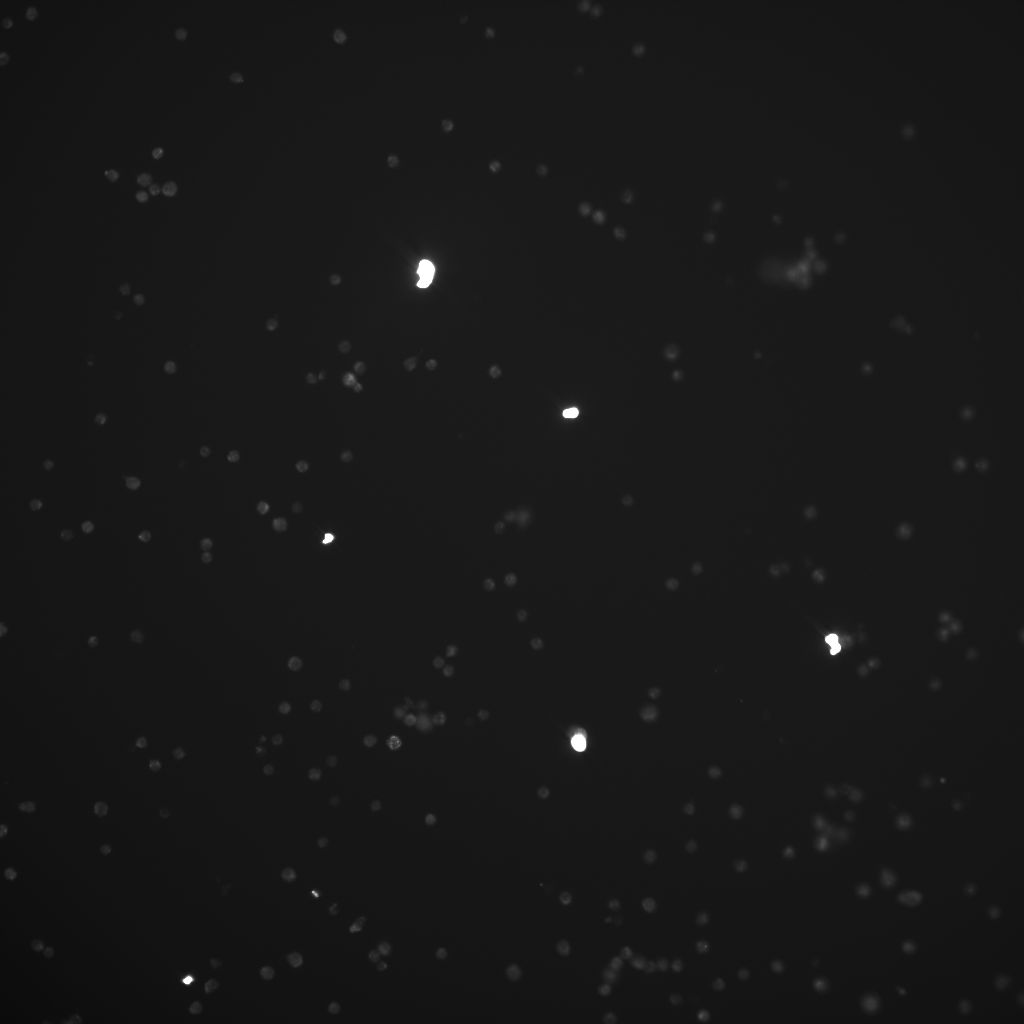

Supplement: Supplementary file 18 [file msb0011-0783-sd18.zip › Snap-151_c3_ORG.png]

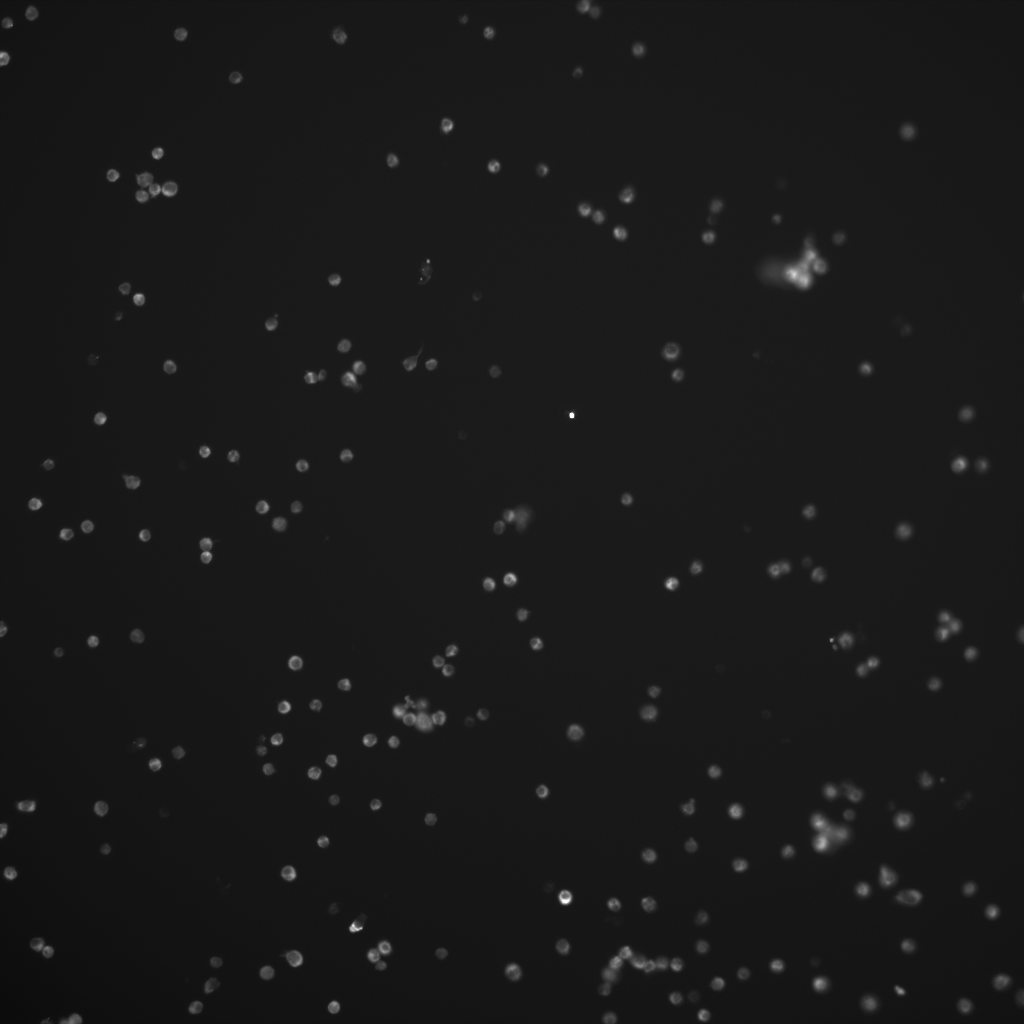

Supplement: Supplementary file 18 [file msb0011-0783-sd18.zip › Snap-151_c4_ORG.png]

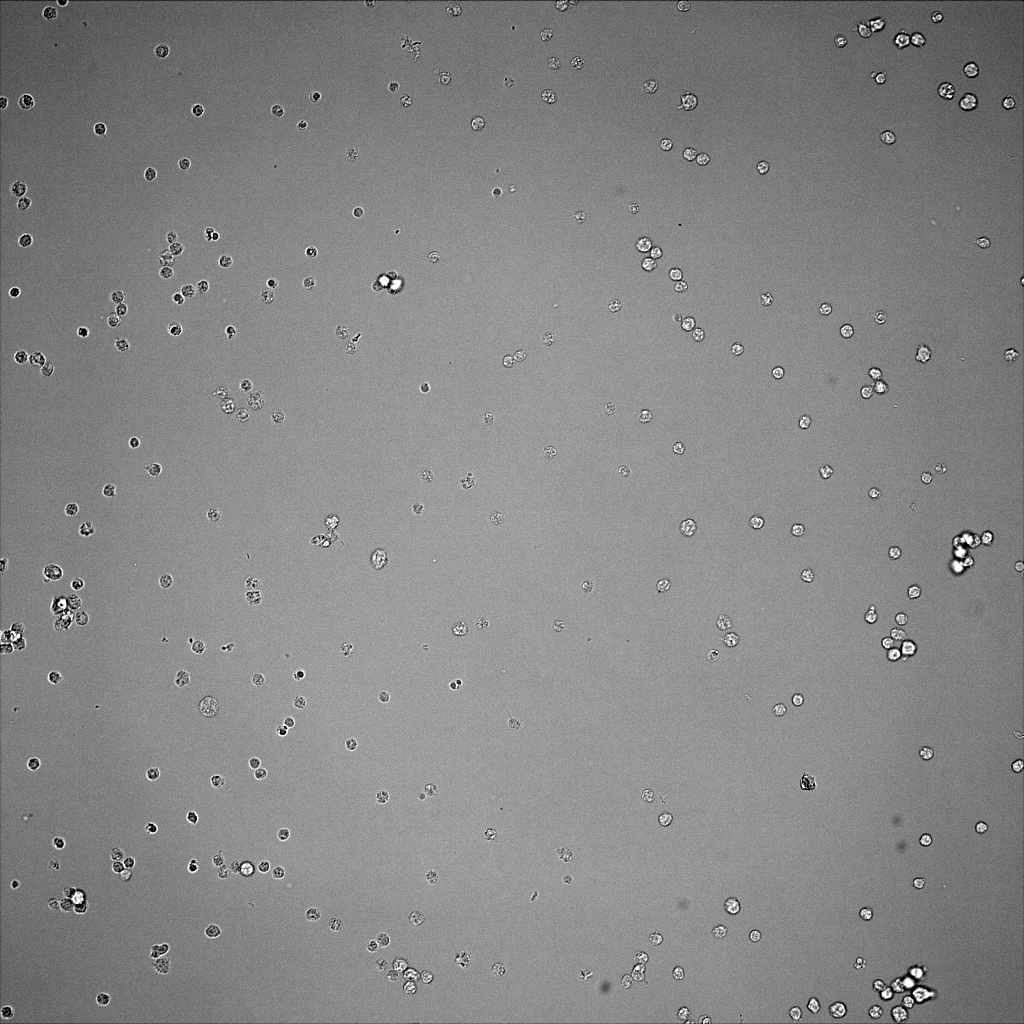

Supplement: Supplementary file 18 [file msb0011-0783-sd18.zip › Snap-152_c1_ORG.png]

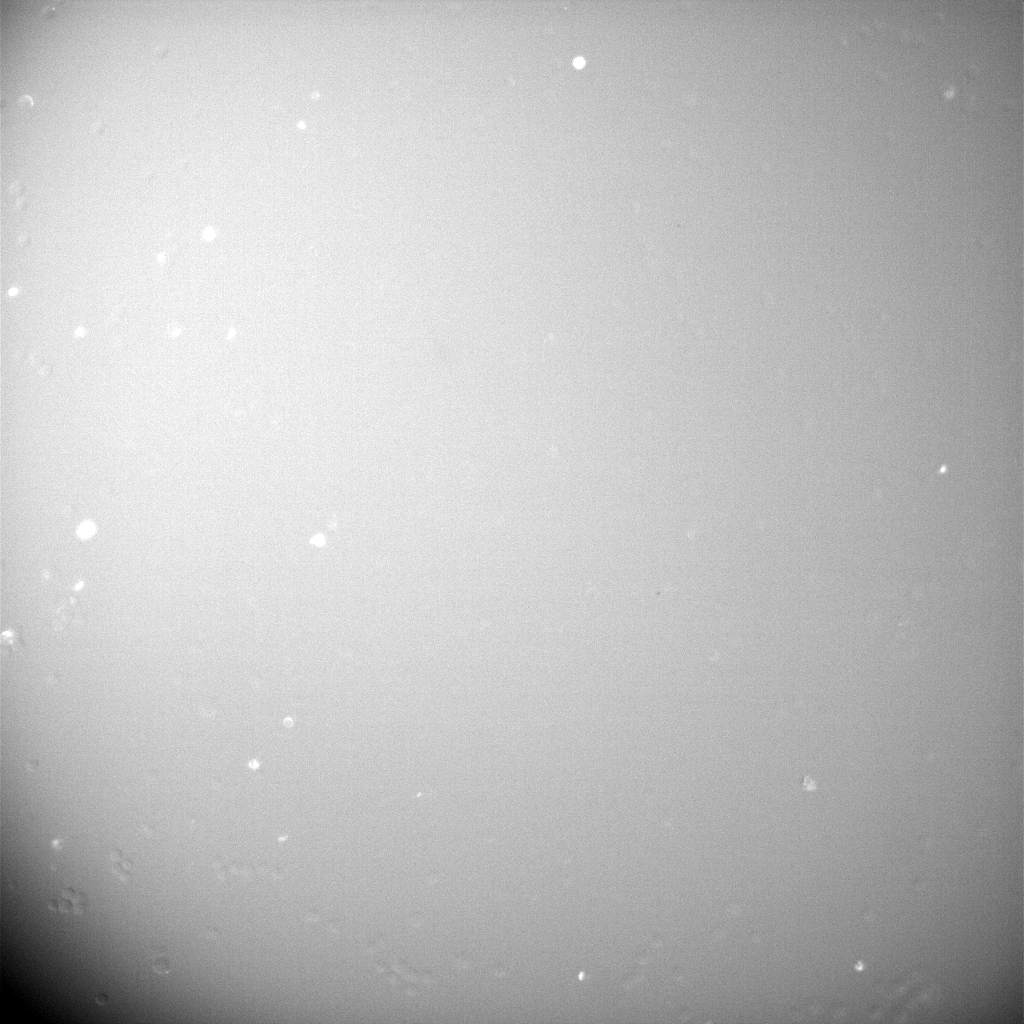

Supplement: Supplementary file 18 [file msb0011-0783-sd18.zip › Snap-152_c2_ORG.png]

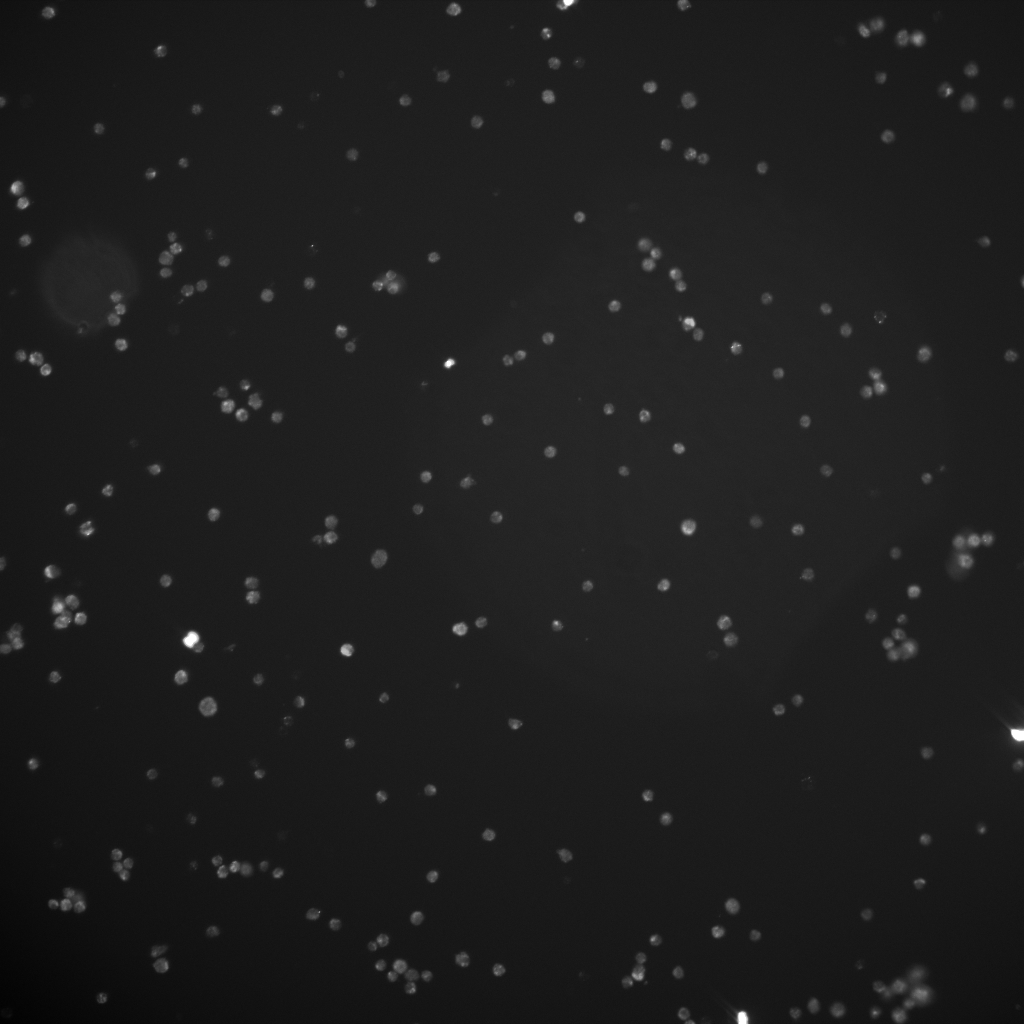

Supplement: Supplementary file 18 [file msb0011-0783-sd18.zip › Snap-152_c3_ORG.png]

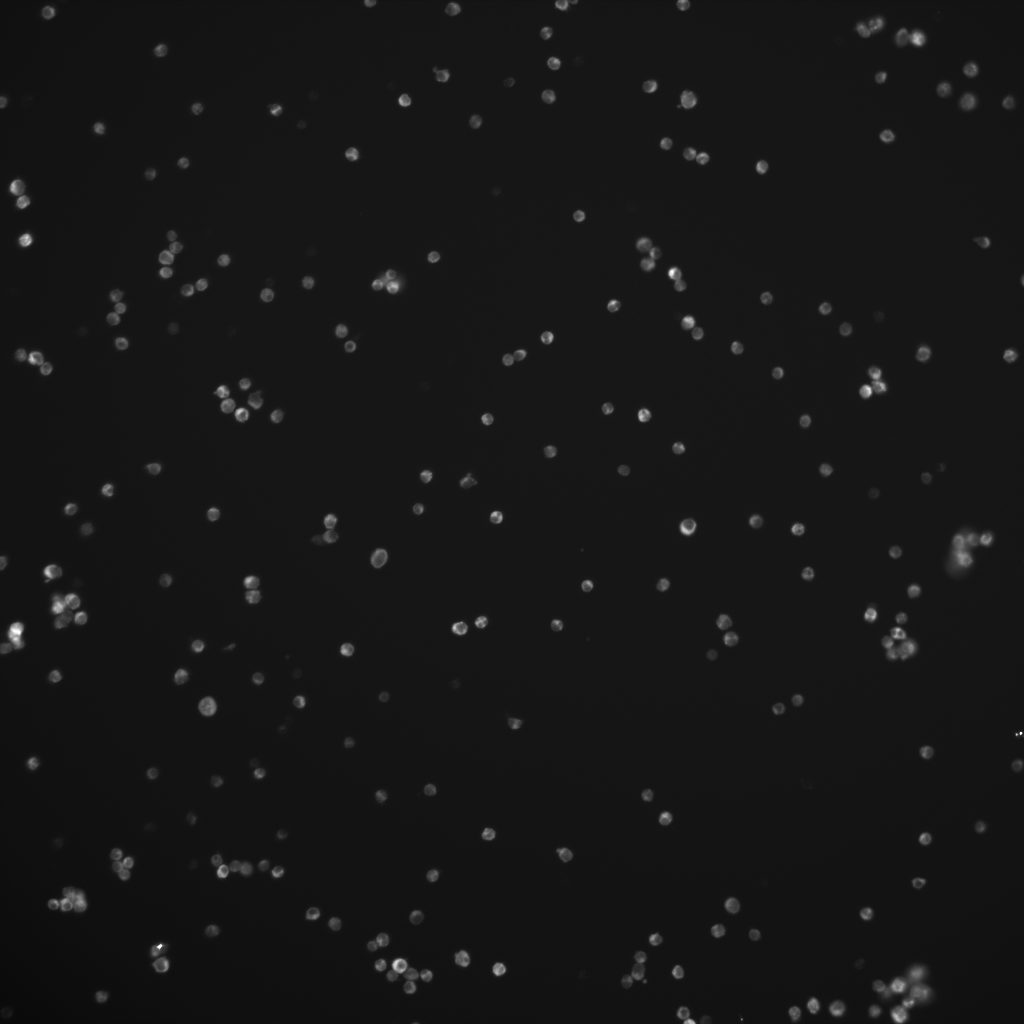

Supplement: Supplementary file 18 [file msb0011-0783-sd18.zip › Snap-152_c4_ORG.png]

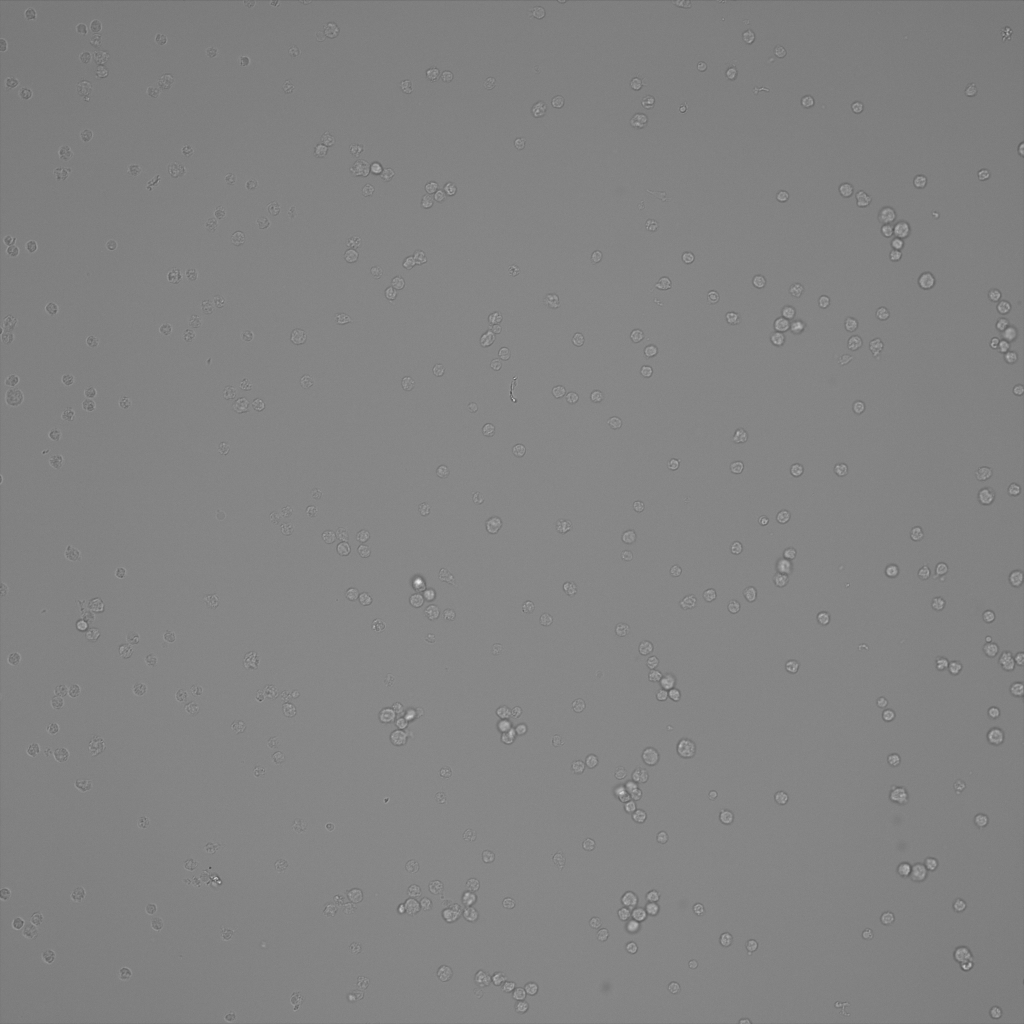

Supplement: Supplementary file 18 [file msb0011-0783-sd18.zip › Snap-153_c1_ORG.png]

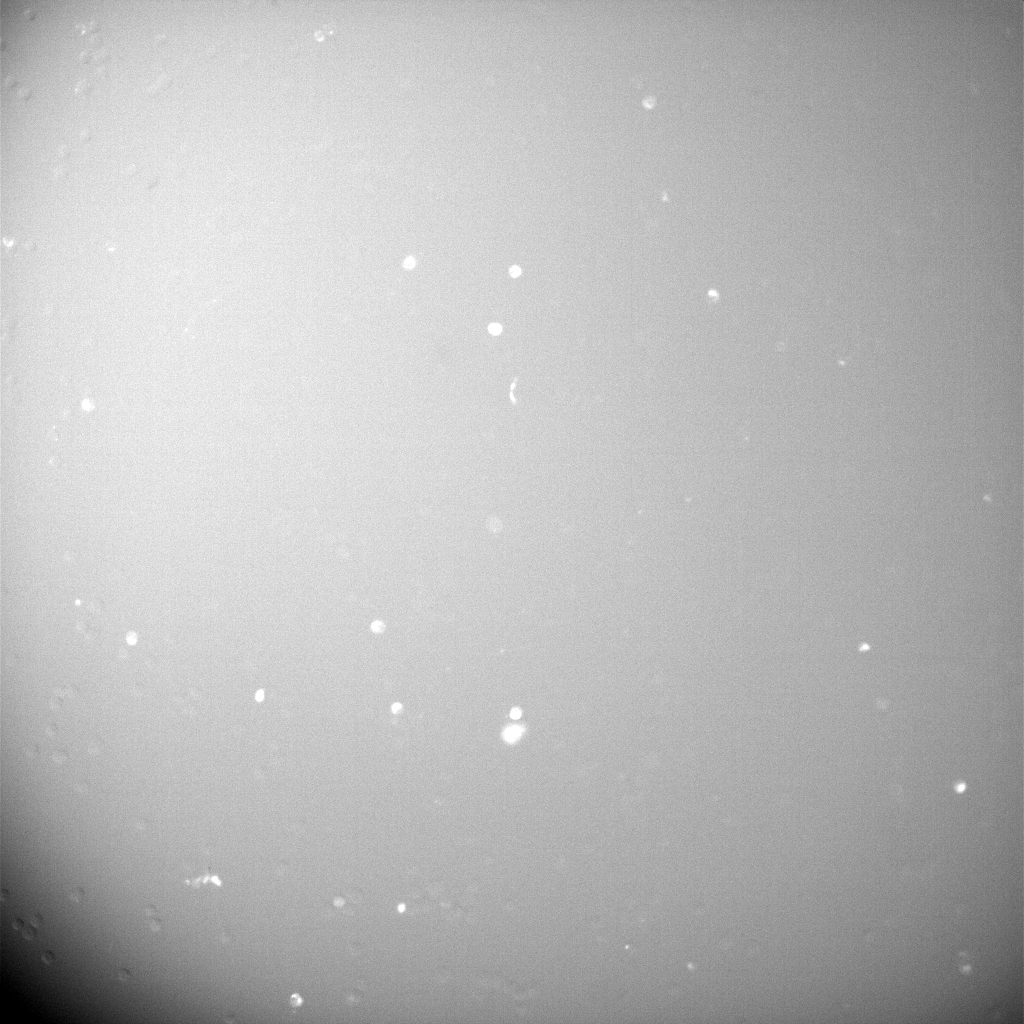

Supplement: Supplementary file 18 [file msb0011-0783-sd18.zip › Snap-153_c2_ORG.png]

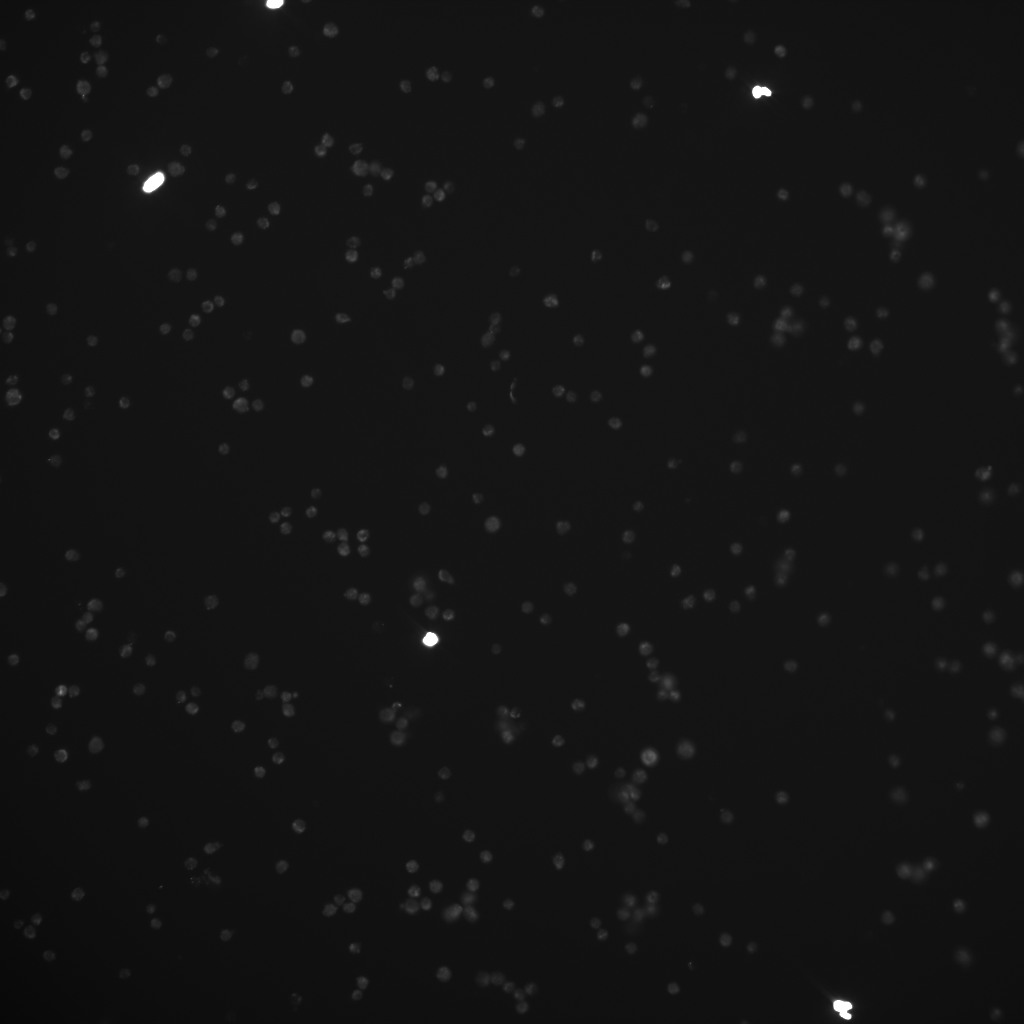

Supplement: Supplementary file 18 [file msb0011-0783-sd18.zip › Snap-153_c3_ORG.png]

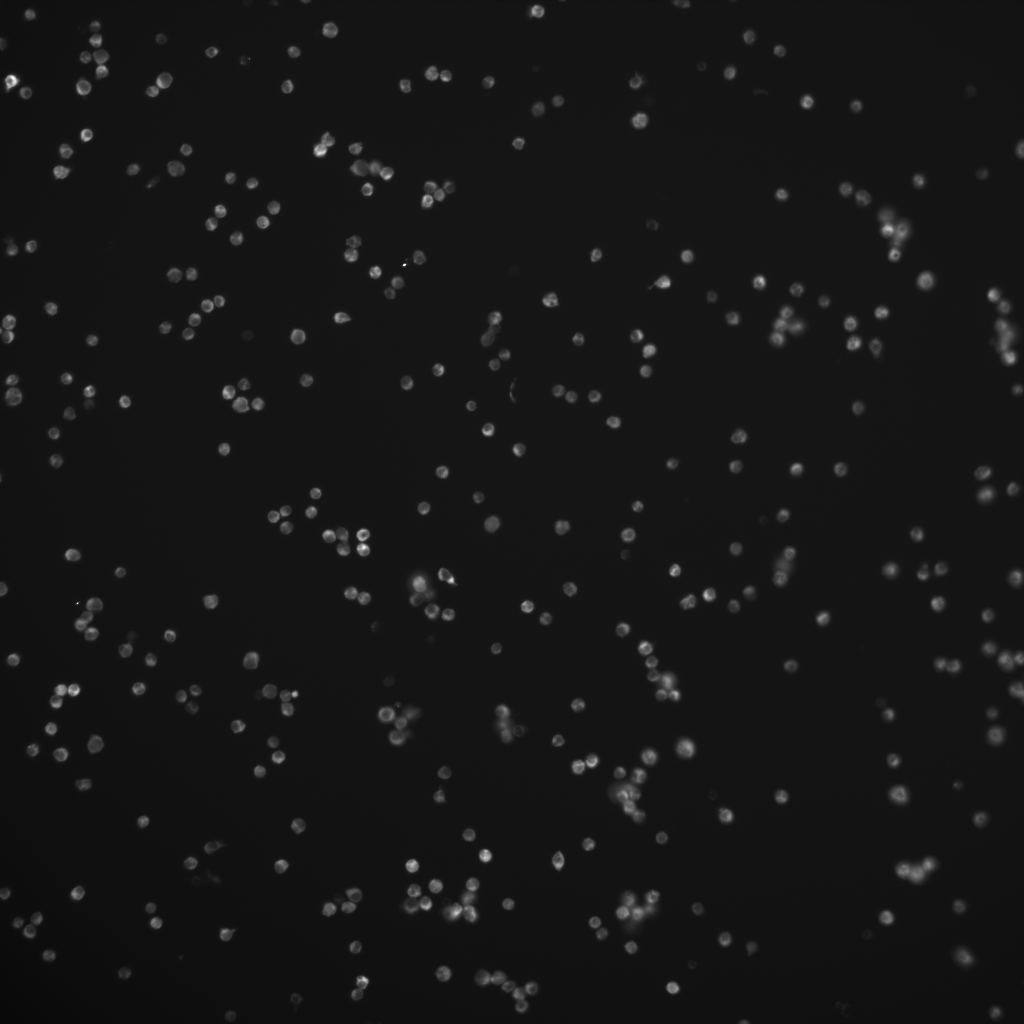

Supplement: Supplementary file 18 [file msb0011-0783-sd18.zip › Snap-153_c4_ORG.png]

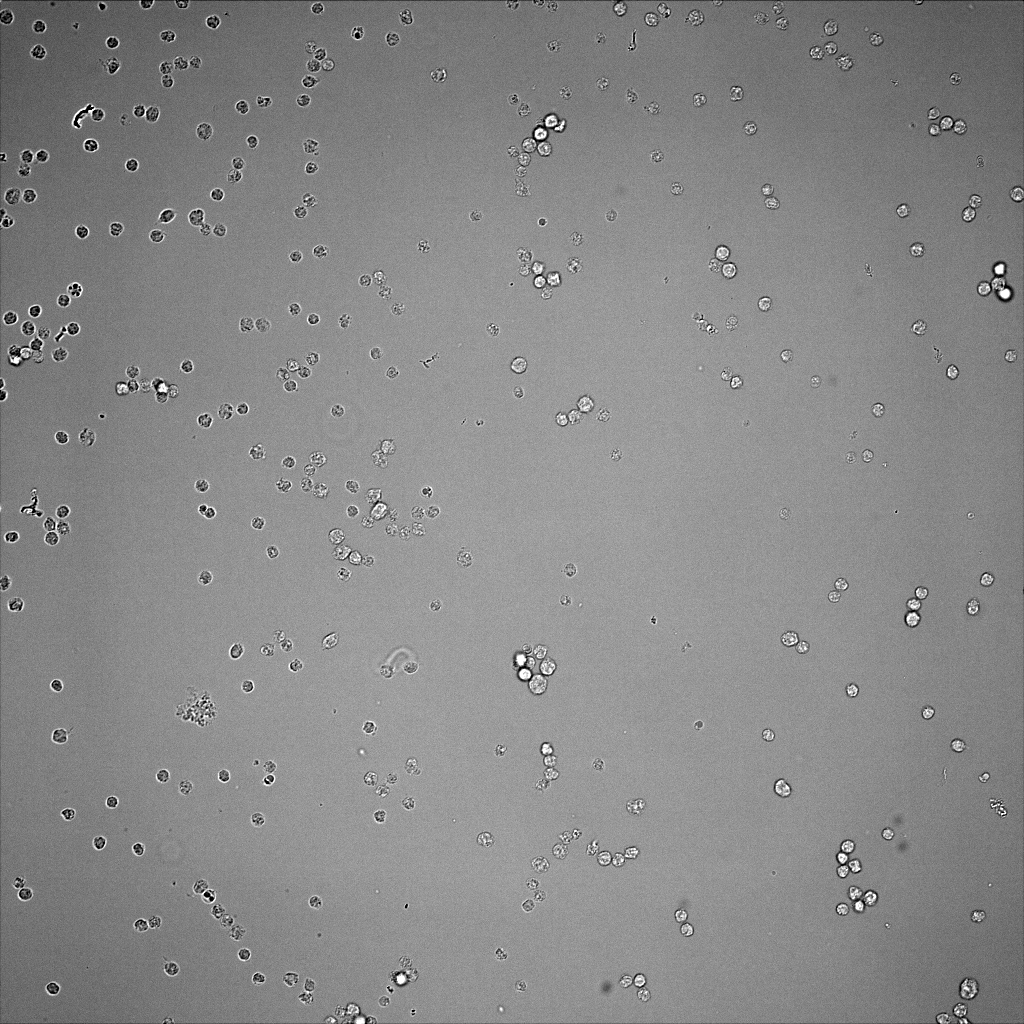

Supplement: Supplementary file 18 [file msb0011-0783-sd18.zip › Snap-154_c1_ORG.png]

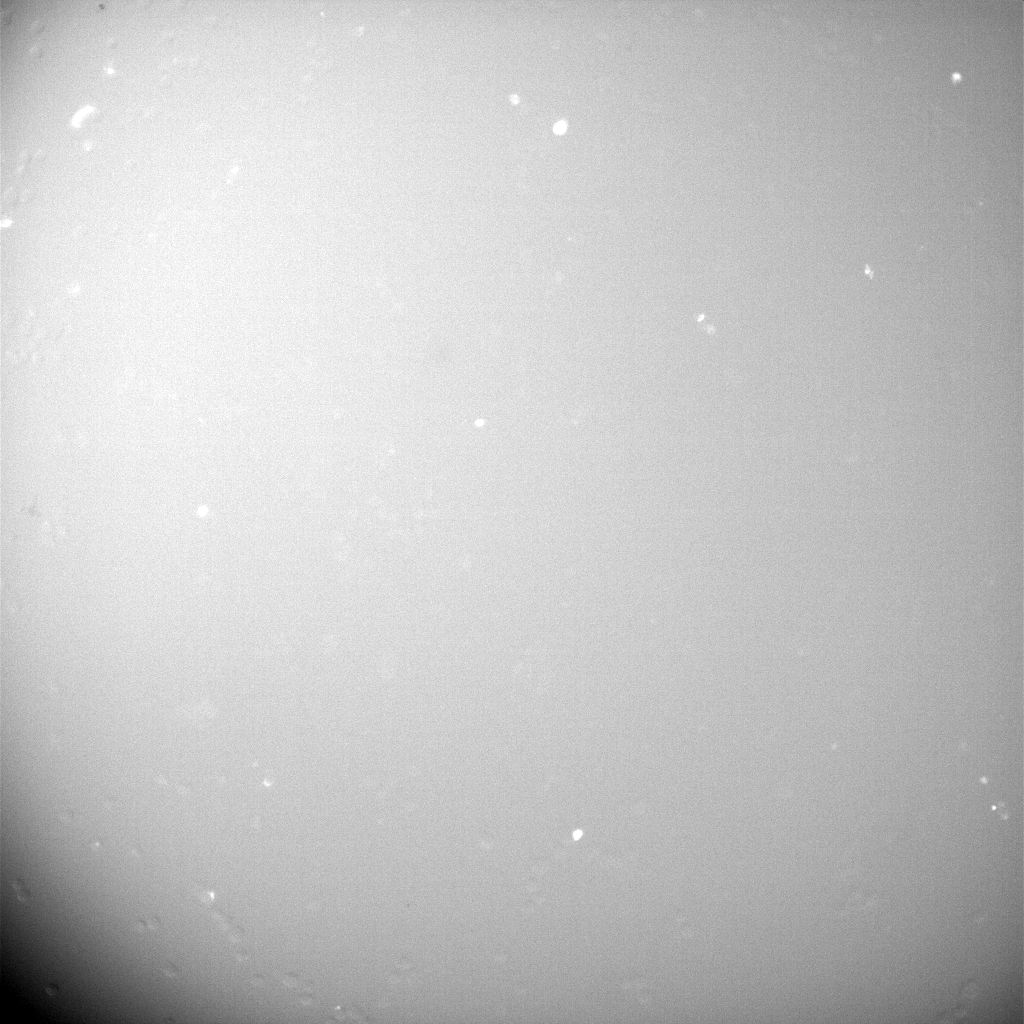

Supplement: Supplementary file 18 [file msb0011-0783-sd18.zip › Snap-154_c2_ORG.png]

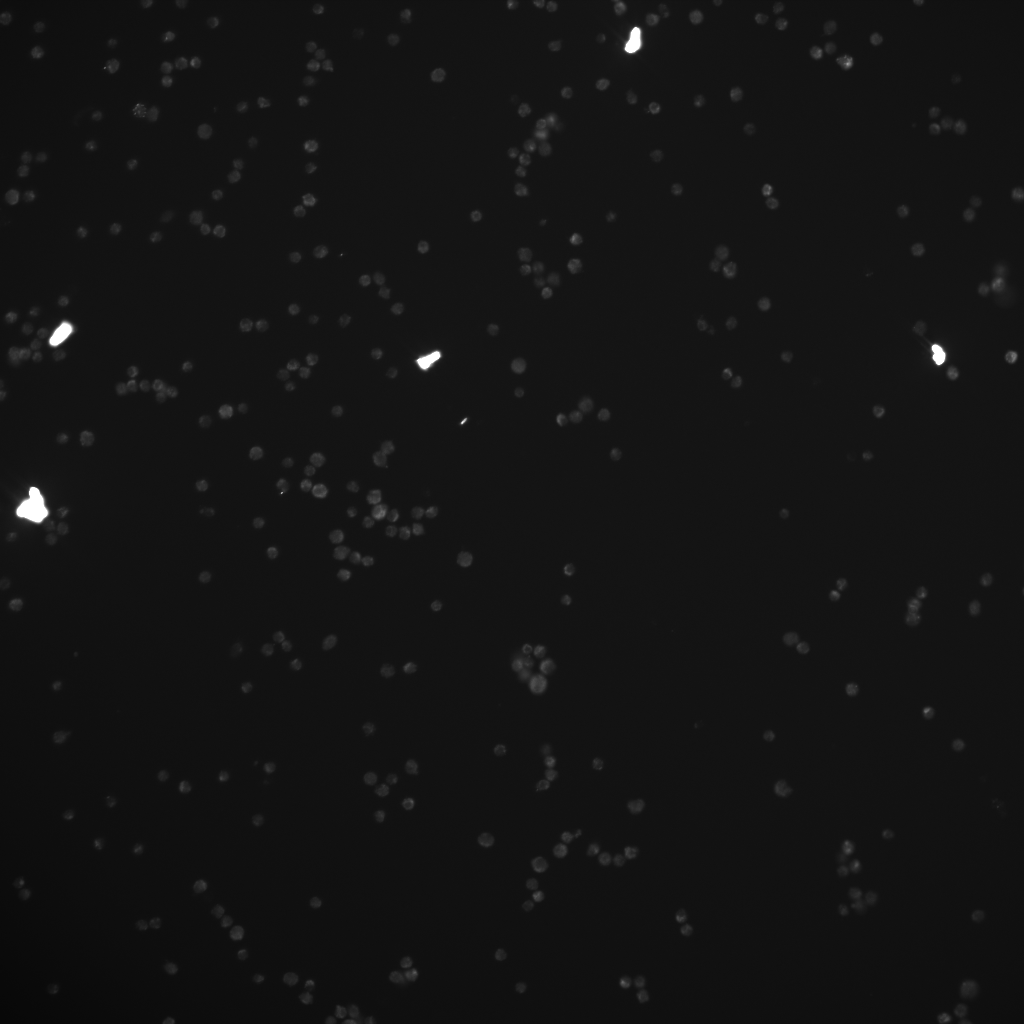

Supplement: Supplementary file 18 [file msb0011-0783-sd18.zip › Snap-154_c3_ORG.png]

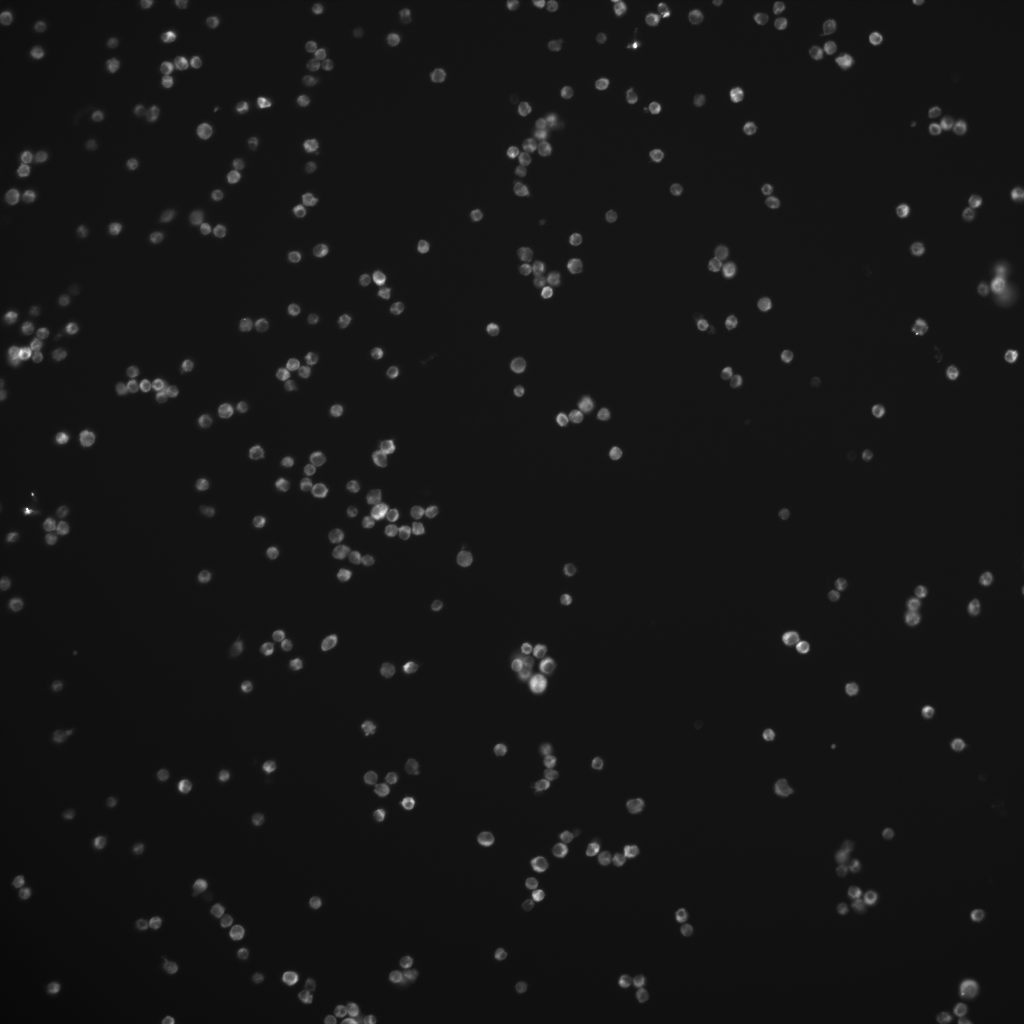

Supplement: Supplementary file 18 [file msb0011-0783-sd18.zip › Snap-154_c4_ORG.png]

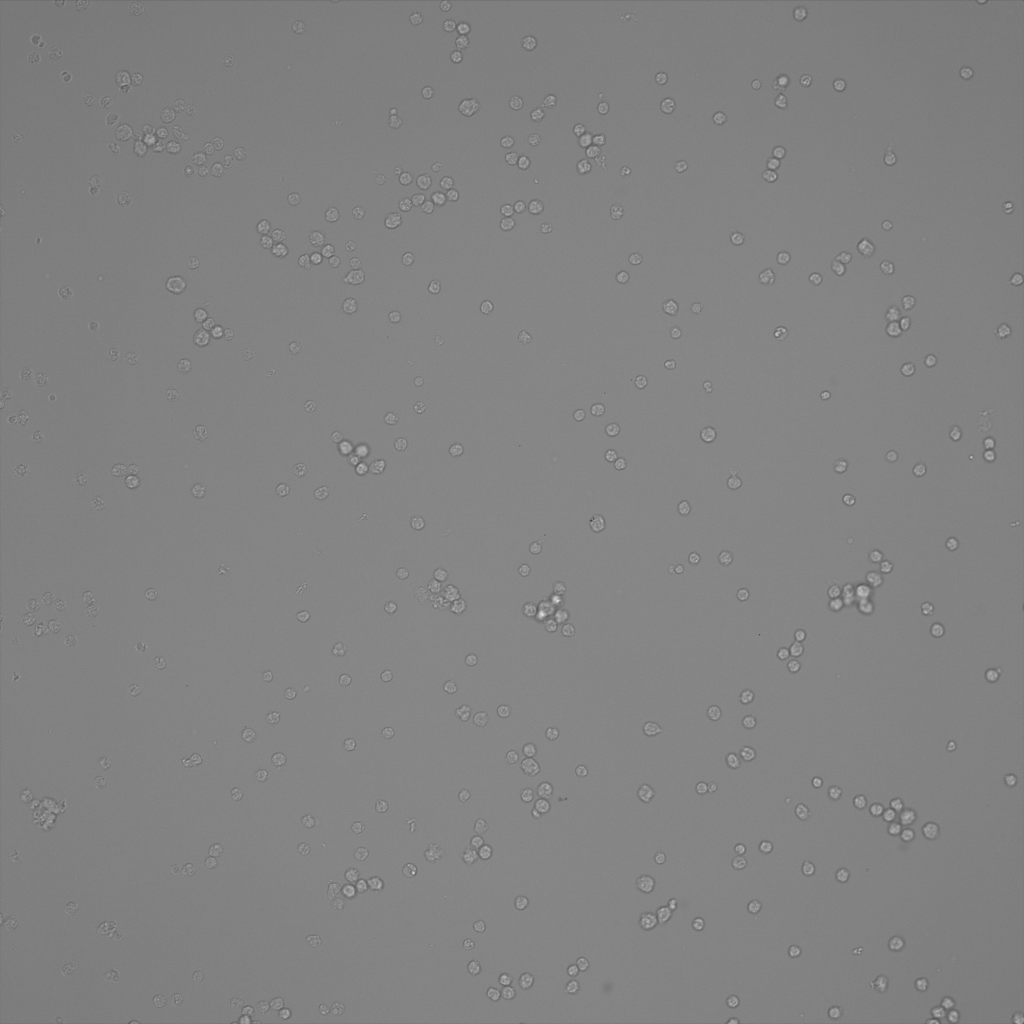

Supplement: Supplementary file 18 [file msb0011-0783-sd18.zip › Snap-155_c1_ORG.png]

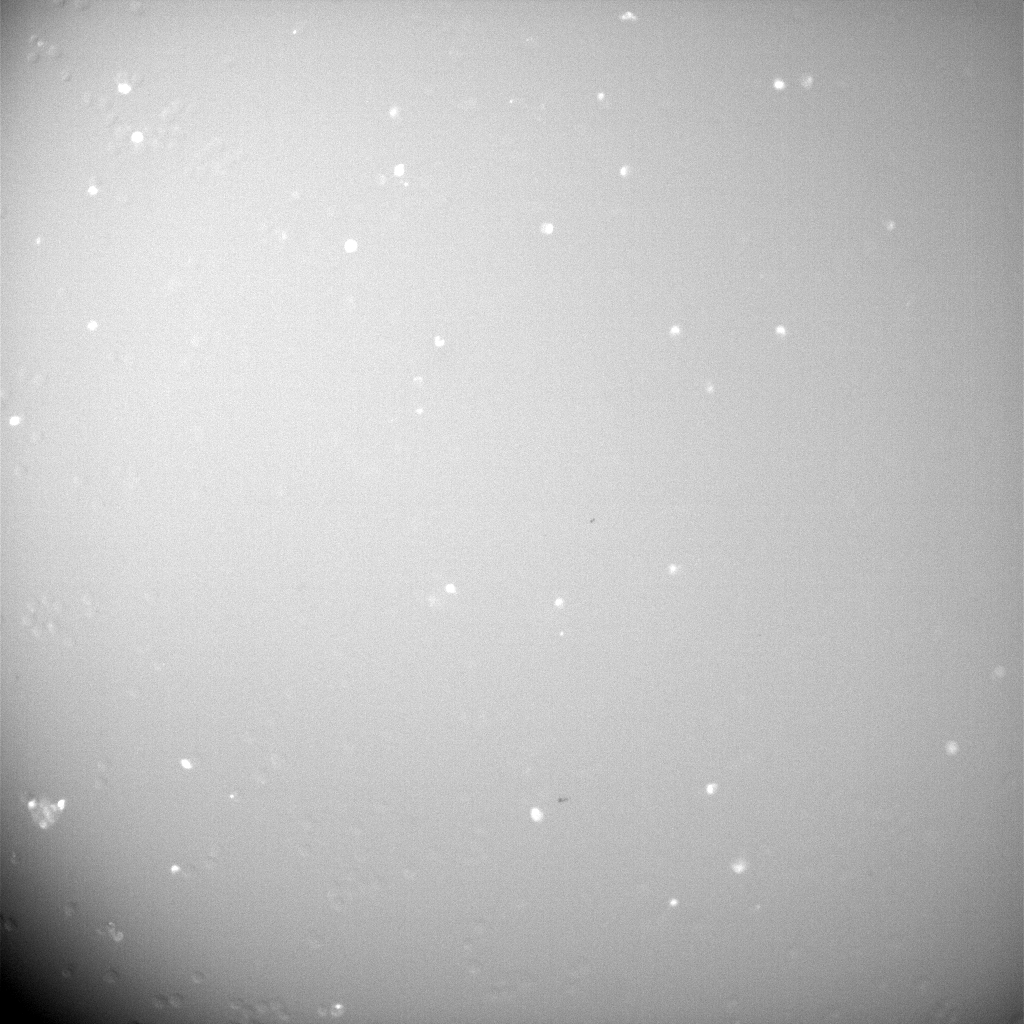

Supplement: Supplementary file 18 [file msb0011-0783-sd18.zip › Snap-155_c2_ORG.png]

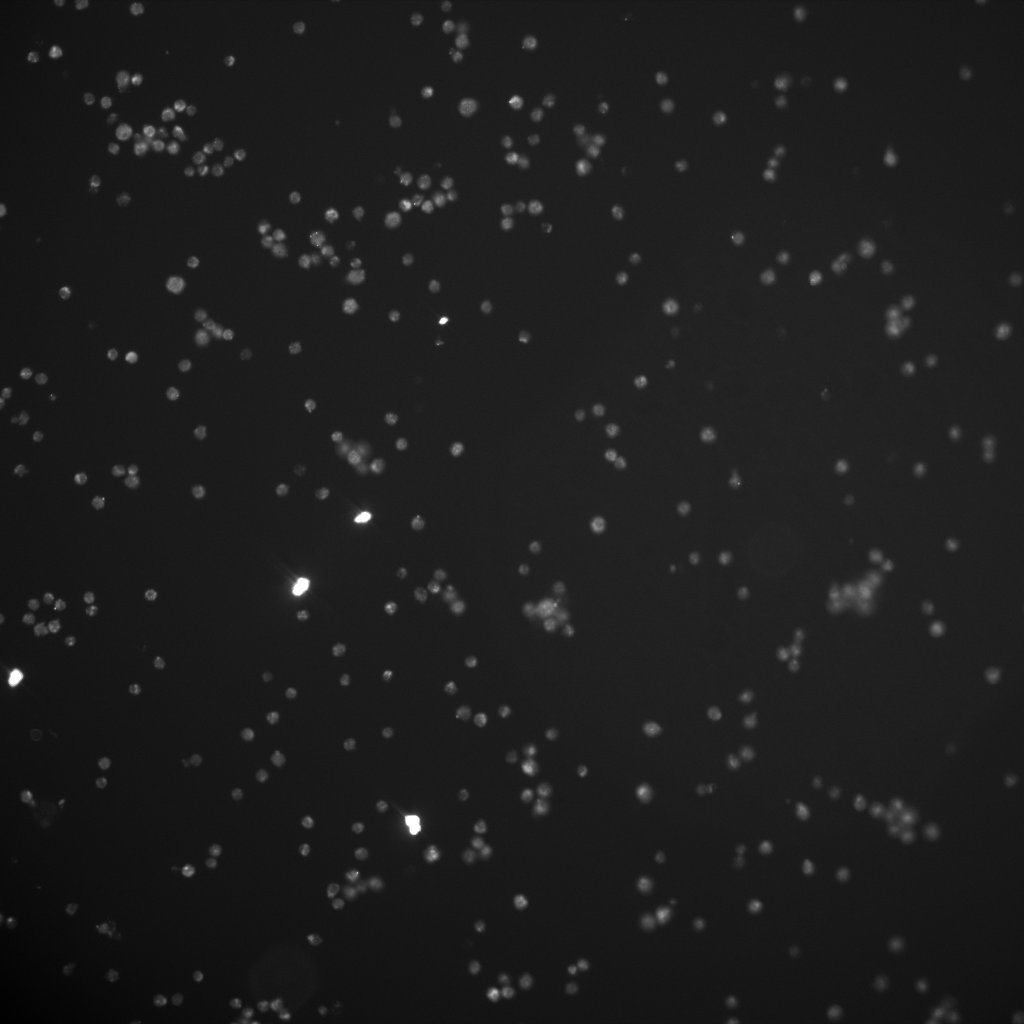

Supplement: Supplementary file 18 [file msb0011-0783-sd18.zip › Snap-155_c3_ORG.png]

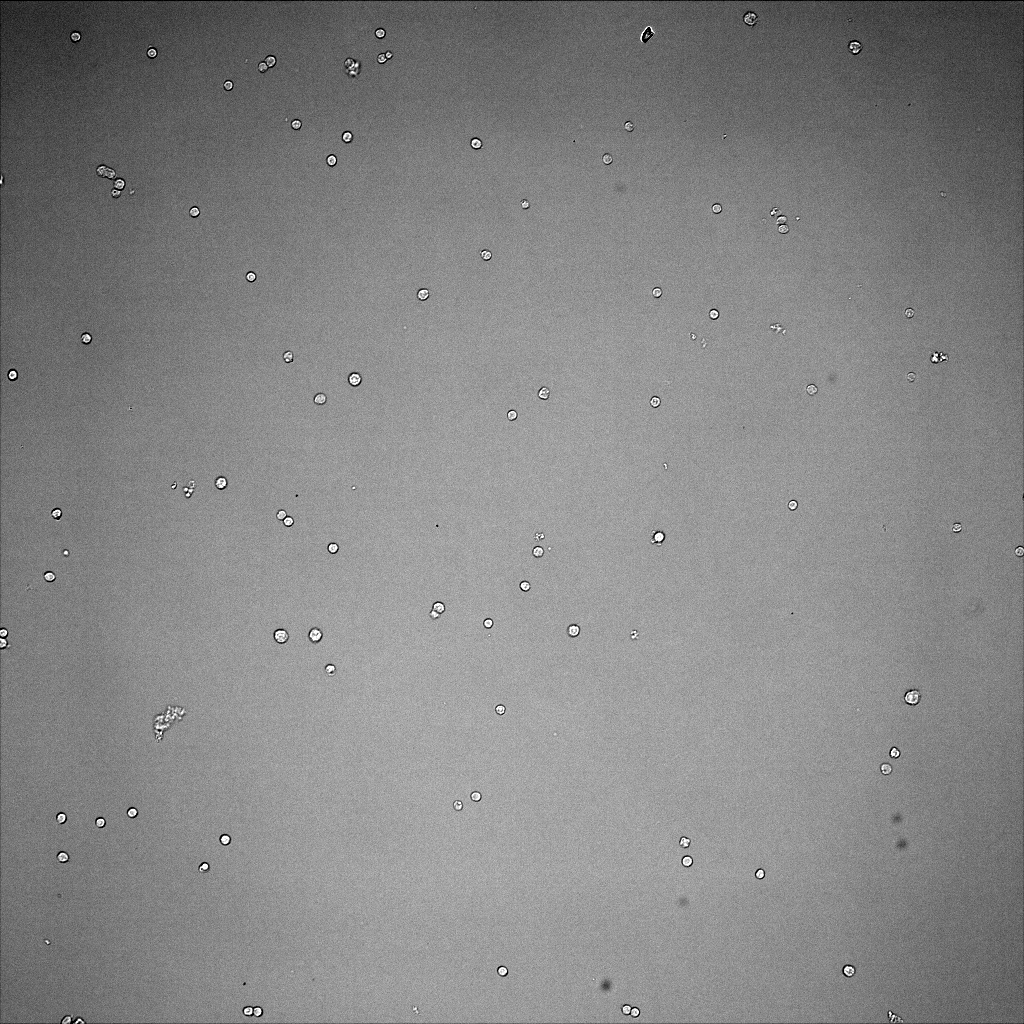

Supplement: Supplementary file 18 [file msb0011-0783-sd18.zip › Snap-165_c1_ORG.png]

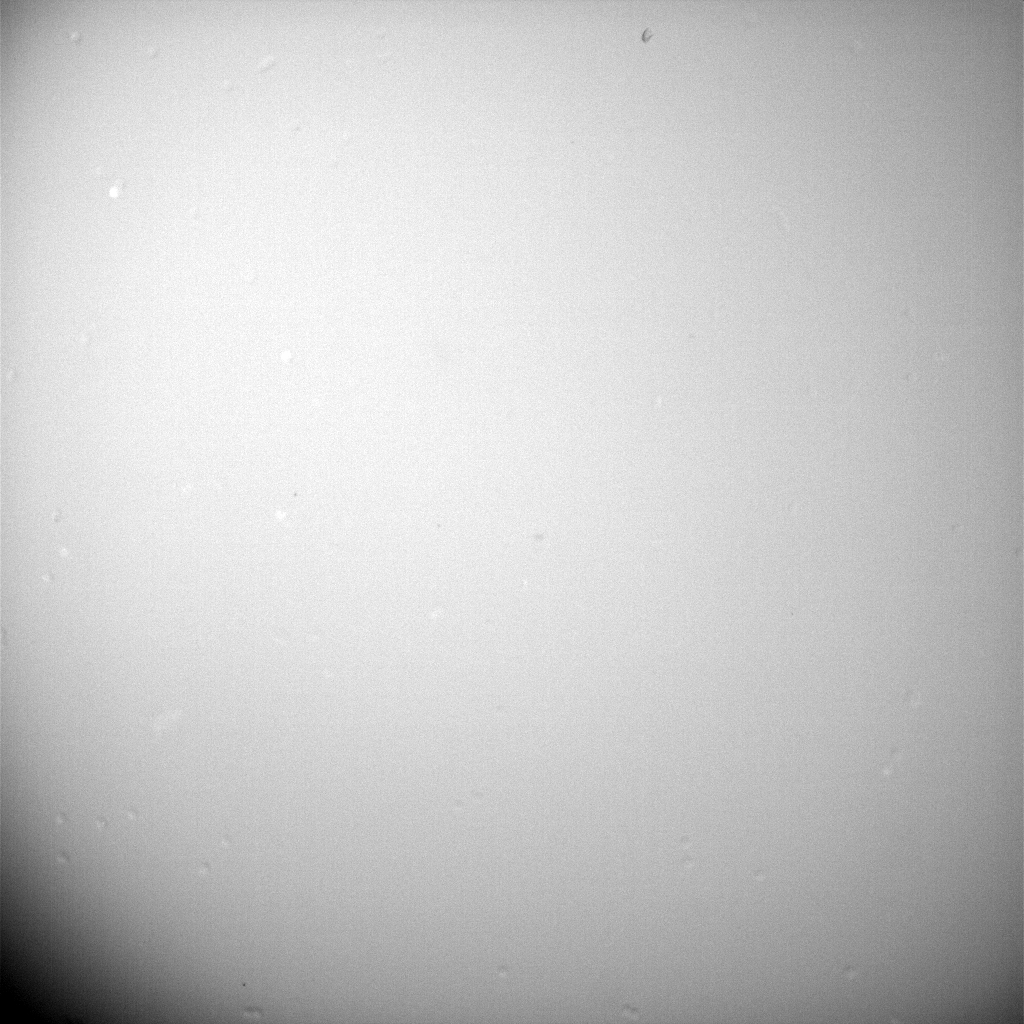

Supplement: Supplementary file 18 [file msb0011-0783-sd18.zip › Snap-165_c2_ORG.png]

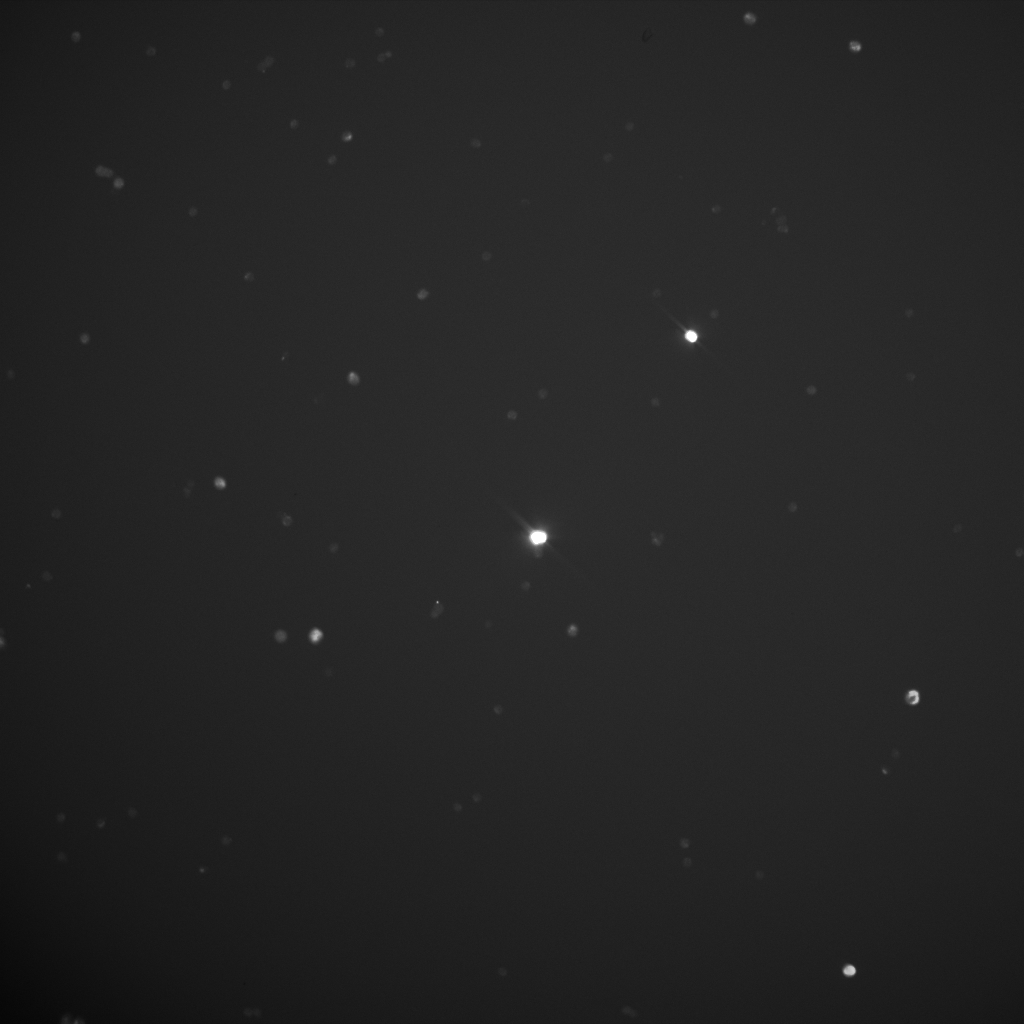

Supplement: Supplementary file 18 [file msb0011-0783-sd18.zip › Snap-165_c3_ORG.png]

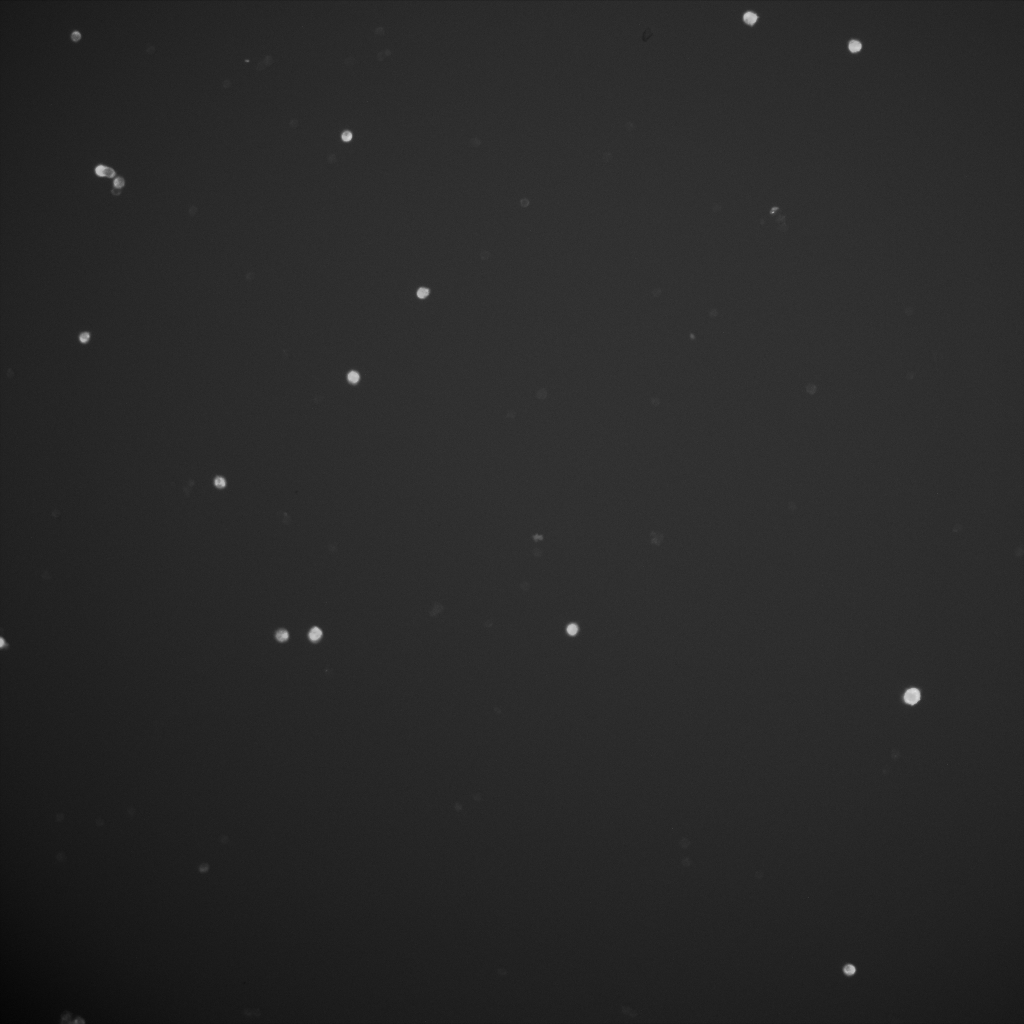

Supplement: Supplementary file 18 [file msb0011-0783-sd18.zip › Snap-165_c4_ORG.png]

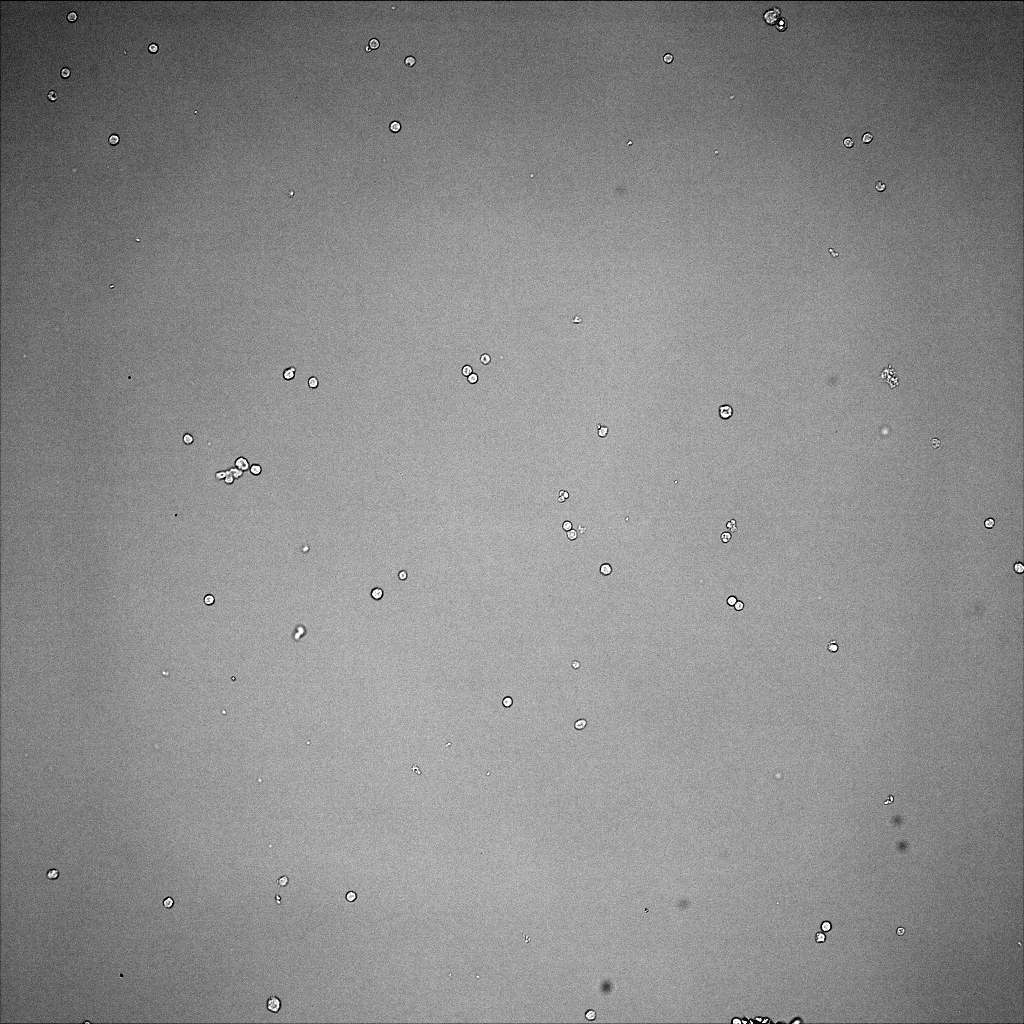

Supplement: Supplementary file 18 [file msb0011-0783-sd18.zip › Snap-166_c1_ORG.png]

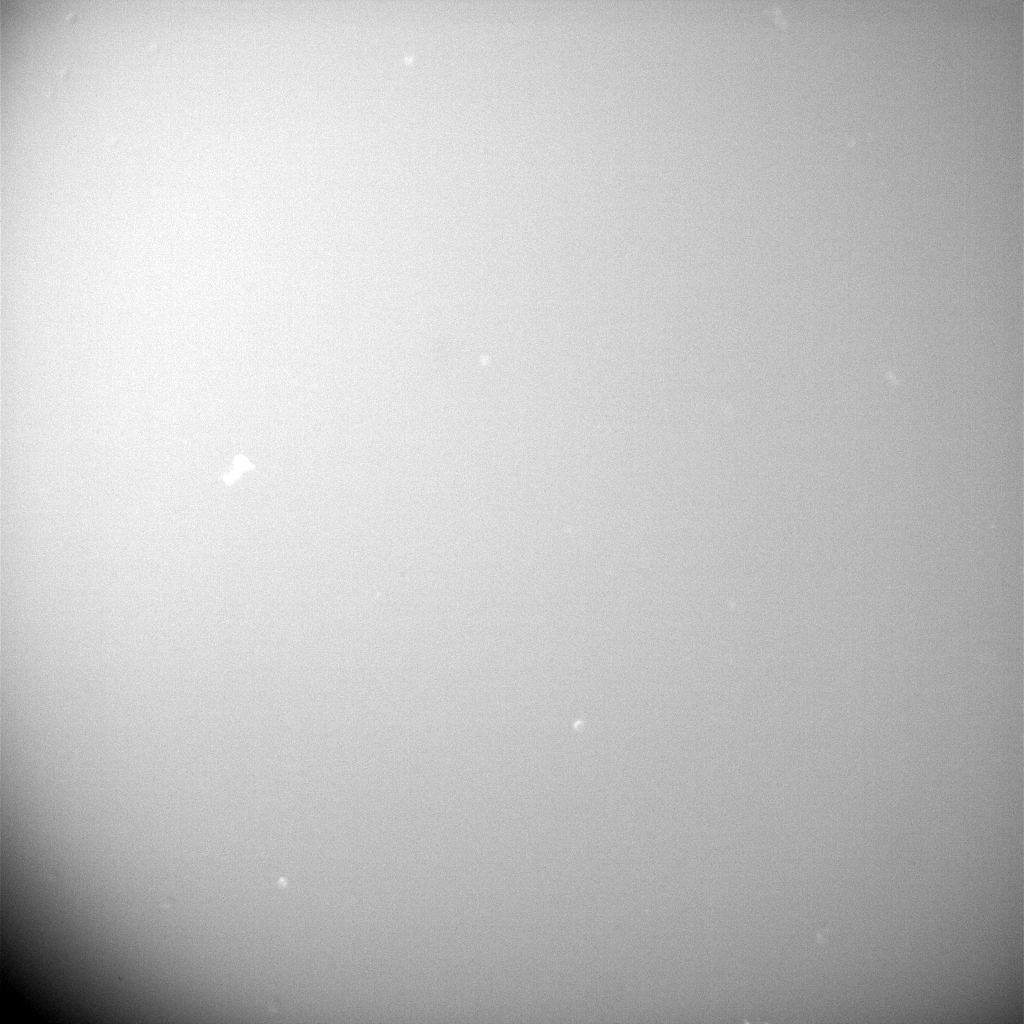

Supplement: Supplementary file 18 [file msb0011-0783-sd18.zip › Snap-166_c2_ORG.png]

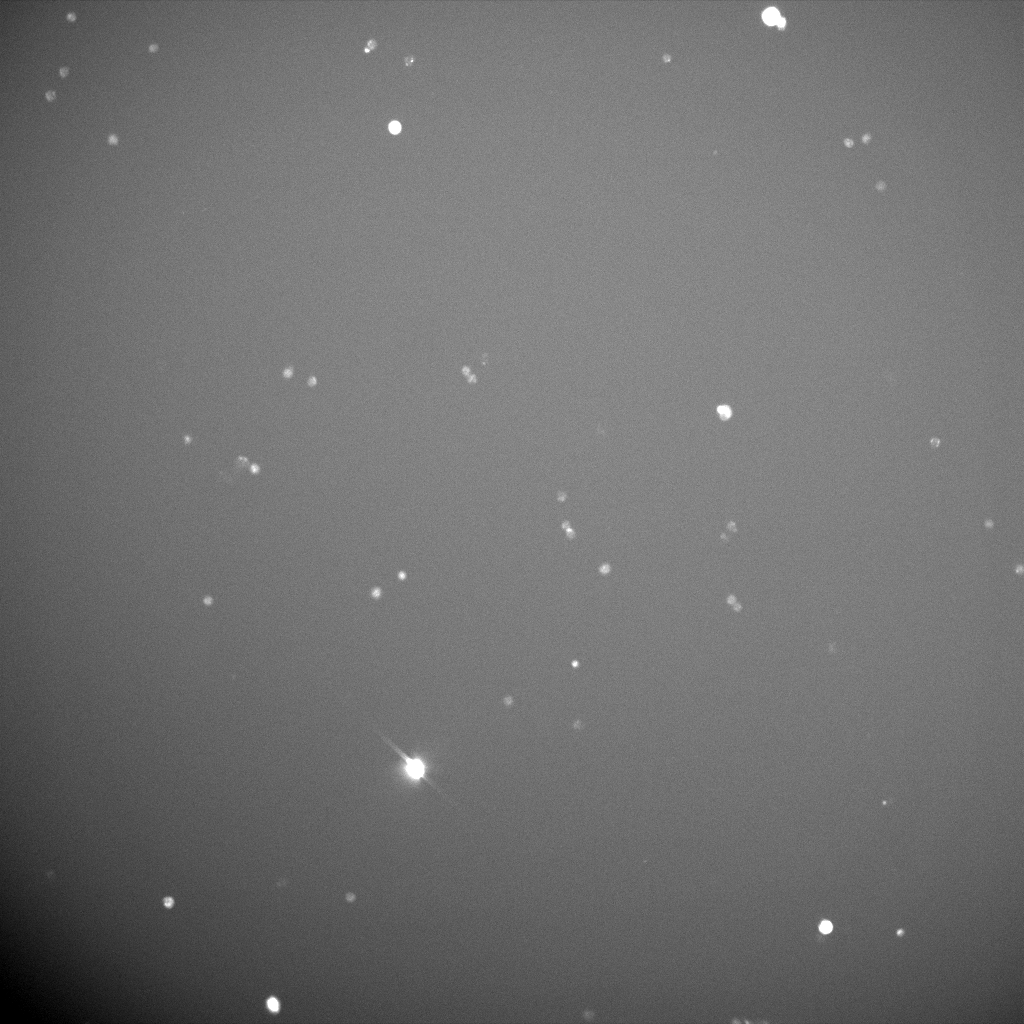

Supplement: Supplementary file 18 [file msb0011-0783-sd18.zip › Snap-166_c3_ORG.png]

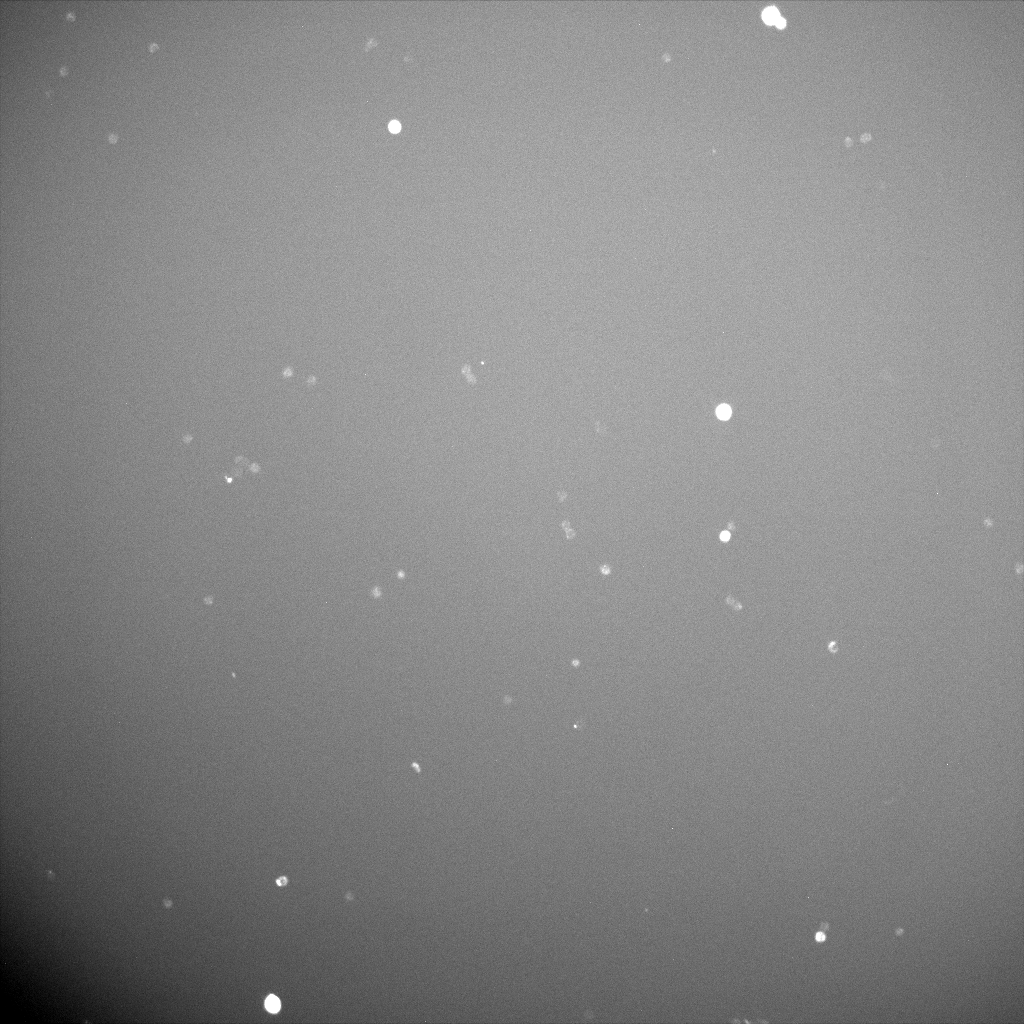

Supplement: Supplementary file 18 [file msb0011-0783-sd18.zip › Snap-166_c4_ORG.png]

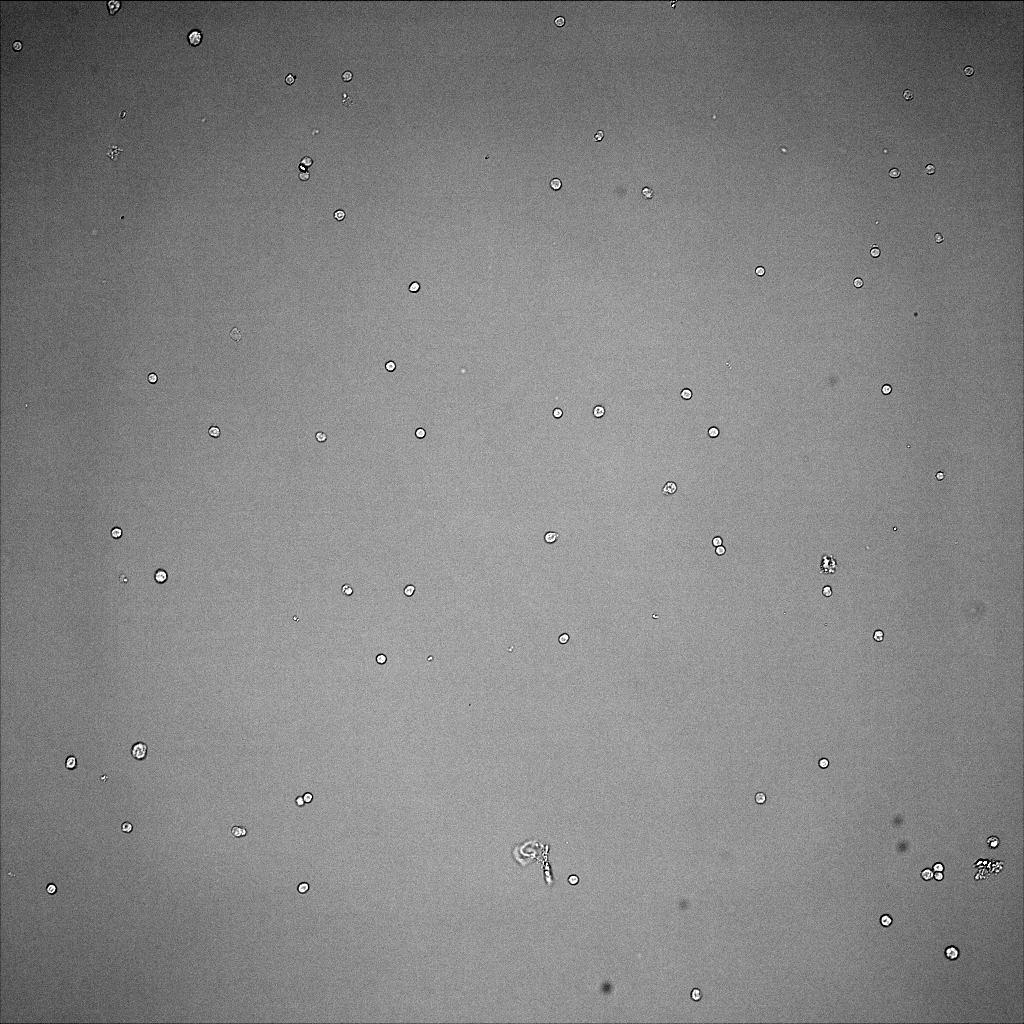

Supplement: Supplementary file 18 [file msb0011-0783-sd18.zip › Snap-167_c1_ORG.png]

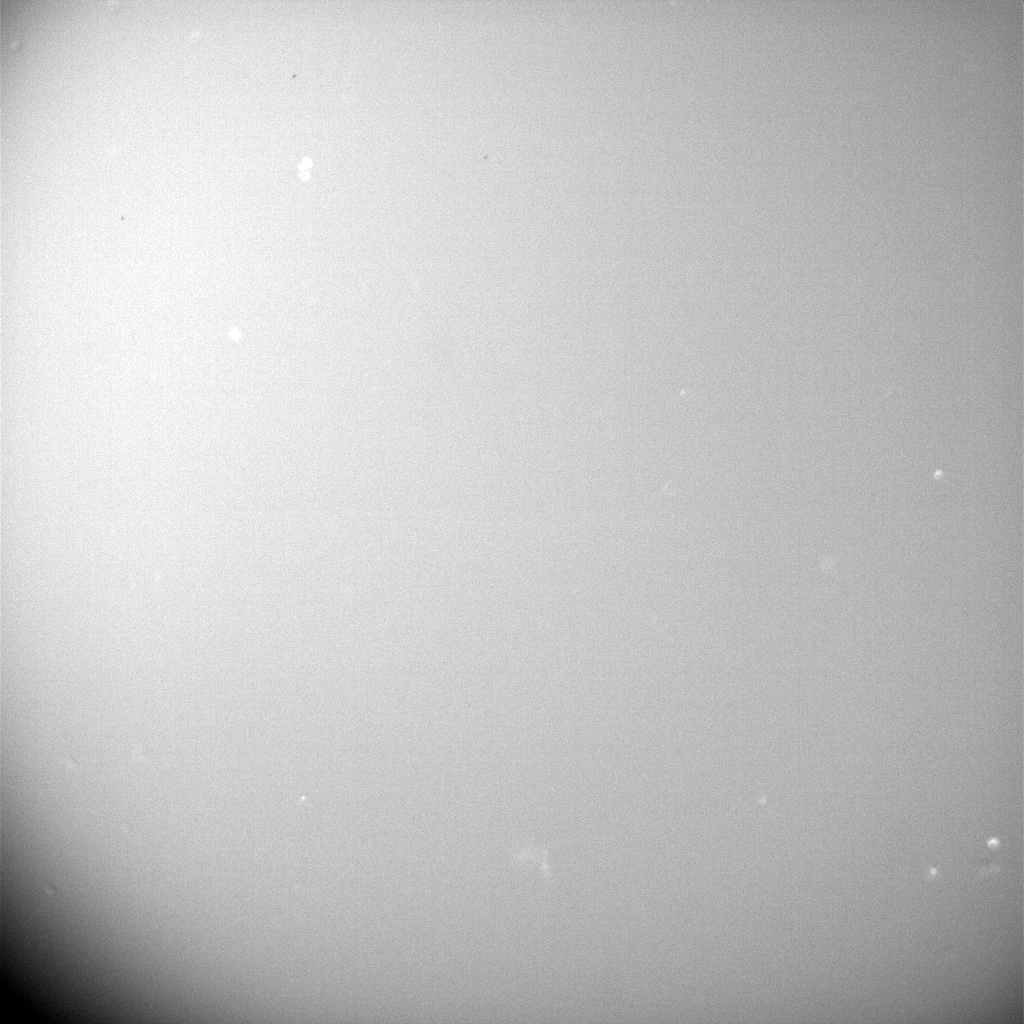

Supplement: Supplementary file 18 [file msb0011-0783-sd18.zip › Snap-167_c2_ORG.png]

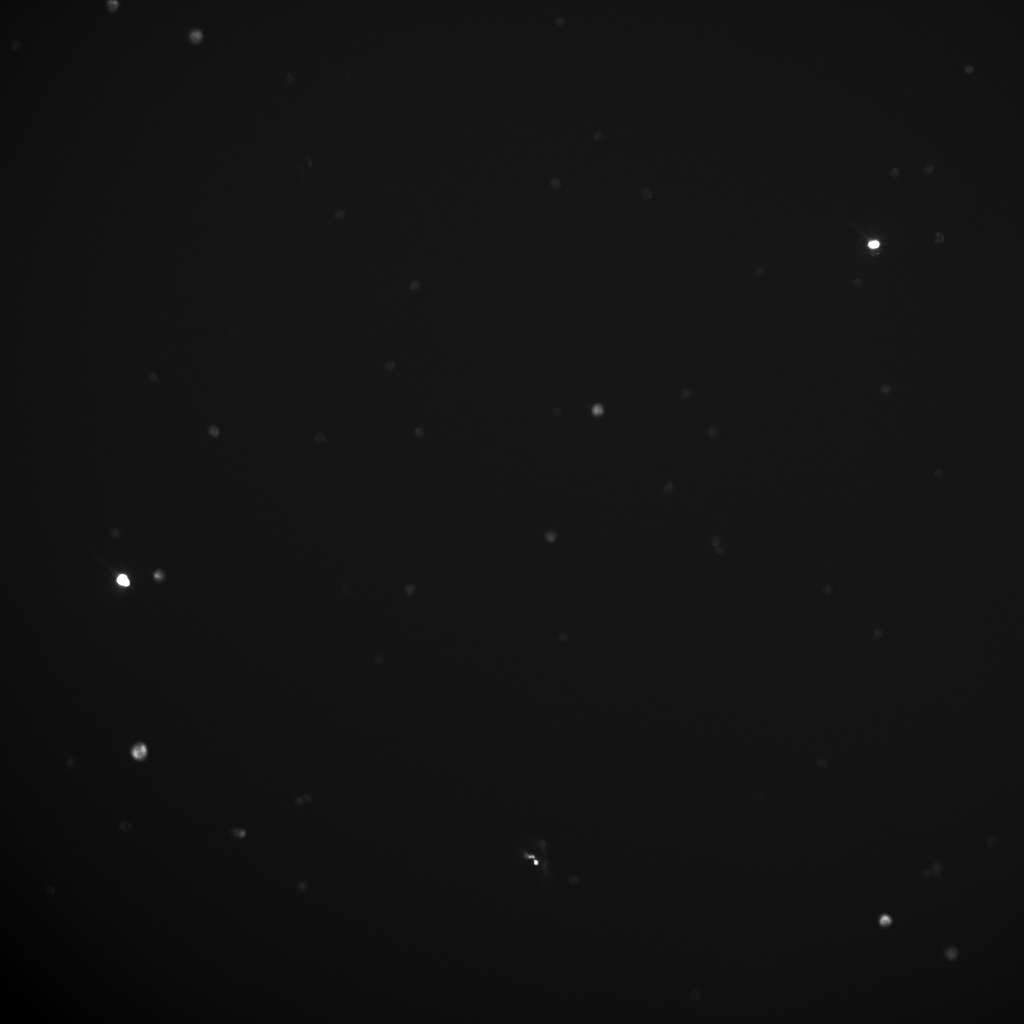

Supplement: Supplementary file 18 [file msb0011-0783-sd18.zip › Snap-167_c3_ORG.png]

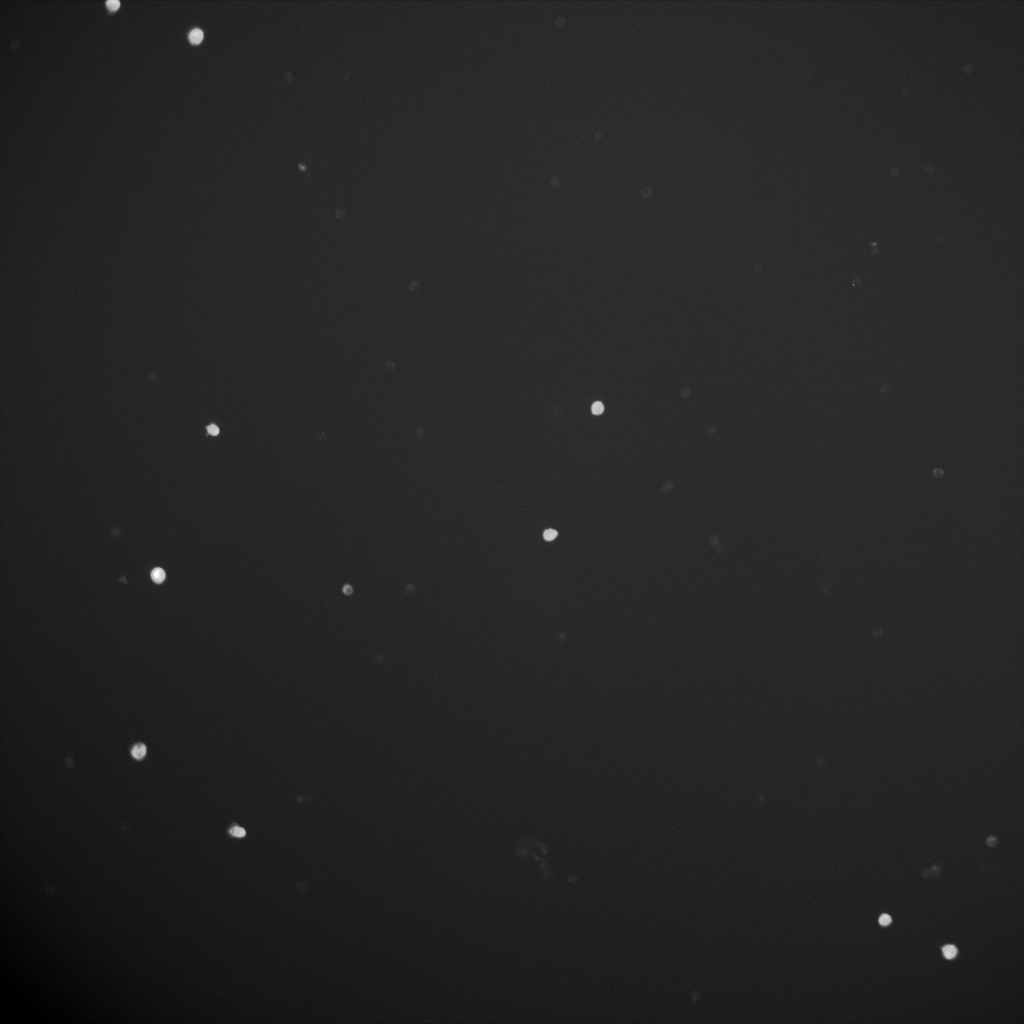

Supplement: Supplementary file 18 [file msb0011-0783-sd18.zip › Snap-167_c4_ORG.png]

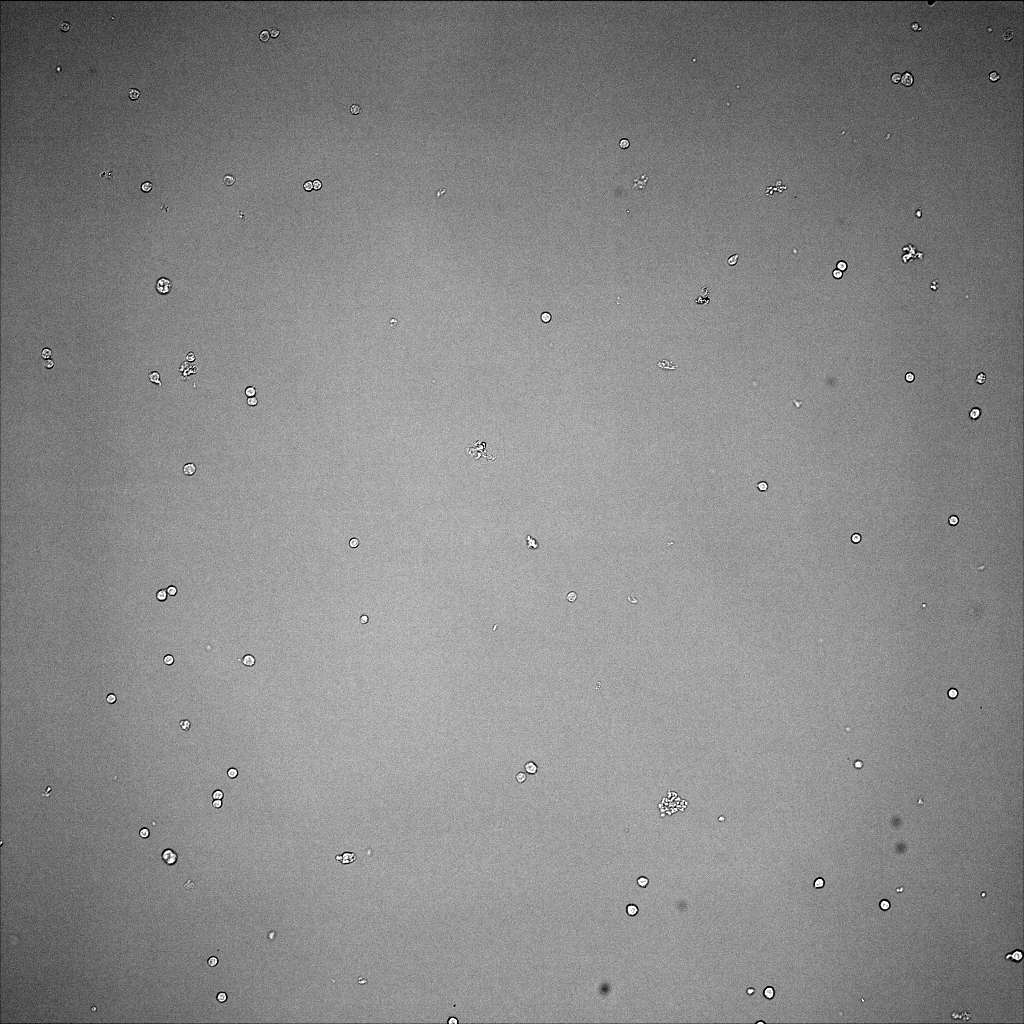

Supplement: Supplementary file 18 [file msb0011-0783-sd18.zip › Snap-168_c1_ORG.png]

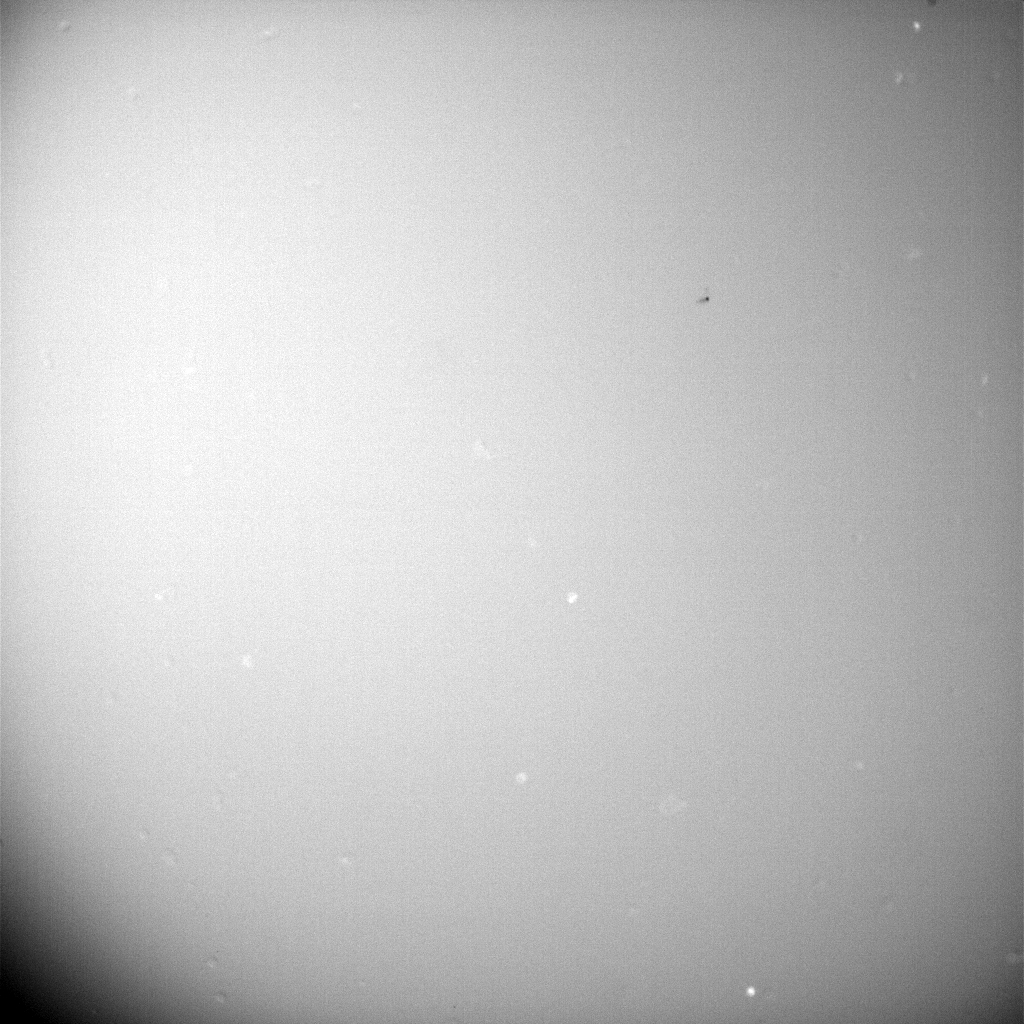

Supplement: Supplementary file 18 [file msb0011-0783-sd18.zip › Snap-168_c2_ORG.png]

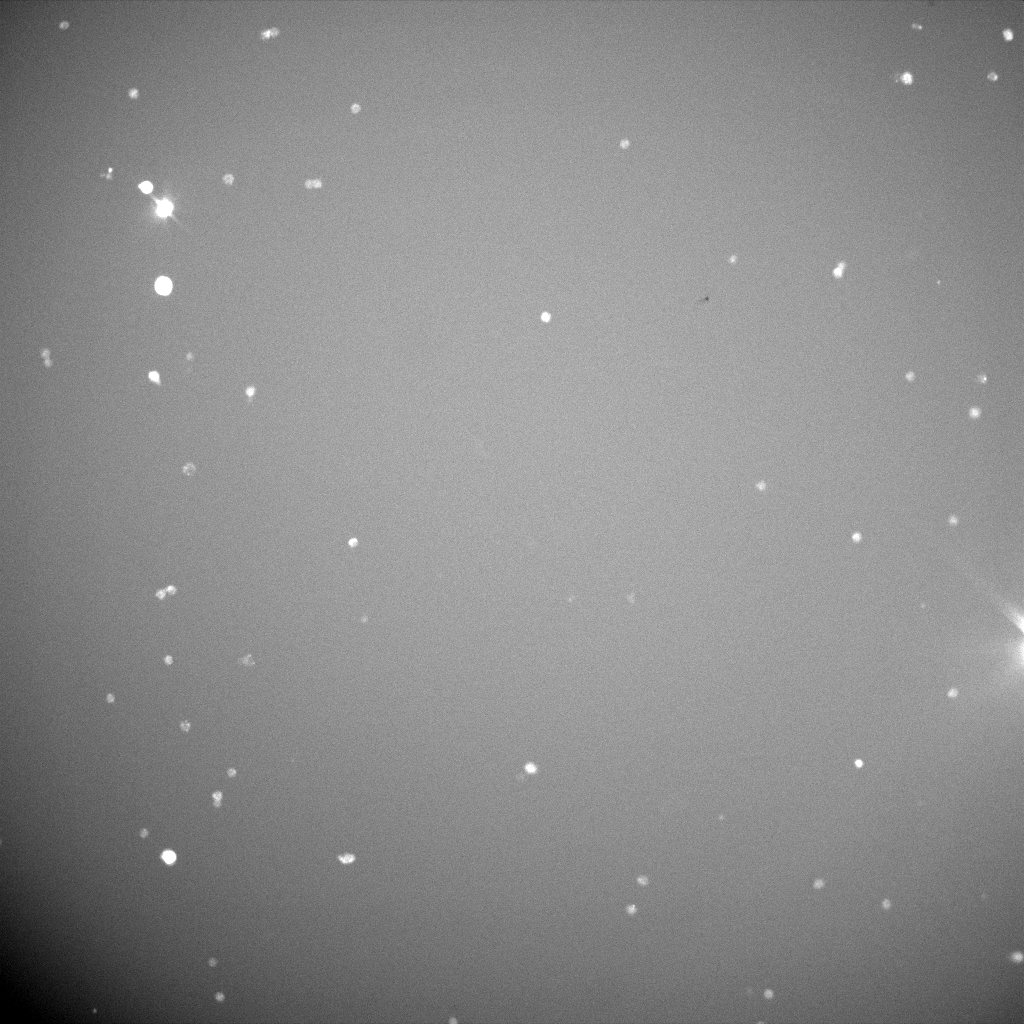

Supplement: Supplementary file 18 [file msb0011-0783-sd18.zip › Snap-168_c3_ORG.png]

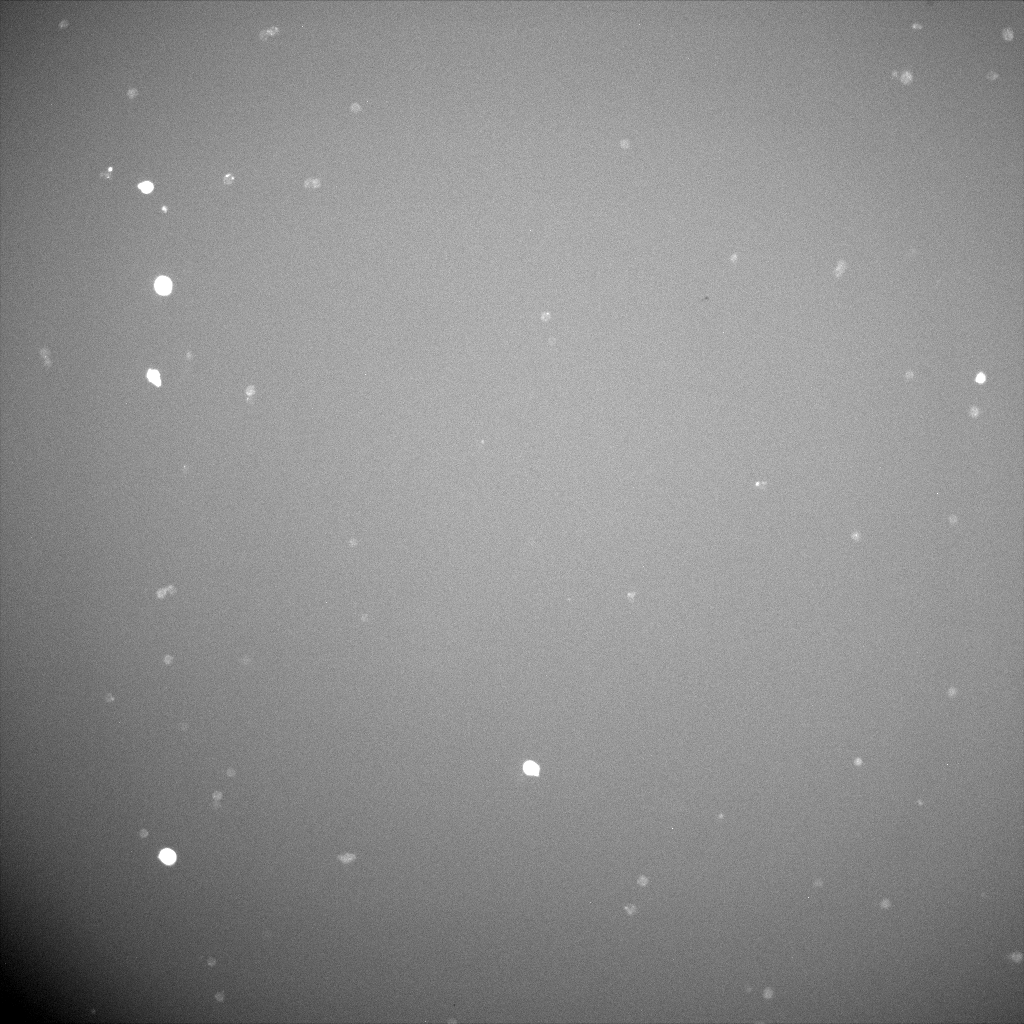

Supplement: Supplementary file 18 [file msb0011-0783-sd18.zip › Snap-168_c4_ORG.png]

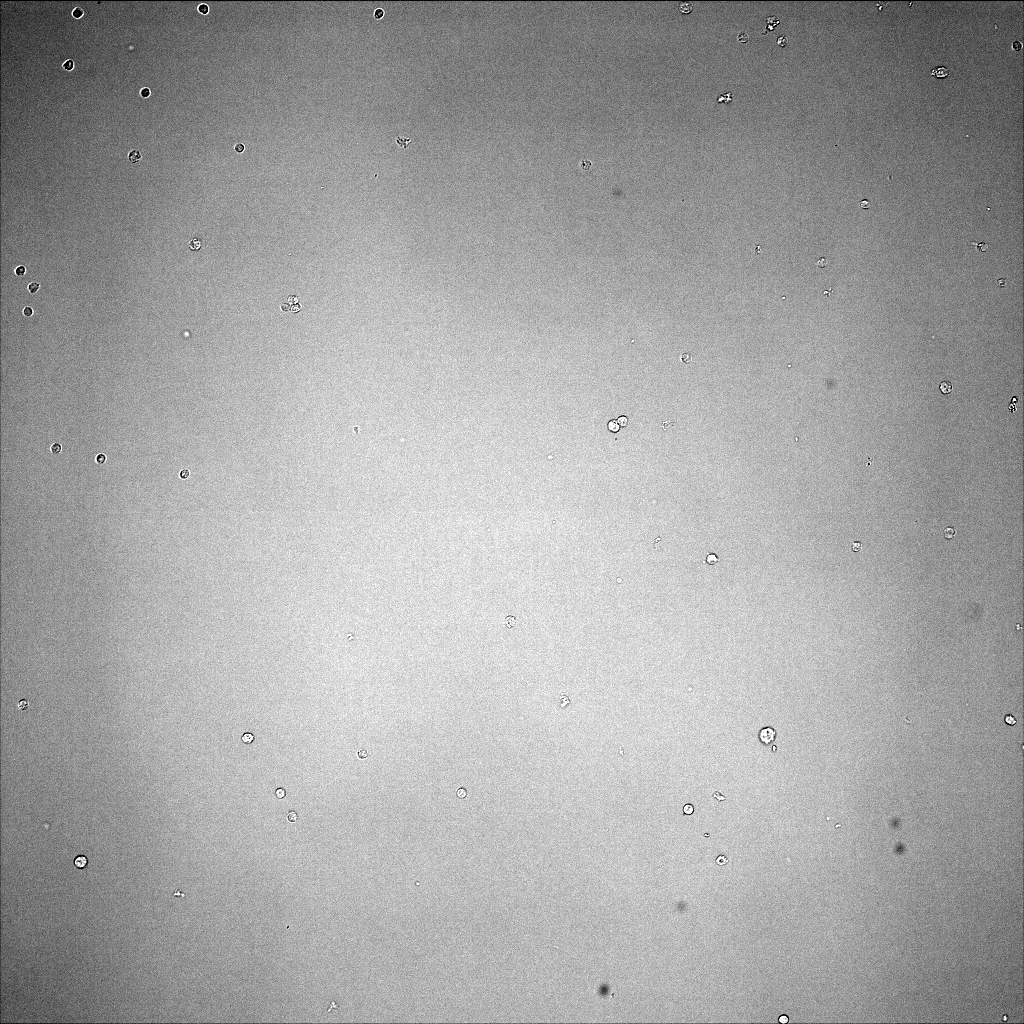

Supplement: Supplementary file 18 [file msb0011-0783-sd18.zip › Snap-169_c1_ORG.png]

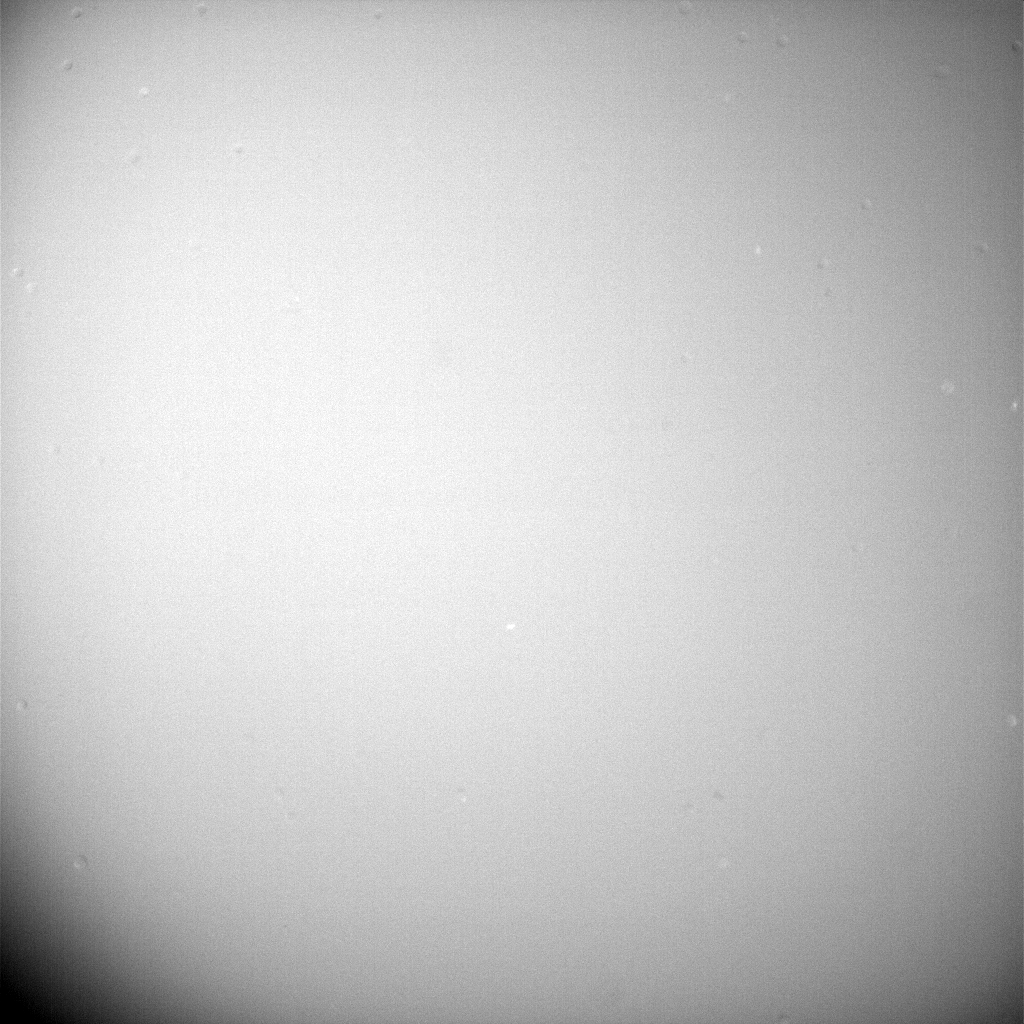

Supplement: Supplementary file 18 [file msb0011-0783-sd18.zip › Snap-169_c2_ORG.png]

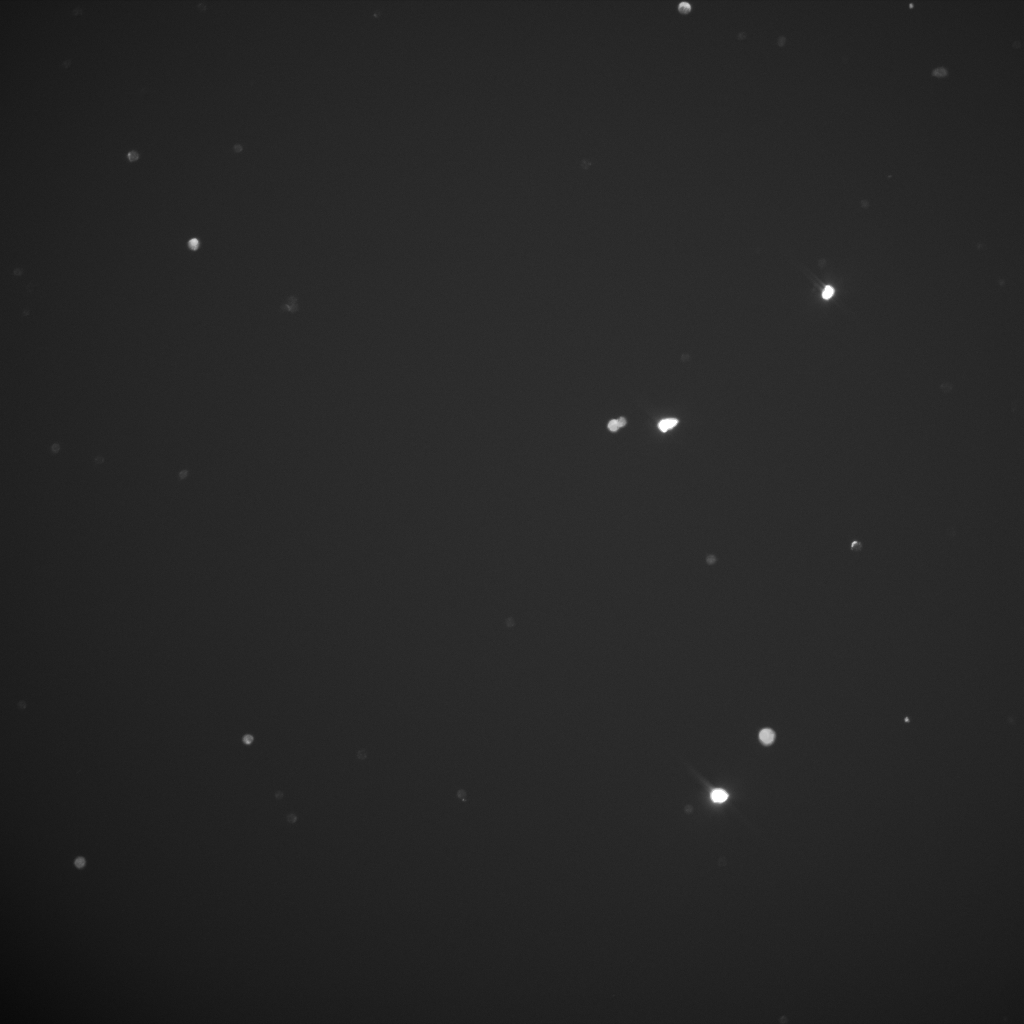

Supplement: Supplementary file 18 [file msb0011-0783-sd18.zip › Snap-169_c3_ORG.png]

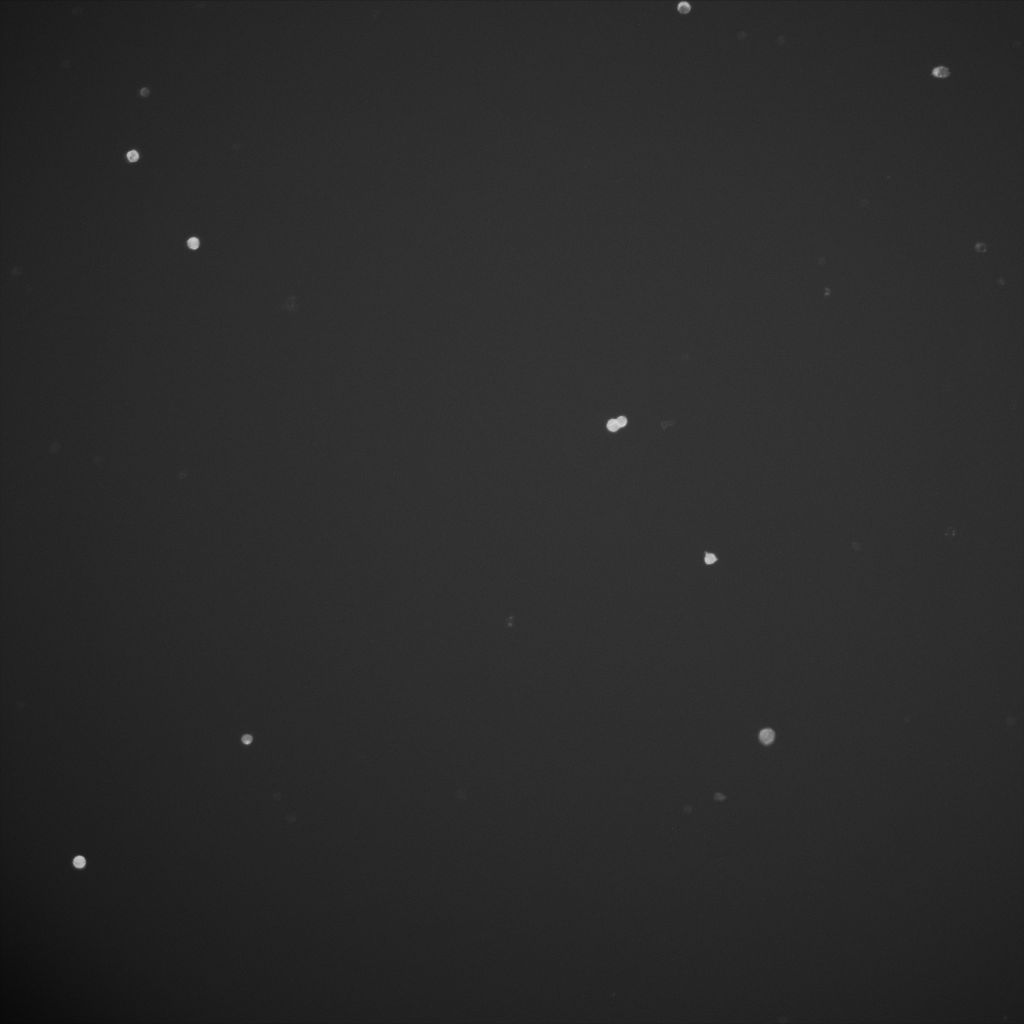

Supplement: Supplementary file 18 [file msb0011-0783-sd18.zip › Snap-169_c4_ORG.png]

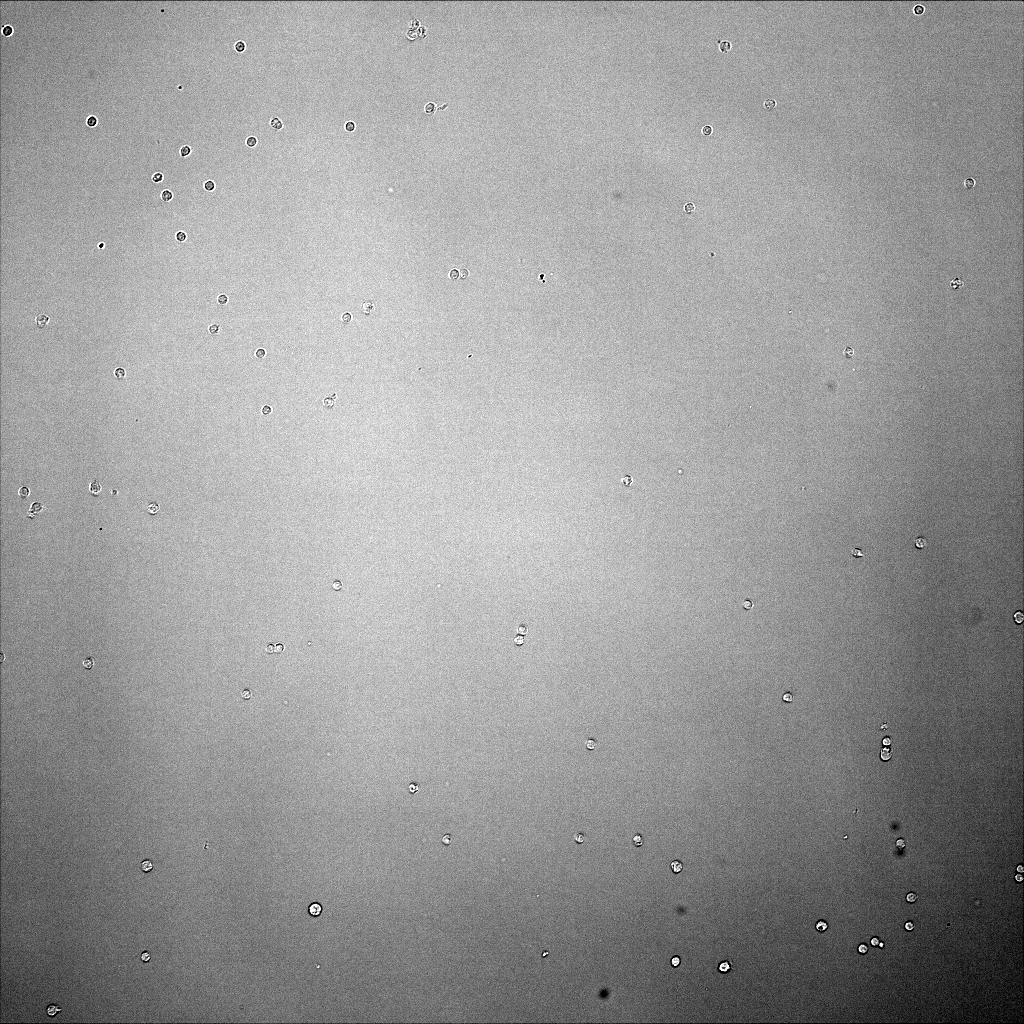

Supplement: Supplementary file 18 [file msb0011-0783-sd18.zip › Snap-170_c1_ORG.png]

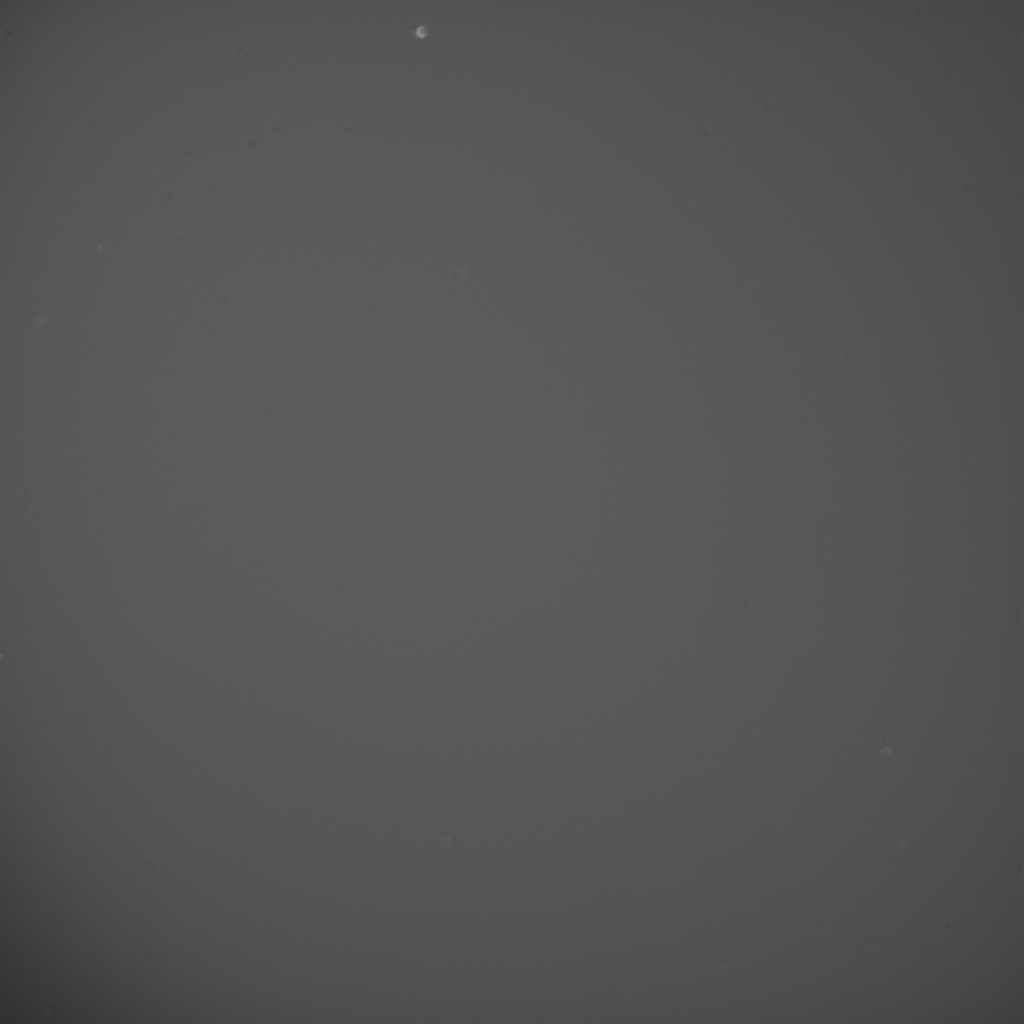

Supplement: Supplementary file 18 [file msb0011-0783-sd18.zip › Snap-170_c2_ORG.png]

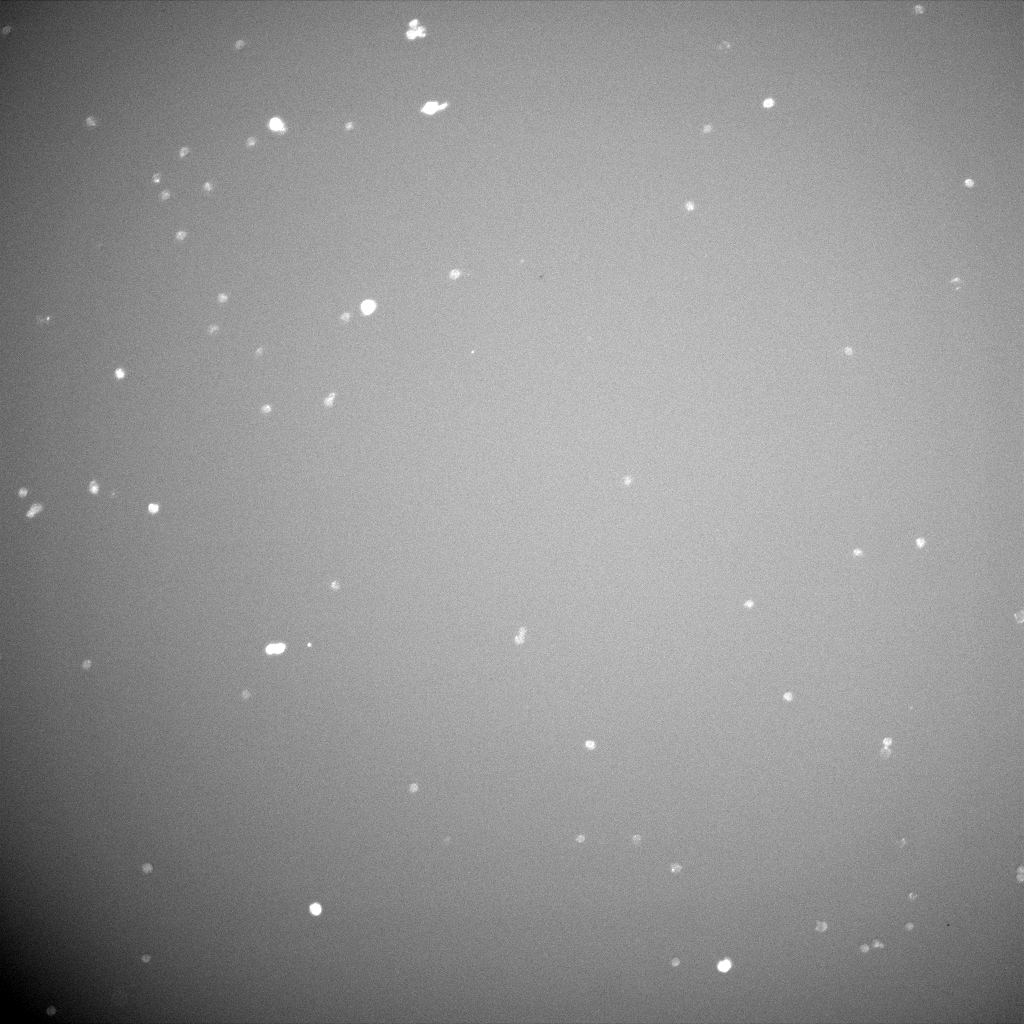

Supplement: Supplementary file 18 [file msb0011-0783-sd18.zip › Snap-170_c3_ORG.png]

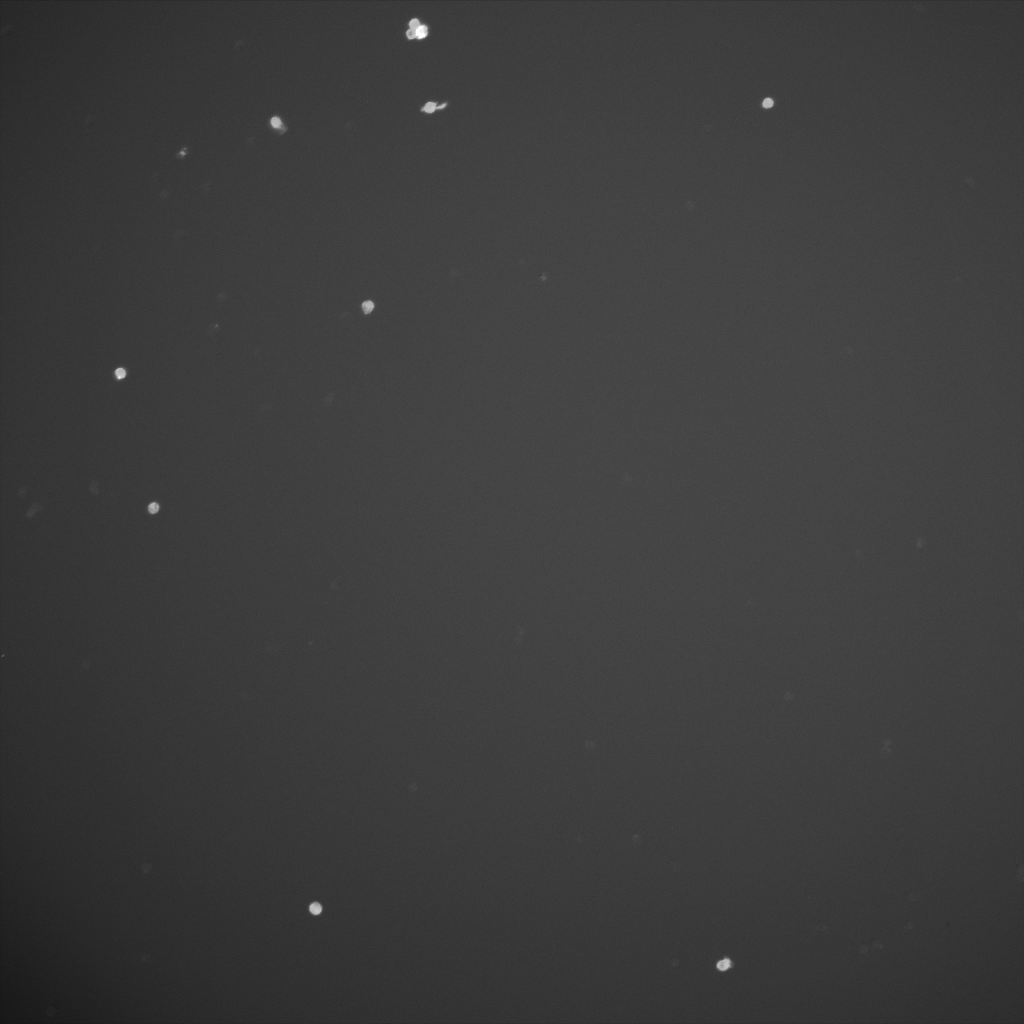

Supplement: Supplementary file 18 [file msb0011-0783-sd18.zip › Snap-170_c4_ORG.png]

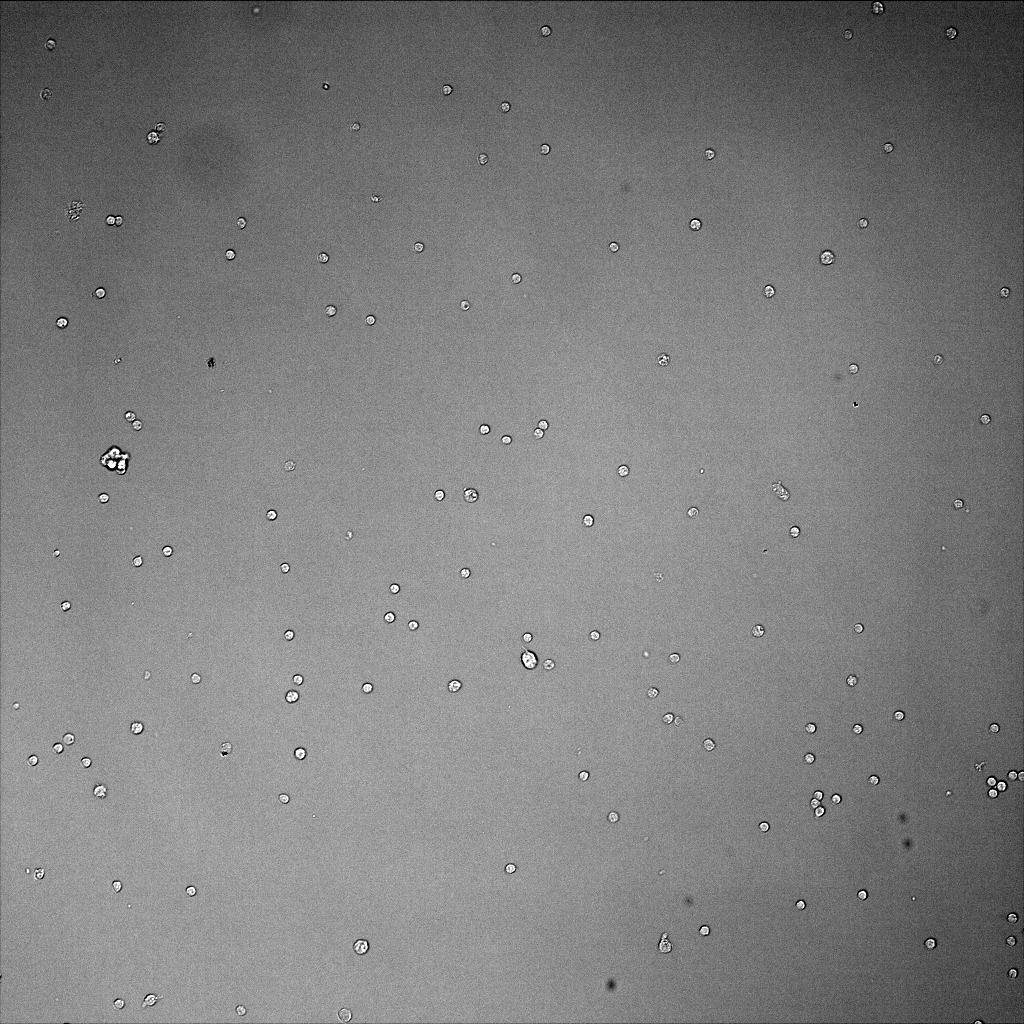

Supplement: Supplementary file 18 [file msb0011-0783-sd18.zip › Snap-171_c1_ORG.png]

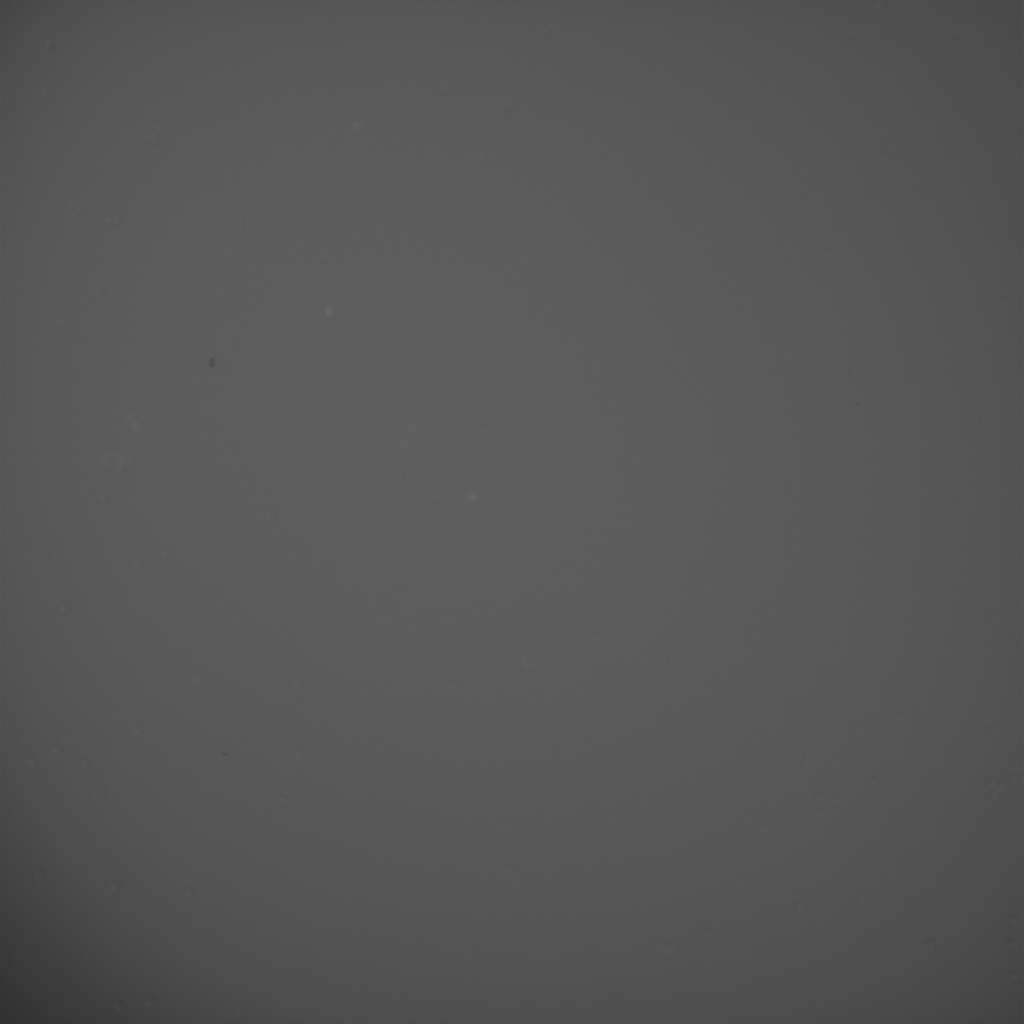

Supplement: Supplementary file 18 [file msb0011-0783-sd18.zip › Snap-171_c2_ORG.png]

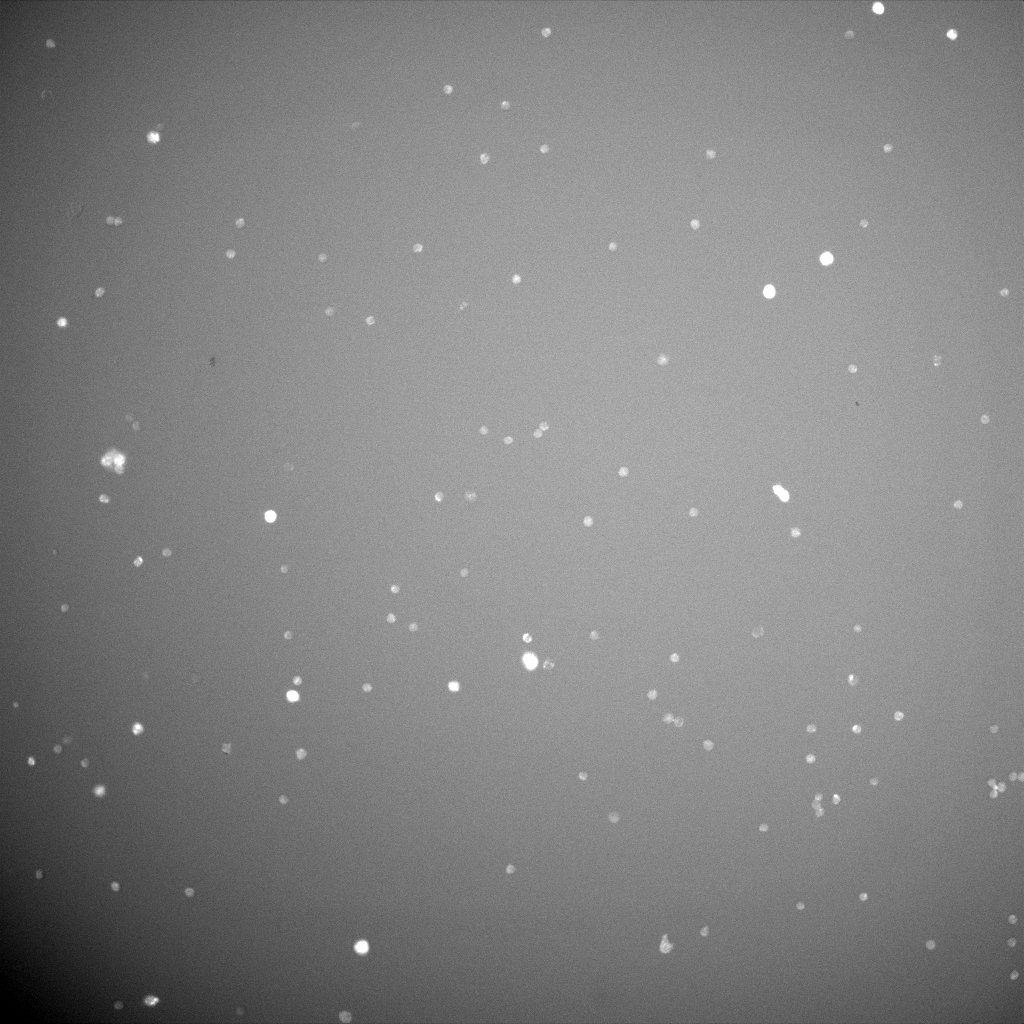

Supplement: Supplementary file 18 [file msb0011-0783-sd18.zip › Snap-171_c3_ORG.png]

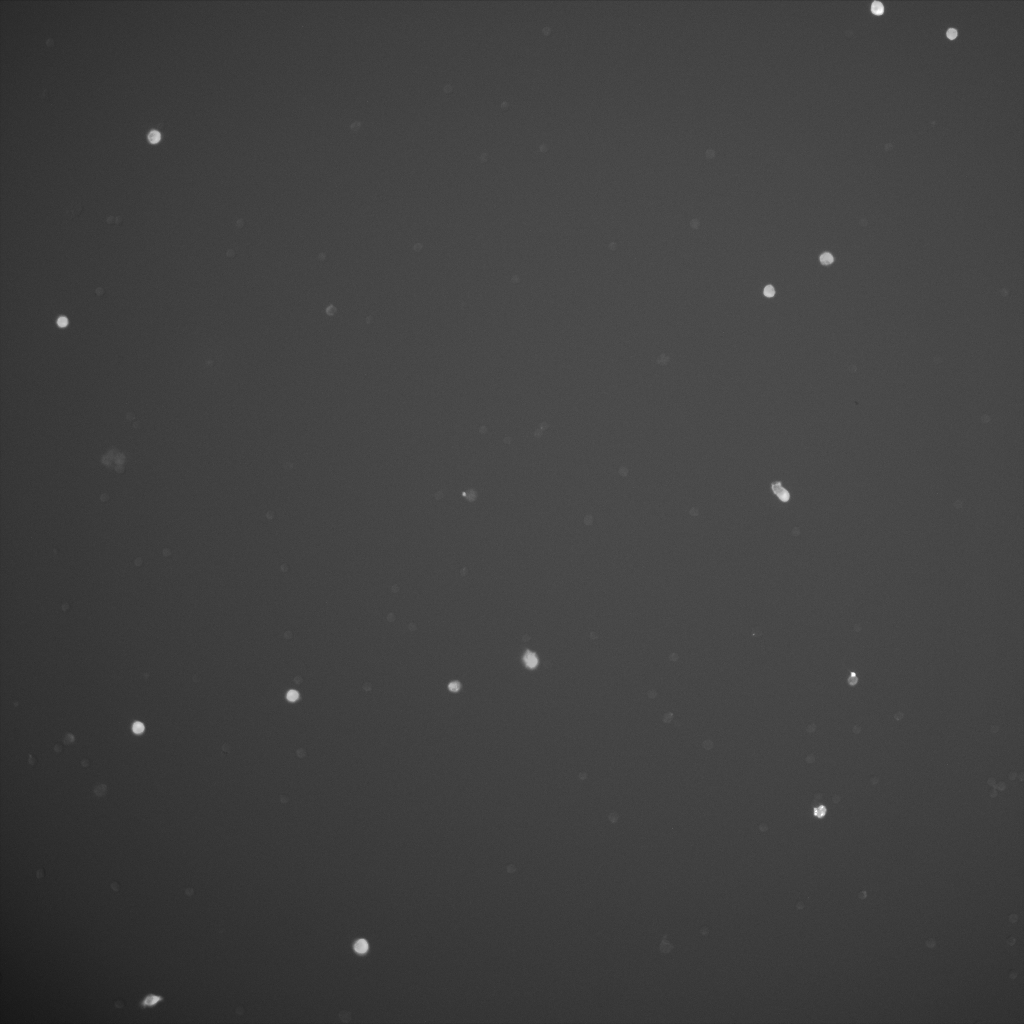

Supplement: Supplementary file 18 [file msb0011-0783-sd18.zip › Snap-171_c4_ORG.png]

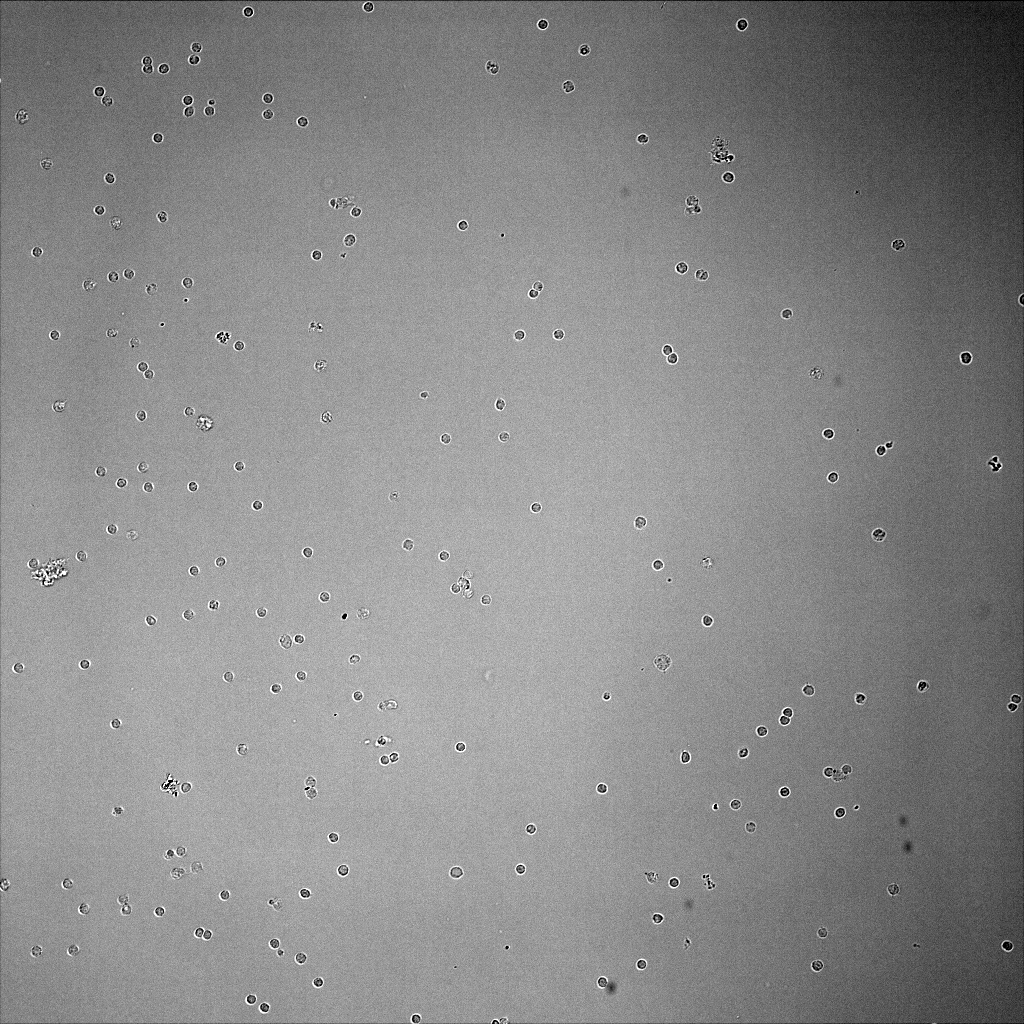

Supplement: Supplementary file 18 [file msb0011-0783-sd18.zip › Snap-172_c1_ORG.png]

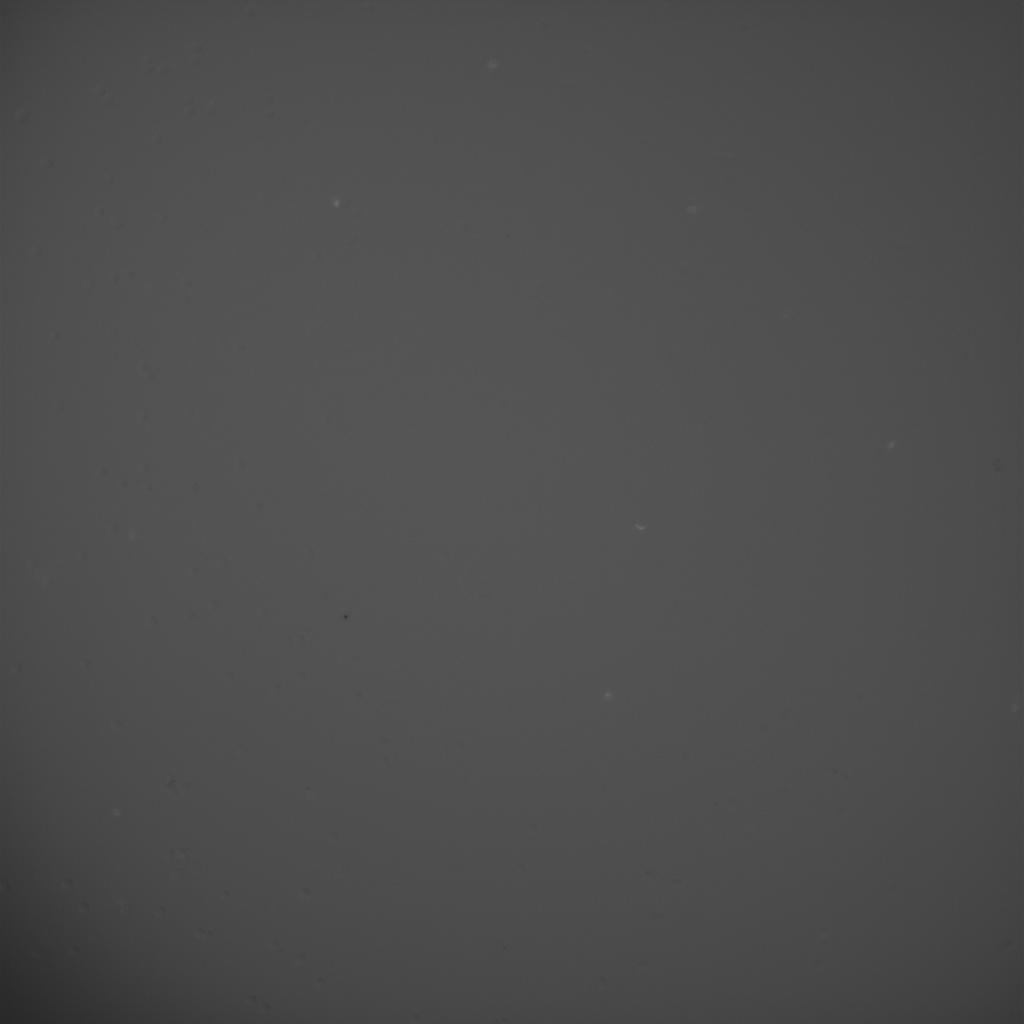

Supplement: Supplementary file 18 [file msb0011-0783-sd18.zip › Snap-172_c2_ORG.png]

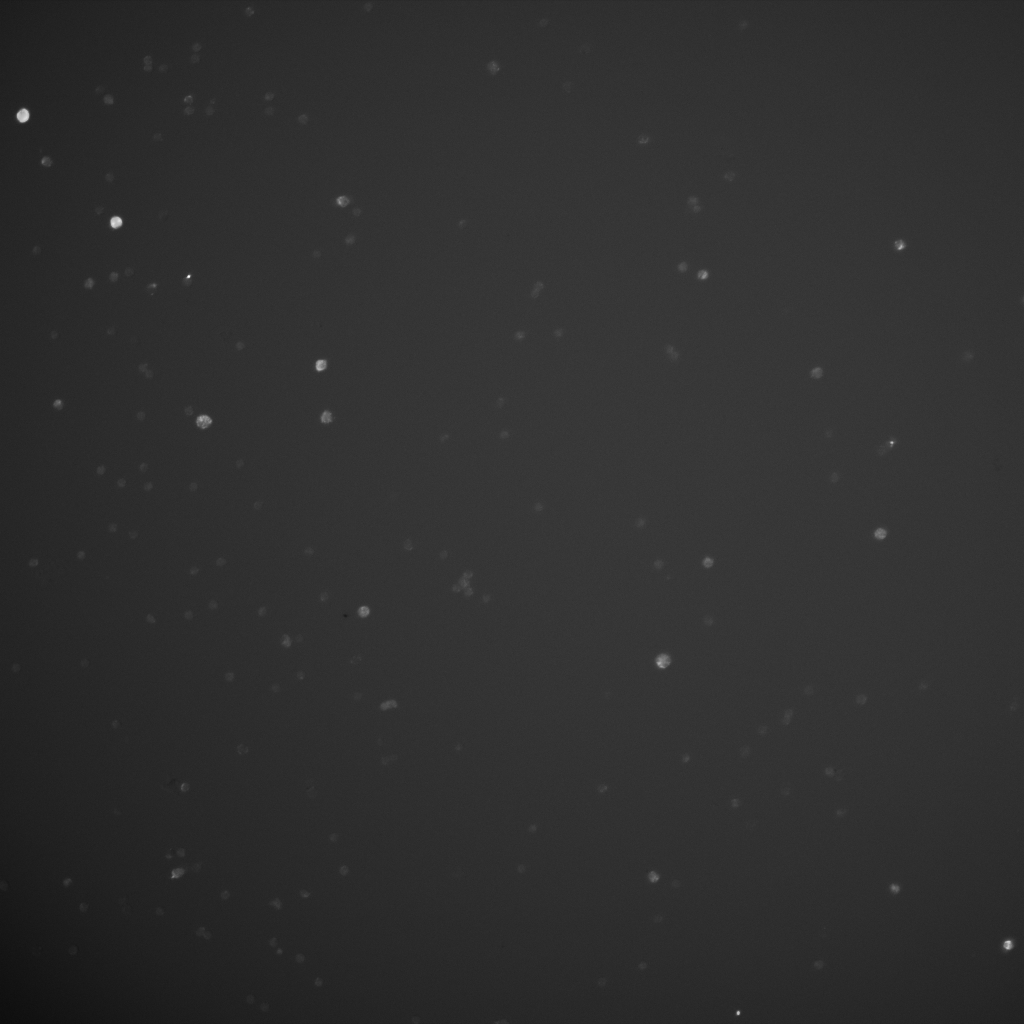

Supplement: Supplementary file 18 [file msb0011-0783-sd18.zip › Snap-172_c3_ORG.png]

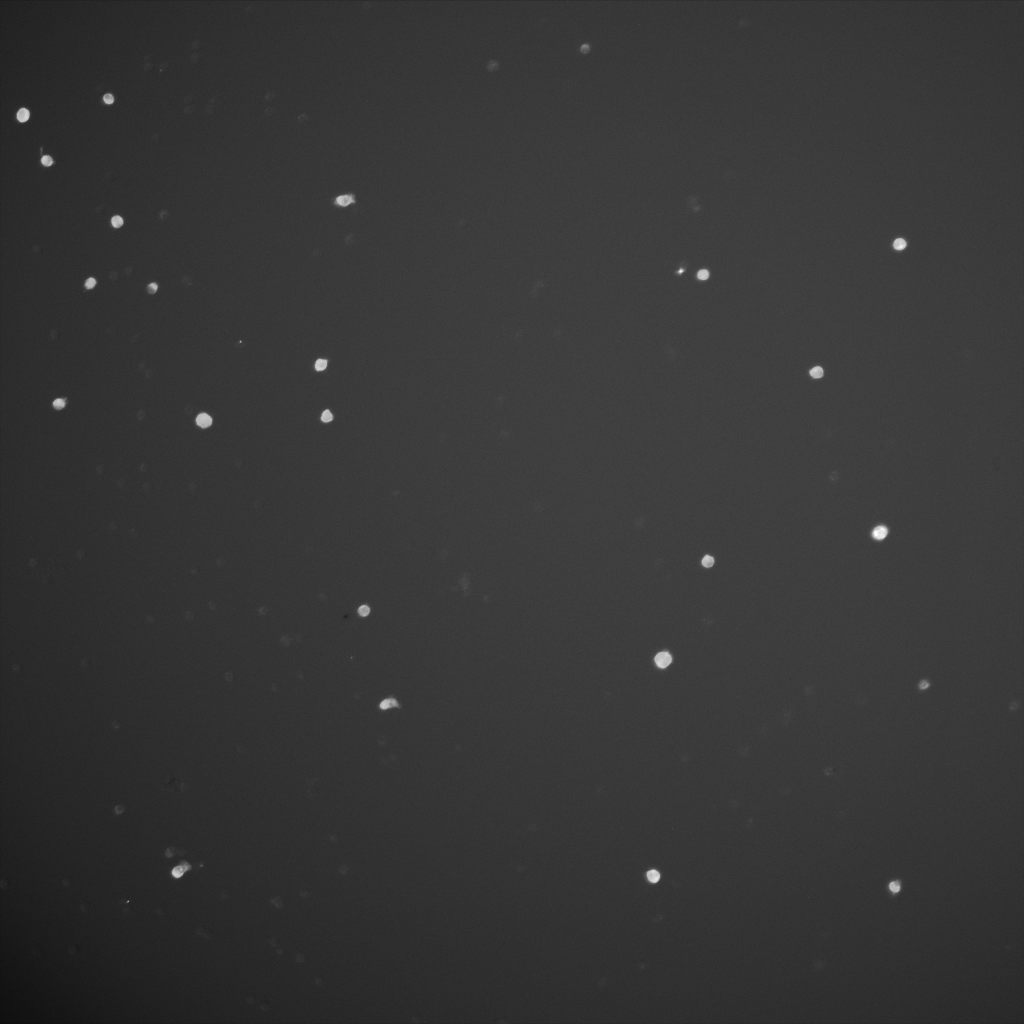

Supplement: Supplementary file 18 [file msb0011-0783-sd18.zip › Snap-172_c4_ORG.png]
