# Supplementary material for: A multi-scale approach reveals that NF-κB cRel enforces a B-cell decision to divide
Source: Mol Syst Biol. 2015 Feb 13;11(2):783. doi: 10.15252/msb.20145554 (PMC4358656; doi:10.15252/msb.20145554)
Supplement: Supplementary file 20 [file msb0011-0783-sd20.docx]

**SUPPLEMENTARY FIGURES**

**Fig. S1. Establishing death and growth thresholds.** To filter out cells that died due to artificial manipulation during preparation (mechanical death), dying generation 0 cells were binned according to their death time and biologically-relevant apoptosis was defined as death that occurred after the first wave of early death (**A**). Similarly, when ordered by death time, cells with death times higher than 12 h typically show increased separation from the previous death time (**B**). Cells that were dead at the start of time-lapse imaging were not included in this analysis. To distinguish between “grower” and “non-grower” cells, the standard deviation in cell size as a function of cell lifetime was plotted. Since the average standard deviation was 168 μm^3^, a growth threshold of roughly twice the standard deviation (350 μm^3^) was assumed to ensure significance (**C**). The final size of cells is bimodal with cells above 800 μm^3^ delineating the large cell peak (**D**). This was used to justify classifying cells with final size > 800 μm^3^ as growers in addition to cells which grew by 350 μm^3^.

**Fig. S2. Cells typically grow less prior to the last division.** Wildtype purified naïve B cells were stimulated with 250 nM CpG and tracked by time lapse microscopy. The fraction of “non-growing” cells that divided (**A green**) or died (**A red**) are shown for each generation. Error bars = 1/n. The quantification was repeated with penultimate dividing cells removed from consideration (**B**).

**Fig. S3**. **FlowMax deconvolution of WT 250 nM CpG stimulated CFSE time series.** CFSE-stained purified murine B cells were stimulated with 250 nM CpG, assayed by flow cytometry and a computational tool, FlowMax, was applied to determine non-redundant sets of maximum-likelihood population model parameters (**A**).To test if a molecular race or a decision are more appropriate, the cyton model, which assumes a race between division and death, (**B**) and fcyton decision-based model (**C**) were used as part of the FlowMax phenotyping. Maximum-likelihood parameter sets obtained from fitting each model to the experimental CFSE time courses were sampled using a Monte-Carlo approach to expose the fraction of responding cells in each generation (Fs), as well as division and death time distributions for undivided (Tdiv_0_,Tdie_0_) and dividing cells (Tdiv_1+_,Tdie_1+_) expected to be observed after any potential censorship and compared to the actual cellular distributions measured by time-lapse microscopy (**D**). The best-fit modeled CFSE histograms from the best-fit cluster of cyton (**E purple**) or fcyton (**E blue**) model parameters overlaid on experimental CFSE histograms collected in duplicate across six days at the indicated time points.

**Fig. S4**. **Cell fate and timing are correlated between siblings and cousins.** B cell lineages from WT 250 nM CpG time-lapse microscopy video were determined by semi-automated tracking. The fraction of differing fates, difference in timing to the start of growth (Tgro), and correlations in division (Tdiv), or death (Tdie) of sister cells (blue) and cousins (red) were calculated. The fraction of sister or cousin cell pairs experiencing different fates (i.e. division and death) is plotted (**A**). Histogram of the differences between the times to decide to start growing (**B**) is shown. Division (**C**) and death (**D**) times are plotted between sister and cousin cells across all generations. Colored lines represent the linear fit through the origin with r-squared values for sisters (top) and cousins (bottom) as indicated.

**Fig. S5.** **NF-κB RelA, Bcl_XL_ mRNA, and mTORc1 inhibition assays.** Analogously to NF-κB cRel, NF-κB RelA average fluorescence was quantified at 0 h and 24 h post stimulation (**A**). Bcl_XL_ mRNA levels were measured by quantitative PCR at 0, 1, 6, and 20 h in wildtype and NF-κB cRel deficient B cells (**B**). Error bars indicate SD. Average cRel fluorescence at 0 h and 24 h after 1 h pretreatment with 1 ng/mL rapamycin, an inhibitor of mTORc1 (**C**). Quadrants delineate significance at 24 h compared to 0 h. Large cell size was defined as >100 pixels empirically to avoid cell-selection bias.

**Fig. S6. Extrinsic noise results in cell-to-cell NF-κB, division, and death variability.** Models for NF-κB signaling, cell-cycling, and apoptosis are summarized (**A**). Bolded species represent the input (active IKK), and output event nodes (cleaved PARP signals death, cdh1 accumulation signals cytokinesis). Extrinsic noise in the form of protein synthesis and degradation rate variability (~normal(rate,rate*0.1)), total protein abundance (~log-normal([protein], [protein]*0.25)), and initial cell size (~normal(1,0.07)) was introduced to each module and 100 simulations were carried out. Active IKK (**B**), nuclear RelA:p50 and nuclear cRel:p50 (**C**), cyclin and cdh1 (**F**), cell mass (**G**), Bcl_XL_ (**H**) and cleaved PARP (**I**) are shown. The published cell-cycle model ([Conradie et al, 2010](#_ENREF_10)) which does not include cell growth control (**D**) and the results for solving 100 independently-distributed simulations (**E**) are shown for reference. Overlapping histograms indicate division (green) and death (red) events. Error bars in (**B,C**) represent s.d.

**Fig. S7. Predicted and experimental average growth trajectories and timing distributions.** Wildtype (red), NF-κB cRel deficient (blue), lower stimulation (green), and rapamycin pretreated (purple) conditions were simulated by removing NF-κB cRel monomer from the NF-κB module, increasing activated IKK rate, and decreasing protein synthesis rates by 30% (**A**). Side by side comparisons of model and time-lapse imaging results from analogous conditions: average size trajectories for cells that increased in size (**B**), cells that did not increase in size (**C**), cumulative distributions for the time to start growing (**D**), time to divide (**E**), and time to die (**F**).The resulting cumulative distributions of the time to start growing (Tgro), time to divide (Tdiv), and time to die (Tdie).

**Fig S8. Unstimulated B cells die within several days *in vitro*.** Wildtype B cells were purified from mouse spleen and suspended at a concentration of 200,000 per mL in complete media. Viable cells were counted at the indicated times by flow cytometry using propidium iodide, and light scatter to exclude dying or dead cells. Error bars are SE for duplicated counts (two wells containing cells from a single spleen sampled at the same time).

**SUPPLEMENTARY TEXT**

**Text S1. Extended methods. CFSE Flow cytometry analysis using the FlowMax computational tool,** calculating the expected probability that a dying cell would have started growing, additional multi-scale modeling methods, model fitting, and parameter sensitivity methodology are described.

**SUPPLEMENTARY FILES**

**File S1. Force Algorithm Semi-automated Tracker Software**. Java-based software developed for tracking B cell populations using a semi-automated curation approach, and physics-based active contour modeling of individual cells.

**File S2. Video of tracked WT B cells stimulated with 250 nM CpG.** Tracked cells are colored and overlaid on phase-contrast images.

**File S3. Video of tracked WT B cells stimulated with 10 nM CpG.** Tracked cells are colored and overlaid on phase-contrast images.

**File S4. Video of tracked NF-κB cRel deficient B cells stimulated with 250 nM CpG.** Tracked cells are colored and overlaid on phase-contrast images.

**File S5. Video of tracked WT B cells stimulated with 250 nM CpG and subject to 1 h pretreatment with 1 ng/mL rapamycin.** Tracked cells are colored and overlaid on phase-contrast images.

**File S6. Single-cell RNAseq gene counts and analysis.** Microsoft Excel spreadsheet containing the workup of the single-cell RNAseq results.

**File S7. Immunofluorescence images and analysis tool.** All immunofluorescence images and the custom Java software used to analyze the images are provided as a zipped file

**File S8. Multi-scale integrated B-cell model files.** Matlab files required to run the multi-scale B-cell model.

**SUPPLEMENTARY TABLES**

**Table S1. Correlations between cell transcriptomes.** Transcriptomes of five large and five small cells were sequenced and aligned to the mouse genome. Gene counts were normalized to RNA spike in pseudo-counts and a constant positive count of 100 was added to all genes to represent experimental sensitivity as seen in the variability in spikein counts between samples (see methods). Colors represent degree of correlation (Pearson’s r). The second large cell was removed from further analysis due to poor correlation with all other cells (similar to negative).

**Table S2. NF-κB target genes that are transcriptional regulators.** A list was adapted from ([Gilmore, 2014](#_ENREF_18)). Transcription factors with motifs upregulated in large (red) or small (green) cells have been highlighted.

**Table S3. Integrated B-cell model species.** Prior to simulation, the model was equilibrated starting from initial concentrations, x_0_, with basal IKK signaling and no death signal (L=0) for a period of 24 h. Due to relatively high initial cyclin levels (see Conradie, 2010), the equilibration was repeated until the cell stopped dividing ([Mass] and [GM] was halved as usual) followed by a 24 h period of quiescent equilibration.

**Table S4. Integrated B-cell model rate constants.** Rate constants used to simulate the physicokinetic models for NF-κB signaling, cell-cycle progression, and apoptosis. Note: We did not model mTOR explicitly in the model because the exact relationship between Myc and TOR is not well characterized in B cells. In our study we found that mTOR is downstream of NF-κB activation, but the exact mechanisms are still poorly known. Instead, we assume that cell growth and the synthesis of general cellular machinery can be approximated by Myc activation, which is known to be under NF-κB regulatory control. The rate constants that we introduced into the model are explored in further detail below (Table S8).

**Table S5. Integrated B-cell model flux reactions.** The fluxes governing the mass-action changes of species in the model as listed.

**Table S6. Integrated B-cell model reactions.** ODE reactions describing the species rate of change are shown in terms of parameters and species. Some reactions were updated to reflect additional species that were introduced to model connections between the three models. Please see Text S1 and Tables S8,9 for more details.

**Table S7. Other simulation parameters.** Parameters describing the population variance (extrinsic noise) within specific modules of the model, multipliers, and general simulations parameters are shown. For more details please refer to the provided Matlab simulation files (File S8).

**Table S8. List of constants in new model reactions.** Constants present in novel model reactions (see Text S1). These values were derived from previously studies or from the datasets in this study. Note that ***f_RE_*** is a fraction that is depenent on the current abundance of specific Rb species in the cell-cycle module as described in the original model, included here to highlight that it was not a fitted quantity.

**Table S9. Parameterization of free parameters and sensitivity analysis.** Free parameters that were part of novel model reactions (see above) were manually fitted according to biological features observed in microscopy datasets of this study. Ranges for values were selected to reflect known distributions for protein and mRNA half-lifes (~1 h to ~100 h) or values which allowed for experimentally observed IKK dynamics, Tdie distributions, NF-κB monomer abundances, and observed population cell count trajectories.

**Table S10. Population response features being fitted during model parameterization.** Description of features identified in the experimental datasets which were selected for manual fitting of model parameters (Table S9).

**Table S11. Evaluating model fitting to experimental WT, cRel KO, low CpG, and Rapamycin treated datasets.** A comparison of the model fit to experimental time-lapse microscopy datasets for WT high CpG, cRel deficient, WT low CpG, and rapamycin treated conditions. Various measuremens of similarity for average cell growth trajectories (Figure S7 B,C), average Tgro, Tdiv, and Tdie timing (Figure S7 D,E,F), and total cell trajectories (Figure 6) are shown.
