# Supplementary material for: A multi-scale approach reveals that NF-κB cRel enforces a B-cell decision to divide
Source: Mol Syst Biol. 2015 Feb 13;11(2):783. doi: 10.15252/msb.20145554 (PMC4358656; doi:10.15252/msb.20145554)
Supplement: Supplementary file 21 [file msb0011-0783-sd21.pdf]

A multi-scale approach reveals that NF B enforces enforces a B-cell decision to divide

Maxim N. Shokhirev, Jonathan Almaden, Jeremy Davis-Turak, Harry A. Birnbaum, Theresa M. Russell, Jesse Vargas, Alexander Hoffmann

Corresponding author: Alexander Hoffmann, University of California, Los Angeles

Review timeline:

Submission date:	16 August 2014
Editorial Decision:	26 September 2014
Revision received:	15 November 2014
Editorial Decision:	26 November 2014
Revision received:	21 December 2014
Accepted:	23 December 2014

Editor: Thomas Lemberger

Transaction Report:

(Note: With the exception of the correction of typographical or spelling errors that could be a source of ambiguity, letters and reports are not edited. The original formatting of letters and referee reports may not be reflected in this compilation.)

1st Editorial Decision 26 September 2014

Thank you again for submitting your work to Molecular Systems Biology. We have now heard back from the three referees who agreed to evaluate your manuscript. As you will see from the reports below, the referees find the topic of your study and your approach of potential interest. They raise, however, several issues that should be convincingly addressed in a revision of the present study.

Without repeating all the points noted by the reviewers, some of the major issues refer to the following:
- the way model parameters were estimated/chosen should be clarified and the impact of these choices should be analyzed.
- model fits and predictions should be analyzed and tested more rigorously with appropriate statistics.

Reviewer #1:

Literature points NF B as a regulator for coordinating cell growth, death and division in B cells. Whether the B cell fate decision of division versus death is a stochastic event or determined during the growth phase was unclear. Shokhirev et al. combined experimental and computational approaches, and demonstrated predetermined B cell fate during the growth phase. Furthermore, the multi-scale mathematical model allowed simulation of cellular phenotypes in response to various perturbations such as reduced exogeneous stimulation, gene knockout, increase protein abundance

© European Molecular Biology Organization

and increase protein variability. This led to predictions such as that cRel protects growing B cells from death.

The main finding of this study is that in wild type B cells, the division versus death fate decision is predetermined during the cell growth phase. Growing B cells predominately undergo division upon reaching a certain cell size because of the action of cRel.

Shokhirev et al. employed time-lapse imaging and single cell RNA-seq to identify signature genes of large versus small B cells. The results confirmed previous finding that NF B activity marks B cell growth. Then, then the authors used cRel knockout B cells to characterized NF- B-mediated molecular network for B cell growth, division and death. Subsequently, an ODE-based multi-scale model was developed based on 3 existing models - B cell NF B signaling, cell cycle, and cell apoptosis.

General remarks

Based on the presented experimental data, I am not fully convinced by the conclusion that B cell fate decision (especially division) is predetermined in the growing phase. See major point #1.

It was difficult for me to determine if the authors model / data integration, especially as related to cell size aspects, was or required correction for concentration/ dilution effects due to volumetric normalization (i.e., were production rates adjusted for cell volume or was a molecular dilution component included). It would be helpful to more explicitly state how this was handled, wand there or not these parameters could be relevant.

The creation of a multi-scale model that incorporates molecular and cellular dynamics is a technical advancement. Many mathematical models have been developed for specific biological processes, such as NF B pathway activity, cell cycling and cell death. However, those biological processes do not act independently to give arise a biological phenotype. Being able to concatenate models for a series of processes in one framework and generate testable hypotheses is a true contribution to systems biology techniques.

The multicellular and dynamic nature of the immune system makes it a good platform for the development of multiscale models. Existing models such as the one development by Palsson et al. (BMC Systems Biology 2013, 7:95) connect modules horizontally through fluxes. Shokhirev et al. concatenated signaling pathway and transcriptional regulatory networks through synthesis of transcription factor, which is major advancement from previous knowledge.

The modeling framework would be interesting to the systems biology community. The biological findings should be interesting to audience who studies cell fate decisions.

Major points

The key conclusion of this study is that B cell fate choice between division and death is pre-determined during the cell growth phase as demonstrated by time-lapse experiments, where in wild type B cells, the occurrence of division in growers was significantly higher than in non-growers (Figure 3). In addition, the authors suggested that cRel controls both cell growth and cell survival (Figure 4).

However, the empirical and simulation results of cRel knockout experiments were somewhat counter-intuitive to the conclusion. In cRel-knockout B cells, the growth profiles of B cells were not affected comparing to the wild type (Figure E7), but the fraction of death in growers was 4 times higher (Figure 6G). The results mean that cRel did not significantly control cell growth, but the choice between cell division and death. Therefore, cell fate decision is regulated independent of cell growth profile. Could the authors elaborate on their conclusions that "B cells may make a decision to enter a sustained growth phase that biases their fate towards division rather than death" in the abstract.

It was difficult for me to determine if the authors model / data integration, especially as related to

cell size aspects, was or required correction for concentration/ dilution effects due to volumetric normalization (i.e., were production rates adjusted for cell volume or was a molecular dilution component included). It would be helpful to more explicitly state how this was handled, wand there or not these parameters could be relevant.

To demonstrate the causative relationship between sustained growth and cell division, the authors could disrupt cell growth profiles and quantify the frequency of cell division.

Minor points

- Presentation and style

o I do not quite understand the percentages labeled on Figures 4F - 4K. Are those percentages for the 24h data?

- Trivial mistakes

o Page 5 "Interestingly, following the first generation, there was a significant..." the sentence needs to be revised.
o Page 7 paragraph 2 "we found that compared to a 0 h control, B cells were larger had higher cRel", check grammar
o Page 9 last paragraph, "next, we simulated the effect cRel-difficiency by setting the translation rate of the cRel protein to zero"
o Page 11 paragraph 3, figures are missed referenced.

Reviewer #2:

In a well-written article, Shokhirev et al. present a multi-scale approach that couples agent-based modeling with single-cell transcriptome and immunofluorescence analyses to elucidate the role of NFkB cRel in enforcing cellular decisions towards mutually exclu-sive fates. The paper seeks to answer an important biological question regarding cell fate -- do processes leading to cell-division and apoptosis proceed concurrently (molecular 'race' hypothesis) or is cell fate pre-determined ('decision' hypothesis that commits cells to either fate)? Although the main biological concepts/paradigms presented in this paper have largely been reported before, this manuscript does provide important contextual data supporting the 'decision' model, thereby arguing against the findings of a 2012 Science paper (Duffy et al., 2012) that supported the 'race' hypothesis. In addition, this work ex-hibits a very nice presentation of the systems biology approach - integrating single-cell experimental data with multi-scale computational modeling and demonstrating that these complex models can in fact be predictive.

In general, the conclusions are well supported and the work is of significant interest to the readers of Molecular Systems Biology. A few minor issues/questions should be addressed:

1. The lines and arrows as well as the time distributions in Figures 3A and 3B are a bit confusing. Should Figure 3A include the early branching? Given that these figures highlight the main biological question on cell fate determination, it might be worthwhile to clarify or re-draw.

2. How sensitive are the results to the cell size thresholds chosen? What about the cells that were just outside of the chosen bounds? Are these the cells that end up dividing in Fig. 3D (cells that divide in the population of "non-growers"?)

3. Some care should be taken when describing the "molecular determinants of cell fate decision." Shokhirev et al. highlight several upregulated and downregulated genes in large vs. small cells. Since the cells are already 'large' versus 'small', it should be noted that the upregulated genes did not necessarily cause the cell fate decision. The upregulated genes could be another phenotype of something up-stream that triggered the cell fate decision. An explicit discussion of this might be useful to the readers.

© European Molecular Biology Organization

4. In the multi-scale model, values for certain free parameters specifying BclXL, CycD, and Myc transcript synthesis and degradation were manually selected from within 'biologically plausible' ranges? How was 'biologically plausible' defined here? How different were these ranges? Given the importance of these parameters to obtaining the model fits, it would be helpful to highlight them by separating them out from the full parameter table.

5. In Figure 2E, the authors noted that progenitors that grew exhibited an initial growth delay followed by rapid growth. Meanwhile generation 1+ growers did not exhibit any delay phase. In Figure 6, the authors note that model simulations suggest that cell growth is not cRel-dependent. Through model perturbations, could the authors delineate what might be causing the initial growth delay in pro-genitors? Some clarification here would be helpful to better understand the model.

6. In Figure 7, the authors showed that the probability of observing dying cells that had grown tripled when cells lacked cRel. However, this was not seen under ra-pamycin treatment (which results in defective cell growth). The authors explain this by stating that cRel's function may be to enforce a decision to divide and that the population response of cRel-deficient cells resembles a molecular race model. The authors should expand on the discussion regarding the specific role of NFkB here for further clarification.

7. Why are error bars only included in Fig. 8F and 8J and not in the other panels?

8. References to Fig. 8 in the last paragraph of the Results section are incorrect.

9. In the text discussion of the IF in "big" cells versus all cells, are the modest in-creases in % reported for big cells significant?

Reviewer #3:

This is an ambitious paper attempting to connect dots across three organisational levels. The strategy at the heart of the paper, that information from top down can inform the selection of possibilities in a bottom up model and vice versa, is sound. However, it is certainly not easy to get ones head around all the parts, and ultimately it is held together with a good dose of faith in the author's judgement in setting the parameters for a large number of ODEs.

There are four sections that hold up the logical fabric:

1. The paper begins with imaging of B cells following stimulation with CpG. Tracking and following cells is performed with new software for manual guided extraction of data. I have not evaluated these - but the code is provided to do so. The data produced seems similar, to the work of Hawkins et al (PNAS, 2009). The main purpose of the data is to develop a population based parametric model - or at least to choose between two alternatives - the competing 'race' model of Hawkins et al. (PNAS 2007) and the 'decision model' (for example Hyrien et al. Biology Direct, 2010). By comparing the predicted proportion of cells that grow and die the decision model is selected. The logic seems to hinge on two observations - 1. The data from figure 3D showing that, at least for the first four generations, all 'growers' went on to divide; and 2. Having shown, paradoxically, that some non-growers divide, a test is developed to evaluate whether they might have been also able to 'die' based on the distribution of times to die. However, this latter proof seems to be applied only to generation 0 cells in the figure and in the supplementary description. The authors should clarify this second argument and confirm it works as well for each generation. Returning to the first argument the authors should explain why they focus on the first four generations and not the latter two, which look to be giving a different answer. Curiously Fig. 3F also seems to suggest that perhaps neither model is giving the right answer and further alternatives could be developed.

The authors then turn to using a population-based version of the two models to apply to CFSE time series data, again to help determine which might be the better description of events. Here, no full statistical evaluation is given (or at least it wasn't used to distinguish them). Both models seemed to do a very good job of fitting to the dividing CFSE populations, but the race model was clearly unable to accommodate the rather large level of death in generation 0. Hence, again, the decision model was chosen.

© European Molecular Biology Organization

Coming to the end of this section, and knowing what is coming, I was left wondering why so much time was spent establishing this model. Both models, tested are published and either could simply have been adopted, with little consequence, one or the other for the subsequent work. If the authors genuinely want to evaluate and develop a 'correct' model of the cellular dynamics then much more work would need to be done on the data sets, and more sophisticated models than used here could be developed and compared with appropriate statistical evaluation, taking account of the number of parameters being exploited and the quality of the fits to the complete data sets. This point is brought up again in the last results section where c-Rel loss is used to illustrate that in fact some race for fates might be occurring, although not at the level of the simple model that assumes complete independence of division and death times. Clearly the situation is not resolved, and this work manages to point out deficiencies in all models to date, although that is not stated by the authors.

2. The second section aims to identify the key molecular determinants of cell fate decisions by single cell analysis. This is done using fluidigm and imaging to evaluate mRNA expression in 24 h small versus large cells. Only 5 cells of each are tested. There does not seem to be any additional value in the single cell approach as the data simply reveals broad differences in expression of fairly well known targets of the CpG pathway. Perhaps the authors can provide more discussion of why they went this route, and again, why it was necessary as published studies of NFkB could have been used to justify the use of the NFkB pathway to drive cell growth and survival in the agent based model development in the next section. A stronger and clearer argument for what particular result is new here, and is used to inform the next section would be helpful.

3. The third section builds a very complex agent-based model for B cell division, death and signalling that stitches together published ODE models of NFkB signalling, apoptosis and cell cycle control. It is claimed that some variations, such as a link to Myc to promote cell growth, were implemented in a new way.

This section is in need of more depth in evaluation and description. The number of parameters governing each agent is astonishing (>200) and there is almost no discussion of how sensitive the model is to various changes or conditions of these components that make up the model. Most of the adjustment in the model seems to be in changing dynamics of only three components (Myc, Bclxl and CycD). Figure 5C is described as a remarkable recapitulation of the observed cellular dynamics and yet, to my eye, it looks quite different and we are left with this subjective manner of evaluation. Why not fit to the data? Granted this agent-based model method is not well suited to classical statistical approaches making it difficult to evaluate. We are asked to take a leap of faith that the various parameter ranges are reasonable and the model is giving a qualitative 'fit' based on a reasonable re-creation of the underlying molecular processes. It is virtually impossible to know if this is correct without predictions and careful evaluation, which is attempted in the last section.

4. In the fourth section agents from section 3 are used to make predictions for how changes in level of proteins, by variation, genetic lesion, or exposure to a drug will alter the population response. Here predictions are made from in silico adjustment and a comparison made to population data. Again no statistics is attempted for what makes a good fit, however, superficially we see similar trends although differences between details in the experiment and in silico dynamics are quite noticeable.
Much stronger tests of the predictions could have been shown. For example predicted changes to division and death times in each generation could be directly compared between data and model to strengthen the argument that the ODEs recapitulate the key drivers of the population behaviour.

In summary, this is on the one hand a paper with a big vision attempting a way forward in the difficult area of spanning three biological areas. It presents an impressive assemblage of skills, techniques, data and models. However, the novel scientific conclusions on which the work must be evaluated, delineating the action of NFkB in regulating subtle changes in division and death fates needs to be strengthened.

© European Molecular Biology Organization

Response to Reviews

We thank the reviewers and editor for evaluating our manuscript and for their enthusiasm regarding the scope, approach, and findings. In response to reviewers' comments we have been able to make substantive improvements in the manuscript, including in the logical flow and interpretation of the data, and clarifying the role of NFκB in the phenomenological cell fate decision.

Below we address questions and issues raised point-by-point.

Editor's summary:
... some of the major issues refer to the following:
- the way model parameters were estimated/chosen should be clarified and the impact of these choices should be analyzed.

In the revised manuscript we have included new supplementary tables (Tables E8, 9,10), which clarify which parameters were not transferred from the previous models but had to be fitted as well as the specific reactions we added/changed to link the models (Text E1). Some of these parameters were fitted to molecular data, and others were fit to emergent properties of the network or population response. We now clarify how each parameter was fit, describe and quantify the features being fitted, and show that the chosen parameter value is near a local minimum (Table E10, Text E1).

While a comprehensive parameter sensitivity analysis of this complex multi-scale model is certainly of interest, it requires the development of new approaches (as standard brute force approaches are not computationally feasible), and we respectfully submit that this is beyond the scope of the present study.

- model fits and predictions should be analyzed and tested more rigorously with appropriate statistics.

We have provided quantitation of how well the model simulations fit the available experimental data, and provided p-values to assess the statistical significance of the fits (Table E11). In addition, we have compiled new extended view text and tables highlighting the model reactions we parameterized, the features we were fitting, the success of the fit in terms of these features, and the effects of changing each free parameter in the model (Text E1, Tables E9 and E10).

Reviewer #1:

Major points

The key conclusion of this study is that B cell fate choice between division and death is pre-determined during the cell growth phase as demonstrated by time-lapse experiments, where in wild type B cells, the occurrence of division in growers was significantly higher than in non-growers (Figure 3). In addition, the authors suggested that cRel controls both cell growth and cell survival (Figure 4).

However, the empirical and simulation results of cRel knockout experiments were somewhat counter-intuitive to the conclusion. In cRel-knockout B cells, the growth profiles of B cells were not affected comparing to the wild type (Figure E7), but the fraction of death in growers was 4 times higher (Figure 6G). The results mean that cRel did not significantly control cell growth, but the choice between cell division and death. Therefore, cell fate decision is regulated independent of cell growth profile. Could the authors elaborate on their conclusions that "B cells may make a decision to enter a sustained growth phase that biases their fate towards division rather than death" in the abstract.

We agree with the reviewer that the results require nuanced discussion and we have elaborated on this. Our results indicate that some B-cells enter a sustained growth phase during which time they

are protected (though not fully) from cell death. This is what biases the fate of growers towards division rather than death, thus constituting phenomenologically a cell fate decision between division and death.
Our results show that NFκB is critical for protecting cells from cell death as cRel-deficient B-cells are much less protected during their growth phase. Thus our data indicates that the fundamental basis of the phenomenological cell fate decision is in fact a race of competing fates that NFκB severely biases.
NFκB may also play a role in making the decision to enter the growth phase per se, but cRel-deficiency merely causes a partially diminished mTOR activity but does not reduce the fraction of cells entering the growth phase. We do not want to rule out that NFκB or cRel is involved in making or executing the decision to enter the growth phase, as other family members, particularly RelA, may compensate. However, $cRel^{-/-}RelA^{-/-}$ hematopoietic precursors are severely defective in producing mature B-cells to begin with, so testing for the NFκB requirement to enter growth phase requires novel genetic tools, that we respectfully request to be considered outside the scope of the present manuscript.

It was difficult for me to determine if the authors model / data integration, especially as related to cell size aspects, was [corrected for] or required correction for concentration/ dilution effects due to volumetric normalization (i.e., were production rates adjusted for cell volume or was a molecular dilution component included). It would be helpful to more explicitly state how this was handled, wand there or not these parameters could be relevant.

The NFκB, cell-cycle, and apoptosis models assume that the concentrations of model species in the cytosol, mitochondria, and nucleus equilibrate quickly with respect to changing cell size throughout the cell-cycle. This is a simplifying assumption that appears reasonable given that cell mass/volume and the amount of general cellular machinery are highly correlated in the model in the cell-cycle model (Conradie, 2010), but future work may address its validity. We have clarified this in the methods section of the main text.

To demonstrate the causative relationship between sustained growth and cell division, the authors could disrupt cell growth profiles and quantify the frequency of cell division.

This is an excellent suggestion. We have used Rapamycin to disrupt growth. Indeed, Tdiv after rapamycin treatment is significantly delayed p-value < 0.0014, Mann-Whitney U test. However, probability that a grower died was still low: 6% normalized to a 24 h period. We now show the p-value in the results of the main text. This indicates that growth and cell size are determinants of entering the cell cycle (as encoded in the Tyson cell cycle model), but that survival signals do not require mTOR activity or sustained cell growth. Our multi-scale model recapitulates these control features.

Minor points

- Presentation and style

o I do not quite understand the percentages labeled on Figures 4F - 4K. Are those percentages for the 24h data?

The quadrants shown in the figure delineate the significance thresholds for cell size and average cell fluorescence for the indicated protein after 24 h as compared to the 0 h control. Percentages show the fraction of cells from the 24 h time point occupying each quadrant. Significant cell size was defined to be greater than 100 pixels manually, while significant abundance was defined as a value that is greater than or equal to the 95th percentile abundance from the 0h datasets. We have updated the methods description to clarify this for the reader.

- Trivial mistakes
o Page 5 "Interestingly, following the first generation, there was a significant..." the sentence needs to be revised. Done
o Page 7 paragraph 2 "we found that compared to a 0 h control, B cells were larger had higher cRel", check grammar Done
o Page 9 last paragraph, "next, we simulated the effect cRel-deficiency by setting the translation

rate of the cRel protein to zero" Done
o Page 11 paragraph 3, figures are missed referenced. Done

We thank the reviewer for pointing out these errors and we have fixed them in the revised manuscript.

Reviewer #2:

In a well-written article, Shokhirev et al. present a multi-scale approach that couples agent-based modeling with single-cell transcriptome and immunofluorescence analyses to elucidate the role of NFκB cRel in enforcing cellular decisions towards mutually exclusive fates. The paper seeks to answer an important biological question regarding cell fate -- do processes leading to cell-division and apoptosis proceed concurrently (molecular 'race' hypothesis) or is cell fate pre-determined ('decision' hypothesis that commits cells to either fate)? Although the main biological concepts/paradigms presented in this paper have largely been reported before, this manuscript does provide important contextual data supporting the 'decision' model, thereby arguing against the findings of a 2012 Science paper (Duffy et al., 2012) that supported the 'race' hypothesis. In addition, this work exhibits a very nice presentation of the systems biology approach - integrating single-cell experimental data with multi-scale computational modeling and demonstrating that these complex models can in fact be predictive.

We thank the reviewer for their appreciation of this systems biology study.

In general, the conclusions are well supported and the work is of significant interest to the readers of Molecular Systems Biology. A few minor issues/questions should be addressed:

1. The lines and arrows as well as the time distributions in Figures 3A and 3B are a bit confusing. Should Figure 3A include the early branching? Given that these figures highlight the main biological question on cell fate determination, it might be worthwhile to clarify or re-draw.

We agree with the reviewer that a non-branching version of Figure 3A would represent the simplest version of the race hypothesis. However, our figure represents the special case where some cells have entered a growth phase, which is a pre-requisite for division. The two branches represent growers and non-growers. We have edited the figure legend to clarify this point.

2. How sensitive are the results to the cell size thresholds chosen? What about the cells that were just outside of the chosen bounds? Are these the cells that end up dividing in Fig. 3D (cells that divide in the population of "non-growers"?)

This is a valid concern. We have repeated the tracking analysis for the wildtype dataset using a more relaxed growth threshold of 1.6 x the standard deviation (280 um^3) and a more stringent 2.5 x the standard deviation (438 um^3). While there are some differences in the number of cells classified to be growers or non-growers for the later generations (4+), as expected given that late generation responders tend to grow less on average (Figure E2), there are only a few cells switching classification in the first few divisions. Given that cell quantification is relatively noisy (Figure 3D) and that cell classifications do not change drastically across a large range of growth thresholds (280-438 um^3), we feel that our original choice for the growth threshold of 2x the standard deviation about the starting size is appropriate. We have included the following graph summarizing this analysis into the extended view figures (Figure E1 Panel E).

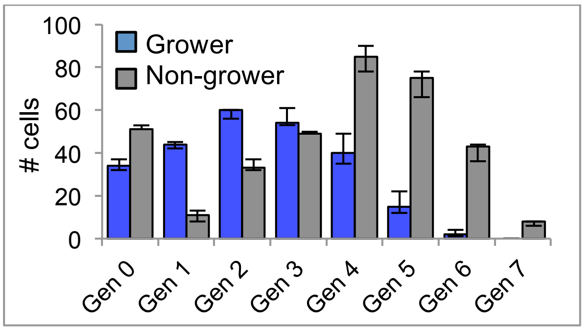

Sensitivity of cell classifications in each generation to the growth threshold for the wildtype 250 μM CpG condition. Bars show the number of cells classified given a 350 μm^3 threshold, and error bars show cell counts for a threshold of 438 μm^3 (more stringent) and a 280 μm^3 threshold (less stringent)

3. Some care should be taken when describing the "molecular determinants of cell fate decision." Shokhirev et al. highlight several upregulated and downregulated genes in large vs. small cells. Since the cells are already 'large' versus 'small', it should be noted that the upregulated genes did not necessarily cause the cell fate decision. The upregulated genes could be another phenotype of something up-stream that triggered the cell fate decision. An explicit discussion of this might be useful to the readers.

We agree with the reviewer that our molecular analysis does not identify how the decision to enter growth phase is made, but that NFκB is involved in executing the downstream decision to die or divide. The loss of cRel does not diminish cells entering growth phase, but it renders cells sensitive to death such that the decision to enter growth phase no longer constitutes a decision between the fates division vs. death. This analysis reveals that the phenomenological cell fate decision is in fact based on a cell fate race that cRel biases in growing cells.
Future work should address how the decision to enter the growth phase is made. It may involve NFκB, but this is at present unclear. We have clarified our interpretation and discussion in the revised text.

4. In the multi-scale model, values for certain free parameters specifying BclXL, CycD, and Myc transcript synthesis and degradation were manually selected from within 'biologically plausible' ranges? How was 'biologically plausible' defined here? How different were these ranges? Given the importance of these parameters to obtaining the model fits, it would be helpful to highlight them by separating them out from the full parameter table.

We agree that this is an important point, and we have addressed it in the revised manuscript. We now include supplementary text and tables (Text E1) describing the model construction, parameters that were not transferred from the previous models but had to be fitted, the reactions affected by these parameters, the biological features we were attempting to fit, the allowed ranges for each free parameter, the features we were not able to fit, as well as a basic sensitivity analysis describing how the model features are affected by 10% changes in each of the parameters. We point interested readers to these supplementary descriptions in the revised manuscript.

5. In Figure 2E, the authors noted that progenitors that grew exhibited an initial growth delay followed by rapid growth. Meanwhile generation 1+ growers did not exhibit any delay phase. In Figure 6, the authors note that model simulations suggest that cell growth is not cRel-dependent. Through model perturbations, could the authors delineate what might be causing the initial growth delay in pro-genitors? Some clarification here would be helpful to better understand the model.

The current model (Conradie *et al.* 2010) does not readily recapitulate the observed initial delay in growth, but this characteristic is included as an explicit delay in the connectivity between NFκB and cell cycle modules via NFκB controlled Myc activation. It is possible that refinements of the models for the NFκB and cell cycle modules might obviate the need for such a delay, but such a comprehensive re-parameterization would be an exercise in over fitting. Our goal in this study was not to produce the best fit, but to determine whether existing models taken "off the shelf" and not necessarily tailored to B cells or our experimental conditions might recapitulate key features of the dynamic population response. The initial delay is not one of them and further work is required to examine the underlying molecular mechanism. We have clarified this in the Discussion.

© European Molecular Biology Organization

6. In Figure 7, the authors showed that the probability of observing dying cells that had grown tripled when cells lacked cRel. However, this was not seen under rapamycin treatment (which results in defective cell growth). The authors explain this by stating that cRel's function may be to enforce a decision to divide and that the population response of cRel-deficient cells resembles a molecular race model. The authors should expand on the discussion regarding the specific role of NFκB here for further clarification.

In the revised manuscript we have clarified the discussion regarding NFκBs and cRel's role in controlling the growth phase entry, and in rendering this step a decision between the two cell fates of division and death. See also our response to point 3.

7. Why are error bars only included in Fig. 8F and 8J and not in the other panels?

We are sorry for the confusion. Panels D,E,G,H,I,K are quantifying a single feature of each simulation. Since we cannot assume a particular null distribution, to obtain error bars we would have to repeat each simulation many times (hundreds to thousands) to learn the underlying distribution for each of the measured statistics. As this would involve considerable additional computational time, analysis, and discussion, we respectfully suggest that this type of analysis should be carried out as part of a subsequent study on the sensitivity of the model predictions. We clarify this point in the figure legend, new methods subsection, and have updated the y-axis labels in the figure to minimize confusion. We also include the calculation of the expected number of progenitor divisions in the extended view text.

8. References to Fig. 8 in the last paragraph of the Results section are incorrect.

We thank the reviewer for pointing this out, and we have fixed it in the revised manuscript.

9. In the text discussion of the IF in "big" cells versus all cells, are the modest increases in % reported for big cells significant?

The comparison to 0 h within each panel is based on significance. The definition of significance here is in the 95th percentile in the 0 h data. Cells that are in the non-double negative quadrant are significantly different at 24 h in one or more features (size and/or abundance). Since the fluorescence and size of the population is dependent on many experimental and biological variables, we use these data (qualitatively rather than quantitatively) to determine if there is dependence of key proteins on NFκB cRel and TOR and/or on being in the growth phase or not.

Reviewer #3:

This is an ambitious paper attempting to connect dots across three organisational levels. The strategy at the heart of the paper, that information from top down can inform the selection of possibilities in a bottom up model and vice versa, is sound. However, it is certainly not easy to get ones head around all the parts, and ultimately it is held together with a good dose of faith in the author's judgement in setting the parameters for a large number of ODEs.

We thank the reviewer for their appreciation for our study. In the revised manuscript we have attempted to clarify the logic and how different parts of the paper hang together. We have also clarified our interpretation of the decision vs. race model.
To alleviate potential concerns about the number of parameters, we have clarified in the revised manuscript that only a handful of reactions that connect previously established models needed to be parameterized and we provide the details on how this was done, and an indication of their relative sensitivity. These can be found in additional supplemental Figures, methods, and text, as detailed below.

There are four sections that hold up the logical fabric:

1. The paper begins with imaging of B cells following stimulation with CpG. Tracking and following

cells is performed with new software for manual guided extraction of data. I have not evaluated these - but the code is provided to do so. The data produced seems similar, to the work of Hawkins et al (PNAS, 2009). The main purpose of the data is to develop a population based parametric model - or at least to choose between two alternatives - the competing 'race' model of Hawkins et al. (PNAS 2007) and the 'decision model' (for example Hyrien et al. Biology Direct, 2010). By comparing the predicted proportion of cells that grow and die the decision model is selected. The logic seems to hinge on two observations - 1. The data from figure 3D showing that, at least for the first four generations, all 'growers' went on to divide; and 2. Having shown, paradoxically, that some non-growers divide, a test is developed to evaluate whether they might have been also able to 'die' based on the distribution of times to die. However, this latter proof seems to be applied only to generation 0 cells in the figure and in the supplementary description. The authors should clarify this second argument and confirm it works as well for each generation.

In the revised manuscript we have attempted to provide a clearer description of our analysis. First, we have shown that some cells but not others enter a growth phase, as defined by a metric whose reliability is obviously limited, leading to both false negatives and false positives. In response to R2, point 2, we show how changes in the metric may change quantitative aspects of the response, though the qualitative conclusions remain consistent. Our data also shows that in subsequent generations the number of cells entering a growth phase is diminished; this may be a reflection of the stringency of the metric to ascertain a growth phase, but it is likely also reflective of the biology, where the penultimate generation often divides the pre-existing mass without adding much to it. This underlies the data in Figure 3D.
Second, we show that entering the growth phase predisposes a cell towards division rather than death, and thus constitutes a cell fate decision. (This is not the case in cRel-deficient cells, in which the growth phase does not protect from death.) Figure 3C shows that this is true for 3-4 generations in our cell growth conditions. However, the analysis shown in Figures 3 E and F can only be done reliably for generation 0 cells as it relies on the fact that Tdiv is typically later than Tdie and that all cells are accounted for prior to division or death. (This does not hold true for later generations as some cells will be entering a particular generation while others have already divided into the next one).

Returning to the first argument the authors should explain why they focus on the first four generations and not the latter two, which look to be giving a different answer. Curiously Fig. 3F also seems to suggest that perhaps neither model is giving the right answer and further alternatives could be developed.

We agree with the Reviewer that the generations appear to behave differently, as evident particularly in Figure 3D. We find that a number of cells identified as non-growers divide in subsequent generations. This may be a reflection of the stringency of the metric to ascertain a growth phase, but it is likely also reflective of the biology, where the penultimate generation often divides the pre-existing mass without adding much to it. The fraction of cells undergoing their final division is greater in later generations and thus a greater number of cells scored as “non-growers” are shown to divide.
Figure 3 F provides support for the model in which entrance into a growth phase represents a decision between the cell fates on death and division. This decision (or its execution) is leaky to some degree, but the data is more consistent with the decision vs the race model. In contrast, cRel-deficient B-cells behave more similar to a race model (Figure 7D), indicating that cRel plays a role in the execution of the decision.

The authors then turn to using a population-based version of the two models to apply to CFSE time series data, again to help determine which might be the better description of events. Here, no full statistical evaluation is given (or at least it wasn't used to distinguish them). Both models seemed to do a very good job of fitting to the dividing CFSE populations, but the race model was clearly unable to accommodate the rather large level of death in generation 0. Hence, again, the decision model was chosen.

Coming to the end of this section, and knowing what is coming, I was left wondering why so much time was spent establishing this model. Both models tested are published and either could simply have been adopted, with little consequence, one or the other for the subsequent work. If the authors genuinely want to evaluate and develop a 'correct' model of the cellular dynamics then much more

© European Molecular Biology Organization

work would need to be done on the data sets, and more sophisticated models than used here could be developed and compared with appropriate statistical evaluation, taking account of the number of parameters being exploited and the quality of the fits to the complete data sets. This point is brought up again in the last results section where c-Rel loss is used to illustrate that in fact some race for fates might be occurring, although not at the level of the simple model that assumes complete independence of division and death times. Clearly the situation is not resolved, and this work manages to point out deficiencies in all models to date, although that is not stated by the authors.

We agree with the Reviewer that at a fundamental level there is a race for fates, one that is skewed by the function of cRel, which is primarily activated in growing cells (Figure 4) and protects then from death Fig.7). Hence, the phenomenological description of a decision is based on the observation that entry into a growth phase predisposes cells to division rather than death. We have clarified our interpretation of the data in the text. This is also reflected in minor edits of the abstract and title.
The multi-scale modeling represent an effort to understand molecular basis of the phenomenological decision, and this led to analysis of the cRel-deficient B-cells. In the revised manuscript, we have clarified the logical sequence.

2. The second section aims to identify the key molecular determinants of cell fate decisions by single cell analysis. This is done using fluidigm and imaging to evaluate mRNA expression in 24 h small versus large cells. Only 5 cells of each are tested. There does not seem to be any additional value in the single cell approach as the data simply reveals broad differences in expression of fairly well known targets of the CpG pathway. Perhaps the authors can provide more discussion of why they went this route, and again, why it was necessary as published studies of NFκB could have been used to justify the use of the NFκB pathway to drive cell growth and survival in the agent based model development in the next section. A stronger and clearer argument for what particular result is new here, and is used to inform the next section would be helpful.

The Reviewer is correct in that the literature identified NFκB as a key determinant of B-cell expansion. However, what these studies did not show is whether NFκB is activated in all cells and the decision to enter growth phase and undergo cell division is made by a downstream signaling network, or whether the decision is made upstream of NFκB and hence NFκB is activated primarily in growing cells. Our single cell analysis provides evidence that is consistent with NFκB executing an upstream decision rather than providing a gene expression program for a downstream decision. This result informed how we established the model and the use of extrinsic noise in the input for the NFκB model. We have clarified this in the revised manuscript.

3. The third section builds a very complex agent-based model for B cell division, death and signalling that stitches together published ODE models of NFκB signalling, apoptosis and cell cycle control. It is claimed that some variations, such as a link to Myc to promote cell growth, were implemented in a new way.

This section is in need of more depth in evaluation and description. The number of parameters governing each agent is astonishing (>200) and there is almost no discussion of how sensitive the model is to various changes or conditions of these components that make up the model. Most of the adjustment in the model seems to be in changing dynamics of only three components (Myc, Bclxl and CycD). Figure 5C is described as a remarkable recapitulation of the observed cellular dynamics and yet, to my eye, it looks quite different and we are left with this subjective manner of evaluation. Why not fit to the data? Granted this agent-based model method is not well suited to classical statistical approaches making it difficult to evaluate. We are asked to take a leap of faith that the various parameter ranges are reasonable and the model is giving a qualitative 'fit' based on a reasonable re-creation of the underlying molecular processes. It is virtually impossible to know if this is correct without predictions and careful evaluation, which is attempted in the last section.

We understand the reviewer's concerns. In the revised manuscript we have included several additional tables and supplemental descriptions, which clarify which parameters were not transferred from the previous models but had to be fitted. Some of these parameters were fitted to molecular data, and others were fit to emergent properties of the network or population response.

We now clarify how each parameter was fit, describe the features we used to fit the model, and show that the chosen parameter value is near a local minimum by showing that 10% changes in each parameter lead to poorer solutions (Table E9).
With a model of this complexity, a comprehensive re-parameterization would be an exercise in overfitting. Thus our goal in this study was not to produce the best fit, but to determine whether existing models taken "off the shelf" and not necessarily tailored to our experimental conditions might recapitulate key features of the dynamic population response. We have now provided a measures/statistics for the fit, and point out features that are not well recapitulated by the model.

4. In the fourth section agents from section 3 are used to make predictions for how changes in level of proteins, by variation, genetic lesion, or exposure to a drug will alter the population response. Here predictions are made from in silico adjustment and a comparison made to population data. Again no statistics is attempted for what makes a good fit, however, superficially we see similar trends although differences between details in the experiment and in silico dynamics are quite noticeable.
Much stronger tests of the predictions could have been shown. For example predicted changes to division and death times in each generation could be directly compared between data and model to strengthen the argument that the ODEs recapitulate the key drivers of the population behaviour.

We agree with the Reviewer that the model can provide a wealth of predictions that in principle are experimentally testable. We submit that much of that work is beyond the scope of the present study, which focuses on the relationships between growth, death and division, and the molecular underpinnings of what appears to be a cell fate decision process.
However, given the availability of a multi-scale model we hoped to illustrate its utility by generating some predictions. We provide in the revised manuscript experimental tests for some prediction in Figure E7. We also provide statistics for the quality of the fitting, and plot residuals highlighting areas of good and poor agreement between the model and dataset (Table E11). This only scratches the surface of what can be done, and we imagine that the present study will catalyze subsequent systems biology studies of lymphocyte dynamics.

In summary, this is, on the one hand a paper with a big vision attempting a way forward in the difficult area of spanning three biological areas. It presents an impressive assemblage of skills, techniques, data and models. However, the novel scientific conclusions on which the work must be evaluated, delineating the action of NFκB in regulating subtle changes in division and death fates needs to be strengthened.

We thank the Reviewer for this evaluation and the suggestions. We have clarified our interpretation of the data and the flow of the work. The decision model fits the phenomenological data in Figure 3. The single cell experimental work in Figure 4 informs our simulation methods of the multi-scale model. This leads to predictions, including that cRel-deficient B-cells are not protected from death when growing (Figure 7) revealing that the fundamental basis of the phenomenological decision is in fact a race model in which cRel biases cell fates in growing cells.

2nd Editorial Decision 26 November 2014

Thank you again for submitting your revised work to Molecular Systems Biology. We are now globally satisfied with the modifications made and I am pleased to inform you that we will be able to accept your manuscript for publication pending the following minor changes:

- We would be grateful if you could include the key data used in this study as dataset file, including the features extracted from the imaging experiments and the flow cytometry data. For the single-cell imaging, it would be good to provide the data (for example as "Source Data for Figure 1") in a format that allows others to re-analyze the full distribution of the extracted features, including the main measurements used in this study (cell volume, generation index, time to grow, time to die, time to divide, etc...). This could also be important to for the data that are directly compared to simulation results and for the data of the cRel deficient cells (Fig 7).

- Ideally, and only if it reasonable, the full set of images could be deposited to Dryad and the respective DOI included in a "Data availability" section at the end of Materials & Methods.

- Please deposit the single-cell functional genomics data to GEO or ArrayExpress and include the respective accession number in a "Data availability" section at the end of Materials & Methods.

2nd Revision - authors' response 21 December 2014

- We would be grateful if you could include the key data used in this study as dataset file, including the features extracted from the imaging experiments and the flow cytometry data. For the single-cell imaging, it would be good to provide the data (for example as "Source Data for Figure 1") in a format that allows others to re-analyze the full distribution of the extracted features, including the main measurements used in this study (cell volume, generation index, time to grow, time to die, time to divide, etc...). This could also be important to for the data that are directly compared to simulation results and for the data of the cRel deficient cells (Fig 7).
- Ideally, and only if it reasonable, the full set of images could be deposited to Dryad and the respective DOI included in a "Data availability" section at the end of Materials & Methods.

While the large size of our time-lapse datasets prohibited upload to Dryad, all of the source images from our time-lapse microscopy have been uploaded to our permanent webserver (http://www.signalingsystems.ucla.edu/max/) and made available for public download along with the software we used to process the images. Alongside, we have included the CFSE flow cytometry datasets used in the study and supplementary files. This is now summarized at the end of the Methods section under "Data availability."

- Please deposit the single-cell functional genomics data to GEO or ArrayExpress and include the respective accession number in a "Data availability" section at the end of Materials & Methods.
- The RNA-seq data should be deposited in an appropriate functional genomics public repository. Please include the accession number in a Data availability section at that end of the Materials & Methods section.

We have also uploaded the single-cell RNA-seq data to GEO (GSE64156) and provide the accession number and link in the "Data availability" section.

© European Molecular Biology Organization
