# Supplementary material for: A multi-scale approach reveals that NF-κB cRel enforces a B-cell decision to divide
Source: Mol Syst Biol. 2015 Feb 13;11(2):783. doi: 10.15252/msb.20145554 (PMC4358656; doi:10.15252/msb.20145554)
Supplement: Supplementary file 18 [file msb0011-0783-sd18.zip › CFAnalyzerReadme.pdf]

Cell Fluorescence Analyzer Basic User Guide

1. Launch the runnable jar file: CFAnalyzer.jar
2. Add some channels by clicking the Add Channel button top right.
    a. A pop-up dialog should prompt you for the channel name each time.
3. Next, load some images by clicking the "Load Images" button
    a. A file dialog box should pop up, allowing you to select the images (PNG only for now)
    b. The images will be sorted into their respective channels in alphabetical order:

Example:

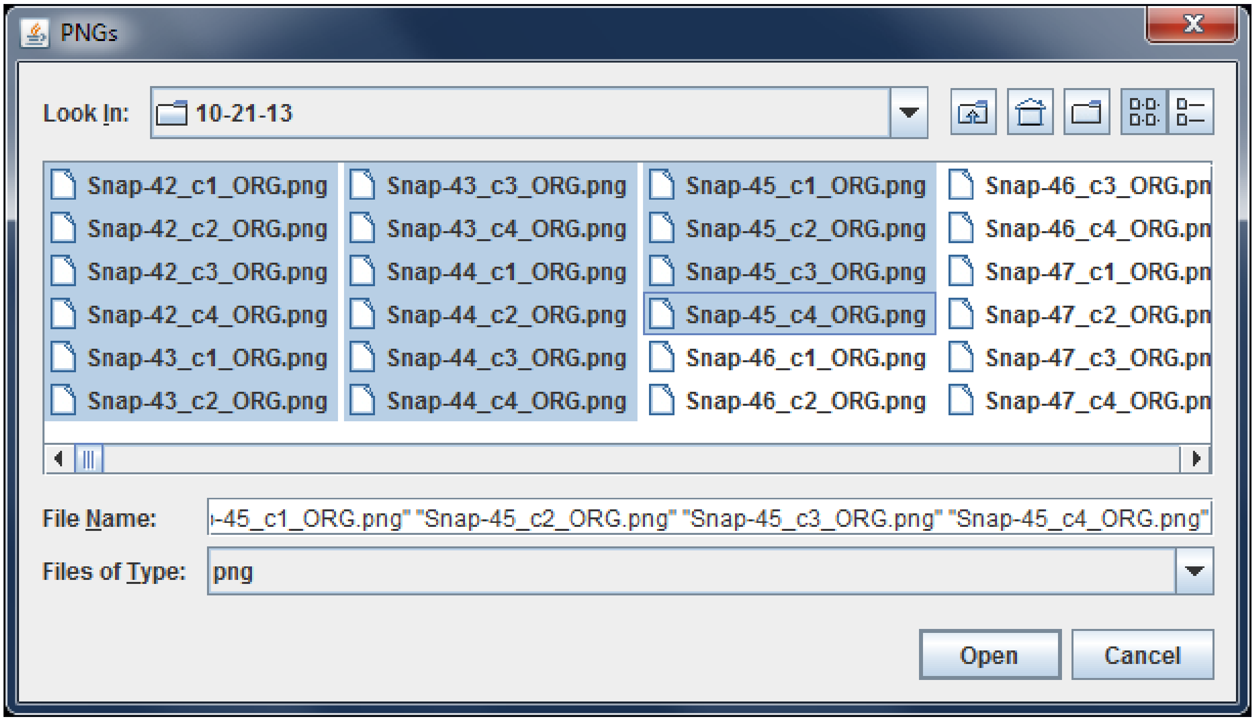

The images will load into the four channels created:

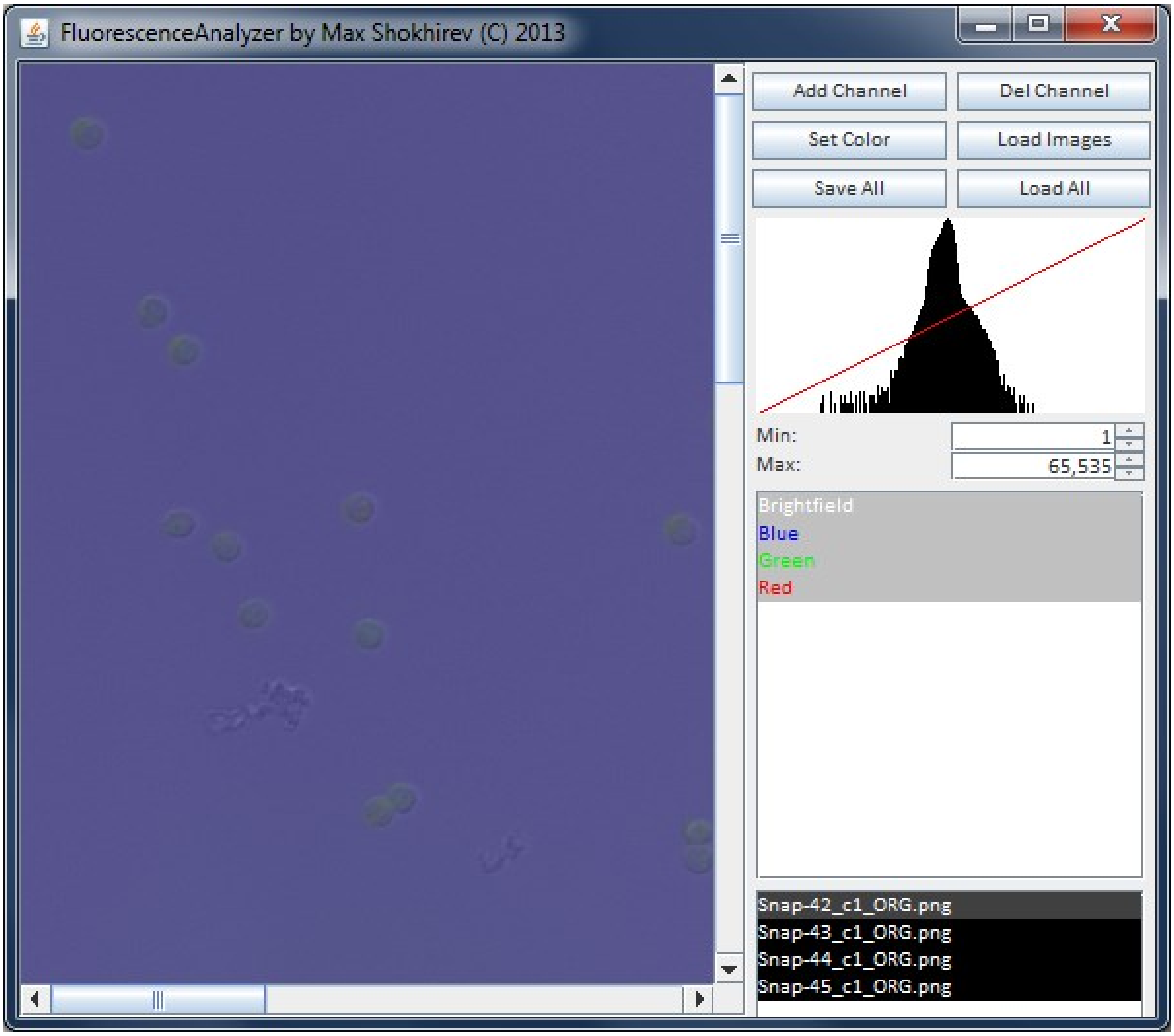

4. The minimum and maximum channel fluorescence values are plotted as a line through the histogram for each channel. Clicking on each image name should update the histogram.
    a. The minimum and maximum values can be changed by the number spinners or by clicking on the histogram directly
5. The selected channel's color can be changed using the "Set Color" button
    a. A separate dialog box will pop up allowing you to click on a color (use the mouse wheel to change the brightness)
6. Multiple channels can be rendered in their respective colors simultaneously by selecting more than one channel from the list.

Selecting cells

Cell segmentation is based on edge detection within the image. If cells are poorly detected, try changing the minimum and maximum values to ensure sufficient contrast in the DIC channel.

Left clicking while holding Ctrl in the vicinity of each cell will do the following:

1. Circle candidates are found by polling each edge pixel (Hough Transform).
2. The best candidate is selected based on the fraction of overlapping edge pixels and its size
3. Vertices are then optimized in an area-preserving manner using a physics-based optimization

You can add placeholder cells which will be ignored during processing (i.e. debris) **by left clicking while holding Shift.**

Press w/s to increase/decrease the area of the candidate (reoptimized). This can also be done by scrolling with the mouse scroll wheel.

Right click to select cells

Selected cells can be deleted with [Delete].

Pressing M will renormalize the fluorescence values to +/- 8 standard deviations about the mean (good for DIC images)

Pressing N will normalize fluorescence images to (+2 sd above mean , mean+40,000), ideal for fluorescence channels.
